# Deep-time gene expression shift reveals an ancient change in avian muscle phenotypes

**DOI:** 10.1371/journal.pgen.1011663

**Published:** 2025-04-11

**Authors:** Christina M. Harvey, Matthew J. Fuxjager, James B. Pease

**Affiliations:** 1 Department of Biology, Wake Forest University, Winston-Salem, North Carolina, United States of America; 2 Department of Ecology, Evolution, and Organismal Biology, Brown University, Providence, Rhode Island, United States of America; 3 Department of Evolution, Ecology, and Organismal Biology, The Ohio State University, Columbus, Ohio, United States of America; Michigan State University, UNITED STATES OF AMERICA

## Abstract

Gene duplication is an important process of molecular evolutionary change, though identifying these events and their functional implications remains challenging. Studies on gene duplication more often focus on the presence of paralogous genes within the genomes and less frequently explore shifts in expression. We investigated the evolutionary history of calsequestrin (CASQ), a crucial calcium-binding protein in the junctional sarcoplasmic reticulum of muscle tissues. CASQ exists in jawed vertebrates as subfunctionalized paralogs CASQ1 and CASQ2 expressed primarily in skeletal and cardiac muscles, respectively. We used an enhanced sequence dataset to support initial duplication of CASQl in a jawed fish ancestor prior to the divergence of cartilaginous fishes. Surprisingly, we find CASQ2 is the predominant skeletal muscle paralog in birds, while CASQ1 is either absent or effectively nonfunctional. Changes in the amino acid composition and electronegativity of avian CASQ2 suggest enhancement to calcium-binding properties that preceded the loss of CASQ1. We identify this phenomenon as CASQ2 “synfunctionalization,” where one paralog functionally replaces another. While additional studies are needed to fully understand the dynamics of CASQ1 and CASQ2 in bird muscles, the long and consistent history of CASQ subfunctions outside of birds indicate a substantial evolutionary pressure on calcium-cycling processes in muscle tissues, likely connected to increased avian cardiovascular and metabolic demands. Our study provides an important insight into the molecular evolution of birds and shows how gene expression patterns can be comparatively studied across phylum-scale deep time to reveal key evolutionary events.

## Author Summary

Duplicated genes adopt different functional relationships that can directly influence physiological processes. Calsequestrin-1 and calsequestrin-2 are two similar proteins expressed from genes that bind calcium needed for strong muscle contractions in vertebrate muscle tissue. We compared gene sequences and expression across all chordates and found that CASQ1 and CASQ2 have specialized functions in the skeletal muscles and heart, respectively, in most vertebrates. However, birds only express CASQ2 in both the heart and skeletal muscles, indicating a shift in muscle function on the molecular level. Furthermore, CASQ1 had its gene silenced or deleted in birds. Our analysis discovered an example of a rarely reported gene family functional change and influences our current understanding of avian evolution and muscle physiology.

## Introduction

Gene duplication, loss, and dosage changes are fundamental processes of genome evolution with the potential to cause major shifts in traits and organismal functions [1–4]. Duplicated genes undergo a variety of changes in their functional roles, including specialization (subfunctionalization), evolution to new functions (neofunctionalization), and compensatory reactions to gene loss [4–7]. Duplicated genes also modulate expression as dosage balance, compensatory drift, and hypofunctionalization [6,7]. Investigations of both the presence and absence of gene duplicates and their relative expression are therefore crucial to understand the impact of these molecular evolutionary processes on key fitness-related phenotypes.

**Fig 1:**
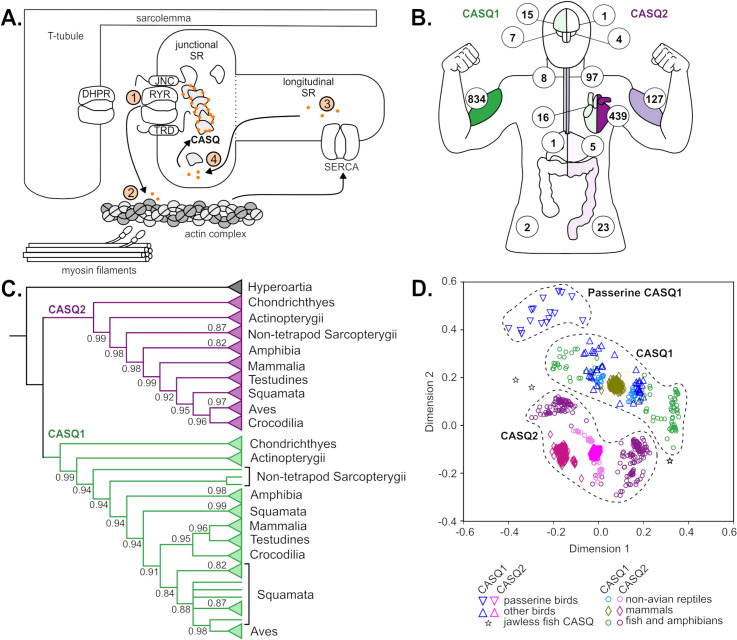
Calsequestrin duplicated in the jawed vertebrate ancestor into two distinct proteins. A) Calsequestrins bind Ca^2+^ ions (orange dots) in the junctional sarcoplasmic reticulum, either in monomeric, dimeric, or polymerized (as shown; DHPR: dihydropyridine receptor; JNC: junctin; TRD: triadin; RYR: ryanodine receptor; SR: sarcoplasmic reticulum; SERCA; sarcoendoplasmic reticulum ATPase). B) Average *CASQ1* and *CASQ2* expression in human tissues from the Genotype-Tissue Expression (GTEx) dataset. Expression of tissue-specific paralogs is represented in transcripts per million reads (TPM) and by the saturation of green (left, CASQ1) or purple (right, CASQ2) relative to blue (no CASQ expression). Values (from top to bottom) depict brain cortex, cerebellum, esophagus muscularis, heart, stomach, skeletal muscle, and transverse colon. C) Gene family cladogram of preduplication CASQ (black), CASQ1 (green), and CASQ2 (purple) amino acid sequences supports a duplication in a jawed vertebrate ancestor. Polytomies were created for branches with a support value of 0.8 or lower. Bootstrap values of 1.0 are not shown. Triangles depict collapsed clades. D) A multidimensional scaling plot of calsequestrin amino acid pairwise sequence distances shows separate, non-intercalated CASQ1 and CASQ2 clusters.

Calsequestrin (CASQ) proteins are the most abundant Ca^2+^-binding proteins in the junctional sarcoplasmic reticulum (jSR) of vertebrate cardiac and skeletal myocytes, forming compact, high-capacity polymers when Ca^2+^ concentrations are high (Fig 1A) [8–13]. While CASQ is one of many components in myocyte calcium cycling, even minor CASQ mutations have been shown to cause lethal stress-induced myopathies in skeletal and cardiac tissues in humans, mice, and chickens [14–18]. Evidence from these and other studies directly demonstrates that calsequestrin is critical to core vertebrate muscle contraction necessary for maintaining high muscle performance [19].

Two subfunctionalized CASQ proteins are present in most vertebrates: skeletal muscle-specific CASQ1 and cardiac muscle-specific CASQ2 (Fig 1B) [20–23]. In mammals, smooth muscles (e.g., the esophagus) may also express low levels of CASQ2 and the cerebellum expresses a small amount of CASQ1 [24,25]. Mammalian CASQ1 and CASQ2 exhibit different calcium-binding capabilities, calcium-sensing thresholds, and proximal interactions with calcium-release channels [14,19,21,26,27].

Previous studies have disagreed on the evolutionary timing of *CASQ* gene duplication and debated the presence of *CASQ1* in cartilaginous fishes, crocodilians, and birds [13,22,28]. In recent studies of molecular expression in Passerine bird muscles, we explored patterns of gene expression in core muscle function genes [29,30]. CASQ1 was among the candidate genes targeted in a PCR amplification study from zebra finch and whole transcriptome analyses of six manakins and a flycatcher, but in neither case could CASQ1 be found in the genome or transcriptomes of these species, respectively [29,30]. The absence of CASQ1 was puzzling because experimental deletion or mutation of *CASQ1* has been shown to alter muscle structure and function in mammals, and presumably the loss of *CASQ1* in other vertebrates would imply significant functional changes in skeletal muscle calcium-cycling processes [31,32].

Synfunctionalization is a rarely reported evolutionary process where one gene takes over the functional role of another paralogous gene [33,34]. The synfunctionalization event must necessarily be preceded by sub- or neo-functionalization where paralogs have subdivided or expanded their functional roles into separate spaces, such that one paralog can assume the function of the other later. After synfunctionalization, a paralog made redundant could later experience pseudogenization or deletion without selective consequence for the traits influenced [33,34]. The maintenance of high muscle performance in Passerine birds despite the apparent loss of genetic inactivation of *CASQ1* led us to hypothesize the potential for synfunctionalization in this case. Observing additional cases of synfunctionalization, especially in a core protein family with the functional impact of calsequestrin, would greatly contribute to our understanding of this gene family’s evolutionary process [34].

In this study, we conduct a deep-time analysis spanning a comprehensive set of chordate calsequestrin sequences and relative expression levels from hundreds of muscle expression profiles. Our results support the presence of *CASQ1* and *CASQ2* genes in all jawed vertebrate groups and a highly conserved pattern of *CASQ1* expression in skeletal muscles and *CASQ2* in cardiac muscles. Birds are an exception to this pattern by expressing a functionally enhanced *CASQ2* as their primary jSR calcium binder in both cardiac and skeletal muscles. We confirm that avian *CASQ1* genes exist, but sequence analysis indicates they are functionally inadequate, pseudogenized, or deleted. We conclude that the combined sequence and expression evidence show CASQ2 has displaced its paralog CASQ1 in an avian ancestor, under a model of paralog synfunctionalization. We also discuss the implications of CASQ synfunctionalization in the context of underlying evolutionary processes and in relation to changes in cell function and physiology in avian ancestors. Our results reinforce that deep-time comparative expression analysis at the class or phylum scope can reveal a markedly different sequence of evolutionary steps than gene presence-absence analyses alone and yield valuable functional insights [35–39]. More broadly, our results show a complex series of evolutionary events that integrate genomic, transcriptomic, structural, and organismal biology connecting changes in gene duplications and gene family expression to core animal physiological traits.

## Results and discussion

### Calsequestrin duplicated in the jawed vertebrate ancestor

We confirmed that calsequestrin is an ancient metazoan protein, distantly related to proteins with calcium-related functions in the endoplasmic reticulum. We identified 1248 calsequestrin genes (1137 chordate, 111 others) from 770 animal species (679 chordate, 91 others), including chordates, placozoans, hydra and corals, nematodes, arachnids, a sponge, and a tardigrade (S1 Fig, and Tables A and B in S1 Data). A phylogeny inferred from 1071 chordate CASQ sequences shows CASQ1 and CASQ2 are each monophyletic, cluster separately in a protein sequence-based multidimensional scaling plot (MDS) and are roughly equivalent amino acid distance from the jawless fish single *CASQ* sequences (Fig 1C and 1D). These features are consistent with a model of a single duplication event generating *CASQ1* and *CASQ2* as separate paralogs that each have remained orthologous through all extant vertebrates. All bony fish have at least three or four CASQ paralogs and salmonids have eight, congruent with inferred whole-genome duplication (WGD) events in their evolutionary history [40,41]. Another phylogeny from 111 non-chordate CASQ-like sequences indicates no duplications outside chordates (S2 Fig).

The presence of a single calsequestrin in all jawless chordates and two copies in all non-avian jawed vertebrates establish evidence that the most likely duplication timing of *CASQ* is in a gnathostome ancestor, potentially synchronous with the “2R” WGD event (Fig 1C) [42]. *CASQ1* and *CASQ2* both have 11 exons with nearly identical exon lengths and intron-exon boundaries for nearly all annotated genes, which further supports a DNA-based duplication. Additionally, *CASQ1* and *CASQ2* are each closely physically linked to several other genes that also exist in a few copies in gnathostomes but only a single copy in jawless chordates (*VANGL1*/*VANGL2*, *NHLH1*/*NHLH2*, *DCAF6*/*DCAF8*, and *ATP1A1*/*ATP1A2*). The presence of multiple linked duplicated genes in proximity to each copy of calsequestrin is further suggestive that calsequestrin was duplicated as part of a large-scale duplication or WGD event. However, *CASQ* remains chromosomally unplaced in lampreys and its neighboring genes in tunicates, lancelets, and hagfish are disparately located in gnathostome genomes. *CASQ1* is not placed in a clear chromosomal duplication block in reconstructions of the ancestral vertebrate linkage groups, preventing a more definitive phylogenetic placement of its duplication [43–45].

### Cardiac calsequestrin has replaced skeletal muscle calsequestrin in birds

**Fig 2:**
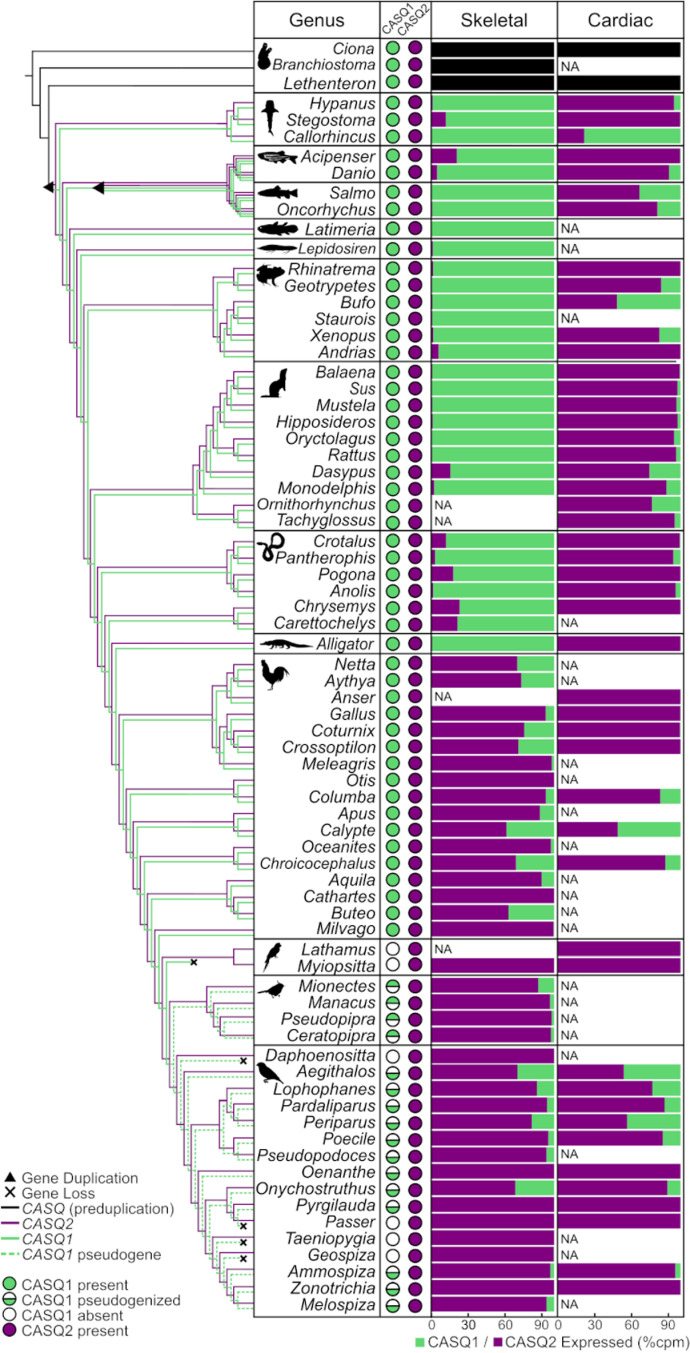
Deep-time comparison of CASQ1 and CASQ2 relative expression shows a shift in birds. RNA expression values of pre-duplication CASQ (black), CASQ1 (green), and CASQ2 (purple) as the percentage of counts per million reads out of the total calsequestrin expression for the skeletal muscle (left column) and cardiac muscle (right column). “NA” is shown for groups where RNA expression data for the given tissue sample was not available. Circle icons indicate the presence (filled), absence (empty), or functionally pseudogenization (half-filled) for each gene. Triangles on the parallel cladograms for CASQ1 (green) and CASQ2 (purple) indicate gene duplication events, and X’s indicate gene loss.

CASQ expression levels from 414 RNA-seq profiles from 93 diverse chordate species show that skeletal muscles predominantly expressed CASQ1 and cardiac tissues CASQ2 in all jawed vertebrate lineages, except birds (Fig 2). The surprising exception is that all bird transcriptomes we sampled expressed CASQ2 as the major form of calsequestrin in both skeletal and cardiac muscle tissue. CASQ1 transcripts are present in many bird species, though the average skeletal muscle expression of CASQ1 in those species is low (83 counts per million; cpm). Levels of CASQ2 expression in skeletal muscles in birds are robust (up to 6322 cpm) and 2.5 times higher than CASQ2 levels in cardiac tissue, on average (Tables C and D in S1 Data). For comparison, the average mammalian CASQ1 expression is 3836 cpm in skeletal muscle tissue, representing over 99% of the total calsequestrin expressed in those tissues. We also confirmed that other calcium binding proteins found commonly in the SR do not noticeably increase or decrease in expression in birds compared to other tetrapods (e.g., CALM, HRC, CALR, PVALB). Furthermore, birds, non-avian reptiles, and mammals all express ryanodine receptor-1 in skeletal muscles and ryanodine receptor-2 in cardiac muscle, meaning other key molecular components of the jSR do not appear to have noticeably changed their expression patterns.

The expression of CASQ2 in chicken skeletal muscles was noted in a few early protein assays of CASQ [24], but we establish here that CASQ2 expression in skeletal muscles is consistent across all 42 bird species sampled. The finding that birds switched their calsequestrin is surprising because expression specificity of CASQ1 in skeletal muscle and CASQ2 in cardiac muscle has been consistent across all other lineages in the entire ~450 Myr evolutionary history of jawed vertebrates (Fig 2). The high conservation of sequence, clear split in sequence identity between CASQ1 and CASQ2, and lack of variation in expression patterns of these paralogs across ~300 Myr of evolutionary history between the jawed vertebrate and avian ancestors all indicate an ancient subfunctionalization that is not genetically labile. Based on these facts, we conclude that the switch in use of CASQ2 in the skeletal muscle in birds is a biologically significant molecular evolutionary event.

### Calsequestrin-1 is present in many (but not all) bird genomes

**Fig 3:**
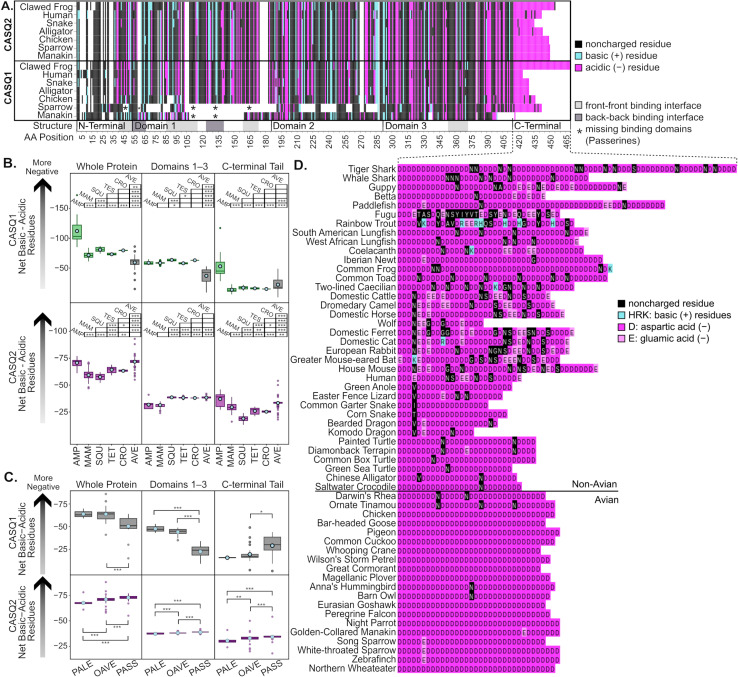
Calsequestrin conservation, amino acid composition, and expression differs across tetrapod groups. A) Amino acid composition and conservation of CASQ2 and CASQ1. The bottom track denotes structurally important binding sites as described in the literature. The tracks above depict acidic residues (cyan) and positive residues (magenta) for 7 species representing major tetrapod clades. Black and white represent non-charged residues and gaps, respectively. B) Relative charge for the entire protein, the structural domains, and the C-terminal for tetrapod (B) and avian (C) CASQ1 (top three graphs) and CASQ2 (bottom three graphs). Dots represent the mean and boxes are the IQR with median center line. In B, each graph contains a diagram to illustrate significant relationships between groups where the group listed on the diagram corresponds to the row of boxes beside it and the columns correspond to the boxplot. C depicts significant relationships between avian clades with brackets (* *P* < 0.05, ** *P* < 0.01, and *** *P* < 0.001). Nonsignificant relationships are not marked. Abbreviations: AMP = amphibians, MAM = mammals, SQU = squamates, TES = Testudines, CRO = crocodilians, PALE = Palaeognathae (ratites), OAVE = other Aves, PASS = passerine birds. D) Amino acid residues of CASQ2 tails from 56 vertebrate species.

The switch to CASQ2 expression in avian skeletal muscles is furthermore surprising because we find that a full-length *CASQ1* gene with no obvious mutational defects is present in the genomes of many bird species and is sometimes even expressed at low levels (Fig 2). We also find *CASQ1* is present in all cartilaginous fish and crocodilian genomes that were previously reported as gene losses. We found these *CASQ1* sequences through intensive surveys of new and updated genome assemblies and *de novo* assembly of transcriptomes. *CASQ1* and *CASQ2* each have the same immediately neighboring genes in all tetrapod genomes surveyed, and highly repetitive content of *CASQ* gene sequences made gene order often more reliable than sequence similarity searches to locate *CASQ1* in many bird genomes. We determined that all birds have a CASQ1 gene present in their genome, except Psittaciformes (parrots) and some Passeriformes (perching birds). Multiple complete Psittaciformes genome assemblies show a complete deletion of *CASQ1* between its two syntenic neighboring genes (S3 Fig). In Passeriformes, *CASQ1* has undergone multiple independent partial or complete deletion events. Both genomic annotation and *de novo* assembly show that exons 2 and 3 are deleted in suboscines, while exons 4 and 5 are deleted in Passeridae (Fig 3A). Other Passerine groups (including zebra finch) appear to have complete deletion of *CASQ1*. Reports of *CASQ1* loss in chicken are likely due to inconsistent assembly at the *CASQ1* locus on microchromosome 25. Vocal learners exhibit high rates of genomic reorganization and nucleotide loss at the *CASQ1* locus, which may have contributed to this gene truncation. Additionally, *CASQ1* is located on microchromosomes in both non-avian reptiles and birds, which potentially affects their dynamics of gene rearrangement and loss [46–49].

The retention of a full-length CASQ1 coding sequence in several birds does leave the possibility that this gene might be functioning in some other context. We quantified expression in chicken lungs, intestines, brains, kidneys, and testes and *CASQ1* was either lowly expressed or exhibited much lower expression than *CASQ2* in all cases (Tables E and F in S1 Data). While it is possible that *CASQ1* has neofunctionalized or maintains a minor undiscovered function in avian tissues, the dramatic drop in skeletal muscle *CASQ1* expression across all birds sampled still supports a synfunctionalization by *CASQ2* with regards to its primary skeletal jSR role. More comprehensive RNA sampling will be necessary to discover any additional CASQ1 functions in birds.

### Avian calsequestrin-2 has enhancements to calcium binding

Calsequestrin is one of the most negatively charged vertebrate proteins with negatively charged amino acids distributed throughout the protein that facilitate the binding of Ca^2+^ ions [9,10,27,50]. CASQ has three structural domains that arrange triangularly and are flanked by unstructured N- and C-terminal tails [9,15]. As the concentration of calcium rises, both calsequestrin proteins stack into dimers, oligomers, helical polymers, and finally a lattice of polymer fibers [12,19]. The N-terminal domain carries a slightly positive charge, and the C-terminal tail is mostly or completely aspartic acid and has a highly negative charge. The negative charge of the molecule as a whole—particularly the C-terminal tail—creates a highly electronegative surface for Ca^2+^ ions to bind [10,11,28].

We analyzed 299 CASQ1 and 507 CASQ2 tetrapod sequences and determined that avian CASQ2 has greater overall net negativity than most other amniotes (Fig 3). While birds and other reptiles do not significantly differ in negativity of the primary domains, birds are significantly more negative in their C-terminal (*P* < 0.001). Thus, increased negativity in avian CASQ2 compared to other amniotes is driven by increased acidic residues in the C-terminal tail. To better understand where this shift occurred, we also compared amino acid sequences within birds (Fig 3C). The C-terminal tail of all non-ratite birds is nearly always completely aspartic acid residues (Fig 3D). Within birds, ratite CASQ2 has a significantly less negative C-terminal compared to non-Passerines (*P* < 0.001) and Passerines have a significantly more negative net charge compared to all other non-ratite birds (*P* < 0.001). Both amphibian CASQ1 and CASQ2 exhibit greater negativity on average than all other tetrapod groups. Amphibian calsequestrins often have longer tails, and this often increases the total number of negative amino acids within the protein. Because this trend is observed in both CASQ paralogs, future research is needed to understand the functional role of calsequestrin in clades diverging before amniotes.

We also tested an alignment of 69 representative vertebrate *CASQ2* coding sequences with aBSREL and found evidence of significant *d*_N_/*d*_S_ increases on the ancestral branch of Sauropsida (LRT, *P* < 0.05 with Holm-Bonferroni correction) [51]. This evidence indicates a possible selective event on the molecule as a whole in a Sauropsida ancestor, followed by an increase in the net number of negatively charged amino acids in the C-terminal tail in birds.

The C-terminal is the major calcium-binding site ion calsequestrin. While high and low-affinity calcium-binding sites can be found across the entire protein, the total negativity of the unstructured C-terminal correlates more closely with the protein’s calcium-binding capacity than the negativity of the protein as a whole [10]. As an example, the mutation of only three charged aspartic acids in the C-terminal tail to neutral alanine was sufficient to reduce CASQ calcium binding by 45% [11]. High calcium concentrations in chicken and quail directly support the increased Ca^2+^ binding of avian CASQ [52,53]. We conclude that CASQ2 likely increased calcium binding capacity dramatically in an avian ancestor, and that additional cumulative boosts to calcium binding appear to have occurred sequentially in ancestors of Neognathae and Passeriformes.

In contrast, avian CASQ1 has a significantly less negative charge compared to all other tetrapod groups, both overall and in the C-terminal tail (all *P* < 0.05; Fig 3B). Oscine and Suboscine passerine CASQ1 sequences have separate deletions that remove large portions of the first and second domains, including the entirety of the intermolecular binding region needed for polymerization (Fig 3A). Based on existing structural models, we conclude that there are no passerine CASQ1 proteins with the structural potential to be capable of any substantial calcium binding or formation of multimers. Additionally, the generally lower net negativity of avian CASQ1 further reinforces the expression data that indicates a severely reduced, if not completely nonfunctional, role in myocyte calcium binding.

### Avian CASQ2 synfunctionalization indicates a shift in cardiac function

Based on the expression, sequence, and structural data we propose a model of avian calsequestrin with an unusual series of gene duplication and loss events. First, in a jawed vertebrate ancestor, the ancestral calsequestrin duplicated and subfunctionalized. Later, in an avian ancestor, changes to the coding sequence or regulation (possibly both) of CASQ2 caused it to functionally replace the activity of CASQ1 in the jSR, a process previously defined as synfunctionalization [33]. Once CASQ2 became synfunctionalized, the displaced CASQ1 decreased in expression in an ancestor of all birds and was subsequently lost, truncated, or pseudogenized in Psittaciformes and Passeriformes lineage. In this synfunctionalization model, we propose that positive selection on CASQ2 C-terminal tail and calcium-binding capacity in cardiomyocytes drove CASQ2 to also become functionally superior to CASQ1 in skeletal muscles. Once rendered effectively redundant in its putative ancestral role, CASQ1 was apparently downregulated and eventually decayed (pseudogenization and loss) in many lineages.

Why would CASQ2 have been under positive selection in avian ancestors? CASQ2 is the predominant calsequestrin observed in chordate cardiomyocytes. Mammals exhibit improved cardiac function compared to ectothermic species due to changes in cardiomyocyte structure [54–56]. These changes include the development of thicker, shorter cardiomyocytes, often with t-tubules that allow for calcium-induced-calcium release to occur simultaneously across the cell [57]. Compared to mammals, avian hearts exhibit further improved performance, as evident by greater cardiac output and stroke volume [56,57]. Birds lack t-tubules and have elongated myocytes more typical of ectothermic vertebrates [57]. Investigations on the cellular structure and calcium-cycling processes of cardiomyocytes in birds have demonstrated that increased cardiac performance is instead linked to changes in SR calcium content and cycling [53,57].

The conclusion from these lines of evidence seems to be that, on a cellular level, avian ancestors developed higher-performing cardiomyocytes in a completely separate way from mammals. Because birds have maintained roughly the same cardiomyocyte structure as reptiles, this performance may have resulted from changes in SR calcium cycling at the molecular level. Studies investigating the relationship between CASQ1 content, calcium leakage, and free calcium content have demonstrated that decreased calcium-binding results in increased calcium leakage via SERCA pumps [20]. Increased CASQ1 in mammalian fast-twitch muscles reduces leakage and prevents additional ATP waste because of improved calcium-binding [20]. As avian cardiomyocytes exhibit increased SR calcium content compared to mammals, improved calcium-binding in the SR likely further contributes to decreased calcium leakage needed to sustain frequent, rapid contractions [57]. Thus, alteration of CASQ2’s Ca^2+^ binding capacity and polymerization capabilities is potentially a major factor contributing to the improved cardiomyocyte performance and subsequent greater cardiovascular function in birds.

While our data clearly supports that CASQ2 has adopted CASQ1’s role in avian skeletal muscles, the primary driver of this shift is less clear, and we can only comment on possibilities. A possible triggering event could have been selection for increased cardiac performance resulting in better calcium-binding capabilities in CASQ2 that could have shifted the dynamics of SR Ca^2+^ flux and led to a reduction of CASQ1 expression in skeletal myocytes. Alternatively, CASQ2 could have been under selection for skeletal muscle performance, potentially related to one of the many physiological differences between modern birds and crocodilians with endothermy and flight as possible candidates. Nonshivering thermogenesis in birds is partially achieved by uncoupling SERCA activity and skeletal muscle contraction [58]. The result of this decoupling is decreased efficiency of SERCA pumps leading to an increase in the amount of heat produced in skeletal muscle tissues. Evidence from mammals has demonstrated both that CASQ mutations can result in hyperthermia-like myopathies and that CASQ expression influences SERCA expression [59]. If positive selection for cardiac performance also resulted in changes to calcium-cycling processes in skeletal muscle tissues, positive selection for CASQ2 in skeletal muscle tissues may be related to the development of avian endothermy, improved cardiovascular and skeletal muscle calcium cycling as a component of flight, or both. Further empirical work to characterize the function of calsequestrins in active avian skeletal muscles will be necessary to test these hypotheses.

### Conclusions

A more complete understanding of genome-trait connections over the long evolutionary histories of animals requires knowledge of both the changing mutational nature of the genomic sequences and their changing context of expression. Our model of calsequestrin synfunctionalization offers new perspectives on the post-duplication relationships of genes and the layered processes of adaptation, pseudogenization, and loss that affect gene families. Defining the intricacies of calcium-cycling in avian muscle tissue requires further empirical investigation, but the evidence we present clearly indicates a substantial event in the molecular evolution of birds. The altered cardiac and skeletal junctional sarcoplasmic reticulum on a molecular level has potentially broad implications for the evolution of key avian cardiovascular, musculoskeletal, and biomechanical traits. Given the many large and small eukaryotic gene families, we suspect many other cases of synfunctionalization are likely to be discovered and hope this work provokes additional investigations of the deep-time evolutionary expression dynamics of animal gene families.

## Materials and methods

### Calsequestrin sequence set construction

We queried NCBI’s GenBank ortholog database for chordate protein sequences labeled as either CASQ, CASQ1, or CASQ2. While this produced many sequences, we feared it left out homologous genes from species with little or poor genome annotation. To overcome this limitation, we used NCBI’s Basic Local Alignment Search Tool (BLAST) [60] protein-protein search option (blastp). BLAST locates homologous sequences by comparing an initial query sequence against NCBI’s GenBank databases. Because these searches may be sensitive to given query parameters, we frequently changed the starting sequence, algorithm, database, and returnable taxa to prevent relying on too broad of a search query. We also used nucleotide sequences from calsequestrin-encoding DNA regions to find CASQ-like DNA sequences using tblastn and blastx. This provided us with additional species that were not found with protein-protein searches alone. We used these same methods to search for homologous sequences in the University of California Santa Cruz’s (UCSC) Genome database.

Because BLAST compares sequences and not gene or protein descriptions to locate homologs, it can return hypothetical or unknown proteins. To confirm that these proteins were truly homologous and not similar (yet unrelated) genes, we viewed the sequences within the genome to identify retained syntenic features. *CASQ1* does not appear in the GRCg7b (GCF_016699485.2) version of the chicken genome, but does appear in the previous GRCq7w_WZ version (GCF_016700215.2). We located a homologous nucleotide sequence for CASQ in the inshore hagfish (*Eptatretus burgeri*) using tblastn and annotated a full length CASQ coding sequence with homologous intron/exon boundaries using the great white shark (*Chacharodon carcharias*) CASQ2 sequence.

### Alignment, quality control, and phylogenetic inference

We aligned our sequences with MAFFT’s L-INS-i progressive method [61] and made small manual edits to sequences when needed (Tables B, G, and H in S1 Data, and S3 and S4 Data files). Some sequences possessed highly unique regions that shared little to no homology with other sequences in the alignment. If a unique motif appeared to be erroneous from an annotation error, we either removed the erroneous portion or removed the entire sequence from the alignment. Calsequestrin possesses a long tail of repeating acidic residues, though the length and exact composition of the tail varies greatly between species. We suspected that the highly variable tails would introduce a large amount of noise in our phylogenetic reconstruction, and so we truncated our sequences by removing the acidic tail.

We inferred our phylogenies using RAxML-ng with WAG [62,63] as our model of protein evolution and used bootstrapping to generate branch supports. We generated bootstrap values using the transfer bootstrap expectation [64]. Our final phylogeny had multiple near-zero branches due to the large number of species included in the alignment with highly similar or identical sequences. iTol’s online tree editor (v6) [65] was used to create the phylogeny in Fig 1C.

### Transcriptome assembly

Sequences were downloaded from the NCBI Sequence Read Archive (https://www.ncbi.nlm.nih.gov/sra) using fastq-dump and assembled using Trinity (v.2.15.1) [66]. If single-end or multiple FASTQ files were available for an individual, the command line was altered to incorporate these files or the correct single-end options, but all other options remained the same. See Tabel B in S1 Data for details on individual assemblies and S2 Data for assembled and newly annotated sequences.

### Identification of calsequestrin genes

Calsequestrin genes were identified in *de novo* and reference transcriptomes by using BLAST to compare transcript sequences against nucleotide sequences (tBLASTn) using known, closely related CASQ1 and CASQ2 protein sequences as the queries. The high conservation of sequence in calsequestrin, its distinctness from other genes, and clear sequence identity between CASQ1 and CASQ2 made identification of these genes generally unambiguous. To exhaustively look for CASQ genes in assembled transcriptomes, we searched down to tBLASTn *E*-value cutoff *E* < 10, but all successful matches had an of *E* < 10^−20^. *De novo* sequences are provided in S2 Data.

### Multidimensional scaling plot

The MDS was calculated from a pairwise distance matrix using simple amino acid distances (all differences = 1; S5 Data). Pairwise distances were calculated using a Python3 script. The MDS was calculated based on Euclidean distances with 10000 iterations using the *mds* function from the *scikit-sklearn* Python3 module and plotted using matplotlib.

### Transcript quantification

Transcript expression levels were quantified using kallisto (v. 0.46.1; https://github.com/pachterlab/kallisto) [67] for species with a reference transcriptome (Table I in S1 Data) and species with de novo Trinity assembly transcriptomes (v.2.15.1). We conducted multiple tests on the effect of additional quality trimming schema and different quantification methods. Quantification with salmon [68], RSEM [69], and STAR [70], and the use of CutAdapt [71] found expression rates reported comparable to kallisto with no additional trimming beyond fastq-dump adapter removal. Based on these tests, we were satisfied that the relative expression numbers reported here are robust to methodology. Expression values are available in Tables C and D in S1 Data. Counts per million (cpm) expression values shown in the main text were calculated as the estimated counts from kallisto divided by the total number of mapped reads by kallisto times 10^6^.

### Calculation of electronegative charge and statistical methods

We quantified an overall net negativity of CASQ proteins, their domains, and the c-terminal by counting the number of positively and negatively charged residues (S5 Data). Aspartic and glutamic acids were counted as a single negative charge (-1) while arginine, histidine, and lysine were each counted as a single positive charge (+1). All other residues were given a value of 0. We then tested for significant differences between groups of tetrapods and separately between birds grouped roughly by their evolutionary relatedness (Paleognathe, Psittaciformes + Passerines, and all other avian species). We calculated *P*-values using pairwise *t*-tests with Bonferroni corrections. Amino acid sequence in Fig 3C was created with Jalview (V2) [72]. To test for selection in CASQ2, we downloaded nucleotide sequences for 69 representative vertebrate sequences and used aBSREL [51] via Datamonkey’s web interface (S7 and S8 Data files) [73].

## Supporting Information

S1 Fig Breakdown of 1,071 sequences used in phylogenetic construction. Color corresponds to the number of sequences attributed to each taxonomic grouping (PRED = pre-duplication jawless fishes, CHO = chondrichthyes, ACT = actinopterygii, SAR = sarcopterygii, AMP = amphibian, REP = reptile, MAM = mammal, AVE = aves). The exact number of sequences per taxonomic group are provided in Table A in S1 Data. A detailed list of sequences and information on exclusion criteria is available in Table B in S1 Data.
(PDF)

S2 Fig Phylogeny of non-chordate calsequestrins with some select chordate sequences. Multiple sequence alignment used to construct the tree is available in S3 Data.
(PDF)

S3 Fig Calsequestrin synteny and exon structure in vertebrates. *CASQ1* (A) and *CASQ2* (B) exhibit varying degrees of conservation in vertebrates. *CASQ1* is usually proximal to *PEA15*, *IRRE*-L, *DCAF8*, and/or *ATP1A2*. In all vertebrates, *CASQ2* is near *VANG*-L and *NHLH2*-L. Calsequestrin paralogs are depicted in black, and representative species are included above each gene block. Arrow depicts direction of transcription. C) *CASQ1* exon structure in vertebrates. In passerines (highlighted in blue) *CASQ1* displays a breakdown in exon structure with missing or modified exons. The length of each exon (boxes) are proportional to the exon length in human *CASQ1*. Present exons are black, missing exons are white, and exons that are present but not homologous are depicted in gray.
(PDF)

S1 Data Table A: Composition of 1,071 species included in phylogenetic reconstruction by paralog and taxonomic group. Table B: CASQ phylogeny sequences. Included are sequences used to construct a phylogenetic tree of CASQ1, CASQ2, and pre-duplication CASQ. Sequences that were pruned (with justification provided) or modified (with a description of the modification) are also included. Table C: CASQ expression date. RNA expression data in total counts, counts per million, and transcripts per million for CASQ1/CASQ2/CASQ. Source material is also provided. Table D: Key for columns in Table C. Table E: Expression of CASQ paralogs in muscle and non-muscle tissues of chicken. Table F: Expression of CASQ paralogs in non-muscle tissues of birds. Table G: Table of chordate protein and nucleotide sequences (when available) obtained through search efforts. De novo assembly sequences are not included. Table H: Table of non-chordate protein and nucleotide sequences obtained through search efforts. Table I: NCBI Reference Transcriptomes used for kallisto mapping
(XLSX)

S2 Data *De novo* assemblies. PDF containing all de novo assembled sequences used for expression analysis and phylogenetic reconstruction.
(PDF)

S3 Data Invertebrate multiple sequence alignment . FASTA file of invertebrate CASQ sequences used to construct the phylogenetic tree in S2 Fig.
(FASTA)

S4 Data Vertebrate multiple sequence alignment. FASTA file of vertebrate CASQ sequences used to construct the phylogenetic tree in Fig 1C.
(FASTA)

S5 Data Annotated multiple sequence alignment (FASTA file) used to estimate electronegativity .
(FASTA)

S6 Data Vertebrate multiple sequence alignment (FASTA file) of CASQ sequences used to construct the MDS in Fig 1D.
(FASTA)

S7 Data Nucleotide alignment (FASTA file) of sequences used to run aBSREL. (FASTA)

S8 Data Output results from aBSREL. (JSON)
