# Supplementary figures and images for: Deep-time gene expression shift reveals an ancient change in avian muscle phenotypes
Source: PLoS Genet. 2025 Apr 11;21(4):e1011663. doi: 10.1371/journal.pgen.1011663 (PMC12037077; doi:10.1371/journal.pgen.1011663)

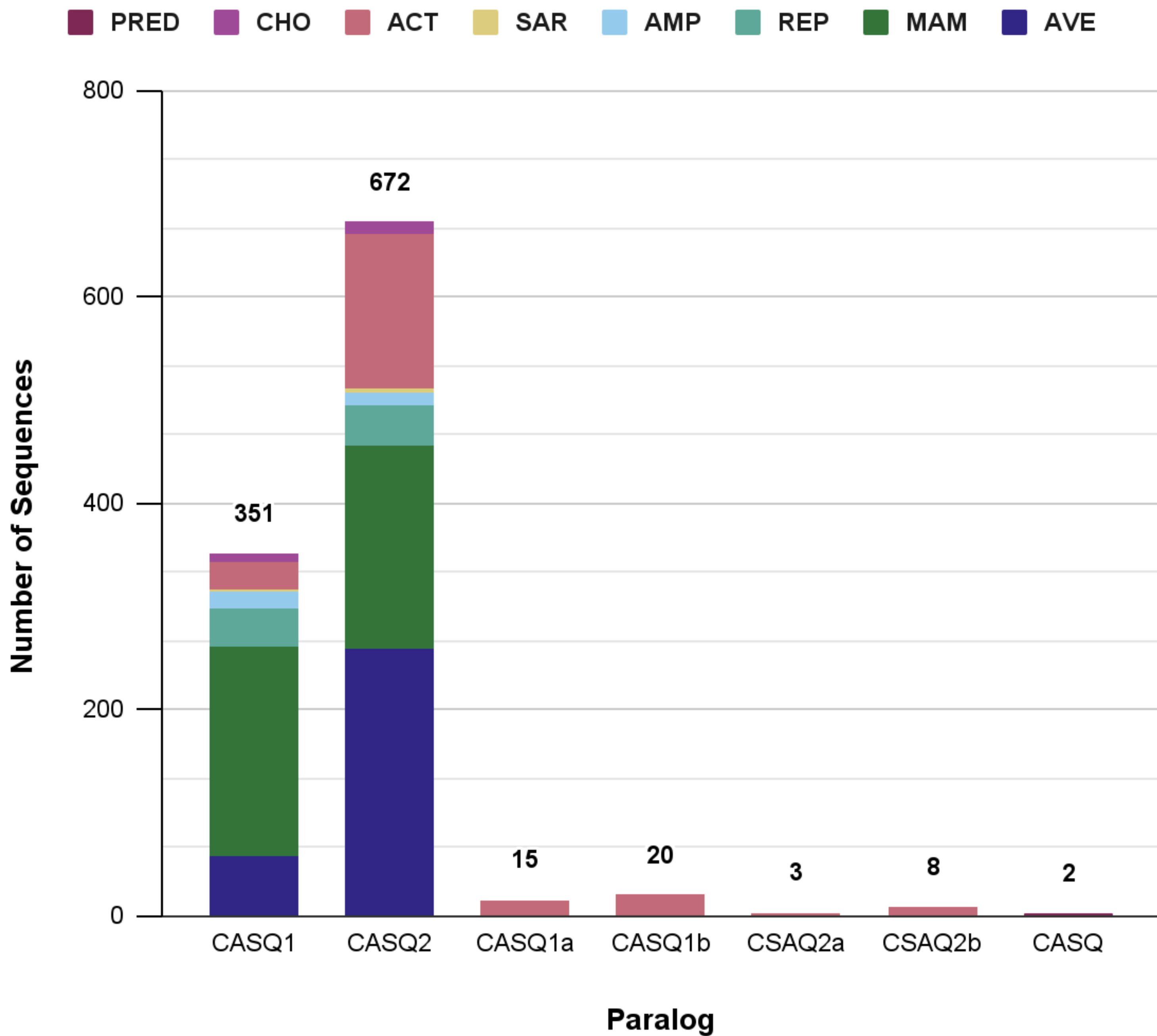

Supplement: S1 Fig — Color corresponds to the number of sequences attributed to each taxonomic grouping (PRED = pre-duplication jawless fishes, CHO = chondrichthyes, ACT = actinopterygii, SAR = sarcopterygii, AMP = amphibian, REP = reptile, MAM = mammal, AVE = aves). The exact number of sequences per taxonomic group are provided in Table A in S1 Data. A detailed list of sequences and information on exclusion criteria is available in Table B in S1 Data. (PDF) [file pgen.1011663.s001.pdf]

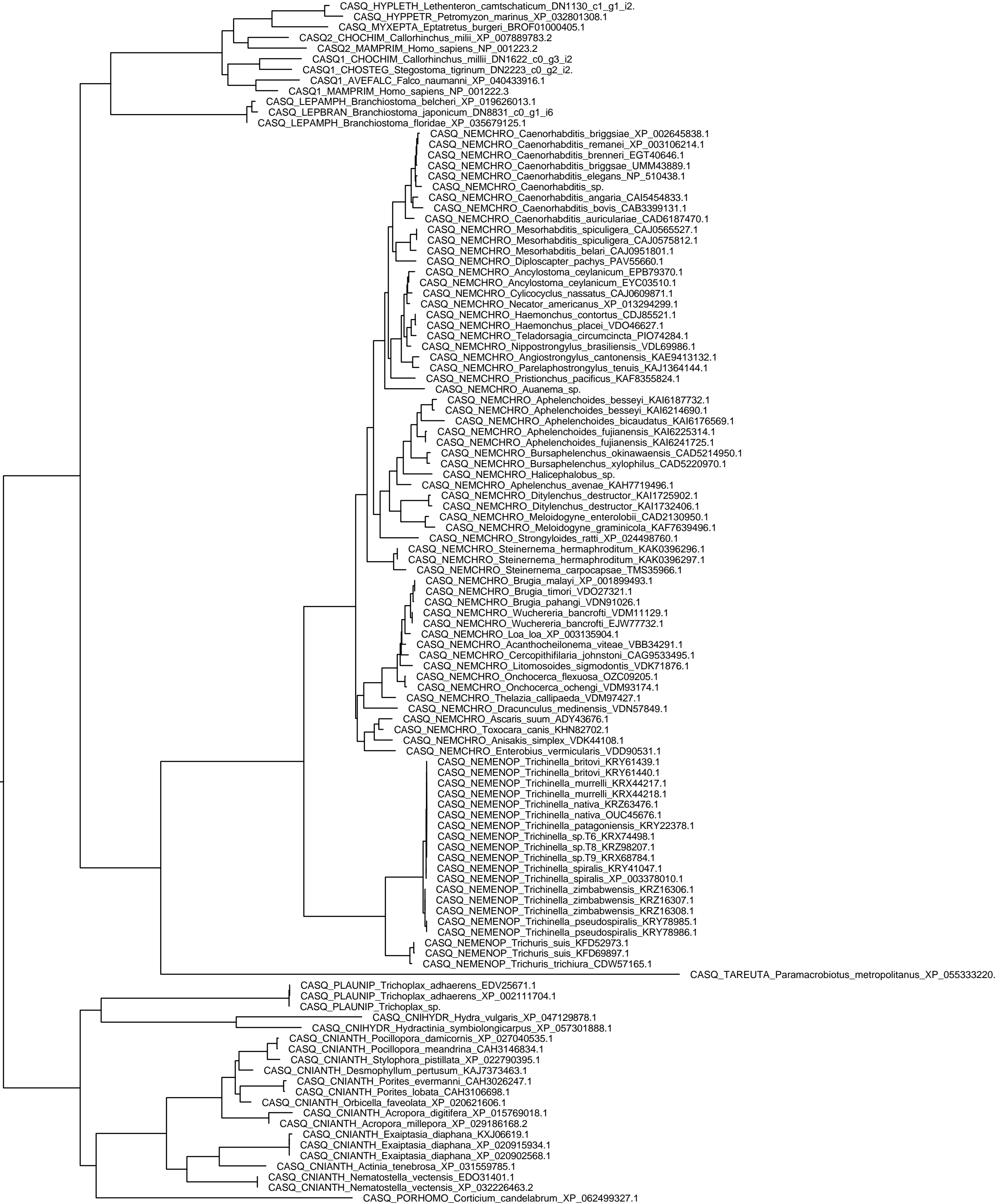

Supplement: S2 Fig — Multiple sequence alignment used to construct the tree is available in S3 Data. (PDF) [file pgen.1011663.s002.pdf]

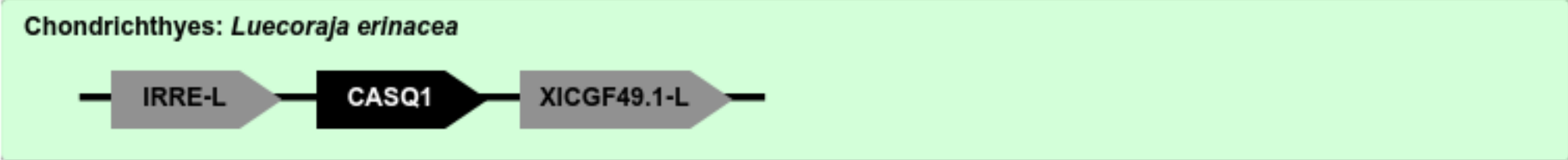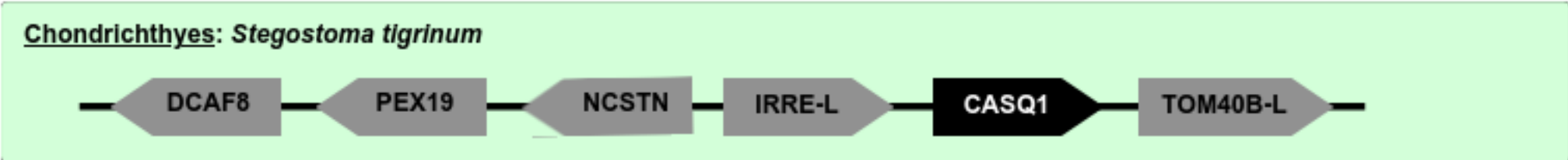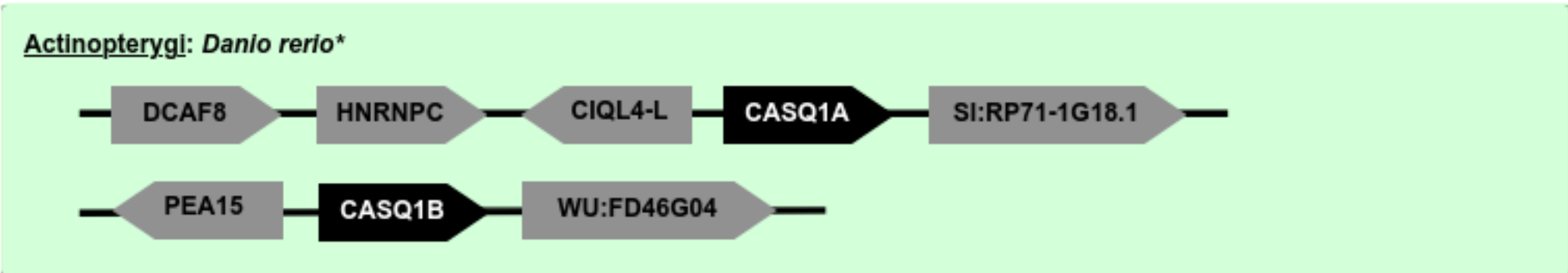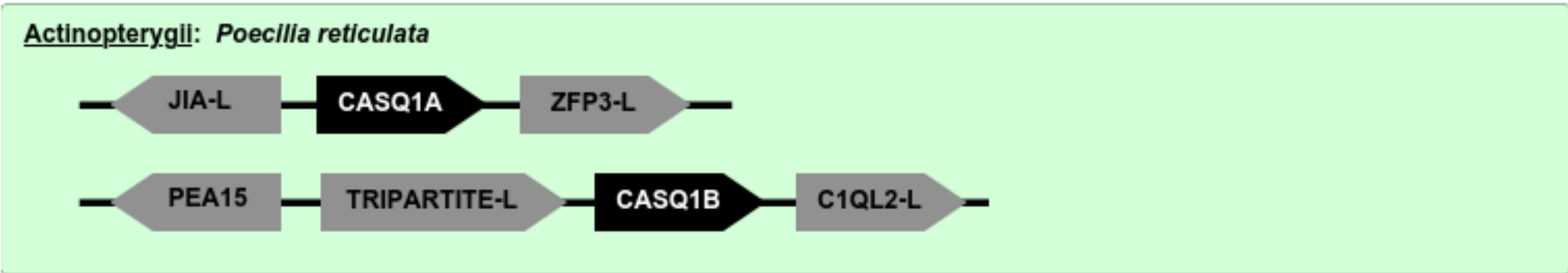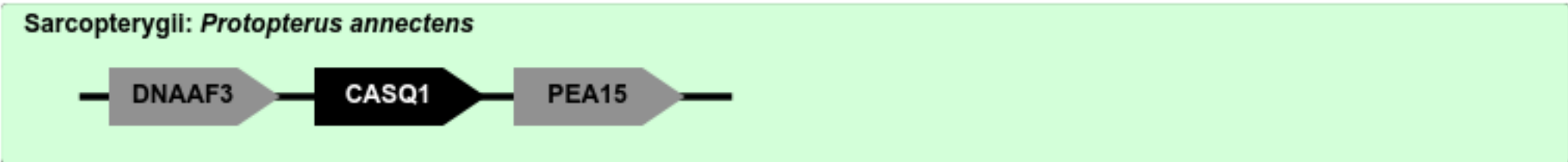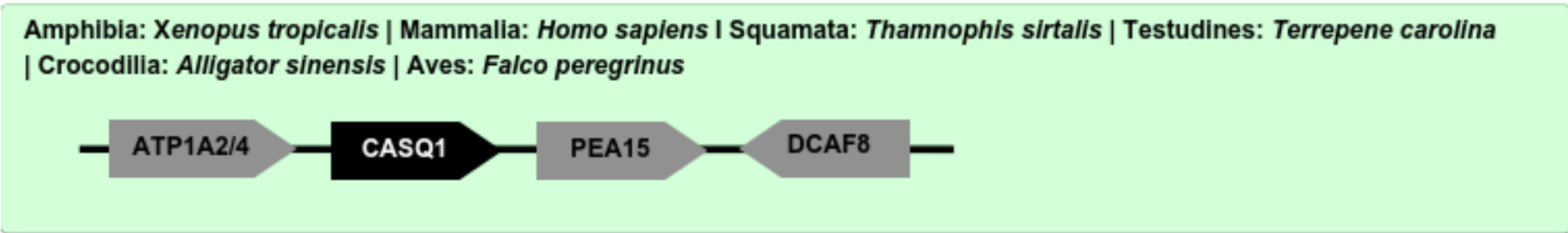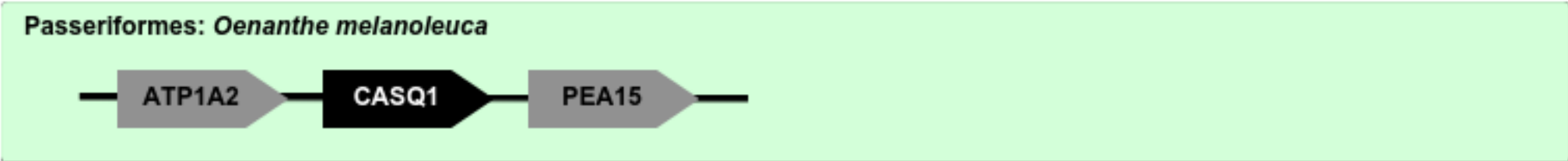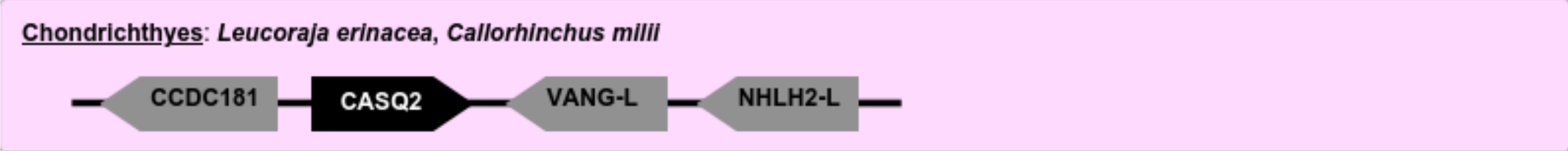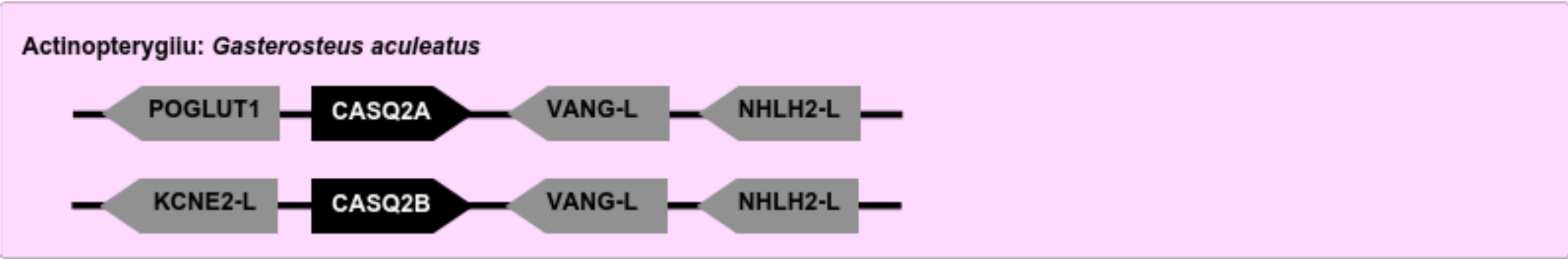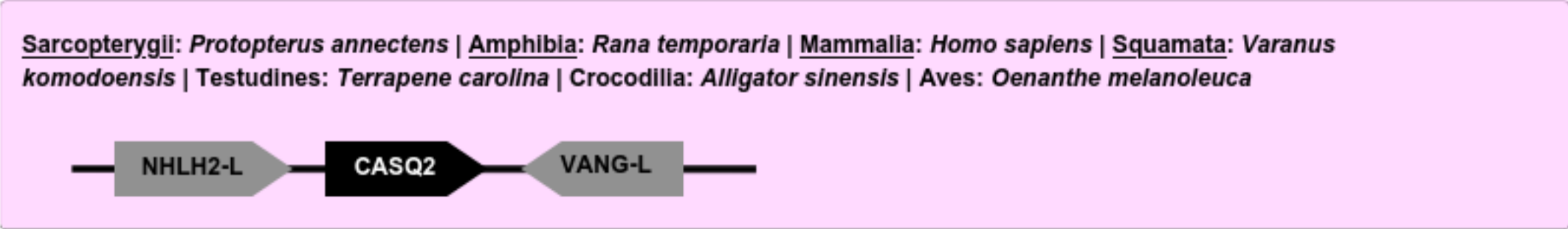

Supplement: S3 Fig — CASQ1 (A) and CASQ2 (B) exhibit varying degrees of conservation in vertebrates. CASQ1 is usually proximal to PEA15, IRRE-L, DCAF8, and/or ATP1A2. In all vertebrates, CASQ2 is near VANG-L and NHLH2-L. Calsequestrin paralogs are depicted in black, and representative species are included above each gene block. Arrow depicts direction of transcription. C) CASQ1 exon structure in vertebrates. In passerines (highlighted in blue) CASQ1 displays a breakdown in exon structure with missing or modified exons. The length of each exon (boxes) are proportional to the exon length in human CASQ1. Present exons are black, missing exons are white, and exons that are present but not homologous are depicted in gray. (PDF) [file pgen.1011663.s003.pdf]
