# Supplementary material for: Deep-time gene expression shift reveals an ancient change in avian muscle phenotypes
Source: PLoS Genet. 2025 Apr 11;21(4):e1011663. doi: 10.1371/journal.pgen.1011663 (PMC12037077; doi:10.1371/journal.pgen.1011663)
Supplement: S2 Data — PDF containing all de novo assembled sequences used for expression analysis and phylogenetic reconstruction. (PDF) [file pgen.1011663.s005.pdf]

Calsequestrin *de novo* assembled and annotated sequences

Christina Harvey, James Pease

Contents

1 Cephalochordata **3**
1.1 *Branchiostoma japonicum* (japanese lancelet) . . . 3

2 Cyclostomata **3**
2.1 *Eptatretus burgeri* (hagfish) . . . 3
2.2 *Lethenteron camtschaticum* (japanese lamprey) . . . 4

3 Chondrichthyes **5**
3.1 *Callorhincus millii* (Australian ghostshark) . . . 5
3.2 *Stegostoma tigrinum* (zebra shark) . . . 6

4 Amphibia **8**
4.1 *Ambystoma mexicanum* (axolotl) . . . 8
4.2 *Andrias davidianus* (giant Chinese salamander) . . . 8

5 Dipnoi **10**
5.1 *Lepidosiren paradoxa* (South American lungfish) . . . 10

6 Aves: Non-Psittaciformes/Passeriformes **12**
6.1 *Anser indicus* (bar-headed goose) . . . 12
6.2 *Aptenodytes patagonicus* (king penguin) . . . 13
6.3 *Apus apus* (red-crested pochard) . . . 14
6.4 *Aythya ferina* (common pochard) . . . 17
6.5 *Buteo hemilasius* (upland buzzard) . . . 18
6.6 *Calypte anna* (Anna's hummingbird) . . . 20
6.7 *Cathartes melambrotus* (greater yellow-headed vulture) . . . 22
6.8 *Chroicocephalus ridibundus* (black-headed gull) . . . 24
6.9 *Coturnix japonica* (Japanese quail) . . . 25
6.10 *Crossoptilon crossoptilon* (white-eared pheasant) . . . 27
6.11 *Milvago chimachima* (yellow-headed caracara) . . . 29
6.12 *Netta rufina* (red-crested pochard) . . . 31
6.13 *Oceanites oceanicus* (Wilson's storm petrel) . . . 33
6.14 *Otis tarda* (great bustard) . . . 35
6.15 *Pygoscelis papua* (gentoo penguin) . . . 36
6.16 *Rhea pennata* (Darwin's rhea) . . . 37

7 Aves: Psittaciformes **39**
7.1 *Lathamus discolor* (Swift parrot) . . . 39
7.2 *Myiopsitta monachus* (monk parakeet) . . . 40

8 Aves: Passeriformes: Acanthisitti and Tyranni **41**
8.1 *Acanthisitta chloris* (rifleman) . . . 41
8.2 *Ceratopipra cornuta* (scarlet-horned manakin) . . . 41
8.3 *Ceratopipra mentalis* (red-capped manakin) . . . 44
8.4 *Lepidothrix coronata* (blue-crowned manakin) . . . 46
8.5 *Manacus vitellinus* (golden-collared manakin) . . . 49
8.6 *Mionectes oleagineus* (ochre-bellied flycatcher) . . . 51
8.7 *Pseudopipra pipra* (white-crowned manakin) . . . 53
8.8 *Xenopipo atronitens* (black manakin) . . . 55

9 Aves: Passeriformes: Passeri **57**
9.1 *Aegithalos concinnus* (black-throated bushtit) . . . 57
9.2 *Aegithalos iouschistos* (black-browed tit) . . . 58
9.3 *Daphoenositta chrysoptera* (varied sittella) . . . 60
9.4 *Lophophanes dichrous* (grey-crested tit) . . . 60
9.5 *Melospiza melodia* (song sparrow) . . . 62
9.6 *Onychostruthus taczanowskii* (white-rumped snowfinch) . . . 64
9.7 *Pardaliparus venustulus* (yellow-bellied tit) . . . 66
9.8 *Passer montanus* (Eurasian tree sparrow) . . . 68
9.9 *Periparus rubidiventris* (rufous-vented tit) . . . 69
9.10 *Poecile palustris* (marsh tit) . . . 71
9.11 *Pseudopodoces humilis* (Tibetan ground-tit) . . . 73
9.12 *Pyrgilauda ruficollis* (rufous-necked snowfinch) . . . 76
9.13 *Taeniopygia castanotis* (Australian zebra finch) . . . 78
9.14 *Zonotrichia albicollis* (white throated sparrow) . . . 79

1 Cephalochordata

1.1 *Branchiostoma japonicum* (japanese lancelet)

From: Trinity *de novo* assembly of SRR12011606. Pre-duplication CASQ from TRINITY_DN8831_c0_g1_i6.

```
>CASQ_Branchiostoma_japonicum_transcript_assembly
CGGTCATATATGGCATAGAGTCATTGGCAAAAATGCGTATTCGACAGTACGACAAGTATGTGCCGTATTTAGCCGTGCTCCATAACCCA
TAGCTGACTGGGGAAGGGAGACCGCTCCTGGAACACGCTCGGCTTGTCTCGACTGTTTCTCTTTGATAACCGGTGGCGGTTTTTATCCA
TACAAGCGAAATGAGGTTGGTTTTGTGGACGGTTTGTTTGCTGCTGGTGTCGGGATGGGGCGCGCTGGCGGACGAACCGGATAACCCTG
AGGACGAGTTCATGTTCCCACCCTGGGACGGAGACAACCGCATCTTGAACATCGGACAGAAGAACTTCAAGAAGGCCGTCAAGGACAGC
GAGGTTCTCATCGTCTTGTTCTCGGGCGACTTCGACGAGCACTCGGAGGGGGACACCCAGGAGGAGGTGTCGGAATACGCCTTACAGGT
CACATGTCAAGTCTTGGAAGACAGGGACGTCGAATGTGGAGAAATCGACTTAGAATCGGACGCAAAATTAGCTAAACAAGAAGGCGTTG
ACGAGCACGGAACGATACTTGTTTACAAGGATGACGAGGTTATCGAGTACCTGGGACACAGAGCGCCAGAAATCCTCATCAGCTTCGTG
GTTGATCTCTTTGAAGATGCTCATGTCGACATCATCAAGGGCAAGGCCGGGAAGGAAGACTTCGACGATGACGATGACGAAGTCAGAGT
TGTCGGCTGGTTCGAGGACAAAAACAGCAAACACTTCAAGGAATTCGAAGACGCTGCTAACCACTTCCCGACTCTCGAGTTCTTTGCGA
CCTTTGACCCTAAGCTTGCCAAGCAGCTGAAGCTGAAGGATCTGAACGATATCCTGATCTACAAGCCGTTCGAGAAGGACCCGGTGGAG
ATCGAGGACGACGACCCGCACGGGCTGGAGGAGCACGAGATCGAGGAGTTCATCGAGGACAACATGGAGCCAATTATTGAGAAATTCAC
GCTGGAAGATCTCCATCGCGTCTGGGACGAGGACGAGGACGACCACATGATCGTGTTCTTCGCCGAGGAGGATGACAAAGAGGGTAAGC
AGTTCCTGAAGCTAATCGCGCAGCTCGCACGCAAGAACGCCGATGAGAACTTGGACTTCATCTGGATCGACCCTGACGAGGTCCCGACG
CTGGAGGACTACTTTGAGGAGGTCTTTGACATCGACCTGGATGATCCTCAGATCGGCGTGGTAGACCTCGATGATAACGACAGCGTGTT
CCTGGACCTGCCTGACGACGGTCTGCCGACCCTGGACGAGCTGCAGGCCTGGGTGGACGACATCCTGGACGGGGACATCGACTATGACG
ATGATGATGACGACGACGACGATGACGATGACGACGACGATGACGACGACGACGACGATGATGAAGACGAAGACGATGACGACGAGGGT
GAGTAGTGAGAACTTGGTGGTGGCAAGAGCAAAACGGCCACTGTCAAGAGACGGAAGCTCCTCGCCATAGATGACGACGACGACGATGA
TGATGATGACGACGACGATGATGATGATGACGATGATGATGACGACGATGATGATGATGATGATGACGACGACGAC
```

```
>CASQ_Branchiostoma_japonicum_CDS
ATGAGGTTGGTTTTGTGGACGGTTTGTTTGCTGCTGGTGTCGGGATGGGGCGCGCTGGCGGACGAACCGGATAACCCTGAGGACGAGTT
CATGTTCCCACCCTGGGACGGAGACAACCGCATCTTGAACATCGGACAGAAGAACTTCAAGAAGGCCGTCAAGGACAGCGAGGTTCTCA
TCGTCTTGTTCTCGGGCGACTTCGACGAGCACTCGGAGGGGGACACCCAGGAGGAGGTGTCGGAATACGCCTTACAGGTCACATGTCAA
GTCTTGGAAGACAGGGACGTCGAATGTGGAGAAATCGACTTAGAATCGGACGCAAAATTAGCTAAACAAGAAGGCGTTGACGAGCACGG
AACGATACTTGTTTACAAGGATGACGAGGTTATCGAGTACCTGGGACACAGAGCGCCAGAAATCCTCATCAGCTTCGTGGTTGATCTCT
TTGAAGATGCTCATGTCGACATCATCAAGGGCAAGGCCGGGAAGGAAGACTTCGACGATGACGATGACGAAGTCAGAGTTGTCGGCTGG
TTCGAGGACAAAAACAGCAAACACTTCAAGGAATTCGAAGACGCTGCTAACCACTTCCCGACTCTCGAGTTCTTTGCGACCTTTGACCC
TAAGCTTGCCAAGCAGCTGAAGCTGAAGGATCTGAACGATATCCTGATCTACAAGCCGTTCGAGAAGGACCCGGTGGAGATCGAGGACG
ACGACCCGCACGGGCTGGAGGAGCACGAGATCGAGGAGTTCATCGAGGACAACATGGAGCCAATTATTGAGAAATTCACGCTGGAAGAT
CTCCATCGCGTCTGGGACGAGGACGAGGACGACCACATGATCGTGTTCTTCGCCGAGGAGGATGACAAAGAGGGTAAGCAGTTCCTGA
AGCTAATCGCGCAGCTCGCACGCAAGAACGCCGATGAGAACTTGGACTTCATCTGGATCGACCCTGACGAGGTCCCGACGCTGGAGGA
CTACTTTGAGGAGGTCTTTGACATCGACCTGGATGATCCTCAGATCGGCGTGGTAGACCTCGATGATAACGACAGCGTGTTCCTGGAC
CTGCCTGACGACGGTCTGCCGACCCTGGACGAGCTGCAGGCCTGGGTGGACGACATCCTGGACGGGGACATCGACTATGACGATGATG
ATGACGACGACGACGATGACGATGACGACGACGATGACGACGACGACGACGATGATGAAGACGAAGACGATGACGACGAGGGTGAGTA
G
```

```
>CASQ_Branchiostoma_japonicum_protein
MRLVLWTVCLLLVSGWGALADEPDNPEDEFMFPPWDGDNRILNIGQKNFKKAVKDSEVLIVLFSGDFDEHSEGDTQEEVSEYALQVTCQ
VLEDRDVECGEIDLESDAKLAKQEGVDEHGTILVYKDDEVIEYLGHRAPEILISFVVDLFEDAHVDIIKGKAGKEDFDDDDDEVRVVGW
FEDKNSKHFKEFEDAANHFPTLEFFATFDPKLAKQLKLKDLNDILIYKPFEKDPVEIEDDDPHGLEEHEIEEFIEDNMEPIIEKFTLED
LHRVWDEDEDDHMIVFFAEEDDKEGKQFLKLIAQLARKNADENLDFIWIDPDEVPTLEDYFEEVFDIDLDDPQIGVVDLDDNDSVFLDL
PDDGLPTLDELQAWVDDILDGDIDYDDDDDDDDDDDDDDDDDDDDDEDEDDDDEGE*
```

2 Cyclostomata

2.1 *Eptatretus burgeri* (hagfish)

```
CASQ Annotation from Scaffold BROF01000405.1
EXON01 BROF01000405.1:569735-569502 (-) (partial, beginning at start codon)
EXON02 BROF01000405.1:567217-567133 (-)
EXON03 BROF01000405.1:552331-552231 (-)
EXON04 BROF01000405.1:551834-551723 (-)
EXON05 BROF01000405.1:550892-550819 (-)
EXON06 BROF01000405.1:528486-528356 (-)
EXON07 BROF01000405.1:524128-524083 (-)
```

EXON08 BROF01000405.1:523992-523938 (-)
EXON09 BROF01000405.1:523755-523655 (-)
EXON10 BROF01000405.1:522609-522535 (-)
EXON11 BROF01000405.1:522202-521954 (-) (partial, ending at start codon)

>CASQ_Eptatretus_burgeri_CDS
ATGGCCCTGTTCTTGCTCATCTCTCTCCTGTCGTTCTCTCTGTCGAGCTCGTCCGGAGAGGCAGGGCTGCGGTTCCCACGCTATGACGG
GAAAGAGCGAGTTTTGGACATCGATGCAGAAAACTGGAAGCGCATCGTCAAGGAGAGCCCCATGCTCTGCTTGCTCTACCACGACTCTC
CGGGCTCTGGGAACACGGCGCAGAAGCAGTTCCACCTGACGGAGCTAACCCTGGAGATGGTCGCCCAGGTTCTGGATGGGCGGGGCATC
ACCTTTGGCCTGGTTGATGCCAAGAAGGACAAGAAAGTTGCCAAGAAGCTTGGTTTGAAGGAGACAGGAAGCATGTACATATTCAAGGA
GGATCACTTGATCGAGTTTGATGGGGAGTTGTCTGCTCACGTTATCACAGAGTTCCTTCTTGATCTGATGGATGACCCGGTGGAGTTCC
TCACGGGAGAGGCAGAGCTTCGAGCAATTTACGCCCTCGATGACGTCAGCAGGGTCATTGGCTACTTCAAGAACGTGGATTCTGAACAC
TACAAGGCTTTTGAAGATGCTGCACGGGACTTCCAGCCCCACATTCCATTCTATGCCACCATTGATAAAGCCACTGCGAAGAAACTTTC
GATGAAGTTGAATGATGTGCAGTTCCATGAACCTTTCACAGATCATCCAGTCAACATGGCTGACAAACCCCACACTGAGGACGAGATAG
TACACTTTGTGAAGGAACATCGCAGGGCGACTCTGCGGAAACTGCGCCCTGAAGACATGTACGAGACGTGGGAGGATGACATTGATGGA
ATACACATCGTTGCATTCGCAGAGGAGGATGATCCAGATGGTTACGAGTTCCTGTCTATCCTAAAGGATGTGGCTCAGGCCAACACTGA
TAATCCTGATCTCAGCATCATTTGGATTGATCCTGATGACTTCCCTCTTATGATCAGCTTCTGGGAAAAAACTTTTGGCATTGACCTCT
TCCGGCCACAGATCGGCGTCGTCAATGTCACAGATGCGGACAGCGTGTGGCTCGCGATGGATGACGAGGAGGACCTGCCAAGTCCAGCG
GAGTTGGAGAAATGGATCAAAGACGTTCTGTCTGGGGAGATCAACACCGAGGATGACGACGTCGAAGGAGATGATGATACAGATGATGA
TGATGACGATGAAGATGATGACACAGATGATGACACGGATGACACCGATGATGACACCGATGATGACACCGATGATGATACAGATGATG
ACGATGATGACGATTAA

>CASQ_Eptatretus_burgeri_protein
MALFLLISLLSFSLSSSSGEAGLRFPRYDGKERVLDIDAENWKRIVKESPMLCLLYHDSPGSGNTAQKQFHLTELTLEMVAQVLDGRGI
TFGLVDAKKDKKVAKKLGLKETGSMYIFKEDHLIEFDGELSAHVITEFLLDLMDDPVEFLTGEAELRAIYALDDVSRVIGYFKNVDSEH
YKAFEDAARDFQPHIPFYATIDKATAKKLSMKLNDVQFHEPFTDHPVNMADKPHTEDEIVHFVKEHRRATLRKLRPEDMYETWEDDIDG
IHIVAFAEEDDPDGYEFLSILKDVAQANTDNPDLSIIWIDPDDFPLMISFWEKTFGIDLFRPQIGVVNVTDADSVWLAMDDEEDLPSPA
ELEKWIKDVLSGEINTEDDDVEGDDDTDDDDDDEDDDTDDDTDDTDDDTDDDTDDDTDDDDDDD*

2.2 *Lethenteron camtschaticum* (japanese lamprey)

From: Trinity *de novo* assembly of SRR5051324. Pre-duplication CASQ from complement of TRINITY_DN1130_c1_g1_i2.

>CASQ_Lethenteron_camtschaticum_transcript_assembly
ACAACAACCACAACAACAACAACCACAACCATCTCCTCCCCCTACTCCTCCTTCTCGTATCTGTCCTCCAAATCACCGCCGCGTCGTCC
TCTTCTGGCCTCGGCTTCCCTCGCTACGATGGAGTCGATCGAGTGCTCGACCTTTCTGCTCGCAACTACAAAGCTGCCTTGAGTTCCCA
CGTGGCCATCGGAGTCCTCCTGCATCGACCCCTGCCGGCTGGAGATGCTATTGCAGCTAAACAGCACGCCATGACGGAGTTGGTGCTGG
AGATGGCTGCCCAGGTGCTGGAGGCGAGGGGCGTCGCGTTCGCTCTGCTCGACGCCGAAGACGACAAGAAGGTGGCTCGCAAACTCGGC
GTGACCGAGGTGGATAGCATGTTCATCTTCAAGGACGACCACGTGGTCGAGTTTGATGGAGAGTTCTCTGCTGACGTCATAGTGGAGTT
CATCCTGGACCTGCTGGAGGACCCGGTGGAGATGCTGACTGGAGATGCTGAGCTGAGGGCGTTCCACTCGGTGGACGAGATTCCCAAAG
TGGCCGGGTTCTTCAAGAATGATCAATCAGAGCACTACAAGGCCTATGAAGACGCTGCAAAAGATTTCCAACCGTACATCCCCTTCTAC
GCGACCTTCGATAAAGCGACTGCCAAGAAATTGTCTCTAAAGCTGAACGAGGTGGACTTCTATGAGCCATTCACGTCCAAAGCTGTTTC
CATTCCGGACAAACCCCACACAGAAGAAGAGTTGGTGGCGTTCATCAAGGCTCACCGCAGGGCAACGTTGAGGAAACTTCGCCCAGAAG
ATATGTACGAGACATGGGAGGATGACATAAACGGCATCCACATCGTCGCCTTTGCTGAGGAAGATGACCCAGATGGTTTCGAGTTTCTC
GAAGTCCTGAAGCAAGTGGCTGAAGACAACACAGACAACGCCGGACTCAGCATCATTTGGATCGACCCCGACGACTTTCCCCTGCTCAT
CTCGTTTTGGGAGAAAACGTTCAAGATTGACCTCTACAGACCGCAGATCGGAGTGGTGAACGTCACGGATGCGGACAGCATCTGGATGA
AGATGGAGGACGAGGAAGACCTTCCCAGCGCACAGGAGCTCCAGGACTGGGTGGAGGACGTTTTGTCGGGAGAGGTCAACACGGAAGAT
GATGATGACGATGATGATGACGACGATGATGATGACGATGATGATGA

Note: Transcript assembly is also a partial CDS.

>CASQ_Lethenteron_camtschaticum_protein_partial
XNNHNNNNHNHLLPLLLLLVSVLQITAASSSSGLGFPRYDGVDRVLDLSARNYKAALSSHVAIGVLLHRPLPAGDAIAAKQHAMTELVL
EMAAQVLEARGVAFALLDAEDDKKVARKLGVTEVDSMFIFKDDHVVEFDGEFSADVIVEFILDLLEDPVEMLTGDAELRAFHSVDEIPK
VAGFFKNDQSEHYKAYEDAAKDFQPYIPFYATFDKATAKKLSLKLNEVDFYEPFTSKAVSIPDKPHTEEELVAFIKAHRRATLRKLRPE
DMYETWEDDINGIHIVAFAEEDDPDGFEFLEVLKQVAEDNTDNAGLSIIWIDPDDFPLLISFWEKTFKIDLYRPQIGVVNVTDADSIWM
KMEDEEDLPSAQELQDWVEDVLSGEVNTEDDDDDDDDDDDDDDD

3 Chondrichthyes

3.1 *Callorhincus millii* (Australian ghostshark)

From: Trinity *de novo* assembly of SRR514107. CASQ1 from TRINITY_DN1622_c0_g3_i2. CASQ2 from compelement of TRINITY_DN2967_c1_g1_i1.

```
>CASQ1_Callorhincus_millii_transcript_assembly
CTCTCGTTCTCTCGTTCTCTCGCCGCCGCCGCCCACCGCCGCCCCCTAACCCCCGCGGACGACAGGGATGATTCCGTCTGCGGCTGCCC
TGCTTCTCCTCCTCGCGGGCTTGGGGCTCGCGGAGGAAGGGATGGACATCCCTCGCTACGACGGGGCGGACCGAGTCCTCCACTTGTCC
CTCAAGAACTACAAGCAGGCGGTCAAGAAGTTCGACATCTTGTGCCTGTACTACCACGAGGCTGTGGAAGACGATGCTGTGTCCTACAA
GGAGTTTGAGTGGGAAGAGCTGACCCTCGAGCTCGCGGCCCAGGTCTTGGAAGATGAAGGAATTGGTTTTGGTTTGGTCGATGCTGAGG
ATGATGATGCTGTTGCCAAAAAACTAGGTCTATCCGAAGTTGGGAGTATATATGTTTTCAAGGAAGATAATGTAATCGAATATGATGGA
CAATTATCATCAGATACAATCGTAGAATTTCTTCTGGACCTTTTGGAAGATGCCGTAGAGATTATTGAAGATGAAACAGAAGTGGATTC
GGTCGAAGATGATGAAGATGAACCCAAATTAATCGGATATTTCAAGAATAAGGAGTCCGAACAATTCCTGACGTACACGGAGGCGGCCG
AGCATTTTCAACCTTATATCACCTTTTACGCCACTTTCGATAAAAAGGTCGCCAAACATTTCGACCTTAAATTGAACGAAGTTGAGTTT
TACGAAGCGTTTACAGACGAAGGAATCACAATCCCAGGAAAACCTTACACTGAGACGGAACTGGTGGAGTTTGTGAACGAACATAAAAG
AAGCACTTTGAGAAAACTGAAACCCGACAGCATGTACGAGACTTGGGAAGATGATATCAACGGCATTCATATCGTTGCCTTCGCTGAAG
AAGATGATGCCGACGGTAACGACTTTCTGGAGCTGTTGAAAGAAGTCGCGGAAGACAACACAGATAATCCCAATTTGAGCATAATTTGG
ATCGACCCTGAAGACTTCCCCTTGTTGAATTTATATTGGGAGAAAACATTTGGAATCGATCTGTCAGAACCTCAGATTGGAGTCGTCAA
TGTCACAGACGCTGACAGTGTGTGGCTGGACCTCGATGATGACGACTTGCCGAGCGCTGACGAGTTGGAGGATTGGATCGAAGACGTTC
TGTCCGGAGACATCAGCCCCGATGACGACGACGACGACGATGATGATGATGACGACGACGACGATGATGACGATGATGATGACTGAGCC
CCTCACCTTCCCTGAGGACAACTG
```

```
>CASQ1_Callorhincus_millii_CDS
ATGATTCCGTCTGCGGCTGCCCTGCTTCTCCTCCTCGCGGGCTTGGGGCTCGCGGAGGAAGGGATGGACATCCCTCGCTACGACGGGGC
GGACCGAGTCCTCCACTTGTCCCTCAAGAACTACAAGCAGGCGGTCAAGAAGTTCGACATCTTGTGCCTGTACTACCACGAGGCTGTGG
AAGACGATGCTGTGTCCTACAAGGAGTTTGAGTGGGAAGAGCTGACCCTCGAGCTCGCGGCCCAGGTCTTGGAAGATGAAGGAATTGGT
TTTGGTTTGGTCGATGCTGAGGATGATGATGCTGTTGCCAAAAAACTAGGTCTATCCGAAGTTGGGAGTATATATGTTTTCAAGGAAGA
TAATGTAATCGAATATGATGGACAATTATCATCAGATACAATCGTAGAATTTCTTCTGGACCTTTTGGAAGATGCCGTAGAGATTATTG
AAGATGAAACAGAAGTGGATTCGGTCGAAGATGATGAAGATGAACCCAAATTAATCGGATATTTCAAGAATAAGGAGTCCGAACAATTC
CTGACGTACACGGAGGCGGCCGAGCATTTTCAACCTTATATCACCTTTTACGCCACTTTCGATAAAAAGGTCGCCAAACATTTCGACCT
TAAATTGAACGAAGTTGAGTTTTACGAAGCGTTTACAGACGAAGGAATCACAATCCCAGGAAAACCTTACACTGAGACGGAACTGGTGG
AGTTTGTGAACGAACATAAAAGAAGCACTTTGAGAAAACTGAAACCCGACAGCATGTACGAGACTTGGGAAGATGATATCAACGGCATT
CATATCGTTGCCTTCGCTGAAGAAGATGATGCCGACGGTAACGACTTTCTGGAGCTGTTGAAAGAAGTCGCGGAAGACAACACAGATAA
TCCCAATTTGAGCATAATTTGGATCGACCCTGAAGACTTCCCCTTGTTGAATTTATATTGGGAGAAAACATTTGGAATCGATCTGTCAG
AACCTCAGATTGGAGTCGTCAATGTCACAGACGCTGACAGTGTGTGGCTGGACCTCGATGATGACGACTTGCCGAGCGCTGACGAGTTG
GAGGATTGGATCGAAGACGTTCTGTCCGGAGACATCAGCCCCGATGACGACGACGACGACGATGATGATGATGACGACGACGACGATGA
TGACGATGATGATGACTGA
```

```
>CASQ1_Callorhincus_millii_protein
MIPSAAALLLLLAGLGLAEEGMDIPRYDGADRVLHLSLKNYKQAVKKFDILCLYYHEAVEDDAVSYKEFEWEELTLELAAQVLEDEGIG
FGLVDAEDDDAVAKKLGLSEVGSIYVFKEDNVIEYDGQLSSDTIVEFLLDLLEDAVEIIEDETEVDSVEDDEDEPKLIGYFKNKESEQF
LTYTEAAEHFQPYITFYATFDKKVAKHFDLKLNEVEFYEAFTDEGITIPGKPYTETELVEFVNEHKRSTLRKLKPDSMYETWEDDINGI
HIVAFAEEDDADGNDFLELLKEVAEDNTDNPNLSIIWIDPEDFPLLNLYWEKTFGIDLSEPQIGVVNVTDADSVWLDLDDDDLPSADEL
EDWIEDVLSGDISPDDDDDDDDDDDDDDDDDDDDD*
```

```
>CASQ2_Callorhincus_millii_transcript_assembly
GTTCTGACTCTGCTTTCTGTCGCTGACTAGCAGCAGTGATCAGTGCTGAAAGAGAAGCGCAAGAGTACTTGTCTGATCTCTCTACTGGC
TCTTCTGTCATCTTTTAAGAAAAGAACAATAATAATCTAACTCTTGCCATGAGTGTTGGCTGGCTCTATTTGGTGACCTTTTGTCTCAC
TTGCACCTGCTGTCGTGCAGAGAAAGGATTAGAAATCCCAAGGTACGATGGGAAGGACAGAGTTATCGATATCGACATGAAAAACTACC
AGAAGGTTATGAAAAAGTTCGATGTATTATGCCTCTTGTATCATGAGGCCATCCCTGCTGGAGACAAAGTTGCTCAGAAACGGTTTGAC
CTGGTGGAACTCGTATTAGAGATGGCAGCTCAAGTCTTGCAGGAAAAAGACGTTGGATTTGGTCTCGTAGATGAGAAGAAAGATGCAAA
ATTAGCCAAGAAATTGGGTCTGATAGAAACTGGGAGTTTGTACATCTTTAAGGAGGATAATCTTATTGAGTACGATGGAGAGCTCTCTG
CTGATGTGCTGGTGGAATTTCTTCTGGACCTGGCAGAAGAACCAGTGGAGATCATTGAAGTGGACGTAGAGATTCAGAGTTTTCTTAGG
ATTGAAGATGAGATCAAACTGATCGGATATTTTAAAAGCGAAGATTCTGAACATTACAAAGCATTTGAGGGAGCAGCTGAGCACTTTCA
CCCTTACATTCATTTTTTTGCTACGTTTGATAAAGGGGTCGCGAAGAAGCTTTCCATGAAGATGAATGAGGTGGATTTTTATGAACCTT
TCATGGATGAACCGATCACCATTCCTGACAAGCCACACTCAGAGGAAGAAATCGTAGAGTTCATTCAGGAGCAAAAGAGGTCAACTCTC
AGAAAACTGCGTCCAGAGAACATGTTTGAGACGTGGGAAGATGATATGGATGGGATCCACATAGTGGCTTTTGCTGAAGAAGAAGATCC
CGATGGTTATGAATTTTTAGCGATTTTAAAAGAAGTAGCAAAAGATAACACCGACAATCCAGAACTCAGCATTATATGGATCGACCCCG
ATGACTTCCCCCTGTTGTTTACATACTGGGAAAAAGTGTTCAAGATTGACCTTCACAAACCTCAGATTGGAGTCGTGAATGTCACTGAT
GCTGACAGTGTCTGGATGGATATTAAAGATGAAGATGAACTGCCATCGGTTGATGAGCTGGAAGATTGGATCCATGACGTGCTCTCAGG
```

AAAGGTTAATACAGAAGATGATGACGACGGTGATGATGACGATGATGATGACGACGATGATGATGAAGATGACGATGAT

>CASQ2_Callorhincus_millii_CDS_partial
ATGAGTGTTGGCTGGCTCTATTTGGTGACCTTTTGTCTCACTTGCACCTGCTGTCGTGCAGAGAAAGGATTAGAAATCCCAAGGTACGA
TGGGAAGGACAGAGTTATCGATATCGACATGAAAAACTACCAGAAGGTTATGAAAAAGTTCGATGTATTATGCCTCTTGTATCATGAGG
CCATCCCTGCTGGAGACAAAGTTGCTCAGAAACGGTTTGACCTGGTGGAACTCGTATTAGAGATGGCAGCTCAAGTCTTGCAGGAAAAA
GACGTTGGATTTGGTCTCGTAGATGAGAAGAAAGATGCAAAATTAGCCAAGAAATTGGGTCTGATAGAAACTGGGAGTTTGTACATCTT
TAAGGAGGATAATCTTATTGAGTACGATGGAGAGCTCTCTGCTGATGTGCTGGTGGAATTTCTTCTGGACCTGGCAGAAGAACCAGTGG
AGATCATTGAAGTGGACGTAGAGATTCAGAGTTTTCTTAGGATTGAAGATGAGATCAAACTGATCGGATATTTTAAAAGCGAAGATTCT
GAACATTACAAAGCATTTGAGGGAGCAGCTGAGCACTTTCACCCTTACATTCATTTTTTTGCTACGTTTGATAAAGGGGTCGCGAAGAA
GCTTTCCATGAAGATGAATGAGGTGGATTTTTATGAACCTTTCATGGATGAACCGATCACCATTCCTGACAAGCCACACTCAGAGGAAG
AAATCGTAGAGTTCATTCAGGAGCAAAAGAGGTCAACTCTCAGAAAACTGCGTCCAGAGAACATGTTTGAGACGTGGGAAGATGATATG
GATGGGATCCACATAGTGGCTTTTGCTGAAGAAGAAGATCCCGATGGTTATGAATTTTTAGCGATTTTAAAAGAAGTAGCAAAAGATAA
CACCGACAATCCAGAACTCAGCATTATATGGATCGACCCCGATGACTTCCCCCTGTTGTTTACATACTGGGAAAAAGTGTTCAAGATTG
ACCTTCACAAACCTCAGATTGGAGTCGTGAATGTCACTGATGCTGACAGTGTCTGGATGGATATTAAAGATGAAGATGAACTGCCATCG
GTTGATGAGCTGGAAGATTGGATCCATGACGTGCTCTCAGGAAAGGTTAATACAGAAGATGATGACGACGGTGATGATGACGATGATGA
TGACGACGATGATGATGAAGATGACGATGAT

>CASQ2_Callorhincus_millii_protein_partial
MSVGWLYLVTFCLTCTCCRAEKGLEIPRYDGKDRVIDIDMKNYQKVMKKFDVLCLLYHEAIPAGDKVAQKRFDLVELVLEMAAQVLQEK
DVGFGLVDEKKDAKLAKKLGLIETGSLYIFKEDNLIEYDGELSADVLVEFLLDLAEEPVEIIEVDVEIQSFLRIEDEIKLIGYFKSEDS
EHYKAFEGAAEHFHPYIHFFATFDKGVAKKLSMKMNEVDFYEPFMDEPITIPDKPHSEEEIVEFIQEQKRSTLRKLRPENMFETWEDDM
DGIHIVAFAEEEDPDGYEFLAILKEVAKDNTDNPELSIIWIDPDDFPLLFTYWEKVFKIDLHKPQIGVVNVTDADSVWMDIKDEDELPS
VDELEDWIHDVLSGKVNTEDDDDGDDDDDDDDDDDEDDDD

3.2 *Stegostoma tigrinum* (zebra shark)

From: Trinity *de novo* assembly of SRR19140277. CASQ1 from complement of TRINITY_DN2223_c0_g2_i2. CASQ2 from complement of TRINITY_DN1720_c0_g1_i9.

>CASQ2_Stegostoma_tigrinum_transcript_assembly
CACCCTCCCCCCCCGCCCCACACACACCAGCCCCTTCTCTTTGGGGTGGGGGGGTTGGGAAAGGGGTGGGAGCCAACATGCCAGTGCTG
CCTCCATGCCCCCCCACATCCACCCATTGGTGACCTCTCTGCTCGCCTCACCGCTTTCTAATTATAGGCGGGGAGGACAGCCTGAGTCC
GGTATTCCTTAATATGGGCGTGTAACTCTGCCAGGCACTATTCTGTACCTCGGGCGGACAAGCCGGCGTGGGGGGCAGGACCTGGAACA
TAGCTCTGGCACTTGCCAAAGCTGCAGCTCACAGCCCTTTTTCCTCCGCTAGCCCTCTGACCTTCTCTCTCTCTCCGTCTCTGCCTCTC
TCCCTCTCTTCGGGAAGAAGGAGCTCTGGTGCTGGTAGCCAGCAAACACCAGACCCTGCCCCTTGTGCCGCTGCCTCAGCTCTGCCACG
CGCGCTGCCCGCCGTCAACGCCCTCCCCTTTGGCACTTCGCGGTCTCCTGATCATACCCTCGACAAAGAAAGCCACAAAACCAGATTCC
CCACCCCACCTTCCTTTCTCCAACCCTTGTGTCTCAAGATGAGGTGCATCTGGTTTTTGGTGTTTGCGTTGACCCTGGCATTGTCCTGG
GCAGAGGAGGGCATTGACATTCCCGAGTACGATGGCGTAGACCGGGTCATCCATCTTTCACCCAAGAACTACAAGCAGGTTCTGAAGAA
GTTTGACATCTTGTGCCTGTACCACCATGAACCCATCGAGGACGACAAGGTCTCACAGAAGCAGTTCGAGTGGGAGGAACTCACTCTGG
AGCTTGCAGCACAGGTCCTCGAGGACGAAAATATTGGTTTCGGATTGGTCGATGCAGAGGAGGATGAGGCTGTTGCAAAGAAACTGGGT
TTGACTGAGGAAGGCAGCATCTATGTCTTCAAGGAGGACAATGTGATCGAGTATGATGGAGAGATGGCCGCTGACACCATTGTGGAGTT
CCTCTTAGACCTTCTCGAAGACCCTATAGAGATGATTGATGATGAAGGCGAGATTGAAGTTTTCGAAGATGATGATGATGAGCCCAAGT
TGGTCGGTTACTTCAAGAATGAAGACTCTGAACATTTCAAAGCTTACAAAGAAGCAGCTGAGGTGTTCCAGCCGTATATCGCCTTTTAT
GCGACATTTAACAAGAGAGCAGCTAATGATCTCGATCTCAAATTGAACCAAATCGATTTCTACGAGCCCTTCATGGATGAATCTGACAC
ATTGCCAGGAAAGACACACACAGAGAAAGAAATCATCGATTATGTGAACAAGCATAAAAGAGCCACCCTCCGGAAGCTGAAGCCCGATA
GCATGTATGAAACCTGGGAGGATGATATAAACGGAATACACATTGTGGTGTTTGCAGAGGAAGATGATGCAGATGGTTACGAGTTCCTG
GAGATTGTGAAGGAGGTTGCTGAAGACAACACTGATAACCCAAACCTGAGCATCATCTGGATCGATCCTGAGGATTTCCCGCTGTTGAA
CCCTTACTGGGAGAAGACGTTTGGTATCGATCTCTCTGAGCCCCAGATTGGGGTGGTTAACGTCACAGATGCAGACAGTGTCTGGCTCA
ACATGGATGAAGATGACTTGCCGAGTGCCGATGAGTTGGAGGATTGGATTGAGGATGTTCTTTCTGGTGAGATAAACACTGAAGATGAC
GATGATGATGAAGATGATGACGATGAAGACGATGACGACGATGATGGTGATGATGATGATGACGATGATGATGATGATGACGATGACGA
TGAAGAATAATTTAACACCCACGTCACCAAATCTCAACATGATGCCAACGATCGTCAACCTATTTATTGCCAACAAAAAACTTGGCTCC
GATCTACAGCGTCTCCTTTTTTTTCTATCTGTAAATCTATATAACTTTAACATTTTGTATTGAAAAATCAATATACTATCTTGCTGGAA
TGGGGAAATGAAAATACAGAGAACAAAAGTGAACTGACAGATCGGAAGAGCGTCG

>CASQ2_Stegostoma_tigrinum_CDS
ATGAGGTGCATCTGGTTTTTGGTGTTTGCGTTGACCCTGGCATTGTCCTGGGCAGAGGAGGGCATTGACATTCCCGAGTACGATGGCGT
AGACCGGGTCATCCATCTTTCACCCAAGAACTACAAGCAGGTTCTGAAGAAGTTTGACATCTTGTGCCTGTACCACCATGAACCCATCG
AGGACGACAAGGTCTCACAGAAGCAGTTCGAGTGGGAGGAACTCACTCTGGAGCTTGCAGCACAGGTCCTCGAGGACGAAAATATTGGT
TTCGGATTGGTCGATGCAGAGGAGGATGAGGCTGTTGCAAAGAAACTGGGTTTGACTGAGGAAGGCAGCATCTATGTCTTCAAGGAGGA
CAATGTGATCGAGTATGATGGAGAGATGGCCGCTGACACCATTGTGGAGTTCCTCTTAGACCTTCTCGAAGACCCTATAGAGATGATTG
ATGATGAAGGCGAGATTGAAGTTTTCGAAGATGATGATGATGAGCCCAAGTTGGTCGGTTACTTCAAGAATGAAGACTCTGAACATTTC

AAAGCTTACAAAGAAGCAGCTGAGGTGTTCCAGCCGTATATCGCCTTTTATGCGACATTTAACAAGAGAGCAGCTAATGATCTCGATCT
CAAATTGAACCAAATCGATTTCTACGAGCCCTTCATGGATGAATCTGACACATTGCCAGGAAAGACACACACAGAGAAAGAAATCATCG
ATTATGTGAACAAGCATAAAAGAGCCACCCTCCGGAAGCTGAAGCCCGATAGCATGTATGAAACCTGGGAGGATGATATAAACGGAATA
CACATTGTGGTGTTTGCAGAGGAAGATGATGCAGATGGTTACGAGTTCCTGGAGATTGTGAAGGAGGTTGCTGAAGACAACACTGATAA
CCCAAACCTGAGCATCATCTGGATCGATCCTGAGGATTTCCCGCTGTTGAACCCTTACTGGGAGAAGACGTTTGGTATCGATCTCTCTG
AGCCCCAGATTGGGGTGGTTAACGTCACAGATGCAGACAGTGTCTGGCTCAACATGGATGAAGATGACTTGCCGAGTGCCGATGAGTTG
GAGGATTGGATTGAGGATGTTCTTTCTGGTGAGATAAACACTGAAGATGACGATGATGATGAAGATGATGACGATGAAGACGATGACGA
CGATGATGGTGATGATGATGATGACGATGATGATGATGATGACGATGACGATGAAGAATAA

>CASQ1_Stegostoma_tigrinum_protein
MRCIWFLVFALTLALSWAEEGIDIPEYDGVDRVIHLSPKNYKQVLKKFDILCLYHHEPIEDDKVSQKQFEWEELTLELAAQVLEDENIG
FGLVDAEEDEAVAKKLGLTEEGSIYVFKEDNVIEYDGEMAADTIVEFLLDLLEDPIEMIDDEGEIEVFEDDDDEPKLVGYFKNEDSEHF
KAYKEAAEVFQPYIAFYATFNKRAANDLDLKLNQIDFYEPFMDESDTLPGKTHTEKEIIDYVNKHKRATLRKLKPDSMYETWEDDINGI
HIVVFAEEDDADGYEFLEIVKEVAEDNTDNPNLSIIWIDPEDFPLLNPYWEKTFGIDLSEPQIGVVNVTDADSVWLNMDEDDLPSADEL
EDWIEDVLSGEINTEDDDDDEDDDDEDDDDDDGDDDDDDDDDDDDDDEE*

>CASQ2_Stegostoma_tigrinum_transcript_assembly
CAAGCAGAAGACGGCATACGAGATTACCACCGGTGACTGGAGTTCAGACGTGTGCTCTTCCGATCTGTTTGAAGCATTCTCTCAATCCC
GTAACCTTTCCAAACAGCGTCAACGTGCCAAGATGGAAACGTTATCTGCATTCAGCGTTAGTTGCTTTGAGAGATAGAAGAAAAGCTCT
TCAAAATGACGATGGCGAATCCCCCTTCGACTCGTGCCTTTGCTGTTTCAGTGCAGTGTATTTTTAGCTGGAAACAACTGGCTTCTTAA
AATGGAGTTTGAAGTGGTAAGGGTGCAGGAGGTTGGTATGTGAAGTATCTGGGCTCTGCCTCCATCTATTCTTTCTCCAGGGACTGTTC
TATTTGTTCCCTCTCCCTCTGTCTGTTTCTCTGGTCAGGAGTTGTAACATGTACTACAGGAGGAAGGCATAATCTTTCCAGTGAAGCAA
TTTTACTTCCTCTTTTGTTTTATTGTGTGGTGTTGTGTAAATAATATAACTTTTACAATGAATACAATCTGGCTCTGTTTGGCGACTTT
CTTTGTCGCCTGCATCAGCTGCAGAGCAGAAGATGGACTTGAATTCCCCCGATATGATGGAAAAGACAGAGTTGCCGATGTCGATATGA
AAAACTACCAAAAGATTTTGGAAAAGTACGATATTCTGTGCCTCTTGTATCACGCGTCGGTTCCTGCAGGAGACAAAGTTGCTCAGAAG
CGGTTTGACCGCGTGGAATTGGTTCTCGAGCTGGCAGCTCAGGTCTTACAAGACAAGAACATTGGATTTGGGCTTGTGGATGAGAAGAA
AGATGCAAAGCTGGCTAAGAAACTGGGTTTGATCGAAATTGGGAGTGTGTATATTTTTAAAGATGATCATCTTATTGAATATGATGGCG
AGCTCTCTCCTGAAGTCCTTGTCGCATTCCTTCTGGACGTGATGGAAGATCCAGTGGAAATAATTGAATCTCGGGCAGAAGTTCGGTCT
TTTGAAAGAACTGATGAAGTGATAAAATTGATTGGATACTTTAAAAGTGAAGCTTCTGAACATTACAAAGCTTTTGAAGATGCAGCAGA
GCACTTCCACCCTTATATCAAGTTCTTTGCAACTTTTAACAAAGGGGTCGCAAAGAAACTCTCACTGAAGATGAATGAGGTGGATTTTT
ATGAGCCTTTCATGGATGATTCGGTCACTATTCCTGATAAACCACACTCAGAAGAAGAAATCGTAGAATTTATTCAAGAACATAAAAGA
TCAACCCTCAGGAAACTGCGTCCAGAGGATGTGTTTGAGACCTGGGAGGATGATATGGATGGGATCCAGATTGTTGCTTTTGCTGAAGA
AGAAGACCCTGATGGTTATGAATTTTTGTCAATTTTAAAAGAGGTAGCAAAAGAAAACACTAATAATCCAGATCTAAGCATTGTGTGGA
TTGACCCAGATGACTTCCCACTGCTGTTTACTTACTGGGAAAAAAACTTTCAAGATTGACCTTCACAAGCCTCAGATTGGAATAGTTAAT
GTCACTGATGCTGACAGCGTCTGGATGGATATAAAAGATGATGATGATCTACCATCAACTGATGAACTAGAAGAATGGCTCCATGATGT
TCTCTCAGGAAAAGTCAATACAGAAGACGATGATGATGATGATGATGATGATGACGACGACGACGACGACAATAACGATGATGATGACA
ATGATAATGATGATGATGATGATGATGATGACGACGACGACGACGACAATAACGATGATGATGACAATGATAATGATGATGACAGTGAT
GATGATGATGACGATGATGATAATGATGATGACGATGACAATGATAACGATGATGATGATTAACTGTTCAGTTTAAGATTAATTATCTA
TACTGTGAACAACAAATTTGCGAATTCTACATGTTTAAAGAGATCGGAAGAGCGTCG

>CASQ2_Stegostoma_tigrinum_CDS
ATGAATACAATCTGGCTCTGTTTGGCGACTTTCTTTGTCGCCTGCATCAGCTGCAGAGCAGAAGATGGACTTGAATTCCCCCGATATGA
TGGAAAAGACAGAGTTGCCGATGTCGATATGAAAAACTACCAAAAGATTTTGGAAAAGTACGATATTCTGTGCCTCTTGTATCACGCGT
CGGTTCCTGCAGGAGACAAAGTTGCTCAGAAGCGGTTTGACCGCGTGGAATTGGTTCTCGAGCTGGCAGCTCAGGTCTTACAAGACAAG
AACATTGGATTTGGGCTTGTGGATGAGAAGAAAGATGCAAAGCTGGCTAAGAAACTGGGTTTGATCGAAATTGGGAGTGTGTATATTTT
TAAAGATGATCATCTTATTGAATATGATGGCGAGCTCTCTCCTGAAGTCCTTGTCGCATTCCTTCTGGACGTGATGGAAGATCCAGTGG
AAATAATTGAATCTCGGGCAGAAGTTCGGTCTTTTGAAAGAACTGATGAAGTGATAAAATTGATTGGATACTTTAAAAGTGAAGCTTCT
GAACATTACAAAGCTTTTGAAGATGCAGCAGAGCACTTCCACCCTTATATCAAGTTCTTTGCAACTTTTAACAAAGGGGTCGCAAAGAA
ACTCTCACTGAAGATGAATGAGGTGGATTTTTATGAGCCTTTCATGGATGATTCGGTCACTATTCCTGATAAACCACACTCAGAAGAAG
AAATCGTAGAATTTATTCAAGAACATAAAAGATCAACCCTCAGGAAACTGCGTCCAGAGGATGTGTTTGAGACCTGGGAGGATGATATG
GATGGGATCCAGATTGTTGCTTTTGCTGAAGAAGAAGACCCTGATGGTTATGAATTTTTGTCAATTTTAAAAGAGGTAGCAAAAGAAAA
CACTAATAATCCAGATCTAAGCATTGTGTGGATTGACCCAGATGACTTCCCACTGCTGTTTACTTACTGGGAAAAAAACTTTCAAGATTG
ACCTTCACAAGCCTCAGATTGGAATAGTTAATGTCACTGATGCTGACAGCGTCTGGATGGATATAAAAGATGATGATGATCTACCATCA
ACTGATGAACTAGAAGAATGGCTCCATGATGTTCTCTCAGGAAAAGTCAATACAGAAGACGATGATGATGATGATGATGATGATGACGA
CGACGACGACGACAATAACGATGATGATGACAATGATAATGATGATGATGATGATGATGATGACGACGACGACGACGACAATAACGATG
ATGATGACAATGATAATGATGATGACAGTGATGATGATGATGACGATGATGATAATGATGATGACGATGACAATGATAACGATGATGAT
GATTAA

>CASQ2_Stegostoma_tigrinum_protein
MNTIWLCLATFFVACISCRAEDGLEFPRYDGKDRVADVDMKNYQKILEKYDILCLLYHASVPAGDKVAQKRFDRVELVLELAAQVLQDK
NIGFGLVDEKKDAKLAKKLGLIEIGSVYIFKDDHLIEYDGELSPEVLVAFLLDVMEDPVEIIESRAEVRSFERTDEVIKLIGYFKSEAS
EHYKAFEDAAEHFHPYIKFFATFNKGVAKKLSLKMNEVDFYEPFMDDSVTIPDKPHSEEEIVEFIQEHKRSTLRKLRPEDVFETWEDDM

DGIQIVAFAEEEDPDGYEFLSILKEVAKENTNNPDLSIVWIDPDDFPLLFTYWEKTFKIDLHKPQIGIVNVTDADSVWMDIKDDDDLPS
TDELEEWLHDVLSGKVNTEDDDDDDDDDDDDDDDNNDDDDNDNDDDDDDDDDDDDDNNDDDDNDNDDDSDDDDDDDDNDDDDDNDNDDD
D*

4 Amphibia

4.1 *Ambystoma mexicanum* (axolotl)

CASQ1 Annotation from Scaffold CM010928.2
EXON01 CM010928.2:11820288-11820046 (-) (partial exon, beginning at start codon)
EXON02 CM010928.2:11794507-11794423 (-)
EXON03 CM010928.2:11731573-11731473 (-)
EXON04 CM010928.2:11714313-11714202 (-)
EXON05 CM010928.2:11681756-11681683 (-)
EXON06 CM010928.2:11678030-11677900 (-)
EXON07 CM010928.2:11652278-11652233 (-)
EXON08 CM010928.2:11650941-11650887 (-)
EXON09 CM010928.2:11641265-11641165 (-)
EXON10 CM010928.2:11639504-11639430 (-)
EXON11 CM010928.2:11623894-11623592 (-) (partial exon, ending at stop codon)

>CASQ1_Ambystoma_mexicanum_CDS
ATGAAGGGGCTTTGGCTGGTGGTCACGGCACTATGCCTGGCAGTGGCGAGCCTGGGCGTCCGGGCGAGCGACGGCCTGGACTTCCCCGA
GTATGACGGCGAGGACCGGGTGATCCACCTGAGCAGCAAGAACTACAAGGCGGCCCTGAAGAAGTACGACGTGCTGGCCGTGCTCTACC
ACGAGCCCATCGAGGAGGACAAGGCTTCCAGGCGGCAGTTTGAGATGGAGGAGCTCATCTTTGAGTTAGCAGCCCAAGTCCTGGAGGAT
AAAGGCGTGGGCTTTGGCCTCCTGGACGCTGAGCACGACGCCGCCGTCGCTAAGAAGCTAGATCTGGATGAGGAGGACAGTATTTACGT
GTTCAAAGACGACGAAGTCATTGAGTACGACGGAGAATTTTCTGCTGACACCTTGGTGGAGTTTCTTCTGGATGTGCTTGAGGACCCGG
TGGAATTTATTGAGGGGAACCACGAGCTCACAGCATTTGAGAACATTGAGGATGAGCCAAAACTCATCGGATACTTCAAGAACGAGGAC
TCTGAACATTACAAGGCATTTGAAGACGCCGCAGAAGAATTCCACCCCTTCATCCCATTTTTTGCAACGTTTGACAGCAAGGTTGCCAA
GAAGCTGACCCTGAAGCTGAATGAAATTGACTACTACGAGCCCTTCCATGATGAGCCCATCACCATCCCCAACAAGCCCAACAGTGAGA
AGGAGATTGTCGACTTCCTCGAGCACCACAAGAGACCCACCTTGAGGAAACTGCAGCCTGATAGCATGTATGAGACTTGGGAGGATGAT
ATGGACGGAATCCACATCGTAGCATTTGCAGAAGAAGATGACCCTGATGGTTATGAATTCTTGGAGATCCTTAAAGAAGTTGCCGAAGA
CAACACTGACAACCCCGATCTCAGCATCATTTGGATTGACCCGGAAGACTTCCCACTGCTGATTCCATACTGGGAGGACACCTTTGACA
TTGACCTCTCCCGACCCCACATCGGTGTGGTGAATGTTACAGATGCTGACAGTATTTGGATGGAAATGGATGATGAAGAAGACCTGCCT
TCAGTCGATGAGCTAGAAGACTGGATTGAAGATGTCCTGGATGGAGACATCAACACTGAAGATGACGATGATGACGATGATGATGACGA
TGATGATGACGATGATGACGACGATGATGATGATGATGATGATGATGATGACGACGACGACGATGACGACGACGATGATGACGATG
ATGATGATGACGATGATGATGACGATGATGATGATGATGACGATGACGATGACGATGATGATGATGATGATGATGAGTAA

>CASQ1_Ambystoma_mexicanum_protein
MKGLWLVVTALCLAVASLGVRASDGLDFPEYDGEDRVIHLSSKNYKAALKKYDVLAVLYHEPIEEDKASRRQFEMEELIFELAAQVLED
KGVGFGLLDAEHDAAVAKKLDLDEEDSIYVFKDDEVIEYDGEFSADTLVEFLLDVLEDPVEFIEGNHELTAFENIEDEPKLIGYFKNE
DSEHYKAFEDAAEEFHPFIPFFATFDSKVAKKLTLKLNEIDYYEPFHDEPITIPNKPNSEKEIVDFLEHHKRPTLRKLQPDSMYETWE
DDMDGIHIVAFAEEDDPDGYEFLEILKEVAEDNTDNPDLSIIWIDPEDFPLLIPYWEDTFDIDLSRPHIGVVNVTDADSIWMEMDDEE
DLPSVDELEDWIEDVLDGDINTEDDDDDDDDDDDDDDDDDDDDDDDDDDDDDDDDDDDDDDDDDDDDDDDDDDDDDDDDDDDDDDDE
*

4.2 *Andrias davidianus* (giant Chinese salamander)

From: Trinity *de novo* assembly of SRR4449178. CASQ1 from TRINITY_DN424_c0_g1_i11. CASQ2 from TRINITY_DN574_c0_g1_i1.

>CASQ1_Andrias_davidianus_transcript_assembly
CTCTGGCAAATGTTGGCCGAGTGGCAAGGGGGAAAAAAAAGACGAAAAAGAACGAAAGAAGGGAGCGAGATACAGAGAAAGAAAGAGTG
CACTAACAACATCTGTACTACGTTCACACCTGCCGGGCTAGAGCGTGGAGCCGTATGGCACACCCCAGTTGTGGGATTAATGAGGACAC
GGGTATATCGGGAAGACATACAACACTTCTACATATATAGACAGGGAGCCGAGGGGTGGGCACTGGTGACAGCTTTTCATAGTACCAAG
TTTGCGGAGATAAGACCGCCCCCCATACGTGTTTTCCTCCTCCCCCCAGCCTCATTTATGGGCTGGTTGTCATTTTTTCTCTTTGCTTT
TCAGCCTGTGCACCTTGGACCAGGGACCACCAGCAGCCCCAGACTCTTGCACCCAACAGCCAGCAAGGAGAGGCTGCTCTCAGCCTTCG
ACCATAGTCCCAGTAAAGAAGGGAGTGGATAGCTGGACCCAGTAAGAGAAAGAGAACACATTTCCAAAGCCAGCTCCCTCTCCCCCCTG
GCCACACTTGATCTCAGTTAGGGAGGTTATTTGTTTTCTTACATTGGGGTTTCTAAATTCCCCCGTGCATTGGAACACCCAAGAGGATG
AAGGGGCTTTGGCTGGTGGTCACGGCGCTATGCCTGGCTGTGATAAGCCTGGGCACCCGGGCTGACGACGGACTGGATTTCCCAGAGTA
CGACGGCGAGGACCGAGTCATTCACCTCAGCACCAAGAACTACAAGGCGGCACTGAAGAAGTACGATGTGCTGGCTGTGCTCTACCATG

AGCCCATTGAAGAGGACAAGGCTTCCCAGCGCCAGTTCGAGATGGAGGAGTTGATCTTTGAGTTAGCAGCCCAAGTCCTAGAGGATAAA
GGGGTGGGATTTGGCCTGTTGGACTCCGAGCACGATGCCGCAGTCGCTAAGAAACTAGGGTTGGATGAGGAAGACAGCATCTACGTGTT
TAAAGATGACGAGATCATTGAGTACGATGGGGAATTTTCTGCGGACACCCTGGTGGAGTTTCTTCTAGATGTGCTCGAGGATCCTGTCG
AATTCATCGAGGGAAACCATGAGCTCGCAGCCTTTGAGAACATAGAGGATGATCCAAAACTCATCGGATACTTCAAGAACGAGGAATCT
GAACATTACAAGGCCTTTGAAGACGCTGCTGAAGAATTTCACCCTTTTATCCCCTTTTTTGCAACTTTTGACAGCAAGGTTGCCAAGAA
GCTATCCCTGAAGCTGAACGAGATTGATTACTACGAACCGTTCCATGATGAGCCCGTCACCATTCCCAACAAACCCAACAGTGAAAAGG
AGATTGTAGACTTCCTCGAGGAACACAAGAGGCCCACATTGAGGAAGCTCCAGCCCGACAGCATGTATGAAACTTGGGAAGATGATATG
GATGGAATCCACATTGTAGCATTCGCAGAAGAAGATGACCCCGATGGTTATGAATTCCTGGAGATCCTGAAAGAAGTTGCTGAAGATAA
CACTGACAACCCTGACCTCAGCATCATCTGGATTGACCCAGAGGACTTCCCACTGCTGATTCCATACTGGGAAGAGACCTTCAACATTG
ACCTGTCCCGACCTCACATCGGTGTGGTCAATGTTACAGATGCTGACAGCATATGGCTGGAAATGGAAGATGAAGAGGACCTGCCTTCA
GTCGATGAGCTTGAAGACTGGATTGAAGATGTCTTGGATGGAGATATCAACACAGAAGATGATGATGATGATGAGGACGACGATGATGA
CGACGACGACGACGACGACGATGACGACGACGATGACGATGACGACGACGATGACGATGACGACGACAAAGATGACGATGATGATGATA
ATGACAAAGATGACGATGATGACGACGATGAAGACGATGACAAAGATGATGACAATGATGATGACGATGATGAAGGCAATGACGACGAG
GATGATGACAAAGATGATGACGATGATGATGATGACAAAATTGTAAAGAAACTTGAAGATGATGATGATGACGATGAAGGTAACGATAG
CTAAAATGATGCATACCATTAAAAAGGAATAGGGATCACTTCTACCCAAGGTATTTATTCCTATTTTGAACATTGGTGGAAGAACTAGC
ACTGTTGTTTGTGTTTAAGAAAATGTCAGTCCCTGTGTTACCTTGTGCTCACATTAGTAGGACATCTTTGCTGCTCAGATTAGCTTCCC
CAGCCTTTGACTATTTAACAAACAGTACTCTGCTGGCGGGAACAAGATGCCTTAAAGCATCTCACATCTGTCCACAGTGAGCATTTCCA
GCAACTAGCCTCTAGGCAAAAACGTGCTATCTGATATCGCGTTGTTACCTTCTAGTAAAACAATCTGGACATACAAACTGGAGATTACG
TTGGTATGAACTGGACTGGACTGGATGTCTACTGGAATGAACTGGTTTTCTGTTGGCATGTACTCGGAGACTTTGCAGCTGAAACTTAA
AACGTGTTGTAATCACTGGTGAAAATAAATTGAAGTGGAAAGAATCCCAAGAAGACAACAAGGGGACCAAGGCACAGACACCAACACAG
CAGGGACGGGGTCTTCTGGGTCAACTGTAAACAATCTGGACACTCTCAAAGACTTCTCTGTGTACAAGCCAGTTCACAGACAACCACTT
CACCAGTAGACCAGTTGACCAGATCTATCAAGTGATTGCCAAAGTTGCCCAGTCGCCCCGTCCTGTGCCTAATCCTCGTTCACGGCAGA
AGCTCTTTGCACTTTCTTCACATGCTACCGTGCTTGCTGGTCCCCTGCAAATATCTGAGCCCGCTGGCTCTGCCAACTAGTGTGTCCCT
CCACTGGGGTGATGCCAAGCTCATTCTGCCTGTCAGACCCGGGACTGTTTTTGTGTGTTTCCCTGTACAACACTGAGCCACCCTGACCA
GCACCCTCCCCCCCCCCCCCCCCCCCCCC

>CASQ1_Andrias_davidianus_CDS
ATGAAGGGGCTTTGGCTGGTGGTCACGGCGCTATGCCTGGCTGTGATAAGCCTGGGCACCCGGGCTGACGACGGACTGGATTTCCCAGA
GTACGACGGCGAGGACCGAGTCATTCACCTCAGCACCAAGAACTACAAGGCGGCACTGAAGAAGTACGATGTGCTGGCTGTGCTCTACC
ATGAGCCCATTGAAGAGGACAAGGCTTCCCAGCGCCAGTTCGAGATGGAGGAGTTGATCTTTGAGTTAGCAGCCCAAGTCCTAGAGGAT
AAAGGGGTGGGATTTGGCCTGTTGGACTCCGAGCACGATGCCGCAGTCGCTAAGAAACTAGGGTTGGATGAGGAAGACAGCATCTACGT
GTTTAAAGATGACGAGATCATTGAGTACGATGGGGAATTTTCTGCGGACACCCTGGTGGAGTTTCTTCTAGATGTGCTCGAGGATCCTG
TCGAATTCATCGAGGGAAACCATGAGCTCGCAGCCTTTGAGAACATAGAGGATGATCCAAAACTCATCGGATACTTCAAGAACGAGGAA
TCTGAACATTACAAGGCCTTTGAAGACGCTGCTGAAGAATTTCACCCTTTTATCCCCTTTTTTGCAACTTTTGACAGCAAGGTTGCCAA
GAAGCTATCCCTGAAGCTGAACGAGATTGATTACTACGAACCGTTCCATGATGAGCCCGTCACCATTCCCAACAAACCCAACAGTGAAA
AGGAGATTGTAGACTTCCTCGAGGAACACAAGAGGCCCACATTGAGGAAGCTCCAGCCCGACAGCATGTATGAAACTTGGGAAGATGAT
ATGGATGGAATCCACATTGTAGCATTCGCAGAAGAAGATGACCCCGATGGTTATGAATTCCTGGAGATCCTGAAAGAAGTTGCTGAAGA
TAACACTGACAACCCTGACCTCAGCATCATCTGGATTGACCCAGAGGACTTCCCACTGCTGATTCCATACTGGGAAGAGACCTTCAACA
TTGACCTGTCCCGACCTCACATCGGTGTGGTCAATGTTACAGATGCTGACAGCATATGGCTGGAAATGGAAGATGAAGAGGACCTGCCT
TCAGTCGATGAGCTTGAAGACTGGATTGAAGATGTCTTGGATGGAGATATCAACACAGAAGATGATGATGATGATGAGGACGACGATGA
TGACGACGACGACGACGACGACGATGACGACGACGATGACGATGACGACGACGATGACGATGACGACGACAAAGATGACGATGATGATG
ATAATGACAAAGATGACGATGATGACGACGATGAAGACGATGACAAAGATGATGACAATGATGATGACGATGATGAAGGCAATGACGAC
GAGGATGATGACAAAGATGATGACGATGATGATGATGACAAAATTGTAAAGAAACTTGAAGATGATGATGATGACGATGAAGGTAACGA
TAGCTAA

>CASQ1_Andrias_davidianus_protein
MKGLWLVVTALCLAVISLGTRADDGLDFPEYDGEDRVIHLSTKNYKAALKKYDVLAVLYHEPIEEDKASQRQFEMEELIFELAAQVLED
KGVGFGLLDSEHDAAVAKKLGLDEEDSIYVFKDDEIIEYDGEFSADTLVEFLLDVLEDPVEFIEGNHELAAFENIEDDPKLIGYFKNEE
SEHYKAFEDAAEEFHPFIPFFATFDSKVAKKLSLKLNEIDYYEPFHDEPVTIPNKPNSEKEIVDFLEEHKRPTLRKLQPDSMYETWEDD
MDGIHIVAFAEEDDPDGYEFLEILKEVAEDNTDNPDLSIIWIDPEDFPLLIPYWEETFNIDLSRPHIGVVNVTDADSIWLEMEDEEDLP
SVDELEDWIEDVLDGDINTEDDDDDEDDDDDDDDDDDDDDDDDDDDDDDDDKDDDDDDNDKDDDDDDDEDDDKDDDNDDDDDEGNDD
EDDDKDDDDDDDDKIVKKLEDDDDDDEGNDS*

>CASQ2_Andrias_davidianus_transcript_assembly
CTGCCCTTTCTTTCCATCCGGGTGGTTATTTTTAGACTGAAACAACTGCTACCTAAAAATGGAGCTGCAGCAGCCCCGGAGCCGGCCGG
GCTATGTACGGTATCCCGTGCTTTGGCCTGCAAGCTCCTGTCTATTCTTGTACTCTCTTTGTCTCCAGCTCTGAGGGTCCCAGAGCAAT
CTGCTCCCCTGTCCCAAGTGCTTCTCCAGGAGGGCCGAGAGAGGCAGTAATCAGGCCACGCTCGCTCCCGCTCGCTTCCCCAGCTCCGC
CAGGTAGGACATAGGTGGAGGCGATGAAGACGGTGGGCCTGGTGCTGCTGGCGGGCGTCCTCCTCGCCCTCTGCTATGCCGAGGAGGGT
CTGGACTTCCCCACCTATGATGGGAAGGACCGGGTCCTGGAGCTGAGTGAGCGCAACCTGAAGCAGGCCCTCAAGAAGTACGACATGCT
CTGCATCTTCTACCACCAGCCCGTGGCACCAGACAAGGTCTCCCAGAAGCGCTTCCAGATGACAGAGATGGTGCTGGAGCTTGCTGCTC
AGGTCCTGGAGAATAAAGGCATCGGAGTTGCACTGATAGATTCCAAGAAGGGTGCAAAACTCGCCAAAAAGCTTGGGTGCACAGAAGAA

GGGAGCCTCTACATCTTCAAGGGCGATAACGTGATCGAGTTTGACGGTCAGCTGGCCGCCCATGTCCTGGTCGAGTTCCTCCTGGATCT
GACGGAGCCCCCAGTGGAGGTTATCAACAGCAAACTGGAACTGCAGGCCTTCGAGAACATCGAGGAGAAGATCAAGCTCATCGGCTTCT
TCAAGAATGAGGAGTCTGAGCATTTTAAAGCATTTGAAGAAGCAGCCGAACATTTCCAGCCTTACATTAAATTCTTCGCCACCTTTGAG
AAAGCGGTTGCAAAAAAACTGAGGCTGAAGATGAACGAGGTGGACTACTACGAACCCTTCATGGATGACCCAGTCACTGTCCCAGAGAA
ACCCTACACAGAGGATGAATTGGTGGAGTTTGTGAACGAGCACAAGAGATCCACGCTGCGCAAGTTACGCCCCGAGGACATGTTTGAGA
CTTGGGAGGATGACCTCCAGGGGATCCACATTGTGGCCTTTTCCGAAGAGGACGACCCAGATGGTTTTGAGTTCCTTCAGATCCTGAAG
CAAGTTGCAAAAGACAACACCAACAATCCTGACCTGAGCATAGTGTGGATCGACCCAGATGATTTCCCACTGCTGATCACCTATTGGGA
GAAGACATTTAAGATCGACCTCTTCAAACCCCAGATTGGAGTGGTGAACGTTACAGATGCTGACAGTGTCTGGATGGAAATCAAAGATG
ATGACGACTTACCCACAGCTGAGGAGCTGGAGGACTGGATCAAAGATGTCCTGTCGGGGAAGGTAAACACCGAAGATGATGACGACGAC
CAGGATGATGATGAGGATGATGACGATGATGACGACGATGATGAGGACGACGATGATGAGGACGATGATGATGATGACGATGATGATGA
TGATGACGATGACGACGATGATGATGATGATGATTAGGTCTACATTCATGCAAACCTTTATTCTGGGAAAAGAGGATCTATTGTCCTTT
GTCTCCAACAAGGGAGAGGGGTGCTTAGGGGGAAAGTCACTGTGTGAGCAGAACCATTGTGATGTCTCCTATTAAGACCGAGTCTCTAT
TTTATTTAATAAACAATCCTTCTGCCAAAAAAAAAAAAAAAAAAAAAAA

>CASQ2_Andrias_davidianus_CDS
ATGAAGACGGTGGGCCTGGTGCTGCTGGCGGGCGTCCTCCTCGCCCTCTGCTATGCCGAGGAGGGTCTGGACTTCCCCACCTATGATGG
GAAGGACCGGGTCCTGGAGCTGAGTGAGCGCAACCTGAAGCAGGCCCTCAAGAAGTACGACATGCTCTGCATCTTCTACCACCAGCCCG
TGGCACCAGACAAGGTCTCCCAGAAGCGCTTCCAGATGACAGAGATGGTGCTGGAGCTTGCTGCTCAGGTCCTGGAGAATAAAGGCATC
GGAGTTGCACTGATAGATTCCAAGAAGGGTGCAAAACTCGCCAAAAAGCTTGGGTGCACAGAAGAAGGGAGCCTCTACATCTTCAAGGG
CGATAACGTGATCGAGTTTGACGGTCAGCTGGCCGCCCATGTCCTGGTCGAGTTCCTCCTGGATCTGACGGAGCCCCCAGTGGAGGTTA
TCAACAGCAAACTGGAACTGCAGGCCTTCGAGAACATCGAGGAGAAGATCAAGCTCATCGGCTTCTTCAAGAATGAGGAGTCTGAGCAT
TTTAAAGCATTTGAAGAAGCAGCCGAACATTTCCAGCCTTACATTAAATTCTTCGCCACCTTTGAGAAAGCGGTTGCAAAAAAACTGAG
GCTGAAGATGAACGAGGTGGACTACTACGAACCCTTCATGGATGACCCAGTCACTGTCCCAGAGAAACCCTACACAGAGGATGAATTGG
TGGAGTTTGTGAACGAGCACAAGAGATCCACGCTGCGCAAGTTACGCCCCGAGGACATGTTTGAGACTTGGGAGGATGACCTCCAGGGG
ATCCACATTGTGGCCTTTTCCGAAGAGGACGACCCAGATGGTTTTGAGTTCCTTCAGATCCTGAAGCAAGTTGCAAAAGACAACACCAA
CAATCCTGACCTGAGCATAGTGTGGATCGACCCAGATGATTTCCCACTGCTGATCACCTATTGGGAGAAGACATTTAAGATCGACCTCT
TCAAACCCCAGATTGGAGTGGTGAACGTTACAGATGCTGACAGTGTCTGGATGGAAATCAAAGATGATGACGACTTACCCACAGCTGAG
GAGCTGGAGGACTGGATCAAAGATGTCCTGTCGGGGAAGGTAAACACCGAAGATGATGACGACGACCAGGATGATGATGAGGATGATGA
CGATGATGACGACGATGATGAGGACGACGATGATGAGGACGATGATGATGATGACGATGATGATGATGATGACGATGACGACGATGATG
ATGATGATGATTAG

>CASQ2_Andrias_davidianus_protein
MKTVGLVLLAGVLLALCYAEEGLDFPTYDGKDRVLELSERNLKQALKKYDMLCIFYHQPVAPDKVSQKRFQMTEMVLELAAQVLENKGI
GVALIDSKKGAKLAKKLGCTEEGSLYIFKGDNVIEFDGQLAAHVLVEFLLDLTEPPVEVINSKLELQAFENIEEKIKLIGFFKNEESEH
FKAFEEAAEHFQPYIKFFATFEKAVAKKLRLKMNEVDYYEPFMDDPVTVPEKPYTEDELVEFVNEHKRSTLRKLRPEDMFETWEDDLQG
IHIVAFSEEDDPDGFEFLQILKQVAKDNTNNPDLSIVWIDPDDFPLLITYWEKTFKIDLFKPQIGVVNVTDADSVWMEIKDDDDLPTAE
ELEDWIKDVLSGKVNTEDDDDDQDDDEDDDDDDDDDEDDDDEDDDDDDDDDDDDDDDDDDDDD*

5 Dipnoi

5.1 *Lepidosiren paradoxa* (South American lungfish)

From: Trinity *de novo* assembly of SRR3632086. CASQ1 from the complement of TRINITY_DN12_c0_g1_i13. CASQ2 from the complement of TRINITY_DN791_c0_g1_i1.

>CASQ1_Lepidosiren_paradoxa_transcript_assembly
CTATCCTTTTCCTTCTCATCTCTTCAGCTTTCCTAAATATGGAGATCATAATTCCTTGAAGCCAGCATGGTGGGGTGGGTGGAGGATTC
TTGCACTCACCTCCTCTTCATTCTTAGTGCCAAATACTTAGGACTAAGATAACAGTATCCACAAGTCTAAATGACTTGATGTGCATTCC
TCTCTTGCTCTCCCAGTCCAAGCTCTGTCCGTCTTGGGCTAACAGCCTCTCCACTCTCCAGTGTGTGTTGCAAGAGTAGGACAGCATAT
CTAAGTGATCGAGAGGGAGAGTGGAGAGAAATGAAGTGGTTCTGGTTCATCCTTGCACTGCTGTGTCTGTGCAGTACTGTAGTCCACCC
ACAGGAAGGTTTGGAATACCCAGAATATGATGGCGAGGACCGTGTGATCCATATCAATAGCAAAAATTACAAGCAGGCGCTCAAGAAGT
TTGAAGTTTTGGTGCTGTTCTACCATGAAGCTGTGGAAGACGATCATCATTCCCAACGCCAGTTTGAAGCAGAAGAGCTTGTTCTTGAG
TTAGCTGCTCAAGTCCTCGAGGATGAAAATGTTGGTTTTGGACTGGTTGATGCTGAAGAAGATGCAGCGGTGGCAAAGAAACTAGAGTT
GGACGAAGAGGACAGTTTATACGTTTTTAAAGGTGATGAGGTGATAGAGTATGATGGCGAGTTTTCTGCTGACACCATAGTAGAATTCA
TCCTTGATGTGATGGAGGATCCCGTAGAGGTTATTGAAGGAGACCATGAGATCCGTGCATTTGAAAACATAGATGATGAGCCCAAACTT
ATTGGCTACTTCAAGAATGAGGAGTCTGAACATTTTCAAGCGTATGAAGATGCAGCTGAGGAATTCCACCCTTTCATCAATTTCTTTGC
CACCTTTAACAGTAAGACTGCTAAGAAACTTGACTTGAAGTTGAACGAAATAGATTATTATGAGCCTTTTATGGAAGACCCCGTCACTA
TTCCCAACAAGCCTAACAGTGAAGAGGAGATTGTTGAGTTTCTTGAGGAACATGAGAGGCCAACTCTGAGGAAGCTTCAGCATGACAGC
ATGTATGAGACTTGGGATGATGACTTGGATGGAATCCACATTGTAGCATTTGCAGAGGAAGATGATCCAGATGGTTATGAGTTCCTGGA
AATTCTGAAGGAAGTTGCAAGAGACAATACAGACAACCCTGATCTTAGTATTATCTGGATTGATCCTGAGGAATTCCCCCTGATGATTC
CATACTGGGAGGATACCTTTGATATTGATTTATCTTGCCCTCAAATCGGTATTGTCAATGTAACTGATGCTGACAGTGAATGGATGGAT

ATTGATGATGAGGAAGATCTTCCATCAGCTGATGAGCTGGAGGATTGGCTTGAGGATGTCCTCTCAGGAGAAATTAACACAGAGGATGA
TGATGATGATGAAGATGACGATGACGATGATGATGATGATGATGACGACGATGATGATGATGATGACGATGATGATGATGATGACGATG
ATGATGATGATGACGA

>CASQ1_Lepidosiren_paradoxa_CDS_partial
ATGAAGTGGTTCTGGTTCATCCTTGCACTGCTGTGTCTGTGCAGTACTGTAGTCCACCCACAGGAAGGTTTGGAATACCCAGAATATGA
TGGCGAGGACCGTGTGATCCATATCAATAGCAAAAATTACAAGCAGGCGCTCAAGAAGTTTGAAGTTTTGGTGCTGTTCTACCATGAAG
CTGTGGAAGACGATCATCATTCCCAACGCCAGTTTGAAGCAGAAGAGCTTGTTCTTGAGTTAGCTGCTCAAGTCCTCGAGGATGAAAAT
GTTGGTTTTGGACTGGTTGATGCTGAAGAAGATGCAGCGGTGGCAAAGAAACTAGAGTTGGACGAAGAGGACAGTTTATACGTTTTTAA
AGGTGATGAGGTGATAGAGTATGATGGCGAGTTTTCTGCTGACACCATAGTAGAATTCATCCTTGATGTGATGGAGGATCCCGTAGAGG
TTATTGAAGGAGACCATGAGATCCGTGCATTTGAAAACATAGATGATGAGCCCAAACTTATTGGCTACTTCAAGAATGAGGAGTCTGAA
CATTTTCAAGCGTATGAAGATGCAGCTGAGGAATTCCACCCTTTCATCAATTTCTTTGCCACCTTTAACAGTAAGACTGCTAAGAAACT
TGACTTGAAGTTGAACGAAATAGATTATTATGAGCCTTTTATGGAAGACCCCGTCACTATTCCCAACAAGCCTAACAGTGAAGAGGAGA
TTGTTGAGTTTCTTGAGGAACATGAGAGGCCAACTCTGAGGAAGCTTCAGCATGACAGCATGTATGAGACTTGGGATGATGACTTGGAT
GGAATCCACATTGTAGCATTTGCAGAGGAAGATGATCCAGATGGTTATGAGTTCCTGGAAATTCTGAAGGAAGTTGCAAGAGACAATAC
AGACAACCCTGATCTTAGTATTATCTGGATTGATCCTGAGGAATTCCCCCTGATGATTCCATACTGGGAGGATACCTTTGATATTGATT
TATCTTGCCCTCAAATCGGTATTGTCAATGTAACTGATGCTGACAGTGAATGGATGGATATTGATGATGAGGAAGATCTTCCATCAGCT
GATGAGCTGGAGGATTGGCTTGAGGATGTCCTCTCAGGAGAAATTAACACAGAGGATGATGATGATGATGAAGATGACGATGACGATGA
TGATGATGATGATGACGACGATGATGATGATGATGACGATGATGATGATGATGACGATGATGATGATGATGACGA

>CASQ1_Lepidosiren_paradoxa_protein_partial
MKWFWFILALLCLCSTVVHPQEGLEYPEYDGEDRVIHINSKNYKQALKKFEVLVLFYHEAVEDDHHSQRQFEAEELVLELAAQVLEDEN
VGFGLVDAEEDAAVAKKLELDEEDSLYVFKGDEVIEYDGEFSADTIVEFILDVMEDPVEVIEGDHEIRAFENIDDEPKLIGYFKNEESE
HFQAYEDAAEEFHPFINFFATFNSKTAKKLDLKLNEIDYYEPFMEDPVTIPNKPNSEEEIVEFLEEHERPTLRKLQHDSMYETWDDDLD
GIHIVAFAEEDDPDGYEFLEILKEVARDNTDNPDLSIIWIDPEEFPLMIPYWEDTFDIDLSCPQIGIVNVTDADSEWMDIDDEEDLPSA
DELEDWLEDVLSGEINTEDDDDDEDDDDDDDDDDDDDDDDDDDDDDDDDDDDD

>CASQ2_Lepidosiren_paradoxa_transcript_assembly
TTTTTTTTTTTCTGTCTGTAATTGTCTTCAGCTGGCAAGCTCCAGTCAAGCAGCTTCTGTCTATCTTGGGACATCCATAGCACTTGCTT
AGAGGCATAGCACTGCATTGCTCTCCTGAAACAGGAGCAATCACTTCTGTTGCCTTGTTGGTTTTCCTTCTTCTTACTTCCTCTGCTGC
TCCAGAATGAAGGCACTTTATTTCTTCTTGCTGGCAATCTTTCACGTAATCTGCTTTTGTAGTGCTGAGGAGGGACTCGCCTTCCCCAC
ATACGATGGCGTGGACCGTGTCATTCACATAAATGATAAGAACTACAAACAGGCACTGAAGAAATTTGACATGCTCTGCCTGCTGTACC
ATGAGCCAGTGGCAGCTGATAAGGTATCCCAGAAGCAGTTTCAGATGGTAGAGTTGGTTCTGGAGCTTGCTGCACAAGTCTTAGAAGAC
AAACGCATTGGATTTGGGTTGGTTGATTCTGAGAAAGATGTTAAAGTTGCTAAGAAGCTTGGTCTGATAGAAGAAGGAAGCATCTATGT
CTTTAAAGATGAAAATGTGATAGAATTTGATGGTGAACTTTCTGCTGATATCTTAGTAGAGTTTCTGTTAGATCTGACAGAAGATCCAG
TGGAGATCATCAACAATGTACTGGAGCTTCGGGCCTTTGATATGATTGATGAAGAAACCAAATTGTTTGGCTACTTTAAAGATGAGGAC
TCTGAACATTATAAAGCATTTGAAGAAGCAGCAGAGAATTTCCAGCCATACATCAAATTCTTTGCTACATTTAATAAAGGGATTGCAAA
GAGGCTCAGTCTCAAAATGAATGAGGTAGACTTTTTTGAACCTTTTATGGATGACTCAGTCACAATTCCTGACAAGCCTTATACAGAGG
ATGAGATTGTGGAATTTATCAATGAGCACAAAAGAGCAACCCTAAGGAAATTACGTCCAGAAGACATGTTTGAAACCTGGGAAGACGAC
ATGGATGGGATCCATATTGTAGCATTTGCTGAAGAAGATGACCCTGATGGTTTTGAGTTCCTTGAGATTCTGAAAGAAGTTGCTAAGGA
CAACACCAACAACCCAGACTTAAGTATTCTCTGGATTGACCCTGATGACTTCCCACTGCTGATCACATATTGGGAGAAGACCTTTAATA
TTGATCTTTTCAAGCCTCAGATTGGTGTAGTGAATGTTACTGATGCTGATAGCATATGGATGGAAATTAAAGATGATGATGACCTACCA
AGTGCAAAAGAGCTGGAGGAATGGGTAGAAGATGTGCTATCAGGGAAAATAAACACCGAGGATGATGATGATGACGATGATGATGATGA
TGATGACAATGATGACGATGACAATGATGATGATGATGATGACAATGATGATGATGATGATGATGATGACGATGACAATGATGATGATG
AATAAACTTTCTGCAGTTAACCTCAAGAATTAAATGAATTATTTGTTTTGCCAGGTTAAGATATGCATACTCTTGTGTTTACAAGACAG
ATGTTTTTTACTAAATTAGTGAATGACATACTATCTCTGGATTGCATACATGTTGAAATAGTGCTGCAGTAAAATTAGTTAACATTGAC
CTTTACTGCACAATGCTAACTCACTTATCAGTCCAGAAAGTTAATAATATATTCAGATTTAGTTGGCAATCCAAGAGTAAGAAGAAGAA
ATGTCTTATTTCTAAATTTTAACACGTTATACTGCAAATCTTCAGGGGAACTGGTTCATTCCAGCAAACACCTTTCTTCAAAAACACCA
TTCTTCAGTTATTACATTTTGAATGCTCAGTGGTAAATGCAATTTCTATAGAAATGAAATGCCTCATGTCTTATTTCATTAAGGGCTAA
GAGGGTAATATCATTTTGTTCCAATTAAGTAACTTAAAAATGTAGATTCATTAATTTGTAAATTCTTGACTTATGTGATTTGTATTAAA
AACAAATGTAAAATTTTGTTATTCAGGTCAGTTGCTTCCAAGAAACATTTTCTTGATCAGTATTCT

>CASQ2_Lepidosiren_paradoxa_CDS
ATGAAGGCACTTTATTTCTTCTTGCTGGCAATCTTTCACGTAATCTGCTTTTGTAGTGCTGAGGAGGGACTCGCCTTCCCCACATACGA
TGGCGTGGACCGTGTCATTCACATAAATGATAAGAACTACAAACAGGCACTGAAGAAATTTGACATGCTCTGCCTGCTGTACCATGAGC
CAGTGGCAGCTGATAAGGTATCCCAGAAGCAGTTTCAGATGGTAGAGTTGGTTCTGGAGCTTGCTGCACAAGTCTTAGAAGACAAACGC
ATTGGATTTGGGTTGGTTGATTCTGAGAAAGATGTTAAAGTTGCTAAGAAGCTTGGTCTGATAGAAGAAGGAAGCATCTATGTCTTTAA
AGATGAAAATGTGATAGAATTTGATGGTGAACTTTCTGCTGATATCTTAGTAGAGTTTCTGTTAGATCTGACAGAAGATCCAGTGGAGA
TCATCAACAATGTACTGGAGCTTCGGGCCTTTGATATGATTGATGAAGAAACCAAATTGTTTGGCTACTTTAAAGATGAGGACTCTGAA
CATTATAAAGCATTTGAAGAAGCAGCAGAGAATTTCCAGCCATACATCAAATTCTTTGCTACATTTAATAAAGGGATTGCAAAGAGGCT
CAGTCTCAAAATGAATGAGGTAGACTTTTTTGAACCTTTTATGGATGACTCAGTCACAATTCCTGACAAGCCTTATACAGAGGATGAGA
TTGTGGAATTTATCAATGAGCACAAAAGAGCAACCCTAAGGAAATTACGTCCAGAAGACATGTTTGAAACCTGGGAAGACGACATGGAT

GGGATCCATATTGTAGCATTTGCTGAAGAAGATGACCCTGATGGTTTTGAGTTCCTTGAGATTCTGAAAGAAGTTGCTAAGGACAACAC
CAACAACCCAGACTTAAGTATTCTCTGGATTGACCCTGATGACTTCCCACTGCTGATCACATATTGGGAGAAGACCTTTAATATTGATC
TTTTCAAGCCTCAGATTGGTGTAGTGAATGTTACTGATGCTGATAGCATATGGATGGAAATTAAAGATGATGATGACCTACCAAGTGCA
AAAGAGCTGGAGGAATGGGTAGAAGATGTGCTATCAGGGAAAATAAACACCGAGGATGATGATGATGACGATGATGATGATGATGATG
ACAATGATGACGATGACAATGATGATGATGATGATGACAATGATGATGATGATGATGATGATGACGATGACAATGATGATGATGAATA
A

>CASQ2_Lepidosiren_paradoxa_protein
MKALYFFLLAIFHVICFCSAEEGLAFPTYDGVDRVIHINDKNYKQALKKFDMLCLLYHEPVAADKVSQKQFQMVELVLELAAQVLEDKR
IGFGLVDSEKDVKVAKKLGLIEEGSIYVFKDENVIEFDGELSADILVEFLLDLTEDPVEIINNVLELRAFDMIDEETKLFGYFKDEDSE
HYKAFEEAAENFQPYIKFFATFNKGIAKRLSLKMNEVDFFEPFMDDSVTIPDKPYTEDEIVEFINEHKRATLRKLRPEDMFETWEDDMD
GIHIVAFAEEDDPDGFEFLEILKEVAKDNTNNPDLSILWIDPDDFPLLITYWEKTFNIDLFKPQIGVVNVTDADSIWMEIKDDDDLPSA
KELEEWVEDVLSGKINTEDDDDDDDDDDDDDNDDDDNDDDDDDNDDDDDDDDDDDNDDDE*

6 Aves: Non-Psittaciformes/Passeriformes

6.1 *Anser indicus* (bar-headed goose)

From: Trinity *de novo* assembly of SRR11308171. No CASQ1 was found. CASQ2 from complement of TRINITY_DN964_c5_g1_i2.

>CASQ2_Anser_indicus_transcript_assembly
CCCGCACTCCCTCTCACTCACTCCCTTGCTCTCAGATGATGCAGAGTGCCAGCAAGTCCCTCAATGGTGTCATATCTGTCCTTTTTATT
CATTGCAGGGTTTATTTTTAGCCTGAAACAACTGCTTCCTAAAAATGGAGTTCCTAATGACACGGGAGCCGGACACAGCTATGTAAGGT
ATCCAGGGCTTGGCCTGACAGCTTCTCCTGTCTGTCTTTGTTGTGAGCCCAGGACAGCAAAGTTTGCTGCTTCCCAACCGCCTTGCAGA
GTTTGGACTGAACACTAGGAAGGCAGCAGAAAGAGAAACTCGGTTCAAACTTCCCCTGGAGCCCTCGGCCTCCCTCTCTTTCCAAAGGG
AGAAGGAGAGAGACCTTTGCCTGCCCTCCGCTGCACTCCCAGGGCTCCCCCATGAAGGCAACTTGCTGGATTCTGGCAGGCTTTTGCCT
GCTTTTCTGCTGCAAGGCAGAAGAGGGACTGAATTTCCCCACCTATGATGGGAAAGACCGAGTGATCGACCTGAATGAGAAGAACTACA
AGCAGGCCCTGAAGAAGTATGACATGCTCTGCCTGCTCTTCCACGAGCCCGTGAGCTCCGACAAGGTCTCCCAGAAGCAGTTCCAGATG
ACGGAGATGGTCCTGGAGCTGGCAGCTCAGGTCCTGGAGCCCAGGAGCATCGGGTTTGGGATGGTGGACTCCAAGAAGGATGCCAAACT
TGCCAAAAAGCTAGGCTTGGTTGAAGAGGGAAGTCTCTACGTCTTTAAGGAGGAGCGGTTGATTGAATTTGATGGGGAGCTGGCCACAG
ATGTCTTGGTGGAATTCCTCTTGGATCTGCTAGAAGACCCCGTGGAGATCATAAACAGCAAGCTGGAGCTTCAGGCCTTTGACCAAATT
GATGAAGAAATCAAACTCATTGGCTACTTCAAAGGAGAAGACTCTGAACATTACAAGGCATTTGAGGAAGCTGCTGAACACTTCCAGCC
CTACATCAAATTCTTTGCCACATTTGACAAAGGGGTCGCCAAGAAGCTAGGTCTGAAGATGAACGAGGTGGACTTCTACGAACCGTTCA
TGGATGAGCCTGTTCACATCCCCGATAAGCCTTACACGGAAGAGGAGCTGGTTGAATTCGTGAAGGAGCACAAAAGAGCCACCTTGCGG
AAGTTGCGTCCAGAAGACATGTTTGAGACGTGGGAGGATGACATGGAAGGAATCCATATCGTGGCCTTCGCTGAAGAAGATGACCCAGA
CGGCTTTGAGTTCCTGGAAATCCTGAAGCAGGTTGCCAGGGACAACACCGATAATCCCGACCTGAGCATTGTGTGGATTGACCCCGACG
ACTTTCCTCTGCTGATCACTTATTGGGAGAAGACCTTCAAGATTGACCTGTTCAGGCCACAGATCGGCGTGGTGAACGTCACAGACGCT
GACAGCGTCTGGATGGAGATCAGAGACGATGATGATCTGCCCTCAGCCGAGGAGCTGGAGGACTGGATAGAAGACGTGCTTTCTGGGAA
GATAAATACTGAAGACGACGATGATGATGATGACGATGATGATGACGACGATGATGATGACGATGACGATGATGATGATGATGATGACG
ACGATGATGACGACGACTAATTGTGACTCTGTACAGTTTGACTTGTGGGGGCCGAGAGGCCTCCCCCCGCGGCAGGTCCCTCCGTTGGA
AGACCTGTGTTTGAGCGTCAGCAAGCACCGCCTGCTCCTCCTTTCCCCCCTGCCCCGCTCTCCCCGCCCTTGCTGGGACCCCCGGCCTG
ATTCTCAGCGGTGCTGAGCCAGCAGAACTGCCCCCGCAGCGGGTGGCACCGGCAGGCACCCAGCACCGCTGTAAATCATGCCTCCTTAG
GAAGGACAATAGGAATCTGCAGGGAACCTGCCCCAGTGCCCAGTGCAGGAGGAAAGCGCCCGCCTGCTCGCTGCCAGAGGGACACAGGG
AGCCAATTTGCATTTTCCGTGCTCATTGGCCCCGCATGCACATGCAAAGACCAAGTCTCACTCCAAGACATAGGGAAGGTCACTGTAAA
GTTTAGCCCTTTTATTAAACAAAATGCCCTTTGCAGCTACTCTGCCAAGACCACCGACTCTTGCAAGTTCTGCTGCACGAGACCATAGC
AACTCATTAACATGCTAGGAGCAGCAAAAAGGTTTGGGAGGGGAGGAAGGTTTAAGGGACGCTAGTTAATTTTCAGAGTCCATAAAACA
ACAGGATACTCATATCAAGCAAAAGGTAGGGCAGGTGGTGTGATACAGGACATCACAGCTTGGAGGTTCTTGAATTTGGAAGTTTTTGA
ATGCTGGAAAGGCTGATTCAGGCCTGAACCCAAACTCTGGAGGCTGAACACCACAGCACTGAACCAAACCATTGGTTCTAAAACTCAGA
GAAACACCAGAGCAGACCCCAGGCCAGTTACAAACTTCTTCAGCTAAGTCTGTGCTGGGGCTACCTTCCCTTTCAGGTGAGCTTCAGCT
GGATTAAAGGTGGTTCACAAGATAGCATACTCCTGCCCATGGAAACATTCTGAGGGGCTCACCCTGAAGCCCCCTATTCCCCATGACCT
TCCAAGCTTTATCTGACTTTTAACTTAAATGCCACATAGCATCTTTTCCCATGACCAAGCTGCTAGGAGGGAACATATCCATCCAACCA
CATTAGATGCTGGATAAAGTGATTTGGGCTATGAGCAGCGAAAGCATTCGAGCTCTTTGGCCTTAACATATTCACTTGCAAAGTCGCTC
CCTGCTTAGAGGCTGAGATGTCCCATGGTAGCCTGTGAGAAGCTCTTTTTACTGCAGACTGAGGAGATCAAGGCGTCATTTCAAAGCCA
ACGGTGTCACATCATACAAAGCTACTCAGTCCTAGCGAGTGCAGCTCCATCCATGTGCAGGTCACTTCAACAGTTAGGTCTCCCTGCTT
TCCCATGCCAGCAGATTTCAATTGTCTTAGCTTATTAGGTTTGACAATAGTGCAATGAATGGAGAGCTTGGAGACAGCTTTATCTTTAT
AAATACATTTATAAATATATTTTCCAGTCATTCAGACTTGTTAGCAAAGATAGGGTACACATGCCCTCTCTTGAAACAGACAAAAAACA
TTGTTCTGGTCTTTCTTTTACTGACTCATGTCAGAGATAAGTGATGTTAGTGGAAATTACAGCGCAAGGGGCAGGAGAATCAGAGCTCA
CTATATGAATGCGAATTAGCCTGATGAGAAACAAGGTTTACACACCATATATTTCCTTTACTGGATGAACTGCAAAGCAAGATTTTACA
GATTTCTTTTTTTTCCTGCCTGTTGGTCAGTCTCTTTGTCTCTCTCTTTCTCTCTATACATGAGCTGAAATAGAAATTGGAAATTACTG
GTATAAAAAGGCTGAAGTACTGATATACTTCTACATGTGCCATGTCCCATTGGTGGAAGGTGCCAGCATGAACACGCAGCTATATATCT

GTGTATCTATCTCTATGCATATGAATGAATACTGTGCTGACGTGGTAGATGACTGTAGGCTGTGCTACCTCCTCATTGAAGATCAAACA
GTCAAACAGAGCACTTCTGAGGCACAATCTGCTAGACTAATAGACAGGACAGCAAAGCCCCAGGTCAGCTATTATATGTTTGCCCTTTC
TTGCTTCTTTATGATGCATAAACACTGCCTGGAAGCCTCAAGCCAAGTGTGTTTGGGGAAACCATGGGGGTGTTCACACTGGCATTGAG
AACAACTCGGGCCCATCCCAGAGGACTTGGTGGATGGAGACTTTTGGAAGGCTTGAAATGCAGTGTGCTCTCAGGTTCCCTCCTCCATG
CACCAGCCTTCTGCTCTTCCCTTTAAATAATTTCAACCAGGGGTTTGCTGGAGAACCCCAGCTTAGAGAGAGAGGGAGCAAGCTGGACT
CAAGACTCCTTCATGCACATAGGTAAGAAACAAGAACAGGCTAAACTGGCAGTTTAGTAGTCTCTTAGTCTGTGATTAAGGGACAAATG
CTGCTTATGTTGCTCACATAAGTACATGCTTATCTTTACCCTGGGGCTATTCTCAGGAGTAAAGTGTGCAAGATTTCATCCTGCACATG
GTAACTTCTTTAGCAGCCTACAAAGGCCCTGCAGCACCAGGTCAGTCATTGCAAAGTCCCATCACTGTCTATAAACATGAAGAATAATG
AAGAATTACTCTATTACCTTTCCCAAATGGCACTGTCCCTGGGCATCCCAATTGCCTGTGTACTTTCCATACAGTACCTCTCCTCCCAC
ACAGGATATCCTTCTAGTCTGATATGCCCTGTGCGTATCTGATGCTCTGAGGACATAACTTAGAAGATACAGACCCTGCAAATAGCACT
GCAGATGTGTTCAGGTGCACAGGTGTTTCACAGGCACACATATGTTTCAGTGTATCAAGCAACAGAGCTTTGGGGAAAAAAATAAATTA
GTTGTGCTTAGGCTTATAAAGATGCTAGAAAGTCTGAGCTCATACTGCACCTGATGGCCTGGTGTTGGTGATTTATGGAAAATTTCCCT
TGAGTTATGAGAGAAAGGGCTGCCACTCATATGGCTGCCACTCTTGATGGGGCCCTCTTTCTCTCTTCCATTCGTCCCGTTACAGTTTC
TTCTAGTTCTCTTTGCCCACAAAGAGGATCATTTTATCTTCATCTTCTACTTACACAGCCAGAAGTGCCCAGTCCTCTTGACCGCCTTC
TTTTGGGATAAACCCGTAAAATTTAAAAGGTGTTGCAGAAGAGGGAGGATTGCAGCAGTGAAGAACGGCAGGCAGCAAAGTCTCTCCCC
TTTGCCCCTAGCACCCACGTTTGCAGTGACTGAACCACTGAACAAAGCAAGGCCCCTCATGAAGGAAATGGCAACTTGAATTTGAGTGC
CTTCCTTGCAGGGACAGGTAAGATACCCAAGTGTTGTGGGTTATGTCTGATGAGGTGGGGGCAGCTCTGCGCAAAACCACTAAAATATT
TTAGTTTCCCAATATATGACCAGTGAAAACAGCTTGCTGGTCCATACTGGCACCAGCAGACCTCGGCCTCCTCCATCTTACAATGGCTA
CACCCAGAGCACTGGGAGGTACTGGTGGGTGATGGCTTTTTCGCTCACAGCTGGAGACCAGGCTGACTTGGTCCTTAGGATGTGCAGGA
TGGCTCACGTGAAACGAGAGTCCCTCCCTCGTCCCAGCGAAAAAGCTCCTTTGCTGTCGAACACATGGCAAATTCAGGAGTGGGGAGGT
CCTGGGCTGTTCTGACAAAAGCACACACAACTGGCACGTCAATAGCAAAGGCCAGTACATTTGGATGAGGAACCAGCAAAAAAAATCACC
TTCTTTATTTTAAAAAAAAGTGTGTTTGTGCACGTGGGCAGCACTGAGTGTTTCCACAGGCAAGTCTGTTAAGACTTTCCCTGTCTTAA
CAAACAGTTCTTAATTGTTATTTAAAAAAAAAAAATCTTAAAAAAAAATCTTAAGCTTTAGTTAGATATTTATACTAAATGGATCTTAATA
TGTACAATGAAATACACCCGCG

>CASQ2_Anser_indicus_CDS
ATGAAGGCAACTTGCTGGATTCTGGCAGGCTTTTGCCTGCTTTTCTGCTGCAAGGCAGAAGAGGGACTGAATTTCCCCACCTATGATGG
GAAAGACCGAGTGATCGACCTGAATGAGAAGAACTACAAGCAGGCCCTGAAGAAGTATGACATGCTCTGCCTGCTCTTCCACGAGCCCG
TGAGCTCCGACAAGGTCTCCCAGAAGCAGTTCCAGATGACGGAGATGGTCCTGGAGCTGGCAGCTCAGGTCCTGGAGCCCAGGAGCATC
GGGTTTGGGATGGTGGACTCCAAGAAGGATGCCAAACTTGCCAAAAAGCTAGGCTTGGTTGAAGAGGGAAGTCTCTACGTCTTTAAGGA
GGAGCGGTTGATTGAATTTGATGGGGAGCTGGCCACAGATGTCTTGGTGGAATTCCTCTTGGATCTGCTAGAAGACCCCGTGGAGATCA
TAAACAGCAAGCTGGAGCTTCAGGCCTTTGACCAAATTGATGAAGAAATCAAACTCATTGGCTACTTCAAAGGAGAAGACTCTGAACAT
TACAAGGCATTTGAGGAAGCTGCTGAACACTTCCAGCCCTACATCAAATTCTTTGCCACATTTGACAAAGGGGTCGCCAAGAAGCTAGG
TCTGAAGATGAACGAGGTGGACTTCTACGAACCGTTCATGGATGAGCCTGTTCACATCCCCGATAAGCCTTACACGGAAGAGGAGCTGG
TTGAATTCGTGAAGGAGCACAAAAGAGCCACCTTGCGGAAGTTGCGTCCAGAAGACATGTTTGAGACGTGGGAGGATGACATGGAAGGA
ATCCATATCGTGGCCTTCGCTGAAGAAGATGACCCAGACGGCTTTGAGTTCCTGGAAATCCTGAAGCAGGTTGCCAGGGACAACACCGA
TAATCCCGACCTGAGCATTGTGTGGATTGACCCCGACGACTTTCCTCTGCTGATCACTTATTGGGAGAAGACCTTCAAGATTGACCTGT
TCAGGCCACAGATCGGCGTGGTGAACGTCACAGACGCTGACAGCGTCTGGATGGAGATCAGAGACGATGATGATCTGCCCTCAGCCGAG
GAGCTGGAGGACTGGATAGAAGACGTGCTTTCTGGGAAGATAAATACTGAAGACGACGATGATGATGATGACGATGATGATGACGACGA
TGATGATGACGATGACGATGATGATGATGATGATGACGACGATGATGACGACGACTAA

>CASQ2_Anser_indicus_protein
MKATCWILAGFCLLFCCKAEEGLNFPTYDGKDRVIDLNEKNYKQALKKYDMLCLLFHEPVSSDKVSQKQFQMTEMVLELAAQVLEPRSI
GFGMVDSKKDAKLAKKLGLVEEGSLYVFKEERLIEFDGELATDVLVEFLLDLLEDPVEIINSKLELQAFDQIDEEIKLIGYFKGEDSEH
YKAFEEAAEHFQPYIKFFATFDKGVAKKLGLKMNEVDFYEPFMDEPVHIPDKPYTEEELVEFVKEHKRATLRKLRPEDMFETWEDDMEG
IHIVAFAEEDDPDGFEFLEILKQVARDNTDNPDLSIVWIDPDDFPLLITYWEKTFKIDLFRPQIGVVNVTDADSVWMEIRDDDDLPSAE
ELEDWIEDVLSGKINTEDDDDDDDDDDDDDDDDDDDDDDDDDDDDDD*

6.2 *Aptenodytes patagonicus* (king penguin)

From: Trinity *de novo* assembly of SRR1324788. No CASQ1 was assembled. CASQ2 from complement of TRINITY_DN1485_c0_g1_i1.

>CASQ2_Aptenodytes_patagonicus_transcript_assembly
-GGTTGTGAGCCCAGGACAGCAGAGTTTGCTGCTCCCCACCAGCCTTGCAGAGTTTGGGCTGATCACTAGGAAGGGAGCAGAAAGAGAA
ACTCGGTTCGAACTTCACCTGGAGCCTTTGGTCTCCCCCTCTTCCCAAATTAAGAAGGAGAGGGACGTTTACCTGCCCTCTGCTGCACT
ACAAGGGCTCCCCTATGAAGGCGACTTGCTGGATCCTGGCAGGTTTTTACCTGCTTTTCTGCTGCAAAGCAGAAGAGGGACTGAATTTC
CCCACCTACGATGGGAAAGACCGAGTGATTGACCTGAACGAGAAGAACTACAAGCAGGCCTTGAAGAAGTACGACATGCTCTGCCTGCT
CTTCCACGAGCCTGTGAGCTCTGACAAGGTCTCCCAGAAGCAGTTCCAGATGACAGAGATGGTCCTGGAGCTGGCAGCTCAGGTCCTGG
AGCCCAGAAGCATTGGCTTTGGGATGGTGGACTCCAAGAAGGATGCCAAACTTGCTAAAAAGCTAGGCTTAGTTGAAGAGGGAAGTCTC
TATGTCTTTAAGGAGGAGCGGTTGATTGAATTTGATGGGGAACTGGCCACAGATGTCTTGGTGGAATTCCTCTTGGATCTGCTAGAAGA
CCCCGTGGAGATCCTAAGCAGCAAGCTGGAGCTTCAGGCCTTTGACCAAATCGATGACGAAATCAAACTCATCGGCTACTTCAAAGGAG

AAGACTCTGAACATTACAAAGCATTTGAAGAAGCCGCCGAACACTTCCAGCCATACATCAAGTTCTTTGCCACCTTCGACAAAGGGGTT
GCCAAGAAGCTAGGTCTGAAGATGAATGAGGTGGACTTCTATGAACCGTTTATGGATGAGCCTGTTCACATCCCTGATAAGCCTTACAC
AGAAGAGGAGCTGGTTGAATTTGTGAAGGAGCACAGAAGGGCCACCTTGAGGAAACTGCGTCCAGAGGACATGTTTGAGACGTGGGAGG
ATGACATGGAGGGAATCCACATTGTGTCCTTTGCCGAAGAAGACGACCCAGATGGTTTTGAGTTCCTGGAAATCCTGAAGCAGGTTGCC
AGGGACAACACCGATAATCCCGACCTGAGCATTGTCTGGATTGACCCCGACGACTTTCCTCTGCTGATCACTTACTGGGAGAAGACCTT
CAAGATTGACCTGTTCAGACCACAGATCGGGGTGGTGAACGTCACAGACGCTGACAGCGTCTGGATGGACATCAGAGATGACGATGACC
TGCCCTCGGCCGAGGAGCTGGAGGACTGGATAGAGGACGTGCTTTCTGGGAAGATAAATACCGAAGATGATGATGATGACGACGATGAT
GATGACGACGATGATGACGACGACGATGATGACGACG

>CASQ2_Aptenodytes_patagonicus_CDS_partial
ATGAAGGCGACTTGCTGGATCCTGGCAGGTTTTTACCTGCTTTTCTGCTGCAAAGCAGAAGAGGGACTGAATTTCCCCACCTACGATGG
GAAAGACCGAGTGATTGACCTGAACGAGAAGAACTACAAGCAGGCCTTGAAGAAGTACGACATGCTCTGCCTGCTCTTCCACGAGCCTG
TGAGCTCTGACAAGGTCTCCCAGAAGCAGTTCCAGATGACAGAGATGGTCCTGGAGCTGGCAGCTCAGGTCCTGGAGCCCAGAAGCATT
GGCTTTGGGATGGTGGACTCCAAGAAGGATGCCAAACTTGCTAAAAAGCTAGGCTTAGTTGAAGAGGGAAGTCTCTATGTCTTTAAGGA
GGAGCGGTTGATTGAATTTGATGGGGAACTGGCCACAGATGTCTTGGTGGAATTCCTCTTGGATCTGCTAGAAGACCCCGTGGAGATCC
TAAGCAGCAAGCTGGAGCTTCAGGCCTTTGACCAAATCGATGACGAAATCAAACTCATCGGCTACTTCAAAGGAGAAGACTCTGAACAT
TACAAAGCATTTGAAGAAGCCGCCGAACACTTCCAGCCATACATCAAGTTCTTTGCCACCTTCGACAAAGGGGTTGCCAAGAAGCTAGG
TCTGAAGATGAATGAGGTGGACTTCTATGAACCGTTTATGGATGAGCCTGTTCACATCCCTGATAAGCCTTACACAGAAGAGGAGCTGG
TTGAATTTGTGAAGGAGCACAGAAGGGCCACCTTGAGGAAACTGCGTCCAGAGGACATGTTTGAGACGTGGGAGGATGACATGGAGGGA
ATCCACATTGTGTCCTTTGCCGAAGAAGACGACCCAGATGGTTTTGAGTTCCTGGAAATCCTGAAGCAGGTTGCCAGGGACAACACCGA
TAATCCCGACCTGAGCATTGTCTGGATTGACCCCGACGACTTTCCTCTGCTGATCACTTACTGGGAGAAGACCTTCAAGATTGACCTGT
TCAGACCACAGATCGGGGTGGTGAACGTCACAGACGCTGACAGCGTCTGGATGGACATCAGAGATGACGATGACCTGCCCTCGGCCGAG
GAGCTGGAGGACTGGATAGAGGACGTGCTTTCTGGGAAGATAAATACCGAAGATGATGATGATGACGACGATGATGATGACGACGATGA
TGACGACGACGATGATGACGACG

>CASQ2_Aptenodytes_patagonicus_protein_partial
MKATCWILAGFYLLFCCKAEEGLNFPTYDGKDRVIDLNEKNYKQALKKYDMLCLLFHEPVSSDKVSQKQFQMTEMVLELAAQVLEPRSI
GFGMVDSKKDAKLAKKLGLVEEGSLYVFKEERLIEFDGELATDVLVEFLLDLLEDPVEILSSKLELQAFDQIDDEIKLIGYFKGEDSEH
YKAFEEAAEHFQPYIKFFATFDKGVAKKLGLKMNEVDFYEPFMDEPVHIPDKPYTEEELVEFVKEHRRATLRKLRPEDMFETWEDDMEG
IHIVSFAEEDDPDGFEFLEILKQVARDNTDNPDLSIVWIDPDDFPLLITYWEKTFKIDLFRPQIGVVNVTDADSVWMDIRDDDDLPSAE
ELEDWIEDVLSGKINTEDDDDDDDDDDDDDDDDDDDX

6.3 *Apus apus* (red-crested pochard)

From: Trinity *de novo* assembly of SRR21777135. CASQ1 from TRINITY_DN776_c0_g1_i43. CASQ2 from TRINITY_DN8210_c0_g1_i3.

>CASQ1_Apus_apus_transcript_assembly
TGGAGCTGGAGGCGCCGGGAGGCGGGGGGGGAAGAAGATGAGACCCCCGGACCCCCCGCGTCCCCCGGGGCCCTGAGCCCCCCGGGGCC
GGGGGAGGGCGAGGGATGCAAAGGGTCAGGGAGACACTGTCCCCCCCTTGGTGCCTTCGGCCACTCGTGGGCTGGGAAGGGGCCTCGTG
TCCGTGTGTCCTCCCCCCCCGGCGGTGTCGGTCTGTCCCGGGGGCCGGAAGGGGCCGTGTCCCTCAGTCCGTCCCCCGGGGCCGTGTCC
GTCCGTCCCCTGCGCTGGTCCCGGCCGGGTTCTCTCTGTCTGTCCGTCCCGGGACACCGAGGCTGTGTCTGTCTGTCCCTTCTCAGGGG
CTGTCCGTTTGTCTGTCCGTCTGTCCCAGCTCTGTCTGTCCGTCCGGTCTCTGGCTGTGTGTGTCCGTCTTGTCCCCTGCTTGTCCGTG
AGTCCCATCCATCCTCCCCCCGTCCCCGTTCGTCCGTCTGTCCGTCCCTGCCCGTCTGTCAGCCCGGTGAGCGATGGGGCTCTGGGGGT
GGGCGCTGGTGCTGGCTGCGCTGTGGGCCGGGGTCCCGGGGGGATCCGGGGGGTCCGGAGGGGGGCTGGCTATCCCCACCCCCGACGGG
CTGTCCCGGGTGGTCCCCGTCACCCTGAGGAACTACAAGGGGCTTCTGCAGCGGTTCCCGGTGCTGGCCGTGCTGCACCGGCCGGGCCG
GGGCCACCACGAGCAGCCGGAGCTGGTCCTGGAGCTGGCAGCCCAGGTGCTGGAGGACAAGGGGGTGGGCTTCGGCCTCATTGACTCTC
AGGAGGATGCAGCTGTCTCCAAAAAGCTGGGTCTGACAGAAGAGGGGAGCATCTATGTGTTTAAGGAGGACGAGGTGATTGAGTATGAC
GGGGAGCTGGCAGCTGACACACTGGTGGAATTCCTGCTGGAGGTACTGGAGGACCCGGTGGAGTTTATTGATGGTGACCACGAGCTCCG
GGCCTTTGAGAACATTGAGGATGATCCCAAACTCATTGGATATTTCAAGAATGAGGACTCAGAGCACTTCAAGGCCTTCCAGGCAGCAG
CGGGGCAGTTCCACCCCTTCATCTCCTTCTTCGCCACCTTCGACAGCAAGGCAGCCGAGCAGCTGACCCTGAGGCTGAATGAGATCAAC
TTCTATGAGCCCTTCATGGAGCAGCCGCTGCCCGTGCCCGGGCCCAGCTCCAGGGAGCAGATCGTGGCCTTCGTGGAGGAACATCGGAG
GGCCACTCTGCGGAAACTCAAACCCGAGAGCATGTACGAGACCTGGGAAGACGACATGGATGGGATCCATATCGTGGCGTTTGCAGAGG
CGGATGATCCAGATGGGTTTGAGTTCCTGGAGATCCTGAAGGAGGTGGCCCGGGACAACACGGACAATCCCGACCTCAGCATCCTCTGG
ATTGACCCTGAGGATTTCCCACTGCTCATCCCTTACTGGGAGGAAACGTTTGACATCGACCTGTCCCGGCCCCAGATTGGGGTGGTCAA
CGTCACTGATGCTGACAGCGTGTGGATGGAGATGGAGGATGAGGATGATTTGCCCGGACCAGAGGAGCTGGAGCAGTGGCTGGAGGATG
TGCTGGCAGGAGAGATCAACACTGAGGATGATGACGACGACGATGATGATGACGACGATGATGATGACGACGATGATGAAGATGATGAC
TCTGAAGTTAAAGAAGAAAATGGTGTATTGGTCTTGAATGATGCAAACTTTGATACCTTTACTGCAGACAAGGACACTGTGCTGCTGGA
GTTCTATGCACCATGGTGTGGGCACTGCAAGCAGTTTGCTCCTGAATATGAAAAGATAGCCAAAACACTGAAGGAAAATGACCCTCCTA
TTCCAGTTGCCAAAGTAGATGCTACTGCAGCCACTTCACTAGCAAGTCGTTTTGATGTCAGTGGCTACCCAACCATCAAAATCCTGAAA
AAAGGCCAGCCTGTTGACTATGATGGTTCTCGGACAGAAGATGCCATTGTGGCCAAAGTCAAGGAGGTTTCTGATCCAAATTGGACCCC
TCCACCAGAAGCTACCCTGGTATTGACCCAGGATAATTTTGACGACGTTGTGAATGGTGCTGACATAATCCTGGTGGAGTTCTATGCTC

CATGGTGTGGACACTGCAAAAGGCTTGCTCCAGAATATGAGAAGGCTGCCCAGGAGCTCAGCAAGCGCACACCTCCTATTCCCCTGGCT
AAAGTCGATGCCACTGCTGAAACTGAGCTTGCAAAGAAGTTTGATGTTACTGGCTACCCAACTCTGAAAATCTTCCGCAAGGGCAAACC
TTATGACTACAGTGGTCCACGGGAAAAATATGGTATTGTTGACTACATGATTGAACAGGCTGGTCCTCCATCCAAACAGATTCAGGCTA
CCAAGCAGGTACAAGAATTTCTGAGGGATGGGGATGATGTCATCATCATTGGTGTCTTTAGTGGAGAGAATGACAAAGCCTACCAACTC
TATCAGGAAGCAGCTAATGGTTTAAGAGAAGATTACAAGTTCCACCACACCTTCAGCAGTGAGATTGCAAAGCTATTGAAGACATCTCC
AGGAAAACTGGTTGTCATGCAGCCAGAAAAATTTCATTCAAAGCACGAGCCCAAGATGCATGTTTTGGATCTTAAACAGGATTCTACAG
ATGGATCAGAGATTAAAGAGCACGTGCTAAAACATGCTTTGCCTCTAGTTGGTCATCGCAAGCCTTCCAATGATGCTAAAAGATATGCA
AAGCGTCCTCTAGTGGTTGTCTATTATTCTGTAGACTTCAGTTTTGACTACCGTGTTGCTACCCAGTACTGGAGAGGCAAAGTCCTGGA
AGTGGCCAAAGACTTCCCTGAATATGTTTTTGCTGTTTCTGATGAGGAAGACTATTCTTCTGAAATAAAAGACTTGGGCCTTCTTGAGA
GTGGAGAGGATGTCAATGTTGCCATTCTGGATGAAGGTGGCAAGAAATACGCCATGGAGCCAGAGGAGTTTGACTCTGATGTACTCAGG
CAGTTTGTGCTGGCATTCAAAAAAGGAAAACTGAAGCCTATTGTGAAGTCCCAGCCAGTGCCAAAAAA

>CASQ1_Apus_apus_CDS
ATGGGGCTCTGGGGGTGGGCGCTGGTGCTGGCTGCGCTGTGGGCCGGGGTCCCGGGGGGATCCGGGGGGTCCGGAGGGGGGCTGGCTAT
CCCCACCCCCGACGGGCTGTCCCGGGTGGTCCCCGTCACCCTGAGGAACTACAAGGGGCTTCTGCAGCGGTTCCCGGTGCTGGCCGTGC
TGCACCGGCCGGGCCGGGGCCACCACGAGCAGCCGGAGCTGGTCCTGGAGCTGGCAGCCCAGGTGCTGGAGGACAAGGGGGTGGGCTTC
GGCCTCATTGACTCTCAGGAGGATGCAGCTGTCTCCAAAAAGCTGGGTCTGACAGAAGAGGGGAGCATCTATGTGTTTAAGGAGGACGA
GGTGATTGAGTATGACGGGGAGCTGGCAGCTGACACACTGGTGGAATTCCTGCTGGAGGTACTGGAGGACCCGGTGGAGTTTATTGATG
GTGACCACGAGCTCCGGGCCTTTGAGAACATTGAGGATGATCCCAAACTCATTGGATATTTCAAGAATGAGGACTCAGAGCACTTCAAG
GCCTTCCAGGCAGCAGCGGGGCAGTTCCACCCCTTCATCTCCTTCTTCGCCACCTTCGACAGCAAGGCAGCCGAGCAGCTGACCCTGAG
GCTGAATGAGATCAACTTCTATGAGCCCTTCATGGAGCAGCCGCTGCCCGTGCCCGGGCCCAGCTCCAGGGAGCAGATCGTGGCCTTCG
TGGAGGAACATCGGAGGGCCACTCTGCGGAAACTCAAACCCGAGAGCATGTACGAGACCTGGGAAGACGACATGGATGGGATCCATATC
GTGGCGTTTGCAGAGGCGGATGATCCAGATGGGTTTGAGTTCCTGGAGATCCTGAAGGAGGTGGCCCGGGACAACACGGACAATCCCGA
CCTCAGCATCCTCTGGATTGACCCTGAGGATTTCCCACTGCTCATCCCTTACTGGGAGGAAACGTTTGACATCGACCTGTCCCGGCCCC
AGATTGGGGTGGTCAACGTCACTGATGCTGACAGCGTGTGGATGGAGATGGAGGATGAGGATGATTTGCCCGGACCAGAGGAGCTGGAG
CAGTGGCTGGAGGATGTGCTGGCAGGAGAGATCAACACTGAGGATGATGACGACGACGATGATGATGACGACGATGATGATGACGACGA
TGATGAAGATGATGACTCTGAAGTTAAAGAAGAAAATGGT

Note: There was not a stop codon found.

>CASQ1_Apus_apus_protein
MGLWGWALVLAALWAGVPGGSGGSGGGLAIPTPDGLSRVVPVTLRNYKGLLQRFPVLAVLHRPGRGHHEQPELVLELAAQVLEDKGVGF
GLIDSQEDAAVSKKLGLTEEGSIYVFKEDEVIEYDGELAADTLVEFLLEVLEDPVEFIDGDHELRAFENIEDDPKLIGYFKNEDSEHFK
AFQAAAGQFHPFISFFATFDSKAAEQLTLRLNEINFYEPFMEQPLPVPGPSSREQIVAFVEEHRRATLRKLKPESMYETWEDDMDGIHI
VAFAEADDPDGFEFLEILKEVARDNTDNPDLSILWIDPEDFPLLIPYWEETFDIDLSRPQIGVVNVTDADSVWMEMEDEDDLPGPEELE
QWLEDVLAGEINTEDDDDDDDDDDDDDDDDDEDDDSEVKEENG

>CASQ2_Apus_apus_transcript_assembly
CAGCTATGTAAGGTATCCAGGGCTAGGCCTGACAGCTTCTCCTGTCTGTCTCTTTGTTGTGAGTCCAGGACAGCAGAGTTTGCTGATCC
CCAGCAGCCTTGCAGAGTTTGGGCTGATCACTAGGAAGGCAGCAGAAAGAGAAACTCAGTTCGAACTTCACTTGGAGACTTTGGTCTCC
CCCTGTTCCCAAATTAAGAAGGAGGGAGACGTTTACCCTTCCTCCACTGCACTCCCAGGGCTCCCCCATGAAGGCGACTTGCTGGATCC
TGGCAGGTTTTTACCTGCTTTTCTGCTGCAAGGCAGAAGAGGGACTGAACTTCCCCACTTATGATGGGAAAGACCGAGTGATTGACCTG
AACGAGAAGAACTACAAGCAGGCCCTGAAGAAGTACGACATGCTCTGCCTGCTCTTCCACGAGCCTGTGAGCTCTGACAAGGTCGCCCA
GAGGCAGTTCCAGATGACAGAGATGGTCCTGGAGCTGGCAGCTCAGGTCCTGGAACCCAGGAGCATTGGCTTTGGGATGGTGGACTCCA
AGAAGGACGCCAAACTTGCTAAAAAGCTAGGTTTGGTTGAAGAGGGGGAGTCTCTATGTCTTTAAGGATGAGCGGTTGATTGAATTTGAT
GGGGAACTGGCCACAGATGTCTTGGTGGAATTCCTCTTGGATCTGCTGGAAGACCCTGTGGAGATCATAAACAGCAAGCTGGAGCTTCA
GGCCTTTGACCAAATCGATGATGAAATCAAACTCATTGGCTACTTCAAAGGAGAAGACTCTGAACATTACAAGGCATTTGAAGAAGCTG
CAGAACACTTCCAGCCATACATCAAGTTCTTTGCCACCTTTGACAAAGGGGTTGCCAAGAAGCTAGGTCTGAAGATGAACGAGGTGGAC
TTCTATGAACCATTTATGGATGAGCCTGTTCACATCCCTGATAAACCTTACACAGAAGAGGAGCTGGTTGACTTTGTGAAGGAGCACAA
AAGGGCCACCTTGAGGAAACTGCGCCCAGAGGACATGTTTGAGACGTGGGAGGATGACATGGAGGGAATCCACATTGTGGCCTTTGCCG
AAGAAGATGACCCAGATGGTTTTGAGTTCCTGGAAATCCTGAAGCAGGTTGCCAGGGACAACACTGATAATCCTGACCTGAGTATTGTC
TGGATTGACCCTGACGACTTTCCTCTGCTGATCACTTACTGGGAGAAGACCTTCAAGATCGACCTGTTCAGACCACAGATCGGGGTGGT
GAATGTGACAGATGCTGACAGCATCTGGATGGAGATCAGCGATGATGACGACCTGCCCACAGCTGAGGAGCTGGAGGACTGGATAGAAG
ATGTGCTTTCTGGGAAGATAAACACTGAAGACGACGACGACGATGACGACGACGACGATGACGACGACGACAATGATGATGACGATGAC
GACGACGATGATGACGACGACGACGACGATGATGATTAACTCTGACTCTGTGCAGCTTGACTTGTGGGGGCTGAGAGGCCTCGCCCCGT
GGCAGGTCCCTCCAGCAGAAGACCTGTGTTTGAGCGTCAGCAAGCACCGCTGCTCCTCTCCCCACCCTGCCCTGCTCCCCCTGCCCTCG
CCGGGACCCCCCAGCCTGATTCTCAGCGGTGCTGAGCCACCAGGACTGCCCCTGCGGCAGCACCGCTGCAAATCATGCCTCCTTAGTAA
GGACAATAGGAATCTGCAGGGAATCTGCCCCCAGTGCCCCAGGGCAGGAGGAAACTGCCTGCCTGCTCGCTGCCAGAGGGACACAGGGA
GCCAGTTTGTATTTTCCATGCTGATCGGCCCAGCACTTAACATCCAAAGACCAAATCTCACTTCAAGACATAGGGAAGGCCATTGTGAA
GTTGAGTCCTTTTATTAAACACAACGCCCCTTGCAGCTGCTCTGCCAAGACCACTCCATCCTGCAAGTTCTGCTGTGGGAGTCTGTAGC
TACTCATTAACATGCTAGGAGCAGAAAAAAAAGCTTTGGGAGGGGAGGAAGGTTTCAGGGGTGTTATTTAAGCTTTCAGCGATTCTATGT
AACAACAGAATTTTCTTGTCTGGCAGTGCAATGCAAAACCAAACACTATGGTCCCTGTCTGAACCTCTTTCTTCTTGAAAGCTCAGGCC

AAAGATAGGGTAAGTGCAAGTGGTCTGATACAGGACATCAAAGCTTGTGAGTTGAGGAACTGCTGGAAAAGCTGATCCAGGTCTGAGCC
CAAGCTCTTGAGGCTGAAGATGATAGCTCTGAGCCAAACCATTGGTCTTAACCCCAAAGAAACACCAGAGCATATGCCAGGCCAGTGCC
AAACACCAGCAGCCAGGTCTGGGCTGGGCCTACCTTGTGTTTCAGCTGAACTGCAGTGGGACTGAAAGTGGTGCACAAGAGACAGACAG
TATACTTCTGCCAGTGGAAATTTTCATAGGGGCCCACCCTGAAGCTCTCTGATTCCCCATGACCTTTCAGCTTTAGCTGATTTTTAATC
CAAAGCCTACCCAGCAGCTTTTCCTATGACCGAGATGTTGGAAGGGAACACATCCATCCAACTATAGTAGATGCTGGAAAATGAAAGTT
GGTCAACAAGCAGTGAAACGATTCAAGCTGTTTGGCTTTTAAGTATTTAGTTGCACAGTTACTCCCTGCTTAAGAGACTCAGATGTCCC
ATGGCAGCCTGCAAGAAGCCTCTTTTGACTGCAGACTGGAAAGGAGCACCGGGCACCAGTTGGCAGTCGAGGGGGTCACTCCCAAAGCC
AAGGGTCAGCAGCAACTCAGTCCTGGCGAGTGCAGCACCATCCACGTGCAAGTCACTGCGACAGTGTTAGGTCTCCCTGCTTCCCCATG
CCAGCAGATTTCAATTGTCTTAGCTTATTAGGTTTGACAATAGTGCAATGAACGGAGAGTTTGGAGAACGTTTCATCTTTATAAATACA
GTTATAAGTATGTTCTCCGGTTGCTCAGACTTGTTAGCATAGGTAGGGTACCCACATCCTCTCTTGCTGCAGACAAATAATTTTGTTCT
GACCTTATACTGGCTTAGGTCAGAGGTAAATGATGTTAGGGGAAAAATCAGTCCAAGGGACAGGAGAATTGGAGTTTGTTACACAAATG
CGAATCAGCCTGATGAGAAACAAGGTTTACACACTATATTTCCATTATTAGATGAACTGCAAAGCAACATTTTATATACTTTTATTTTT
CCTGCCTGTTGATCAGTCTCTTTGACTCTCTCTCTACGTTAGCTGAAATAGAAATTTGAAACTACTGATATAAAAAAGCTGAAGTACTG
ATATATTCCTAAGTGTGCCATATCCTCTTGGTGGAAAGTGCCAGCATGAACAATACACAGCTGGATATCTCTATATCTCTATGTATATG
AATGAATTCTGTGCTTCTATGGTATATGACTGTAGGCCACGTTAGCTCTTCATTGAACACTGAACACTCAGCCCTTGCTTTTCAGGCAC
AGCTTATTAGAATAGACAGGACAGCAAAGCCTCTATGTCAGCTATTCTCTTTTATATACTTTTCTGCTTCTTTACTATGGATAAGCACT
GCCTGGAAGACTTGAACCAAGTGAGTCTGGGGAGACCATCCACCTGGTCACAAGAGGGTTGTGGACAGCTCAGCCTCATCCCAGCGGAC
CTGCTGGAGACTTCCAGAAGGCTTGAAATGCAGAGTGCTCAGCCTGGAGCTGAGCACTTCACACACTTCTCCTTCACACACCTCTCTCT
CCTGGCCAGAGGCCTGCCCAGGTCCTTCCTTCTGAACTTTCCTTTCAGCTTTTTCCTCAAGAGGTTTACTGGGGGACCCTGGCTCAGAG
AGGAGGAGCAAGCAGGACACAAGGTCTCCCTGGTGCACATTGGTAAGGAGTGAGAACGGGCAAAAGGAGCAGTTTAGTAGTCTCCTTGT
CTGTGACTAATGGACAAATGCCACATACACTACTCACCTCTGCAAAGTACTTGTTTATCTTTACCCTGGGGCTGTTCCCAGGAGTTAAA
CTTAACACACAGTTTCACCCTGCATAAGGTAACAGCCTGCACAGGCCCTGCAGCTCCAGATCAGTCACCACAAAGAGTCGTGGGCTTCT
ATGAACATGAAGAATAAACAAAAAGTTGCTCTACTGCCTTTCCCAACTGGCACAGTCTCCAGACATCCCAATGGCCTCGGTACTCTCCA
TGTACCTCCCCTCCCACTGTGGATGCCTGTCTGTTCTGATGTGCCTCTCTGCAGATGCTCTAAGAGGCACAGCTTATGCAGGCAGACCC
TGCAAACAGCCCTAGGGAGGTGTTTGAGTGCTCAGGCACATACACAAACCAGTGCATCAAGCAACAGAGCTCTGGGGGGGAAGCAATTA
ATGTAGTGGTGCTTCAGCTCATACTGAGGCCAGTGGCTGGTGTCAGTGTTTTATGGAAAGTTTCCTTTGAGTTATAAGAAAAAGGGCTA
ATCCTATGGCTGCCACTGCTTCATGGTGCCAGAGCAGCCCTTGATGGGGCCACCCCTCCCCTCTTCCCCCCATTATTGTTTTCATGGTT
CTCTTTGCCCACCAACAGGAACTTTTTATCTTGAGCTTATGGGATAAACACATAAAAAGAAAGTGTGGTGGCATAGGAGGGATTGCAGC
AATGAAGAAAATGAGACAGCAGAGGCTCTCCCATTCACCCACAAAGCCTTGTCTTCCCAAAGTTATGGAGAGGCTCTGTGAACCAGAAG
CTGCTCCGAGCTAATGTCAACCCTTTGGGAAACAGTTCTGGAAGGAGAGGCCAAGGGAGGCATGCTGGAAGTGACAGATTACTCCGTAT
TTTCAGGGTCAGCCATTTTCGTTATGCTTTTGAGGAGTGGTGAGAGTCTCCTGTGCTGCCCCTGGAAGAGGAATAGACCTGTAGTGACA
CCAGAGATCTCTGCTGCTTCCATCCCACAACAGAGCTGCTCAGCCAGGTGCCAACATGCACACACAGCCGAGGACACCGGCTGCTTCTG
CATGTCTGTTCACACCTCATACCTCAGAAAGGACTGATTTAGTACTGAGGCCAAACAAAAAACCTTTGCCTTTTATTCCCAGAGAAACA
ATTAATTAGCCAGGAACATGTAACTTCCCAGCTGCTGACTTGCAGTGCTGTGAAAACAGGCCGGGATTGCACCTGGAATCCATTCTGCC
CCAGCAAAGTTTTGGGGACAGACCCCAGACAGCCATTTGCACCCTGATGCTTGGACTTGGGGAGGCTGAAGAGCAAGAAGGGCTCCAGT
GACAACAAGCACCAAGGCCCTCCAGGGCAAAGCAAACTGCCTCTGGGCTACTTTACATCCCAGGTATGCCTATGTTGTGCAGTACAATG
CCACCTGCTAGAAACTGGAAGCAGCAGAGCACAGCAAGAGGATAATTAACCTGGATAACGAGCCTGTGCCAGCAGAACTGGGTCCTACC
TTAGCCCCAAGACTGTGCAGCACTTCACTGCACTGCAGTATTCCCTGTCTTTGCCCTCTTCCCTTGTAGCACCAACATCTGGGCAAAGC
AGCCCTCCAAGGGTCCGTACCCCTTAGGGCCAGCAGTGGGATGGCTCTTACACCTGCTGGGCAGAAGAAGATGTCTAATGACAATAGAT
GTGGTTCAAGGCAGACCCAGCACTCTTGGCTGTGCTCCCAGAACACTCCCTGCCAAGTTTAAAAGGTGCTAAACTATTTAAAGGGGAAG
AAAAAAAAACACCAAAACAACACAAAAAACCCCAAAAGTACAGAGCTTCCTTGCAGCTTGAAGATCCATATCTGGAGTCAGATTACAAG
CAGCTTTTGGGATGGAAGCAAAGATCCTTCCGGTGCCTTGCTCCCCCTTTCAGCTTCCCCCTTGCATCTTCTCTTGCTGAGCCCCTGTG
ATGAAGGATGGTGAAAGACATCAGCAAAGCACTTGAAGGACTTCTGGTATTGTCCCTCATGATTTGGACCAAGAAGAAAACACACCATT
TTCACTTTCTTTTCAGCCCTCTTGACTCATTAATCTTCTGGTCTTTACTGTCTCCCCCAGTTTTCTTGCTTGCTCCATTTCTACCTAGC
CTGCCTTCCTGTTCTCCTCAAACCACCCCCACAGTGTATGGTATCGAGGGTTGCTTAAGGACAAGGCTCTCCCCTGTCACTTCTTCCTG
AGCATCTCCCCAGCCCAGGGAGCAGGCAGCCTCCCTCTTGCCACAGCCAGGAACCAACCGTGGTGGCCCCCATGACATGGGCCATGGCT
GTGAACACAAGGAGGGACATTTCCTTCCTAAGGAAAAAGACAACCGGGGAAGGCTGACACTGGCTTTCATGTTGCGGGGGACAGGTGGG
ACACACCCAAGGTGAAGCCATTTCACCAGCCTGGGCCCTGTGGAAATACCAGGAAATTTTTTTGGGGGGTTGGGTACTTTTTCTGATTG
CCCCAAAATCCAGATGAGAAACCATCACCCTTTAGCCAAGACCCAACTGCACAGGGAGTGGTTGGAACATCTTGGCTACCACCATGGCA
AGAGGTCAGTCACCCCAGTGCCACTGGTAGTCACACCACACCACCACCCAAGTAGAAGACAAGTGGCCTGACTTCACATACAAGAAATA
AGCAACAAGGGTAAAACAAAATGAGTTTGCTGCAGGAGTGATCCCATACTTCAACAGCAGCCAGAAAGTTAACTCACTGCCCAAGAAGA
CAGGCAGTTATCCTGACTCAATCCTCCTTCAGTTGTGGGTGGCAAATTACTTAAATAGCCACGGACAAGCTCATGCTCTGCTTTTGGAT
TTGGAAGAAGCCCAGCTTGAATCAGGTCCCTCCCTCAATAACGCAGGTAAAAATAAATTATGTTCGACAGGAGAACACCCTGGAAATTT
GCCTGATGTTAATTGTTTTGCTTTAAAATCCGATCAAACGTGTCAAAGCCCTGCTCCTGCCAGCGACAAAGAAAGAGGCTATTGAAAGA
CAAAAGTAACTAAATGTTTGGAGAACATTTTAGTCCCCGCAAAATGAAATCGCTGGAGGAATTTGCACAGCTTCCCACAGTCTTGTTAC
TGAATTACTAAACCAGTTTCTCCCAGCCACCTACAAAGCAAATTTATAATCAACTGTAGGATCTGGTATTATGAGTACTCAAGAATGAG
CTTTTACAAAATAGAAGCTAAAAGGTTGGCAGATCTGTTTCACATCTGTAGCCCAGATATAATCAGCTCCTTTTCCTCTTTTAAGGCAT
TACCACGGCATCTGACTTGTGCTGAAGAGAGGTCCAAAGAGCTAAAAAGTTCTGCCTTTTAAAACTTCCAGAGAGGCTGAATCAAATGT
ATTTTATTAAGAACTTCCTTCTCTCTGTGCATTTTTTCATTCATGTAACCTTTTCAAATAGTAAGAAGGAAAAAAACCAACAACAAACA
AACAAAAAGACAACCAAGACTGTAGACATTTAAGCACTTGTAAGCCTGATTTTTGCATTTGGAAGAGACTTAAAATGTTTCAATTTACA

GAGTAAATGCCTAGAGCAAAAAAAAAA

>CASQ2_Apus_apus_CDS
ATGAAGGCGACTTGCTGGATCCTGGCAGGTTTTTACCTGCTTTTCTGCTGCAAGGCAGAAGAGGGACTGAACTTCCCCACTTATGATGG
GAAAGACCGAGTGATTGACCTGAACGAGAAGAACTACAAGCAGGCCCTGAAGAAGTACGACATGCTCTGCCTGCTCTTCCACGAGCCTG
TGAGCTCTGACAAGGTCGCCCAGAGGCAGTTCCAGATGACAGAGATGGTCCTGGAGCTGGCAGCTCAGGTCCTGGAACCCAGGAGCATT
GGCTTTGGGATGGTGGACTCCAAGAAGGACGCCAAACTTGCTAAAAAGCTAGGTTTGGTTGAAGAGGGGAGTCTCTATGTCTTTAAGGA
TGAGCGGTTGATTGAATTTGATGGGGAACTGGCCACAGATGTCTTGGTGGAATTCCTCTTGGATCTGCTGGAAGACCCTGTGGAGATCA
TAAACAGCAAGCTGGAGCTTCAGGCCTTTGACCAAATCGATGATGAAATCAAACTCATTGGCTACTTCAAAGGAGAAGACTCTGAACAT
TACAAGGCATTTGAAGAAGCTGCAGAACACTTCCAGCCATACATCAAGTTCTTTGCCACCTTTGACAAAGGGGTTGCCAAGAAGCTAGG
TCTGAAGATGAACGAGGTGGACTTCTATGAACCATTTATGGATGAGCCTGTTCACATCCCTGATAAACCTTACACAGAAGAGGAGCTGG
TTGACTTTGTGAAGGAGCACAAAAGGGCCACCTTGAGGAAACTGCGCCCAGAGGACATGTTTGAGACGTGGGAGGATGACATGGAGGGA
ATCCACATTGTGGCCTTTGCCGAAGAAGATGACCCAGATGGTTTTGAGTTCCTGGAAATCCTGAAGCAGGTTGCCAGGGACAACACTGA
TAATCCTGACCTGAGTATTGTCTGGATTGACCCTGACGACTTTCCTCTGCTGATCACTTACTGGGAGAAGACCTTCAAGATCGACCTGT
TCAGACCACAGATCGGGGTGGTGAATGTGACAGATGCTGACAGCATCTGGATGGAGATCAGCGATGATGACGACCTGCCCACAGCTGAG
GAGCTGGAGGACTGGATAGAAGATGTGCTTTCTGGGAAGATAAACACTGAAGACGACGACGACGATGACGACGACGACGATGACGACGA
CGACAATGATGATGACGATGACGACGACGATGATGACGACGACGACGACGATGATGATTAA

>CASQ2_Apus_apus_protein
MKATCWILAGFYLLFCCKAEEGLNFPTYDGKDRVIDLNEKNYKQALKKYDMLCLLFHEPVSSDKVAQRQFQMTEMVLELAAQVLEPRSI
GFGMVDSKKDAKLAKKLGLVEEGSLYVFKDERLIEFDGELATDVLVEFLLDLLEDPVEIINSKLELQAFDQIDDEIKLIGYFKGEDSEH
YKAFEEAAEHFQPYIKFFATFDKGVAKKLGLKMNEVDFYEPFMDEPVHIPDKPYTEEELVDFVKEHKRATLRKLRPEDMFETWEDDMEG
IHIVAFAEEDDPDGFEFLEILKQVARDNTDNPDLSIVWIDPDDFPLLITYWEKTFKIDLFRPQIGVVNVTDADSIWMEISDDDDLPTAE
ELEDWIEDVLSGKINTEDDDDDDDDDDDDDDNDDDDDDDDDDDDDDDDD*

6.4 *Aythya ferina* (common pochard)

From: Trinity *de novo* assembly of ERR12723487. CASQ1 from TRINITY_DN965_c1_g1_i1.

>CASQ2_Aythya_ferina_transcript_assembly
GCCGTCTGTCCGTGGGTCCGCCCCCCCGTGCTGTCCGTCCGTCTGTCCATCCACCCGTCCGTCCGTCCCCCCCCCCCCCCCGCAGCTCC
CTCCTCGTCCCTTCCTCCCCCCCCCTCCCCGACCCCCCATGCGGGTCTGGGGGCTGCTCCTGGCCCTGCTGGCCCTGGGGGTGGCGGCC
GGGGGGGGGTCCCGGGGGGGTCTCCGCTTCCCCACCCACGACGGCTCCGGGCGGCTGCTGCCGGTGCCGGCCCGGGACCCCCCCGCGGC
GCTCGGGCGCTTCCCGGTGCTGGCCCTGCTCTACCACGGCCCCCCCGCGGGGAACGAGGCTCAGCGCCGCCTGCACACGGGGGAGCTCG
TCCTGGAGCTGGCAGCCCAGGTGCTGGAGGACAGGGGCGTGGGGTTCGGCCTCGTGGACGCCCAGAAGGAAGCGGCCGTGGCCGAGAAG
CTGGGGCTGACGGAGGAGAACAGCATCTACATCTTCAAGGGGGACAAGGTGATCGAGTACGACGGGGAGCTGGCGGCCGACACGCTGGT
GGAGTTCCTGCTGGATGTGCTGGAGGAGCCGGTGGAGTTCATTGAGGGCGACCGCGAGCTCCGGGCCTTCGAGAACATCGAGGAGGACC
CCAAGGTCATCGGCTACTTCGAGGGCAAGGACTCGGAGCACTTCAAGGCTTTCGAGGAGGCGGCGGCGCAGTTCCACCCCTACGTCCCC
TTCTTCGCCACCTTTGACGCCAAGGTGGCCAAGAAGCTGACGCTGAAGCTGAACGAGGTCGACTTCTACGAGCCCTTCATGGAGGAGCC
GTTCACCCTCCCCGCCCGGCCCCCCGGCACCGAGGACATCGTGGCCTTCGTGGGGGGGCACCGGCGGACGACGCTGCGGAAGCTCAAA
CCCAAGAGCATGTACGAGACATGGGAGGATGACATCAATGGGATCCACATCGTGGCCTTCGCTGAGGAGGACGATCCGGATGGGTTCG
AGTTCCTGGAGATCCTGAAGGACCTGGCCCGGGACAACACCGACAACCCCGACCTCAGCATCATCTTGATTGACCCCGAGGACTTCCC
GCTGCTCATCCCTTACTGGGAGAAAACCTTCAACATCGACCTGTCCCGGCCCCAGATCGGGGTGGTCAATGTCACCGATGCGGACAGC
GTGTGGCTGGAGATGGACGAGGACGACCTGCCGGGAGCCGAGGAGCTGCAGGAGTGGATCCAGGACGTGCTGGAGGGAGAGATCAGCA
CCGGGGACGACGAGGACGATGATGATGAAGATGATGATGAAGATGACGATGATGATGATGATGATTACGATGATGACGAAGGCTCTTC
C

Note: Partial CASQ2, missing $3'$ *end.*

>CASQ2_Aythya_ferina_CDS_partial
ATGCGGGTCTGGGGGCTGCTCCTGGCCCTGCTGGCCCTGGGGGTGGCGGCCGGGGGGGGGTCCCGGGGGGGTCTCCGCTTCCCCACCCA
CGACGGCTCCGGGCGGCTGCTGCCGGTGCCGGCCCGGGACCCCCCCGCGGCGCTCGGGCGCTTCCCGGTGCTGGCCCTGCTCTACCACG
GCCCCCCCGCGGGGAACGAGGCTCAGCGCCGCCTGCACACGGGGGAGCTCGTCCTGGAGCTGGCAGCCCAGGTGCTGGAGGACAGGGGC
GTGGGGTTCGGCCTCGTGGACGCCCAGAAGGAAGCGGCCGTGGCCGAGAAGCTGGGGCTGACGGAGGAGAACAGCATCTACATCTTCAA
GGGGGACAAGGTGATCGAGTACGACGGGGAGCTGGCGGCCGACACGCTGGTGGAGTTCCTGCTGGATGTGCTGGAGGAGCCGGTGGAGT
TCATTGAGGGCGACCGCGAGCTCCGGGCCTTCGAGAACATCGAGGAGGACCCCAAGGTCATCGGCTACTTCGAGGGCAAGGACTCGGAG
CACTTCAAGGCTTTCGAGGAGGCGGCGGCGCAGTTCCACCCCTACGTCCCCTTCTTCGCCACCTTTGACGCCAAGGTGGCCAAGAAGCT
GACGCTGAAGCTGAACGAGGTCGACTTCTACGAGCCCTTCATGGAGGAGCCGTTCACCCTCCCCGCCCGGCCCCCCGGCACCGAGGACA
TCGTGGCCTTCGTGGGGGGGCACCGGCGGACGACGCTGCGGAAGCTCAAACCCAAGAGCATGTACGAGACATGGGAGGATGACATCAAT
GGGATCCACATCGTGGCCTTCGCTGAGGAGGACGATCCGGATGGGTTCGAGTTCCTGGAGATCCTGAAGGACCTGGCCCGGGACAACAC
CGACAACCCCGACCTCAGCATCATCTTGATTGACCCCGAGGACTTCCCGCTGCTCATCCCTTACTGGGAGAAAACCTTCAACATCGACC
TGTCCCGGCCCCAGATCGGGGTGGTCAATGTCACCGATGCGGACAGCGTGTGGCTGGAGATGGACGAGGACGACCTGCCGGGAGCCGAG

GAGCTGCAGGAGTGGATCCAGGACGTGCTGGAGGGAGAGATCAGCACCGGGGACGACGAGGACGATGATGATGAAGATGATGATGAAGA
TGACGATGATGATGATGATGATTACGATGATGACGAAGGCTCTTCC

>CASQ2_Aythya_ferina_protein_partial
MRVWGLLLALLALGVAAGGGSRGGLRFPTHDGSGRLLPVPARDPPAALGRFPVLALLYHGPPAGNEAQRRLHTGELVLELAAQVLEDRG
VGFGLVDAQKEAAVAEKLGLTEENSIYIFKGDKVIEYDGELAADTLVEFLLDVLEEPVEFIEGDRELRAFENIEEDPKVIGYFEGKDSE
HFKAFEEAAAQFHPYVPFFATFDAKVAKKLTLKLNEVDFYEPFMEEPFTLPARPPGTEDIVAFVGGHRRTTLRKLKPKSMYETWEDDIN
GIHIVAFAEEDDPDGFEFLEILKDLARDNTDNPDLSIILIDPEDFPLLIPYWEKTFNIDLSRPQIGVVNVTDADSVWLEMDEDDLPGAE
ELQEWIQDVLEGEISTGDDEDDDDEDDDEDDDDDDDDYDDDEGSS

6.5 *Buteo hemilasius* (upland buzzard)

From: Trinity *de novo* assembly of SRR25651956. CASQ1 from TRINITY_DN629_c0_g1_i3. CASQ2 from TRINITY_DN2332_c0_g1_i1.

>CASQ1_Buteo_hemilasius_transcript_assembly
CCGATCTCGCGCCCTGAGCCCCCTCCCGTGCAGTGTTGGGGGGACAGGGAATGCAGGGGGGGTCGGGGTGCTACTGTCCCCCGCTTTGC
GCCTTCAGCCCCTTTGGGGCTGGGAAGGGGCCATGTGTCTGTGTGTCCCCGGCTGTGTCCGTCTGTCCCCTCCTGGCGAGGCCGAAAGG
GGCTGTGTCTGTCCGTGTCCCCCCCCCAGGGGCCGAGTCTTTCTGTGTCCCCCCCCCCGCCTCGGGGGCCGTGTCTCTCCGTCCCCTCT
GGGGCCAGGCAGGGGCTGCGTGTCCGTCCCCTGTGCTGGGGCTGTTAGTCCCAGCCGGGCTCTGTCTGTCTGTCTTTCCATCCCCGGAC
GCAAAGGCATCATCCGTCTGTCCCCAAGGCTCTGTCTGTCTGTCCCCATCTGGCTGTTGTCCAGCCCAGGGGCTGGCTGGCTGTCCCCG
GGACTGTCTGTCCGTCTCTCCGTTCCCTGGCTGTGTGTCTATCCCTGCTCATCTGTGAGTCCATCTGTCCGTGGGTCCCATCCCCATCT
GTCCCTCTGTCCGTCCCTCTCTGCAGGTCTGTATCTGCCTGTCTGTCCGTGGGTCCCATCCACCCACCCGTCTGTCCCCATCCGTGTGT
CCGTCCCCGTCCGGGGGTCTGCCTGGCGGGCGATGGGGGTCTGGGGATGGGTGCTGGCACTGCTGGTGCTGGGGGCCGGGGCCCCGGGG
GGGGCAGGGGACGGCCTGGACTTCCCCACATACGACGGGCTGGACCGGGTGCTGCCCGTCACCCTGAAGAACTACAAGGCGATGCTGAA
GCGGTTCCCGGTGCTGGCCCTGCTCCACCACCGCCCCAGCCAGGGTGACCGGGCGGCCCAGCGCCACAGCGAGATGGAGGAGCTCATCT
TGGAGCTGGCAGCCCAGGTGCTGGAGGACAAGGGAGTGGGCTTCGGCCTCGTCGACTCTGAGAAAGACGCAGCTGTGGCCAAAAAGCTG
GGCATGACAGAGGAGGACAGCATCTACGTGTTCAAGGAGGATGAGGTGATTGAGTATGACGGGGAGCTGGCAGCAGACACGCTGGTGGA
GTTCCTGCTGGATGTGCTGGAAGACCCGGTGGAGTTCATTGAGGGTGACCATGAGCTCCAGGCCTTCGAGAACATCGAGGATGACCCCA
AACTCATCGGCTACTTCAAGAACAAGGACTCAGAGCACTTCAAGGCCTTTGAGGAGGCGGCTGAGGAATTTCACCCCTACATCCCCTTC
TTCGCCACCTTTGACAGCAAGGCTGCCAAGAAGCTCACCCTGAAGCTGAACGAGATCGACTTCTACGAGCCCTTCATGGAGGAGCCGCT
GACCATCCCTGACCGGCCCAACAGCAAGGAGGAGATCATGGCCTTTGTGGAGGAGCACAAGCGGGCCACTCTGCGGAAACTCAAACCCG
AGAGCATGTACGAGACCTGGGAGGATGACATGGACGGGATCCACATCGTGGCCTTCGCAGAGGAGGATGATCCAGATGGGTTTGAGTTC
CTGGAGATCCTGAAGGATGTGGCCCAGGACAACATGGACAACCCTGACCTCAGCATCCTCTGGATCGACCCTGAGGATTTCCCGCTGCT
CATCCCTTACTGGGAGAAAACCTTCAACATTGACCTGTCCCATCCCCAGATTGGGGTGGTCAACGTCACCGACGCCGACAGCGTGTGGC
TGGAGATGGCGGACGAGGACGACCTGCCCAGCCCGGCAGAGCTGGAGGAGTGGATCGAGGACGTGCTGGCAGGCGAGATCAACACCGAG
GACGACGACGAGGAGGATGATGACGATGATGATGATGACGACGATGACAACTAGGCCTTGTGCTGGGCTTCCGCACCCGTCCCTGTCCC
TGAGCGAGCCATTAAAGTACCCAAAGAACCAG

>CASQ1_Buteo_hemilasius_CDS
ATGGGGGTCTGGGGATGGGTGCTGGCACTGCTGGTGCTGGGGGCCGGGGCCCCGGGGGGGGCAGGGGACGGCCTGGACTTCCCCACATA
CGACGGGCTGGACCGGGTGCTGCCCGTCACCCTGAAGAACTACAAGGCGATGCTGAAGCGGTTCCCGGTGCTGGCCCTGCTCCACCACC
GCCCCAGCCAGGGTGACCGGGCGGCCCAGCGCCACAGCGAGATGGAGGAGCTCATCTTGGAGCTGGCAGCCCAGGTGCTGGAGGACAAG
GGAGTGGGCTTCGGCCTCGTCGACTCTGAGAAAGACGCAGCTGTGGCCAAAAAGCTGGGCATGACAGAGGAGGACAGCATCTACGTGTT
CAAGGAGGATGAGGTGATTGAGTATGACGGGGAGCTGGCAGCAGACACGCTGGTGGAGTTCCTGCTGGATGTGCTGGAAGACCCGGTGG
AGTTCATTGAGGGTGACCATGAGCTCCAGGCCTTCGAGAACATCGAGGATGACCCCAAACTCATCGGCTACTTCAAGAACAAGGACTCA
GAGCACTTCAAGGCCTTTGAGGAGGCGGCTGAGGAATTTCACCCCTACATCCCCTTCTTCGCCACCTTTGACAGCAAGGCTGCCAAGAA
GCTCACCCTGAAGCTGAACGAGATCGACTTCTACGAGCCCTTCATGGAGGAGCCGCTGACCATCCCTGACCGGCCCAACAGCAAGGAGG
AGATCATGGCCTTTGTGGAGGAGCACAAGCGGGCCACTCTGCGGAAACTCAAACCCGAGAGCATGTACGAGACCTGGGAGGATGACATG
GACGGGATCCACATCGTGGCCTTCGCAGAGGAGGATGATCCAGATGGGTTTGAGTTCCTGGAGATCCTGAAGGATGTGGCCCAGGACAA
CATGGACAACCCTGACCTCAGCATCCTCTGGATCGACCCTGAGGATTTCCCGCTGCTCATCCCTTACTGGGAGAAAACCTTCAACATTG
ACCTGTCCCATCCCCAGATTGGGGTGGTCAACGTCACCGACGCCGACAGCGTGTGGCTGGAGATGGCGGACGAGGACGACCTGCCCAGC
CCGGCAGAGCTGGAGGAGTGGATCGAGGACGTGCTGGCAGGCGAGATCAACACCGAGGACGACGACGAGGAGGATGATGACGATGATGA
TGATGACGACGATGACAACTAG

>CASQ1_Buteo_hemilasius_protein
MGVWGWVLALLVLGAGAPGGAGDGLDFPTYDGLDRVLPVTLKNYKAMLKRFPVLALLHHRPSQGDRAAQRHSEMEELILELAAQVLEDK
GVGFGLVDSEKDAAVAKKLGMTEEDSIYVFKEDEVIEYDGELAADTLVEFLLDVLEDPVEFIEGDHELQAFENIEDDPKLIGYFKNKDS
EHFKAFEEAAEEFHPYIPFFATFDSKAAKKLTLKLNEIDFYEPFMEEPLTIPDRPNSKEEIMAFVEEHKRATLRKLKPESMYETWEDDM
DGIHIVAFAEEDDPDGFEFLEILKDVAQDNMDNPDLSILWIDPEDFPLLIPYWEKTFNIDLSHPQIGVVNVTDADSVWLEMADEDDLPS
PAELEEWIEDVLAGEINTEDDDEEDDDDDDDDDDDN*

>CASQ2_Buteo_hemilasius_CDS
GTCCTTTTTTTTCATTGCAGGGTTTATTTTTAGCCTGAAACAACTGCTTCCTAAAAATGGAGTTCCTAATGACACGGGAGCTGGACACA
GCTATGTAAGGTATCCAGGGCTTGGCCTGACAGCTTCTCCTGTCTGTCTCTTTGTTGTAAGCTCAGGACAGCAAAGTTTGCTGCTCCCC
ACCAGCCTTGCAGCGTTTGGGCTGATCACTAGGAAGGCAGCAGAAAGAGAAACTCGGTTTGAACTTCACCTGGAGACTTTGGTCTCCCC
CTCTTCCCAAATTAAGAAGGAGAGGGACGTTTACATCCCCTCTGCTGCACTCCAAGGGCTCCCCTATGAAGGCGACTTGCTGGATCCTT
GCAGGTTTTTACCTGCTTTTCTGCTGCAAAGCAGAAGAGGGACTGAATTTCCCCACTTACGATGGGAAAGACCGAGTGATCGACCTGAA
CGAGAAGAACTACAAGCAGGCCCTGAAGAAGTACGACATGCTCTGCCTACTCTTCCACGAGCCCGTGAGCTCTGACAAGGTCTCCCAGA
AGCAGTTCCAGATGACAGAGATGGTCCTGGAGCTGGCAGCTCAGGTCCTGGAGCCCAGGAGCATCGGCTTCGGGATGGTGGACTCCAAG
AAGGACGCCAAACTTGCCAAAAAGTTAGGCTTGGTTGAAGAGGGAAGCCTCTATGTGTTTAAGGAGGAGCGGTTGATTGAATTTGATGG
GGAACTGGCCACGGATGTGTTGGTGGAATTCCTCTTGGACTTGCTAGAAGACCCTGTGGAGATCATAAACAACAAGCTGGAGCTTCAGG
CCTTTGACCAAATTGACGAGGAAATCAAACTCATCGGCTACTTCAAAGGAGAAGACTCTGAACATTACAAGGCATTTGAAGAAGCCGCC
GAACACTTCCAGCCATACATCAAGTTCTTTGCCACCTTCGACAAAGGGGTTGCCAAGAAGCTAGGGCTGAAGATGAACGAGGTGGACTT
CTATGAACCGTTTATGGATGAGCCCGTTCACATCCCCGACAAGCCTTACACAGAAGAGGAGCTGGTTGAATTTGTGAAGGAGCACAAAA
GGGCCACCTTGCGAAAACTGCGCCCAGAGGACATGTTTGAGACGTGGGAGGACGACATGGAGGGAATCCACATCGTGGCCTTCGCCGAA
GAAGACGACCCAGATGGTTTTGAGTTCCTGGAAATCCTGAAGCAGGTTGCCAGGGACAACACCGATAATCCCGACCTGAGCATTGTCTG
GATTGACCCCGACGACTTTCCTCTGCTGATCACTTACTGGGAGAAGACCTTCAAGATTGACCTGTTCAGACCACAGATTGGGGTGGTGA
ACGTCACAGACGCTGACAGCATCTGGATGGAGATCAGAGACGACGACGACCTGCCCACAGCCGAGGAGCTGGAGGACTGGATAGAGGAC
GTGCTTTCCGGGAAGATAAACACCGAAGACGATGATGACGATGACGACGACGATGATGATGACGATGATGACGACGATGACGACGACGA
TGATGACGACGATGATGACGACGATGACGATGACGATGACGATGATGACGACGACGACGACGACTAACCGTGACTCTGCGCAGTTTGAC
TTGTGGGGGCCGAGAGGCCTCCCCCTGCGGCAGGTCCCTCTATCAGAAGACCTGTGTTTGAGCGTCAGCAAGCACCGCTGCTCCTCTTT
CCCCCCCTGCCCCGCTCCCCCTGCCCCTGCCCTCGCCGGGACACCTCCCCCCCGGCCTGATTCTCGGCGGTGCTGAGCCACCGGGACTG
CCCCTGCGGGCACCCAGCACCGCTGCAAATCATGCCTCCTTAGTCAGGACAATAGGAATCTGCAGGGAATCTGCCTCGAGTGTCCCAGG
GCAGGAGAAAAGTGCCCGCCTGCTCGCTGCCAGAGGGAGACAGGGAGCCAATTTGTGTTTTCCGTGCTCGTCGGCCCAGCGCTTAACAT
CCAAAGACCAAATCTCACTTCAAGACATAGGGAAGGCCACTGTAAAATTAAGTCCTTTTATTAAACTCGATGCCCTTTGCAGCTACTCT
GCCAAGACCACTCAATCCTGCAAGTTCTGCTGCGGGAGGCTGTAGCTGCTCATTCGCACGCTAGGAGCAGCAAAAAGCTTTGGGAGGGG
AGGAAGGTTTTAGGGATGCTATTTTAAGTTTTCAGTGATTCCGTATAACGCCAGGTTATTCTCGTCTGGCAGCCCAATGCAAGACCAAA
CACCACAGTCCCTGTCTGAACCTCTTTCTTTTTGAAAGCTCAGGCCAAAGACAGGGTAAACGCAGGTGGTCTGACAGGGGCCGTCAAAG
CTTGCGAGGTCCTGAACTGCTGGAAAGGCTGATCCAGGTCTGAGCCCAAACTCTCGAGGCTGAAGGTCATGGCACTGAGCCAAACTGTT
GGTTTTCACCCCAAGGAAACACCAGAGCAGAGTCCAGGCCAGTTCCAAACATCTGCAGCCAGGTCTGGGCTGGGTCTACCTTCCCTTTC
AGGTGACCCTCAGCTGGATTGCAGGTGATGCACACGAGATAGACGGCATACTCTCCTGCCAGTGGAAGCATTCACAGGGGCCCACCCTG
AAGCTCTCCTATTCCCCCTGACTTTCCAGCTTTAGCTGACTTTTAACCCAAAGCCTACATAGCATCTTTTCCCACGACCAAGATGTTGG
GAGGGAACACGCCCATCCAACGGTATTAGATGCTGGATAACATGCGTTGGGCAACGAGCAGTGAAAGGATTGAAGCTGTTTGGCTTTTA
AGTGTTCAGTTGCACAGTTACTCCCTGCTTGAGAGACTCGGATGTCCCACAGCAGGCTGCGAGAAGCCTCTTTTGACTGCGGACTGAGG
AGGAGCTCAGGGCACCAGTTCGCAGTCGAGGAGGTCGCTCGCAAAGCGAAGAGTCAGCAACTCGGTCCTGGCGAGTGCAGCACCATCCA
TGTGCAGGTCACCGCGACAGCGTTAGGTCTCCCTGCTTTCCCATGCCGGCAGATTTCAGTTGTCTTAGCTTATTAGGTTTGACAATAGT
GCAATGAATGGAGAGTTTGGAGATAGTTTTATCTTTATAAATACATTTATAAAATATGTTCTCCAGTTGTTCAGACTTGTTAGCATAGGT
AAGGTACACAGGTCCTCTCTTGCTACAGACGAAAAACATTGTTCTGACCTTTCTTATACCGGCTTACGTCAGAGGTAGGAGTCGTTAGG
GGAAATAACAGTGCGAGGGACAGGAGAATCAGAGTTCGTTATACGAATGCAAATCAGCCTGATGAGAAACAAGGTTTACACACTATCTA
TTTCTTTTATCAGATGAACTGCAAAGCAAGATTTTATAGATTTTTTTTTTTCCTGCCTGTTGATCAGTCTCTTTGACTCACTCTCTCTC
TCTACATTAGCCGAAATAGAAATTTGAAACTCCTGGTATAAAAAAGCTGAAGTACTGATATATTCCTACGTGTGCCATAACCCCTTGGT
GGAAAGTGCCAGCACGAACAACACGCAGCTGGATATCTATATATCTCTATGTATATGAATGAATACTGTGCTTACACGGTATACAACTG
GAGGCCACGTTAGCTCCTCATTGAACATCACTCAACCCGTGCTTTTAAGGCACGGCCTATTAGAAAAACAGACAGGACAGCAAAGCCCC
CAGATCAACTATTTGCCCTTTCTTGCTTCTTTACAACGCACAAACACTGCCTGGAAGACTTGAACTAAGTGTGTTTGGGGAAACCATCC
ACTTGGTCGTGCTGGGATTACGAACAACTCAGCCTCATCCCAGAGGACCCGGTGGAGACTTCCAAAAGCCTGGAAATGCAGAGTGCTGC
TCTCAGCCTGGAGGTCCCCTCCTCTCTTTCACTCGCACCCACTCTCTCCTGGCCAGAGGGGTGCCCAGCACCCTCCGTCTGCTCTTTCA
GCTGTTTCTTCAAGTGGTTTCCTGGAAGACCCTGGCTCAGAGAGAGAGCGAGCAAGCGGGACTCGAGGTCCCGTTGATGCACATCCGTA
AGGAGCAAGAACGGGCAAATCTAGCCATTTAGTAGTCTCCTAGTCTGTGATTAAGGAACAAATACTCACCTGAGAAAAGTACTTGCTTA
TCTTTACCTTGGGGCTACTCCCAGAAGTAAACCACACACAGTTTCACCCTGCACAGGCCCTGCAGCTCCAGATCGGTCACTACAAAGTG
CCATGGATTTCTATAAACACGAAGAATAAATGAAGAGTTACTCTACTGCCTTTCCTAACCGGCAGTCTCTGGCCATCCCGACTGCCTGG
GTACTTTCCACGTACCTCCCCTCCCGCTGTGGATGCCTGTCTATTCTGATGTGCCCCTCTCTAGACGCTCCGAGGGGCACAGCTCACAC
GGGCAGACCCTGCAAACGGCACTACAGGCGTTTCACAGGCACGTACACATTTCAATGCATCGAGCAACAGAGCTCTGGGGGGGAAAAAG
TAATTAGTGTAGTGGTGCTTAGGTTTATACAGAGGCCACTTTGGCTGGGCTCATACTGGGGGTGGTGGGCTGGTGTCAGCGGTTTATGG
AAAGTTTCCTTGGAGTTGTGAGAGAACGGGCTAATCCTACGGCTGCCACTCCTTCACGGTGCCAGCGCGGTCCTTGACGGGGGCCACCC
CTTCTCTCTTCCCTCATCCCCTCATTACAGTTTTCATCGTTCTCTTTGCCCACAAACTGGAACTTTCCATCTTCAGCTTTTGGGATAAA
CACATAAAAAGTTAAGAAAACGTTGCAATGGAGGAAGGATTGCGGCAATGAAGACGAGTAGGCAGCAAAGCCTCTCCTGTTCACCCGTA
ACACCTCGTCTACCCTGAGTTCATAGTGACCAAACCGCTGAAGGAGGTAATACCCCTTGTGAAGGAGATGGCAGCTTGAACTTGAGTGT
ATTCCTCGCAGGGACAGGTAAGACAGTGGTATGGGGAGGTATCACACGCAGGACCCCAGGCACGTGCACTCCTGGGTTCCTCTGTCAGA
AATAGGCACAACCGGCATTTGAAAGGCACTGGTGGCCCTGAGGAGCAAAGTCAGCCTAATCCTGCCCACTACACACTGAAAAGCAAAAG
GTCAGTACGTTTGCATGAGAAATCAGCAAAAACCCCCTTCTTTATTTAAAAAAAAAAAAAAAAAAAATTGTGCATGTGGGCAGCACTGAG
TGTTTCTGCAGGCAAGTCTGTTTAAGACTGTCCCTCTCTTAGCAAAGAGTTCTTTTTCTCCAAAGCATTAATTAGACATTCATATTAAA

```
TGGGTCTTAATACACAGAGATAAGGAATTGTATTTTGCCCTCATCCAACTGCAAGTGCTTCAAGCTGATCAGTCACTTCAAAGTACCCC
AAACCTAGTCTTACCAGCCCCTGATGACAACAAGGATCCCCCCTCACAAGTGATATGTGGCAAGAACACGCAGTTGTACCCCACCTCCT
CCCAGTCCCCAGACTGGTTGGTGTGCTCTGTGTACTACAGGCCACCCAGTACCGCTCATGGGCAATGATCAAGGGAGAGGGAAACCCAT
CGAGAGCCACATATTGCAGCTATCATATAGAAGCACGATGGAGAAAGCTTCTGCCTTTGCAGCAGACCCAAGGACACTGATGGAGAGGC
TCTGGGAACTCGAAACGCCTCTGAGCTAACGTCAACCATTTGGGAAACAGCTCCAGAAGGAGAGGACAAGGGAGGCATTCCGGAAATCG
CAGATTACTCCATATTTTCAGGGTCAGCAGTTTTCATGATGTTTATGAGGAGTGGTGAGAATCTCCTGTGCTGCCCTTGAGAGAGGAAC
GGCCCCGTAGTGAAACCAGAGATCTCGGCTGCTTCCCATCCCAGAATGAAGCTGCGCAGCCAGATGCCAACACGCACGCACAGCTGAGG
ACACCGGCTGCTTCTCCTTGTCCGTCTGCACATCATACCTCAGAAATGATTGATTCATTACTGAGACCAAACAAAAAACATTGCCTTTT
ATTCCCAGGGAAATAATTAATTAGACAGGAACATATCACTTCCCAGCTGCCACTGACTTGCAGTGCTGTGAAAACAGGCCAGGATTACA
TCTGGATTCCTCTCTGCCCCAGCAAAGCTTTGGCTTTGGGGAAGCCCCCCACACAGCCAGTTGCCTGGACTCGGGGGGCTGAAGAGGGG
GAACGGGTGAACAGGCGACAAGCACCAAGGCCCTCCAGGGCAAAGCAAACTGCCTCCTGGCTACTTTTACACCCCAGGTACATCCACGC
CGTGCAGTGCAACACCATCCCGTAGAAACTGGAGAGACAGCGAAGCACAGCAGGAAGGATAATTAACCTCGGTAATGAGGCTGCGCCAG
CAGGACTGTGTCCTACCCGAGACCCAAGGATCTGTGTGTCAACCCACCGTGGTGCAGCATCCCCTGCCTTTTCCCTGCTCCCTCGTACC
ACCGACATCTGGGCAAAGCAGCCCTTCAATGGTCAGCAGCCCTTGGGGCCAGCAGTGGGACAAATCTCACACCTCTTTGGCAGAAGAAG
AAGATGTCTAACGATGATGCAGACCATGGGGCAGGGACCAGGGCAGAGCCAGCCCTCTTGGCCCAGCTCCCAAAACACTCCCTGCCAGG
TTTAAAAGGTGCTAAACTATTTAAAGGGGGAGGAAAAAAAAAAAAAAAGAAGGCTACAGAGCTTCCTTGCAGCTTGAAGACCCGTCTCTA
GAGTCAGATTACAAGCAGCTTTTGGG
```

```
>CASQ2_Buteo_hemilasius_CDS
ATGAAGGCGACTTGCTGGATCCTTGCAGGTTTTTACCTGCTTTTCTGCTGCAAAGCAGAAGAGGGACTGAATTTCCCCACTTACGATGG
GAAAGACCGAGTGATCGACCTGAACGAGAAGAACTACAAGCAGGCCCTGAAGAAGTACGACATGCTCTGCCTACTCTTCCACGAGCCCG
TGAGCTCTGACAAGGTCTCCCAGAAGCAGTTCCAGATGACAGAGATGGTCCTGGAGCTGGCAGCTCAGGTCCTGGAGCCCAGGAGCATC
GGCTTCGGGATGGTGGACTCCAAGAAGGACGCCAAACTTGCCAAAAAGTTAGGCTTGGTTGAAGAGGGAAGCCTCTATGTGTTTAAGGA
GGAGCGGTTGATTGAATTTGATGGGGAACTGGCCACGGATGTGTTGGTGGAATTCCTCTTGGACTTGCTAGAAGACCCTGTGGAGATCA
TAAACAACAAGCTGGAGCTTCAGGCCTTTGACCAAATTGACGAGGAAATCAAACTCATCGGCTACTTCAAAGGAGAAGACTCTGAACAT
TACAAGGCATTTGAAGAAGCCGCCGAACACTTCCAGCCATACATCAAGTTCTTTGCCACCTTCGACAAAGGGGTTGCCAAGAAGCTAGG
GCTGAAGATGAACGAGGTGGACTTCTATGAACCGTTTATGGATGAGCCCGTTCACATCCCCGACAAGCCTTACACAGAAGAGGAGCTGG
TTGAATTTGTGAAGGAGCACAAAAGGGCCACCTTGCGAAAACTGCGCCCAGAGGACATGTTTGAGACGTGGGAGGACGACATGGAGGGA
ATCCACATCGTGGCCTTCGCCGAAGAAGACGACCCAGATGGTTTTGAGTTCCTGGAAATCCTGAAGCAGGTTGCCAGGGACAACACCGA
TAATCCCGACCTGAGCATTGTCTGGATTGACCCCGACGACTTTCCTCTGCTGATCACTTACTGGGAGAAGACCTTCAAGATTGACCTGT
TCAGACCACAGATTGGGGTGGTGAACGTCACAGACGCTGACAGCATCTGGATGGAGATCAGAGACGACGACGACCTGCCCACAGCCGAG
GAGCTGGAGGACTGGATAGAGGACGTGCTTTCCGGGAAGATAAACACCGAAGACGATGATGACGATGACGACGACGATGATGATGACGA
TGATGACGACGATGACGACGACGATGATGACGACGATGATGACGACGATGACGATGACGATGACGATGATGACGACGACGACGACGACT
AA
```

```
>CASQ2_Buteo_hemilasius_protein
MKATCWILAGFYLLFCCKAEEGLNFPTYDGKDRVIDLNEKNYKQALKKYDMLCLLFHEPVSSDKVSQKQFQMTEMVLELAAQVLEPRSI
GFGMVDSKKDAKLAKKLGLVEEGSLYVFKEERLIEFDGELATDVLVEFLLDLLEDPVEIINNKLELQAFDQIDEEIKLIGYFKGEDSEH
YKAFEEAAEHFQPYIKFFATFDKGVAKKLGLKMNEVDFYEPFMDEPVHIPDKPYTEEELVEFVKEHKRATLRKLRPEDMFETWEDDMEG
IHIVAFAEEDDPDGFEFLEILKQVARDNTDNPDLSIVWIDPDDFPLLITYWEKTFKIDLFRPQIGVVNVTDADSIWMEIRDDDDLPTAE
ELEDWIEDVLSGKINTEDDDDDDDDDDDDDDDDDDDDDDDDDDDDDDDDDDDDDDDDDD*
```

6.6 *Calypte anna* (Anna's hummingbird)

From: Trinity *de novo* assembly of SRR21777129. CASQ1 from `TRINITY_DN355_c0_g1_i24`. CASQ2 from `TRINITY_DN61_c0_g1_i2`.

```
>CASQ1_Calypte_anna_transcript_assembly
GGGGCTGTGTCTGTCTGTCTGTCCCTCTGTCCTTCCCCCCCCCCTCCCTCCACCTTTTGGGGGGGGGGGGCTGTGTCCGTCCGTCCCCTG
CGGGTTCTGTCCGTCCGTCTGTCTGTCCCTGGGCACTGCCACGCACCGGCTGCCTGCCCCCACGACCCTCCCACGACCACCCCCCCCCC
CGGCTGTGTCCGTCTGTCTGTCCCCTGGGCTTGGTGGCCCCCCCGTGGCCCTCTGTGTCCCTCTGTCCGTCCGTCGGCGATGGGTTCC
TGGGGGTGGGCTCTGGCTCTGCTGGCGTTGTGGGGGGGGGTCCCAGGGGTCTCTGGGGCGGTGCCGGGGGGTGCGGGGGGGGCCCTGTC
TGTCCCCACCCCTGACGGAAGGTCACGGGTGGTCTCGGTCACCCTGAGGAACTTCCAAGGGCTGCGGCAAAGGTTCCCGGTGCTGGCTC
TGCTGCACAGACCCCCCCGGGACCCCCACAACCCTAAGATGGTCCTGGAGCTGGCAGCCCAGGTGCTGGAGGAGAAGGGGGTGGGCTTC
GGCCTCCTCGACTCAGAGAAGGACGCAGCCATCACCAAAAAGCTGGGCCTGACAGAGGAGGGCAGCATCTACGTGTTCAAGGAGGATGA
AGTGATCGAGTACGACGGGGAGATGGCAGCCAACACCCTGGTGGAATTCCTGCTGGATGTGCTCGAGGACCCAGTGGAGTTCATTGAGG
GTGACCACGAGCTCCAGGCCTTCGAGAACATCGAGGAGGACCCCAAACTCATTGGGTACTTCAAGGACAAGGACTCAGAGAATTTCAAA
GCCTTTAAGGAGGCAGCTGAGCAGCTCCACCCCTACGTCCCTTTCTTTGCCACCTTTGACACCAAGGTGGCCAAAAAGCTGAGGCTGAG
GTTGAACAAGATCAACTTCTACCAACCCTTCCTGGAGGAGCCACTGCCCATCCCCGGCAGCCCCCACACCCGGGGGGAGATCCTGGACT
TTGTGGAGCAGAACAGGAGGCCCCCTCTGCGGAAACTCAAACCCGAGAGCATGTACGAGACCTGGGAGGATGACATGGATGGGATCCAC
ATCGTGGCCTTTGCTGAGGCAGATGATCCAGATGGGTTCGAGTTCCTGGAGATCCTGAAGGACGTGGCCCGGGATAACGTGGACAACCC
TGACCTCAGCATCCTCTGGATCGACCCCAAGGATTTCCCGCTGCTCATCCCTTACTGGGAGAAAACCTTCGGCATCGACCTCTCCGTGC
```

CCCAGCTTGGGGTGATCAATGTCACCGACGCTGACAGCGTGTGGCTTGAGATGGTGGATGAGGATGATCTGCCCGGGCCGGAGGAGGTG
GAGCAGTGGCTGAAGGATGTGCTGGAGGATGAGGAGGATGATGATGGCAACGATGACAATGATGATGAAGGTGATGATGACAACGGTGA
TAATGGTGCTGATGACAGCGATGATGATGATGATGATGATGATGATGATGATGATGATGATGATGATGAAAACAAGGAGGATGACAGTG
ATGAGGAGGACAACGACAGTGATGACAACACCTAAACCTTGGGTTCTGCTCCCTCTCCAAGACATTAAAGTCCCCAAAGATCCCGACTG
TGTCACCAAGGTGGGGACACGGGGGGGCTGGAGGTACTTTGGGAACAGGAGAGACCCCCCCTCACCCCACGAGACCCCCGGGGTGCGGG
AAACCCCCGTGGGCTTTGGACACTCGTGGTGGCTCCTCCTTGGGGTGACAACAGGCGGATTGATGTCCCCAAAAACATGCCAGGGGTGG
GGGGCGTCAGGGGTGGGGGCTACTTCGGCCCCGCCTCTCTAAGCCCCGCCCATTCGGCCCCTCCCCGATAAGCCCCGCCCCCTCGGCCC
CTCCCCGCTGCTCAGAGCGGCGGCACCGAGACACCGAGCGGTCGCACCGGCACCGGCACCGCCACCGGCACCGCCACAGCCGGCCGCCA
TGGCCGAGTACCGCACTCTGCTGGAGGAGCTGTCCCGGAACATCACGGCCGAGGACCTGGAGACGCTGAAATCCGCCTGCAAGGAGGAC
ATCCCCAGCGGGGAGAGCGAGGCCATCGCCACCGGGCAGCACTGGTTCCAGTTCCTGGAGAAACACAGCAAACTGGACCGGGACAACCT
CTCCTACATCGAGCACATCTTCGAGATCTCCCGCCGCCCCGACCTCCTGACCATGGTGGTTCAGTACCGCACCCACGTCCTCAAGATCT
CCGAGGAGGACGAGGTGGACACCAAACTCACGCGTATCCCCAGCGCCAAGAAGTACAAGGACATCATCCGGCAGCCCTCGGAAGAGGAG
ATCATCAAACTGGCCCCCCCACCCAAGAAAGCCTGAGGGGGCGTGAGGAGGAGGAGGAGGAGGAGGAAGGCTGTGCCCCCCCCCCT
GTGTGTGTCCCCCCCTTTC

>CASQ1_Calypte_anna_CDS
ATGGGTTCCTGGGGGTGGGCTCTGGCTCTGCTGGCGTTGTGGGGGGGGGTCCCAGGGGTCTCTGGGGCGGTGCCGGGGGGTGCGGGGGG
GGCCCTGTCTGTCCCCACCCCTGACGGAAGGTCACGGGTGGTCTCGGTCACCCTGAGGAACTTCCAAGGGCTGCGGCAAAGGTTCCCGG
TGCTGGCTCTGCTGCACAGACCCCCCCGGGACCCCCACAACCCTAAGATGGTCCTGGAGCTGGCAGCCCAGGTGCTGGAGGAGAAGGGG
GTGGGCTTCGGCCTCCTCGACTCAGAGAAGGACGCAGCCATCACCAAAAAGCTGGGCCTGACAGAGGAGGGCAGCATCTACGTGTTCAA
GGAGGATGAAGTGATCGAGTACGACGGGGAGATGGCAGCCAACACCCTGGTGGAATTCCTGCTGGATGTGCTCGAGGACCCAGTGGAGT
TCATTGAGGGTGACCACGAGCTCCAGGCCTTCGAGAACATCGAGGAGGACCCCAAACTCATTGGGTACTTCAAGGACAAGGACTCAGAG
AATTTCAAAGCCTTTAAGGAGGCAGCTGAGCAGCTCCACCCCTACGTCCCTTTCTTTGCCACCTTTGACACCAAGGTGGCCAAAAAGCT
GAGGCTGAGGTTGAACAAGATCAACTTCTACCAACCCTTCCTGGAGGAGCCACTGCCCATCCCCGGCAGCCCCCACACCCGGGGGGAGA
TCCTGGACTTTGTGGAGCAGAACAGGAGGCCCCCTCTGCGGAAACTCAAACCCGAGAGCATGTACGAGACCTGGGAGGATGACATGGAT
GGGATCCACATCGTGGCCTTTGCTGAGGCAGATGATCCAGATGGGTTCGAGTTCCTGGAGATCCTGAAGGACGTGGCCCGGGATAACGT
GGACAACCCTGACCTCAGCATCCTCTGGATCGACCCCAAGGATTTCCCGCTGCTCATCCCTTACTGGGAGAAAACCTTCGGCATCGACC
TCTCCGTGCCCCAGCTTGGGGTGATCAATGTCACCGACGCTGACAGCGTGTGGCTTGAGATGGTGGATGAGGATGATCTGCCCGGGCCG
GAGGAGGTGGAGCAGTGGCTGAAGGATGTGCTGGAGGATGAGGAGGATGATGATGGCAACGATGACAATGATGATGAAGGTGATGATGA
CAACGGTGATAATGGTGCTGATGACAGCGATGATGATGATGATGATGATGATGATGATGATGATGATGATGATGATGAAAACAAGGAGG
ATGACAGTGATGAGGAGGACAACGACAGTGATGACAACACCTAA

>CASQ1_Calypte_anna_protein
MGSWGWALALLALWGGVPGVSGAVPGGAGGALSVPTPDGRSRVVSVTLRNFQGLRQRFPVLALLHRPPRDPHNPKMVLELAAQVLEEKG
VGFGLLDSEKDAAITKKLGLTEEGSIYVFKEDEVIEYDGEMAANTLVEFLLDVLEDPVEFIEGDHELQAFENIEEDPKLIGYFKDKDSE
NFKAFKEAAEQLHPYVPFFATFDTKVAKKLRLRLNKINFYQPFLEEPLPIPGSPHTRGEILDFVEQNRRPPLRKLKPESMYETWEDDMD
GIHIVAFAEADDPDGFEFLEILKDVARDNVDNPDLSILWIDPKDFPLLIPYWEKTFGIDLSVPQLGVINVTDADSVWLEMVDEDDLPGP
EEVEQWLKDVLEDEEDDDGNDDNDDEGDDDNGDNGADDSDDDDDDDDDDDDDDDDENKEDDSDEEDNDSDDNT*

>CASQ2_Calypte_anna_transcript_assembly
GCCTGACAGCTTCTCCTGTCTGTCTCTTTGTTGTGAGCTCAGGACAGCAGAGTTTGCTGCTCCTCAGCAGCCTTGCAGAGTTTGGGCTG
ATCACTAGGAAGGCAGCAGAAAGAGAAACTCGGTTCGAAGTTCACCTGGAGCCTTTGGTCTCCTCCTGTTCCCAGTCTAAGAAGGAGTG
GGACATTCATCTGTCCTCCGTCGCACTCCAGGGGCTCCCCCATGAAGGCAACTTGCTGGATCCTGGCAGGTTTTTACCTGCTTTTCTGC
TGCAGGGCAGAAGAGGGACTGAACTTCCCCACTTACGATGGGAGAGACAGGGTGATCGACCTGAACGAGAAGAACTACAAACAGGCCCT
AAAGAAGTATGAAATGCTCTGCCTGCTCTTCCATGAGCCCGTGAGCTCTGACAAGGTTTCCCAGAAGCAGTTCCAGATGACAGAGATGG
TCCTGGAGCTGGCAGCTCAGGTCCTGGAGCCCAGGAGCATTGGCTTTGGGATGGTGGACTCCAAGAAGGATGCCAAACTTGCTAAAAAG
CTGGGCTTGGTTGAAGAGGGAAGTCTCTATGTCTTTAAGGATGAGCGCTTGATTGAATTTGATGGGGAACTGGCCACAGATGTCTTGGT
GGAATTCCTTTTGGATCTGCTTGAAGACCCTGTGGAGGTCATCAACAGCAAGCTGGAGCTCCAGGCCTTTGATCAAATCAATGATGAAA
TCAAACTCATCGGCTACTTCAAAGGAGAAGATTCTGAACATTACAAGGCATTTGAAGAAGCTGCTGAACACTTCCAGCCATATGTCAAG
TTCTTTGCCACCTTTGACAAAGGGGTTGCCAAGAAGCTAGGTCTGAAGATGAATGAGGTGGACTTCTATGAACCATTTATGGATGAGCC
TGTTCACATCCCTGATAAACCTTACTCAGAGGAGGAACTAGTTGAATTTGTGACGGAGCACAAAAGGGCCACCTTGAGGAAACTGCGCC
CAGAGGACATGTTTGAGACATGGGAGGACGACATGGAGGGAATCCACATTGTGGCATTCGCTGAAGAAGACGACCCAGATGGGTTTGAG
TTCCTGGAAGTCCTGAAGCAGGTTGCCAGGGACAACACTGATAATCCTGACCTGAGCATTGTCTGGATTGACCCTGATGACTTTCCTCT
GCTGATCACTTACTGGGAGAAGACCTTTAATATCGACCTGTACAGACCACAGATTGGGGTGGTGAACGTCACAGATGCTGACAGCATCT
GGATGGAGATCAGTGATGACGATGACCTGCCCACGGCCGAGGAGCTGGAGGACTGGATAGAGGATGTGCTTTCTGGGAAGATAAACACT
GAAGATGATGACGATGACGACGACGATGACGACGACGACGATGACGATAATGATGACGACGACGACGATGACGACGATGACGACGATGA
CGACGACGACGATTAACTGTGACTGTGCAGTTTGACTTGTGGGAGGCTGAGAGCCCCCCCCTTCCCCCGCGGCAGGTCCCTCCATCGGA
AGACCTGTGTTTGAGCGTCAGCAAGCACCGCTGCTCCTCTTCCCCTCTCCTCCCCCCCTCCCCCGTTTCTCTCCCCCCGCCCCTGCCCT
CGCCGGGACCCCCCAGCCTGATTCTCAGTGGTGCTGAGCCCCAGCGGCAGCACCGCTGCCAATCATGCCTCCTTAGTAAGGACTATAGG
AATCTGCAGGGAATCCGCGCCCAGTGCCCCAGGGCAGGAGAAAAGTGCCTGCCCGCTCGCTGCCAGAGGGACACAGGGAGGCAATTTGT
ATTTCCCGTGCTCATCGGCCCAGCACTTAACATCCAAAGACCAAATCTCACCTCAAGACATAGGGAAGGCCACTGTAAAACTTCAGTCC

TTTTATTAAACACGATGCCCTTTGCAGCTACTCTGCCGAGACCACTCCATCCTGCGAGTTGTGCTGTGGGAAGTCTGTGACTACTCATT
AGCATGCTAGGAGCAGCAGAAAGCTTGGGGAGGGGAGGAAGGTTTTAAGGATGCTATTTAAGTTTTCAGTGATTCTGTATAACAACAGA
ATATTCTTGTCTGGCAGTGCAATGCAAAACCAAACACCCTGAACCTCTTTCTTCTTGAAAGCTCAGGCCAAAGGTAGGTTAAATGCAAG
TGCTGTGATATGGGATGTCAAAGCTTGGGAGTACCTGAACTGATGGAAAGGCTGATCCAGGTCTGAGCACAAATTATTGAGGCTGAGGA
TCACAGCATTGAGCCAGACAATTTGTCTCAGCCCCAAAGAAACACCAGAGCAGACCATAGGCCAGTTCCAAACATCTGCAGCCAAGTCT
GGGCTGGAACAGAATTGAAGGTGGTGCATGTGAGATAAACAGCATACTCCTTCCTGCCAGTGGAAATTTTCAGTCTACCTTGAGGCACT
CCAATTTCCCATAACCTTCCAGATTTAGCTGACTTTTAATCCAAAGCCTACCCAGCATCTTCTCCCATGACCAAGACATTGGGAGAGAG
AACAGCCATCCAACCATAGTAGATGCTGGAAAATGAGAATTGGGCAATGAGCAGTGAAAGGATTCAAGCTGTTTGTCTTTTAAATGTTC
AGTTGCACAGTTACTCCCTCCTTAGAGACTGGGATGTCCCATGGTAGCCCACAAGAAGCCTCTTTTGACTGCAGACTGAGGAGGAGCTC
AGCACACCAGTTGGCAGTCAAGAGGGTCACTCACAAATCCAAGACTCAGCAGCAATTCAGTCCTGGTGAGCGCAGCACCATCCGTGTGC
AGGTCACTGTGACAGTGTTAGGTGTCCCTGCTTCCCCATGCTGGCAGATTTTAATTGTCTTAGCTTCTTAGGTTTGACAATAGTGCAAT
GAATGGAGAGTTTGGAAATGTTTTTATCCTTATAAATTTATAAATATGTTCTCCAGTCGTTCAGACTTGTTAGCATAGGCAGGGTACAC
ATATCCTTTCTTGCTACAGACAAAAAAAACATTGTTCTGACCTTTCTTGTACTGGCTTACGTCAGAGGTAAGTGATGTTAGGGGAAATA
ACCGTGCAAGGGACAGTAGAATTTGAGGTTGTTACACAAATGTGAATCAGTCTGATGAGAAACAAGGTTTACACATGATATATTTCCTT
TATCAGATGAACTGCAAAGCCAGATTTTATAGACTTCTTTTTTTTTTCCTGCCTGTTGATCAGTCTCTTTGACTCTCTATCTATGTTAG
CTGAAATAGAAATTTGAAACTACTGGTACAAAAAAGCTGAAGTACTGGTATATTCCTACATGTGCCATATCCTCTTGGTGGTAAGTGCC
AACATGAACAATACACAGCTGGATATCTCTGTATCTTTATGTATATGAATGGATACTGTGCATCTATGGTATATGCCGAGGCCATGCTA
GCTCTTCATTGAACATCAAACACTCAGCCCTTGCTTCTCAGGCACAGCTTAATAGAATAGACAGCAAAGCCTCCCAGTCAACTATTGTC
TTATAGATACTTGCCCTTTCTTGCTTCTTTATGATGGATAAGCACTGCCTGGAAAATTTGAACCAAGTGAGTTTGGGGAGACCATCCAC
CTGGTCACATGAGGGTTGTGAACAACTCACCCCCATCTCAGAGGATCTGCTGGAGACTTTCAGAAGGCACAAAATACAGAGTGCTTTCA
GCCCGGAGGTCCTGTCCTTCACACACCCCTCTCTCCTGGCTGGAAAGGTGCCCTGCTTCTGCTGTTTCCTTTCAGCCTTTTCCTTGAGA
GGTTTGCTGGAGGCTTCTGGCTCAGAGAGAGAGCAAGCAGGACTCAGGGTCTCATTGATGCACATGGCTAAGGAGTGAGATCAAGTAAA
AAGAGCAGTTTAGTAGTCTCTGTGATTAAGGGACAAATGCTGCTTATGTCACTAACCTCAGCAAAGTACTTCTTTATCTTTGCCTTGGG
GCTATTCCCCAGAGTTAACCACACTCAGTGTCACCATCACCTTGCCTAAGGTAACAGCCTGCACGGGCCTTGCAGCTCCAGGTCAGTCA
CTGGAAAGTGCCATGAATTTCTATAAATGTGAAGAATAAATGAAAAGTTGCTCTACTGACTTTCCTAGCTGGCACTGTCTCTGGACATC
CCATTTGGCTGGATACTCTCCATGTCCCTCCCCTCTTGCTGTGGATGCCCATCTGCTCTGAAGTGCCCCTCTCTGGATGCTCTGGGAGG
CACAGCCTGTGCAGGCAGACCATGCCAATGGCACTGCAGACACCTTCAAGTGCACAGGCAGGTGCACAAGTCACTGCATCAAGCAACAG
AGGTTGGGAGGGATGAAAAATAATG

>CASQ2_Calypte_anna_CDS
ATGAAGGCAACTTGCTGGATCCTGGCAGGTTTTTACCTGCTTTTCTGCTGCAGGGCAGAAGAGGGACTGAACTTCCCCACTTACGATGG
GAGAGACAGGGTGATCGACCTGAACGAGAAGAACTACAAACAGGCCCTAAAGAAGTATGAAATGCTCTGCCTGCTCTTCCATGAGCCCG
TGAGCTCTGACAAGGTTTCCCAGAAGCAGTTCCAGATGACAGAGATGGTCCTGGAGCTGGCAGCTCAGGTCCTGGAGCCCAGGAGCATT
GGCTTTGGGATGGTGGACTCCAAGAAGGATGCCAAACTTGCTAAAAAGCTGGGCTTGGTTGAAGAGGGAAGTCTCTATGTCTTTAAGGA
TGAGCGCTTGATTGAATTTGATGGGGAACTGGCCACAGATGTCTTGGTGGAATTCCTTTTGGATCTGCTTGAAGACCCTGTGGAGGTCA
TCAACAGCAAGCTGGAGCTCCAGGCCTTTGATCAAATCAATGATGAAATCAAACTCATCGGCTACTTCAAAGGAGAAGATTCTGAACAT
TACAAGGCATTTGAAGAAGCTGCTGAACACTTCCAGCCATATGTCAAGTTCTTTGCCACCTTTGACAAAGGGGTTGCCAAGAAGCTAGG
TCTGAAGATGAATGAGGTGGACTTCTATGAACCATTTATGGATGAGCCTGTTCACATCCCTGATAAACCTTACTCAGAGGAGGAACTAG
TTGAATTTGTGACGGAGCACAAAAGGGCCACCTTGAGGAAACTGCGCCCAGAGGACATGTTTGAGACATGGGAGGACGACATGGAGGGA
ATCCACATTGTGGCATTCGCTGAAGAAGACGACCCAGATGGGTTTGAGTTCCTGGAAGTCCTGAAGCAGGTTGCCAGGGACAACACTGA
TAATCCTGACCTGAGCATTGTCTGGATTGACCCTGATGACTTTCCTCTGCTGATCACTTACTGGGAGAAGACCTTTAATATCGACCTGT
ACAGACCACAGATTGGGGTGGTGAACGTCACAGATGCTGACAGCATCTGGATGGAGATCAGTGATGACGATGACCTGCCCACGGCCGAG
GAGCTGGAGGACTGGATAGAGGATGTGCTTTCTGGGAAGATAAACACTGAAGATGATGACGATGACGACGACGATGACGACGACGACGA
TGACGATAATGATGACGACGACGACGATGACGACGATGACGACGATGACGACGACGACGATTAA

>CASQ2_Calypte_anna_protein
MKATCWILAGFYLLFCCRAEEGLNFPTYDGRDRVIDLNEKNYKQALKKYEMLCLLFHEPVSSDKVSQKQFQMTEMVLELAAQVLEPRSI
GFGMVDSKKDAKLAKKLGLVEEGSLYVFKDERLIEFDGELATDVLVEFLLDLLEDPVEVINSKLELQAFDQINDEIKLIGYFKGEDSEH
YKAFEEAAEHFQPYVKFFATFDKGVAKKLGLKMNEVDFYEPFMDEPVHIPDKPYSEEELVEFVTEHKRATLRKLRPEDMFETWEDDMEG
IHIVAFAEEDDPDGFEFLEVLKQVARDNTDNPDLSIVWIDPDDFPLLITYWEKTFNIDLYRPQIGVVNVTDADSIWMEISDDDDLPTAE
ELEDWIEDVLSGKINTEDDDDDDDDDDDDDDDNDDDDDDDDDDDDDDDD*

6.7 *Cathartes melambrotus* (greater yellow-headed vulture)

From: Trinity *de novo* assembly of SRR10852844. No CASQ1 was found. CASQ2 from TRINITY_DN1839_c1_g1_i3.

>CASQ2_Cathartes_melambrotus_transcript_assembly
ATGGAGTTCCTAATGACACGGGAGCTGGACACAGCTATGTAAGGTATCCAGGGCTTGGCCTGACAGCTTCTCCTGTCTGTCTCTTTGTT
GTGAGCCCAGGACAGCAAAGTTTGCTGCTCCCCACCAGCCTTGCAGCGTTCGGTCTGATCACTAGGAAGGCAGCAGAAAGAGAAACTCG
GTTCGAACTTCACCTGGAGCCTTTGGTCTCCCCCTCTTCCCAAATTAAGCAGGAGAGGGACATTTACCTGCCCTCTGCTGCACTCCAAG
GGCTCCCTTATGAAGGCGACTTGCTGGATCCTGGCAGGTTTTTACCTGCTTTTCTGCTGCAAGGCAGAAGAGGGACTGAATTTCCCCAC
TTACGATGGGAAAGACCGAGTGATCGACCTGAATGAGAAGAACTACAAGCAGGCCTTGAAGAAGTACGACATGCTCTGCCTGCTCTTCC

ACGAGCCTGTGAGCTCTGACAAGGTCTCCCAGAAACAGTTCCAGATGACAGAGATGGTCCTGGAGCTGGCGGCTCAGGTCCTGGAGCCC
AGAAGCATCGGCTTCGGGATGGTGGACTCCAAGAAGGACGCCAAACTTGCCAAAAAATTAGGCTTGGTTGAAGAAGGAAGCCTCTATGT
CTTTAAGGAGGACCGGTTGATTGAATTTGATGGGGAACTGGCCACGGATGTCTTGGTGGAATTCCTCTTGGATCTGCTAGAAGACCCTG
TGGAGATCATAAACAACAAGCTGGAACTTCAGGCCTTTGACCAAATCGACGAGGAAATCAAACTCATCGGCTACTTCAAAGGAGAAGAC
TCTGAACATTACAAGGCATTTGAAGAAGCTGCTGAACACTTCCAGCCATACATCAAGTTCTTTGCCACCTTCGACAAAGGGGTTGCCAA
GAAGCTAGGTCTGAAGATGAACGAGGTGGACTTCTATGAACCGTTCATGGATGAGCCTGTTCACATCCCCGATAAGCCTTACACAGAAG
AGGAGCTGGTTGAATTTGTGAAGGAGCACAAAAGGGCCACCTTGCGGAAGCTGCGCCCAGAGGACATGTTTGAGACGTGGGAGGATGAC
ATGGAGGGAATCCACATCGTGGCCTTCGCCGAAGAAGATGACCCAGATGGTTTTGAGTTCCTGGAAATCCTGAAGCAGGTTGCCAGGGA
CAACACCGATAATCCCGACCTAAGCATTGTCTGGATCGACCCCGACGACTTTCCTCTGCTGATCACTTACTGGGAGAAGACCTTCAAGA
TTGACCTGTTCAGACCACAGATCGGGGTGGTGAACGTCACAGATGCTGACAGCGTCTGGATGGAGATCAGAGATGACGATGACCTGCCC
ACAGCCGAGGAGCTGGAGGACTGGATAGAGGACGTGCTTTCTGGGAAGATAAATACCGAAGATGATGACGACGATGATGACGATGATGA
CGACGATGACGACGATGACGACGATGATGACGACGATGATGACGACGATGACGACGATGACGACTAACTGTGACTCTGTGCAGTTTGAC
TTGTGGGGGCCGAGAGGCCTCCCCCTGCGCAGGTCCCTCCATCGGAAGACCTGTGTTTAAGCGTCAGCAAGCACCGCTGCTCCTTTTTC
CCCCCTGCCCCGCTCCCCCTGCCCCTGCCCTTGCCGGGACCCCTCGGCCTGATTCTCAGTGGTGCTGAGCCACCGGGACTGCCCCTGCG
GCGGGCGGCGCCTGCAGGCACCCAGCACCGCTGCAAATCATGCCTCCTTAGTAAGGACAATAGGAATCTGCAGGGAATCTGCCCCGAGT
GTCCCAGGGCAGGAGAAAAGTGCCTGCCTGCTCGCTGCCAGAGGGACACAGGGAGCCAGTTTGTATATTTTCGATGCTCATTGGCCCAG
CACTTAACATCCAAAGACCAAATCTCACTTCAAGACATAGGGAAGGCCACTGTAAAGTTTAGTTCTTTTATTAAACTCAATGCCCTTTG
CAGCTACTCTGCCAAGATCACTCAATCCTGCAAGTTCTGCTGCGAGAGTCTAGCTACTCATGAACATGCTAGGAGCAGCAAAAAGCTTT
GGGAGGGGAGAAAGGTTTTAGGGATGCTATTTAAGTTTTCAGCGATTCCATATAATGACAGGATATTCTTGTCTGGCAGCGCAATGCAA
AATCAAACACCACGGTCCCTGTCTGAACCTCTTTCTTCTTGAAAGCTCAGGCCAAAGATAGGGTAAGTGCAGGTGGTCTGATATGGGAC
ATCAAAGGTTGCAAGTTCACGAACTGCTGGAAAGGCTGATCCAGGTCTGAGCCCAAAATCTTGAGGCTGAAGATCATGGCACTGAGCCA
AACCTTTGGTCTTAACCCCAAAGAAACACCAGAAGCAGACTCCAGGCCAGTTCCAAACATCTGCAGCCAGGTCTGGGCTGGGTCTACCT
TCCCTTTCAGGTGAACCTCAGCTGGATTGAAGGTGGTGCACAGGAGACAGACAGCATACTCCTGCCAGTGGAAGCATTCATAGGGGCCC
ATCCTGAAGCCCTCCGGTTCCCCATGACCTTCGAGCTTTAGCTGACTTCTAACCCAAAGCCTACGTAGCATCTTTTCCCATGACCAAGA
CGTCGCAAGGGAACACATCCGTCCAACCATATTAGATGCTGGATAACATGTGTTGGGCAATGAGCAGTGAAAGGATTCAAGCTGTGTGG
CTTTTAAGTATTCAGTTACACAGTTACTACCTGCTTAGAGACTCAGATGTCTCTCATGGTAGTCCGTGAGAAGCCTCTTTTGACTGCAG
ACTGAGAAGGAGCTCAGAGCGCCGGTTTGCAGTCAAGGGGGTCACTCACAAAGCAAAGGGTCAGCCACTCAGTCCTGGCGAGTGCAGCA
CCATCCATGTGCAGGTCACTGCGACAGTGTTAGGTCTCCCTGCTTTCCCATGCCGGCAGATTTCAATTGTCTTAGCTTGTTAGGTTTGA
CGATAGTGCAATGAATGGAGAGCTTGGAGATAGTTTTATCTTTATAAATACATTTATAAATATGTTCTCCAGTCGTTCAGACTTGTTAG
CATAGGTAGGGTACACACGTCCTCTCTTGCTACAGACGAAAAACATTGTTCTGACCTTTCTTATACTGGCTTACGTCAATGGTAAGTGA
TGTTAGGAGAAATAACAGTGCAAGGGACAGGAGAATCAGAGTTCATTATACGAATGCAAATCAGCCCGATGAGAAACAAGGTTTACACA
CTATATATTTCCTTTTTCAGATGAACTGCAAAGCAAGATTTTATAGATTATTTTTTTTCCTGCCTGTTGATCAGTCTCTTTGACTCTCT
CTCTCTCTACATTAGCTGAAATAGAAATTTGAAACTACTGGTATAAAAAAGCTGAAGTACTGATATATTCCTACGTGTGCCATAGCCTC
TTGGTGGAAAGTGCCAGCATGAACAACACGCAGCTGGATACCTATATATCTCTATGTATACGAATGAACACTGTGCTTATATAGTATAT
GACTGTAGGCCACCTTAGCTCCTCATTGAACATCAAACACTCAACCCGTGCTTTTAAGGCACAGCCTATTAGAAGAATAGACAGGACAG
CAAAGCCCCCAGGTCAACAATTTGCCCTTTCTTACTTCTTTACGATGCATAAGCACTGCCTGGAAGACTTGAACCAAGTGTGTTTGGGG
AGACCATCCACCTGGTTGCACTGGGGTTATGAACAACTCAGCCCCATCCCAGAGGACCTGGTGGAGACTTTCAAAAGTCTTGAAATGCA
GAGTGCTCTCAGCCTGGAGGTCCCCTCCTCTCTTTCACTCACACACCCCTCTCTCCTGGCCGGAGGGGTGCCCAGCACCCTCCTTCTGC
TCTTTCAGCTGTATCCTTGAGAGATTTGCTGGAGGACCCCGGCTCAGAGAGAGAGGGAGCAAGTGGGACTCAAGGTCTTGTTGATGCAC
ATCGGTAAGGAGCAAGAACAGGCAAAACTAGCCATTTAGTAGTCTCCTAGTCTGTGATTAAGGGACAAATATCTGAGAAAAGTACTTTT
TTATCTTTACCTTGGGACTATTCCCAGGAGTAAAACACGCACAGTTTCACCCTGCATAAGGTAACAGCCTGCACAGGCCCTGCAGCTCC
AGATCAGTCACTGCAAAGTGCCATGAATTTCTACAAACATAAAGAATAAACAAAGAGTTGCTCTGCTGCCTTTCCTAACTGGCACTGTC
TCCGGACATCCCCATTGCCTGGGTGCTGTCCATGTACCTCCCCTCCCGCTGTGGATGCCTGTCTATTCTGATGTGCCCCTCTCTAGCTG
CTCCGAGAGGCACAGCTTTCACAGGCAGAGCAGGCAGACCCTGCAAACGGCACTACGGGCGTGTTCAAATGCACAGGTGTTTCACAGGC
ACGTACACATTTTAATGCATCAAGCAACAGAGCTCTGGGAGAAAAAAATAATTAATGAAGCGGTGCTTAGGCTTATACAGAGGCCACTG
AGGCTAAGCTCATACTGGGGCTGGTGGGCTGGTGTCAGTGGTTTATGGAATGTTCCCCTTGAATTATGAGAGAAAGGGCTAATCCTATG
GCTGCCGCTCCTTCAAGGTGCCAGCACAGTACTTGACGGTGCCACCCCTTCTCTCTTCCCTCATCCCCCCATTACGGTTTTCATCATTC
TCTTTGCCCACGAACAGGAACTTTTTATTGTCAGCTTTTGGGATAAACACATAAAAAGTTAAGAAAACGTTGCAGCAGAGGGAGGATTG
CATCAATGAAGATGAGTAGGCAGCAAAGCCTCTCCCATTCACCCGTAACACCTCGTCTGCCCTGAGTTCATAGCACCTGAACCACTGAA
CGAGGCAATACCCCTTGTGAAGGAGATGGCAGCTTGAATGTGAGTGTCCTCCTTGCAGGGACAGGTAAGATAGTGGTATGAGGAGGTAT
CACACGCAAGACCCCAGGCACAGTCGCTCCTGAGCTCCTCTGTCAGAAGCAGGCACAACTGGCATTTGAAAGGCACTGGTGGCCCTGAA
GAGCAAAGTCAGCCTAACCCTGCCCACTACACACTGAAAACCAAAAGGTCAGTATGTTTGGATGAGGAACCAGCAAAAAAACCTTCTTT
ATTTAAAAAAAAAAATATTGTCCATGTGGGCAGCACTGAGTGTTTCTGCAGGCAAGTCTGTTTAGACTGTCCCTCTCTTAGCAAGTAATT
CTTTTTCTCCAAAGTGTTAATTAGACATTCATATTAAGTGGGTCTTAATACACATAAAGACAAGAAATTGTATTTTGCTCTCATCCAAC
TGCGAGTGCTTCAAGCTGATCAGTCACTTCAAAGTAACCAAAAACCTAGTCTTACCAGCTTCCAACGACAGGGAGGACCACCCTGGCAA
GTGATATGTGGCAAGAACATGAAGTCATACACCACCTCCTCCCAGTCCCCAGACACGGGGCCAGGGGCCAGAGAAGAGGGGTTCGTTTG
GTGTGCTGTACCACAGGCCACCCC

>CASQ2_Cathartes_melambrotus_CDS
ATGAAGGCGACTTGCTGGATCCTGGCAGGTTTTTACCTGCTTTTCTGCTGCAAGGCAGAAGAGGGACTGAATTTCCCCACTTACGATGG

GAAAGACCGAGTGATCGACCTGAATGAGAAGAACTACAAGCAGGCCTTGAAGAAGTACGACATGCTCTGCCTGCTCTTCCACGAGCCTG
TGAGCTCTGACAAGGTCTCCCAGAAACAGTTCCAGATGACAGAGATGGTCCTGGAGCTGGCGGCTCAGGTCCTGGAGCCCAGAAGCATC
GGCTTCGGGATGGTGGACTCCAAGAAGGACGCCAAACTTGCCAAAAAATTAGGCTTGGTTGAAGAAGGAAGCCTCTATGTCTTTAAGGA
GGACCGGTTGATTGAATTTGATGGGGAACTGGCCACGGATGTCTTGGTGGAATTCCTCTTGGATCTGCTAGAAGACCCTGTGGAGATCA
TAAACAACAAGCTGGAACTTCAGGCCTTTGACCAAATCGACGAGGAAATCAAACTCATCGGCTACTTCAAAGGAGAAGACTCTGAACAT
TACAAGGCATTTGAAGAAGCTGCTGAACACTTCCAGCCATACATCAAGTTCTTTGCCACCTTCGACAAAGGGGTTGCCAAGAAGCTAGG
TCTGAAGATGAACGAGGTGGACTTCTATGAACCGTTCATGGATGAGCCTGTTCACATCCCCGATAAGCCTTACACAGAAGAGGAGCTGG
TTGAATTTGTGAAGGAGCACAAAAGGGCCACCTTGCGGAAGCTGCGCCCAGAGGACATGTTTGAGACGTGGGAGGATGACATGGAGGGA
ATCCACATCGTGGCCTTCGCCGAAGAAGATGACCCAGATGGTTTTGAGTTCCTGGAAATCCTGAAGCAGGTTGCCAGGGACAACACCGA
TAATCCCGACCTAAGCATTGTCTGGATCGACCCCGACGACTTTCCTCTGCTGATCACTTACTGGGAGAAGACCTTCAAGATTGACCTGT
TCAGACCACAGATCGGGGTGGTGAACGTCACAGATGCTGACAGCGTCTGGATGGAGATCAGAGATGACGATGACCTGCCCACAGCCGAG
GAGCTGGAGGACTGGATAGAGGACGTGCTTTCTGGGAAGATAAATACCGAAGATGATGACGACGATGATGACGATGATGACGACGATGA
CGACGATGACGACGATGATGACGACGATGATGACGACGATGACGACGATGACGACTAA

>CASQ2_Cathartes_melambrotus_protein
MKATCWILAGFYLLFCCKAEEGLNFPTYDGKDRVIDLNEKNYKQALKKYDMLCLLFHEPVSSDKVSQKQFQMTEMVLELAAQVLEPRSI
GFGMVDSKKDAKLAKKLGLVEEGSLYVFKEDRLIEFDGELATDVLVEFLLDLLEDPVEIINNKLELQAFDQIDEEIKLIGYFKGEDSEH
YKAFEEAAEHFQPYIKFFATFDKGVAKKLGLKMNEVDFYEPFMDEPVHIPDKPYTEEELVEFVKEHKRATLRKLRPEDMFETWEDDMEG
IHIVAFAEEDDPDGFEFLEILKQVARDNTDNPDLSIVWIDPDDFPLLITYWEKTFKIDLFRPQIGVVNVTDADSVWMEIRDDDDLPTAE
ELEDWIEDVLSGKINTEDDDDDDDDDDDDDDDDDDDDDDDDDDDDDDD*

6.8 *Chroicocephalus ridibundus* (black-headed gull)

From: Trinity *de novo* assembly of ERR12321242. CASQ1 from TRINITY_DN884_c0_g1_i8. CASQ2 from complement of TRINITY_DN2071_c0_g2_i2.

>CASQ1_Chroicocephalus_ridibundus_transcript_assembly
GGGGCCAGGCAGGGGCTGCGCGTCCGTCCCCTGCGCTGGTCCCAGCCGGGCTCCGTCCGTCCGTCCGTCCCTGCATCGTCCCTCTGTCC
CCGGGGCTGTGTCTGTCTGTCTGTCTGTCTGTCCCCCTGCGGCTGCCGTCTGTCCCCAGGGCCGTGCGTCTGTCCCCAGGGCCGTCTGT
CCGTCCCCAGGGCCGTCTGTCCGTCCCCTGGCTGTGCGTCCGTCCCCACCCGTCCGTGAGTCCCGTCCCTGTCTGTCCGTGGGTCCCAT
CTCTTTCTGTCCGTCCGTCCGTCCGTCCGTCCCCTGGCCGTGTGTCCCTCCCTGCTCGTCCGTGAGTCCTGTCCCCCGCTGTCCGTCTG
TCCGTCCTTACCTGTCCCTGAGGCCAGTCCCCATCTGTCCGTCTGTCTGTCCCCAACTCCTGTCCCTGTCTGTCCGTGAGTGCTGTCCC
CTTCTGTCCCCGTCCGTCTGTCCGTCTGTCTCTGCAGCTCCATATCTGCCCTTGGGCCCCATCCACCCGCCCGTCCGTGTGTCTGTCCC
CGCTCTCCGTGTATCCGTCCCCATCCGTGTATCCATCCCCATCCGTGTGTCCATCCCCATCCGTGTATCCGTCCCCGTCCGTGTGTCTG
TCCCTGTCCGTGTATCCATCCCTGTCTGTGTGTCCATCCCCATCCGTGTGTCCGTCCCCATCCCTGTGTCCATCCCTCTCCGTGTATCC
ATCCCCGTCCGTCTGCACGTCCCCGTCCGTGTGTCCATCCCTGTCCATCTGTCCGTCCCCATCCGTGTATCCGTCCCCGTCCGTCTGTC
CGCCCGGCGGGCGATGGGGCTGTGGAGGTGGGTGCTGGCGCTGTTGGCGCTGGGTGCCGGGTGCCCGGGGGGGGCCAGGGAGGGCCTGG
ACTTCCCCACCCCCGATGGCCTGGACCGGGTGCTGCCCGTCACCCTGAAGAACTACAAGGCGCTGCTGAAGCGGTTCCCGGTGGTGGCC
CTGCTCCACCACCGCCCCCCCCGCGGTGACCGGGGCGCCCAGCGCCACCAGGAGATGGAGGAGCTGGTCCTGGAGCTGGCAGCCCAGGT
GCTGGAGGACAAGGGAGTGGGCTTCGGCCTCGTCGACTCTGAGAAGGACGCGGCCGTCGCCAAAAAGCTGGGCCTGACGGAGGAGGACA
GCATCTACGTCTTCAAGGAGGACGAGGTGATCGAGTACGACGGGGAGCTGGCGGCGGACACGCTGGTGGAGTTCCTGCTGGATGTGCTG
GAGGACCCGGTGGAGTTCATTGAGGGCGACCACGAGCTCCAGGCCTTCGAGAACATCGAGGACGACCCCAAACTCATCGGCTACTTCAA
GAACGAGGAGTCGGAGCACTTCAAAGCCTTCGAGGAGGCGGCGGAGGAGTTCCACCCCTACATCCCCTTCTTCGCCACCTTCGACAGCA
AGATGGCCAAGAAGCTGACGCTGAAGCTGAACGAGATCGACTTCTACGAGCCCTTCATGGAGGAGCCAAAGACCATCCCCGACCGGCCC
AACAGCAAGGAGGAGATCGTGGCCTTCGTGGAGGAGCACAAGAGGGCCACGCTGCGGAAACTCAAACCCGAGAGCATGTACGAGACCTG
GGAGGACGACATGGACGGGATCCACATCGTGGCTTTCGCAGAGGAGGATGATCCGGATGGGTTCGAGTTCCTGGAGATCCTGAAGGACG
TGGCCCAGGACAACACGGACAACCCCGACCTCAGCATCATCTGGATCGATCCCGAGGATTTCCCGCTGCTCATCCCTTACTGGGAGAAA
ACCTTCAACATCGACCTGTCCCGGCCCCAGATCGGGGTGGTCAACGTCACCGACGCCGACAGCGTATGGCTGGAGATGACGGACGAGGA
GGATCTGCCGGGGCCGGAGGAGCTGGAAGAGTGGCTTGAGGACGTGCTGGCGGGGGACATCAACACCGAGGACGACGACGATGACGACG
ACGACGACGACGACGACGATGACGACTAGGCCTCGTGCTGGCCCCAGCCCTGTCCCCCATTAAAGTCCCCAAAGAACCAGGCTGTGTCG
CCGTGGGGGGGGGGACACACACACAAGATCGGAAGAGCACACGTCTGAACTCCAGTCACATATGCGCATCTCGTATGCCG

>CASQ1_Chroicocephalus_ridibundus_CDS
ATGGGGCTGTGGAGGTGGGTGCTGGCGCTGTTGGCGCTGGGTGCCGGGTGCCCGGGGGGGGCCAGGGAGGGCCTGGACTTCCCCACCCC
CGATGGCCTGGACCGGGTGCTGCCCGTCACCCTGAAGAACTACAAGGCGCTGCTGAAGCGGTTCCCGGTGGTGGCCCTGCTCCACCACC
GCCCCCCCCGCGGTGACCGGGGCGCCCAGCGCCACCAGGAGATGGAGGAGCTGGTCCTGGAGCTGGCAGCCCAGGTGCTGGAGGACAAG
GGAGTGGGCTTCGGCCTCGTCGACTCTGAGAAGGACGCGGCCGTCGCCAAAAAGCTGGGCCTGACGGAGGAGGACAGCATCTACGTCTT
CAAGGAGGACGAGGTGATCGAGTACGACGGGGAGCTGGCGGCGGACACGCTGGTGGAGTTCCTGCTGGATGTGCTGGAGGACCCGGTGG
AGTTCATTGAGGGCGACCACGAGCTCCAGGCCTTCGAGAACATCGAGGACGACCCCAAACTCATCGGCTACTTCAAGAACGAGGAGTCG
GAGCACTTCAAAGCCTTCGAGGAGGCGGCGGAGGAGTTCCACCCCTACATCCCCTTCTTCGCCACCTTCGACAGCAAGATGGCCAAGAA
GCTGACGCTGAAGCTGAACGAGATCGACTTCTACGAGCCCTTCATGGAGGAGCCAAAGACCATCCCCGACCGGCCCAACAGCAAGGAGG
AGATCGTGGCCTTCGTGGAGGAGCACAAGAGGGCCACGCTGCGGAAACTCAAACCCGAGAGCATGTACGAGACCTGGGAGGACGACATG

GACGGGATCCACATCGTGGCTTTCGCAGAGGAGGATGATCCGGATGGGTTCGAGTTCCTGGAGATCCTGAAGGACGTGGCCCAGGACAA
CACGGACAACCCCGACCTCAGCATCATCTGGATCGATCCCGAGGATTTCCCGCTGCTCATCCCTTACTGGGAGAAAACCTTCAACATCG
ACCTGTCCCGGCCCCAGATCGGGGTGGTCAACGTCACCGACGCCGACAGCGTATGGCTGGAGATGACGGACGAGGAGGATCTGCCGGGG
CCGGAGGAGCTGGAAGAGTGGCTTGAGGACGTGCTGGCGGGGGACATCAACACCGAGGACGACGACGATGACGACGACGACGACGACGA
CGACGATGACGACTAG

>CASQ1_Chroicocephalus_ridibundus_protein
MGLWRWVLALLALGAGCPGGAREGLDFPTPDGLDRVLPVTLKNYKALLKRFPVVALLHHRPPRGDRGAQRHQEMEELVLELAAQVLEDK
GVGFGLVDSEKDAAVAKKLGLTEEDSIYVFKEDEVIEYDGELAADTLVEFLLDVLEDPVEFIEGDHELQAFENIEDDPKLIGYFKNEES
EHFKAFEEAAEEFHPYIPFFATFDSKMAKKLTLKLNEIDFYEPFMEEPKTIPDRPNSKEEIVAFVEEHKRATLRKLKPESMYETWEDDM
DGIHIVAFAEEDDPDGFEFLEILKDVAQDNTDNPDLSIIWIDPEDFPLLIPYWEKTFNIDLSRPQIGVVNVTDADSVWLEMTDEEDLPG
PEELEEWLEDVLAGDINTEDDDDDDDDDDDDDDD*

>CASQ2_Chroicocephalus_ridibundus_transcript_assembly
CTTTGTTGTGAGCCCAGGACAGCAGAGTTTGCTGCTCCCCACCAGCCTTGCAGGGTTTGGGCTGATCACTAGGAAGGCAGCAGAAAGAG
AAACTCAGGTCAAACTTCACCTGGAGCCTTTGGTCTCCCCCTCTTCCCAAATTAAGAAGGAGGGGGACGTTTACCTGTCCTCCGCTGCA
CTCCAAGGCCTCCCCTATGAAGGCGACTTGCTGGATCCTGGCAGGTTTTTACCTGCTTTTCTGCTGCAAGGCAGAAGAGGGACTGAACT
TCCCCACTTATGATGGGAAAGACCGAGTGATCGACTTGAACGAGAAGAACTACAAGCAGGCCCTGAAGAAGTATGACATGCTCTGCCTG
CTCTTCCACGAGCCCGTAAGCTCTGACAAGGTCTCCCAGAAGCAGTTCCAGATGACAGAGATGGTCTTGGAGCTGGCGGCTCAGGTCCT
GGAGCCCAGGAGCATTGGCTTCGGGATGGTAGACTCCAAGAAGGATGCCAAACTTGCTAAAAAGCTAGGCTTGGTTGAAGAGGGAAGTC
TCTATGTCTTTAAGGAAGAGCGGTTGATTGAATTTGATGGGGAACTGGCCACGGATGTCTTGGTGGAATTTCTCTTGGATCTGCTAGAA
GACCCTGTGGAGATCATAAACAGCAAGCTGGAGCTTCAGGCCTTTGACCAAATCGATGAGGAAATCAAACTCATTGGCTACTTCAAAGG
AGAAGACTCTGAACATTACAAAGCATTTGAAGAAGCTGCTGAACACTTCCAGCCCTACATCAAGTTCTTCGCCACATTCGACAAAGGGG
TTGCCAAGAAGCTAGGTCTGAAGATGAATGAGGTGGACTTCTATGAACCATTTATGGATGAACCTGTTCACATCCCTGATAAGCCTTAC
ACAGAAGAGGAGCTGGTTGAATTTGTGAAGGAGCACAAAAGGGCCACCTTGAGGAAACTGCGCCCAGAGGACATGTTTGAGACGTGGGA
GGATGACATGGACGGAATCCACATTGTGGCCTTCGCCGAAGAAGATGACCCAGATGGTTTTGAGTTCCTGGAAATCCTGAAGCAGGTTG
CCAGGGACAACACCGATAATCCTGACCTGAGCATTGTCTGGATTGACCCCGACGACTTTCCTCTGCTGATCACTTACTGGGAGAAGACT
TTCAAGATTGACCTGTTCAGACCACAGATCGGGGTGGTGAACGTCACAGACGCTGACAGCATCTGGATGGAGATCAGAGACGATGATGA
CCTGCCCACGGCCGAGGAGCTGGAGGACTGGATAGAGGATGTGCTTTCTGGGAAGATAAATACCGAAGATGATGATGACGATGATGACG
ATGATGACGATGATGATGATGACGACGACGATGATGATGATGATGATGACGACGACGACGACGATGACGATGACTAACTGTGACTCTGT
GCAGTTTGACTTGTGGGGGCTGAGAGGCCTCCCTCCGTGGCAGGTCCTTCCATCGGAAGACCTGTGTTTGAGCGTCAGCAAGCACCGCT
GCTCCTCTTCCCCGCCCCCCCCCCCCGCCCCCGCCGGGCCCCCCCCCCCCCCTCCCCCCCCACACCCCCCCCACCACCCCCCCCACCC
CGCCGCGGCGGGTTCCCCGTCGTCGCCATGGCG

¿CASQ2_Chroicocephalus_ridibundus_CDS
ATGAAGGCGACTTGCTGGATCCTGGCAGGTTTTTACCTGCTTTTCTGCTGCAAGGCAGAAGAGGGACTGAACT

¿CASQ2_Chroicocephalus_ridibundus_protein
MKATCWILAGFYLLFCCKAEEGLNFPTYDGKDRVIDLNEKNYKQALKKYDMLCLLFHEPVSSDKVSQKQFQMTEM

6.9 *Coturnix japonica* (Japanese quail)

From: Trinity *de novo* assembly of SRR2968876. CASQ1 from complement of TRINITY_DN225_c0_g1_i6. CASQ2 from TRINITY_DN659_c0_g3_i3.

>CASQ1_Coturnix_japonica_transcript_assembly
CAGTTGAGGGGGGGGGCGGGTTGTGACCCCCCCATGGTGCACCGTGACCCCCCGAGGGGGACCCTGCCCGCCCCACGGGGATCGCCGGC
CTGTGCCCCCCCTTTGTGCCCCATCTATAGAATCTATACAACAGCGGGGGGGGACGTTGGGGGGGGGGTCACGCTGTCACCCCCGTCAC
CGCCCCCCGGCCGGAAGGGGCTGTGACTGCCCGCTCCCCGGGGGGGGGAAGGAGTCCGTCTGTCCGTCCGTCCGTTCGTCGCTGTGGGT
CCGGCTGTGGGTCCGTGTGGGGCTGTGAGTCCGTCTGTCCGTCCCTGTGGGGCTCTGAGTGCCTGTGTGTCGCTGAGGGTCCGTCTGTC
CGTCCCTGTGGGGCTGTGAGTCCGTCTGTCCGTCCCTGTGGGGCTGTCGGTCCGTCTGTCCGTCCCTGTGGGGCTGTCGGTCCGTCTGT
CCGTCCCTGTGGGGCTGTGGGTCCGTCTGTGGGTCCGTCTGCCCCACCAGATCCGGCCCGTCCGTCTCTGAGCATTGACGTGTCCCCCA
GGTGCTGCCCGTCCGTCCGTCCGTCCGTCCCAACCCTCCGTCCCCTCATTCACATCCATCCCTCCGTCCGTCTGTCCGTCCCGATCCGA
CCCCCCCGTCCCCTCCGTCCGTCCGTCCGTCCCCGCAGCCCGCCCCGTCCTCTCCCGTGCCCGCAGCTCCTCCTGTCTCCCGGTTCGAT
GTGGGGCTGCCGGTGGCTGCTGGCGCTGCTGGCGCTGGGGGCGTCCGCCCCGGGGGGGGCCGAGGAGGGGCTGCGCTTCCCCTCCCACG
ACGGGGCGCCCCGCGTCGTGTCCGTCACCGCCCTCAATCACCGCGAGGCGCTGCGCAGCTCCGCGGTGCTCGCGCTGCTCCTGCACCGA
CCCGGGGGGGCCCGGAGCGAACGGGAGGAGAGAGTGCTCGAGCTGGCAGCGCAGGTGCTGGAGGACAAAGGAGTGGGGTTCGGCCTCGT
GGATGCAGAGAAGGAGGCGGATACAGCCCAGAAGTTGGGGATGACGGAGGAGAACAGCATCTACATCTTTAAGGGGGACGAGGTGATTG
AGTACGACGGCGAGCTGTCGGCCGACACGCTGGTGGAGTTCCTGCTGGATGTGCTGGAGGACCCGGTGGAGTTCATTGAGGGCGACCAC
GAGCTGCACGCGTTCGAGAACATTGAGGAGGACCCCAAAGTCATCGGATACTTCGAGGGGGAGGAGTCAGAGAATTTCAAAGCCTTCTC
AGCGGCAGCGGCAGAATTCCACCCCTACATCCCCTTCTTTGCCACCTTCGACCCCAAGGTGGCCAGGAAACTGACCCTAAAGCTCAACG
AGATCGACTTCTATGAGCCCTTCATGGAGGAACCGCTCACGCTGCTGACGCAGCCCCACGGCAGAGAGGAGATCGCCCGCTTCGTGGAG
GAGCACAAGCGGGCAACGCTGCGGAAGCTCAAACCTGAGAGTATGTACGAGACATGGGAGGACGATATCGATGGGATTCATATCGTGGC

CTTTGCAGAGGAGGATGATCCTGACGGCTTTGAGTTCCTGGAGACCCTGAAGGACGTGGCCCGGGACCACACTGAGAACCCCAAACTCA
GCATCATCTGGATCGACCCCGAGGACTTCCCGCTGCTCATCCCCTTCTGGGAGAAAACCTTCGGCATCGACCTGTCCCGGCCACAGATC
GGAGTGGTCAACGTCACCGATGCTGACAGCGTGTGGCTGCCCATGGCGGATGAGAACGACCTTCCGGACGCGGCGGAGCTGGACGCGTG
GATCGGGGCCGTCCTGGAGGGGGAGGTCAGCACCGAGAGCGATGACGGACATTAGGGGCAGCCCGGCCCCGCATCGCGGCCATTAAAGC
GCTTTAATACAGGACAGAACCGACACTTAATACAGGACAGAACCGACACTTAATACCGGACAGAACCGACACGGCGCTGTGGGACTGCG
GGACATGGGGGGAACCGTGCGGGGTGGAACCGGGTGTGTGGGATGGAGGGGACAGCGCAGGGACGGGACATGGGGACACCCCAAAGATG
GGGCACCCTGCAGGGGTGGGGTCCCCCGCCGGGGTCGCCCCGTTGTTGCGGACAGACCCGCCGGGGCTGGGACACGCGGGGCTGAACCC
GGCCGGGGTCGGGTCGGTCCGTGTCCGCCGGGATCCCCCCCCCCAAATCCCCCCAGACCGGGAAAT

>CASQ1_Coturnix_japonica_CDS
ATGTGGGGCTGCCGGTGGCTGCTGGCGCTGCTGGCGCTGGGGGCGTCCGCCCCGGGGGGGGCCGAGGAGGGGCTGCGCTTCCCCTCCCA
CGACGGGGCGCCCCGCGTCGTGTCCGTCACCGCCCTCAATCACCGCGAGGCGCTGCGCAGCTCCGCGGTGCTCGCGCTGCTCCTGCACC
GACCCGGGGGGGCCCGGAGCGAACGGGAGGAGAGAGTGCTCGAGCTGGCAGCGCAGGTGCTGGAGGACAAAGGAGTGGGGTTCGGCCTC
GTGGATGCAGAGAAGGAGGCGGATACAGCCCAGAAGTTGGGGATGACGGAGGAGAACAGCATCTACATCTTTAAGGGGGACGAGGTGAT
TGAGTACGACGGCGAGCTGTCGGCCGACACGCTGGTGGAGTTCCTGCTGGATGTGCTGGAGGACCCGGTGGAGTTCATTGAGGGCGACC
ACGAGCTGCACGCGTTCGAGAACATTGAGGAGGACCCCAAAGTCATCGGATACTTCGAGGGGGAGGAGTCAGAGAATTTCAAAGCCTTC
TCAGCGGCAGCGGCAGAATTCCACCCCTACATCCCCTTCTTTGCCACCTTCGACCCCAAGGTGGCCAGGAAACTGACCCTAAAGCTCAA
CGAGATCGACTTCTATGAGCCCTTCATGGAGGAACCGCTCACGCTGCTGACGCAGCCCCACGGCAGAGAGGAGATCGCCCGCTTCGTGG
AGGAGCACAAGCGGGCAACGCTGCGGAAGCTCAAACCTGAGAGTATGTACGAGACATGGGAGGACGATATCGATGGGATTCATATCGTG
GCCTTTGCAGAGGAGGATGATCCTGACGGCTTTGAGTTCCTGGAGACCCTGAAGGACGTGGCCCGGGACCACACTGAGAACCCCAAACT
CAGCATCATCTGGATCGACCCCGAGGACTTCCCGCTGCTCATCCCCTTCTGGGAGAAAACCTTCGGCATCGACCTGTCCCGGCCACAGA
TCGGAGTGGTCAACGTCACCGATGCTGACAGCGTGTGGCTGCCCATGGCGGATGAGAACGACCTTCCGGACGCGGCGGAGCTGGACGCG
TGGATCGGGGCCGTCCTGGAGGGGGAGGTCAGCACCGAGAGCGATGACGGACATTAG

>CASQ1_Coturnix_japonica_protein
MWGCRWLLALLALGASAPGGAEEGLRFPSHDGAPRVVSVTALNHREALRSSAVLALLLHRPGGARSEREERVLELAAQVLEDKGVGFGL
VDAEKEADTAQKLGMTEENSIYIFKGDEVIEYDGELSADTLVEFLLDVLEDPVEFIEGDHELHAFENIEEDPKVIGYFEGEESENFKAF
SAAAAEFHPYIPFFATFDPKVARKLTLKLNEIDFYEPFMEEPLTLLTQPHGREEIARFVEEHKRATLRKLKPESMYETWEDDIDGIHIV
AFAEEDDPDGFEFLETLKDVARDHTENPKLSIIWIDPEDFPLLIPFWEKTFGIDLSRPQIGVVNVTDADSVWLPMADENDLPDAAELDA
WIGAVLEGEVSTESDDGH*

>CASQ2_Coturnix_japonica_transcript_assembly
CTAACCTCCACTCCCTCCTGCGCTCCCTCACACTCCCTCTCACTCGCTCCCTCGCTCTCAGATGATGCAGAGTGCCAGCAAGTCCCTCA
ATGCTGTCATATCTGTCCTTTTTATTCATTGCAGGGTTTATTTTTAGCCTGAAACAACTGCTTCCTAAAAATGGAGTTCCTAATGACAC
GGGAGCTGGACACAGCTATGTAAGGTATCCAGGGCTTGGCCTGACAGCTTCTCCTGTCTGTCTCTTTGTTGTGAGCCCAGGACAGCAAA
GTTTGTTGCTTGCCACCAGCCTTGCAGAGCTTGGACTGAACACTAGGAAGGCAGCAGAAAGAGAAACTCGGTGCAGACGCCCACTGGAG
CCCTTGGCCTCCTTCTCTTCCCAAACTGAGAAGGAGAGAGACCTTTGCCTGCGCTGCACTGAACTCCAAGGGTTCCCCAATGAAGGCGA
CTTGCTGGATCCTGGCAGGCTTTTGCCTGCTTTTCTGCTGCAAGGCGGAAGAAGGACTCAATTTCCCCACATATGATGGGAAAGACCGT
GTGATCGACCTGAATGAGAAGAACTACAAGAGTGCCCTGAAGAAGTATGACATGCTTTGTCTGCTCTTCCACGAGCCGGTGGGCTCCGA
CAGGGTGTCCCAGAAGCAGTTCCAGATGACAGAGATGGTCCTGGAGCTGGCAGCTCAGGTCCTGGAGCCTAGAAGCATTGGCTTTGGGA
TGGTGGATTCCAAGAAGGATGCCAAACTCGCCAAAAAGCTTGGCTTGGTTGAAGAGGGAAGTCTCTATGTCTTTAAGGAGGAACGGTTG
ATTGAATTTGATGGGGAACTGGCCACAGACGTCTTGGTGGAATTCCTCTTGGATCTGCTAGAAGACCCCGTGGAGGTCATAAACAGCAA
GCTGGAGCTTCAGGCCTTTGACCAAATTGACGACGAAATCAAACTTATCGGCTACTTCAAAGGAGAAGACTCTGAACATTATAAGGCAT
TTGAAGAGGCTGCTGAACACTTCCAGCCATATGTCAAATTCTTTGCCACATTCGACAAAGGGGTTGCCAAGAAACTGGGTCTGAAGATG
AATGAGGTGGACTTCTATGAACCATTCATGGATGACCCTGTTCACATCCCCGATAAGCCTTACACAGAAGAGGAGCTGGTTGAATTTGT
GAAGGAGCACAAAAGAGCCACCTTGCGGAAGCTGCGTCCAGAAGACATGTTTGAGACATGGGAAGATGACATGGAAGGAATCCATATCG
TGGCCTTTGCTGAAGAAGATGACCCAGACGGCTTTGAGTTCCTGGAAATCCTGAAGCAGGTTGCCAGGGACAACACTGATAATCCTGAC
CTGAGCATTGTGTGGATTGACCCTGACGACTTTCCTCTGCTCATCACTTACTGGGAGAAGACATTCAAGATTGACCTGTTCAGGCCGCA
GATTGGGATTGTGAATGTCACAGACGCTGACAGTGTCTGGATGGACATCAGAGATGATGATGACCTGCCCACAGCCGAGGAGCTGGAGG
ACTGGATAGAAGATGTGCTTTCTGGGAAGATAAACACTGAAGATGATGACGACGACGATGATGATGATGACGACGACGATGATGATGAT
GACGATGATGATGACGATGATGACGACGATGATGATGACGATGATGATGACTAACTGTGACTCTGTACAGTTTGACTTGTGGAGGCCAA
GAGGCCTCCCCCTGTGGCAAGTCCCTTCATCAGAAGACCTGTGTTTGAGCATCAGCAAGCACCGCCTGCTCCTTCTTTCCCCCTTGCCC
TGCTCCCCCTACCCCTGCTGGGACCCCCGGCCTGATTCTCAGAGGTGCTGAGCCACCAGGACTGCCACCTTTGCAGGTGGCACCGGCAG
GTGCCCAGCACCGCTGTAAATCATGCCTCCGTAGAAAGGACAATAGGAATCTGTAGGGAACCTGCCCCAGTGCCCAGTGCAGGAAGAAA
GCACCTGCATGCTCACTGCCAAAGGGACACAGGGAGCCAGTTTGTATTTTCCATGCTCATCGGCCCATCATGTACATCCAAAGACCAAA
TCTCACTCGATACAGAGGGAAGGTCACTGTAAAATTTAGTCCTTTCATTAAACAAAATGCCCTTTGCAGCTACTCTGCCAAGACCACTG
GATCCTGAAAGTTCTACTCTAGGAGTGTACAGCTACTCATTAACATACTAAGAGCAGCAACACATTTGAGGGGGGGAAGACAGGTTTAG
GAACACTAGCTAGTGTTCCTA

>CASQ2_Coturnix_japonica_transcript_CDS
ATGAAGGCGACTTGCTGGATCCTGGCAGGCTTTTGCCTGCTTTTCTGCTGCAAGGCGGAAGAAGGACTCAATTTCCCCACATATGATGG
GAAAGACCGTGTGATCGACCTGAATGAGAAGAACTACAAGAGTGCCCTGAAGAAGTATGACATGCTTTGTCTGCTCTTCCACGAGCCGG

TGGGCTCCGACAGGGTGTCCCAGAAGCAGTTCCAGATGACAGAGATGGTCCTGGAGCTGGCAGCTCAGGTCCTGGAGCCTAGAAGCATT
GGCTTTGGGATGGTGGATTCCAAGAAGGATGCCAAACTCGCCAAAAAGCTTGGCTTGGTTGAAGAGGGAAGTCTCTATGTCTTTAAGGA
GGAACGGTTGATTGAATTTGATGGGGAACTGGCCACAGACGTCTTGGTGGAATTCCTCTTGGATCTGCTAGAAGACCCCGTGGAGGTCA
TAAACAGCAAGCTGGAGCTTCAGGCCTTTGACCAAATTGACGACGAAATCAAACTTATCGGCTACTTCAAAGGAGAAGACTCTGAACAT
TATAAGGCATTTGAAGAGGCTGCTGAACACTTCCAGCCATATGTCAAATTCTTTGCCACATTCGACAAAGGGGTTGCCAAGAAACTGGG
TCTGAAGATGAATGAGGTGGACTTCTATGAACCATTCATGGATGACCCTGTTCACATCCCCGATAAGCCTTACACAGAAGAGGAGCTGG
TTGAATTTGTGAAGGAGCACAAAAGAGCCACCTTGCGGAAGCTGCGTCCAGAAGACATGTTTGAGACATGGGAAGATGACATGGAAGGA
ATCCATATCGTGGCCTTTGCTGAAGAAGATGACCCAGACGGCTTTGAGTTCCTGGAAATCCTGAAGCAGGTTGCCAGGGACAACACTGA
TAATCCTGACCTGAGCATTGTGTGGATTGACCCTGACGACTTTCCTCTGCTCATCACTTACTGGGAGAAGACATTCAAGATTGACCTGT
TCAGGCCGCAGATTGGGATTGTGAATGTCACAGACGCTGACAGTGTCTGGATGGACATCAGAGATGATGATGACCTGCCCACAGCCGAG
GAGCTGGAGGACTGGATAGAAGATGTGCTTTCTGGGAAGATAAACACTGAAGATGATGACGACGACGATGATGATGATGACGACGACGA
TGATGATGATGACGATGATGATGACGATGATGACGACGATGATGATGACGATGATGATGACTAA

>CASQ2_Coturnix_japonica_protein
MKATCWILAGFCLLFCCKAEEGLNFPTYDGKDRVIDLNEKNYKSALKKYDMLCLLFHEPVGSDRVSQKQFQMTEMVLELAAQVLEPRSI
GFGMVDSKKDAKLAKKLGLVEEGSLYVFKEERLIEFDGELATDVLVEFLLDLLEDPVEVINSKLELQAFDQIDDEIKLIGYFKGEDSEH
YKAFEEAAEHFQPYVKFFATFDKGVAKKLGLKMNEVDFYEPFMDDPVHIPDKPYTEEELVEFVKEHKRATLRKLRPEDMFETWEDDMEG
IHIVAFAEEDDPDGFEFLEILKQVARDNTDNPDLSIVWIDPDDFPLLITYWEKTFKIDLFRPQIGIVNVTDADSVWMDIRDDDDLPTAE
ELEDWIEDVLSGKINTEDDDDDDDDDDDDDDDDDDDDDDDDDDDDDDDDDDD*

6.10 *Crossoptilon crossoptilon* (white-eared pheasant)

From: Trinity *de novo* assembly of SRR24187829. CASQ1 from complement TRINITY_DN961_c0_g1_i1. CASQ2 from complement TRINITY_DN1090_c0_g1_i1.

>CASQ1_Crossoptilon_crossoptilon_transcript_assembly
GCCCTGTCACCGCCCCATGGCCGAAAGGGGCTGCGGCTGTCCCCTCCCCGGGGGGGCTGGGAGGGAAGGCGTCCGCCTGTCTGTCCGTC
CGTTGCTGTGGGTCCGGCTGTGGGTCCCTGTGGGGCTGTGGGTCCATCCGTCCCTGTGGGGCTCTCCTCCCGTCTGTCCGTGCGATGTT
GGGCTGCCGGTGGCTGCTGGCGCTGCTGGCGGTGGGGTTTCCCCCCCCGGGGGGGGCCGAGGAGGGGCTCCGCTTCCCCTCCCACGACG
GAGCGCCGCGGGTTCTGTCCGTCACAGCCAGCAATTACCGCGCGGCGCTGCGGGGCTCCCCGGTGGTCGCGCTGCTCCTTCATCGGCCC
GGGGGGACGCGGAGGGAACGGGAGGAGCGCGTGCTCGAGCTGGCGGCGCAGGTGCTGGAGGACAAAGGAGTGGGGTTTGGCTTCGTGGA
TGCGGAGAAGGATGTGGCCACGGCCCAGAAGTTGGGGATGACGGAGGAGAACAGCATCTACATCTTTAAGGGAGATGAGGTGATTGAGT
ACGACGGCGAGCTGTCAGCCGACACGCTGGTGGAATTCCTGCTGGATGTGCTGGAGGACCCTGTGGAGTTCATTGAGGGTGACCATGAG
CTGCGTGCGTTCGAGAACATTGAGGAGGACCCCAAAGTCATCGGATACTTCAAGGGGGTGGAATCAGAGTATTTCAAAGCCTTCTCGGC
GGCAGCAGCAGAGTTCCACCCTTACATCCCCTTCTTTGCCACCTTTGACCCCAAGGTGGCCAGGAAACTGACCCTAAAGCTCAACGAGA
TCGACTTCTATGAACCCTTCATGGAGGAACCACTCACACTCCCCACACCGCCCCAAGGCAAAGAGGAGATCGCCGCCTTCGTGGAAGAG
CACAAGCGGGCAACACTGCGGAAGCTCAAACCTGAGAGTATGTATGAGACATGGGAGGATGACATTGATGGGATTCACATCGTGGCCTT
TGCAGAGGAGGACGATCCTGACGGCTTCGAGTTCCTGGAGACCCTCAAGGAGGTGGCGCGGGACAACACCGATAACCCCGAACTCAGCA
TCATCTGGATCGACCCCGAGGACTTCCCGCTGCTCATCCCCTTCTGGGAGAAAACCTTCGGCATCGACCTCTCCCGGCCCCAAATCGGA
GTGGTCAATGTCACAGATGCTGACAGTGTGTGGCTGCCGATGGCGGATGAGGACGACCTTCCGGACGCGGCGGAGTTGGAGGAGTGGAT
CGAGGACGTTCTGGAGGGGGAGATCAGCACTGAGGACGATGATGATGATGATGCCGATGACGACGCCGATGACGATGATTAGGGTCACT
CCGTCCCTGTCCCCATCCCTGTGCGTGGCCATTAAAGTCCCCATAGAGCCACT

>CASQ1_Crossoptilon_crossoptilon_CDS
ATGTTGGGCTGCCGGTGGCTGCTGGCGCTGCTGGCGGTGGGGTTTCCCCCCCCGGGGGGGGCCGAGGAGGGGCTCCGCTTCCCCTCCCA
CGACGGAGCGCCGCGGGTTCTGTCCGTCACAGCCAGCAATTACCGCGCGGCGCTGCGGGGCTCCCCGGTGGTCGCGCTGCTCCTTCATC
GGCCCGGGGGGACGCGGAGGGAACGGGAGGAGCGCGTGCTCGAGCTGGCGGCGCAGGTGCTGGAGGACAAAGGAGTGGGGTTTGGCTTC
GTGGATGCGGAGAAGGATGTGGCCACGGCCCAGAAGTTGGGGATGACGGAGGAGAACAGCATCTACATCTTTAAGGGAGATGAGGTGAT
TGAGTACGACGGCGAGCTGTCAGCCGACACGCTGGTGGAATTCCTGCTGGATGTGCTGGAGGACCCTGTGGAGTTCATTGAGGGTGACC
ATGAGCTGCGTGCGTTCGAGAACATTGAGGAGGACCCCAAAGTCATCGGATACTTCAAGGGGGTGGAATCAGAGTATTTCAAAGCCTTC
TCGGCGGCAGCAGCAGAGTTCCACCCTTACATCCCCTTCTTTGCCACCTTTGACCCCAAGGTGGCCAGGAAACTGACCCTAAAGCTCAA
CGAGATCGACTTCTATGAACCCTTCATGGAGGAACCACTCACACTCCCCACACCGCCCCAAGGCAAAGAGGAGATCGCCGCCTTCGTGG
AAGAGCACAAGCGGGCAACACTGCGGAAGCTCAAACCTGAGAGTATGTATGAGACATGGGAGGATGACATTGATGGGATTCACATCGTG
GCCTTTGCAGAGGAGGACGATCCTGACGGCTTCGAGTTCCTGGAGACCCTCAAGGAGGTGGCGCGGGACAACACCGATAACCCCGAACT
CAGCATCATCTGGATCGACCCCGAGGACTTCCCGCTGCTCATCCCCTTCTGGGAGAAAACCTTCGGCATCGACCTCTCCCGGCCCCAA
ATCGGAGTGGTCAATGTCACAGATGCTGACAGTGTGTGGCTGCCGATGGCGGATGAGGACGACCTTCCGGACGCGGCGGAGTTGGAGG
AGTGGATCGAGGACGTTCTGGAGGGGGAGATCAGCACTGAGGACGATGATGATGATGATGCCGATGACGACGCCGATGACGATGATTA
G

>CASQ1_Crossoptilon_crossoptilon_prtotein
MLGCRWLLALLAVGFPPPGGAEEGLRFPSHDGAPRVLSVTASNYRAALRGSPVVALLLHRPGGTRREREERVLELAAQVLEDKGVGFGF
VDAEKDVATAQKLGMTEENSIYIFKGDEVIEYDGELSADTLVEFLLDVLEDPVEFIEGDHELRAFENIEEDPKVIGYFKGVESEYFKAF

SAAAAEFHPYIPFFATFDPKVARKLTLKLNEIDFYEPFMEEPLTLPTPPQGKEEIAAFVEEHKRATLRKLKPESMYETWEDDIDGIHIV
AFAEEDDPDGFEFLETLKEVARDNTDNPELSIIWIDPEDFPLLIPFWEKTFGIDLSRPQIGVVNVTDADSVWLPMADEDDLPDAAELEE
WIEDVLEGEISTEDDDDDDADDDADDDD*

>CASQ2_Crossoptilon_crossoptilon_transcript_assembly
ACTCACTCTCTCACTCTCAAATGATGCAGAGTGCCAGCAAGTCCCTCAATGCTGTCATATCTGTCCTTTTTATTCATTGCAGGGTTTAT
TTTTAGCCTGAAACAACTGCTTCCTAAAAATGGAGTTCCTAATGACACGGGAGCTGGACACAGCTATGTAAGGTATCCAGGGCTTGGCC
TGACAGCTTCTCCTGTCTGTCTCTTTGTTGTGAGCTCAGGACAGCAAAGTTTGTTGCTTGCCACCAGCCTTGCAGAGCTTGGACTGAAC
ACTAGGAAGGCAGCAGAAAGAGAAACTCGGTGCAGACGCCCACTGGAGCCCTTGGCCTCCTTCTCTTCCCAAACTGAGAAGGAGAGAGA
CCTTTGCCAGCGCTGCACTGAACTCCAAGGGTTCCCCAATGAAGGCGACTTGCTGGATCCTGGCAGGCTTTTGCCTGCTTTTCTGCTGC
AAGGCAGAAGAAGGACTGAATTTCCCCACGTATGATGGGAAAGACCGAGTGATCGACCTGAACGAGAAGAACTACAAGCATGCCCTGAA
GAAATATGACATGCTTTGTCTGCTCTTCCATGAGCCCGTGAGCTCTGACAGGGTGTCCCAGAAGCAGTTCCAGATGACGGAGATGGTCC
TGGAGCTGGCAGCTCAGGTCCTGGAGCCTAGGAGCATCGGCTTTGGGATGGTGGATTCCAAGAAGGATGCCAAACTCGCCAAAAAGCTA
GGCTTGGTTGAAGAGGGAAGTCTCTATGTGTTTAAGGAGGAGCGGTTGATTGAATTTGATGGGGAACTGGCCACAGATGTCTTGGTGGA
ATTCCTCTTGGATCTGCTAGAAGACCCTGTGGAAATCATAAACAGCAAGCTGGAGCTTCAAGCCTTTGACCAAATTGACGATGAAATCA
AACTGATTGGCTACTTCAAAGGAGAAGACTCTGAACATTACAAGGCGTTTGAAGAGGCTGCTGAACACTTCCAACCATACGTCAAATTC
TTTGCCACATTCGACAAAGGGGTTGCTAAGAAACTGGGTCTGAAGATGAACGAGGTGGACTTCTATGAACCATTCATGGATGATCCTGT
TCACATCCCTGATAAACCTTACACAGAAGAGGAGCTGGTTGAATTTGTGAAGGAGCACAAAAGAGCCACCTTGCGGAAGCTGCGCCCAG
AAGACATGTTTGAGACATGGGAAGATGACATGGAAGGAATCCATATCGTGGCCTTTGCTGAAGAAGATGACCCAGACGGCTTTGAGTTC
CTGGAAATCCTGAAGCAGGTTGCCAGGGACAACACTGATAATCCTGACCTGAGCATTGTGTGGATCGACCCTGACGACTTTCCTCTGCT
TATCACTTACTGGGAGAAGACCTTCAAGATTGACCTGTTCAGGCCGCAGATTGGGATTGTGAATGTCACAGACGCTGATAGTGTCTGGA
TGGAGATCAGAGACGATGATGACCTGCCCACAGCCGAGGAGCTGGAGGACTGGATAGAGGATGTGCTTTCTGGGAAGATAAATACTGAA
GATGATGATGATGACGATGACGATGATGATGACGACGATGACGATGATGATGATGACGATGATGATGACGACGATGATGACGACGACGA
TGATGATGACTAACTGTGACTCTGTACAGTTTGACTTGTGGAGGCCGAGAGGCCTCCCCCTGTGGCAGGTCCCTTCATCAGAAGACCTG
TGTTTGAGCGTCAGCAAGCACCGCCTGCTCCTCCTTTCCCCCTTGCCCCGCTCTCCCTGCCCCTGCTGGGACCCCCGGCCTGATTCTCA
GAGGTGCTGAGTCACCAGGACTGCCACCTTGGTGGGTGGCACCGGCAGGTGCCCAGCACCGCTGTAAATCATGCCTCCGTAGAAAGGAC
AATAGGAATCTGTAGGGAACCTGCCCCAGTGCCCAGTGCAGGAGGAAAGCACCTGCATGCTCACTGCCAGAGGGACACAGGGAGCCAGT
TTGTATTTTCCATGCTCATCGGCCCATCATGTACATCCAAAGACCAAATCTCACTCAATACAGAGGGAAGGTCACTGTAAAGTTTAGTC
CTTTCATTAAACAAAATGCCCTTTGCAGCTACTCTGCCAAGACCACTGGATCCTGAAAGTTCTGCTGTAGGAGTGTAAAGCTACTTCTT
AACATACTAGGAGCAGCAAAACATTTGAGAGGGGGAAGACAGTTTTAGGAACGCTAGTTAATGTTCCTAAAATAATTTTTAGTGATTCC
ATAAAATGACAGTATACTTGTATCTGACAGAGGATGCAAAACCTAACACCACAATCTGACATGCTCTCTTCAAGCAAAAAATAGAGTAA
GTGCAGGTGGTTTTAGATGGGACAGCACAGCTTGCGAGTTCTTGAGCTTGGAAGTTTCTGAATGCTGGAAAGGCTGATGCAGGTCTGAG
CCCAGACTCTCAAGACTGAAGATCACTGCACTGAGCCTGACTGTTGGTTCTTAATCTTGAGGAAACACTGGAACTGACCTCAGGCCAGT
TCCAAACATCTGCAGCCAGGTCCGAGCTGAGGTGGTGAATGTGAGATAGCACACTTCTGCCCATAGAAACCTTCATAGGGGCTCACCCT
GAAGCCTTCCTATTCCTCATGACCTTCCAGCTTTAGGTGACTTTAACCAAAATTCCATGTAGCATCTTTTCCCATGACCAAGGTGTTAG
GAGGGAACATATTCATCCAACCATACTAGATGCTGGATTAAGTGAGTTGGGCAATGAGCAATGAAAGCAATCAAGCTGGTCAACTTCTA
CATATTGTTGCAAATTCAGTCCCTGCGTAGAGACTTGGATGTCCCACGGTAGCCTGTGAGAAACCTCTTCCAGACTGAGGTGGAGTTCA
TGGCATCAGCTTGCAGCAAAGGGGGCCATGAGGTAAAGGGCCAGTGACTGAGTCCCAGTGAGTGCCACTCCATCCATGTGCAGGTCACT
TCAACAGCATTAGGTCTCCCTGCTTTCCCATGCCAGCAGATTTCAATTGTCTTAGCTTCTTAGGTTTGACAATAGTGCAATGAATGGAG
AGCTTGGAGATGCTTTATCTTTATAAATACTTTATAAATATATTTTCCAGTAATTCAGACTTGGTAGCAGAGATGGGATGTACATGTCC
TCTCCTGAAACAGTCAAAAAACATTGTTCTGGTCTTTCTTAAGAAAACTGGCTTAGAGATAAGTCAGAGATAAGTGATGTTAGTGGAAA
TAATAGTGCAAGGAACAAGAGATTCAGTGCACGCTGTATGAAAATGAATCAGCCTGACAAGAAACAAGGTTTACACACCATATATTTCC
TTCATTGGATAAGCTGCAAAGCAAGATTTTATAGATTTCTTTTTCTCCTTGATGTTGGTCAGTCTCTTTGTTTCTCTCTTTCCCTCTAT
ACTTGAGCTGAAATAGAAATTTGAAATTACTAGTATAAATCGGCTGAAGTACTGATCTACCTCTGTGTGTGCCATGTCCCCTCAGTGGG
AGGCGCCAGCATGAAAAACACTCAGCTGGATATCTACATATCTCTGTGAATGACTGCTGTGCCTATATGGTAGATGGCTGTAGGCCGTG
CTAGCTCCACATTGAGCATCCGACACACAACCAGCACTTTTCAGGCACTATCTATTAGCATGACAGGACAGCTACGTCCTCAAGTCGGG
TATTATCTGTGTTAGCCCTTTCTTATTTCTTTATGATGTATAAGCACTTTCTGGAAGACTTGAACCAAGTGTGTTTGGGGAGATCATCT
GGGTGTTCACACCAGTGTGCATAGCGGTGAACAGCTCAGCCCCAATCCAAAGGACCTGGTGGAGACTATGAGAAGGCTTGAAATGCAGT
GTGCTGTCAGACTGGAGGTCCTCTCTCCAGACCAGAAAGGTGCTCAGTGCTATCCCTCTGCTCTGCCCTTTTAGCTATTTCAGCCAGGA
GAGGGGGAACAAGCTGAACTCAAAGACTCATTTACGCACATTAGTAAAGAGCAAGAACAGGCTAAACTGGCAATTTAGTAGTCTCTTAG
TCTGTGATTAAGGGACAAACTCTGCTTATGTTTCACACCTGAGAATAATACTTGCTTATCTTTACCTTGGAGATATTTCCAGGAGGAAA
GCATGCAAGATTTCACTCTGCACATTGTAACTTCCTTAGCAGCTCTGCAGCCCCAGATCAGTCATTGCTAAGACCTGCCACTGTCGATA
AACATGAAGAATAAACAAAGACTTGCTCTTTCCCAGCCAGCACTGTCCCTGGACACCCCAGCTGCTTGGATACTTTCCTTGTAATACCA
CTGCTCCCAGACATCCTTCTAGTCTGATATGCCTCTGTCCATATGCTCCAATGGACATAAATTAAGCAGAAGTAATAGACCCTGCAAAC
AGCATTGCAGATGTGTTCAGGTGCACAGGTGGTGTTTAACCTTATACAGATACTAGAAAGACTGATCTCACACTGCAGCCAGTAGGCTG
GTGTTGGTGATTTATGGAGAATTCCCCCTGTATTATGAGAGAAAGGGCTGATCATATGGCTGCCACTCCTCAATGGGGTCAGCACAGTT
CTTGAAGGGGACTGCCCTTCTCTCTTCCCTCAGTCCCATGTTATGGCTTTCTTTAGTTCTCTTTGACCACAAAGGGAATCGTTTTATCT
CCAGCTTTTACTTACAAAGCCAGAAGTGCCCAGTGTCTTCTTTTGGGGTAAACCCATCAAAAATTAACAGGTGTTGCAGAAGAGGAAGG
ATTACAGCAATGAAGAACAGCAGGTAGCCAAGGGGCTCTCGTTTACCCACAGCACCAAGGTTCACAGGGACCTGAATAAGACAAAGCTC
CTCGTGAAGGAGCTGAAAGTTTGAATTTGAGTGTCTTCCTTCCAGGGACAGGTAAGATACCTGTGTGTTGTGGGTTACAGCTGCTGAGA

CAGGGAGAAACTCTGCACAAAACCATTGAAATGTTTTAATTTCCCAATACACTACTGATGTGGGTCCATACCCACACCTGCATACATTA
CTCTCCTCCATCTTATGATAGTTACATCCACAGCACGAGGAGGTGCAGTGGGCCGCACTCAGTGTTTCTGCAGGCAGGTCTATCGGATG
AGGGCAAAACACAGTTCATCATCTTCATGTGTATTAAGACCAATTTAATTGGGATGTCAGATTACCACTTCAGTGAAAATATATATTAG
ATCTATCAGAAGCACAGAGGAGGAAGCTTCTGCCATTGCAGCAGGCCTAAGGACATTGCTGGAGAGACTCTGTGAACCAGAACCATGTC
TGAGCTAATGTCAATCCCTTGGAAAAGTTTCTGAAGGAGAGGACAAGGGTGGCACAGCGAAGTCACAGGGTGCTTCATATTTTCATGGT
CAGCAGTTCTTCTGGTGTTTCTAGAATAAGGGTGGTCTCCCATGCTGGCAAATTGGACACCTTGGCTGTTTCCCATCCTGAAGAGGTAG
ATACTGGCTGCTTCTCTGTGTCTATCTACACATCAGAAATGATTCAGGGATTCATTACCAAGGCCAAACAATAAACATTGCCTTTTATT
CTCAATTAATTAAACAATTAATTAGACAGGAACATATCAATTCCTGGCTACTGAAGTCTTGCAGTCCTGCGAAAATGAGGCAGGATTAC
ATCTGGATCTCTCTCTGCCTCAGCAAAACTTCAGAAAAGCCCCATATAGGGCTAGTCACACACCAATGCCTGGACTTAGGGACCTGAAG
AGGGACGAGCAGTGAACAGGCAACAAAGAACCAAGAAAGGGCAATTATCCTGGGTTAATCTGGCTGTGCCAGCAGGACTGAGCCCTATC
TGAGACCCAAGGCTTGGTAACCACTGTGCTGCAGCATCTCCTGCCTATACCCCAGTTCCTCAAGCCTGGTACCCCTGACACCCAACCTA
GGCCAGCCCAGATCCAGGGGAACAACCCTTGCCAGGTCTAAAAGGTGCTGAAACTGTTAAATTAATGAATAAATAAAAAATTATTCTGC
TACTCTGCCTGAGCACCAGATGAGTGGCAAAGCTTCAGGCATAAGAAATAAGCCTCAAGCCCAAGAAATAAGGGTTAAACAAACAGATC
TTTAACAGAGTAATTATCCATACTTTAGCAGCAGCCAGAAAGCCAACCACCCACCTAAGAAGAGCAGCAGCCGTCCTGACTGTGCCCTT
CATTGGCAGTGGGTGGCAAATGTCTCAAACCATGGCCGTGGGCAAACTCCTGCTCTGCTTGGGCTGGGAGGAGAACCTGTCTGCCTGTG
AAACAAGTAACATGGGTAAAAATAAAACTACGCGTGACCAGTGAGATTGGCCTGATGTGGGATTTTGCTCTAAAATTCAAACCAGTGTG
CCAAAGCCCACTCCTGCTAGTGACAAAGGAAAAGGAGTTACTGAATGACAGAAGTAACCAAGTGTTTGAAGAACAGTTTAGCCCCTGCA
AAATGAAATCACTGAAGGAATTTGCAATGCTTCCCAGTCTAGTTACTGAATTACTAAGCCAGTTTCTCCCAACTACTTACAAAGCAAAA
TTGTAATCAACTGTAGGGTCTGGTATTATGAGTACTCAAGAATGAGCTTTTACAAAATAGAAGCTAAAAGATTTACAGATCTGTTTCAC
GTCTGTGGTGCAGATATAATCAGCTCCTTCTTGTTCCCTGTTTTAAAGCATGCTCACAGCATCTGACTTGTGCTAAAAGAAGGTCCAA
AGAGCTAAAAGTTCTGAGTGTCTCTTAAAAGTCCCACAGAGGCTGAATCAACTGTATTTTATCAAGAACTCCGTTCTCTCCACTCTTA
TGGAGTTGTTATTTATGTCGCCTTTTCACATGTTAAAAAGAAGGACTGCAGAAGTCCAAGCATTTAGAAGCCTGATTTTCACCTCCGG
AAAAATCAGGCACAAAATGTTATAATTCAAAGAAAAGCTTCATGTCTAGAGCAATCTATGGTGTACCTTCATGTTCCTAAGGATGCCG
G

>CASQ2_Crossoptilon_crossoptilon_CDS
ATGAAGGCGACTTGCTGGATCCTGGCAGGCTTTTGCCTGCTTTTCTGCTGCAAGGCAGAAGAAGGACTGAATTTCCCCACGTATGATGG
GAAAGACCGAGTGATCGACCTGAACGAGAAGAACTACAAGCATGCCCTGAAGAAATATGACATGCTTTGTCTGCTCTTCCATGAGCCCG
TGAGCTCTGACAGGGTGTCCCAGAAGCAGTTCCAGATGACGGAGATGGTCCTGGAGCTGGCAGCTCAGGTCCTGGAGCCTAGGAGCATC
GGCTTTGGGATGGTGGATTCCAAGAAGGATGCCAAACTCGCCAAAAAGCTAGGCTTGGTTGAAGAGGGAAGTCTCTATGTGTTTAAGGA
GGAGCGGTTGATTGAATTTGATGGGGAACTGGCCACAGATGTCTTGGTGGAATTCCTCTTGGATCTGCTAGAAGACCCTGTGGAAATCA
TAAACAGCAAGCTGGAGCTTCAAGCCTTTGACCAAATTGACGATGAAATCAAACTGATTGGCTACTTCAAAGGAGAAGACTCTGAACAT
TACAAGGCGTTTGAAGAGGCTGCTGAACACTTCCAACCATACGTCAAATTCTTTGCCACATTCGACAAAGGGGTTGCTAAGAAACTGGG
TCTGAAGATGAACGAGGTGGACTTCTATGAACCATTCATGGATGATCCTGTTCACATCCCTGATAAACCTTACACAGAAGAGGAGCTGG
TTGAATTTGTGAAGGAGCACAAAAGAGCCACCTTGCGGAAGCTGCGCCCAGAAGACATGTTTGAGACATGGGAAGATGACATGGAAGGA
ATCCATATCGTGGCCTTTGCTGAAGAAGATGACCCAGACGGCTTTGAGTTCCTGGAAATCCTGAAGCAGGTTGCCAGGGACAACACTGA
TAATCCTGACCTGAGCATTGTGTGGATCGACCCTGACGACTTTCCTCTGCTTATCACTTACTGGGAGAAGACCTTCAAGATTGACCTGT
TCAGGCCGCAGATTGGGATTGTGAATGTCACAGACGCTGATAGTGTCTGGATGGAGATCAGAGACGATGATGACCTGCCCACAGCCGAG
GAGCTGGAGGACTGGATAGAGGATGTGCTTTCTGGGAAGATAAATACTGAAGATGATGATGATGACGATGACGATGATGATGACGACGA
TGACGATGATGATGATGACGATGATGATGACGACGATGATGACGACGACGATGATGATGACTAA

>CASQ2_Crossoptilon_crossoptilon_protein
MKATCWILAGFCLLFCCKAEEGLNFPTYDGKDRVIDLNEKNYKHALKKYDMLCLLFHEPVSSDRVSQKQFQMTEMVLELAAQVLEPRSI
GFGMVDSKKDAKLAKKLGLVEEGSLYVFKEERLIEFDGELATDVLVEFLLDLLEDPVEIINSKLELQAFDQIDDEIKLIGYFKGEDSEH
YKAFEEAAEHFQPYVKFFATFDKGVAKKLGLKMNEVDFYEPFMDDPVHIPDKPYTEEELVEFVKEHKRATLRKLRPEDMFETWEDDMEG
IHIVAFAEEDDPDGFEFLEILKQVARDNTDNPDLSIVWIDPDDFPLLITYWEKTFKIDLFRPQIGIVNVTDADSVWMEIRDDDDLPTAE
ELEDWIEDVLSGKINTEDDDDDDDDDDDDDDDDDDDDDDDDDDDDDDDDD*

6.11 *Milvago chimachima* (yellow-headed caracara)

From: Trinity *de novo* assembly of SRR10853036. CASQ1 from TRINITY_DN2610_c0_g1_i2. CASQ2 from TRINITY_DN525_c0_g1_i1.

>CASQ1_Milvago_chimachima_transcript_assembly
CAGGGCGGCCGGGCGGCCCAGCGCCACCGCGAGATGGAGGAGCTCATCCTGGAGCTGGCAGCCCAGGTGCTGGAGGACAAGGGGGTGGG
CTTCGGCTTGGTTGACTCTGAGAAGGATGCAGCTGTGGCCAAAAAGCTGGGCCTGACGGAGGAGGACAGCATCTACGTGTTCAAGGAAG
ACGAAGTGATCGAGTACGACGGGGAGCTGGCAGCGGACACACTGGTGGAATTCCTGCTGGATGTGCTGGAGGACCCGGTAGAGTTCATC
GAGGGCGACCATGAGCTCCAGGCCTTTGAGAACATCGAGGATGACCCCAAACTCATCGGCTACTTCAAGAACGAGGACTCAGAGCACTT
CAAGGCTTTTGAGGAGGCGGCAGAGGAGTTTCACCCCTACATCGCCTTCTTTGCCACCTTTGACAGCAAGGTCGCCAAGAAGCTGACGC
TGAAGCTGAACGAGATCGATTTCTATGAGCCCTTCATGGAGGAGCCGCTGACCATCCCCGAGCGGCCCAACAGCAAGGAGGAGATCGTG
GCCTTCGTGGAGGAGCACAAGCGGGCCACTCTCCGGAAACTCAAACCCAAGAGCATGTATGAGACCTGGGAAGATGACATGGATGGGAT
CCACATTGTGGCCTTCGCAGAGGAGGATGATCCGGATGGGTTTGAGTTCTTGGAGATCCTGAAGGATGTGGCCCGGGACAACACGGACA

ACCCTGACCTCAGCATCCTCTGGATCGACCCCGAGGATTTCCCACTGCTCATCCCTTACTGGGAGAAAACCTTCAACATCGACCTGTCC
CGGCCCCAGATCGGGGTCGTCAACGTCACCGATGCCGACAGCGTGTGGCTGGAGATGGCGGACGAGGACGACCTGCCCGGGCCGGAGGA
GCTGGAGGAGTGGATCGAGGATGTGCTGGCAGGAGAGATCAACACCGAGGACGACGATGACGACAATGACGAGGACGACGAGGACGATG
AGGGTGATGAGGACGACTAGGCCTCGCGCTGGGCCTCCGCTCCGTCCCCGTCCCCATCCCTGTGCGCGCCATTAAAGTCCCCGAGAGCC
AGG

>CASQ1_Milvago_chimachima_CDS_partial
CAGGGCGGCCGGGCGGCCCAGCGCCACCGCGAGATGGAGGAGCTCATCCTGGAGCTGGCAGCCCAGGTGCTGGAGGACAAGGGGGTGGG
CTTCGGCTTGGTTGACTCTGAGAAGGATGCAGCTGTGGCCAAAAAGCTGGGCCTGACGGAGGAGGACAGCATCTACGTGTTCAAGGAAG
ACGAAGTGATCGAGTACGACGGGGAGCTGGCAGCGGACACACTGGTGGAATTCCTGCTGGATGTGCTGGAGGACCCGGTAGAGTTCATC
GAGGGCGACCATGAGCTCCAGGCCTTTGAGAACATCGAGGATGACCCCAAACTCATCGGCTACTTCAAGAACGAGGACTCAGAGCACTT
CAAGGCTTTTGAGGAGGCGGCAGAGGAGTTTCACCCCTACATCGCCTTCTTTGCCACCTTTGACAGCAAGGTCGCCAAGAAGCTGACGC
TGAAGCTGAACGAGATCGATTTCTATGAGCCCTTCATGGAGGAGCCGCTGACCATCCCCGAGCGGCCCAACAGCAAGGAGGAGATCGTG
GCCTTCGTGGAGGAGCACAAGCGGGCCACTCTCCGGAAACTCAAACCCAAGAGCATGTATGAGACCTGGGAAGATGACATGGATGGGAT
CCACATTGTGGCCTTCGCAGAGGAGGATGATCCGGATGGGTTTGAGTTCTTGGAGATCCTGAAGGATGTGGCCCGGGACAACACGGACA
ACCCTGACCTCAGCATCCTCTGGATCGACCCCGAGGATTTCCCACTGCTCATCCCTTACTGGGAGAAAACCTTCAACATCGACCTGTCC
CGGCCCCAGATCGGGGTCGTCAACGTCACCGATGCCGACAGCGTGTGGCTGGAGATGGCGGACGAGGACGACCTGCCCGGGCCGGAGGA
GCTGGAGGAGTGGATCGAGGATGTGCTGGCAGGAGAGATCAACACCGAGGACGACGATGACGACAATGACGAGGACGACGAGGACGATG
AGGGTGATGAGGACGACTAG

>CASQ1_Milvago_chimachima_protein_partial
QGGRAAQRHREMEELILELAAQVLEDKGVGFGLVDSEKDAAVAKKLGLTEEDSIYVFKEDEVIEYDGELAADTLVEFLLDVLEDPVEFI
EGDHELQAFENIEDDPKLIGYFKNEDSEHFKAFEEAAEEFHPYIAFFATFDSKVAKKLTLKLNEIDFYEPFMEEPLTIPERPNSKEEIV
AFVEEHKRATLRKLKPKSMYETWEDDMDGIHIVAFAEEDDPDGFEFLEILKDVARDNTDNPDLSILWIDPEDFPLLIPYWEKTFNIDLS
RPQIGVVNVTDADSVWLEMADEDDLPGPEELEEWIEDVLAGEINTEDDDDDNDEDDEDDEGDEDD*

>CASQ2_Milvago_chimachima_transcript_assembly
ATCCAGGGCTTGGCCAGACAGCTTCTCCTGTCTGTCTCTTTGTTGTGAGCCCAGGACAGCACAGGCTGCTGCTCCCCACCAGCCCTGCA
GCGTTTGCACTGATCACTAGGAAGGCAGCAGAAAGAGAAACTCGGTTCGAACTTCACCTGGACCCTTTCGTCTCCCCCTCTTCTCGAAT
TCAGAAGGAGGGGGACGTTTACCTGCCCTGCGCTGCGCACCAAGGGCTCCCCTATGAAGGCGACTTGCTGGATCCTGGCAGGTTTTTAC
CTGCTTTTCTGCTGCAAGGCAGAAGAGGGACTGAACTTCCCCACTTACGATGGGAAAGACCGAGTGATCGACCTGAACGAGAAGAACTA
CAAGCAGGCCCTGAAGAAGTATGACATGCTCTGCCTGCTCTTCCATGAGCCTGTGAGCTCGGACAAAGTCTCCCAGAAGCAGTTCCAGA
TGACTGAGATGGTCCTGGAGCTGGCAGCTCAGGTCCTGGAGCCCAGAAGAATTGGCTTCGGGATGGTGGACTCCAAGAAGGATGCCAAA
CTTGCCAAAAAGTTAGGCTTGGTTGAAGAGGGAAGTCTCTATGTCTTTAAGGAGGAGCGGTTGATTGAATTTGATGGGGAACTGGCCAC
AGATGTCTTGGTGGAATTCCTCTTGGATCTGCTAGAAGACCCTGTGGAGATCATAAACAACAAGCTGGAGCTCCAGGCCTTTGACCAAA
TCGACGAGGAAATCAAACTCATCGGCTACTTCAAAGGAGAAGACTCTGAACATTACAAGGCATTTGAAGAAGCTGCTGAACACTTCCAG
CCATACATCAAATTCTTTGCTACCTTCGACAAAGGGGTTGCCAAGAAGCTAGGTCTGAAGATGAATGAGGTGGACTTCTATGAACCGTT
TATGGACGAGCCTGTTCACATCCCCGATAAGCCTTACACAGAAGAGGAGCTGGTTGAATTTGTGAAGGAGCACAAAAGGGCCACCTTGC
GGAAGCTGCGCCCAGAGGACATGTTTGAGACGTGGGAGGACGACATGGAGGGAATCCACATCGTAGCCTTTGCTGAAGAAGACGACCCA
GATGGTTTTGAGTTCCTGGAAATCCTGAAGCAGGTTGCCAGGGACAACACCGATAATCCCGACCTGAGCATTGTCTGGATTGACCCCGA
CGACTTTCCTCTGCTGATCACTTACTGGGAGAAGACCTTCAAGATTGACCTGTTCAGACCACAGATTGGAGTGGTGAACGTCACAGACG
CTGACAGCATCTGGATGGAGATCAGAGATGATGATGACCTGCCCACAGCCGAGGAGCTGGAGGACTGGATAGAGGATGTGCTTTCTGGG
AAGATAAATACTGAAGATGATGATGACGACGATGACGATGATGACGACGATGACGATGACGACGACGATGACGATGACTAACTGTGATT
CTGTGCTGTTTGACTTGTGGGGACCGAGAGCCTCCCCCCAGCGGCAGGTCCCTCCATCGGAAGACCTGTGTTTGAGCGTCAGCAAGCAC
CGCTGCTCCTCTTTCCCCCCTTGCCCCGCTCTCCCTGCCCCTGCCATTGCTGGGACCCCCTGGGCCTGATTCTCAGTGGTGCTGAGCGA
CCAGGACTGCCTGCAGGCACCCAGCACCGCTGCAAATCATGCCTCCTTAGTAAGGACAATAGGAATCTGCAGGGAATCTGCCCCGAGTG
TCCCAGGGCAGGAGAAAAGTGTCTGCCTGCTTGCTGCCAGAGGGACACAGGGAGCCAATTTGTATTTTCCACGCTCATCAGCCCAGCAC
TTAACATCCAAAGACCAAATCTCCCTTCAAGACATAGGGAAGGCGACTGTAAAGTTCAGTCCTTTTATTAAACACAATGCCCTTTGCAG
CTACTCTGCCAAGACCACTCAATCCTGCAAGTTCTGCTGCGGGAGTCTGTAGCTACTCATTAACATGCTAGGAGCAGCAAAAAGCTTTG
GGAGGGGAGGAAGGTTTTAGGGATGCTATTTAAATTTTCAGTGATACCGTATAACAACAGGATATTCTTGTCTGGCAGCGCAAGGCAAA
ACCAAACACCACAGTCCCTGTCTGAACCTCTTTCTTCTTGAAGGCTCAGGCCAAAGCTAGGGTAAGTGCAGGTGGTGGGATATAGGACA
TCAAAGCTTGTGAGTTCCTGAACTGCTAGAAAGGCTGATCCAGGTCTGAGCCCAAACTCTGCGGCTGAAGATCATGGCACTGAGCCAAA
CCATTGGTCTTAACCCCAAAGTAACACCAGAGCAAACTCCAGGCCAGCTGCAAACATCTGCAGCCAGGTCTGAGCTGGGCCGACCTTCT
CTTTTAGGGGAACCTCAGCTGGATGGAAGGTGGTGCACACGAGACAGACAGCATACTCCTGCCAGTGGAAACATTCATAGGGGCCCACC
CCTGAAGCCCTCCTATTCCCCATGACCTCCCAGCTTTAGCTGACTCTTAACCCAAAGCCTGCAGAGCATCTTTTCCCATGACCAAGATG
TTGGGAGGGAACACATCCGTCCAACCATATCAGATGCTGGGTAACGTGCGTCGGGCAATGAGCAGCAAAAGGATGCAAGCTGTTTGGCT
TTTAAGTATTCAGTTGCACAATTACTCCCTGCTTAGAGACTCAGATGTCCCGTGGTAGCCCGTGAGAAGCCTCTTTTGACTGCAGATGG
AGAAAGAGCTCAGTCAAGGGGGTCACTCACAAAGCAAAGGGTCAGCAACTCAGTCCTGGCGAGTGCAGCACCATCCATGTGCAGGTCAC
TGCGACAGCGTTAGGTCTCCCTGCTTCCCCGTGCCAGCAGATTTCAATTGTCTTAGCTTATTAGGTTTGACAATAGTGCAATGAATGGA
GAGTTTGGAGGTAGTTTTATCTTTATAAATACATTTATAAATATGTTCTCCAGTCGTTCAGACTTGTTAGCATAGGTAGGGTACACATG
TCCTCTCTTGCTACAGACGAAAAACATTGTTCTGACCTACCTTATACTGGCTTATGTCAGAGGTAAGTGATGTTAGGGGAAATAAAAGT

ACGAGGGACAGGAGAATCAGAGCTCGTTATACAAATGTGAATCAGCCTGATCAGAAACAAGGTTTACACACTATATATTTCCTTTATCA
GATGAACTGCAAAGCAAGATTTTATAGATTTTTTTTTTTCCTGCCTGTTGATCAGTCTCTGTGACTCTCTCTCTACGTTAGCTGAAGTA
GGAATTTGAAACTACTGGTATAAAAAAGCTGAAGTACTGATATGTTCCTATGTGTGCCATCTCCCCTTGGTGGAAAGTGCCAGCACAAA
CAACACACAGCTGGATATCTGTGCATCTCTATGTATATGAGTTAATGTGCTTATACGATATATGACCATAGGCCATGTTAGCTTCTCAT
GGAACATCAAACACTCAACCCATGCTTCTAAGGCACAGCCTGTGAGAAGAAAAGACAGGACAGCAAAGCCCCCAGGTCAACTGTTTGCC
CTTTCTTGCTTCTTTACGATGCACAAGTACTGCCTGGAAGACTCGAACTAAGTGTGTTTGGGGAGAGCATCCACCTGGTCACGCTGGGG
TTATGAACAACTCAACCCCATCCCAGAGGACCTGGTGGAGGCTTTCAAAAGCCTTGAAATGCAGCGTGTGCTCTCAGCCTAGAGGTCTC
CTCCTCTCTTCCACTCACACCCTGCGCTCTCCTGGCCAGAGGGGTGCCCAGCACCCGGCTTCTGCTCTTTCCTTTCAGCTGTTTCCTCA
AGAGCTTTGCTGGAGGACCCCAGCTCAGAGGGAGGGAGCAAGCAGCACTCAGGGTCTCATTGATGTACATCAGTAAGGAGCAAGAACAG
GCAAAACCAGCCCTTCGGTAGTCTCCTAGTCTGTGGTTAAGGGACAAATGCTGACCTGAGAAAAGTACTTGTTTATCTCGACCTTGGAG
CTATTCCCAGGAGTAAAACATGCACAGTTTCACCCTGCGTAAGGTAACAGCCTGGCCAGACCCTGCAACTCCAGATCAGTCACTGCAAA
GTGCTGTGAATTTCTAGAAACCTAAAGAATAAAGGAAGAGTTGCTCTAACTGGCATTGTCTCCGGACATCCCGATTGCCTGGGTACTTT
CCATGTACCTCTCCTCCCGCTGGGGATGCCCATCTGTTCTGATGTGCCCCTCTCTGGATGCTCCGAGAGGCACAGCTCACACAGGCAGA
CCCTGCAAACAGCACTACCTACGTGTTCAAGTGCACACGTGTTTCACAGGCACGCACATCTTCCAATGCGTCAAGCAACAGAGCTCTGG
TGGGGGAAAATAATTCATGTAGTGGTG

>CASQ2_Milvago_chimachima_CDS
ATGAAGGCGACTTGCTGGATCCTGGCAGGTTTTTACCTGCTTTTCTGCTGCAAGGCAGAAGAGGGACTGAACTTCCCCACTTACGATGG
GAAAGACCGAGTGATCGACCTGAACGAGAAGAACTACAAGCAGGCCCTGAAGAAGTATGACATGCTCTGCCTGCTCTTCCATGAGCCTG
TGAGCTCGGACAAAGTCTCCCAGAAGCAGTTCCAGATGACTGAGATGGTCCTGGAGCTGGCAGCTCAGGTCCTGGAGCCCAGAAGAATT
GGCTTCGGGATGGTGGACTCCAAGAAGGATGCCAAACTTGCCAAAAAGTTAGGCTTGGTTGAAGAGGGAAGTCTCTATGTCTTTAAGGA
GGAGCGGTTGATTGAATTTGATGGGGAACTGGCCACAGATGTCTTGGTGGAATTCCTCTTGGATCTGCTAGAAGACCCTGTGGAGATCA
TAAACAACAAGCTGGAGCTCCAGGCCTTTGACCAAATCGACGAGGAAATCAAACTCATCGGCTACTTCAAAGGAGAAGACTCTGAACAT
TACAAGGCATTTGAAGAAGCTGCTGAACACTTCCAGCCATACATCAAATTCTTTGCTACCTTCGACAAAGGGGTTGCCAAGAAGCTAGG
TCTGAAGATGAATGAGGTGGACTTCTATGAACCGTTTATGGACGAGCCTGTTCACATCCCCGATAAGCCTTACACAGAAGAGGAGCTGG
TTGAATTTGTGAAGGAGCACAAAAGGGCCACCTTGCGGAAGCTGCGCCCAGAGGACATGTTTGAGACGTGGGAGGACGACATGGAGGGA
ATCCACATCGTAGCCTTTGCTGAAGAAGACGACCCAGATGGTTTTGAGTTCCTGGAAATCCTGAAGCAGGTTGCCAGGGACAACACCGA
TAATCCCGACCTGAGCATTGTCTGGATTGACCCCGACGACTTTCCTCTGCTGATCACTTACTGGGAGAAGACCTTCAAGATTGACCTGT
TCAGACCACAGATTGGAGTGGTGAACGTCACAGACGCTGACAGCATCTGGATGGAGATCAGAGATGATGATGACCTGCCCACAGCCGAG
GAGCTGGAGGACTGGATAGAGGATGTGCTTTCTGGGAAGATAAATACTGAAGATGATGATGACGACGATGACGATGATGACGACGATGA
CGATGACGACGACGATGACGATGACTAA

>CASQ2_Milvago_chimachima_protein
MKATCWILAGFYLLFCCKAEEGLNFPTYDGKDRVIDLNEKNYKQALKKYDMLCLLFHEPVSSDKVSQKQFQMTEMVLELAAQVLEPRRI
GFGMVDSKKDAKLAKKLGLVEEGSLYVFKEERLIEFDGELATDVLVEFLLDLLEDPVEIINNKLELQAFDQIDEEIKLIGYFKGEDSEH
YKAFEEAAEHFQPYIKFFATFDKGVAKKLGLKMNEVDFYEPFMDEPVHIPDKPYTEEELVEFVKEHKRATLRKLRPEDMFETWEDDMEG
IHIVAFAEEDDPDGFEFLEILKQVARDNTDNPDLSIVWIDPDDFPLLITYWEKTFKIDLFRPQIGVVNVTDADSIWMEIRDDDDLPTAE
ELEDWIEDVLSGKINTEDDDDDDDDDDDDDDDDDDDDDD*

6.12 *Netta rufina* (red-crested pochard)

From: Trinity *de novo* assembly of ERR12507423

>CASQ1_Netta_rufina_transcript_assembly
-CCCCCCCCCCCCCCAACTCTCTCCCGGGGGGGGGGCCACCAAGGGGCTGCGTGGCCGCTGCCCCCCCCCTCCCCTCCCACCCCCATCC
CAGGCGCTGGGGCTGCGGGTCCCGGCCGGGCCGCGTCCGTCTGTCCGTGTGTCCGTGTGTCCGTCCGTCCATCCGTCTGTCTGTCCGTC
TGCCTGTCCGTGTGTCCGTCTGTCCCCTCTGTCCCCAAGTGTCCGGGTGTCCCCCTCCGGCTGCCACCCGGGCCGGGGGCTGTCTGTCT
GTCCGTCCGTCCGTCCGCCTGTCCGTGTGTCCGACCCCCTGTGCTGTCCGTCCGTCTGTCCGTCCGCCTGTTCCCTGTCCGTGTGTCCG
TCCCCGTGCCGGCTGTCTGTCCGTCCGGCTCTCCTTTTCCGTGTGCTGTCTGTCCCGGTGCTGTCCGTCCGTCTGTCCGTCCCCGTTCT
GTCCGTCCGTCTGTCTGTCCCCTTGCTGTCCATCCGTCCGTCTGTCCGTCCCCAGGTTGCCTGTCCCCGTGCCGGCTGTCTGTCCGTCC
AGCTGTCCGTCTGTCCGTCCCTGTGCTATCCGTCTGTCCATCTGTCCGTCCCCGTCCTGTCTGTCCGTCCGTCCGTCCATCCATCCGTC
CGTCTGTCCCCGTGCTCTCCGTCCATCCGTCTGTCCATCCGTCCCCCCCCCGCAGCTCCCTCCTCGTCCCTTCCTCCCCGCCCCCCCCC
CGACCCCCATGCGGGTCTGGGGGCTGCTCCTGGCCCTGCTGGCCCTGGGGGTGGCGGCCGGGGGGGGGTCCCGGGGGGGTCTCCGCTTC
CCCACCCACGACGGCTCCGGGCGGCTGCTGCCGGTGCCGGCCCGGGACCCCCCCGCGGCGCTCGGGCGCTTCCCGGTGCTGGCCCTGCT
CTACCACGGCCCCCCCGCGGGGAACGAGGCTCAGCGCCGCCTGCACACGGGGGAGCTCGTCCTGGAGCTGGCAGCCCAGGTGCTGGAGG
ACAGGGGCGTGGGGTTCGGCCTCGTGGACGCCCAGAAGGAAGCGGCCGTGGCCGAGAAGCTGGGGCTGACGGAGGAGAACAGCATCTAC
ATCTTCAAGGGGGACAAGGTGATCGAGTACGACGGGGAGCTGGCGGCCGACACGCTGGTGGAGTTCCTGCTGGATGTGCTGGAGGAGCC
GGTGGAGTTCATTGAGGGCGACCGCGAGCTCCAGGCCTTCGAGAACATCGAGGAGGACCCCAAGGTCATCGGCTACTTCGAGGGCAAGG
ACTCAGAGTACTTCAAGGCTTTCGAGGAGGCGGCGGCGCAGTTCCACCCCTACGTCCCCTTCTTCGCCACCTTTGACGCCAAGGTGGCC
AAGAAGCTGACGCTGAAGCTGAACGAGGTCGACTTCTACGAGCCCTTCATGGAGGAGCCGCTCACCCTCCCCGCCCGGCCCCCCGGCAC
CGAGGACATCGTGGCCTTCGTGGGGGGGCACCGGCGGGCGACGCTGCGGAAGCTCAAACCCAAGAGCATGTACGAGACATGGAGGATG
ACATCAATGGGATCCACATCGTGGCCTTCGCAGAGGAGGACGATCCGGATGGGTTCGAGTTCCTGGAGATCCTGAAGGACCTGGCCCGG

GACAACACCGACAACCCTGACCTCAGCATCATCTTGATTGACCCCGAGGACTTCCCGCTGCTCATCCCTTACTGGGAGAAAACCTTCAA
CATCGACCTGTCCCGGCCCCAGATCGGGGTGGTCAATGTCACCGATGCGGACAGCGTGTGGCTGGAGATGGACGAGGACGACCTGCCGG
GAGCCGAGGAGCTGCAGGAGTGGATCCAGGACGTGCTGGAGGGAGAGATCAGCACCGGGGACGATGATGAGGATGACGACGATGACGAT
GATGAAGATGAAGATGATGAAGATGACGATGAAGATGACGATGATGATGATGACGATGATGAAGATGATGATGATGATGATGAAGATGA
TGATGATGATGATGAAGATGATGATGATGA

>CASQ1_Netta_rufina_CDS_partial
ATGCGGGTCTGGGGGCTGCTCCTGGCCCTGCTGGCCCTGGGGGTGGCGGCCGGGGGGGGGTCCCGGGGGGGTCTCCGCTTCCCCACCCA
CGACGGCTCCGGGCGGCTGCTGCCGGTGCCGGCCCGGGACCCCCCCGCGGCGCTCGGGCGCTTCCCGGTGCTGGCCCTGCTCTACCACG
GCCCCCCCGCGGGGAACGAGGCTCAGCGCCGCCTGCACACGGGGGAGCTCGTCCTGGAGCTGGCAGCCCAGGTGCTGGAGGACAGGGGC
GTGGGGTTCGGCCTCGTGGACGCCCAGAAGGAAGCGGCCGTGGCCGAGAAGCTGGGGCTGACGGAGGAGAACAGCATCTACATCTTCAA
GGGGGACAAGGTGATCGAGTACGACGGGGAGCTGGCGGCCGACACGCTGGTGGAGTTCCTGCTGGATGTGCTGGAGGAGCCGGTGGAGT
TCATTGAGGGCGACCGCGAGCTCCAGGCCTTCGAGAACATCGAGGAGGACCCCAAGGTCATCGGCTACTTCGAGGGCAAGGACTCAGAG
TACTTCAAGGCTTTCGAGGAGGCGGCGGCGCAGTTCCACCCCTACGTCCCCTTCTTCGCCACCTTTGACGCCAAGGTGGCCAAGAAGCT
GACGCTGAAGCTGAACGAGGTCGACTTCTACGAGCCCTTCATGGAGGAGCCGCTCACCCTCCCCGCCCGGCCCCCCGGCACCGAGGACA
TCGTGGCCTTCGTGGGGGGGCACCGGCGGGCGACGCTGCGGAAGCTCAAACCCAAGAGCATGTACGAGACATGGGAGGATGACATCAAT
GGGATCCACATCGTGGCCTTCGCAGAGGAGGACGATCCGGATGGGTTCGAGTTCCTGGAGATCCTGAAGGACCTGGCCCGGGACAACAC
CGACAACCCTGACCTCAGCATCATCTTGATTGACCCCGAGGACTTCCCGCTGCTCATCCCTTACTGGGAGAAAACCTTCAACATCGACC
TGTCCCGGCCCCAGATCGGGGTGGTCAATGTCACCGATGCGGACAGCGTGTGGCTGGAGATGGACGAGGACGACCTGCCGGGAGCCGAG
GAGCTGCAGGAGTGGATCCAGGACGTGCTGGAGGGAGAGATCAGCACCGGGGACGATGATGAGGATGACGACGATGACGATGATGAAGA
TGAAGATGATGAAGATGACGATGAAGATGACGATGATGATGATGACGATGATGAAGATGATGATGATGATGATGAAGATGATGATGATG
ATGATGAAGATGATGATGATGA

>CASQ1_Netta_rufina_protein_partial
MRVWGLLLALLALGVAAGGGSRGGLRFPTHDGSGRLLPVPARDPPAALGRFPVLALLYHGPPAGNEAQRRLHTGELVLELAAQVLEDRG
VGFGLVDAQKEAAVAEKLGLTEENSIYIFKGDKVIEYDGELAADTLVEFLLDVLEEPVEFIEGDRELQAFENIEEDPKVIGYFEGKDSE
YFKAFEEAAAQFHPYVPFFATFDAKVAKKLTLKLNEVDFYEPFMEEPLTLPARPPGTEDIVAFVGGHRRATLRKLKPKSMYETWEDDIN
GIHIVAFAEEDDPDGFEFLEILKDLARDNTDNPDLSIILIDPEDFPLLIPYWEKTFNIDLSRPQIGVVNVTDADSVWLEMDEDDLPGAE
ELQEWIQDVLEGEISTGDDDEDDDDDDDEDEDDEDDDEDDDDDDDDDEDDDDDDEDDDDDDDEDDDDX

>CASQ2_Netta_rufina_transcript_assembly
GTCCTCCCAGCATCGGCATCCTCCACTCCCTCCCGCACTCCCTCTCACTCACTCCCTCGCTCTCAGATGATGCAGAGTGCCAGCAAGTC
CCTCAATGGTGTCATATCTGTCCTTTTTATTCATTGCAGGGTTTATTTTTAGCCTGAAACAACTGCTTCCTAAAAATGGAGTTCCTAAT
GACACGGGAGCCGGACACAGCTATGTAAGGTATCCAGGGCTTGGCCTGACAGCTTCTCCTGTCTGTCTTTGTTGTGAGCCCAGGACAGC
AAAGTTTGCTGCTTCCCAACTGCCTTGCAGAGTTTGGACTGAACACTAGGAAGGCAGCAGAAAGAGAAACTCGGTTCAAACTTCCCCTG
GAGCCCTCGGCCTCCTTCTCTTTCCAAAGGGAGAAGGAGAGAGACCTTTGCCTGCCCTCCGCTGCACTCCCAGGGCTCCCCCATGAAGG
CGACTTGCTGGATTTTGGCAGGCTTTTGCCTGCTTTTCTGCTGCAAGGCAGAAGAGGGACTGAATTTCCCCACCTATGATGGGAAAGAC
CGAGTGATCGACCTGAACGAGAAGAACTACAAGCAGGCCCTGAAGAAGTACGACATGCTCTGCCTGCTCTTCCACGAGCCCGTGAGCTC
CGACAAGGTCTCCCAGAAGCAGTTCCAGATGACGGAGATGGTCCTGGAGCTGGCAGCTCAGGTCCTGGAGCCCAGGAGCATTGGGTTTG
GGATGGTGGACTCCAAGAAGGATGCCAAACTCGCCAAAAAGCTAGGCTTGGTTGAAGAGGGAAGCCTCTACGTCTTTAAGGAGGAGCGG
TTGATTGAATTTGATGGGGAGCTGGCCACAGATGTCTTGGTGGAATTCCTCTTGGATCTGCTAGAAGACCCCGTGGAGATCATAAACAG
CAAGCTGGAGCTTCAGGCCTTTGACCAAATTGACGAGGAAATCAAACTCATTGGCTACTTCAAAGGAGAAGACTCTGAACATTACAAGG
CATTTGAGGAAGCTGCTGAACACTTCCAGCCCTACATCAAATTCTTTGCCACCTTTGACAAAGGGGTCGCCAAGAAGCTAGGCCTGAAG
ATGAACGAGGTGGACTTCTATGAACCGTTCATGGATGAGCCTGTTCACATCCCTGATAAACCTTACACGGAAGAGGAGCTGGTTGAATT
CGTGAAGGAGCACAAAAGAGCCACCTTGCGGAAGCTGCGTCCAGAAGACATGTTTGAGACGTGGGAGGATGACATGGAAGGAATCCATA
TCGTGGCCTTTGCTGAAGAAGATGATCCAGACGGCTTTGAGTTCCTGGAAATCCTGAAGCAGGTTGCCAGGGACAACACCGATAATCCC
GACCTGAGCATTGTGTGGATTGACCCCGACGACTTTCCTCTGCTGATCACTTACTGGGAGAAGACCTTCAAGATTGACCTGTTCAGGCC
ACAGATCGGCGTGGTGAACGTCACAGACGCTGACAGCGTCTGGATGGAGATCAGAGACGATGACGACCTGCCCTCGGCCGAGGAGCTGG
AGGACTGGATAGAGGACGTGCTTTCTGGGAAGATAAATACTGAAGACGATGACGACGACGATGATGATGATGACGACGACGACGATGAT
GACGACGACGATGACGACGATGATGATGACGATGATGACGACGACGATGATGACGACGACGATGACGACGATGATGATGACGACGACGA
TGATGATGACGACTAATTGTGACTCTGTACAGTTTGACTTGTGGGGGCTGAGAGGCCTCCCCCCGCGGCAGGTCCCTCTGTTGGAAGAC
CTGT

>CASQ2_Netta_rufina_CDS
ATGAAGGCGACTTGCTGGATTTTGGCAGGCTTTTGCCTGCTTTTCTGCTGCAAGGCAGAAGAGGGACTGAATTTCCCCACCTATGATGG
GAAAGACCGAGTGATCGACCTGAACGAGAAGAACTACAAGCAGGCCCTGAAGAAGTACGACATGCTCTGCCTGCTCTTCCACGAGCCCG
TGAGCTCCGACAAGGTCTCCCAGAAGCAGTTCCAGATGACGGAGATGGTCCTGGAGCTGGCAGCTCAGGTCCTGGAGCCCAGGAGCATT
GGGTTTGGGATGGTGGACTCCAAGAAGGATGCCAAACTCGCCAAAAAGCTAGGCTTGGTTGAAGAGGGAAGCCTCTACGTCTTTAAGGA
GGAGCGGTTGATTGAATTTGATGGGGAGCTGGCCACAGATGTCTTGGTGGAATTCCTCTTGGATCTGCTAGAAGACCCCGTGGAGATCA
TAAACAGCAAGCTGGAGCTTCAGGCCTTTGACCAAATTGACGAGGAAATCAAACTCATTGGCTACTTCAAAGGAGAAGACTCTGAACAT
TACAAGGCATTTGAGGAAGCTGCTGAACACTTCCAGCCCTACATCAAATTCTTTGCCACCTTTGACAAAGGGGTCGCCAAGAAGCTAGG
CCTGAAGATGAACGAGGTGGACTTCTATGAACCGTTCATGGATGAGCCTGTTCACATCCCTGATAAACCTTACACGGAAGAGGAGCTGG

TTGAATTCGTGAAGGAGCACAAAAGAGCCACCTTGCGGAAGCTGCGTCCAGAAGACATGTTTGAGACGTGGGAGGATGACATGGAAGGA
ATCCATATCGTGGCCTTTGCTGAAGAAGATGATCCAGACGGCTTTGAGTTCCTGGAAATCCTGAAGCAGGTTGCCAGGGACAACACCGA
TAATCCCGACCTGAGCATTGTGTGGATTGACCCCGACGACTTTCCTCTGCTGATCACTTACTGGGAGAAGACCTTCAAGATTGACCTGT
TCAGGCCACAGATCGGCGTGGTGAACGTCACAGACGCTGACAGCGTCTGGATGGAGATCAGAGACGATGACGACCTGCCCTCGGCCGAG
GAGCTGGAGGACTGGATAGAGGACGTGCTTTCTGGGAAGATAAATACTGAAGACGATGACGACGACGATGATGATGATGACGACGACGA
CGATGATGACGACGACGATGACGACGATGATGATGACGATGATGACGACGACGATGATGACGACGACGATGACGACGATGATGATGACG
ACGACGATGATGATGACGACTAA

>CASQ2_Netta_rufina_protein
MKATCWILAGFCLLFCCKAEEGLNFPTYDGKDRVIDLNEKNYKQALKKYDMLCLLFHEPVSSDKVSQKQFQMTEMVLELAAQVLEPRSI
GFGMVDSKKDAKLAKKLGLVEEGSLYVFKEERLIEFDGELATDVLVEFLLDLLEDPVEIINSKLELQAFDQIDEEIKLIGYFKGEDSEH
YKAFEEAAEHFQPYIKFFATFDKGVAKKLGLKMNEVDFYEPFMDEPVHIPDKPYTEEELVEFVKEHKRATLRKLRPEDMFETWEDDMEG
IHIVAFAEEDDPDGFEFLEILKQVARDNTDNPDLSIVWIDPDDFPLLITYWEKTFKIDLFRPQIGVVNVTDADSVWMEIRDDDDLPSAE
ELEDWIEDVLSGKINTEDDDDDDDDDDDDDDDDDDDDDDDDDDDDDDDDDDDDDDDDDDDDDDDDDD*

6.13 *Oceanites oceanicus* (Wilson's storm petrel)

From: Trinity *de novo* assembly of SRR10853016

>CASQ1_Oceanites_oceanicus_transcript_assembly
CCGGGGGGGGCCGGGGAGGGCCTGGACTTCCCCACCCCCGACGGGCTGGGGCGGGTGCTGCCCGTCACCCTGAGCAACTACAGGGCGCT
GCGGAGGCGGTTCCCAGTGCTGGCCCTGCTCCTCCACCACCGGGCCACCCAGTGCCACCGCGAGATGGAGGAGCTGGTCCTGGAGCTGG
CAGCCCAGGTACTGGAGGAGAAGGGAGTGGGCTTCGGCCTCGTCGACTCCGAGAAGGACGCAGCCGTGGCCAAAAAGCTGGGCCTGAGG
GAGGAGGGCAGCATCTACGTGTTCAAGGAGGACGAGGTGATCGAGTACGACGGGGAGCTGGCGGCGGACACGCTGGTGGAGTTCCTGCT
GGATGTGCTGGAGGAGCCGGTGGAGTTCATTGAGGGCGACCACGAGCTCCAGGCCTTCGAGAACATCGAGGATGACCCCAAACTCATCG
GCTACTTCAAGAACGAAGACTCAGAGCACTTCAAGGCCTTCGAGGCGGCGGCGGCGGAGTTTCACCCCTACATCCCCTTCTTCGCCACC
TTGGACAGCAAGGTGGCCAAGCAGCTCACCCTGAAGCTGAACGAGATCGACTTCTACGAGCCCTTCGTGGAGGAGCCGCTGGCCATCCC
CGCCCGGCCGGCCAGCAAGGAGGAGATCGTGGCCTTCGTGGAGGAGCACAGGCGTGCCACTCTGAGGAAACTCAAACCCGAGAGCATGT
ACGAGACCTGGGAGGATGACATGGACGGGATCCACATCGTGGCCTTCGCAGAGGCGGATGATCCGGATGGGTTCGAGTTCCTGGAGATC
CTGAAGGACGTGGCCCGGGATAACACGGACAACCCCGACCTCAGCATCCTCTGGATTGACCCCAAGGATTTCCCGCTGCTCATCCCCTA
CTGGGAGAAAACCTTCAACATCGACCTGTCCCGCCCCCAGATTGGGGTTGTCAACGTCACCGACGCCGACAGCGTGTGGCTGGAGATGG
CGGACGAGGACGACCTGCCCGGCCCCGCGGAGCTGGAGGAGTGGATCGAGGACGTGCTGGCGGGAGAGACCAACACCGGGGACGATGAC
GATGACAACGACGATGATGACGACGATGACGATGACAACGATGACGATGAAGACGATGATGACGATGACAACGACGACGACGATGATGA
CGATGACGACTAGGCCTTGTGCTGGGCCTCCACTCCTGTCCCTGTCCCTGTCCCTGTCCCTGTCAGTCGTCG

>CASQ1_Oceanites_oceanicus_partial
CCGGGGGGGGCCGGGGAGGGCCTGGACTTCCCCACCCCCGACGGGCTGGGGCGGGTGCTGCCCGTCACCCTGAGCAACTACAGGGCGCT
GCGGAGGCGGTTCCCAGTGCTGGCCCTGCTCCTCCACCACCGGGCCACCCAGTGCCACCGCGAGATGGAGGAGCTGGTCCTGGAGCTGG
CAGCCCAGGTACTGGAGGAGAAGGGAGTGGGCTTCGGCCTCGTCGACTCCGAGAAGGACGCAGCCGTGGCCAAAAAGCTGGGCCTGAGG
GAGGAGGGCAGCATCTACGTGTTCAAGGAGGACGAGGTGATCGAGTACGACGGGGAGCTGGCGGCGGACACGCTGGTGGAGTTCCTGCT
GGATGTGCTGGAGGAGCCGGTGGAGTTCATTGAGGGCGACCACGAGCTCCAGGCCTTCGAGAACATCGAGGATGACCCCAAACTCATCG
GCTACTTCAAGAACGAAGACTCAGAGCACTTCAAGGCCTTCGAGGCGGCGGCGGCGGAGTTTCACCCCTACATCCCCTTCTTCGCCACC
TTGGACAGCAAGGTGGCCAAGCAGCTCACCCTGAAGCTGAACGAGATCGACTTCTACGAGCCCTTCGTGGAGGAGCCGCTGGCCATCCC
CGCCCGGCCGGCCAGCAAGGAGGAGATCGTGGCCTTCGTGGAGGAGCACAGGCGTGCCACTCTGAGGAAACTCAAACCCGAGAGCATGT
ACGAGACCTGGGAGGATGACATGGACGGGATCCACATCGTGGCCTTCGCAGAGGCGGATGATCCGGATGGGTTCGAGTTCCTGGAGATC
CTGAAGGACGTGGCCCGGGATAACACGGACAACCCCGACCTCAGCATCCTCTGGATTGACCCCAAGGATTTCCCGCTGCTCATCCCCTA
CTGGGAGAAAACCTTCAACATCGACCTGTCCCGCCCCCAGATTGGGGTTGTCAACGTCACCGACGCCGACAGCGTGTGGCTGGAGATGG
CGGACGAGGACGACCTGCCCGGCCCCGCGGAGCTGGAGGAGTGGATCGAGGACGTGCTGGCGGGAGAGACCAACACCGGGGACGATGAC
GATGACAACGACGATGATGACGACGATGACGATGACAACGATGACGATGAAGACGATGATGACGATGACAACGACGACGACGATGATGA
CGATGACGACTAG

>CASQ1_Oceanites_oceanicus_protein_partial
PGGAGEGLDFPTPDGLGRVLPVTLSNYRALRRRFPVLALLLHHRATQCHREMEELVLELAAQVLEEKGVGFGLVDSEKDAAVAKKLGLR
EEGSIYVFKEDEVIEYDGELAADTLVEFLLDVLEEPVEFIEGDHELQAFENIEDDPKLIGYFKNEDSEHFKAFEAAAAEFHPYIPFFAT
LDSKVAKQLTLKLNEIDFYEPFVEEPLAIPARPASKEEIVAFVEEHRRATLRKLKPESMYETWEDDMDGIHIVAFAEADDPDGFEFLEI
LKDVARDNTDNPDLSILWIDPKDFPLLIPYWEKTFNIDLSRPQIGVVNVTDADSVWLEMADEDDLPGPAELEEWIEDVLAGETNTGDDD
DDNDDDDDDDDDNDDDEDDDDDDNDDDDDDDDD*

>CASQ2_Oceanites_oceanicus_transcript_assembly
CAGGGCTTGGCCTGACAGCTTCTCCTGTCTGTCTCTTTGTTGTGAGCCCAGGACAGCAGAATTTGCTGCTCCCCACCAGCCTTGCAGAG
TTTGGGCTGATCACTAGGAAGGCAGCAGAAAGAGAAACTTGGTTCGAACTTCACCTGCAGCCTTTGGTCTCTCCCTCTTCCCAAATTAA
GAAGGAGAGGGATGTTTAGCTGCCCTCTGCTGCGCTCCAAGGGCTCCCCTATGAAGGCGACTTGCTGGATCCTGGCAGGTTTTTACCTG
CTTTTCTGCTGCAAAGCAGAAGAGGGACTGAATTTCCCCACTTATGATGGGAAAGACCGAGTGATCGACCTGAATGAGAAGAACTACAA

GCAGGCCCTGAAGAAGTACGACATGCTCTGCCTGCTCTTCCACGAGCCTGTGAGCTCTGACAAGGTCTCCCAGAAGCAGTTCCAGATGA
CAGAGATGGTCCTGGAGCTGGCAGCTCAGGTCCTGGAGCCCAGGAGCATTGGCTTTGGGATGGTAGACTCCAAGAAGGATGCCAAACTT
GCTAAAAAGCTAGGTTTGGTTGAAGAGGGAAGTCTCTATGTCTTTAAGGAGGAGCGGTTGATTGAATTTGATGGGGAACTGGCCACGGA
TGTCTTGGTGGAATTCCTCTTAGATCTGCTAGAAGACCCCGTGGAGATCCTAAACAGCAAGCTGGAGCTTCAGGCCTTTGACCAAATCG
ATGACGAAATCAAACTCATCGGCTACTTCAAAGGAGAAGACTCTGAACATTACAAGGCATTTGAAGAAGCTGCCGAACACTTCCAGCCG
TACATCAAGTTCTTTGCCACCTTCGACAAAGGGGTTGCCAAGAAGCTAGGTCTAAAGATGAATGAGGTGGACTTCTATGAACCGTTTAT
GGATGAGCCTGTTCACATCCCTGATAAGCCTTACACAGAAGAGGAGTTGGTTGAATTTGTGAAGGAGCACAAAAGGGCCACCTTGAGGA
AACTACGCCCAGAGGACATGTTTGAGACATGGGAGGATGACATGGAGGGAATTCACATTGTGGCCTTCGCCGAAGAAGACGACCCAGAT
GGTTTTGAGTTCCTGGAAATCCTGAAGCAGGTTGCCAGGGACAACACTGATAATCCCGACCTGAGCATTGTCTGGATTGACCCCGACGA
CTTTCCTCTGCTGATCACTTACTGGGAGAAGACCTTCAAGATTGACCTGTTCAGACCACAGATCGGGGTGGTGAACGTCACAGACGCTG
ACAGCATCTGGATGGAGATCAGAGATGACGATGACCTGCCCACGGCCGAGGAGCTGGAGGACTGGATAGAGGACGTGCTTTCTGGGAAG
ATAAATACTGAAGACGATGATGATGATGATGACGATGATGACGATGATGATGACGATGATGATGATGATGATGATGACGACGATGATGA
TGACGATGATGACGACGACTAACTATGACTCTGTGCCATTTGACTTGTGGGGGCTGAGAGGCCTCCCCCCGCGGCAGGTCCCTCCATTG
GAAGACCTGTGTTTGAGCGTCAGCCAGCACCGCTGCTCTTCTTCTCCCCTGCCCTGCTCCCCCTGCCCCTGCCCTTGCCAGGACCCCCT
GGCCTGATTCTCAACGGTGCTGAGCCACCGGGACTGCCCCTGCGGCAGGCGGTGCCTGCAGGCACCCAGCACCGCTGTAAATCATGCCT
CCTTAGTAAGGATAATAGGAATCTGCAGGGAATCTGCCCCCAGTGCCCCAGGGCAGGAGGAAAGTGCCTGCCTGCTCACTGCCAGAGGG
ACACAGGGAGCCAATTTGTATTTCCCATGCTCATCGGCCCAGCACTTAATGTCCAAAGACCAAATCTCACTTCAAGACATAGGGAAGGC
CACTGTAAAGTTTAGTCCTTTTATTAAACACAATGCCCTTTGCAGCTACTCTGCCAAGACCACTCAATCCTGCAAGTTCTGCTGCGGGA
GTCCTCATTAACATGCTAGGAGCAGCAAAAAGCTCTGCGGGGGGGAGGAAGCTTTTAGGGATGTTATTTAAGTTTTCAGTGATTCCATA
TAACGAAAGGATATTCTTGTCTGGCAGCATAATGAAAAACCAAACACCACGGTCCCTGTCTGAACCTCTTTCTTCTTGAAAGCTCAGGC
CAAAGATAGGGTAAGTGCAGGTGGTTTGATATGGGACATCAAAGCTTGCAAGTTCCTGAACTGCTAGAAAGGCTGATCCAGGTCTGAGC
CCAAACTCTTGAGGCTGAAGGTCATGGCACTCAGCCAAACCGTTGGTCTTAATCCCAAAGAAATAACAGAGCAGACCCAGGCCAGTTCC
AAACATCTGCAGCCAGGTCTGGGGCTAGGCCTACCTTGCCTTTCAGATGAACCTCAGCGGGATTGAAGGTGGTGCACATGAGATAGACA
GCATACTCCTGCCAGTGGAAACATTCATGGGGCTCATCCCTGAAGCCCTCCTATTCCCCAGGATCTTCCAGCTTTAGCTGACTTTTAAT
CCAAAGCCTACATAGCATCTTTTCCCATGACCAAGATGTTGGGAGGGAACACATCCATCCAACCGTATTAGATGCTGGATAGTGAGAGT
TGGGCAATGAGCAGTGAAAGGATTCAAGCTGTTTGGCTTTTAAGTATTCAGTTGCACAGTTACTCCTTGCTTGGAGACTTGGACATCCC
ACGGTAGCCTGCGAGAAGCCTCTTTTGACTGCAGACTGAGAAGGAGCTGAGGGCACCAGTTCGCAGTCAAGGGGGTCACTCACAAAGCA
AAGGGGTCAGCAGCTCAGTCCTGGCGAGTGCAGCACCATCCATGTGCAGGTCACTGCGACAGTGTTTTGTCTCCCTGCTTTCCCATGCC
AGCAGATTTCAATTGTCTTAGCTTATTAGGTTTGACAATAGTGCAATGAATGGAGAGTTTGGAGATAGTTTTATCTTTATAAATACATT
TATAAATATGTTCTCCAGTTGTTCAGACTTGTTAGCATAGGTAGGGTATACATGTCCTCTCTTGCTACAGACGAAAAACATTGTTCTGA
CCTTTCTTATACTGGCTTACATCAGAGGTAAGTGATGTTAGGGGAAATAACAGTGCGAAGGACAGAAGAATCAGAGTTCATTATATGAA
TGCAAATCAGCCTGATGAGAAACAAGGTTTACACACTATATATTTCCTTTATCAGATGAACTGCAAAGCAAGATTTTATAGATTTTTTT
TTTTCCTGCCTGTTGATCAGTCTCTTTGACTCTCTCTCTCTTTACATTAGCTGAAATAGAAATTTGAAACTACTGGTATAAAAAAGCTG
AAGTACTGCTATATTCCTACGTGTGCTATATCCCCTTGGTGGAAAGTGCCAGCATGAACAATACGCAGCTGGATATCTATATATCGCTA
TGTATGTGAATGAATACTGTGCTTCTATGGTACATGACTGTAGGCCACGTTAGCTCCTCATTGAACATCAAACACTCTACCTGTGCTTT
TCAGGCACAGCTTATTAGAAGAATAGACACGACACCAAAGGCCCTAGGTCAACTACTGTCTTTTATATACTTGCCCTTTCTTGCTTCTT
TATGATGGATAAGCACTGCCTGGAAGACTTGAACTAAGTGAGTTTGGGGACACCATCCACCTGGTCATACTGGGGTTATGAACAACTCA
GCTCCATCCCAGAGGACCTGGTGGAGATTTTCAGAAGGCTTGAAATGCAGAGTGCTCTCAGCCTGGAGGTCCCCTCCTCTCCTTCACAC
ACCCCTCTCTCCTGGCCGGAGGGGTGTCCAGCACCCTCTTTCTGCTCTCTCCTTTCAGCTTTTTCCTCGAGAGGTTTGCTGGAGGACCC
CGGTTCAGACAAAGAGGTAGCAAGAGGGACTCAAGTTCTCATTGATGCACATCAGTAAGGAGCGAGAACAGTCAAAACTAGCAGTTTAG
TAGTCTCCTAGTCTGTGATTAAGGGACAAATGCTGCTTATGTTACACACCTGAGCAAAGCACTTGTTTATTTTTACCTTGGGGCTATTC
CCAGGAGTAAAACACACACAGTTTCACGTGGCATAAGGTAACAGCTCTGGATCAGTCACCGCAAAGTGCCATGAATTTCTATAAACATG
AAGAATAAATGAAAAGTTGCTCTATTGTCTTTCCCAGCTGGCACTGTCTCCAGATGTCCGAATTGCCTGGGTACTCTCCATGTACCTCC
CCTCCCGCTGTGGATGCCCTTCTATTCTAATGTGCCCCTCTCTAGATGCTCTGAGAGGCACAGCTTACACAGGCAGAGCAGGCAGACCC
TGCAAAAGGCACTGCGGACGTAGTCAAGTGCACAGATGTTTCACAGGCACGTACGCATTTCAATGCATCAAGCAACAAAGCTCTGCGAG
GAAAAAAATAATTGATGTAGTGGTGCTTAGGCTTATACAGAGGCCAGTGAGGCTGAGCTCATACTGGGGCTGGTGTCAGTAATTTATGG
AAAGTTTCCCTTAAGTTATGAGAGAGAGGGCTAATCCTATGGCTGCCACTCTCACGGTGCCAGCGCAGTCCTTGATGGGGCCACCCTTT
CCCTCTTCCCTCATCCCCCCATTACGGTTTTCATGGTTCTCTTTGCCCACGAATGGCAACTTATCTTCAGCTTATGGGATAAACACATA
AAAAGTTAAGAAAACGTTGCAGCAGAGAAAGGAATGCAGCAATGAAGAAGAGCAGGCAGCAAAGCCTCTCCCATTCACCCATAACACCT
CATCTGCCCCGAGTTCACAGTGACCGAACCACTGAACAAGGTAATACCCCTTGTGAAGAAGATGTTGGCTTGAATTTGAGTGTCTTCCT
TGCAGGTAAGATACCAGCATGTGGAGGTATCACACACAGGACCCCACGCACAGGTGCTCCTGGGCTCCTCTGTCAGAAGTAGGCACAAC
TGGCACATGAAAGGCACAGGCGACCCTGAGGAGCAAAGTCAGGCTAACCCTGCCCAGTACGCACTCAAAAGCAAAAGGTCAGTAAGTTT
GGATGAGGAACCAGCAAAAAAACTTCTTTATTTAAAAAAATAAAAAGTGTGCGTATGGGCAGCACTGAATGTTTCTGCAGGCAAGTCTA
AGACTGTCCCTGTCTTAGCAAATAGTTCTTTTTTTCCCAAAGTGTTAATTAGATTTTCATATTAAATGGGTCTTAATGCACATAAAGAA
AGACAAGGAATCATACTTTGTCCTCATCCAACTGCGAGTGCTTCAAGCTGATCAGTCATTGCAAAGTACTCAAAAAACCTAGCCTTACC
AGCCCCCAACAATGGTGAGGACCCTCTTCGCAAGAAATACATGGCAAGAACACGAATTTATACGCCACCTCCTCCCAGTCCCCAGACAC
AGGGCCAGGGGCCACAGAAGAGGGGTTTGTTCGGTGTGCTGTACCACAGGCCACCCCATGCTCTTCATGGGCAATGATCAAGGGAGAGG
GAAACCCATCAAGAGCCATATACTACAGCTATCATACAGAAGCTCGGAGAAGAAAGCTTCTGCCTTTGCAGTGGACCCAAGGACAATGA
TGGAGAGGCTCTGTGAACCAGAAACTGCTCTGAGCTAACGTCAACCCTTTGGGGAACAGCTCCAGAAGGAGAGAACAAGGGAGGCACGC
TGGAAGTGACAGATTACCCCATATTTTCAGGGTCAGCAGTTTTTGTGATGTTTCTGAGGAGCTGTGAGAGGCTCCTGTGCTGCCGTTGG

GAGAGGAACAGTCCTGTAGTGAAACCAGAGATCTCAAGCTGCTTCCCATCCCAGAACAGAGCTGCTCGGTCGGATGTCAATACAGACAC
ATAGGCGAGGACACCGGCTGCTTCTCCTTGTCTGTCCACACATCATACCTCGGAAATGATTGTTTCATTACCAAGACCAAACAAAAACC
ATTGCCTTTTATTCCCAGAGAAATAATTAATTAGACAGGAACATATCATTTCCCAGCTGGCGCTGACTTGCAGTGTTGTGAAAACAGGC
CAGAATTGCATCTGGATTCCTCTCTGCCCCAGCAAAGCCTTGGCTTTGGGGAAGCCCTACACACAGCCAGTTGCACACTGGTGCTTGGA
CTTGGGGGGCTGAAGAACAAGAATGGCTCAAAAAGCACGAAGGCCCTCCAGGGCAAAGCAAACTGCCTCCTGGCTACTTTTACACCCCA
GGTATGTCTACGCTGTGCAGTGCAATGCCATCTGCTAGAAACTGGAAGCAGCAGAGCACAGCAGGAGGGTAATTAACCTCGGTAATGAG
GCTGCGCCAGCAGGACTGTGTCCTACTCGAGACCCAAGACTCTGTGCAGCACCCTGCTGCGCTGCAGCATCCCCTGCCTTTGCCCCATC
CCCTTGTACCACTGAGACCGGGGCAAAGCAGCCCTCCAAAGGTCAGTATCCCTTGGGGCCAGCAGTGGGATGGTGCTCACACCTGTTTG
GCAGAAGAAGATGTCTAATGACGATGTGGACCATGGGGCAGGGTCCGGGGCAGAGCTGGCCCTCTTGGCCCTGCTCCCAGAATGCTCCC
TGCCAGGTTTAAAAGGTGCTAAACTGTTTAAAGAGGAAAAAAAAAAAAAAAAGCCTTATAGGTTTCTGTGTTGCACATGACTCATCTTTGC
CATTGTAACTAAAACATTTCCACTAGATTTGCCCAATTCTGTATTTATTTCACTCAAAAGTGTTTCTGCAGAAGGTGAGGGGGGGAAGC
AACGCCCTCCTTCATTTCTCCCAAAATGATGCAAAATGAGGAATTTTGGGGTGGGG

>CASQ2_Oceanites_oceanicus_CDS
ATGAAGGCGACTTGCTGGATCCTGGCAGGTTTTTACCTGCTTTTCTGCTGCAAAGCAGAAGAGGGACTGAATTTCCCCACTTATGATGG
GAAAGACCGAGTGATCGACCTGAATGAGAAGAACTACAAGCAGGCCCTGAAGAAGTACGACATGCTCTGCCTGCTCTTCCACGAGCCTG
TGAGCTCTGACAAGGTCTCCCAGAAGCAGTTCCAGATGACAGAGATGGTCCTGGAGCTGGCAGCTCAGGTCCTGGAGCCCAGGAGCATT
GGCTTTGGGATGGTAGACTCCAAGAAGGATGCCAAACTTGCTAAAAAGCTAGGTTTGGTTGAAGAGGGAAGTCTCTATGTCTTTAAGGA
GGAGCGGTTGATTGAATTTGATGGGGAACTGGCCACGGATGTCTTGGTGGAATTCCTCTTAGATCTGCTAGAAGACCCCGTGGAGATCC
TAAACAGCAAGCTGGAGCTTCAGGCCTTTGACCAAATCGATGACGAAATCAAACTCATCGGCTACTTCAAAGGAGAAGACTCTGAACAT
TACAAGGCATTTGAAGAAGCTGCCGAACACTTCCAGCCGTACATCAAGTTCTTTGCCACCTTCGACAAAGGGGTTGCCAAGAAGCTAGG
TCTAAAGATGAATGAGGTGGACTTCTATGAACCGTTTATGGATGAGCCTGTTCACATCCCTGATAAGCCTTACACAGAAGAGGAGTTGG
TTGAATTTGTGAAGGAGCACAAAAGGGCCACCTTGAGGAAACTACGCCCAGAGGACATGTTTGAGACATGGGAGGATGACATGGAGGGA
ATTCACATTGTGGCCTTCGCCGAAGAAGACGACCCAGATGGTTTTGAGTTCCTGGAAATCCTGAAGCAGGTTGCCAGGGACAACACTGA
TAATCCCGACCTGAGCATTGTCTGGATTGACCCCGACGACTTTCCTCTGCTGATCACTTACTGGGAGAAGACCTTCAAGATTGACCTGT
TCAGACCACAGATCGGGGTGGTGAACGTCACAGACGCTGACAGCATCTGGATGGAGATCAGAGATGACGATGACCTGCCCACGGCCGAG
GAGCTGGAGGACTGGATAGAGGACGTGCTTTCTGGGAAGATAAATACTGAAGACGATGATGATGATGATGACGATGATGACGATGATGA
TGACGATGATGATGATGATGATGATGATGACGACGATGATGATGACGATGATGACGACGACTAA

>CASQ2_Oceanites_oceanicus_protein
MKATCWILAGFYLLFCCKAEEGLNFPTYDGKDRVIDLNEKNYKQALKKYDMLCLLFHEPVSSDKVSQKQFQMTEMVLELAAQVLEPRSI
GFGMVDSKKDAKLAKKLGLVEEGSLYVFKEERLIEFDGELATDVLVEFLLDLLEDPVEILNSKLELQAFDQIDDEIKLIGYFKGEDSEH
YKAFEEAAEHFQPYIKFFATFDKGVAKKLGLKMNEVDFYEPFMDEPVHIPDKPYTEEELVEFVKEHKRATLRKLRPEDMFETWEDDMEG
IHIVAFAEEDDPDGFEFLEILKQVARDNTDNPDLSIVWIDPDDFPLLITYWEKTFKIDLFRPQIGVVNVTDADSIWMEIRDDDDLPTAE
ELEDWIEDVLSGKINTEDDDDDDDDDDDDDDDDDDDDDDDDDDDDDDDDDD*

6.14 *Otis tarda* (great bustard)

From: Trinity *de novo* assembly of SRR24858770. No CASQ1 was assembled. CASQ2 from TRINITY_DN284_c0_g2_i2.

>CASQ2_Otis_tarda_transcript_assembly
GGACAGTAAAGTTTGCTGCTCCTCACCGGCCTTGCAGCGTTTGGACTGATCACTAGGAAGGCAGCAGAAAGAGAAACTCGGCTCAAACT
TCACCTGGAGACTTTGGTCTCCCCCTCTCCCTAAATTAAGAAGAGGGGAGACGTTTACCCGCCCTCCGCTGCACTCCAAGGTTTTCCCC
TATGAAGGCGACTTGCTGGATCCTGGCAGGTTTTTACCTGCTTTTCTGCTGCAAAGCAGAGGAGGGACTGAATTTTCCCACTTATGATG
GGAAAGACCGAGTGATTGACCTGAATGAGAAGAACTACAAGCAGGCCCTGAAGAAGTACGACATGCTCTGCCTGCTCTTCCACGAGCCC
GTGAGCTCTGACAAGGTCTCTCAGAAGCAGTTCCAGATGACAGAGATGGTCCTGGAGCTGGCAGCTCAAGTCCTGGAGCCCAGGAGCAT
CGGCTTTGGGATGGTGGACTCCAAGAAGGATGCCAAACTTGCTAAAAAGCTAGGCTTGGTTGAAGAGGGAAGTCTCTATGTCTTTAAGG
AGGACCGGTTGATTGAATTTGATGGGGAACTGGCCACGGATGTCTTGGTGGAATTCCTCTTGGATCTGCTAGAAGACCCTGTGGAGATC
ATAAACAGCAAGCTGGAGCTTCAGGCCTTTGACCAAATTGACGAGGAAATCAAACTTATCGGCTACTTCAAAGGAGAAGACTCTGAACA
TTACAAGGCATTTGAAGAAGCCGCCGAACACTTCCAGCCGTACATCAAGTTCTTTGCCACCTTCGACAAAGGGGTTGCCAAGAAGCTAG
GTCTGAAGATGAATGAGGTGGACTTCTATGAACCGTTTATGGATGAGCCTGTTCACATCCCCGATAAGCCTTACACAGAAGAGGAGCTG
GTTGAATTTGTGAAGGAGCACAAAAGGGCCACCTTGCGGAAGCTGCGCCCAGAGGACATGTTTGAGACGTGGGAGGATGACATGGAGGG
AATCCACATTGTGGCCTTTGCCGAAGAAGATGACCCAGATGGTTTTGAGTTCCTGGAAATCCTGAAGCAGGTTGCCAGGGACAACACCG
ATAATCCTGACCTGAGTATTGTCTGGATTGACCCCGATGACTTTCCTCTGCTGATCACTTACTGGGAGAAGACCTTCAAGATTGACCTG
TTCAGACCACAGATTGGAGTGGTGAACGTCACAGATGCTGACAGCATTTGGATGGAGATCAGAGATGACGATGACCTGCCCACGGCAGA
GGAGCTGGAGGACTGGATAGAGGATGTGCTTTCTGGGAAGATAAATACTGAAGATGATGACGACGATGACGACGATGATGACGATGATG
ACGATCATGACGATGATGATGATGATGACGATGATGATGATGACGACGATGATGATGATGACTAACTGTGACTCTGTGCAGTTTGACTT
GTGGGGGCTGAGAGGCCTCCCCTCGTGGCAGGTCCCTCCATCAGAAGACCTGTATTTGAGCGTCAGCAAGCACCGCTGCTCCTCTTTTC
CCCTTGCCCCGCTCCCCTTGCCCCAACCCCCCCGCCTGATTCTCAGCGGTGCTGAGCCACCGAGACTGCCCCTGTGGTGGGCAGCACCT
GCAGGCACCCAGCACCGCTGTAAATCATGCCTCCATAGTAAGGACAATAGGAATCTGCAGGGAATCTGCCCCCAGTGCCCCAGAGCAGG
AGGAAAGCGCCTGCCTGCTTGCTGCCAGAGGGACACAGGGAGCCAATTTGTATTTTCCATGCTCATTGGCCCAGCACTTACATCCAAAG
ACCAAATCTCACTTCAAGACATAGGAAAGGCCACTGTAAACTTTAGTCCTTTTATTAAACACAATGCCCTTTGCAGCTACTCTGCCAAG

ACCACTCCATCCTGCAAGTTCTGCTGCGGGAGTCTGTAGCTACTCATTAACGTGCTAGGAGCAGCAGAAAGCTTTGGGAGGGGAGGAAG
GTTTTAGGGATGCTGTTTAAGTTTTCAGTGATTCCATATAATGACAGCATATTCTTGTCTGGCAGAATAATGCAAAACAAATCACCATG
GTCCCTGTCTGAACCTCTTCCTTCTTGAAAGCTCAGGTGAAAGACAGGGTAAGCGCGGGTGGTCTGATACGGGACATCAAAACTGGAGG
TTCCTGAACTGCTGGAAAGGCTGATCCAGATCTGAGCCCAAACTCTTGAGGGTGAAGATCACGGCACCGAGCCAAATTGTTGGTCTTAG
CCCCAAAGAAACGCTAGAGCAGACTCCAGGCCAGTTCCAAACATCTGCAGCCAGGCCTGGGCCTACCTTCCCTTTCAGGTGAACTTCAG
CTGGATTGGAGGTGGTGCACCTGAGATAGACAGCATATTCCTGCCAGTGGAAACATTCATAGGGGCCCACCCTGAAGCCCTCCTGTTCC
CCATGACCTTCCAGCTTTAGCTGACTTTTAACCCAAAGCCTACATAGCATCTTTTCCCATGACCAAGATGTTGGGCGGGGAACAGATCC
GTCCAGCCATATTAGATGCTGGATGACGTGCGTTGGGCAATGAGCAGTGAAAGGATTCAAGCTGTTTGGCTTTTAAGTATTCAGTTGCA
CAGTTACTCCCTGCTTTGAGACTCGGATGTCCCATGGTAGCCTGTGAGAAGCCTCTTTTGACTGCAGACTGAGAAGGAGCTAAGGGCAC
CAGTTCACAGTCAAGAGGTCACTCACAAAGCAAAGGGTCAGCAACTCAGCCCTGGTGAGCGCAGCACCATCCATGTGCAGGTCACTGCA
ACAGTGTTAGGTCTCCCTGCTTTCCCATACTGGCAGATTTCAATTGTCTTAGCTTATTAGGTTTGACAATAGTGCAATGAATGGAGAGT
TTGGACATAGTTTTATCTGTATAAATACATTTATAAATATGTTCTCCAGTCGTTCAGACTTGTTAGCATAGGTAGAGTACACATGTCCT
CTCTTGCTACAGATGAAAATTATTCTCCTGACCTTTCTTATAGTGGTTTACGTCAGAGGTAAGTGATGTTAGTGGAAATAACAGTGCAA
GGGACAGGAGAATCAGAGCTCGTTATACAAATGTAAATCAGCCTGATGAGAAACAAGGTTTACACATTATATATTTCCTTTTCAGATGA
ACTTCAAAGCAAGATTTTACAGATTTTTTTTTCTTGCCTGTTGATCAGTCTCTTTGACTCTCTCTCTCTCTCTCTACGTTAGCTGAAAT
AGAAAATTTAAACTACTGATACAAAAAGGCTGAAGTACTGATATACTCCTACATGTGCCATATCGTCTTGGTGGAAAGTGCCAGCATGA
ACAACACGCAGCTGGGTATCTCTATATCTCTATGTATATGAACGAATATGTGCTTATACGGTATATGACGGTAGGCCATGTTAGCTGCT
CATTGAACATCAGTCAACCCATGCTTTTAAGGCATAGCCTATTAGAAGAATAGACAGGACAGCAAAGCCCCCAGGTCAACTATTTGCCC
TTTCTTGCCTCTTTATGATGTATAAGCACTGCCTGGGAGACTTGAACTAAGTGTGTTTGGGGAGACCATCCCCAAACTGGGGTTATGGA
CAACTCAGCCCCATCCCAGAGGACCTGGTGGAGACTTTCAAAAACCTTGAAAACCAGAGTGCTCTCAGCCTGGAGCTCCCCTCCTCTCT
TTCACTCACGCCCGCCTCTCTCCTGGCCAGAGGGCTGCCCAGCAGCCTCCTTCTGCTCTCTCCTTTCCTTGAGAGGTTTGCTGGAGGAC
CCCGGCTCAGAGAGAGAGGGAGCAAGTGGGACACAAGGTGTCATTTATATACACCAGTAAGGAGTGGGAACAGGAGACAAAAGTAGCAG
TTTAGTAGTCTTCTAGTCCGTGACTGAAGGACAAATGCTGCTTATGTTACTCACCTGAGCAAAATACTTGTTTGTCTTTACCTTGGGGC
TACTCCCAGGAGTAAAAAACGCAGTTTCACCCTGCCTAAGGTAACAGCCTGCACAGGCCCTGCAGCTCCAGATCAGTCACCACAAATTG
CCATGAATTTCTATAAACATGAAGAATAAACAACAAGTTTCTCTACTGCCGTTCCCAACTGGCACTGTCTCTGGACATCCCGACTGCCT
GGGTAGTCTCCATGTACCTCCCCTCCTGCTGTGGATGCCGGTCTATTCCGATGCGTCCCTCTCTAGATGGTCCGAGAGGCAGAGCTTAC
AGAGGCAGAGCAGACAGACCCTGCGAATGGCACTACGGCTGTGTTCAAGTGCACAGATGTTTCACAGGCATGTACATATTTCAATGCAT
CCAGCAACAGAGCCCTGGGGAAAAAAAAAATTAATGTAGTGGTGCTTAGGGTTATACAGAGGCCAGTGAGGCTGAGCTCATGCTGGGGC
TAGTGGGCTGGTGTCAGTGCTTTATGGAAAGTTTCCCTCAAGTTATGAAAGAAAGGCCCAATTCTATAGCTGCTACTCCTTCATGGTGC
CAGCGCAGTCCTTGATGGGGCCATCCTTTCCCTCTGCTCTCATCCCCCCATTACAGTTCTTACGGTTTTCTTTGCCCACAAACAGGAAC
TTTTTATCTTCAGCTTATGGGATAAACACATAAAAAGTTAAGAAAATCATAGAATCATAGAATGGTTAGAGTTGGAAGGGACCTTCAAA
GGTCACCTAGCCCAACCCCCCTGCAAGGAGTAGGGA

>CASQ2_Otis_tarda_CDS
ATGAAGGCGACTTGCTGGATCCTGGCAGGTTTTTACCTGCTTTTCTGCTGCAAAGCAGAGGAGGGACTGAATTTTCCCACTTATGATGG
GAAAGACCGAGTGATTGACCTGAATGAGAAGAACTACAAGCAGGCCCTGAAGAAGTACGACATGCTCTGCCTGCTCTTCCACGAGCCCG
TGAGCTCTGACAAGGTCTCTCAGAAGCAGTTCCAGATGACAGAGATGGTCCTGGAGCTGGCAGCTCAAGTCCTGGAGCCCAGGAGCATC
GGCTTTGGGATGGTGGACTCCAAGAAGGATGCCAAACTTGCTAAAAAGCTAGGCTTGGTTGAAGAGGGAAGTCTCTATGTCTTTAAGGA
GGACCGGTTGATTGAATTTGATGGGGAACTGGCCACGGATGTCTTGGTGGAATTCCTCTTGGATCTGCTAGAAGACCCTGTGGAGATCA
TAAACAGCAAGCTGGAGCTTCAGGCCTTTGACCAAATTGACGAGGAAATCAAACTTATCGGCTACTTCAAAGGAGAAGACTCTGAACAT
TACAAGGCATTTGAAGAAGCCGCCGAACACTTCCAGCCGTACATCAAGTTCTTTGCCACCTTCGACAAAGGGGTTGCCAAGAAGCTAGG
TCTGAAGATGAATGAGGTGGACTTCTATGAACCGTTTATGGATGAGCCTGTTCACATCCCCGATAAGCCTTACACAGAAGAGGAGCTGG
TTGAATTTGTGAAGGAGCACAAAAGGGCCACCTTGCGGAAGCTGCGCCCAGAGGACATGTTTGAGACGTGGGAGGATGACATGGAGGGA
ATCCACATTGTGGCCTTTGCCGAAGAAGATGACCCAGATGGTTTTGAGTTCCTGGAAATCCTGAAGCAGGTTGCCAGGGACAACACCGA
TAATCCTGACCTGAGTATTGTCTGGATTGACCCCGATGACTTTCCTCTGCTGATCACTTACTGGGAGAAGACCTTCAAGATTGACCTGT
TCAGACCACAGATTGGAGTGGTGAACGTCACAGATGCTGACAGCATTTGGATGGAGATCAGAGATGACGATGACCTGCCCACGGCAGAG
GAGCTGGAGGACTGGATAGAGGATGTGCTTTCTGGGAAGATAAATACTGAAGATGATGACGACGATGACGACGATGATGACGATGATGA
CGATCATGACGATGATGATGATGATGACGATGATGATGATGACGACGATGATGATGATGACTAA

>CASQ2_Otis_tarda_protein
MKATCWILAGFYLLFCCKAEEGLNFPTYDGKDRVIDLNEKNYKQALKKYDMLCLLFHEPVSSDKVSQKQFQMTEMVLELAAQVLEPRSI
GFGMVDSKKDAKLAKKLGLVEEGSLYVFKEDRLIEFDGELATDVLVEFLLDLLEDPVEIINSKLELQAFDQIDEEIKLIGYFKGEDSEH
YKAFEEAAEHFQPYIKFFATFDKGVAKKLGLKMNEVDFYEPFMDEPVHIPDKPYTEEELVEFVKEHKRATLRKLRPEDMFETWEDDMEG
IHIVAFAEEDDPDGFEFLEILKQVARDNTDNPDLSIVWIDPDDFPLLITYWEKTFKIDLFRPQIGVVNVTDADSIWMEIRDDDDLPTAE
ELEDWIEDVLSGKINTEDDDDDDDDDDDDDDHDDDDDDDDDDDDDDDDD*

6.15 *Pygoscelis papua* (gentoo penguin)

From: Trinity *de novo* assembly of SRR2980760. No CASQ1 was assembled. CASQ2 from complement of TRINITY_DN7282_c0_g1_i1.

>CASQ2_Pygoscelis_papua_transcript_assembly
TACGCGGGGACAGCTTCTCCTGTCTGTCTCTTTGTTGTGAGCCCAGGACAGCAGAGTTTGCTGCTCCCCACCAGCCTTGCAGAGTTTGG
GCTGATCACTAGGAAGGCAGCAGAAAGAGAAACTCAGTTCGAACTTCACCTGGAGCCTTTGGTCTCCCCCTCTTCCCAAATTAAGAAGG
AGAGGGACGTTTGCCTGCCCTCTGCTGCACTCCAAGGGCTCCCCTATGAAGGCGACTTGCTGGATCCTGGCAGGTTTTTACCTGCTTTT
CTGCTGCAAAGCAGAAGAGGGACTGAATTTCCCCACCTACGATGGGAAAGACCGAGTGATCGACCTGAACGAGAAGAACTACAAGCAGG
CCTTGAAGAAGTACGACATGCTCTGCCTGCTCTTCCATGAGCCCGTGAGCTCTGACAAGGTCTCCCAGAAGCAGTTCCAGATGACAGAG
ATGGTCCTGGAGCTGGCAGCTCAGGTCCTGGAGCCCAGGAGCATTGGCTTCGGGATGGTGGACTCAAAGAAAGATGCCAAACTTGCTAA
AAAGTTAGGCTTAGTTGAAGAGGGAAGTCTCTATGTCTTTAAGGAGGAGCGGTTGATTGAATTTGATGGGGAACTGGCCACAGATGTCT
TGGTGGAATTCCTCTTGGATCTGCTAGAAGACCCTGTGGAGATCCTAAACAGCAAGCTGGAGCTTCAGGCCTTTGACCAAATGGATGAC
GAAATCAAACTCATCGGCTACTTCAAAGGAGAAGACTCTGAACATTACAAAGCATTTGAAGAAGCCGCCGAACACTTCCAGCCGTACAT
CAAGTTCTTTGCCACCTTCGATAAAGGGGTTGCCAAGAAGCTAGGTCTGAAGATGAATGAGGTGGACTTCTATGAACCGTTTATGGATG
AGCCTGTTCACATCCCCGATAAGCCTTACACAGAAGAGGAGCTGGTTGAATTTGTGAAGGAGCACAAAAGGGCCACCTTGAGGAAACTG
CGTCCAGAGGACATGTTTGAGACGTGGGAGGATGACATGGAGGGAATCCACATTGTGGCCTTTGCCGAAGAAGACGACCCAGATGGTTT
TGAGTTCCTGGAAATCCTGAAGCAGGTTGCCAGGGACAACACCGATAATCCCGACCTGAGCATTGTCTGGATTGACCCCGACGACTTTC
CTCTGCTGATCACTTACTGGGAGAAGACCTTCAAGATTGACCTGTTCAGACCACAGATCGGGGTGGTGAACGTCACAGACGCTGACAGC
GTCTGGATGGACATCAGAGATGACGATGACCTGCCCACGGCCGAGGAGCTGGAGGACTGGATAGAGGATGTGCTTTCTGGGAAGATAAA
TACCGAAGATGATGATGATGACGACGACGATGATGATGACGACGATGACGATGATGATGATGACGATGACGATGACGATGATGACGACG
ACGACGACTAACTGTGACTCTGTGCAGTTTGACTTGTGGGGGCTGAGAGGCCTCCCCCCGCAGCAGGTCCCTCCATCGGAAGACCTGTG
TTTGAGCGTCAGCAAGCACCACTGCTCCTCTTTCCCCCCTGCCCTGCTCCCCCTGCCCCTGCCCTTGCAGGGACCCCCCGGCCTGATTC
TCAGCGGTGCTGAGCCACTGGGACTGCCCCTGGGGCAGGCGGGGCCCGCAGGCACCCCGCACCGCCGTAAAAAATGCCTCCTTTGTAAG
GACAATAAGAACTTGCAGGGGGGCT

>CASQ2_Pygoscelis_papua_CDS
ATGAAGGCGACTTGCTGGATCCTGGCAGGTTTTTACCTGCTTTTCTGCTGCAAAGCAGAAGAGGGACTGAATTTCCCCACCTACGATGG
GAAAGACCGAGTGATCGACCTGAACGAGAAGAACTACAAGCAGGCCTTGAAGAAGTACGACATGCTCTGCCTGCTCTTCCATGAGCCCG
TGAGCTCTGACAAGGTCTCCCAGAAGCAGTTCCAGATGACAGAGATGGTCCTGGAGCTGGCAGCTCAGGTCCTGGAGCCCAGGAGCATT
GGCTTCGGGATGGTGGACTCAAAGAAAGATGCCAAACTTGCTAAAAAGTTAGGCTTAGTTGAAGAGGGAAGTCTCTATGTCTTTAAGGA
GGAGCGGTTGATTGAATTTGATGGGGAACTGGCCACAGATGTCTTGGTGGAATTCCTCTTGGATCTGCTAGAAGACCCTGTGGAGATCC
TAAACAGCAAGCTGGAGCTTCAGGCCTTTGACCAAATGGATGACGAAATCAAACTCATCGGCTACTTCAAAGGAGAAGACTCTGAACAT
TACAAAGCATTTGAAGAAGCCGCCGAACACTTCCAGCCGTACATCAAGTTCTTTGCCACCTTCGATAAAGGGGTTGCCAAGAAGCTAGG
TCTGAAGATGAATGAGGTGGACTTCTATGAACCGTTTATGGATGAGCCTGTTCACATCCCCGATAAGCCTTACACAGAAGAGGAGCTGG
TTGAATTTGTGAAGGAGCACAAAAGGGCCACCTTGAGGAAACTGCGTCCAGAGGACATGTTTGAGACGTGGGAGGATGACATGGAGGGA
ATCCACATTGTGGCCTTTGCCGAAGAAGACGACCCAGATGGTTTTGAGTTCCTGGAAATCCTGAAGCAGGTTGCCAGGGACAACACCGA
TAATCCCGACCTGAGCATTGTCTGGATTGACCCCGACGACTTTCCTCTGCTGATCACTTACTGGGAGAAGACCTTCAAGATTGACCTGT
TCAGACCACAGATCGGGGTGGTGAACGTCACAGACGCTGACAGCGTCTGGATGGACATCAGAGATGACGATGACCTGCCCACGGCCGAG
GAGCTGGAGGACTGGATAGAGGATGTGCTTTCTGGGAAGATAAATACCGAAGATGATGATGATGACGACGACGATGATGATGACGACGA
TGACGATGATGATGATGACGATGACGATGACGATGATGACGACGACGACGACTAA

>CASQ2_Pygoscelis_papua_protein
MKATCWILAGFYLLFCCKAEEGLNFPTYDGKDRVIDLNEKNYKQALKKYDMLCLLFHEPVSSDKVSQKQFQMTEMVLELAAQVLEPRSI
GFGMVDSKKDAKLAKKLGLVEEGSLYVFKEERLIEFDGELATDVLVEFLLDLLEDPVEILNSKLELQAFDQMDDEIKLIGYFKGEDSEH
YKAFEEAAEHFQPYIKFFATFDKGVAKKLGLKMNEVDFYEPFMDEPVHIPDKPYTEEELVEFVKEHKRATLRKLRPEDMFETWEDDMEG
IHIVAFAEEDDPDGFEFLEILKQVARDNTDNPDLSIVWIDPDDFPLLITYWEKTFKIDLFRPQIGVVNVTDADSVWMDIRDDDDLPTAE
ELEDWIEDVLSGKINTEDDDDDDDDDDDDDDDDDDDDDDDDDDDDDDD*

6.16 *Rhea pennata* (Darwin's rhea)

From: Trinity *de novo* assembly of SRR27716236. CASQ1 from TRINITY_DN16371_c0_g2_i1. CASQ2 from complement of TRINITY_DN1139_c0_g1_i9.

>CASQ1_Rhea_pennata_transcript_assembly
TGGGGGCTGTGCCTGGAGCTGCGCGCGCTCCCGCGGGGCTGCCCGTCCGTCCGTCCGTCCATCCGTCCATCCATCCGTCCGTCCATCCA
TCCATCCATCCATCCATCCATCCATCCATCTGCCCATCCGCCCCGCGGACTGAGTGGCTGTCCGGTCCCTCCGTGAGTCGGCACCCCTC
CGTCCATCCGTCCATCCCGTCCGTCCGTCCCGCAAGCGGGCCATGCGGGGCTGGGCTTGGGCGCTGGCGGCGCTGGCGCTGGCCGCCTG
GGCGCCCGGGCCGACGCCGGCCGAGGAGGGCCTCGATTTCCCCACCTACGACGGCGTCGACCGCGTCCTGGCCGTCACGGGCAAGAACT
ACAAGGCGATGCTGAAACGGTTCCCGGTGCTGGCCCTGCTCTACCACGAGCCCGTGGGCAGCGACCGGGTGGCCCAGCGCCACTTCGAG
ATGGAGGAGCTCATCCTGGAGCTGGCGGCGCAGGTCCTGGAGGACAAGGGTGTCGGGTTTGGCCTCGTCGACTCCGAGAAGGATGCGGC
CGTGGCCAAGAAGCTGGGCCTGACGGAGGAGGACAGCATCTACGTGTTCAAGGAGGACGAGGTGATCGAGTACGACGGGGAGCTGGCAG
CGGACACCCTGGTGGAGTTCCTGCTGGACGTGCTGGAGGACCCTGTGGAGTTCATTGAGGGCGACCATGAGCTCCAGGCCTTCGAGAAC
ATCGAGGATGACCCCAAACTCATTGGCTACTTCAAGAACGAGGACTCAGAGCACTTCAAGGCCTTTGAGGAGGCGGCAGAGGAGTTCCA
CCCCTACATCCCCTTCTTCGCCACCTTTGATAGCAAGGTGGCCAAAAAACTGACTCTGAAGCTCAATGAGATTGACTTCTATGAGCCCT
TCATGGAAGAGCCGGTCACAATCCCTGACAAACCCAACAGCAAGGAAGAGATTGTGGAGTTTGTGGAGGAGCACAAGAGGGCAACTCTG

CGGAAACTCAAGCCCGAGAGCATGTACAACACGTGGGAGGACGACATGGACGGGATCCACATCGTGGCCTTTGCCGAGGAGGATGACCC
GGACGGTTTCGAGTTCCTGGAGATCCTCAAGGACGTGGCCCAGGACAACACGGACAACCCCGACCTCAGCATGATCTGGATCGACCCCG
AGGACTTCCCGCTGCTCATCCCCTACTGGGAGAAGACCTTCAACATCGACCTGTCTCGGCCCCAGATCGGGGTGGTCAACGTCACGGAC
GCCGACAGCGTGTGGCTGGAGATGGCGGACGAGGACGACCTGCCCAGCACGGCGGAGCTGGAGCAGTGGATCGAGGACGTGCTGGAGGG
CGAGATCAACACGGAGGATGACGACGAGGACGACGATGACGACGATGACGATGACGACGACTAGGCCCCGGGGCCGGGCCGTGGCCCTC
TGCCCTGCCCCGCTGTCCCCTCCCCTGTCCCTGTCCGAGCCGGTCGAGCCATTAAAGACCCCGAGGAACCC

>CASQ1_Rhea_pennata_CDS
ATGCGGGGCTGGGCTTGGGCGCTGGCGGCGCTGGCGCTGGCCGCCTGGGCGCCCGGGCCGACGCCGGCCGAGGAGGGCCTCGATTTCCC
CACCTACGACGGCGTCGACCGCGTCCTGGCCGTCACGGGCAAGAACTACAAGGCGATGCTGAAACGGTTCCCGGTGCTGGCCCTGCTCT
ACCACGAGCCCGTGGGCAGCGACCGGGTGGCCCAGCGCCACTTCGAGATGGAGGAGCTCATCCTGGAGCTGGCGGCGCAGGTCCTGGAG
GACAAGGGTGTCGGGTTTGGCCTCGTCGACTCCGAGAAGGATGCGGCCGTGGCCAAGAAGCTGGGCCTGACGGAGGAGGACAGCATCTA
CGTGTTCAAGGAGGACGAGGTGATCGAGTACGACGGGGAGCTGGCAGCGGACACCCTGGTGGAGTTCCTGCTGGACGTGCTGGAGGACC
CTGTGGAGTTCATTGAGGGCGACCATGAGCTCCAGGCCTTCGAGAACATCGAGGATGACCCCAAACTCATTGGCTACTTCAAGAACGAG
GACTCAGAGCACTTCAAGGCCTTTGAGGAGGCGGCAGAGGAGTTCCACCCCTACATCCCCTTCTTCGCCACCTTTGATAGCAAGGTGGC
CAAAAAACTGACTCTGAAGCTCAATGAGATTGACTTCTATGAGCCCTTCATGGAAGAGCCGGTCACAATCCCTGACAAACCCAACAGCA
AGGAAGAGATTGTGGAGTTTGTGGAGGAGCACAAGAGGGCAACTCTGCGGAAACTCAAGCCCGAGAGCATGTACAACACGTGGGAGGAC
GACATGGACGGGATCCACATCGTGGCCTTTGCCGAGGAGGATGACCCGGACGGTTTCGAGTTCCTGGAGATCCTCAAGGACGTGGCCCA
GGACAACACGGACAACCCCGACCTCAGCATGATCTGGATCGACCCCGAGGACTTCCCGCTGCTCATCCCCTACTGGGAGAAGACCTTCA
ACATCGACCTGTCTCGGCCCCAGATCGGGGTGGTCAACGTCACGGACGCCGACAGCGTGTGGCTGGAGATGGCGGACGAGGACGACCTG
CCCAGCACGGCGGAGCTGGAGCAGTGGATCGAGGACGTGCTGGAGGGCGAGATCAACACGGAGGATGACGACGAGGACGACGATGACGA
CGATGACGATGACGACGACTAG

>CASQ1_Rhea_pennata_protein
MRGWAWALAALALAAWAPGPTPAEEGLDFPTYDGVDRVLAVTGKNYKAMLKRFPVLALLYHEPVGSDRVAQRHFEMEELILELAAQVLE
DKGVGFGLVDSEKDAAVAKKLGLTEEDSIYVFKEDEVIEYDGELAADTLVEFLLDVLEDPVEFIEGDHELQAFENIEDDPKLIGYFKNE
DSEHFKAFEEAAEEFHPYIPFFATFDSKVAKKLTLKLNEIDFYEPFMEEPVTIPDKPNSKEEIVEFVEEHKRATLRKLKPESMYNTWED
DMDGIHIVAFAEEDDPDGFEFLEILKDVAQDNTDNPDLSMIWIDPEDFPLLIPYWEKTFNIDLSRPQIGVVNVTDADSVWLEMADEDDL
PSTAELEQWIEDVLEGEINTEDDDEDDDDDDDDDDD*

>CASQ2_Rhea_pennata_transcript_assembly
TGCTTCTCCTGTCTGTCTCTTTGTTTTGAGCCCAGGACTGCAAAGTTTGCTGCTTCCCACCAGCCTTGCAGAGTTTGGACTGATCATTA
GGAAGGCAGAAGAAACTGTTCAAACTTCATCAGTGTCTTTGGTCTCCCTCTCTTTCCAAACAGAGAAGAAAGGAGACATTCACCTGCCT
TCCACTCCACTCCAGAGGTTTCCCTATGAAGGCAACTTGCTGGATTCTGGCAGGTTTTTACCTGCTTTTCTGCTGCAAAGCAGAAGAGG
GACTACATTTCCCCACTTACGATGGGAAAGACCGAGTGATTGACTTGAACGAAAAGAACTATAAGCAAGCCCTGAAGAAGTATGACATG
CTATGCTTGCTCTTCCATGAGCCTGTGAGCTCCGACAAGGTCTCCCAGAAGCAGTTCCAGATGACAGAGATGGTCCTGGAGCTGGCAGC
TCAGGTCCTGGAACCTCGGAGCATTGGCTTTGGTATGGTGGACTCCAAGAAGGATGCCAAGCTTGCCAAAAAGCTAGGCTTAGTTGAAG
AAGGAAGCCTCTATGTCTTTAAAGAGGAAAGATTGATTGAATTTGATGGGGAACTGGCCACAGATGTCTTGGTGGAATTCCTCTTGGAT
CTGCTAGAAGACCCTGTGGAGATCATAAACAGCAAGCTGGAACTTCAGGCCTTTGATCAAATCGATGAGGAAATCAAACTCATTGGCTA
CTTCAAAGGAGAAGACTCTGAACATTACAAAGCATTTGAGGAGGCAGCTGAACACTTTCAGCCATACATCAAATTTTTTGCCACCTTTG
ACAAAGGGGTTGCCAAGAAACTAGGTCTGAAGATGAATGAAGTGGATTTCTATGAACCATTTATGGATGAACCTGTTCACATCCCCGAT
AAGCCTTACACAGAGGAGGAATTGGTTGAATTTGTGAAGGAGCACAAAAGGGCCACCTTGCGGAAGCTGCGCCCAGAAGACATGTTTGA
GACCTGGGAGGATGACATGGAAGGAATCCACATTGTGGCCTTTGCTGAAGAAGATGATCCAGATGGCTTTGAGTTCCTGGAAATTCTGA
AGCAGGTTGCCAGGGACAACACGGATAATCCCGATCTGAGCATTGTGTGGATTGACCCCGATGACTTTCCACTGCTGATCACTTACTGG
GAGAAGACTTTCAAGATTGACCTGTTCAGACCACAGATTGGGGTGGTGAACGTCACAGATGCTGACAGTGTCTGGATGGAGATCAGAGA
TGACGATGACCTGCCCACAGCTGAAGAGCTGGAAGACTGGATAGAGGATGTGCTTTCTGGGAAGATAAATACTGAAGATGATGACGATG
ATGATGACGATAACGATGATGATGATGACAATGACGACGATGATGATGATGACGACGACGATGACGATGATGACGATGATTAGCTTTGA
CTGTGTACAGTTTGACTTGTGGGTGCTGAGAGGCCTCCACTGCAGCAGGTCACTTCATCAGAAGACCTGTGTTTGAGTGTCAGCAAGCA
CCTCCCTGCTCCTCCTTCCCCCTCTGCCCTGCTTCCCTTGCCCTTGCTAGGAGTCCCCACAGCCTGATTCTCAGCGGTGCTTGAGCGAC
CAGGATGCCCCCTGCAGTGGGTGACGTCTGCAGGCACCCAGCACCACTGTAAATCATGCTTCCTTAATACAGACAGTAAGAATTTGCAG
GGAATCTCCCACGAATCTCCCCAAGGTGGGAGGAAAAGTGCCTGCCCACTCACTGCCAGAGAAACACATTGAGCCAATTTATATTATCC
ATGCTCATCAGCCCAACACTTACTTAACATCCAAAGACCAAATCTCATTCATAGAGAAGGTCACTGTAAAGTTTAGTTCTTTTTATCAA
ACAAAATACGCTTTGCAGCTACCCTGCCAAGACCACTGAATCCTCCTGAAAGTTCTGCTGTGGGAGTCCATAGCTACTCATTACCATGT
TGTAAGTAGCAAAAAGGTTTGGGAAGGGACAAAGGCTTTACAGAGACTAATTAAATTTTCAGCTATTCTATAAAACAGGATATTCTTGT
CTGGCAAAGCAGTGCAAAACAAAAACAACAAAAAAAAAAATAGAGCCTATCTGGCACAGTGTCTTCTTGAAAGCTCAGGCT

>CASQ2_Rhea_pennata_CDS
ATGAAGGCAACTTGCTGGATTCTGGCAGGTTTTTACCTGCTTTTCTGCTGCAAAGCAGAAGAGGGACTACATTTCCCCACTTACGATGG
GAAAGACCGAGTGATTGACTTGAACGAAAAGAACTATAAGCAAGCCCTGAAGAAGTATGACATGCTATGCTTGCTCTTCCATGAGCCTG
TGAGCTCCGACAAGGTCTCCCAGAAGCAGTTCCAGATGACAGAGATGGTCCTGGAGCTGGCAGCTCAGGTCCTGGAACCTCGGAGCATT
GGCTTTGGTATGGTGGACTCCAAGAAGGATGCCAAGCTTGCCAAAAAGCTAGGCTTAGTTGAAGAAGGAAGCCTCTATGTCTTTAAAGA
GGAAAGATTGATTGAATTTGATGGGGAACTGGCCACAGATGTCTTGGTGGAATTCCTCTTGGATCTGCTAGAAGACCCTGTGGAGATCA

TAAACAGCAAGCTGGAACTTCAGGCCTTTGATCAAATCGATGAGGAAATCAAACTCATTGGCTACTTCAAAGGAGAAGACTCTGAACAT
TACAAAGCATTTGAGGAGGCAGCTGAACACTTTCAGCCATACATCAAATTTTTTGCCACCTTTGACAAAGGGGTTGCCAAGAAACTAGG
TCTGAAGATGAATGAAGTGGATTTCTATGAACCATTTATGGATGAACCTGTTCACATCCCCGATAAGCCTTACACAGAGGAGGAATTGG
TTGAATTTGTGAAGGAGCACAAAAGGGCCACCTTGCGGAAGCTGCGCCCAGAAGACATGTTTGAGACCTGGGAGGATGACATGGAAGGA
ATCCACATTGTGGCCTTTGCTGAAGAAGATGATCCAGATGGCTTTGAGTTCCTGGAAATTCTGAAGCAGGTTGCCAGGGACAACACGGA
TAATCCCGATCTGAGCATTGTGTGGATTGACCCCGATGACTTTCCACTGCTGATCACTTACTGGGAGAAGACTTTCAAGATTGACCTGT
TCAGACCACAGATTGGGGTGGTGAACGTCACAGATGCTGACAGTGTCTGGATGGAGATCAGAGATGACGATGACCTGCCCACAGCTGAA
GAGCTGGAAGACTGGATAGAGGATGTGCTTTCTGGGAAGATAAATACTGAAGATGATGACGATGATGATGACGATAACGATGATGATGA
TGACAATGACGACGATGATGATGATGACGACGACGATGACGATGATGACGATGATTAG

>CASQ2_Rhea_pennata_protein
MKATCWILAGFYLLFCCKAEEGLHFPTYDGKDRVIDLNEKNYKQALKKYDMLCLLFHEPVSSDKVSQKQFQMTEMVLELAAQVLEPRSI
GFGMVDSKKDAKLAKKLGLVEEGSLYVFKEERLIEFDGELATDVLVEFLLDLLEDPVEIINSKLELQAFDQIDEEIKLIGYFKGEDSEH
YKAFEEAAEHFQPYIKFFATFDKGVAKKLGLKMNEVDFYEPFMDEPVHIPDKPYTEEELVEFVKEHKRATLRKLRPEDMFETWEDDMEG
IHIVAFAEEDDPDGFEFLEILKQVARDNTDNPDLSIVWIDPDDFPLLITYWEKTFKIDLFRPQIGVVNVTDADSVWMEIRDDDDLPTAE
ELEDWIEDVLSGKINTEDDDDDDDDNDDDDDNDDDDDDDDDDDDDDDD*

7 Aves: Psittaciformes

7.1 *Lathamus discolor* (Swift parrot)

From: Trinity *de novo* assembly of SRR26186077. CASQ2 from complement of TRINITY_DN2200_c1_g1_i7.

>CASQ2_Lathamus_discolor_transcript_assembly
AGAAAAAGAAACTCGGTTTGAACTTCACCTGGAGCCTTTGGTCTCTCCTTCTCCCCAAATTAAGAAGGAGGGGGATGTTTACCTGCCCT
CTGCTGCACTCCAAGGTCTCCCCTATGAAGGCAAGTTGCTGGATCCTGGCAGGTCTTTACCTGCTTTCCTGCTGCAAGGCAGAAGAGGG
ACTGAACTTCCCCACTTATGATGGGAAAGACCGAGTGATCGACCTGAACGAGAAGAACTACAAGCAGGCCCTGAAGAAGTATGACATGC
TCTGCCTGCTCTTCCACGAGCCAGTGAGCTCTGACAAGGTCTCCCAGAAGCAGTTCCAGATGACAGAGATGGTCCTGGAGCTGGCGGCT
CAGGTCCTGGAGCCCAAGAGCATTGGCTTCGGGATGGTTGACTCCAAGAAGGATGCCAAACTTGCCAAGAAGTTAGGCTTGGTTGAAGA
GGGAAGTCTCTATGTCTTTAAGGAAGAGCGGTTGATTGAATTTGATGGGGAACTGGCCACAGATGTCTTGGTGGAATTCCTCTTGGATG
TGCTAGAAGACCCTGTGGAAGTCATAAACAGCAAGCTGGAGCTTCAGGCCTTTGACCGAATCGATGAGGAAATCAAACTCATTGGCTAC
TTCAAAGGAGAAGACTCTGAACATTACAAAGCATTTGAAGAAGCTGCTGAACACTTCCAGCCGTACGTCAAGTTCTTTGCCACCTTTGA
CAAGGGGGTTGCCAAGAAGCTAGGTCTGAAGATGAACGAGGTGGACTTCTATGAACCGTTTATGGATGAGCCTGTTCACATCCCTGATA
AGCCTTACTCAGAAGAGGAGCTGGTTGAATTTGTGAAGGAGCACAAGAGGGCCACCTTGCGGAAGCTGCGCCCAGAGGACATGTTTGAG
ACATGGGAGGATGACATGGAGGGAATTCACATCGTGGCCTTCGCAGAAGAAGACGACCCAGATGGGTTTGAGTTCCTGGAAGTCCTGAA
GCAGGTTGCCAGGGACAACACCGAAAATCCCGACCTGAGCATTGTCTGGATTGACCCCGACGACTTCCCTCTGCTCATCACCTACTGGG
AGAAGACCTTCAAGATTGACCTGTTCAAGCCACAGATTGGGGTGGTGAACGTCACAGACGCTGACAGCATCTGGATGGACATCAGAGAT
GATGATGACCTGCCCACAGCAGAGGAGCTGGAGGACTGGATAGAAGATGTGCTTTCTGGGAAGATAAATACCGAAGATGACGATGACGA
TGATGATGACGATGACGATGATGATGATGACGATGATGGATGATGATGATGATGATGATGGATGATGAAGATGACGATGATGATGATGA
CGATGATGGACGATGATGACGATGATTAATGATGGATGATGACGATGATAATGGATGAAGATGACGATGATGGATGACGATGATAATGG
ATGATGATGATGATGGATGATGATGATGATGATAATGGATGATGATGATGACGATGATGGATGATGATGATGATGGATGATGATGTTGA
TGATGACGATGATGATGATGACGACGATGACGACGACGATGACGACGATGATGATGATGATGATTAACTGTGACTCTGTGCAGTTTGAC
TTGTGGGGGCCCAGAGCCCTCCTCCTTCGGCAGGTCCCTCCATTGGAAGGCCTGTGCTTGAGCGTCAGCAAGCACCGATGCTCTCCTTT
GCCCCCTGCCCTGCTCCCCCTGCCCCTGCCTCTGCCCTTGCCGGGACCCCGCAGCCTGATTCTCAGTGGTGCTGAGCCACCAGGACTGC
CCCTGCAGGCACCCAGCACCGCTGCAAATCATGCCTCCTTAGAAAGGACAATAGGAATCCACAGGGAATCTGCCCTGAGCATCCCAGGG
TGGGAGAAAAGTGCCTGCCTGCTCACTGCCAGAGGCACACAGGAAGCCAAGTTGTGTTTTCCATGCTCATCGGCCCAGCACTTAACATC
CAAAGACCAAATCTCACTTCAAGACACAGGGAAGGCCACTGTAAAGTTTAGTCCTTTTATTAAACACAATGCCCTTTGCAGCTTATTCT
GCCAAGACCACTCAATCCTGG

>CASQ2_Lathamus_discolor_CDS
ATGAAGGCAAGTTGCTGGATCCTGGCAGGTCTTTACCTGCTTTCCTGCTGCAAGGCAGAAGAGGGACTGAACTTCCCCACTTATGATGG
GAAAGACCGAGTGATCGACCTGAACGAGAAGAACTACAAGCAGGCCCTGAAGAAGTATGACATGCTCTGCCTGCTCTTCCACGAGCCAG
TGAGCTCTGACAAGGTCTCCCAGAAGCAGTTCCAGATGACAGAGATGGTCCTGGAGCTGGCGGCTCAGGTCCTGGAGCCCAAGAGCATT
GGCTTCGGGATGGTTGACTCCAAGAAGGATGCCAAACTTGCCAAGAAGTTAGGCTTGGTTGAAGAGGGAAGTCTCTATGTCTTTAAGGA
AGAGCGGTTGATTGAATTTGATGGGGAACTGGCCACAGATGTCTTGGTGGAATTCCTCTTGGATGTGCTAGAAGACCCTGTGGAAGTCA
TAAACAGCAAGCTGGAGCTTCAGGCCTTTGACCGAATCGATGAGGAAATCAAACTCATTGGCTACTTCAAAGGAGAAGACTCTGAACAT
TACAAAGCATTTGAAGAAGCTGCTGAACACTTCCAGCCGTACGTCAAGTTCTTTGCCACCTTTGACAAGGGGGTTGCCAAGAAGCTAGG
TCTGAAGATGAACGAGGTGGACTTCTATGAACCGTTTATGGATGAGCCTGTTCACATCCCTGATAAGCCTTACTCAGAAGAGGAGCTGG
TTGAATTTGTGAAGGAGCACAAGAGGGCCACCTTGCGGAAGCTGCGCCCAGAGGACATGTTTGAGACATGGGAGGATGACATGGAGGGA
ATTCACATCGTGGCCTTCGCAGAAGAAGACGACCCAGATGGGTTTGAGTTCCTGGAAGTCCTGAAGCAGGTTGCCAGGGACAACACCGA
AAATCCCGACCTGAGCATTGTCTGGATTGACCCCGACGACTTCCCTCTGCTCATCACCTACTGGGAGAAGACCTTCAAGATTGACCTGT

TCAAGCCACAGATTGGGGTGGTGAACGTCACAGACGCTGACAGCATCTGGATGGACATCAGAGATGATGATGACCTGCCCACAGCAGAG
GAGCTGGAGGACTGGATAGAAGATGTGCTTTCTGGGAAGATAAATACCGAAGATGACGATGACGATGATGATGACGATGACGATGATGA
TGATGACGATGATGGATGA

>CASQ2_Lathamus_discolor_protein
MKASCWILAGLYLLSCCKAEEGLNFPTYDGKDRVIDLNEKNYKQALKKYDMLCLLFHEPVSSDKVSQKQFQMTEMVLELAAQVLEPKSI
GFGMVDSKKDAKLAKKLGLVEEGSLYVFKEERLIEFDGELATDVLVEFLLDVLEDPVEVINSKLELQAFDRIDEEIKLIGYFKGEDSEH
YKAFEEAAEHFQPYVKFFATFDKGVAKKLGLKMNEVDFYEPFMDEPVHIPDKPYSEEELVEFVKEHKRATLRKLRPEDMFETWEDDMEG
IHIVAFAEEDDPDGFEFLEVLKQVARDNTENPDLSIVWIDPDDFPLLITYWEKTFKIDLFKPQIGVVNVTDADSIWMDIRDDDDLPTAE
ELEDWIEDVLSGKINTEDDDDDDDDDDDDDDDDDDG*

7.2 *Myiopsitta monachus* (monk parakeet)

From: Trinity *de novo* assembly of SRR13480237. No CASQ1 found. CASQ2 from TRINITY_DN542_c0_g1_i1.

>CASQ2_Myiopsitta_monachus_transcript_assembly
CAGGGCTTGGCCTGACAGCTTCTCCTGTCTGTCTCTTTGTTGTGAGCCCAGGACAGCAAAGTTTGCTGTTCCCCACCAGCCTTGCAGCA
TTCGGGCTGATCATTAACAGGGCAACAGAAAAAGAAACTCAGTTTGAACTTCACCTGGAGACTTTGGTCTTTCCTTCTCCCCAAATTAA
GAAGGAGGGCGATGTTTACCTGCCCTCTGCTGCACTCCAAGGTCTCCCCTATGAAGGCGACTTGCTGGATCCTGGCAGGTCTTTACCTG
CTTTCCTGCTGCAAGGCGGAAGAGGGACTGAACTTCCCCACTTATGATGGGAAAGACCGAGTGATCGACCTGAACGAGAAGAACTACAA
GCAGGCCCTGAAGAAGTATGACATGCTCTGCCTGCTCTTCCACGAGCCGGTGAGCTCTGACAAGGTCTCTCAGAAGCAGTTCCAGATGA
CAGAGATGGTCCTGGAGCTGGCGGCTCAGGTCCTGGAGCCCAAGAGCATTGGCTTTGGGATGGTTGACTCCAAGAAGGATGCCAAACTT
GCCAAGAAGTTAGGCTTGATTGAAGAGGGAAGTCTCTATGTCTTTAAGGAAGAGCGGTTGATTGAATTTGATGGGGAACTGGCCACAGA
TGTCTTGGTGGAATTCCTCTTGGATGTGCTAGAAGACCCTGTGGAGGTCATAAACAGCAAGCTGGAGCTTCAGGCCTTTGACCGAATCG
ATGAGGAAATCAAACTCATTGGCTACTTCAAAGGAGAAGACTCTGAACATTACAAAGCATTTGAAGAAGCTGCTGAACACTTCCAGCCT
TACGTCAAGTTCTTTGCCACCTTTGACAAAGGGGTTGCCAAGAAGCTAGGTCTGAAGATGAATGAGGTGGACTTCTATGAACCGTTTAT
GGATGAGCCTGTTCACATCCCTGATAAGCCTTACTCAGAAGAGGAGCTGGTTGAATTCGTGAAGGAGCACAAAAGAGCCACCTTGCGGA
AGCTGCGCCCAGAGGACATGTTTGAGACATGGGAGGATGACATGGAGGGAATTCACATCGTGGCCTTTGCAGAAGAAGACGACCCAGAT
GGGTTTGAGTTCCTGGAAGTCCTGAAGCAGGTTGCCAGGGACAACACCGATAATCCCGACCTGAGCATTGTCTGGATTGACCCCGACGA
CTTCCCTCTGCTCATCACCTACTGGGAGAAGACCTTCAAGATCGACCTGTTCAAGCCACAGATCGGGGTGGTGAACGTCACGGACGCTG
ACAGCGTCTGGATGGACATCAGAGATGATGATGACCTGCCCACAGCAGAGGAGCTGGAGGACTGGATAGAAGATGTGCTTTCTGGGAAG
ATAAATACCGAAGACGATGATGATGACGATGATGATGATGACGATGATGATGACGACGATGATGACGACGACGATGATGATGACAACGA
TGACGATGACGACGATGACGATGACGATGACGACGATGATTAACTGTGACTGTGCAGTTTGACTTGTGGGGGCCCAGAGCCCTCCCGCT
GCGGCAGGTCCCTCCATTGGAAGACCTGTGCTTGAGCGTCAGCAAGCACCGCTGCTCTTCTTTGCCCCCTGCCCTGCTCCCCCTGCCCC
TGCCCCTGCCCTTGCCGGGAACCCCCCAGCCTGATTCTCAGTGGTGCTGAGCCACCAGGACTGCCCCTGCAGGTACCCGGCACCGCTGC
AAATCATGCCTCCTTAGGAAGGACAATAGGAATCCGCAGGGAATATGCCCCAAGTGTCCCAGGGTGGGAGAAAAGTGCCTGCCTGCTCA
CTGCCAGAGGGACGCAGGAAGCCAAGTTGTGTTTCCATGCTCATCGGCCCAGCACTTAACATCGAAAGACCAAATCTCACTTCAAGGCG
GAGGGAAGGCCACTGTAAACTTTAGTCCTTTTATTAAACACAACGCCCTTTGCAGCTTACTCTGCCAAGACTCAACCCTGCAGGTTCTG
CTGCAGGAGTCTGTAGCTGCTCACTGACACGTTTAGAGCAGCAAAAAGATTTGGGAAAGGAGAAGGGTTTTAGGGATTCTATTTAAGTT
TTCAGTGATTCCATATAGTGACAGGATAGTCTTCTCTGGCAGCGCAATGCAAACCAACCACCACAGTCCCTGTCTGAACCTCTTCTTGA
AAGCTCAGGCCAAAGCTAGGGTAAGTGCAGTTAGTCTGATATGGGACATCAGCGTTCGTGAGTTCCTGAACGGCTGGAAAGGCTGATCC
AGGTCTGAGCCCAACCTCTTGAGGCTGAAGATCATGGACCAAATCGTTGGCCTTAACCCCAAACAACACCAGAGCAGACTCCAGGCCAG
TTGCAAACATCTGCAACCAGGTCTGGGCTGGGCCTACCTTCCCTTTCAGGTGAACAGATTGAAGGTGGTGCACATGAGACAGTGCCAGT
GAAAACATTCTTGGGGGCCCACCCTGAAGCCCTCCTATTGCCCAAGACCTTCCAGCTTTGGCTGACTTTCAACCCATAGCCTACAGAGC
GTCTTTTCCCATGACCAAGATGCTGGGAGGGAATGCATCCATCCAAACACATTAGATCCTGGACAACATGCGTTAGGCAATGAGCAGCG
AAAGCTGTTTGGCTTTTACATATTCAGTTGCACAGTTACTCCCTGCTTAGAGACTCATGGTAGCCCATGAGAAGCCTCTTTTGACTGCA
GACTGAGAAGGAGCTCATAGTCAAGGTGGTCACTCTCAAAGCAGAGGGTCAGCAACTCCGTCCTGGTGAGTGCAGCACCATCCATGTGC
AGGTCACTGCAACAGCGTTAGGTCTCCCTGCTTTCCCATGCCAGTAGATTTCAGTTGTCTTAGCTTCTTAGATTTGACAATAGTGCAAC
GAATGGAAAGTTTGGAGATAGTTTTATCTTTATAAATACATTTATAAATATGTTCTCCAGTTGTCCAGACTTGTTAGCATAGGTTAACA
TGTCCTTTCTTGCTACAGGTGAAAACCATTGTCCTGACCTTTCTTATACTGGCTTATGTCAGAGGTAAGTGATGTTAAAGGGGAAATAA
CAGTGTGAGGGACAGGAGAAGCAGAGCTCCTTGTACAAATGCGACTCAGCCTGAGGAGACACGGGGTTTACACATTATACGTTTCCTTT
ATCAGATGAACTGCAAAGCAATATTTTATAGATTTGTTTTCCCCTGCCTGTTGATCAGTCTCTTTGACTCTGTCTCTACATTAGCTGAA
ATAGATATTTTAAACTACTGGTATAACAAAGCTGGAGTACTGATATATTCCTATGTGTACTTAGCCCCTTGGTGCAAAGTGCCAGCATG
GACAACGCACAGCTGGGTATCTATTTACCTCTATGTATATGAACAAATACTGTGCTTATATGGGATATGACCATAGGCCACATTAGCTC
CTCACTGAACATCACTCAACCCGTGCTTTTAAGACACAGCCTATTAGAAGAAGAGACAGAACAGCAAGGCATCCCCAGGTCAACTCTTT
GCCTCTTCTTGCTTCTATATGATGTGTGGAAGACTGAAACCAAGTGTGATTGGGGAGACCATCCACGCATGAGCAACTCAGTCCCGTGC
CAGAGGACCTGGTGGAGACTTTCAGAAGCCTGGAAATGCAGGGTGCTCTCAGCCTGGAGGTGCCCTCCTCTCCTCCAATCACATCCTCC
TCTCTCCTGGCCAGAGGGGTGCCCAGCATCCTCCTCCTGCTCTTTCCTTTCAGCTGCTCACTGCACAGGTTTGCTGGAGGACCTTGGCT
CAGAGAGAGGGGCTCAAGGTCTCGTGGATGCGCGTTGGTATGTAGCAAGAGCAAGCAAACCTAGCCATTTAGGAGTCTCCCAGTCTCTG
ATTAAGGGATAAACACTCAGCTGAGGAAAGGACTTGTTTACCTTGGGGCTATTCTCAGGAGTAAAATACTCACAGTTTTACCCTGCATA
AGGTAACAGTTTGCACAATCCCTGCAGCACCGCACTGGGCACTGCTAAGTGCTTTGAAGTTCTACAAGCACAAGAGTTGCTCTGCTGCC

TTTCCTAACTGGCACTGTCTCTGGACACCCTGATTGCCTGAATACTTTCTCTATAGCTTCCACTGTGGAAGCCTGTCTATTCTGAGGGT
TCCCTCTCTAGATGCTCTGAGAGGCAAGACTGACACAGGCAGACCCTGCAAAGGACATTACAGATGTGTTCAAGCGCACGGCTGTTTCA
CAGGCACATACACATTTCAATACACCAAGCAACAGTGCTCGGGGGGGGGAAAGAATAATGTGGTGCTTAGGCTTATACAGAGGCCACTG
AGGCTGAGCCGACGTCAGTGGTTTATGGCTTCTCCTGAGTTAGATGAGAAAGGGCTAATCCTACAGCTGCCACTCCTCATGGGGCCAGC
GCAGTCTTTGATGGGGCCACGCTTTCTCTGCTCCCTTACTCCCCCAGTATGGTTTTCTTCCTTGTCTTTGCCCAAGAATGGGATTTTTT
TATTACTTGCTTTAGGGGTAAACACATAAAAATTGAGGAACCTTTGCAGCAGAGGAAGGATTGCAGCAATAAAGATGAGTGCTCAGCAC
AGCCTCTCCCATTCACCCCTAACACCTTCTCTGCGCTGTGTTTGTAGCCGCTGAACCACTGAATGAGGCAATACCCCTTGCGAAGGAGG
TGGCAACTTGAACTTGAGTGTCTTCCTGCAGGGACAGGGACGATAGCGGTATGTGGAGGTAACACATACAGGACCCAAGCATGGGCACT
CCTGGGCTCCTCTATCAGTAGTAGGCGCAATCGGCATTTGAAGGCGGCCAACTACACTCTGAAAGCAAAATGTCAGTAGGTTTGGATGA
GGAGTCAGCCAAAAAACCCTTCTTTGTTTAAAACAAAATTGCTGTGGGCAGCACTGAGTGTTCCTGCAGGGAACTGTGTGAAGACTATT
CCTCTCTTAGTAAATAGTTCTTTTTCTCCTGAGTGTTAATTAGACATTCACATTAAATGAGTCTTGGTACACGTAAAGACAAGGAATTG
TATTTTGCCCTCATCCGGCTGCAAGTGCTACAAGCTGATCAATCAAAGTACCCCAAAACCTAAT

>CASQ2_Myiopsitta_monachus_CDS
ATGAAGGCGACTTGCTGGATCCTGGCAGGTCTTTACCTGCTTTCCTGCTGCAAGGCGGAAGAGGGACTGAACTTCCCCACTTATGATGG
GAAAGACCGAGTGATCGACCTGAACGAGAAGAACTACAAGCAGGCCCTGAAGAAGTATGACATGCTCTGCCTGCTCTTCCACGAGCCGG
TGAGCTCTGACAAGGTCTCTCAGAAGCAGTTCCAGATGACAGAGATGGTCCTGGAGCTGGCGGCTCAGGTCCTGGAGCCCAAGAGCATT
GGCTTTGGGATGGTTGACTCCAAGAAGGATGCCAAACTTGCCAAGAAGTTAGGCTTGATTGAAGAGGGAAGTCTCTATGTCTTTAAGGA
AGAGCGGTTGATTGAATTTGATGGGGAACTGGCCACAGATGTCTTGGTGGAATTCCTCTTGGATGTGCTAGAAGACCCTGTGGAGGTCA
TAAACAGCAAGCTGGAGCTTCAGGCCTTTGACCGAATCGATGAGGAAATCAAACTCATTGGCTACTTCAAAGGAGAAGACTCTGAACAT
TACAAAGCATTTGAAGAAGCTGCTGAACACTTCCAGCCTTACGTCAAGTTCTTTGCCACCTTTGACAAAGGGGTTGCCAAGAAGCTAGG
TCTGAAGATGAATGAGGTGGACTTCTATGAACCGTTTATGGATGAGCCTGTTCACATCCCTGATAAGCCTTACTCAGAAGAGGAGCTGG
TTGAATTCGTGAAGGAGCACAAAAGAGCCACCTTGCGGAAGCTGCGCCCAGAGGACATGTTTGAGACATGGGAGGATGACATGGAGGGA
ATTCACATCGTGGCCTTTGCAGAAGAAGACGACCCAGATGGGTTTGAGTTCCTGGAAGTCCTGAAGCAGGTTGCCAGGGACAACACCGA
TAATCCCGACCTGAGCATTGTCTGGATTGACCCCGACGACTTCCCTCTGCTCATCACCTACTGGGAGAAGACCTTCAAGATCGACCTGT
TCAAGCCACAGATCGGGGTGGTGAACGTCACGGACGCTGACAGCGTCTGGATGGACATCAGAGATGATGATGACCTGCCCACAGCAGAG
GAGCTGGAGGACTGGATAGAAGATGTGCTTTCTGGGAAGATAAATACCGAAGACGATGATGATGACGATGATGATGATGACGATGATGA
TGACGACGATGATGACGACGACGATGATGATGACAACGATGACGATGACGACGATGACGATGACGATGACGACGATGATTAA

>CASQ2_Myiopsitta_monachus_protein
MKATCWILAGLYLLSCCKAEEGLNFPTYDGKDRVIDLNEKNYKQALKKYDMLCLLFHEPVSSDKVSQKQFQMTEMVLELAAQVLEPKSI
GFGMVDSKKDAKLAKKLGLIEEGSLYVFKEERLIEFDGELATDVLVEFLLDVLEDPVEVINSKLELQAFDRIDEEIKLIGYFKGEDSEH
YKAFEEAAEHFQPYVKFFATFDKGVAKKLGLKMNEVDFYEPFMDEPVHIPDKPYSEEELVEFVKEHKRATLRKLRPEDMFETWEDDMEG
IHIVAFAEEDDPDGFEFLEVLKQVARDNTDNPDLSIVWIDPDDFPLLITYWEKTFKIDLFKPQIGVVNVTDADSVWMDIRDDDDLPTAE
ELEDWIEDVLSGKINTEDDDDDDDDDDDDDDDDDDDDDDDDDNDDDDDDDDDDDDDD*

8 Aves: Passeriformes: Acanthisitti and Tyranni

8.1 *Acanthisitta chloris* (rifleman)

From: Assembled from NCBI XP_009077545 and XP_009074786 fragments of CASQ2

>CASQ2_Acanthisitta_chloris_XP_009077545_XP_009074786_protein
MKATCWILAGFYLLFCCKAEEGLNFPTYDGKDRVIDLNEKNYKQALKKYDMLCLLFHEPVSSDRISQKQFQMTEMVLELAAQVLEPRSI
GFGMVDSKKDAKLAKKLGLFEEGSLYVFKDERLIEFDGELATDVLVEFLLDLLEDPVEVINSKLELQAFDQIDDEIKLIGYFKGEDSEH
FKAFEEAAEQFQPYIKFFATFDKGVAKKLGLKMNEVDFYEPFMDEPVHIPDKPYSEEELVDFVREHKRATLRKLRPEDMFETWEDDMEG
IHIVAFAEEDDPDGFEFLEILKQVARDNTDNPDLSIVWIDPDDFPLLITYWEKTFKIDLFRPQIGVVNVTDADSIWMEITDDDDLPTAE
ELEDWIEDVLSGKINTEDDDDDEDDDDDDDDDDDDDDDDDDDDDDDDDDD*

8.2 *Ceratopipra cornuta* (scarlet-horned manakin)

From: Trinity *de novo* joint assembly of SRR18056720 and SRR18056721. CASQ1 from the complement of TRINITY_DN1667_c0_g1_i1. CASQ2 from TRINITY_DN530_c0_g1_i5.

>CASQ1_Ceratopipra_cornuta_transcript_assembly
GCTGCGGGAGCTGCGGGAGCTGCGGGAGCTGCGGGAGCTGCGGGAGCTGCTGCGGCGCTTCCCGGGGCTGGAGCTGCTCCACAGGGACC
CCCGCCACCGGGACACCCCGGACAGGGACCCCCGGGATGGGGACCCCCGGGACAGGGACCCCCGGGACAGGGACACCGGGGACGGGGAC
ACCCCTGACAGAGACCCCCGCGACGGGGACCCCCGGGAGAAGGAGCCGGTGATGGAGGTGCCGCAGGTGCCCGTGAGGGAGGTGACATC
GGAGCAGGAGCTCCAGGACTTCGAGAACGTGGAGGAGGAGCCCAAAGTGATCGGGCACTTCCAGGGACCCAGCTCGGAGCATTTCGGCT
CCTTCGCGGCGGCCGCGCGGAACCTTCACCCCCCCGTGCCCTTCTTCGTCACCTTCGACGCACAGGTGGGCCGGAGGCTGCGGCTGCGG
CTGAACGAGATTCACCTGTACCCCCCCTTCCAGGACACCCCCCGCACCCTCGGGGGGGGCCCCGCGGACCCCCCCCCAGAGCCCCCCGA
CATCGTGGCCTTCGTGGAGAACAACCAGCGGTCCCCGCTGAGGAAACTCAAACCCCAAACCCCGCTCGAGACCTGGGAGGAGGCGGCAG

GAGGGACCCTCGTGGTGGCCTTTGCGGAGGGGGATGAGCCGGATGGCTTCGAATTCCTGGCGATCCTGAAGGACGTGGCCCGGGACAAC
CCCGAGCTGCGAATCCTCTGGATCGACCCCCACGACTTCCCCACGCTCGTCCCTTACTGGGAGGAAATTTTCGACCTCGACCTTTCCCG
GCCCCAGCTCGGGGTCATCGACGGCACCGACGGTGCCGGGGTGTGGCTGGAGATGCAGCAGCAGGAGGAGGAGGAGGAGGAGGAGGAAG
ATCTGCCGGGGCCGGGGGAGGTCCAGGAGTG

>CASQ1_Ceratopipra_cornuta_CDS_partial
GCTGCGGGAGCTGCGGGAGCTGCGGGAGCTGCGGGAGCTGCGGGAGCTGCTGCGGCGCTTCCCGGGGCTGGAGCTGCTCCACAGGGACC
CCCGCCACCGGGACACCCCGGACAGGGACCCCCGGGATGGGGACCCCCGGGACAGGGACCCCCGGGACAGGGACACCGGGGACGGGGAC
ACCCCTGACAGAGACCCCCGCGACGGGGACCCCCGGGAGAAGGAGCCGGTGATGGAGGTGCCGCAGGTGCCCGTGAGGGAGGTGACATC
GGAGCAGGAGCTCCAGGACTTCGAGAACGTGGAGGAGGAGCCCAAAGTGATCGGGCACTTCCAGGGACCCAGCTCGGAGCATTTCGGCT
CCTTCGCGGCGGCCGCGCGGAACCTTCACCCCCCCGTGCCCTTCTTCGTCACCTTCGACGCACAGGTGGGCCGGAGGCTGCGGCTGCGG
CTGAACGAGATTCACCTGTACCCCCCCTTCCAGGACACCCCCCGCACCCTCGGGGGGGGCCCCGCGGACCCCCCCCCAGAGCCCCCCGA
CATCGTGGCCTTCGTGGAGAACAACCAGCGGTCCCCGCTGAGGAAACTCAAACCCCAAACCCCGCTCGAGACCTGGGAGGAGGCGGCAG
GAGGGACCCTCGTGGTGGCCTTTGCGGAGGGGGATGAGCCGGATGGCTTCGAATTCCTGGCGATCCTGAAGGACGTGGCCCGGGACAAC
CCCGAGCTGCGAATCCTCTGGATCGACCCCCACGACTTCCCCACGCTCGTCCCTTACTGGGAGGAAATTTTCGACCTCGACCTTTCCCG
GCCCCAGCTCGGGGTCATCGACGGCACCGACGGTGCCGGGGTGTGGCTGGAGATGCAGCAGCAGGAGGAGGAGGAGGAGGAGGAGGAAG
ATCTGCCGGGGCCGGGGGAGGTCCAGGAGTG

>CASQ1_Ceratopipra_cornuta_protein_partial
XLRELRELRELRELRELLRRFPGLELLHRDPRHRDTPDRDPRDGDPRDRDPRDRDTGDGDTPDRDPRDGDPREKEPVMEVPQVPVREVT
SEQELQDFENVEEEPKVIGHFQGPSSEHFGSFAAAARNLHPPVPFFVTFDAQVGRRLRLRLNEIHLYPPFQDTPRTLGGGPADPPPEPP
DIVAFVENNQRSPLRKLKPQTPLETWEEAAGGTLVVAFAEGDEPDGFEFLAILKDVARDNPELRILWIDPHDFPTLVPYWEEIFDLDLS
RPQLGVIDGTDGAGVWLEMQQQEEEEEEEEDLPGPGEVQE

>CASQ2_Ceratopipra_cornuta_transcript_assembly
TGCAGAGTGCCAGCAAGTCCCTCAATAGTGTCATATCTGTCCTTTTTATTCATTGCAGGGTTTATTTTTAGCCTGAAACAACTGCTTCC
TAAAAATGGAGTTCCTAATGACACGGGAGCTGGACACAGCTATGTAAGGTATCCAGGGCTTGGCCTCACAGCTTCTCCTGTCTGTCTCT
CTCTTTGTTGTGAGTCCAGGACAGCAAAGTTTGCTGCTCCCCACCAGCCTTGCAGTGTTTGGGCTGATCACCAGGAAGGCAGCAGAAAG
AGAAACTCAGTTCAAACTTCACCTGAAGCCTTGGTCTCCCCCTCTTCCCAAATCAAGAAAGAGAGGGACATTTACCCACCCTTTGCTGC
ACTCCAAGGGCACCCCTATGAAGGCAACTTGCTGGATCCTGGCAGGTTTTTACCTGCTTTTCTGCTGCAAGGCAGAAGAGGGACTGAAC
TTTCCCACTTACGATGGGAAAGACCGAGTGATCGACCTGAACGAGAAGAACTACAAGCAGGCTCTGAAGAAGTATGACATGCTCTGCCT
GCTCTTCCACGAGCCTGTGGGTTCTGACAAGATCTCCCAAAAGCAGTTCCAGATGACAGAGATGGTCCTGGAGCTGGCAGCTCAGGTCC
TGGAGCCCAGAAGCATCGGTTTTGGGATGGTGGACTCCAAGAAGGATGCCAAACTTGCCAAAAAATTAGGCTTGCTTGAAGAGGGAAGT
CTCTATGTCTTTAAGGATGAGCGGTTGATCGAATTTGATGGGGAACTGGCCACAGATGTCTTGGTGGAATTCCTCTTGGATTTGCTAGA
AGACCCCGTGGAGGTCATAAACAGCAAGCTGGAGCTTCAGGCCTTTGACCAGATCAATGATGAAATCAAACTCATCGGCTACTTTAAAG
GAGAAGACTCTGAACATTTCAAGGCATTTGAAGAAGCTGCTGAACAATTCCAGCCGTACATCAAGTTCTTTGCTACTTTTGACAAAGGG
GTTGCCAAGAAGCTAGGTCTGAAGATGAATGAGGTGGACTTCTATGAACCATTTATGGATGAGCCTGTTCACATCCCTGATAAGCCTTA
CACAGAAGAGGAGCTGGTTGAATTTGTGAGAGAGCACAGAAGGGCCACCTTGAGGAAGCTGCGCCCAGAGGACATGTTTGAGACGTGGG
AGGATGACATGGAGGGTATCCACATCGTAGCCTTCGCTGAAGAAGACGACCCAGACGGTTTTGAGTTCCTGGAAATTCTGAAGCAGGTT
GCCAGGGACAACACCGATAATCCTGACCTGAGCATTGTCTGGATTGACCCTGATGACTTTCCTCTGCTGATCACTTACTGGGAGAAGAC
CTTCAAGATCGACTTGTTCAGACCACAGATCGGGGTGGTGAATGTCACAGACGCTGACAGCATCTGGATGGACATCAGAGATGATGATG
ATCTGCCCACAGCTGAGGAGCTGGAAGACTGGATAGAAGATGTGCTTTCTGGGAAGATAAATACTGAAGATGATGATGATGACGACGAT
GACGACGACGATGATGACGATGACGACGATGATGACGATGATGATGACGACGATGATGATGACGACGATGATGACGATGACTAACTGTG
ACTCTGTGCAGTTTGACTTGTGGGGCCCGAGAGCCCTCCCCCTGAGGCAGGTCCTTCCATTGGAAGGCCTGTGTTTGAGCAAGCACCGC
TGCTCCTATTTGCCCTCTGCCCTGCTCCCCCTGCCCCTGCCCTTGCCGGGACCCCCTGGCCTGATTCTCAGTGGTGCTGAGCCACCGGG
ACTGCCCCTGCGGTGGGCAGTGCCTGCAGGCACCCAGCACCACTGCAAATCATGCCTCCTTAGTAAGGACAACAGGAATCTGCAGGGAA
TCTGCCCCGAGTGTCCCAGGGCAGGAGAAAAGTGCCTGCCTGCTTGCTGCCAGAGGGACGCAGGGAGCCAGTTTGTGTTTTCCATGTTC
ATCAGCCCAGCACTTAACATCCAAAGACCAAATCTCCCTTCAAGACATTGAGAAGGCCACTGTAAAGTTTAGTCCTTTTATTAAACACA
ATGCCCTTTGCAGATACTCTGCCAAGACCACTCAATCCTGCAAGTCCTGCTGCGGGAGTCCCTAGCTAACGCTAGGAGCAGCAAAATGC
TTTGGGAGGGGAGGAAGGTTTTAGGGATGCTATTAAAGTTTTCAGTGATTCCACATAACAACAGGATATTCTTGTCTGGCAGTGTGATG
CAAAACCAGTCACCATGCCCTCTGTCTGAACCTCTTTTCTTGAAAGCTCGGGCCATGGCCAGGGAAGTGCAGATGGTCTGATAGAAGGC
ATAAAAGCTCACAAGTTCCTGAACTGCTGGAAAGGCTGATCCAGGTCTGAGTCCAAACTCTTGAGTTTGAAGACCATGGCACTGAGTCA
AGCCAATGGAAACATCCATAGGGGCCCACCCTGAAGCTCTCCGATTCCCCATGACCTTCTGGCTTTAGCTGACTCTTCACTCAAAGCCC
ACGTAGCACCTATTCCCATGACCAAGATGTTGGGAGGGAACACACCCATCCAACTACATTTGATATTGGATGAGGTACGTCGGGCAATG
AGCGGCGAACGGATTCAAGTTGTTTGGCTTTAAGTATTTAGTTGCACAGTTACTCCCTGCTTAGAGACTTGGATGTCCCATAGCAGCCT
GTGAGAAGCCTCTTTTGACTGCAGGAGCTCAGGGTGCCAGTTTGCAGTCAAGGGGGTCGCTCACAAAGCAAAGGGTCTGCAACACAGTC
CTGGTGAGTGCAGCACCATCCACATGCAGGTCACTGTGGCAGCATTAAATCTCCCTGCTTTCCCATGCCAGCAGATTTCAATTGTCTTA
GCTTATTAGGTTTGACAATAGTACAATGAACAGAGAGTTCAGAGATAGTTTTATCTCTATATACACATTCATAAGGATGTTCTCTGGTT
GTTCACGCTTGTTAGCATAGGTAGGGTATGCATCTCCTTTCTTGCTACAGATGGAAAACATTGTCCTGACTGTTCTTTTCCTGGCTTAC
GTCAGAGGTAAGTGATATTAGAGGAAATAATAGTGTGAGGGACAGGAGAATCAGAGCTCATTACACAAGTGTGAATCAGCCTGATGAGA
AACAACTATATATTTCCTTTATCAGATGAACCACAAAGCAAGATTTTATAGATTTTTTTTTTGCTGCCTGTTGATCAGTCTCTTTGACT

CTCCTCTACATTAGCTGAAACAGAATTTTGAAACTACTGGTATAAAAAAGCTAAAGTATTGATATATTCCTATGTATGCCATATCCCTT
GGTAGAAAATGCCAGTATGAACAACACATGGCTGGATATCTATATATCTCTATGCATATGAATGAATACTGTGCTAATATGGCATATGA
CTGTAGGCCAAGTTAGCTCCTCATTGAATATAAAACATTCAACATGTGCTTTTAAGACACAGCTATTAGAAGAATAGACAAGGCAGCAA
AGCCCCCAGGCCAACTTTTCATGCTTTCTTGCTTTCTTATGATACATACGCACTGCCTGGAAGACGAACCAACTTTTTGGGGGAAACCA
TTCACCTGGTTGCTCTGGGGTGACGAACAACTTTCAAAAGCCTTGAAATGCAGAGTACTCTCAGCATGGAGGTCCCCTTCTCCTTTTCT
CTCACATTTGCCTCTCTCCTGGTCAACAGGGTGCCCAGCACTCTCCTTCTACTCTTTCCTTTCAGCTCTTTCCTCAAGAGGTTTGCTGG
AGGACCCTGGCTCAGAGAGAGAGGGAGCAGGCAGGAATCAAGGTCTCATTCATGCACACTGGTAAAGAGCAAGAAGAGGCAAAACTGGC
CATCTAGTGGTCTCCCAGTCTGCAATTAAGGGACAACTACTCACCTGAGAAAAATACATGCTTATTTTTACCTTGGGGCTATTCCTAGG
AGTTAAACATGCCCAGTTTCACCCTTCATAAGGCAACAGCCTGCACAGGCCCTGCAGCTCCATATCAGTCACTCTAAACACGAACAATA
AACGAAGAGCTGCTCTACTGCCTTTCCTACCTAGCACAGTCTCTGGACATCTCAACTACCTGGATACTTTCCATGTAGCTCCCCTCCTG
CTGTGGATGCCTGTCTATTCTAATGTGTCCTTCTCCAGACGCACTGAGAGGCACAACTTACCCAGGCAGAGCAGGCAGACCCTGAAAAT
AACGCTATGGACACGTTCAAGTGCACAGGTGTTTCACAGGTATTCATATATTTCAAAGCAACAGAGCTCTGGGGGGGAAAACAGAATAA
ATAATGTAGAAGTGTAGAGGTTGAGCTCATATGGGGGCTGGTGGACTGGTGCCACTGTTTTATGGACAGTTTTCCTTGAGTTATGAAAG
GGTTAATGCTACAGCTGCCACTCCTTCGTGGTGCCAGTGAAGTCCTTGATGGGGCTGCCCTTTCTTTCTTGCCTAATCCCCCCAGTACA
ATTTTCATCATTCTCTTTGCCCACAAACAGGAACTGTTTATTATCAGCTTTTAGGACAAACACATGAAAAGTTAAGAAATGTTGCAGCA
GAGGAAGGATTCCAGCAATGAGGATGCGTGGGCAGCAAACCCATTTACCTGTAACACTTCATCTGCCCCAAGATCCTAGCGACCAAACT
GCTGAATGAGGCAATACCTCTTGTGAAGGAGATGGCAGCTTAAACTTGAGTGACTTCCTTGCAGGGATCAGTAAAACAGTGGTATGGGG
AGGACACACAGGACTCCAGGCACAGGTATTCCCGGGCTCCTCTGTCAGAACTAGGTACAACTGGCATTTGAAAGGCAGTGGTGGCCCTG
AGGAACTAAGTCAGCCTAACTCCCCCACTACACACTGACAAGCAAAAGGTTGATTTGGGTGAGGAACCTGCAAAAAACCCTTAATTAAA
AAAAAAAAAAAAGAAATTGTGTACATGAGCAGCACTGAGTGTTTCCGCAAGCACATCTGTTAAGACTGTCCCTCTCTTCAAGAATAGTT
CTTTTTCTCCAGCATGTTAATTAGACATATTAAATGAGTCTTAACACACACAAAGACAAGTAATTGGATTTTTCCCTCATCCAACTATG
AGTACGTCAAGCTGATCAGTTACTTCATAGAATCATTCAATCATTTTTGTTGGAAAAGACCCCTAAGATCATTGAGACCAACCATTAAT
CCACCACTGCCAAGTAAGTACCCAAAATAACCCAAAAGATAGTCTTACCAGCCCCCAATGACAGTAAGGACCCCACCTCCCCACAGGTG
ATACATGGCAAGAACACCAAGTCATACACCACCACCTCCTCCCAGTCACCAGACACCGGGCCAGAGGCCACAGCCGAGGGATTTGTTTG
ATGTGCTGTACCACAGGCCACTCTGTGCTGCTCATGGGCAACAACCAAGGCAGATGGAAAGCCACCAGGACCCACATCCAGCAGCTATC
ATACTGCAGCATGGAGGAGAAAATTTCTGCAGTGGGCCCAAGGTCACTGACGTAGAGGCTCTGTGAACCCAAAACTGCTCTGAGCTTAG
GTCAACCATTTGGGAAACAGTTCCAGAAGGAGGGGACAACAGAGGAACTTTGGAAACAGCAGATTACTCCACGGTTTCAGGGTCAGCGG
TTTCTGTGATGTTTCTGAGGAGTTTTGAGGCTCCTGTATCTCCCCTGGGAGAGGAACGGACCCATAGTGAAACCAGAGATCTTGGCTGC
TTCCCATCCCAAAACAAAGCTGCTCAGCCAGACACTAACATGAACGTGGAGCTGAGGACACCAGCTGCTTCTTCATGTCTGTCTGCACA
TCATACCTAAAGAAATGATCGATTCATTACAGAAACCAAACAAAAAACATTGTCTTTTATTCCCAGGAAAATAATTAATTAGACAGGAA
CATATCACTTCCCAGCTGCCACTGACTTGCAGTCCTGTGAAAAGAGGCCAAGATTGCATCTGGCTTCATCTCCACTGCAGCAAAGCTTT
GAGCTTTGGCTTTGAGGAAGTCTCACACAGAGCCAGTTGCACACCAACGCCTGGACCTGGGTGCCTGAAAAGGAGGAGCAGGTAAACAG
CCAACAAGCACAAAGGCCCTCCGAGACAAAGCACCCTGCCTCCTGGCTGCTTTTACACCCCAGGTATGTCTATGCCATGCAGTGCAAGA
CCATCTGCTAGAAACTGGAAGCAGCAGAGAGCAGCAGGCAGGATAACTGACCTGAGGCTGCACCAGCAGGACTGTGTCCTACCTGGGAC
CCAAGACCGTGTGTGTCAACCCACTGCACTGCAGCATCCCCTGCTCCCTTTTTGCCACTCCCTTGTACCACTGGCATCTAGCAAAGCAG
CGCTTCGATGGTCAGTACCCCTGGAGGACAGCCATGGGATGGCTCTCATACCTGTTTGGCAGAAGATGCTGTCTAATGATGACACAGGT
CACGAGGCAGGTCCAGTGCAGAGCCAGCCCTCTCGGCCTTGCTCCCTGAACACTCTCTGCCAGGTTTAAAAGGTGCTAAACTATTTAAA
GGGAAAAAAAAAAAGCTACAGAACTTTCTTGCAGCTTGAAGATCCATTTCTGGAATCAGATTACAAACAGAGACGGAAGCAAAGATCCTT
CCGGTGCCTCACTACCCCCTCATGCTTTCTCTTGCCAAGCCCCTGTCAAAGGGCATTGTCCCTAATGCTATGGAAAATAAGAAGAAAAC
AAGCCTTTTCACCTTCTTTTCATCCCTCTTGAATTTCTACTCTTCTGATCCTCACTGGCTCCCCTACTTTATTTTCTCTCTCCATTTCT
ATTTAACCTGTCTTCCTCTTTTTCTTAACCCACCCCCACAGCATATGGTACTGAGGGTCACTTAAGGACACAGCCCTTCCCTGCCCCTA
CTGCTTGGGCTCATCCCAGCCCAAGGAGACAGCAGCCTCCCCCACACCACTGCCAGGAACTGGGACGGACAACAACTGGGACGGACAAC
ATTTCCTTTCTGAGGGGAAAGACAGTCGAACAAGGCTGTCACTGCCTTTCATTTTGAGGGGGACAGGTGGGACATGCTCAAGGAGAAGC
CATTTCACCAGCCTGGGCACTGAGGAAAAAACAGAAATTTTTTTTTGGTGGGCATCTTTTCCCATTGTCCCAAAATCCAGATGAGGAAC
CATCACCCTGCAATCAAGCCCTGACTGTTGCCACCGTGGAAAGAGACCAGTAACCCCCCGTGACACCAGCGATCACACCGCTTCTGCTC
ACCATCTGAGCAGAAGACAAGTGTCATGGCTTCACATACAAGAAATAAGCCTCCAGGGCTAAATGAGCTGAGTTTGCTGGAGGGGGTAT
CCCATACTTTAACAGCAGCCAGAAAGCCAACTCCCTGCCGGAGACACAACCAGTGGTTGTCCTGACCGAGTTCTCCTTCAGTTGTGGGT
GGTAAATGACTTAAATAGCCATGGGCAACCTCATCTTCTGCTTTTGGATTTGGAGGAAGCCCAGCTTGAATCAGGTCCTTCCCTCAGTA
ACACAGGTAAAAATAAAGTCATGTTTGACCAGAGAGCAGCCCTGAATTTTGCCTGGTGTTAGATTTTTGCTTTAAAATTCAGTCCAACA
CGTCCAAGGCCTGCTCCTGCTAGTGACAAAGAGAGAGGTTATTGAACAACAAAAGTAACTAAATGTTTGAGGAACATTTCAGTCCCCAC
AAAATGAAATTGCTGAAGGAATTTGCACAGCTTCCCACAGTCTAGTTACTGAATTACTAAACCAGTTTCTCACAACCACTTACAAAGCA
GAATTATAATTAACTGAAGGATCTG

>CASQ2_Ceratopipra_cornuta_CDS
ATGAAGGCAACTTGCTGGATCCTGGCAGGTTTTTACCTGCTTTTCTGCTGCAAGGCAGAAGAGGGACTGAACTTTCCCACTTACGATGG
GAAAGACCGAGTGATCGACCTGAACGAGAAGAACTACAAGCAGGCTCTGAAGAAGTATGACATGCTCTGCCTGCTCTTCCACGAGCCTG
TGGGTTCTGACAAGATCTCCCAAAAGCAGTTCCAGATGACAGAGATGGTCCTGGAGCTGGCAGCTCAGGTCCTGGAGCCCAGAAGCATC
GGTTTTGGGATGGTGGACTCCAAGAAGGATGCCAAACTTGCCAAAAAATTAGGCTTGCTTGAAGAGGGAAGTCTCTATGTCTTTAAGGA
TGAGCGGTTGATCGAATTTGATGGGGAACTGGCCACAGATGTCTTGGTGGAATTCCTCTTGGATTTGCTAGAAGACCCCGTGGAGGTCA
TAAACAGCAAGCTGGAGCTTCAGGCCTTTGACCAGATCAATGATGAAATCAAACTCATCGGCTACTTTAAAGGAGAAGACTCTGAACAT

TTCAAGGCATTTGAAGAAGCTGCTGAACAATTCCAGCCGTACATCAAGTTCTTTGCTACTTTTGACAAAGGGGTTGCCAAGAAGCTAGG
TCTGAAGATGAATGAGGTGGACTTCTATGAACCATTTATGGATGAGCCTGTTCACATCCCTGATAAGCCTTACACAGAAGAGGAGCTGG
TTGAATTTGTGAGAGAGCACAGAAGGGCCACCTTGAGGAAGCTGCGCCCAGAGGACATGTTTGAGACGTGGGAGGATGACATGGAGGGT
ATCCACATCGTAGCCTTCGCTGAAGAAGACGACCCAGACGGTTTTGAGTTCCTGGAAATTCTGAAGCAGGTTGCCAGGGACAACACCGA
TAATCCTGACCTGAGCATTGTCTGGATTGACCCTGATGACTTTCCTCTGCTGATCACTTACTGGGAGAAGACCTTCAAGATCGACTTGT
TCAGACCACAGATCGGGGTGGTGAATGTCACAGACGCTGACAGCATCTGGATGGACATCAGAGATGATGATGATCTGCCCACAGCTGAG
GAGCTGGAAGACTGGATAGAAGATGTGCTTTCTGGGAAGATAAATACTGAAGATGATGATGATGACGACGATGACGACGACGATGATGA
CGATGACGACGATGATGACGATGATGATGACGACGATGATGATGACGACGATGATGACGATGACTAA

>CASQ2_Ceratopipra_cornuta_protein
MKATCWILAGFYLLFCCKAEEGLNFPTYDGKDRVIDLNEKNYKQALKKYDMLCLLFHEPVGSDKISQKQFQMTEMVLELAAQVLEPRSI
GFGMVDSKKDAKLAKKLGLLEEGSLYVFKDERLIEFDGELATDVLVEFLLDLLEDPVEVINSKLELQAFDQINDEIKLIGYFKGEDSEH
FKAFEEAAEQFQPYIKFFATFDKGVAKKLGLKMNEVDFYEPFMDEPVHIPDKPYTEEELVEFVREHRRATLRKLRPEDMFETWEDDMEG
IHIVAFAEEDDPDGFEFLEILKQVARDNTDNPDLSIVWIDPDDFPLLITYWEKTFKIDLFRPQIGVVNVTDADSIWMDIRDDDDLPTAE
ELEDWIEDVLSGKINTEDDDDDDDDDDDDDDDDDDDDDDDDDDDDDDDDDD*

8.3 *Ceratopipra mentalis* (red-capped manakin)

From: Trinity *de novo* joint assembly of SRR18056698 and SRR18056699. CASQ1 from TRINITY_DN6650_c0_g1_i3. CASQ2 from TRINITY_DN526_c1_g1_i1.

>CASQ1_Ceratopipra_mentalis_transcript_assembly
CGTGTGTCCACCCCGGTGTCCGTCTGTCCGTCCGTGTGTCCGTGTGTCCGCCCCGGTGTCCGTGTGTCCACCCCCGTGTCCGTGTGTCC
ACCCCGGTGTCCGTGTGTCCCCCCGTCCCCGTGTCCGTGTGTCCATCCCTGTGTCCCCCTGTCCCCGTGTCCGTGTGTCCCCCCCGTTG
TCCCGATGTCCGTCTGTCTGCGGGCTGTGGTCGCTCTGTCCGTTCTGTCCGTGCTGTCGCTGTGCCCGGCGGTCCCGGGGGTCTCAGCC
CGGGGGGGGCTGCACTCGCCCCCCCCCGGCGGGCGGGGCCGGGGGGTCGCCGCGACCCCGCGGGAGCTGCGGGAGCTGCGGGAGCTGCT
GCGGCGCTTCCCGGGGCTGGCGCTGCTCCACAGGGACCCCCGGGACGGGGACCCCCGGGACAGGGACCCCCGGGATGGGGACACTCCGG
ACAGGGTCACCCGAGAATGGGACCCCCAGGAGAAGGAGCCGGTGATGGAGGTGCCGCAGGTGCCCGTGAGGGAGGTGACATCGGAGCAG
GAGCTCCAGGACTTCGAGAACGTGGAGGAGGAGCCCAAAGTGATCGGGCACTTCCAGGGACCCAGCTCGGAGCATTTCGGCTCCTTCGC
GGCGGCCGCGCGGAACCTTCACCCCCCCGTGCCCTTCTTCGTCACCTTCGACGCACAGGTGGGCCGGAGGCTGCGGCTGCGGCTGAACG
AGATTCACCTGTACCCCCCCTTCCAGGACACCCCCCGCACCCTCGGGGGGGGCCCCGCGGACCCCCCCCCAGACCCCCCCGACATCGTG
GCCTTCGTGGAGAACAACCAGCGGTCCCCGCTGAGGAAACTCAAACCCCAAACCCCGCTCGAGACCTGGGAGGAGGCGGCAGGAGGGAC
CCTCGTGGTGGCCTTTGCGGAGGGGGATGAGCCGGATGGCTTCGAATTCCTGGCGATCCTGAAGGACGTGGCCCGGGACAACCCCGAGC
TGCGAATCCTCTGGATCGACCCCGACGACTTCCCGACGCTCGTCCCTTACTGGGAGGAAATTTTCGACCTCGACCTTTCCCGGCCCCAG
CTCGGGGTCATCGACGGCACCGACGGTGCCGGGGTGTGGCTGGAGATGCAGCAGCAGCAGCAGGAGGAGGAGGAGGAGGAGGAGGAAGA
TCTGCCGGGGCCGGGGGAGGTC

>CASQ1_Ceratopipra_mentalis_CDS_partial
ATGTCCGTCTGTCTGCGGGCTGTGGTCGCTCTGTCCGTTCTGTCCGTGCTGTCGCTGTGCCCGGCGGTCCCGGGGGTCTCAGCCCGGGG
GGGGCTGCACTCGCCCCCCCCCGGCGGGCGGGGCCGGGGGGTCGCCGCGACCCCGCGGGAGCTGCGGGAGCTGCGGGAGCTGCTGCGGC
GCTTCCCGGGGCTGGCGCTGCTCCACAGGGACCCCCGGGACGGGGACCCCCGGGACAGGGACCCCCGGGATGGGGACACTCCGGACAGG
GTCACCCGAGAATGGGACCCCCAGGAGAAGGAGCCGGTGATGGAGGTGCCGCAGGTGCCCGTGAGGGAGGTGACATCGGAGCAGGAGCT
CCAGGACTTCGAGAACGTGGAGGAGGAGCCCAAAGTGATCGGGCACTTCCAGGGACCCAGCTCGGAGCATTTCGGCTCCTTCGCGGCGG
CCGCGCGGAACCTTCACCCCCCCGTGCCCTTCTTCGTCACCTTCGACGCACAGGTGGGCCGGAGGCTGCGGCTGCGGCTGAACGAGATT
CACCTGTACCCCCCCTTCCAGGACACCCCCCGCACCCTCGGGGGGGGCCCCGCGGACCCCCCCCCAGACCCCCCCGACATCGTGGCCTT
CGTGGAGAACAACCAGCGGTCCCCGCTGAGGAAACTCAAACCCCAAACCCCGCTCGAGACCTGGGAGGAGGCGGCAGGAGGGACCCTCG
TGGTGGCCTTTGCGGAGGGGGATGAGCCGGATGGCTTCGAATTCCTGGCGATCCTGAAGGACGTGGCCCGGGACAACCCCGAGCTGCGA
ATCCTCTGGATCGACCCCGACGACTTCCCGACGCTCGTCCCTTACTGGGAGGAAATTTTCGACCTCGACCTTTCCCGGCCCCAGCTCGG
GGTCATCGACGGCACCGACGGTGCCGGGGTGTGGCTGGAGATGCAGCAGCAGCAGCAGGAGGAGGAGGAGGAGGAGGAGGAAGATCTGC
CGGGGCCGGGGGAGGTC

>CASQ1_Ceratopipra_mentalis_protein_partial
MSVCLRAVVALSVLSVLSLCPAVPGVSARGGLHSPPPGGRGRGVAATPRELRELRELLRRFPGLALLHRDPRDGDPRDRDPRDGDTPDR
VTREWDPQEKEPVMEVPQVPVREVTSEQELQDFENVEEEPKVIGHFQGPSSEHFGSFAAAARNLHPPVPFFVTFDAQVGRRLRLRLNEI
HLYPPFQDTPRTLGGGPADPPPDPPDIVAFVENNQRSPLRKLKPQTPLETWEEAAGGTLVVAFAEGDEPDGFEFLAILKDVARDNPELR
ILWIDPDDFPTLVPYWEEIFDLDLSRPQLGVIDGTDGAGVWLEMQQQQQEEEEEEEEDLPGPGEV

>CASQ2_Ceratopipra_mentalis_transcript_assembly
-GTGAGGTCTGTCCCTTCACAAACCCTCCCTGCATATTCCCAGGACTGACATCTTCCACTCCCTCCCTCTTGCTCATTCCCTCTCTCTC
AGATGATGCAGAGTGCCAGCAAGTCCCTCAATAGTGTCATATCTGTCCTTTTTATTCATTGCAGGGTTTATTTTTAGCCTGAAACAACT
GCTTCCTAAAAATGGAGTTCCTAATGACACGGGAGCTGGACACAGCTATGTAAGGTATCCAGGGCTTGGCCTCACAGCTTCTCCTGTCT
GTCTCTCTCTTTGTTGTGAGTCCAGGACAGCAAAGTTTGCTGCTCCCCACCAGCCTTGCAGTGTTTGGGCTGATCACCAGGAAGGCAGC
AGAAAGAGAAACTCAGTTCAAACTTCACCTGAAGCCTTGGTCTCCCCCTCTTCCCAAATCAAGAAAGAGAGGGACATTTACTCACCCTT

TGCTGCACTCCAAGGGCACCCCTATGAAGGTAACTTGCTGGATCCTGGCAGGTTTTTACCTGCTTTTCTGCTGCAAGGCAGAAGAGGGA
CTGAACTTTCCCACTTACGATGGGAAAGACCGAGTGATCGACCTGAACGAGAAGAACTACAAGCAGGCTCTGAAGAAGTATGACATGCT
CTGCCTGCTCTTCCATGAGCCTGTGAGTTCTGACAAGATCTCCCAAAAGCAGTTCCAGATGACAGAGATGGTCCTGGAGCTGGCAGCTC
AGGTCCTGGAGCCCAGAAGCATCGGTTTTGGGATGGTGGACTCCAAGAAGGATGCCAAACTTGCAAAAAAATTAGGCTTGCTTGAAGAG
GGAAGTCTCTATGTCTTTAAGGATGAGCGGTTGATCGAATTTGATGGGGAACTGGCCACAGATGTCTTGGTGGAATTCCTCTTGGATTT
GCTAGAAGACCCCGTGGAAGTCATAAACAGCAAACTGGAGCTTCAGGCCTTTGACCAGATCGATGATGAAATCAAACTCATCGGCTACT
TTAAAGGAGAAGACTCTGAACATTTCAAGGCATTTGAAGAAGCTGCTGAACAATTCCAGCCGTACATCAAGTTCTTTGCTACTTTTGAC
AAAGGGGTTGCCAAGAAGCTAGGTCTGAAGATGAATGAGGTGGACTTCTATGAACCATTTATGGATGAGCCTGTTCACATCCCTGATAA
GCCTTACACAGAAGAGGAGCTGGTTGAATTTGTGAGAGAGCACAGAAGGGCCACCTTGAGGAAGCTGCGCCCAGAGGACATGTTTGAGA
CGTGGGAGGATGACATGGAGGGTATCCACATCGTAGCCTTCGCTGAAGAAGACGACCCAGACGGTTTTGAGTTCCTGGAAATTCTGAAG
CAGGTTGCCAGGGACAACACCGATAATCCTGACCTGAGCATTGTCTGGATTGACCCTGATGACTTTCCTCTGCTGATCACTTACTGGGA
GAAGACCTTCAAGATCGACTTGTTCAGACCACAGATCGGGGTGGTGAATGTCACAGACGCTGACAGCATCTGGATGGACATCAGAGATG
ATGATGATCTGCCCACAGCTGAGGAGCTGGAAGACTGGATAGAAGATGTGCTTTCTGGGAAGATAAATACTGAAGATGATGATGATGAT
GATGACGACGATGACGATGATGACGATGACGACGATGATGACGATGATGATGACGACGATGATGATGACGACGATGATGATGACGACGA
TGATGACGATGACTAACTGTGGCTCTGTGCAGTTTGACTTGTGGGGCCCGAGAGCCCTCCCCCTGAGGCAGGTCCTTCCATTGGAAGGC
CTGTGTTTGAGCAAGCACCACTGCTCCTATTTGCCCCCTGCCCTGCTCCCCCTGTCCCTGCCCTTGCCGGGACCCCCTGGCCTGATTCT
CAGTGGTGCTGAGCCACCGGGACTGCCCCTGCGGTGGGCAGTGCCTGCAGGCACCCAGCACCGCTGCAAATCATGCCTCCTTAGTAAGG
ACAACAGGAATCTGCAGGGAATCTGCCCCGAGTGTCCCAGGGCAGGAGAAAAGTGCCTGCCTGCTTGCTGCCAGAGGGACGCAGGGAGC
CAGTTTGTGTTTTCCATGTTCATCAGCCCAGCACTTAACATCCAAAGACCAAATCTCCCTTCAAGACATTGAGAAGGCCACTGTAAAGT
TTAGTCCTTTTATTAAACACAATGCCCTTTGCAGATACTCTGCCAAGACCACTCAATCCTGCAAGTCCTGCTGCGGGAGTCCCTAGCTA
ACGCTAGGAGCAGCAAAATGCTTTGGGAGGGGAGGAAGGTTTTAGGGATGCTATTAAAGTTTTCAGTGATTCCACATAACAACAGGATA
TTCTTGTCTGGCAGTGTGATGCAAAACCAGTCACCATGCCCTCTGTCTGAACCTCTTTTCTTGAAAGCTTGGGCCATGGCCGGGGAAGT
GCAGATGGTCTGATAGGAGGCATAAAAGCTCGCAGGTTCCTGAACTGCTGGAAAGGCTGATCCAGGTCTGAGTCCAAACTCTTGAGTTT
GAAGACCATGGCACTGAGTCAAGCCAATGGAAACATCCGTAGGGGTCCACCCTGAAGCTCTCCTATTCCCCATGACCTTCTGGCTTTAG
CTGATTCTTCACTCAAAGCCCACGTAGCATCTATTCCCATGACCAAGATGTTGGGAGGGAACACACCCATCCAACTACATTTGATATTG
GATGAGGTACGTCGGGTAATGAGCGGCGAATGGATTCAAGTTGTTTGGCTTTAAGTATTTAGTTGCACAGTTACTCCCTGCTTAGAGAC
TTGGATGTCCCATAGTAGCCTGCGAGAAGCATCTTTTGACTGCAGGAGCTCAGGGTGCCAGTTTGCAGTCAAGGGGGTCACTCACAAAG
CAAAGGGTCTGCAACACAGTCCTGGTGAGTGCAGCACCATCCACATGCAGGTCACTGTGGCAGCATTAAATCTCCCTGCTTTCCCATGC
CAGCAGATTTCAATTGTCTTAGCTTATTAGGTTTGACAATAGTACAATGAACAGAGAGTTCGGAGATAGTTTTATCTCTATATACACAT
TTATAAGGATGTTGTCTGGTTGTTCAGGCTTGTTAGTATAGGTAGGGTATGCATCTCCTTTCTTGCTACAGATGGAAAACATTGTCCTG
ACTGTTCTTTTACTGGCTTACGTCAGAGGTAAGTGATATTAGAGGAAATAACAGTGTGAGGGACAGGAGAATCAGAGCTCATTACACAA
GTGTGAATCAGCCTGATGAGAAACAAGGTTCACACACTATATATTTCCTTTATCAGATGAACCACAAAGCAAGATTTTATAGATTTTTT
TTTTGCTGCCTGTTGATCAGTCTCTTTGACTCTCCTCTACATTAGCTGAAACAGAATTTTGAAACTACTGGTATAAAAAAGCTAAAGTA
TTGATATATTCCTATGTATGCCATATCCCTTGGTAGAAAATGCCAGTATGAACAACACATGGCTGGATATCTATATATCTCTATGCATA
TGAATGAATACTGTGCTAATATGGCATATGACTGTAGGCCAAGTTAGCTCCTCATTGAATATAAAACATTCAACATGTGCTTTTAAGAC
ACAGCTATTAGAAGAATAGACAAGGCAGCAAAGCCCCCAGGCCAACTTTTCATGCTTTCTTGCTTCCTTATGATACATAAGCACTGCCT
GGAAGACGAACCAACTTTTTGGGGGAAACCATTCACCTGGTTGCTCTGGGGTGACGAACAACTTTCAAAAGCCTTGAAATGCAGAGTGC
TCTCAGCATGGAGGTCCCCTTCTCCTTTTCTCTCACATTTGCCTCTCTCCTGTTCAACAGGGTGCCCAGCACTCTCCTTCTACTCTTTC
CTTTCAGCTCTTTCCTCAAGAGGTTTGCTGGAAGACCCTGGCTCAGAGAGAGAGGGAGCAGGCAGGAATCAAGGTCTCATTCATGCACA
CTGGTAAAGAGCAAGAAGAGGCAAAACTGGCCATCTAGTGGTCTCCCAGTCTGCAATTAAGGGACAACTACTCACCTGAGAAAAACACA
TGCTTATTTTTACCTTGGGGCTATTCCTAGGAGTTAAACATGCCCAGTTTCACCCTTCATAAGGCAACAGCCTGCACAGGCCCTGCAGC
TCCATATCAGTCACTCTAAACACGAACAATAAACGAAGAGCTGCTCTACTGCCTTTCCTAACTAGCACAGTCTCCAGACATCTCAACTA
CCTGGATACTTTCCATGGAGCTGCCCTCCTGCTGTGGATGCCTGTCTATTCTAATGTGTCCTTCTCCAGATGCACTGAGAGGCACAACT
TACCCAGGCAGAGCAGGCAGACCCTGCAAATAATGCTACGGACATGTTCAAGTGCACAGGTGTCTCACAGGCATTCATATATTTCAAAG
CAACAGAGCTCTGGGGGGGAAAACAGAATAAATAATGTAGAAGTGTAGAGGTTGAGCTCATATGGGGGCTGGTGGACTGGTGCCAGTGT
TTTATGGACAGTTTTCCTTGAGTTATGAAAGGTCTAACGCTACAGCTGCCACTCCTTCGTGGTGCCAGTGAAGTCCTTGATGGGGCTGC
CCTTTCTTTCTTGCCTAATCCCCCCAGTACAATTTTCATCATTCTCTTTGCCCACAAACAGGAACTGTTTATTATCAGCTTTTAGGACA
AACACATGAAAAGTTAAGAAATGTTGCAGCAGAGGAAGGATTCCAGCAATGAGGATGGGTGGGCAGCAAACCCATTTACCTGTAACACT
TCATCTGCCCCAAGATCCTAGCGACCAAACTGCTGAATGAGGCAATACCTCTTGTGAAGGAGATGGCAGCTTAAACTTGAGTGACTTCC
TTGCAGGGATCAGTAAAACAGTGGTATGGGGAGGACACACAGGACTCCAGGTACAGGTATTCCCGGGCTCCTCTGTCAGAACTAGGTAC
AACTGGCATTTGAAAGGCAGTGGTGGCCCTGAGGAACTAAGTCAGCCTAACTCCCCCCACTACACACTGACAAGCAAAAGGTTGATTTG
GGTGAGGAACCTGCAAAAAACCCTTAATAAAAAAAAAAAAAAAAAAGAAATTGTGTACATGAGCAGCACTGAGTGTTTCCACAAGCACAT
CTATTAAGACTGTCCCTCTCTTCAAGAATAGTTCTTTTTCTCCAGCATGTTAATTAGACATATTAAATGAGTCTTAACACACACAAAGA
CAAGTAATTGGATTTTTCCCTCATCCAACTGTGAGTACTTCAAGCTGATCAGTTACTTCATAGAATCATTCAATCATTTTTGTTGGAAA
AGACCCCTAAGATCATTGAGACCAACCATTAATCCACCACTGCCAAGTAAGTACCCAAAATAACCCAAAAGATAGTCTTACCAGCCCCC
AGTGACAGTAAGGACCCCACCCCCCCACAAGTGATACATGGCAAGAACACCAAGTCATACACCACCACCTCCTCCCAGTCACCAGACAC
CGGGCCAGAGGCCACAGCCGAGGGATTTGTTTGGTGTGCTGTACCACAGGCCACCCCGTGCTGCTCATGGGCAACAACCAAGGCAGATG
GAAAGCCACCAGGACCCACATCCAGCAGCTATCATACTGCAGCATGGAGGAGAAAGTTTCTGCAGTGGGCCCAAGGTCACTGACGTAGA
GGCTCTGTGAACCCAAAACTGCTCTGAGCTTAGGTCAACCATTTGGGAAACAGCTCCAGAAGGAGGGGACAACAGAGGAACTTTGGAAA
CAGCAGATTACTCCACGGTTTCAGGGTCAGCGGTTTCTGTGATGTTTCTGAGGAGTTTTGAGGCTCCTGTATCTCCCCTGGGAGAGGAA

TGGACCCATAGTGAAACCAGAGATCTTGGCTGCTTCCCATCCCAAAACAAAGCTGCTCAGCCAGACACTAACATGAACGTGGAGCTGAG
GACACCAGCTGCTTCTTCATGTCTGTCTGCACATCATACCTAAAGAAATGATCGATTCATTACAGAAACCAAACAAAAAACATTGTCTT
TTATTCCCAGGAAAATAATTAATTAGACAGGAACATATCACTTCCCAGCTGCCACTGACTTGCAGTCCTGTGAAAAGAGGCCAAGATTG
CATCTGGCTTCATCTCCACTGCAGCAAAGCTTTGAGCTTTGGCTTTGAGGAAGTCTCACACAGAGCCAGTTGCACACCAATGCCTGGAC
CTGGGTGCCTGAAAAGGAGGAGCAGGTAAACAGGCAACAAGCACCAAGGCCCTCTGAGACAAAGCACCCTGCCTCCTGGCTGCTTTTAC
ACCCCAGGTATGTCTATGCCATGCAGTGCAAGACCATCTGCTAGAAACTGGAAGCAGCAGAGAGCAGCAGGCAGGATAACTGACCTCAG
TAATGAGGCTGCACCAGCAGGACTGTGTCCTACCTGAGACCCAAGACCGTGTGTGTCAACCCACTGCACTGCAGCATCCCCGCTCCCTT
TTTGCC

>CASQ2_Ceratopipra_mentalis_CDS
ATGAAGGTAACTTGCTGGATCCTGGCAGGTTTTTACCTGCTTTTCTGCTGCAAGGCAGAAGAGGGACTGAACTTTCCCACTTACGATGG
GAAAGACCGAGTGATCGACCTGAACGAGAAGAACTACAAGCAGGCTCTGAAGAAGTATGACATGCTCTGCCTGCTCTTCCATGAGCCTG
TGAGTTCTGACAAGATCTCCCAAAAGCAGTTCCAGATGACAGAGATGGTCCTGGAGCTGGCAGCTCAGGTCCTGGAGCCCAGAAGCATC
GGTTTTGGGATGGTGGACTCCAAGAAGGATGCCAAACTTGCAAAAAAATTAGGCTTGCTTGAAGAGGGAAGTCTCTATGTCTTTAAGGA
TGAGCGGTTGATCGAATTTGATGGGGAACTGGCCACAGATGTCTTGGTGGAATTCCTCTTGGATTTGCTAGAAGACCCCGTGGAAGTCA
TAAACAGCAAACTGGAGCTTCAGGCCTTTGACCAGATCGATGATGAAATCAAACTCATCGGCTACTTTAAAGGAGAAGACTCTGAACAT
TTCAAGGCATTTGAAGAAGCTGCTGAACAATTCCAGCCGTACATCAAGTTCTTTGCTACTTTTGACAAAGGGGTTGCCAAGAAGCTAGG
TCTGAAGATGAATGAGGTGGACTTCTATGAACCATTTATGGATGAGCCTGTTCACATCCCTGATAAGCCTTACACAGAAGAGGAGCTGG
TTGAATTTGTGAGAGAGCACAGAAGGGCCACCTTGAGGAAGCTGCGCCCAGAGGACATGTTTGAGACGTGGGAGGATGACATGGAGGGT
ATCCACATCGTAGCCTTCGCTGAAGAAGACGACCCAGACGGTTTTGAGTTCCTGGAAATTCTGAAGCAGGTTGCCAGGGACAACACCGA
TAATCCTGACCTGAGCATTGTCTGGATTGACCCTGATGACTTTCCTCTGCTGATCACTTACTGGGAGAAGACCTTCAAGATCGACTTGT
TCAGACCACAGATCGGGGTGGTGAATGTCACAGACGCTGACAGCATCTGGATGGACATCAGAGATGATGATGATCTGCCCACAGCTGAG
GAGCTGGAAGACTGGATAGAAGATGTGCTTTCTGGGAAGATAAATACTGAAGATGATGATGATGATGATGACGACGATGACGATGATGA
CGATGACGACGATGATGACGATGATGATGACGACGATGATGATGACGACGATGATGATGACGACGATGATGACGATGACTAA

>CASQ2_Ceratopipra_mentalis_protein
MKVTCWILAGFYLLFCCKAEEGLNFPTYDGKDRVIDLNEKNYKQALKKYDMLCLLFHEPVSSDKISQKQFQMTEMVLELAAQVLEPRSI
GFGMVDSKKDAKLAKKLGLLEEGSLYVFKDERLIEFDGELATDVLVEFLLDLLEDPVEVINSKLELQAFDQIDDEIKLIGYFKGEDSEH
FKAFEEAAEQFQPYIKFFATFDKGVAKKLGLKMNEVDFYEPFMDEPVHIPDKPYTEEELVEFVREHRRATLRKLRPEDMFETWEDDMEG
IHIVAFAEEDDPDGFEFLEILKQVARDNTDNPDLSIVWIDPDDFPLLITYWEKTFKIDLFRPQIGVVNVTDADSIWMDIRDDDDLPTAE
ELEDWIEDVLSGKINTEDDDDDDDDDDDDDDDDDDDDDDDDDDDDDDDDDD*

8.4 *Lepidothrix coronata* (blue-crowned manakin)

From: Trinity *de novo* joint assembly of SRR18056691 and SRR1805702. CASQ1 from the complement of TRINITY_DN822_c0_g1_i3. CASQ2 from TRINITY_DN1584_c1_g1_i1.

>CASQ1_Lepidothrix_coronata_transcript_assembly
-CATCCCTGTGTGTCCACCCCCGTGTCCGTGTGTTCCCCCTGTCCCCGTGTCCGTGTGTCCATCCCGGTGTCCGTGTGTCCCCCCGGTG
TCCCCGTGTCTGTGTGTCCATCCCGGTGTCCGTGTGTCCCCCCCGGTGTCCGTGTGTCCCCCCGGTGCCCCCGATGTCCGTCTGTCGGC
GGGCTCTGGCCGTTCTGGCCCTTCTGTCGCTGCTGCCGCTGTGCCCGGTGCTCCCGGGGGTCTCTGCCCGGGGGGGGCTTCACTCGCCC
ACCCCCGGCGGGCGGGGCCGGGGGGTCGCCGCGACCCCGCGGGAGCTGCGGGAGCTGCTGCGGCGCTTCCCGGGGCTGGCGCTGCTCCC
GAGGGACCCCCGGGATGGGGACACCGGGGACAGGGACCCCCGCGACCGGGACACCCGGGACAGGGACCCCCGCGACCGGGACACCCCGG
ACAGGGACCCCCGGGACGGGGACACCCGGGACAAGGACCCCCGAGACGGGGATACCCCGGACAGGGACCCCCGGGAGAAGGAGCCGGTG
ATGGAGGTGCCCGTGAGGGAGGTGAGGTCGGAGCAGGAGCTCCAGGACTTCGAGAACATGGAGGAGGAGCCCAAAGTGATCGGGCACTT
CCAGGGACCCAGCTCGGAGCATTTCGGCTCCTTCGCTGCGGCCGCGCGGAACCTTCACCCCCCCGTGCCCTTCTTCGTCACCTTCGACC
CACAGGTGGCCCAGAGGCTGCGGCTGCGGCTGAACCAGATCGCCCTGTACCCCCCCTTCCAGGACACCCCCCGCACCCTCGGGGGGGGC
CCCGCGGACCCCCCCGCGGACCCCCCCGCGGACCCCCCCGCGATCGCCGCCTTCGTGGAGAACAACCAGCGGTCCCCGCTGAGGAAACT
CAAACCCCAAACCGAGCCCTGGGAGGAGGCGACGGGAGGGACCCTCGTGGTGGCCTTTGCGGAGGGGGATGAGCCGGATGGCTTCGAAT
TCCTGGCGATCCTGAAGGACGTGACCCGGGACACCCCCGGGCTGCGAATCCTCTGGATCGACCCCGACGATTTCCCGCCGCTCGTCCCT
TACTGGGAGGAAATTTTCGACCTCGACCTGTCCCGGCCCCAGCTCGGGGTCATCGACGGGACCGACGTGAGTGGGACCCCCCCGGGACC
CCCCCGAGTGTCCCCTGAGCCCCAAAATGTCCCTTGACCCCCCCAAAATGTCCTCTGATCCCCCAAAGTGCCCCCTG

>CASQ1_Lepidothrix_coronata_CDS
ATGTCCGTCTGTCGGCGGGCTCTGGCCGTTCTGGCCCTTCTGTCGCTGCTGCCGCTGTGCCCGGTGCTCCCGGGGGTCTCTGCCCGGGG
GGGGCTTCACTCGCCCACCCCCGGCGGGCGGGGCCGGGGGGTCGCCGCGACCCCGCGGGAGCTGCGGGAGCTGCTGCGGCGCTTCCCGG
GGCTGGCGCTGCTCCCGAGGGACCCCCGGGATGGGGACACCGGGGACAGGGACCCCCGCGACCGGGACACCCGGGACAGGGACCCCCGC
GACCGGGACACCCCGGACAGGGACCCCCGGGACGGGGACACCCGGGACAAGGACCCCCGAGACGGGGATACCCCGGACAGGGACCCCCG
GGAGAAGGAGCCGGTGATGGAGGTGCCCGTGAGGGAGGTGAGGTCGGAGCAGGAGCTCCAGGACTTCGAGAACATGGAGGAGGAGCCCA
AAGTGATCGGGCACTTCCAGGGACCCAGCTCGGAGCATTTCGGCTCCTTCGCTGCGGCCGCGCGGAACCTTCACCCCCCCGTGCCCTTC
TTCGTCACCTTCGACCCACAGGTGGCCCAGAGGCTGCGGCTGCGGCTGAACCAGATCGCCCTGTACCCCCCCTTCCAGGACACCCCCCG
CACCCTCGGGGGGGGCCCCGCGGACCCCCCCGCGGACCCCCCCGCGGACCCCCCCGCGATCGCCGCCTTCGTGGAGAACAACCAGCGGT

CCCCGCTGAGGAAACTCAAACCCCAAACCGAGCCCTGGGAGGAGGCGACGGGAGGGACCCTCGTGGTGGCCTTTGCGGAGGGGGATGAG
CCGGATGGCTTCGAATTCCTGGCGATCCTGAAGGACGTGACCCGGGACACCCCCGGGCTGCGAATCCTCTGGATCGACCCCGACGATTT
CCCGCCGCTCGTCCCTTACTGGGAGGAAATTTTCGACCTCGACCTGTCCCGGCCCCAGCTCGGGGTCATCGACGGGACCGACGTGAGTG
GGACCCCCCCGGGACCCCCCCGAGTGTCCCCTGAGCCCCAAAATGTCCCTTGA

>CASQ1_Lepidothrix_coronata_protein
MSVCRRALAVLALLSLLPLCPVLPGVSARGGLHSPTPGGRGRGVAATPRELRELLRRFPGLALLPRDPRDGDTGDRDPRDRDTRDRDPR
DRDTPDRDPRDGDTRDKDPRDGDTPDRDPREKEPVMEVPVREVRSEQELQDFENMEEEPKVIGHFQGPSSEHFGSFAAAARNLHPPVPF
FVTFDPQVAQRLRLRLNQIALYPPFQDTPRTLGGGPADPPADPPADPPAIAAFVENNQRSPLRKLKPQTEPWEEATGGTLVVAFAEGDE
PDGFEFLAILKDVTRDTPGLRILWIDPDDFPPLVPYWEEIFDLDLSRPQLGVIDGTDVSGTPPGPPRVSPEPQNVP*

>CASQ2_Lepidothrix_coronata_transcript_assembly
CTCTTGCTCATTCCCTCTCTCTCAGATGATGCAGAGTGCCAGCAAGTCCCTCAATAGTGTCATATCTGTCCTTTTTATTCATTGCAGGG
TTTATTTTTAGCCTGAAACAACTGCTTCCTAAAAATGGAGTTCCTAATGACACGGGAGCTGGACACAGCTATGTAAGGTATCCAGGGCT
TGGCCTCACAGCTTCTCCTGTCTGTCTCTCTCTTTGTTGTGAGTCCAGGACAGCAAAGTTTGCTGCTCCCCACCAGCCTTGCAGTGTTT
GGGCTGATCACCAGGAAGGCAGCAGAAAGAGAAACTCAGTTCAAACTTCACCTGAAGCCTTGGTCTCCCCCTCTTCCCAAATCAAGAAA
GAGAGGGACATTTATCCACCCTTTGCTGCACTCCAAGGGCACCCCTATGAAGGCAACTTGCTGGATCCTGGCAGGTTTTTACCTGCTTT
TCTGCTGCAAGGCAGAAGAGGGACTGAACTTCCCCACTTACGATGGGAAAGACCGAGTGATCGACCTGAACGAGAAGAACTACAAGCAG
GCTCTGAAGAAGTATGACATGCTCTGCCTGCTCTTCCATGAGCCTGTGAGTTCTGACAAGGTCTCCCAAAAGCAGTTCCAGATGACAGA
GATGGTCCTGGAGCTGGCAGCTCAGGTCCTGGAGCCCAGAAGCATTGGTTTTGGGATGGTGGACTCCAAGAAGGATGCCAAACTTGCCA
AAAAATTAGGCTTGCTTGAAGAGGGAAGTCTCTATGTCTTTAAGGATGAGCGGTTGATTGAATTTGATGGGGAACTGGCCACAGATGTC
TTGGTGGAATTCCTCTTGGATTTGCTAGAAGACCCCGTGGAGGTCATAAACAGCAAGCTGGAGCTTCAGGCCTTTGACCAGATCGATGA
TGAAATCAAACTCATCGGCTACTTTAAAGGAGAAGACTCTGAACATTTCAAGGCATTTGAAGAAGCTGCTGAACAATTCCAGCCGTACA
TCAAGTTCTTTGCTACTTTTGACAAAGGGGTTGCCAAGAAGCTAGGTCTGAAGATGAATGAGGTGGACTTCTATGAACCATTTATGGAT
GAGCCTGTTCACATCCCTGATAAGCCTTACACAGAAGAGGAGCTGGTTGAATTTGTGAGAGAGCACAGAAGGGCCACCTTGAGGAAGCT
GCGCCCAGAGGACATGTTTGAGACGTGGGAGGATGACATGGACGGTATCCACATCGTAGCCTTCGCTGAAGAAGATGACCCAGATGGTT
TTGAGTTCCTGGAAATTCTGAAGCAGGTTGCCAGGGACAACACTGATAATCCTGACCTGAGCATTGTCTGGATTGACCCTGATGACTTT
CCTCTGCTGATCACTTACTGGGAGAAGACCTTCAAGATCGACTTGTTCAGACCACAGATCGGGGTGGTGAATGTCACAGACGCTGACAG
CATCTGGATGGACATCAGAGATGATGATGATCTGCCCACAGCTGAGGAGCTGGAAGACTGGATAGAAGATGTGCTTTCTGGGAAGATAA
ATACTGAAGATGATGATGATGACGATGATGACGACGATGATGATGACGATGACGACGATGACGACGATGATGATGACGACGACGATGAC
GATGACTAACTGTGACTCTGTGCAGTTTGACTTGTGGGGCCCGAGAGCCCTCCCCCTGCGGCAGGTCCTTCCATTGGAAGGCCTGTGTT
TGAGCAAGCACTGCTGCTCCTATTTGCCCCCTGCCCTGCTCCCCCTGCCCCTGCCCTTGCCGGGACCCCCTGGCCTGATTCTCAGTGGT
GCTGAGCCACCGGGACTGCCCCTGCGGTGGGCAGTGCCTGCAGGCACCCAGCACCGCTGCAAATCATGCCTCCTTAGTAAGGACAATAG
GAATCTGCAGGGAATCTGCCCCCAGTGTCCCAGGGCAGGAGAAAAGTGCCTGCCTGCTTGCTGCCAGAGGGACGCAGGGAGCCAGTTTG
TGTTTTCCATGTTCATCAGCCCAGGACTTAACATCCAAAGACCAAATCTCCCTTCAAGACATTGAGAAGGACACTGTAAAGTTTAGTCC
TTTTATTAAACACAATGCCCTTTGCAGATACTCTGCCAAGACCACTCAATCCTGCAAGTCCTGCTGCGGGAGTCCCTAGCTAACGCTAG
GAGCAGCAAAATGCTTTGGGAGGGGAGGAAGGTTTTAGGGATGCTATTAAAGTTTTCAGTGATTCCACATAACAACAGGATATTCTTGT
CTGGCAGTGTGATGCAAAACCAGTCGCCATGCCCTCTGTCTGAACCTCTTTTCTTGAAAGCTCGGGCCATGGCCGGGGAAGTGCAGATG
GTCTGATAGGAGGCATAAAAGCTCACAAGTTCCTGAACTGCTGGAAAGGCTGATCCAGGTCTGAGTGCAAACTCTTGAGTTTGAAGACC
ATGGCACTGAGTCAAGCCAATGGAAACATCCGTAGGGGCCCACCCTGAAGCTCTCCTATTCCCCATGACCTTCTGGCTTTAGCTGACTC
TTCACTCAAAGCCCATGTAGCATCTATTCCCATGACCAAGCTGTTGGGAGGGAACACACCCATCCAACTACATTTGATATTGGATGAGG
TACGTCGGGCAATGAGCGGCGAATGGATTCAAGTTGTTTGGCTTTAGGTATTTAGTTGCACAGTTACTCCCTGCTGAGAGACTCGGATG
TCCCATAGTAGCCTGCGAGAAGCATCTTTTGACTGCAGGAGCTCAGGGTGCCAGTTTGCAGTCAAGGGGGTCGCTCACAAAGCAAAGGG
TCTGCAACACAGTCCTGGTGAGTGCAGCACCATCCACATGCAGGTCACTGTGGCAGCATTAAATCTCCCTGCTTTCCCATGCCAGCAGA
TTTCAATTGTCTTAGCTTATTAGGTTTGACAATAGTACAGTGAACAGAGAGTTCGGAGATAGTTTTATCTCTATATACACATTTATAAG
GATGTTCTCTGGTTGTTCAGGCTTGTTAGCATAGGTAGGGTATGCATCTCCTTTCTTGCTACAGATGGAAAACATTGTCCTGACTGTTC
TTTTACTGGCTTACGTCAGAGGTAAGTGATATTAGAGGAAATAACAGTGTGAGGGACAGGAGAATCAGAGCTCATTACACAAGTGTGAA
TCAGCCTGATGAGAAACAAGGTTCACACACTATATATTTCCTTTATCAGATGAACCACAAAGCAAGATTTTATAGATTTTTTTTTTTGC
TGCCTGTTGATCAGTCTCTTTGACTCTCCTCTACATTAGCTGAAACAGAAATTTGAAACTACTGGTATAAAAAAGCTAAAGTATTGATA
TATTCCTATGTATGCCATATCCCTTGGTAGAAAATGCCAGTATGAACAACACATGGCTGGATATCTATATATATCTATGCATATGAATG
AATACTGTGCTAATATGGCATATGACTGTAGGCCAAGTTAGCTCCTCACTGAATATAAAACATTCAACATGTGCTTTTAAGACACAGCT
ATTAGAAGAATAGACAAGGCAGCAAAGCCCCCAGGCCAACTTTTCATGCTTTCTTGCTTCCTTATGATACATAAGCACTGCCTGGAAGA
CGAACCAACTTTCTGGGGGAAACCATTCACCTGGTTGCTCTGGGGTGACGAACAACTTTCAAAAGCCTTGAAATGCAGAGTGCTCTCAG
CATGGAGGTCCCCTTCTCCTTTTCTCTCACATTTGCCTCTCTCCTGGCCAACAGGGTGCCCAGCACTCTACTCTTTCCTTTGAGCTCTT
TCCTCAAGAGGTTTGCTGGAGGACCCTGGGTCAGAGAGAGAGGGAGCAGGCAGGACTCAAGGTCTCATTCATGCACATTGGTAAAGAGC
AAGAAGAGGCAAAACTGGCCATCTAGTGGTCTCCCAGTCTGCAATTAAGGGACAACTACTCACCTGAGAAAAATACATGCTTATTTTTA
CCTTGGGACTACTCCTAGGAGTTAAACATGCCCAGTTTCACCCTTCATAAGGCAACAGCCTGCACAGGCCCTGCAGCTCCATATCAGTC
ACTCTAAACACGAAGAATAAACGAAGAGCTGCTCTACTGCCTTTCCTAACTAGCAGAGTCTCCAGACATCTCAACTACCTGGATACTTT
CCATGTAGCTCCCCTCCTGCTGTGGATGCCTGTCTCTTCTAATGTGCCCTTCTCCAGACGCACTGAGAGGCACAGCTTACCCAGGCAGA
GCAGGCAGACCCTGCAAATAATGCTATGGACACGTTCAAGTGCACAGGTGTTTCACAGGCATTCATATATTTCAAAGCAACAGAGCTCT
GGGGGGAAAATAGAATAAATAATGTAGAAGTGTAGAGGTTGAGCTCATATGGGGGCTGGTGGACTGGTTTATGTGTTTTATGGACAGTT

TTCCTTGAGTTATGAAAGGGCTAATGCTACAGCTGCCACTCCTTCGTGGTGCCAGTGAAGTCCCTGACGGGGCTGCCCTTTCTTTCTTG
CCTAATCCCCCCAGTACAATTTTCATCATTCTCTTTGCCCACAAACAGGAACTGTTTATTATCAGCTTTTAGGACAAATACATGAAAAG
TTAAGAAATGATGCAGCAGAGGAAGGATTCCAGCAATGAGGATGGGTGGGCAGCAAACTCATTTACCTGTAACACTTCATCTGCCCCAA
GATCCTAGTGACCAAACTGCTGAATGAGGCAATACCTCTTGTGAAGGAGATGGCAGCTTAAACTTGAGTGACTTCCTTGCAGGGATCAG
TAAAACAGTGGTATGGGGAGGACACACAGGACTCCAGGCACAGGTATTCCTGGGCTCCTCTGTCAGAACTAGGTACAACTGGCATTTGA
AAGGCAGTGGTGGCCCTGAGGAACTAAGTCAGCCTAACTCCCCCCACTACACAGTAACAAGCAAAAGGTTGATTTGGGTGAGGAACCTG
CAAAAACCCCTTAATTAAAAAAAAAAAAAAGAAAGAAATTGTGTACATGAGCAGCACTGAGTGTTTCCGCAAGCACATCTGCTAAGACTG
TCCCTCTCTTCAAGAATAGTTCTTTTTCTCCAGCATGTTAATTAGACATATTAAATGAGTCTTAACACACATAAAGAAAAGTAATTGGA
TTTTTCTCTCATCCAACTGTGAGTACTTCAAGCTGATCAGTTACTTCATAGAATCATTCAATCATTTTTGTTGGAAAAGACCCCTAAGA
TCATTGAGTCCAACCATTAATCCACCACTGCCAAGTAAGTACCCAAAATAACCCAAAAGATAGTCTTACCAGCCCCCAGTGACAGTAAG
GACCCCACCCCCCCACAAGTGATACATGGCAAGAACACCAAGTCATACACCACCACCTCCTCCCAGTCACCAGACACGGGGCCAGAGGC
CACAGCCAAGGGATTTGTTTAGTGTACCACAGGCCACCCCGTGCTGCTCATGGGCAACAACCAAGGCAGATGGAAAGCCACCGGGAGCC
ACATCCTGCAGCTATCATACTGTAGCATGGAGGAGAAAGTTTCTGCAGTGGGCCCAAGGTCACTGACGTAGAGGCTCTGTGAACCCAAA
ACTACTCTGAGCTTATGTCAACCATCTGGGAAAGAGCTCCAGAAGGAGGGGACAACAGAGGAACTTTGGAAACAGCAGATTACTCCACG
GTTTCAGGGTCAGCGTTTTCTGTGATGTTTCTGAGGAGTTTTGAGGCTCCTGTGTCTCCCCTGGGAGAGGAATGGACCCGTACTGAAAC
CAGAGATCTTGGCTCCTTCCCATCCCAAAACAAAGCTGCTCAGCCAGACACCAACATGAACGTGGAGCTGAGGACACCAGCTGCTTCTT
CATGTCTGTCTGCACATCATACCTAAAGAAATGATCGATTCATTACAGAAACCAAACAAAAAACATCATCTTTTATTCCCAGGAAAATA
ATTAATTGGACAGGAACATATCACTTCCCAGCTGCCACTGACTTGCAGTCCTGTGAAAAGAGGCCAAGATTGCATCTGGCTTCATCTCT
GCAGCAAAGCTTTGAGCTTTGGCTTTGAGGAAGTCTCACACAGAGCCAGTTGCACACCAATGCCTGGACCTGGGGGTCTGAAAAGGAGG
AGCAGGTAAACAGGCAACAAGCACCAAGGCCCTCCGAGACAAAGCACCCTGGCTCCTGGCTGCTTTTACACCCCAGGTATGTCTATGCC
ATGCAGTGCAAGACCATCTGCTAGAAACTGGAAGCAGCAGAGAGCAGCAGGCAGGATAACTGACCTCAGTAATGAGGCTGCACCAGCAG
GGCTGTGTCCTACCTGAGACCCAAGACCGTGTGTGTCAACCCACTGCACTGCAGCATCCCCTGCTCCCTTTTTGGCACTCCCTTGTACC
ACTGGCATCTAGCAAAGCAGCGCTTCGATGGTCAGTACCCCTGGGGGACAGCCATGGGATGGCTCTCATACCTGTTTGGCAGAAGATGC
TGTTTAATGATGACACAGATCATGAGGCAGGTCCAAGTGCAGAGCCAGCCCTCTCGGCCTTGCTCCCTGAACACTCTCTGCCAGGTTTA
AAAGGTGCTAAACTATTTAAAGGGGAAAAAAAAAAAGCTACAGAACTTTCTTGCAGCTTGAAGATCCGTTTCTGGAATCAGATTACAAA
CAGAGATGGAAGCAAAGATCCTTCCGGTGCCTCACTCCCCCCTCGTGCTTTCTCTTGCCAAGCCCCTGTCAAAGGGCATTGTCCCTAAT
GCTATGGAAAATAAGAAGAAAACAAGCCTTTTCACCTTCTTTTCATCCCTCTTGAATTTCTACTCTTCTGATCCTCACTGGCTCCCCTA
CTTTATTTTCTCTCTCCATTTCTATTTAACCTGTCTTCCTCTTTTTCTTAACCCACCCCCACAGCATATGGTACTGAGGGTCACTTAAG
GACACAGCCCTTCCCTGCCCCTACTGCTTGGGCTCATCCCAGTCCAAGGAGACAGCAGCCTCCCCCACACCACTGCCAGGAACTGGCAC
GGACAACAACTGGGAAGGACAACATTTCCTTTCTGAGGGGAAAGGCAGTTGAACAAGGCTGTCACTGCCTTTCATTTTGAGGGGGACAG
GTGGGACATGCTCAAGGAGAAGCCATTTCACCAGCCTGGGCACTGAGGAAAAACCAGAATTTTTTTTTGGTGGGCATCTTTTCCCATTG
TCCCAAAATCCAGATGAGGAACCATCACCCTGCAATCAAGCCCTGACTGTTGCCACCATGGAAAGAGACCAGTAACCCCCAGTGACACC
AGCGATCACACCGCTTCTGCTCACCATCTGAGCAGAAGACAAGTGTCATGGCTTCACATACAAGAAATAAGCCTTCAGGGCTAAATGAG
CTGAGTTTGCTGAGGGGGTATCCCATACTTTAACAGCAGCCAGAAAGCCAACTCCCTGCCGGAGACACAACCAGTGGTTGTCCTGACCG
AGTTCTCCTTCAGTCGTGGGTGGTAAATTATTTAAATAGCCATGGGCAACCTCATCTTCTGCTTTTGGATTTGGAGGAAGCCCAGCTTG
AATCAGGTCCTTCCCTCAGTAACACAGGTAAAAATAAAGTCATGTTTGACCAGAGAGCAGCCCTGAATTTTGCCTGGTGTTAGATTTTT
GCTTTAAAATTCAGTCCAACACGTCTCAGCCCTGCTCCTGCTAGTGACAAAGAGAGAGGTTATTGAACAACAAAAGTAACTAAATGTTT
GAGGAACATTTCAGTCCCCACAAAATGAAATT

>CASQ2_Lepidothrix_coronata_CDS
ATGAAGGCAACTTGCTGGATCCTGGCAGGTTTTTACCTGCTTTTCTGCTGCAAGGCAGAAGAGGGACTGAACTTCCCCACTTACGATGG
GAAAGACCGAGTGATCGACCTGAACGAGAAGAACTACAAGCAGGCTCTGAAGAAGTATGACATGCTCTGCCTGCTCTTCCATGAGCCTG
TGAGTTCTGACAAGGTCTCCCAAAAGCAGTTCCAGATGACAGAGATGGTCCTGGAGCTGGCAGCTCAGGTCCTGGAGCCCAGAAGCATT
GGTTTTGGGATGGTGGACTCCAAGAAGGATGCCAAACTTGCCAAAAAATTAGGCTTGCTTGAAGAGGGAAGTCTCTATGTCTTTAAGGA
TGAGCGGTTGATTGAATTTGATGGGGAACTGGCCACAGATGTCTTGGTGGAATTCCTCTTGGATTTGCTAGAAGACCCCGTGGAGGTCA
TAAACAGCAAGCTGGAGCTTCAGGCCTTTGACCAGATCGATGATGAAATCAAACTCATCGGCTACTTTAAAGGAGAAGACTCTGAACAT
TTCAAGGCATTTGAAGAAGCTGCTGAACAATTCCAGCCGTACATCAAGTTCTTTGCTACTTTTGACAAAGGGGTTGCCAAGAAGCTAGG
TCTGAAGATGAATGAGGTGGACTTCTATGAACCATTTATGGATGAGCCTGTTCACATCCCTGATAAGCCTTACACAGAAGAGGAGCTGG
TTGAATTTGTGAGAGAGCACAGAAGGGCCACCTTGAGGAAGCTGCGCCCAGAGGACATGTTTGAGACGTGGGAGGATGACATGGACGGT
ATCCACATCGTAGCCTTCGCTGAAGAAGATGACCCAGATGGTTTTGAGTTCCTGGAAATTCTGAAGCAGGTTGCCAGGGACAACACTGA
TAATCCTGACCTGAGCATTGTCTGGATTGACCCTGATGACTTTCCTCTGCTGATCACTTACTGGGAGAAGACCTTCAAGATCGACTTGT
TCAGACCACAGATCGGGGTGGTGAATGTCACAGACGCTGACAGCATCTGGATGGACATCAGAGATGATGATGATCTGCCCACAGCTGAG
GAGCTGGAAGACTGGATAGAAGATGTGCTTTCTGGGAAGATAAATACTGAAGATGATGATGATGACGATGATGACGACGATGATGATGA
CGATGACGACGATGACGACGATGATGATGACGACGACGATGACGATGACTAA

>CASQ2_Lepidothrix_coronata_protein
MKATCWILAGFYLLFCCKAEEGLNFPTYDGKDRVIDLNEKNYKQALKKYDMLCLLFHEPVSSDKVSQKQFQMTEMVLELAAQVLEPRSI
GFGMVDSKKDAKLAKKLGLLEEGSLYVFKDERLIEFDGELATDVLVEFLLDLLEDPVEVINSKLELQAFDQIDDEIKLIGYFKGEDSEH
FKAFEEAAEQFQPYIKFFATFDKGVAKKLGLKMNEVDFYEPFMDEPVHIPDKPYTEEELVEFVREHRRATLRKLRPEDMFETWEDDMDG
IHIVAFAEEDDPDGFEFLEILKQVARDNTDNPDLSIVWIDPDDFPLLITYWEKTFKIDLFRPQIGVVNVTDADSIWMDIRDDDDLPTAE
ELEDWIEDVLSGKINTEDDDDDDDDDDDDDDDDDDDDDDDDDDDDD*

8.5 *Manacus vitellinus* (golden-collared manakin)

From: Trinity *de novo* joint assembly of SRR18056686 and SRR18056687. CASQ1 from the complement of TRINITY_DN587_c0_g1_i27. CASQ2 from TRINITY_DN2017_c0_g1_i2.

```
>CASQ1_Manacus_vitellinus_transcript_assembly
CCCCGTTCCCCCCGATGTCCGTCTGTCCGCGGGCTGTGGCCGTTCTGGCCGTTCTGTCGCTGCTGTCGCTGTGCCCGGGGGTCCCGGGG
GTCTCTGCCCGGGGGGGGCTGCACTTGCCCCCCCGGGGGCGGGGCCGGGGGGTCGCCGCGACCCCGCGGGAGCTGCGGGAGCTGCGGGA
GCTGCTGCGGCGCTTCCCGGGGCTGGCGTTGCTCCCGAGGGACCCCCGAGAGGGGGACACCCCGGACAGGGACCCCCGCGACGGGAACA
CCCCGGACAGGGACCCCCGAGAATGGAACCCCCAGGAGAAGGAGCCGGTGATGGAGGTGCCGCAGGAGCCCGTGAGGGAGGTGACATCG
GTGCTGGTGAGGGAGGTGACATCGGAGCAGGAGCTCCAGGACTTCGAGAACCTGGAGGAGGAGCCCAAAGTGATCGGGCACTTCCAGGG
ACCCGGCTCGGAGCATTTCGGCTCCTTCGCGGCGGCCGCGCGGACCCTTCACCCCCCCGTGCCCTTCTTCGTCACCTTCGACGCACAGG
TGGCCCGGAGGCTGCGGCTGCGGTTGAACCAGATTGTCCTGTACCCCCCCTTCCAGGACACCCCCCACACCCTCGGGGGGGGCCCCGCG
GACCCCCCCCCGGACCCCCCAACCATCGTGACCTTCGTGGAGAGCAACCAGCGGTCCCCGCTGAGGAAACTCAAACCCCAAACCCCGCA
CGAGCCCTGGGAGGAGGCGACGGGAGGGACCCTCGTGGTGGCCTTTGCGGAGGGGGATGAGCCGGATGGCTTCGAATTCCTGGCGATCC
TGAAGGACGTGGCCCGGGACAACCCCGGGCTCAGAATCCTCTGGATCGACCCCGACGATTTCCCGCCGCTCGTCCCTTACTGGGAGGAA
ATTTTCGACCTCGACCTGTCCCGGCCCCAGCTCGGGGTCATCAACGGGACTGACGGTGTCGGGGTGTGGCTGGAGATGCAGCAGCAGCA
GCAGCAGGAGGAGGAGGAGAAGGAG
```

```
>CASQ1_Manacus_vitellinus_CDS_partial
ATGTCCGTCTGTCCGCGGGCTGTGGCCGTTCTGGCCGTTCTGTCGCTGCTGTCGCTGTGCCCGGGGGTCCCGGGGGTCTCTGCCCGGGG
GGGGCTGCACTTGCCCCCCCGGGGGCGGGGCCGGGGGGTCGCCGCGACCCCGCGGGAGCTGCGGGAGCTGCGGGAGCTGCTGCGGCGCT
TCCCGGGGCTGGCGTTGCTCCCGAGGGACCCCCGAGAGGGGGACACCCCGGACAGGGACCCCCGCGACGGGAACACCCCGGACAGGGAC
CCCCGAGAATGGAACCCCCAGGAGAAGGAGCCGGTGATGGAGGTGCCGCAGGAGCCCGTGAGGGAGGTGACATCGGTGCTGGTGAGGGA
GGTGACATCGGAGCAGGAGCTCCAGGACTTCGAGAACCTGGAGGAGGAGCCCAAAGTGATCGGGCACTTCCAGGGACCCGGCTCGGAGC
ATTTCGGCTCCTTCGCGGCGGCCGCGCGGACCCTTCACCCCCCCGTGCCCTTCTTCGTCACCTTCGACGCACAGGTGGCCCGGAGGCTG
CGGCTGCGGTTGAACCAGATTGTCCTGTACCCCCCCTTCCAGGACACCCCCCACACCCTCGGGGGGGGCCCCGCGGACCCCCCCCCGGA
CCCCCCAACCATCGTGACCTTCGTGGAGAGCAACCAGCGGTCCCCGCTGAGGAAACTCAAACCCCAAACCCCGCACGAGCCCTGGGAGG
AGGCGACGGGAGGGACCCTCGTGGTGGCCTTTGCGGAGGGGGATGAGCCGGATGGCTTCGAATTCCTGGCGATCCTGAAGGACGTGGCC
CGGGACAACCCCGGGCTCAGAATCCTCTGGATCGACCCCGACGATTTCCCGCCGCTCGTCCCTTACTGGGAGGAAATTTTCGACCTCGA
CCTGTCCCGGCCCCAGCTCGGGGTCATCAACGGGACTGACGGTGTCGGGGTGTGGCTGGAGATGCAGCAGCAGCAGCAGGAGGAGG
AGGAGAAGGAG
```

```
>CASQ1_Manacus_vitellinus_protein
MSVCPRAVAVLAVLSLLSLCPGVPGVSARGGLHLPPRGRGRGVAATPRELRELRELLRRFPGLALLPRDPREGDTPDRDPRDGNTPDRD
PREWNPQEKEPVMEVPQEPVREVTSVLVREVTSEQELQDFENLEEEPKVIGHFQGPGSEHFGSFAAAARTLHPPVPFFVTFDAQVARRL
RLRLNQIVLYPPFQDTPHTLGGGPADPPPDPPTIVTFVESNQRSPLRKLKPQTPHEPWEEATGGTLVVAFAEGDEPDGFEFLAILKDVA
RDNPGLRILWIDPDDFPPLVPYWEEIFDLDLSRPQLGVINGTDGVGVWLEMQQQQQEEEEKE
```

```
>CASQ2_Manacus_vitellinus_transcript_assembly
CCCAGGACTGTCATCTTCCACTCCCTCCCTCTTGCTCATTCCCTCTCTCTCAGATGATGCAGAGTGCCAGCAAGTCCCTCAATAGTGTC
ATATCTGTCCTTTTTATTCATTGCAGGGTTTATTTTTAGCCTGAAACAACTGCTTCCTAAAAATGGAGTTCCTAATGACACGGGAGCTG
GACACAGCTATGTAAGGTATCCAGGGCTTGGCCTCACAGCTTCTCCTGTCTGTCTCTCTCTTTGTTGTGAGTCCAGGACAGCAAAGTTT
GCTGCTCCCCACCAGCCTTGCAGCGTTTGGGCTGATCACCAGGAAGGCAGCAGAAAGAGAAACTCAGTTCAAACTTCACCTGAAGCCTT
GGTCTCCCCCTCTTCCCAAATCAAGAAAGAGAGGGATATTTACCCACCCTTTGCTGCACTCCAAGGGCACCCTTATGAAGGCAACTTGC
TGGATCCTGGCAGGTTTTTACCTGCTTTTCTGCTGCAAGGCAGAAGAGGGACTGAACTTCCCCACTTACGATGGGAAAGACAGAGTGAT
CGACCTGAACGAGAAGAACTACAAGCAGGCTCTGAAGAAGTATGACATGCTCTGCCTGCTCTTCCATGAGCCTGTGAGTTCTGACAAGG
TCTCCCAAAAGCAATTCCAGATGATAGAGATGGTCCTGGAGCTGGCAGCTCAGGTCCTGGAGCCCAGAAGCATCGGTTTTGGGATGGTG
GACTCCAAGAAGGATGCCAAACTTGCCAAAAAATTAGGCTTGCTTGAAGAGGGAAGTCTCTATGTCTTTAAGGATGAGCGGTTGATCGA
ATTTGATGGGGAACTGGCCACAGATGTCTTGGTGGAATTCCTCTTGGATTTGCTAGAAGACCCCGTGGAGGTCATAAACAGCAAGCTGG
AGCTTCAGGCCTTTGACCAGATCGATGATGAAATCAAACTCATCGGCTACTTTAAAGGAGAAGACTCTGAACATTTCAAGGCATTTGAA
GAAGCTGCTGAACAATTCCAGCCGTACATCAAGTTCTTTGCTACTTTTGACAAAGGGGTTGCCAAGAAGCTAGGTCTGAAGATGAATGA
GGTGGACTTCTATGAACCATTTATGGATGAGCCTGTTCACATCCCTGATAAGCCTTACACAGAAGAGGAGCTGGTTGAATTTGTGAGAG
AGCACAGAAGGGCCACCTTGAGGAAGCTGCGCCCAGAGGACATGTTTGAGACGTGGGAGGATGACATGGAGGGTATCCACATCGTAGCC
TTCGCTGAAGAAGACGACCCAGATGGTTTTGAGTTCCTGGAAGTTCTGAAGCAGGTTGCCAGGGACAACACCGATAATCCTGACCTGAG
CATTGTCTGGATTGACCCTGATGACTTTCCTCTGCTGATCACTTACTGGGAGAAGACCTTCAAGATCGACTTGTTCAGACCACAGATCG
GGGTGGTGAATGTCACAGACGCTGACAGCATCTGGATGGACATCAGAGATGATGATGATCTGCCCACAGCTGAGGAGCTGGAAGACTGG
ATAGAAGATGTGCTTTCTGGGAAGATAAATACTGAAGATGATGATGATGACGACGATGACGACGACGATGATGACGATGACGACGATGA
TGACGATGATGATGACGACGATGATGAAGACGACGATGATGACGATGACTAACTGTGACTCTGTGCAGTTTGACTTGTGGGGCCCGAGA
GCCCTCCCCCTGCGGCAGGTCCTTCCATTGGAAGGCCTGTGTTTGAGCAAGCACCGCTGCTCCTATTTGCCCCCTGCCCTGCTCCCCGT
GCCCCTGCCCTTGCCGGGACCCCCTGGCCTGATTCTCAGTGGTGCTGAGCCACCGGGACTGCCCCTGCGGTGGGCAGTGCCTGCAGGCA
CCCAGCACCGCTGCAAATCATGCCTCCTTAGTAAGGACAATAGGAATCTGCAGGGAATCTGCCCCGAGTGTCCCAGGGCAGGAGAAAAG
```

TGCCTGCCTGCTTGCTGCCAGAGGGACGCAGGGAGCCAGTTTGTGTTTTCCATGTTCATCAGCCCAGCACTTAACATCCAAAGACCAAA
TCTCCCTTCAAGACATTGAGAAGGCCTCTGTAAAGTTTAGTCCTTTTATTAAACACAATGCCCTTTGCAGATACTCTGCCATGACCACT
CAATCCTGCAAGTCCTGCTGCGGGAGTCCCTAGCTAACACTAGGAGCAGCAAAATGCTTTGGGAGGGGGAGGAAGGTTTTAGGGATGCTA
TTAAAGTTTTTGGTGATTCCACATAACAACAGGATATTCTTGTCTGGCAGTGTGATGCAAAACCAGTCACCATGCCCTCTGTCTGAACC
TCTTTTCTTGAAAGCTCGGGCCATGGCCGGGGAAGTGCAGATGGTCTGATAGGAGGCATAAAAGCTTGCAAGTTCCTGAACTGCTGGAA
AGGCTGATCCAGGTCTGAGTCCAAACTCTTGAGTTTGAAGACCATGGCACTGAGTCAAGCCAATGGAAACATCCGTAGGGGCCCACCCT
GAAGCTCTCCTATTCCCCATGACCTTCTGGCTTTAGCTGACTCTTCACTCAAAGCCCACGTAGCATCTATTCCCATGACCAAGATGTTG
GGAGGGAACACACCCATCCAACTACATTTGATATTGGATGAGGTATGTCGGGCAATGAGCGGCGAATGGATTCAAGTTGTTTGGCTTTA
AGTATTTAGTTGCACAGTTACTCCCTGCTTAGAGACTTGGATGTCCCATAGTAGCCTGCGAGAAGCATCTTTTGACTGCAGGAGCTCAG
GGTGCCAGTCAAGGGGGTTGCTCACAAAGCAAAGGGTCTGCAACACAGTCCTGGTGAGTGCAGCACCATCCACATGCAGGTCACTGTGG
CAGCAGCATTAAATCTCCCTGCTTTCCCATGCCAGCAGATTTCAATTGTCTTAGCTTATTAGGTTTGAAAATAGTACAATGAACAGAGA
GTTCGGAGATAGTTTTATCTCTATATACACATTTATAAGGATGTTCTCTGGTTGTTCAGGCTTATTAGCATAGGTAGGGTATGCATCTC
CTTTCTTGCTACAGATGGAAAACATTGTCCTGACTGTTCTTTTACTGGCTTACGTCAGAGGTAAGTGATATTAGAGGAAATAACAGTGT
GAGGGACAGGAGAATCAGAGCTCATTACACAAGTGTGAATCAGCCTGATGAGAAACAAGGTTCACACACTATATATTTCCTTTATCAGA
TGAACCACAAAGCAAGATTTTATAGATTTTTTTTTTGCTGCCTGTTGATCAGTCTCTTTGACTCTCCTCTACATTAGCTGAAACAGAAA
TTTGAAACTACTGGTATAAAAAAGCTAAAGTATTGATATATTCCTATGTATGCCATATCCCTTGGTAGAAAATGCCAGTATGAACAACA
CATGGCTGGATATCTATATATCTCTATATCTCTATGCATATGAATGAATACTGTGCTAATATGGCATATGACTGTAGGCCAAGTTAGCT
CCTCATTGAATATAAAACATTCAACATGTGCTTTTAAGACACAGCTATTAGAAGAATAGACAAGGCAGCAAAGCCCCCAGGCCAACTTT
TCATGCTTTCTTGCTTCCTTATGATACATAAGCACAGCCTGGAAGAAGAACCAACTTTTTGGGGGAAACCATTCACCTGGTTGCTCTGG
GGTGACGAACAACTTTCAAAAGCCTTGAAATGCAGAGTGCTCTCAGCATGGAGGTCCCCTTCTCCTTTTCTCTCACATTTGCCTCTCTC
CTGGCCAACAGGGTGCCCAGCACTCTCCTTCTACTCTTTCCTTTGAGCTCTTTCCTCAAGAGGTTTGCTGGAGGACCCTGGCTCAGAGA
GAGAGGGAGCAGGCAGGACTCAAGGTCTCATTCATGCACACTGGTAAAGAGCAAGAAGAGGCAAAACTGGCCATCTAGTGGTCTCCCAG
TCTGCAATTAAGGGACAACTACTCACCTGAGAAAAATACATGCTTATTTTTACCTTGGGGCTATTCCTAGGAGTTTAACATGCCCAGTT
TCACCCTTCATAAGGCAACAGCCTGCACAGGCCCTGCAGCTCCATATCAGTCACTCTAAACACGAAGAATAAACAAAGAGCTGCTCTAC
TGCCTTTCCTAACTAGCACAGTCTCCGGACATCTCAACTACCTGGATACTTTCCATGTAGCTCCCCTCCTGCTGTGGATGCCTGTCTAT
TCTAATGTGCCCTTCTCCAGACGCACTGAGAGGCACAACTTACCCAGGCAGAGCAGGCAGACCCTGCAAATAATGCTACAGACACGTTC
AAGTGCACAGGTGTTTCACAGGCATTCATATATTTCAAAGCAACAGAGCTCTGGGGGGAAAATAGAATAAATAATGTAGAAGTGTAGAG
GTTGAGCTCATATGGGGGCTGGTGGACTGGTGCCAGTGTTTTATGGACAGTTTTCCTTGAGTTATGAAAGGGCTAATGCTACAGCTGCC
ACTCCTTCGTGGTGCCAGTGAAGTCCTTGATGGGGCTGCCCTTTCTTTCTTGCCTAATCCCCCCAGTACAATTTTCATCATTCTCTTTG
CCCACAAACAGGAACTGTTTATTATCAGCTTTTAGGACAAACACATGAAAAGTTAAGAAATGTTGCAGCAGAGGAAGGATTCCAGCAAT
GAGGATGGGTGGGTAGCAAACCCATTTACCTGTAACACTTCATCTGCCCCAAGATCCTAGTGACCAAACTGCTGAATGAGGCAATACCT
CTTGTGAAGGAGATGGCAGCTTAAACTTGAGTGACTTCCTTGCAGGGATCAGTAAAACAGTGGTTTGAGGAGAACACACAGGACTCCAG
GCACAGGTATTCCTGGGCTCCTCTGTCAGAACTAGGTACAACTGGCATTTGAAAGGCAGTGGTGGCCCTGAGGAACTAAGTCAACCTAA
CTCCCCCCACTACATACCGACAAGCAAAAGGTTGATTTGGGTGAGGAACCTGCAAAAACCCCTTAATTAAAAAAAAAAAAAAAAAGAAAT
TGTGTACATGAGCAGCACTGTTTCCGCAAGCACATCTGTTAAGACTGTCCCTCTCTTCAAGAATAGTTCTTTTTCTCCAGCATGTTAAT
TAGACATATTAAATGAGTCTTAACACGCATAAAGACAAGTAATTGGATTTTTCCCTCATGTAACTGTGAGTACTTCAAGCTGATCAGTT
ACTTCATAGAATCATTCAATCATTTTTGTTGGAAAAGACCCCTAAGATCATTGAGTCCAACCATTAATCCACCACTGCCAAGTAAGTAC
CCAAAATAACTCAAAAGATAGTCTTACCAGCCCCCAATGACACTAAGGACCCCACCCCCCCACAAGTGATACATGGCAAGAACACCAAG
CCCTACACCACCACCACCTCCTCTTAGTCACCAGACACGGGGCCAGAGGCCACAGCCAAGGGATTTGTTTGGTGTGCTGTACCACAGGC
CACCCCGTGCTGCTCATGGGCAACAACCAAGGCAGATGGAAAGCCACCAGGAGCCACATCCTGCAGCTATCATACTGCAGCATGGAGGA
GAAAGTTTCTGCAGTGGGCCCAAGGTCACTGACGTAGAGGCTCTGTGAACCCAAAACTGCTCTGAGCTTATGTCAACCATTTGGGAAAC
AGCTCCAGAAGGAGGGGACAACAGAGGAACTTTGGAAACAGCAGATTACTCCACGGTTTCAGGGTCAGCAGTTTCTGTGATGTTTCTGA
GGAGTTTTGAGGCTCCCGTGTCTCCCCTGGGAGAGGAATGGACCCGTAGTGAAACCAGAGATTTTGGCTGCTTCCCATCCCAAAACAAA
GCTGCTCAGCCAGACGCCAACATGAACGTGGAGCTGAGGACACCAGCTGCTTCTTCATGTCTGTCTGCACATCATACCTAAAGAAATGA
TTGATTCATTACAGAAACCAAACAAAAAACATCATCTTTTATTCCCAGGAAAATAATTAATTAGACAGGAACATATCACTTCCCAGCTG
CCACTGACTTGCAGTCCTGTGAAAAGAGGCCAAGATTGCATCTGGCTTCATCTCCACTGCAGCAAAGCTTTGAGCTTTGGCTTTGAGGA
AGTGTCACACAGAGCCAGTTGCACACCAATGCCTGGACCTGGGGGTCTGAAAAGGAGGAGCAGGTAAACAGGCAACAAGCACCAAGGCC
CTCTGAGACAAAGCACCCTGCCTCCTGGTTGCTTTTACACCCCAGGTATGTCTATGCCATGCAGTGCAAGACCATCTGCTAGAACCTGG
AAGCAGCAGAGAGCAGCAGGCAGGATAACTGACCTCAGTAATGAGGCTGTACCAGCAGGACTGTGTCCTACCTGAGACCCAAGAAAGAC
TGTGTGTGTCAACCCACTGCACTGCAGCATCCTCTGCTCCCTTTTTGCCACTCCCTTGTACCACTGGCATCTAGCAAAGCAGTGCTTCG
ATGGTCAGTACCCCTGGGGGACAGCCATGGGATGGATCTCATACCTGTTTGGCAGAAGATGCTGTCTAATGATGACACAGGTCATGAGG
CAGGTCCAGTGCAGAGCCAGCCCTCTCGGCCTTGCTCCCTGAACACTCTCTGCCAGGTTTAAAAGGTGCTAAACTATTTAAAGGGAAAG
AAAAAAAGCTACAGAACTTTCTTGCAGCTTGAAGATCCATTTCTGGAATCAGATTACAAACAGAGATGGAAGCAAAGATCCTTCCGGTG
CCTCACTCCCCCCTCATGCTTTTTCTTGCCAAGCCCCTGTCAAAGGGCATTGTCCCTAATGCTATGGAAAATAAGAAGAAAACAAGCCT
TTTCACCTTCTTTTCATCCCTCTTGAATTTCTACTCTTCTGATCCTCACTGGCTGCCCTACTTTATTTTCTCTCTCCATTTCTATTTAA
CCTGTCTTCCTCTTTTTCTTAACCCACCCCCACAGCATATGGTACTGAGGGTCACTTAAGGACACAGCCCTTCCCTGCCCCTACTGCTT
GGGCTCATCCCAGCCCAAGGAGACAGCAGCCTCCCCCACACCACTGCCAGGAACTGGCACAGACAACAACTGGGACGGACAACATTTCC
TTTCTGAGGGGAAAGACAGTCGAACAAGGCTGTCACTGCCTTTCATTTTGAGGGGGACAGGTGGGACATGCTCAAGGAGAAGCCATTTC
ACCAGCCTGGGCACTGAGGAAAAACCAGAATTTTTTTTTGGTGGGCATCTTTTCCCATTGTCCCAAAATCCAGATGAGGAACCATCACC
CTGCAATCAAGCCCTGACTGTTGCCACCGTGGAAAGAGACTGGTAACCCCCAGTGACACCAGCGATCACACCGCTTCTGCTCACCCTCT

GAGCAGGAGACAAGTGTCATGGCTTCACATACAAGAAATAAGCCTCCAGGGCTAAATGAGCTGAGTTTGCTGGAGGGGGTATCCCATAC
TTTAACAGCAGCCAGAAAGCCAACTCCCTGCCGGAGACACAACCAGTGGTTGTCCTGACCGAGTTCTCCTTCAGTCGTGGGTGGTAAAT
GACTTAAATAGCCATGGGCAACCTCATCTTCTGCTTTTGGATTTGGAGGAAGCCCAGCTTGAATCAGGTCCTTCCCTCAGTAACACAGG
TAAAAATAAAGTCATGTTTGACCAGAGAGCAGCCCCGAATTTTGCCTGGTGTTAGATTTTTGCTTTAAAATTCAGTCCAACACGTCCAA
GCCCTGCTCCTGCTAGTGACAAAGAGAGAGGTTATTGAACAACAAAAGTAACTAAATGTTTGAGGAACATTTCAGTCCCCACAAAATGA
AATTGCTGAAGGAATTTGCACAGCTTCCCACAGTCTAGTTACTGAATTACTAAACCAGTTTCTCACAACCACTTACAAAGCAAAATTAT
AATTAACTGTAGGATCTGGTATTATGAGTACTCAAGAATGAGCTTTTACAAAATAGAAGCTAAAAGGTTTGCAGATCTGTTTCACATCT
GTGGCCCAGATATAATCAGCTCCTTCTTGTTCCCTGTTTTAAAGCATGCCCACAACATCTGACTTGCTGAAGAGAGGTCCAAAGAGCTA
TAATGTTCTGAATGTCTCTTAAAACTTTCAAAGAGGCTGAATCAAATGTGT

>CASQ2_Manacus_vitellinus_CDS
ATGAAGGCAACTTGCTGGATCCTGGCAGGTTTTTACCTGCTTTTCTGCTGCAAGGCAGAAGAGGGACTGAACTTCCCCACTTACGATGG
GAAAGACAGAGTGATCGACCTGAACGAGAAGAACTACAAGCAGGCTCTGAAGAAGTATGACATGCTCTGCCTGCTCTTCCATGAGCCTG
TGAGTTCTGACAAGGTCTCCCAAAAGCAATTCCAGATGATAGAGATGGTCCTGGAGCTGGCAGCTCAGGTCCTGGAGCCCAGAAGCATC
GGTTTTGGGATGGTGGACTCCAAGAAGGATGCCAAACTTGCCAAAAAATTAGGCTTGCTTGAAGAGGGAAGTCTCTATGTCTTTAAGGA
TGAGCGGTTGATCGAATTTGATGGGGAACTGGCCACAGATGTCTTGGTGGAATTCCTCTTGGATTTGCTAGAAGACCCCGTGGAGGTCA
TAAACAGCAAGCTGGAGCTTCAGGCCTTTGACCAGATCGATGATGAAATCAAACTCATCGGCTACTTTAAAGGAGAAGACTCTGAACAT
TTCAAGGCATTTGAAGAAGCTGCTGAACAATTCCAGCCGTACATCAAGTTCTTTGCTACTTTTGACAAAGGGGTTGCCAAGAAGCTAGG
TCTGAAGATGAATGAGGTGGACTTCTATGAACCATTTATGGATGAGCCTGTTCACATCCCTGATAAGCCTTACACAGAAGAGGAGCTGG
TTGAATTTGTGAGAGAGCACAGAAGGGCCACCTTGAGGAAGCTGCGCCCAGAGGACATGTTTGAGACGTGGGAGGATGACATGGAGGGT
ATCCACATCGTAGCCTTCGCTGAAGAAGACGACCCAGATGGTTTTGAGTTCCTGGAAGTTCTGAAGCAGGTTGCCAGGGACAACACCGA
TAATCCTGACCTGAGCATTGTCTGGATTGACCCTGATGACTTTCCTCTGCTGATCACTTACTGGGAGAAGACCTTCAAGATCGACTTGT
TCAGACCACAGATCGGGGTGGTGAATGTCACAGACGCTGACAGCATCTGGATGGACATCAGAGATGATGATGATCTGCCCACAGCTGAG
GAGCTGGAAGACTGGATAGAAGATGTGCTTTCTGGGAAGATAAATACTGAAGATGATGATGATGACGACGATGACGACGACGATGATGA
CGATGACGACGATGATGACGATGATGATGACGACGATGATGAAGACGACGATGATGACGATGACTAA

>CASQ2_Manacus_vitellinus_protein
MKATCWILAGFYLLFCCKAEEGLNFPTYDGKDRVIDLNEKNYKQALKKYDMLCLLFHEPVSSDKVSQKQFQMIEMVLELAAQVLEPRSI
GFGMVDSKKDAKLAKKLGLLEEGSLYVFKDERLIEFDGELATDVLVEFLLDLLEDPVEVINSKLELQAFDQIDDEIKLIGYFKGEDSEH
FKAFEEAAEQFQPYIKFFATFDKGVAKKLGLKMNEVDFYEPFMDEPVHIPDKPYTEEELVEFVREHRRATLRKLRPEDMFETWEDDMEG
IHIVAFAEEDDPDGFEFLEVLKQVARDNTDNPDLSIVWIDPDDFPLLITYWEKTFKIDLFRPQIGVVNVTDADSIWMDIRDDDDLPTAE
ELEDWIEDVLSGKINTEDDDDDDDDDDDDDDDDDDDDDDDDDDEDDDDDDD*

8.6 *Mionectes oleagineus* (ochre-bellied flycatcher)

From: Trinity *de novo* joint assembly of SRR18056689 and SRR18056690. CASQ1 from the complement of TRINITY_DN83_c0_g1_i18. CASQ2 from TRINITY_DN3423_c0_g1_i4.

>CASQ1_Mionectes_oleagineus_transcript_assembly
AGGAGCTGGAGCGGGGTTGGAAAAGGGGGGGGACACGGAGGGGACAGGGCTGAGCCCCCCAAGGGGGTCCCGGACCCCGCGGATCCCCC
CGAGCCGTGATGGGGGGACACCCACGCAGGGTCCGTGGTGGGAAGGAGCGTGTCGGGGTCCGTCTGTCCCCGCCGGGTCCGTCTGTCCC
CTCCTGGCGGGGCCGGAAGGGGCTGGGACCGTCCGGGTGTCCCTGCAGGGCTGTGTTTGACCGACACGCGTCCGTCTGTCCCCCACTCG
TGTCCGTCCCCGCTGCTCCCTGAGCCCCCTCCCTGCCTGTCCGTCCGTCTGTCCGTCCCTCTCTCCCTGTGTCCGTGTCCCCGCGTGTC
CGTGTCCCCCCCGATGTGGCTCTGGGCGTGGGCGCTGGCGCTGCTGGCTCTGAGCGCGGGGGTCCCGGGGGGGTCGGGCCAGGGGGGGC
TTCACTTACCGACCCTCCGCGGGCGGGGCCGGGAGGTCGCGGTGACCCCGCGGGACCACCGGGAGCTGCTGCGGCGCTTCCCGGGGCTG
GAGCTGCTGCCGCGGGACCCCCGGGACGGGGAACCCCGAGAGAAGGAGCTGGTGACGGAGGTGCCCCCCCAGGTGCCGGAGCAGCCGGT
GGCGCTGGTGGCAGAGGGGGTGGCGGAGGAGCAGCTCCGAAACTTCGGGGACATCGAGGAGGAGCCCAAAGTCATCGGGTACTTCCAGG
GACAGCACTCGGAGCGTTTCCGGTCCTTCGTGGCGGCCGCGCGGCACTTCCACCCGTCCGTGCCCTTCTTCGCCACCTTCGACAGCCGG
GCGGCCGAGAGGCTGAGGCTGAGGCTGAACCAGATCGATCTGTACCCCCCGTTCCAGGAGCTGCCCCGGACCCTCGGGGGGGGCCCCGG
GGACACCCCCACCATCGTGGCCTTCGTGGAGCAGAACAAGCGGGCCACGCTGAGGAAACTCAAACCCCAGACCCTGCACGAGGCCTGGG
AGGACGCAACAAACGGGATCCTCATCGTGGCCTTCGCAGAGGGGGATGAGCCGGATGGCTTCGAATTCCTGGAGATCCTGAAGGACGTG
GCCCGGGACAGCTCGGCCAAGCCCCAGCTCAGCATCCTCTGGATCGACCCCGACGATTTCCCGCCGCTCGTCCCTTACTGGAGGAAAT
CTTCGACCTCGACCTGTCCCGGCCCCAGCTGGGGGTGCTCAACGGCACCCACGCCAGCAGCGTGTGGTTGGAGATGGAGGAGGAGGATC
TGCCGGGCCCGGGGGACATCCACGAGTGGCTCCGGGGGGTCTTGGGGGGGCTCCTCGGCCCCGGGGGGGACAACGGGGACAGCGAGGAT
GAAGATGGTGAAGATGAGGGTGAGGATGATGAAGAAGATGAAGATGAAGATGATGAGGATGATGATGAGAATGATGATGATGATGATGA
CGATGATGAAGATGACGATGATTAGGCCATAAAATCCCCC

>CASQ1_Mionectes_oleagineus_transcript_CDS
ATGTGGCTCTGGGCGTGGGCGCTGGCGCTGCTGGCTCTGAGCGCGGGGGTCCCGGGGGGGTCGGGCCAGGGGGGGCTTCACTTACCGAC
CCTCCGCGGGCGGGGCCGGGAGGTCGCGGTGACCCCGCGGGACCACCGGGAGCTGCTGCGGCGCTTCCCGGGGCTGGAGCTGCTGCCGC
GGGACCCCCGGGACGGGGAACCCCGAGAGAAGGAGCTGGTGACGGAGGTGCCCCCCCAGGTGCCGGAGCAGCCGGTGGCGCTGGTGGCA
GAGGGGGTGGCGGAGGAGCAGCTCCGAAACTTCGGGGACATCGAGGAGGAGCCCAAAGTCATCGGGTACTTCCAGGGACAGCACTCGGA

GCGTTTCCGGTCCTTCGTGGCGGCCGCGCGGCACTTCCACCCGTCCGTGCCCTTCTTCGCCACCTTCGACAGCCGGGCGGCCGAGAGGC
TGAGGCTGAGGCTGAACCAGATCGATCTGTACCCCCCGTTCCAGGAGCTGCCCCGGACCCTCGGGGGGGGCCCCGGGGACACCCCCACC
ATCGTGGCCTTCGTGGAGCAGAACAAGCGGGCCACGCTGAGGAAACTCAAACCCCAGACCCTGCACGAGGCCTGGGAGGACGCAACAAA
CGGGATCCTCATCGTGGCCTTCGCAGAGGGGGATGAGCCGGATGGCTTCGAATTCCTGGAGATCCTGAAGGACGTGGCCCGGGACAGCT
CGGCCAAGCCCCAGCTCAGCATCCTCTGGATCGACCCCGACGATTTCCCGCCGCTCGTCCCTTACTGGGAGGAAATCTTCGACCTCGAC
CTGTCCCGGCCCCAGCTGGGGGTGCTCAACGGCACCCACGCCAGCAGCGTGTGGTTGGAGATGGAGGAGGAGGATCTGCCGGGCCCGGG
GGACATCCACGAGTGGCTCCGGGGGGTCTTGGGGGGGCTCCTCGGCCCCGGGGGGGACAACGGGGACAGCGAGGATGAAGATGGTGAAG
ATGAGGGTGAGGATGATGAAGAAGATGAAGATGAAGATGATGAGGATGATGATGAGAATGATGATGATGATGATGACGATGATGAAGAT
GACGATGATTAG

>CASQ1_Mionectes_oleagineus_protein
MWLWAWALALLALSAGVPGGSGQGGLHLPTLRGRGREVAVTPRDHRELLRRFPGLELLPRDPRDGEPREKELVTEVPPQVPEQPVALVA
EGVAEEQLRNFGDIEEEPKVIGYFQGQHSERFRSFVAAARHFHPSVPFFATFDSRAAERLRLRLNQIDLYPPFQELPRTLGGGPGDTPT
IVAFVEQNKRATLRKLKPQTLHEAWEDATNGILIVAFAEGDEPDGFEFLEILKDVARDSSAKPQLSILWIDPDDFPPLVPYWEEIFDLD
LSRPQLGVLNGTHASSVWLEMEEEDLPGPGDIHEWLRGVLGGLLGPGGDNGDSEDEDGEDEGEDDEEDEDEDDEDDDENDDDDDDDED
DDD*

>CASQ2_Mionectes_oleagineus_transcript_assembly
CTGACATCTTCCACTCCCTCCCTCTTGCTCGTTCCCTCTCTCTCAGATGATGCAGAGTGCCAGCAAGTCCCTCAATGGTGTCATATCTG
TCCTTTTTATTCATTGCAGGGTTTATTTTTAGCCTGAAACAACTGCTTCCTAAAAATGGAGTTCCTAATGACACGGGAGCTGGACACAG
CTATGTAAGGTATCCAAGGCTTGGCCTCACAGCTTCTCCTGTCTGTCTCTCTCTTTGTTGTGAGTCCAGGACAGCAAAGTTTGCTGCTC
CCCACCAGCCTTGCAGTGTTTGTGCTGATCATCAGGAAGGCAGCAGGAAGAGAAACTCAGTTCAAACTTCACCTGAAGCCTTGGTCTCC
CCCTCTTCCCAAATCAAGAAAGAGAGGGACATTTACCCACCCTTTGCTGCACTCCAAGGGCACCCCTATGAAGGCAACTTGCTGGATCC
TGGCAGGTTTTTACCTGCTTTTCTGCTGCAAGGCAGAAGAGGGACTGAACTTCCCCACTTACGATGGGAAAGACCGAGTGATCGACCTG
AACGAGAAGAACTACAAGCAGGCCCTGAAGAAGTATGACATGCTCTGCCTGCTCTTCCATGAGCCTGTGAGTTCTGACAAGATCTCCCA
AAAGCAGTTCCAGATGACAGAGATGGTCCTGGAGCTGGCAGCTCAGGTCCTGGAGCCCAGAAGCATCGGTTTTGGGATGGTGGACTCCA
AGAAGGACGCCAAACTTGCCAAAAAATTAGGCTTACTTGAAGAGGGAAGTCTCTATGTCTTTAAGGATGAGCGGTTGATTGAATTTGAT
GGGGAGCTGGCCACAGATGTCTTGGTGGAATTCCTCTTGGATTTGCTAGAAGACCCCGTGGAGGTCATAAACAGCAAGCTGGAGCTTCA
GGCCTTTGACCAGATCGATGATGAAATCAAACTCATCGGCTACTTCAAAGGAGAAGACTCTGAACATTTTAAGGCATTTGAAGAAGCTG
CTGAACAATTCCAGCCATACATCAAGTTCTTTGCTACTTTTGACAAAGGGGTTGCCAAGAAGCTAGGTCTGAAGATGAATGAGGTGGAC
TTCTATGAACCATTTATGGATGAGCCTGTTCACATCCCTGATAAGCCTTACACAGAAGAGGAGCTGGTTGAATTTGTGAGAGACCACAG
AAGGGCCACCTTGAGGAAGCTACGCCCAGAGGACATGTTTGAGACGTGGGAGGATGACATGGAGGGTATCCACATTGTAGCCTTCGCTG
AAGAAGACGACCCAGATGGTTTTGAGTTCCTGGAAATCCTGAAGCAGGTTGCCAGGGACAACACCGATAATCCTGACCTGAGCATTGTC
TGGATTGACCCTGATGACTTTCCTCTGCTGATCACTTACTGGGAGAAGACCTTCAAGATCGACTTGTTCAGACCACAGATCGGGGTGGT
GAATGTCACAGACGCTGACAGCATCTGGATGGACATCAGAGATGATGATGACCTGCCCACAGCTGAGGAGCTGGAAGACTGGATAGAGG
ATGTGCTTTCTGGGAAGATAAATACTGAAGATGATGATGACGATGATGATGATGATGACGACGATGACGACGACGATGATGATGATGAT
GACGATGACGACGATGATGATGATGACGACGACGACGATGACTAACTGTGACTCTGTGCAGTTTGACTTGTGGGGCCCAAGAGCCCTCC
CCCTGCGGCAGGTCCCTCCACTGGAAGGCCTGTGTTTGAGCGTCAGCAAGCACCGCTGCTCCTCTTTGCCCCCTGCCCTGCTCCCCCTT
CCCCTGCCCTTGCCAGGACCCCCTGGCCTGATTCTCAGTGGTGCTGAGCCACCAGGACTGCCCCTGCGGTGGGCGGTGCCTGCAGGCAC
CCAGCACCGCTGCAAATCATGCCTCCTTAGTAAGGACAATAGGAATCTGCAGGGAATCTGCCCCGAGTGTCCCAGGGCGGGAGAAAAGT
GCCTGCCTGCTTGCTGCCAGAGGGATGCAGGGAACCACTTTGTGTTTTCCATGTTCATCAGCACAGCACTTAACATCCAAAGACCAAAT
CTCCCTTCAAGACATTGAGAAGGCCGCTGTAAAGTTTAGTCATTTTATTAAACACAATGCCCTTTGCAGATACTCTGCCAAGACCACTC
CATCCTGCAAGTCCTGCTGTGGGAGTCCCTAGCTACTCATTAAAATGCTAGGAGCAGCAAAATGCTTTGGGAGGGGAGGAAGGTTTTAG
GGATGCTATTAAAGTTTTCAGTGATTCCACATGACAACAGGATATTCTTGTCTGGCAGTGTGATGCAAAACCGAGCACCATGCCCCCTG
TCTGAACCACTTTTCTTGAAAGCTCGGGCCATGGTGAGGGGAAGTGCAAGTGGTCTGATACGAGGCATACACGCTCGTGAGTTTCTGAA
CTGCTGGAAATGCTGATCCAGGTCTGAGCCCAGTTTAAGTCCAAACTCTTGAGTTTGAAGCCCATGGAACTGAGCCAAGCCAATGAAAA
CATCCGGAGGGGCCCACCCTGAAGCTCTCCTATTCCCCATGACCTTCCAGCTTTAGCTGACTCTTCACTCAAAGCCCACATAGCATCTA
TTCCCACGACCAAGATGTTGGGAGGGAACACAGCCATCCAACTATATTAGATATTGGATGAGGTACGGCAGGCAATGAGCAGCGAATGG
ATTCAAGTTGTTTGGCTTCTAAGTATTTAGTGGCACAGTTACTCCCTGCTTAGAGACTCAGATGTCCCACAGCAACCTGTGAGAACCAT
CTTTTGACTGCAGGAGCTCAGGGTGCCAGTTTGCAGTCAAAGGACTCACTCATGAAGGTCTGTGACACAGTCCTGGTGAGCGCAGCACC
ATCCACATGCAGGTCACTGTGGCAGCACTAAGTCTCCCTGCTCTCTCATGCCAGCAGATTTCAATTGTCTTAGCTTATTAGGTTTGACA
ATAGTGCAATGAACAGAGAATTCGGAGATAGTTTTATCTCTATAAATACATTTATAAGGATATTGTTCAGGCTTGCTAGCAGAGGTAGG
GTATGCATCTCCTTTCTTGCTACAGATGGAAAATGTTGTCCTGACCTTTCTTTTATTGGCTTACGTCAGAGGTAAGTGATATTAGGGGA
AATAACAGCGTGCGGGACAGGAGAATCAGAGCTCATTACCCAAGTGTGAATCAGCCTGATGAGAAACAAGGTTCACACAACATATATTT
CCTTTATCAGATGAATCGCAAAGCAAGATTTTATAGATTTTTTTTTTTTTT

>CASQ2_Mionectes_oleagineus_CDS
ATGAAGGCAACTTGCTGGATCCTGGCAGGTTTTTACCTGCTTTTCTGCTGCAAGGCAGAAGAGGGACTGAACTTCCCCACTTACGATGG
GAAAGACCGAGTGATCGACCTGAACGAGAAGAACTACAAGCAGGCCCTGAAGAAGTATGACATGCTCTGCCTGCTCTTCCATGAGCCTG
TGAGTTCTGACAAGATCTCCCAAAAGCAGTTCCAGATGACAGAGATGGTCCTGGAGCTGGCAGCTCAGGTCCTGGAGCCCAGAAGCATC
GGTTTTGGGATGGTGGACTCCAAGAAGGACGCCAAACTTGCCAAAAAATTAGGCTTACTTGAAGAGGGAAGTCTCTATGTCTTTAAGGA

TGAGCGGTTGATTGAATTTGATGGGGAGCTGGCCACAGATGTCTTGGTGGAATTCCTCTTGGATTTGCTAGAAGACCCCGTGGAGGTCA
TAAACAGCAAGCTGGAGCTTCAGGCCTTTGACCAGATCGATGATGAAATCAAACTCATCGGCTACTTCAAAGGAGAAGACTCTGAACAT
TTTAAGGCATTTGAAGAAGCTGCTGAACAATTCCAGCCATACATCAAGTTCTTTGCTACTTTTGACAAAGGGGTTGCCAAGAAGCTAGG
TCTGAAGATGAATGAGGTGGACTTCTATGAACCATTTATGGATGAGCCTGTTCACATCCCTGATAAGCCTTACACAGAAGAGGAGCTGG
TTGAATTTGTGAGAGACCACAGAAGGGCCACCTTGAGGAAGCTACGCCCAGAGGACATGTTTGAGACGTGGGAGGATGACATGGAGGGT
ATCCACATTGTAGCCTTCGCTGAAGAAGACGACCCAGATGGTTTTGAGTTCCTGGAAATCCTGAAGCAGGTTGCCAGGGACAACACCGA
TAATCCTGACCTGAGCATTGTCTGGATTGACCCTGATGACTTTCCTCTGCTGATCACTTACTGGGAGAAGACCTTCAAGATCGACTTGT
TCAGACCACAGATCGGGGTGGTGAATGTCACAGACGCTGACAGCATCTGGATGGACATCAGAGATGATGATGACCTGCCCACAGCTGAG
GAGCTGGAAGACTGGATAGAGGATGTGCTTTCTGGGAAGATAAATACTGAAGATGATGATGACGATGATGATGATGATGACGACGATGA
CGACGACGATGATGATGATGATGACGATGACGACGATGATGATGATGACGACGACGACGATGACTAA

>CASQ2_Mionectes_oleagineus_protein
MKATCWILAGFYLLFCCKAEEGLNFPTYDGKDRVIDLNEKNYKQALKKYDMLCLLFHEPVSSDKISQKQFQMTEMVLELAAQVLEPRSI
GFGMVDSKKDAKLAKKLGLLEEGSLYVFKDERLIEFDGELATDVLVEFLLDLLEDPVEVINSKLELQAFDQIDDEIKLIGYFKGEDSEH
FKAFEEAAEQFQPYIKFFATFDKGVAKKLGLKMNEVDFYEPFMDEPVHIPDKPYTEEELVEFVRDHRRATLRKLRPEDMFETWEDDMEG
IHIVAFAEEDDPDGFEFLEILKQVARDNTDNPDLSIVWIDPDDFPLLITYWEKTFKIDLFRPQIGVVNVTDADSIWMDIRDDDDLPTAE
ELEDWIEDVLSGKINTEDDDDDDDDDDDDDDDDDDDDDDDDDDDDDDDDDD*

8.7 *Pseudopipra pipra* (white-crowned manakin)

From: Trinity *de novo* joint assembly of SRR18056705 and SRR18056706. CASQ1 from the complement of TRINITY_DN9759_c0_g2_i5. CASQ2 from the complement of TRINITY_DN2004_c1_g1_i1.

>CASQ1_Pseudopipra_pipra_transcript_assembly
GCTGCGGGAGCTGCGGGAGCTGCGGGAGCTGCGGGAGCTGCGGGAGCTGCTGCGGCGCTTCCCGGGGCTGGCGCTGCTCCACAGGGACC
CCCGGGACAGGGACCCCCGGGACGGGGACCCCCGGGACAGGGACACCCGGGACAGGGAACCCCGGGACAGGGACCCCCGGGACAGGGAC
ACCCGGGACAGGGACCCCCGCGACAGGGACCCCCGGGAGAAGGAGCCGGTGATGGAGGTGCCGCAGGTGCCCGTGAGGGAGGTGACATC
GGAGCAGGAGCTCCAGGACTTCGAGAACGTGGAGGAGGAGCCCAAAGTGATCGGGCACTTCCAGGGACCCAGCTCGGAGCATTTCGGCT
CCTTCGCGGCGGCCGCGCGGAACCTTCACCCCCCCGTGCCCTTCTTCGTCACCTTCGACCCACAGGTGGCCCGGAGGCTGCGGCTGCG
GCTGAACCAGATTGACCTGTACCCCCCCTTCCAGGACACCCCCCGCACCCTCGGGGGGGGCCCCGCGGACCCCCCCCCAGACCCCCCC
GACATCGTGGCCTTCGTGGAGAACAACCAGCGGTCCCCGCTGAGGAAACTCAAACCCCAAACCCCGCTCGAGACCTGGGAGGAGGCGG
CCGGAGGGACCCTCGTGGTGGCCTTTGCGGAGGGGGACGAGCCGGATGGTTTCGAATTCCTGGCGATCCTGAAGGACGTGGCCCGGGA
CAACCCCGAGCTGCGAATCCTCTGGATCGACCCCGACGATTTCCCGACGCTCGTCCCTTACTGGGAGGAAATTTTCGACCTCGACCTT
TCCCGGCCCCAGCTCGGGGTCATCGACGGCACCGACGGTGCCGGGGTGTGGCTGGAGATGCAGCAGGAGGAGGAGGAGGAGGAGGAGG
A

>CASQ1_Pseudopipra_pipra_CDS_partial
GCTGCGGGAGCTGCGGGAGCTGCGGGAGCTGCGGGAGCTGCGGGAGCTGCTGCGGCGCTTCCCGGGGCTGGCGCTGCTCCACAGGGACC
CCCGGGACAGGGACCCCCGGGACGGGGACCCCCGGGACAGGGACACCCGGGACAGGGAACCCCGGGACAGGGACCCCCGGGACAGGGAC
ACCCGGGACAGGGACCCCCGCGACAGGGACCCCCGGGAGAAGGAGCCGGTGATGGAGGTGCCGCAGGTGCCCGTGAGGGAGGTGACATC
GGAGCAGGAGCTCCAGGACTTCGAGAACGTGGAGGAGGAGCCCAAAGTGATCGGGCACTTCCAGGGACCCAGCTCGGAGCATTTCGGCT
CCTTCGCGGCGGCCGCGCGGAACCTTCACCCCCCCGTGCCCTTCTTCGTCACCTTCGACCCACAGGTGGCCCGGAGGCTGCGGCTGCG
GCTGAACCAGATTGACCTGTACCCCCCCTTCCAGGACACCCCCCGCACCCTCGGGGGGGGCCCCGCGGACCCCCCCCCAGACCCCCCC
GACATCGTGGCCTTCGTGGAGAACAACCAGCGGTCCCCGCTGAGGAAACTCAAACCCCAAACCCCGCTCGAGACCTGGGAGGAGGCGG
CCGGAGGGACCCTCGTGGTGGCCTTTGCGGAGGGGGACGAGCCGGATGGTTTCGAATTCCTGGCGATCCTGAAGGACGTGGCCCGGGA
CAACCCCGAGCTGCGAATCCTCTGGATCGACCCCGACGATTTCCCGACGCTCGTCCCTTACTGGGAGGAAATTTTCGACCTCGACCTT
TCCCGGCCCCAGCTCGGGGTCATCGACGGCACCGACGGTGCCGGGGTGTGGCTGGAGATGCAGCAGGAGGAGGAGGAGGAGGAGGAGG
A

>CASQ1_Pseudopipra_pipra_protein_partial
XLRELRELRELRELRELLRRFPGLALLHRDPRDRDPRDGDPRDRDTRDREPRDRDPRDRDTRDRDPRDRDPREKEPVMEVPQVPVREVT
SEQELQDFENVEEEPKVIGHFQGPSSEHFGSFAAAARNLHPPVPFFVTFDPQVARRLRLRLNQIDLYPPFQDTPRTLGGGPADPPPDPP
DIVAFVENNQRSPLRKLKPQTPLETWEEAAGGTLVVAFAEGDEPDGFEFLAILKDVARDNPELRILWIDPDDFPTLVPYWEEIFDLDLS
RPQLGVIDGTDGAGVWLEMQQEEEEEEEX

>CASQ2_Pseudopipra_pipra_transcript_assembly
CCTCAATAGTGTCATATCTGTCCTTTTTATTCATTGCAGGGTTTATTTTAAGCCTGAAACAACTGCTTCCTAAAAATGGAGTTCCTAAT
GACACGGGAGCTGGACACAGCTATGTAAGGTATCCAGGGCTTGGCCTCACAGATTCTCCTGTCTGTCTCTCTCTTTGTTGTGAGTCCAG
GACAGCAAAGTTTGCTGCTCCCCACCAGCCTTGCAGTGTTTGGGCTGATCACCAGGAAGGCAGCAGAAAGAGAAACTCAGTTCAAACTT
CACCTGAAGCCTTGGTCTCCCCCTCTTCCCAAATCAAGAAAGAGAGGGACATTTACCCACCCTTTGCTGCACTCCAAGGGCACCCCTAT
GAAGGCAACTTGCTGGATCCTGGCAGGTTTTTACCTGCTTTTCTGCTGCAAGGCAGAAGAGGGACTGAACTTCCCCACTTACGATGGGA
AAGACCGAGTGATCGACCTGAACGAGAAGAACTACAAGCAGGCTCTGAAGAAGTATGACATGCTCTGCCTGCTCTTCCATGAGCCTGTG
AGTTCTGACAAGATCTCCCAAAAGCAGTTCCAGATGACAGAGATGGTCCTGGAGCTGGCAGCTCAGGTCCTGGAGCCCAGAAGCATCGG

TTTTGGGATGGTAGACTCCAAGAAGGATGCCAAACTTGCCAAAAAATTAGGCTTGCTAGAAGAGGGAAGTCTCTATGTCTTTAAGGATG
AGCGGTTGATCGAATTTGATGGGGAACTGGCCACAGATGTCTTGGTGGAATTCCTCTTGGATTTGCTAGAAGACCCCGTGGAGGTCATA
AACAGCAAGCTGGAGCTTCAGGCCTTTGACCAGATCGATGATGAAATCAAACTCATCGGCTACTTTAAAGGAGAAGACTCTGAACATTT
CAAGGCATTTGAAGAAGCTGCTGAACAATTCCAGCCGTACATCAAGTTCTTTGCTACTTTTGACAAAGGGGTTGCCAAGAAGCTAGGTC
TGAAGATGAATGAGGTGGACTTCTATGAACCATTTATGGATGAGCCTGTTCACATCCCTGATAAGCCTTACACAGAAGAGGAGCTGGTT
GAATTTGTGAGAGAGCACAGAAGGGCCACCTTGAGGAAGCTGCGCCCAGAAGACATGTTCGAGACGTGGGAGGATGACATGGAGGGTAT
CCACATCGTAGCCTTCGCTGAAGAAGATGACCCAGACGGTTTTGAGTTCCTGGAAATTCTGAAGCAGGTTGCCAGGGACAACACCGATA
ATCCTGACCTGAGCATTGTCTGGATTGACCCTGATGACTTTCCTCTGCTGATCACTTACTGGGAGAAGACCTTCAAGATCGACTTGTTC
AGACCACAGATCGGGGTGGTGAATGTCACAGACGCTGACAGCATCTGGATGGACATCAGAGATGATGATGATCTGCCCACAGCTGAGGA
GCTGGAAGACTGGATAGAAGATGTGCTTTCTGGGAAGATAAATACTGAAGATGATGATGATGATGACGATGACGACGACGATGACGACG
ATGACGACGATGATGACGATGATGATGACGACGACGATGATGACGACGATGATGACGATGACTAACTGTGACTCTGTGCAGTTTGACTT
GTGGGGCCCGAGAGCCCTCCCCCTGCGGCAGGTCCTTCCATTGGAAGGCCTGTGTTTGAGCAAGCACCGCTGCTCCTATTTGCCCCCTG
CCCTGCTCCCCCTGCCCCTGCCCTTGCCGGGACCCCCTGGCCTGATTCTCAGTGGTGCTGAGCCACCGGGACTGCCCCTGCGGTGGGCA
GTGCCTGCAGGCACCCAGCACCGCTGCAAATCATGCCTCCTTAGTAAGGACAATAGGAATCTGCAGGGAATCTGCCCCGAGTGTCCCAG
GGCAGGAGAAAAGTGCCTGCCTGCTTGCTGCCAGAGGGACGCAGGGAGCCAGTTTGTGTTTTCCATGTTCATCAGCCCAGCACTTAACA
TCCAAAGACCAAATCTCCCTTCAAGACATTGAGAAGGCCACTGTAAAGTTTAGTCCTTTTATTAAACACAATGCCCTTTGCAGATACTC
TGCCAAGACCACTCAATCCTGCAAGTCCTGCTGCGGGAGTCCCTAGCTAACGCTAGGAGCAGCAAAATGCTTTGGGAGGGGGAGGAAGGT
TTTAGGGATGCTATTAAAGTTTTCAGTGATTCCACATAACAACAGGATATTCTTGTCTGGCAGTGTGATGCAAAACCAGTCACCATGCC
CTCTGTCTGAACCTCTTTTCTTGAAAGCTCGGGCCATGGTTGGGGAAGTGCAGATGGTCTGATAGGAGGCATAAAAGCTCGCAAGTTCC
TGAACTGCTGGAAAGGCTGATCCAGGTCTGAGTCCAAACTCTTGAGTTTGAAGACCATGGCACTGAGTCAAGCCAATAGAAACATCCGT
AGGGGCCCACCCTGAAGCTCTCCTATTCCCCATGACCTTCTGGCTTTAGCTGACTCTTCACTCAAAGCCCACGTAGCATCTATTCCCAT
GACCAAGATGTTGAGAGGGAACACACCCATCCAACTACATTTGATATTGGATGAGGTACGTCGGGCAATGAGGGGCAAATGGATTCAAG
TTGTTTGGCTTTAAGTATTTAGTTGCACAGTTACTCCCTGCTTAGAGACGTGGATGTCCCATAGTAGCCTGTGAGAAGCATCTTTTGAC
TGCAGGAGCTCAGGGTGCCAGTTTGCAGTCAAGGGGGTCGCTCACAAAGCAAAGGGTCTGCAACACAGTCCTGGTGAGAGCAGCACCAT
CCACATGCAGGTCACTGTGGCAGCATTAAATCTCCCTGCTTTCCCATGCCAGCAGATTTCAATTGTGTTAGCTTATTAGGTTTGACAAT
AGTACAATGAACAGAGAGTTCGGAGATAGTTTTATCTCTATATACACATTTATAAGGATGTTCTCTGGTTGGTCAGGCTTGTTAGCATA
GGTAGGGTATGCATCTCCTTTCTTGCTACAGATGGAAAACATTGTCCTGACTGTTCTTTTACTGGCTTATGTCAGAGGTAAGTGATATT
AGAGGAAATAACAGTGTGAGGGACAGGAGAATCAGAGCTCATTACACAAGTGTGAATCAGCCTGATGAGAAACAAGGTTCACACAGTAT
ATATTTCCTTTATCAGATGAACCACAAAGCAAGATTTTATAGATTTTTTTTTTTGCTGCCTGTTGATCAGTCTCTTTGACTCTCCTCTA
CATTAGCTGAAACAGAAATTTGAAACTACTGGTATAAAAAAGCTGAAGTATTGATATATTCCTATGTATGCCATATCCCTTGGTAGAAA
ATGCCAGTATGAACAACACATGGCTGGATATCTATATATCTCTATGCATATGAATGAATACTGTGCTAATATGGCATATGACTGTAGGC
CAAGTTAGCTCCTCATTGAATATAAAACATTCAACATGTGCTTTTTAGACACAGCTATTAGAAGAATAGACAAGGCAGCAAAGCCCCCA
GGCCAACTTTTCATGCTTTCTTGCTTCCTTATGATACATAAGCACTGCCTGGAAGACGAACCAACTTTTTGGGGGAAACCATTCACCTG
GTTGCTCTGGGGTGACGAACAACTTTCAAAAGCCTTGAAATGCAGAGTGCTCTCAGCATGGAGGTTCCCTTCTCCTTTTCTCTCATATT
TGCCTCTCTCCCGGCCAACAGGATGCCCAGCACTCTCCTTCTACTCTTTCCTTTGAGCTCTTTCCTCAAGAGGTTTGCTGGAAGACCCT
GGCTCAGAGAGAGAGGGAGCAGGCAGGACTCAAGGTCTCATTCATGCACACTGGTAAAGAGCAAGAAGAGGCAAAACTGGCCATCTAGT
GGTCTCCCAGTCTGCAATTAAGGGACAACTACTTACCTGAGAAAAATACATGCTTATTTTTACCTTGGGGCTATTCCTAGGAGTTAAAC
ATGCCCAGTTTCACCCTTCATAAGGCAACAGCCTGCACAGGCCCTGCAGCTCCATATCAGTTACTCTAAATACGAAGAATAAATGAAGA
GCTGCTCTACTGCCTTTCCTAACTAGCACAGTCTCCGGACATCTCATCTACCTGGATACTTTCCATGTAGCTCCCCTCCTGCTGTGGAT
GCCTGTCTATTCTAATGTGCCCTTCTCCAGACGCACTGAGAGGCACAGCTTACCCAGGCAGAGCAGGCAGACCCTGCAAATAATGCTAT
GGACACGTTCAAGTGCACAGGTGTTTCACAGGCATTCATATATTTCAAAGCAACAGAGCTCTGGGGGAAAAATAGAATAAATAATGTAG
AAGTGTAGAGGTTGAGCTCATATAGGGGCTGGTGGACTGGTGCCAGTGTTTTATGGACAGTTTTCCTTGAGTTATGAAAGGGCTAATGC
TACAGCTGCCACTCCTTCGTGGTGCCAGTGAAGTCCTTGATGGGGCTGCCCCTTTCTTTCTTGCCTAATCCCCCCAGTACAGTTTTCATC
ATTCTCTTTGCCCACAGACAGGAACTGTTTATTATCAGCTTTTAGGAGAAACACATGAAAAGTTAAGAAATGTTGCAGCAGAGGAAGGA
TTCCAGCAATGAGGATGGATGGGCAGCAAACCCATTTACTTGTAACACTCCATCTGCCCAAGATCCTAGCAACCAAACTGCTGAATGAG
GCAATACCTCTTGTGAAGGAGATGGGAGCTTAAACTTGAGTGACTTCCTTGCAGGGATCAGTAAAACAGTGGTATGGGGAGGACACACA
GGACTCCAGGCACAGGTATTCCCGGGCTCCTCTGTCAGAACTAGGTACAACTGGCATTTGAAAGGCAGTGGTGGCCCTGAGGAACTAAG
TCAGCCTAACTCCCCCCACTACACACTGACAAGCAAAAGGTTGATTTGGGTGAGGAACCTGCAAAAAAACCCTTATTAAGAAAAAAAAAA
AGAAACTGTGTACATGAGCAGCACTGAGTGTTTCTGCAAGCACATCTGTTAAGACTGTCCCTCTCTTCAAGAATAGTTCTTTTTCTCCA
GCATGTTAATAAGACATATTAAATGAGTCTTAACACACATAAAGACAAGTAACTGGATTTTTCCCTCATCCAAATGTGAGTACTTCAAG
CTGATCAGTTACTTCATAGAATCATTCAATCATTTTTGTTGGAAAAGACCCCTAAGATCATTGAGTCCAACCATTAATCCACCACTGCC
AAGTAAGTACCCAAAATAACCCAAAAGATAGTCTTACCAGCCCCCAATGACAGTAAGGACCCCACCCCCCCACAAGTGATACATGGCAA
GAACACCAAGTTACACCACCACCTCCTCCCAGTCACCAGACACGGGGCCAGAGGCCACAGCCAAGGGATTTGTTTGGTGTGCTGTACCA
CAGGCCACCCCGTGCTGCTCATGGGCAACAACCAAGGCAGATGGAAATCCACCAGGAGCCACATCCTGCAGCTATCATACTGCAGCAGG
GAGGAGAAAGTTTCTGCAGTGGGCCCAAGGTCACTGACGT

>CASQ2_Pseudopipra_pipra_CDS
ATGAAGGCAACTTGCTGGATCCTGGCAGGTTTTTACCTGCTTTTCTGCTGCAAGGCAGAAGAGGGACTGAACTTCCCCACTTACGATGG
GAAAGACCGAGTGATCGACCTGAACGAGAAGAACTACAAGCAGGCTCTGAAGAAGTATGACATGCTCTGCCTGCTCTTCCATGAGCCTG
TGAGTTCTGACAAGATCTCCCAAAAGCAGTTCCAGATGACAGAGATGGTCCTGGAGCTGGCAGCTCAGGTCCTGGAGCCCAGAAGCATC

GGTTTTGGGATGGTAGACTCCAAGAAGGATGCCAAACTTGCCAAAAAATTAGGCTTGCTAGAAGAGGGAAGTCTCTATGTCTTTAAGGA
TGAGCGGTTGATCGAATTTGATGGGGAACTGGCCACAGATGTCTTGGTGGAATTCCTCTTGGATTTGCTAGAAGACCCCGTGGAGGTCA
TAAACAGCAAGCTGGAGCTTCAGGCCTTTGACCAGATCGATGATGAAATCAAACTCATCGGCTACTTTAAAGGAGAAGACTCTGAACAT
TTCAAGGCATTTGAAGAAGCTGCTGAACAATTCCAGCCGTACATCAAGTTCTTTGCTACTTTTGACAAAGGGGTTGCCAAGAAGCTAGG
TCTGAAGATGAATGAGGTGGACTTCTATGAACCATTTATGGATGAGCCTGTTCACATCCCTGATAAGCCTTACACAGAAGAGGAGCTGG
TTGAATTTGTGAGAGAGCACAGAAGGGCCACCTTGAGGAAGCTGCGCCCAGAAGACATGTTCGAGACGTGGGAGGATGACATGGAGGGT
ATCCACATCGTAGCCTTCGCTGAAGAAGATGACCCAGACGGTTTTGAGTTCCTGGAAATTCTGAAGCAGGTTGCCAGGGACAACACCGA
TAATCCTGACCTGAGCATTGTCTGGATTGACCCTGATGACTTTCCTCTGCTGATCACTTACTGGGAGAAGACCTTCAAGATCGACTTGT
TCAGACCACAGATCGGGGTGGTGAATGTCACAGACGCTGACAGCATCTGGATGGACATCAGAGATGATGATGATCTGCCCACAGCTGAG
GAGCTGGAAGACTGGATAGAAGATGTGCTTTCTGGGAAGATAAATACTGAAGATGATGATGATGATGACGATGACGACGACGATGACGA
CGATGACGACGATGATGACGATGATGATGACGACGACGATGATGACGACGATGATGACGATGACTAA

>CASQ2_Pseudopipra_pipra_protein
MKATCWILAGFYLLFCCKAEEGLNFPTYDGKDRVIDLNEKNYKQALKKYDMLCLLFHEPVSSDKISQKQFQMTEMVLELAAQVLEPRSI
GFGMVDSKKDAKLAKKLGLLEEGSLYVFKDERLIEFDGELATDVLVEFLLDLLEDPVEVINSKLELQAFDQIDDEIKLIGYFKGEDSEH
FKAFEEAAEQFQPYIKFFATFDKGVAKKLGLKMNEVDFYEPFMDEPVHIPDKPYTEEELVEFVREHRRATLRKLRPEDMFETWEDDMEG
IHIVAFAEEDDPDGFEFLEILKQVARDNTDNPDLSIVWIDPDDFPLLITYWEKTFKIDLFRPQIGVVNVTDADSIWMDIRDDDDLPTAE
ELEDWIEDVLSGKINTEDDDDDDDDDDDDDDDDDDDDDDDDDDDDDDDD*

8.8 *Xenopipo atronitens* (black manakin)

From: Trinity *de novo* joint assembly of SRR18056705 and SRR18056706. CASQ1 from the complement of TRINITY_DN743_c0_g1_i11. CASQ2 from TRINITY_DN673_c0_g1_i1.

>CASQ1_Xenopipo_atronitens_transcript_assembly
CCGTGTGTCCATCCCCCTGTCCCCGTGTCCGTGTGTCCATCCCCGTGTCCACCCCCGTGTCCGTGTGTCCATCCCCGTGTCCATCCCCG
TGTCCGTGTGTCCATCCCAGTGTCCCCCGTGTCCGTGTGTCCATCCCCGTGTCCGTGTGTCCATCCCCGTGTCCATCCCCGTGTCCGTG
TGTCCATCCCCCTGTCCCTGTGTCTGTGTGTCCATCCCGGTGTCCCCGATGTCCGTCTGTCGGGGGGCTCTGGCCGCGCTGTCGCTGCT
GTCGCTGTGCTCGGTGGTCCCGGGGGTCTCAGCCCGGGGGGGGCTTCACTTGCCCACCCCCGGCGGGCGGGGCCGGGGGGTCGCCGCGA
CCCCGCGGGAGCTGCGGGAGCTTCTGCGGCGCTTCCCGGGGCTGGCGCTGCTCCCGAGGGACCCCCGCGACGGGGACACCCGAGAATGG
GACCCCCGGGACGGGGACCCCCAGGACAAGGAGCTGGTGATGGAGGTGCCACAGGTGCCCGTGAGGGAGGTGACATCGGAGCGGGAGCT
CCAGGACTTCGAGAACCTGGAGGAGGAGCCAAAGGTGATCGGGTACTTCCGGGGACCGAGCTCGGAGCATTTCGGCTCCTTCGCTGTGG
CCGCGCGGAACCTTCACCCCTCCGTGCCCTTCTTCGTCACCTTCGACGGACAGGTGGCCCGGAGGCTGCGGCTGCGGCTGAACCAGCTG
GACCTGTACCCCCCCTTCCAGGACACCCCCCGGACCCTCGGGGGGGGCCCCGCAGACCCCCCCCCAGACCCCCCCGCCATCGTGGCCTT
CGTGGAGAACAACCAGCGGTCCCCGCTGAGGAAACTCAAACCCCAAACCCCGCACGAGACCTGGGAGGAGGCGACAGGAGGGACCCTCG
TGGTGGCCTTTGCGGAGGGGGATGAGCCGGATGGCTTCGAATTCCTGGCGATCCTGAAGGACGTGGCCCGGGACAACCCCGAGCTGCGA
ATCCTCTGGATCGACCCCGACGATTTCCCGCCGCTCGTCCCTTACTGGGAGGAAATTTTCGACCTCGACCTGTCCCGGCCCCAGCTCGG
GGTCATCCACGGGACCGACGGTGTCAGTGTGTGGCTGGAGATGCCGGAGGAGGAGGAGGAGGAGGAGGAAGATCTGCCGGGGCCGG
GGG

>CASQ1_Xenopipo_atronitens_CDS_partial
ATGTCCGTCTGTCGGGGGGCTCTGGCCGCGCTGTCGCTGCTGTCGCTGTGCTCGGTGGTCCCGGGGGTCTCAGCCCGGGGGGGGCTTCA
CTTGCCCACCCCCGGCGGGCGGGGCCGGGGGGTCGCCGCGACCCCGCGGGAGCTGCGGGAGCTTCTGCGGCGCTTCCCGGGGCTGGCGC
TGCTCCCGAGGGACCCCCGCGACGGGGACACCCGAGAATGGGACCCCCGGGACGGGGACCCCCAGGACAAGGAGCTGGTGATGGAGGTG
CCACAGGTGCCCGTGAGGGAGGTGACATCGGAGCGGGAGCTCCAGGACTTCGAGAACCTGGAGGAGGAGCCAAAGGTGATCGGGTACTT
CCGGGGACCGAGCTCGGAGCATTTCGGCTCCTTCGCTGTGGCCGCGCGGAACCTTCACCCCTCCGTGCCCTTCTTCGTCACCTTCGACG
GACAGGTGGCCCGGAGGCTGCGGCTGCGGCTGAACCAGCTGGACCTGTACCCCCCCTTCCAGGACACCCCCCGGACCCTCGGGGGGGGC
CCCGCAGACCCCCCCCCAGACCCCCCCGCCATCGTGGCCTTCGTGGAGAACAACCAGCGGTCCCCGCTGAGGAAACTCAAACCCCAAAC
CCCGCACGAGACCTGGGAGGAGGCGACAGGAGGGACCCTCGTGGTGGCCTTTGCGGAGGGGGATGAGCCGGATGGCTTCGAATTCCTGG
CGATCCTGAAGGACGTGGCCCGGGACAACCCCGAGCTGCGAATCCTCTGGATCGACCCCGACGATTTCCCGCCGCTCGTCCCTTACTGG
GAGGAAATTTTCGACCTCGACCTGTCCCGGCCCCAGCTCGGGGTCATCCACGGGACCGACGGTGTCAGTGTGTGGCTGGAGATGCCGGA
GGAGGAGGAGGAGGAGGAGGAAGATCTGCCGGGGCCGGGGG

>CASQ1_Xenopipo_atronitens_protein_partial
MSVCRGALAALSLLSLCSVVPGVSARGGLHLPTPGGRGRGVAATPRELRELLRRFPGLALLPRDPRDGDTREWDPRDGDPQDKELVMEV
PQVPVREVTSERELQDFENLEEEPKVIGYFRGPSSEHFGSFAVAARNLHPSVPFFVTFDGQVARRLRLRLNQLDLYPPFQDTPRTLGGG
PADPPPDPPAIVAFVENNQRSPLRKLKPQTPHETWEEATGGTLVVAFAEGDEPDGFEFLAILKDVARDNPELRILWIDPDDFPPLVPYW
EEIFDLDLSRPQLGVIHGTDGVSVWLEMPEEEEEEEEEDLPGPGX

>CASQ2_Xenopipo_atronitens_transcript_assembly
CAGCAAGTCCCTCAATAGTGTCATATCTGTCCTTTTTATTCATTGCAGGGTTTATTTTTAGCCTGAAACAACTGCTTCCTAAAAATGGA
GTTCCTAATGACACGGGAGCTGGACACAGCTATGTAAGGTATCCAGGGCTTGGCCTCACAGCTTCTCCTGTCTGTCTCTCTCTTTGTTG
TGAGTCCAGGACAGCAAAGTTTGCTGCTCCCCACCAGCCTTGCAGTGTTTGGGCTGATCACCAGGAAGGCAGCAGAAAGAGAAACTCAG

TTCAAACCTCACCTGAAGCCTTGGTCTCCCCCTCTTCCCAAATCAAGAAAGAGAGGGACATTTACCCACCCTTTGCTGCACTCCAAGGG
CACCCCTATGAAGGCAACTTGCTGGATCCTGGCAGGTTTTTACCTGCTTTTCTGCTGCAAGGCAGAAGAGGGACTGAACTTCCCCACTT
ACGATGGGAAAGACCGAGTGATCGACCTGAATGAGAAGAACTACAAGCAGGCTCTGAAGAAGTATGACATGCTCTGCCTGCTCTTCCAT
GAGCCTGTGAGTTCTGACAAGGTCTCCCAAAAGCAGTTCCAGATGACAGAGATGGTCCTGGAGCTGGCAGCTCAGGTCCTGGAGCCCAG
AAGCATTGGTTTTGGGATGGTGGACTCCAAGAAGGATGCCAAACTTGCCAAAAAATTAGGCTTGCTTGAAGAGGGAAGTCTCTATGTCT
TTAAGGATGAGCGGTTGATCGAATTTGATGGGGAACTGGCCACAGATGTCTTGGTGGAATTCCTCTTGGATTTGCTAGAAGACCCCGTG
GAGGTCATAAACAGCAAGCTGGAGCTTCAGGCCTTTGACCAGATCGATGATGAAATCAAACTCATCGGCTACTTTAAAGGAGAAGACTC
TGAACATTTCAAGGCATTTGAAGAAGCTGCTGAACAATTCCAGCCGTACATCAAGTTCTTTGCTACTTTTGACAAAGGGGTTGCCAAGA
AGCTAGGTCTGAAGATGAATGAAGTGGACTTCTATGAACCATTTATGGATGAGCCTGTTCACATCCCTGATAAGCCTTACACAGAAGAG
GAGCTGGTTGAATTTGTGAGAGAGCACAGAAGGGCCACCTTGAGGAAGCTGCGCCCAGAGGACATGTTTGAGACGTGGGAGGATGACAT
GGAGGGTATCCACATCGTAGCCTTCGCTGAAGAAGACGACCCAGATGGTTTTGAGTTCCTGGAAATTCTGAAGCAGGTTGCCAGGGACA
ACACTGATAATCCTGACCTGAGCATTGTCTGGATTGACCCTGATGACTTTCCTCTGCTGATCACTTACTGGGAGAAGACCTTCAAGATC
GACTTGTTCAGACCACAGATCGGGGTGGTGAATGTCACAGATGCTGACAGCATCTGGATGGACATCAGAGATGATGATGATCTGCCCAC
AGCTGAGGAGCTGGAAGACTGGATAGAAGATGTGCTTTCTGGGAAGATAAATACTGAAGATGATGATGATGACGACGATGACGACGATG
ACGATGACGATGATGACGATGATGACGATGATGATGACGATGATGATGATGATGACGATGATGACGATGACTAACTGTGACTCTGTGCA
GTTTGACTTGTGGGGCCCGAGAGCCCTCCCCCTGCGGCAGGTCCCTCCATTGGAAGGCCTGTGTTTGAGCAAGCACTGCTGCTCCTATT
TGCCCCCTGCCCTGCTCCCCCTGCCCCTGCCCTTGCCGGGACCCCCTGGCCTGATTCTCAGCGGTGCTGAGCCACCGGGACTGCCCCTG
CGGTGGGCAGTGCCTGCAGGCTCCCAGCACCGCTGCAAATCATGCCTCCTTAGTAAGGACAATAGGAATCTGCAGGGCATCTGCCCCCA
GTGTCCCAGGGCAGGAGAAAAGTGCCTGCCTGCTTGCTGCCAGAGGGACGCAGGGAGCCAGTTCCTGTTTTCCATGTTCGTCAGCCCAG
CACTTAACATCCAAAGACCAAATCTCCCTTCAAGACATTGAGAAGGACACTGTAAAGTTTAGTCCTTTTATTAAACACAATGCCCTTTG
CAGATACTCTGCCAAGACCACTCAATCCTGCAAGTCCTGCTGTGGGAGTCCCTAGCTAACGCTAGGAGCAGCAAAATGCTTTGGGAGGG
GAGGAAGGTTTTAGGGATGCTATTAAAGTTTTCAGTGATTCCACATAACAACAGGATATTCTTGTCTGGCAGTGTGATGCAAAACCAGG
CGCCGTGCCCTCTGTCTGAACCTCTTTTCTTGAAAGCTCGGGCCATGGCCGGGGAAGTGCAGATGGTCTGACAGGAGGCATAAAAGCTC
GCAAGTTCCTGAACTGCTGGAAAGGCTGATCCAGGTCTAAGCCCAATCTGAGTCCAAACTCTTGAGTTTGAAGACCATGGCACTGAGTA
AAGCCAATGGAAACATCTGTAGGGGCCCACCCTGAAGCTCTCCTATTCCCCATGACCTTCTGGCTTTAGCTGACTCTTCATTCAAAGCC
CACGTAGCATCTATTCCCATGACCAAGATGTTGGGAGGGAACACACCCATCCAACTATATTTGATATTGGATGAGGTATGTTGGACAAT
GAGCGGTGAATGGATTCAAGTTGTTTGGCTTTAAGTATTTAGTTGCACAGTTACTCCCTGCTTAGAGACTCAAATGTCCCATAGTAGCC
TGTGAGAAGCATCTTTTGACTGCAGGAGCTCAGGGTTTCAGTTTGCAGTCAAGGGGGTTGCTCACAAAGCAAAGGGTCTGCAACACAGT
CCTGGTGAGTGCAGCACCATCCACATGCAGGTCACTGTGGCAGCATTAAATCTCCCTGCTTTCCCATGCCAGCAGATTTCAATTGTCTT
AGCTTATTAGGTTTGACAATAGTACAATGAACAGAGAGTTCGGAGATAGTTTTATCTCTATATACACATTTATAAGGATGTTCTCTGGT
TGTTCAGGCTTGTTAGCATAGGTAGGGTATGCATCTCCTTTCTTGCTACAGATGGAAAACATTGTCCTGACTGTTCTTTTACTGGCTTA
CGTCAGAGGTAAGTGATATTAGAGGAAATAACAGTGTGAGGGACAGGAGAATCAGAGCTCATTACACAAGTGTGAATCAGCCTGATGAG
AAACAAGGTTCACACACTATATATTTCCTTTATCAGATGAACCACAAAGCAAGATTTTATAGATTTTTTTTTTTGCTGCCTGTTGATCA
GTCTCTTTGACTCTCTCTCTACATTAGCTGAATAGAAATTTGAAACTGCTGGTATAAAAAAGCT

>CASQ2_Xenopipo_atronitens_CDS
ATGAAGGCAACTTGCTGGATCCTGGCAGGTTTTTACCTGCTTTTCTGCTGCAAGGCAGAAGAGGGACTGAACTTCCCCACTTACGATGG
GAAAGACCGAGTGATCGACCTGAATGAGAAGAACTACAAGCAGGCTCTGAAGAAGTATGACATGCTCTGCCTGCTCTTCCATGAGCCTG
TGAGTTCTGACAAGGTCTCCCAAAAGCAGTTCCAGATGACAGAGATGGTCCTGGAGCTGGCAGCTCAGGTCCTGGAGCCCAGAAGCATT
GGTTTTGGGATGGTGGACTCCAAGAAGGATGCCAAACTTGCCAAAAAATTAGGCTTGCTTGAAGAGGGAAGTCTCTATGTCTTTAAGGA
TGAGCGGTTGATCGAATTTGATGGGGAACTGGCCACAGATGTCTTGGTGGAATTCCTCTTGGATTTGCTAGAAGACCCCGTGGAGGTCA
TAAACAGCAAGCTGGAGCTTCAGGCCTTTGACCAGATCGATGATGAAATCAAACTCATCGGCTACTTTAAAGGAGAAGACTCTGAACAT
TTCAAGGCATTTGAAGAAGCTGCTGAACAATTCCAGCCGTACATCAAGTTCTTTGCTACTTTTGACAAAGGGGTTGCCAAGAAGCTAGG
TCTGAAGATGAATGAAGTGGACTTCTATGAACCATTTATGGATGAGCCTGTTCACATCCCTGATAAGCCTTACACAGAAGAGGAGCTGG
TTGAATTTGTGAGAGAGCACAGAAGGGCCACCTTGAGGAAGCTGCGCCCAGAGGACATGTTTGAGACGTGGGAGGATGACATGGAGGGT
ATCCACATCGTAGCCTTCGCTGAAGAAGACGACCCAGATGGTTTTGAGTTCCTGGAAATTCTGAAGCAGGTTGCCAGGGACAACACTGA
TAATCCTGACCTGAGCATTGTCTGGATTGACCCTGATGACTTTCCTCTGCTGATCACTTACTGGGAGAAGACCTTCAAGATCGACTTGT
TCAGACCACAGATCGGGGTGGTGAATGTCACAGATGCTGACAGCATCTGGATGGACATCAGAGATGATGATGATCTGCCCACAGCTGAG
GAGCTGGAAGACTGGATAGAAGATGTGCTTTCTGGGAAGATAAATACTGAAGATGATGATGATGACGACGATGACGACGATGACGATGA
CGATGATGACGATGATGACGATGATGATGACGATGATGATGATGATGACGATGATGACGATGACTAA

>CASQ2_Xenopipo_atronitens_protein_partial
MKATCWILAGFYLLFCCKAEEGLNFPTYDGKDRVIDLNEKNYKQALKKYDMLCLLFHEPVSSDKVSQKQFQMTEMVLELAAQVLEPRSI
GFGMVDSKKDAKLAKKLGLLEEGSLYVFKDERLIEFDGELATDVLVEFLLDLLEDPVEVINSKLELQAFDQIDDEIKLIGYFKGEDSEH
FKAFEEAAEQFQPYIKFFATFDKGVAKKLGLKMNEVDFYEPFMDEPVHIPDKPYTEEELVEFVREHRRATLRKLRPEDMFETWEDDMEG
IHIVAFAEEDDPDGFEFLEILKQVARDNTDNPDLSIVWIDPDDFPLLITYWEKTFKIDLFRPQIGVVNVTDADSIWMDIRDDDDLPTAE
ELEDWIEDVLSGKINTEDDDDDDDDDDDDDDDDDDDDDDDDDDDDDDDDDDD*

9 Aves: Passeriformes: Passeri

9.1 *Aegithalos concinnus* (black-throated bushtit)

From: Trinity *de novo* assembly of SRR7244720. CASQ1 from TRINITY_DN1058_c0_g1_i3 and CASQ2 from complement(TRINITY_DN2780_c0_g1_i1).

```
>CASQ1_Aegithalos_concinnus_denovo_transcript_assembly
TGTCCGTGTGTCCGTGTGTCCCCAAAGCCTGTCCGTGTGTCCGTGTGTCCGTGTGTCCCCAAGGTCTGTGTGTCCCCAGGGTGTCTCTG
TCCTCTGCCCCTCGCTGGGCCCTGTCCCCGCCTGTCCCTCTGTCCCCGTCCGTGTGTCCGTGTGTCCATCCCCGTGTCCGTGTGTCCCC
ATCCCTGTCCCCGTGTCCATCCCCGTGTCCGTGTGTCCATCCCCGTGTCCCCGTGTCCGTGTGTCCATCCCCGTGTCCGTGTGTCCATC
CCCGTGTCCCCGTGTCCGTGTGTCCATCCCCGTGTCCCCCTGTCCCTGTCCCCGTGTCCCCCTGTCCCCCTTTCCCCGTGTCCCCATCC
CTGTCCCCGTGTCCATCCCCGTGTCCCTGTCCCGTGTCCCCATGTCCCCGCCGTGTCCGTGTGTCCATCCCCGTGTCCCCCTGTCCCCC
CCGTGTCCCTGGGGGTCTCTCCTGGCGCTGCTGGCGCTGAGCTCGGGGCTCTCGGGGGGCTCCGGGGGGCGATGCCCGCTCCGGGCGGG
GACCCCCGGTGACCTGCGGGGACACTGCCCGGTGCTGGCGCTGCCCCGGCCCGGGGACAGGGGACAGGGGGACGGGGGACAGCGGCCCG
AGGAGCTGGAGGCCCCGGGCCCCCCCGTGCAGGTGGTGGCAGCAGGACGGGAGCTCGAGGCCTTTGGGGACATCGAGGAGGAGCCCAAA
GTCATCGGGTACTTCGAGGGACGGGACTCGGAGCCCTTCCAGGCCTTCTCTGCCGCCGCCCGGCGCTTCCACCCCTCCCTCCTCTTCTT
CGCCACCTTCGACCCCCAGGCGGCGCAGGCGCTGGGGCTGGGGCTGCACCAGCTCCTTCTCTTCGAGCCCTTCCTGGAGCAGCCCCGGA
GCTTCCGGGGGCACCCCGGGGACCCCCCCGGCATCGAGGCCTTCCTGGAGGGGCACCGGAGGGCCACTCTGCGGAAACTCAAAGCTCAG
AGCGTGGCCGAGAGCTGGGAGGTCACCTCGGACGGGATCCACATCGTGGCCTTCGCCGAGGGGGATGATCCCGACGGGTTCGAGTTCCT
GGACACCCTGAGGGAGGTGGCGCGGGACAGGAGGGACGATCCCGACTTCAGCATCCTCTGGATCGACCCCGACGATTTCCCGCTGCTCG
TCCCCTCCTGGGAGGACACCTTTGACATCGACCTGTCCCGGCCCCAGCTGGGCGTGCTCAATGGCACCGACCCTGCCCGCAGCGTGTGG
CTGGACATGGAGGATGAGGAGGATCTGCCGGGGCCTGAGGAGGTCCTGGAGTGGCTCGAGGAGGTTCTGGAGGGGGACACTGGGGACAG
CGATGGCGATGAGGATGACGGTGGAGATGGAGACGATGAAGACGATGATGAAGATGATGAAGATGAAGATGATGATGACAAAGTTGATG
ACAATGACAACGATGCTGACTGACAGCAGAGATGACGATGATGATGATGAAGATGGCAATGACGATGACAATGACTGATGATGACAAGG
ACGATGATGACAATGATGATGACAGCAACAATGACATTGGTGATGATGAAGATGATGATGACAATGACCATGACAATGATGATGAAAAT
AATGGTGATGGTGAATGACAATGATGATGACATTGACAATGACGATGATGATGATGGTGATGATGTTGACTGGGCCATAAAATCCCCAA
AAGCCCAGGGAGTCCCCAGGGGAATGGGGATCCCTGGGGATGGAAAAAAAAAAAAGGA
```

```
>CASQ1_Aegithalos_concinnus_denovo_CDS
ATGTCCCCGCCGTGTCCGTGTGTCCATCCCCGTGTCCCCCTGTCCCCCCCGTGTCCCTGGGGGTCTCTCCTGGCGCTGCTGGCGCTGAG
CTCGGGGCTCTCGGGGGGCTCCGGGGGGCGATGCCCGCTCCGGGCGGGGACCCCCGGTGACCTGCGGGGACACTGCCCGGTGCTGGCGC
TGCCCCGGCCCGGGGACAGGGGACAGGGGGACGGGGGACAGCGGCCCGAGGAGCTGGAGGCCCCGGGCCCCCCCGTGCAGGTGGTGGCA
GCAGGACGGGAGCTCGAGGCCTTTGGGGACATCGAGGAGGAGCCCAAAGTCATCGGGTACTTCGAGGGACGGGACTCGGAGCCCTTCCA
GGCCTTCTCTGCCGCCGCCCGGCGCTTCCACCCCTCCCTCCTCTTCTTCGCCACCTTCGACCCCCAGGCGGCGCAGGCGCTGGGGCTGG
GGCTGCACCAGCTCCTTCTCTTCGAGCCCTTCCTGGAGCAGCCCCGGAGCTTCCGGGGGCACCCCGGGGACCCCCCCGGCATCGAGGCC
TTCCTGGAGGGGCACCGGAGGGCCACTCTGCGGAAACTCAAAGCTCAGAGCGTGGCCGAGAGCTGGGAGGTCACCTCGGACGGGATCCA
CATCGTGGCCTTCGCCGAGGGGGATGATCCCGACGGGTTCGAGTTCCTGGACACCCTGAGGGAGGTGGCGCGGGACAGGAGGGACGATC
CCGACTTCAGCATCCTCTGGATCGACCCCGACGATTTCCCGCTGCTCGTCCCCTCCTGGGAGGACACCTTTGACATCGACCTGTCCCGG
CCCCAGCTGGGCGTGCTCAATGGCACCGACCCTGCCCGCAGCGTGTGGCTGGACATGGAGGATGAGGAGGATCTGCCGGGGCCTGAGGA
GGTCCTGGAGTGGCTCGAGGAGGTTCTGGAGGGGGACACTGGGGACAGCGATGGCGATGAGGATGACGGTGGAGATGGAGACGATGAAG
ACGATGATGAAGATGATGAAGATGAAGATGATGATGACAAAGTTGATGACAATGACAACGATGCTGACTGA
```

```
>CASQ1_Aegithalos_concinnus_denovo_protein
MSPPCPCVHPRVPLSPPCPWGSLLALLALSSGLSGGSGGRCPLRAGTPGDLRGHCPVLALPRPGDRGQGDGGQRPEELEAPGPPVQVVA
AGRELEAFGDIEEEPKVIGYFEGRDSEPFQAFSAAARRFHPSLLFFATFDPQAAQALGLGLHQLLLFEPFLEQPRSFRGHPGDPPGIEA
FLEGHRRATLRKLKAQSVAESWEVTSDGIHIVAFAEGDDPDGFEFLDTLREVARDRRDDPDFSILWIDPDDFPLLVPSWEDTFDIDLSR
PQLGVLNGTDPARSVWLDMEDEEDLPGPEEVLEWLEEVLEGDTGDSDGDEDDGGDGDDEDDDEDDEDEDDDDKVDDNDNDAD*
```

```
>CASQ2_Aegithalos_concinnus_denovo_transcript_assembly
ACGGGAGCTGGACACAGCTATGTAAGGTATCCAGGGCTTGACAGCTTCCCCCGTCTGTCTCTCTCTTTGTTGTGAGTACAGGACAGCAA
AGTTTGCTGCTCCCCACCAGCCTTGCTGTGTTTGGGCTCAACACTAGGAAAGCAGCAAAGAGAAACTTAGAGGAACTAAGAGAAACTTA
GTTCAAACTTCACCTGGAGCCTTTGGTCTCCCTCTCTTCTCAAATTAAGAAAGAGGGGGACATTTACCCACCCTTTGTTGCACTCCAAG
GGTGCCCCTATGAAGGCAATTTGCTGGATCCTGGCAGGTTTTTACCTGCTTTTCTGCTGTAAGGCAGAAGAGGGACTGAACTTCCCTAC
TTATGATGGGAAAGACCGAGTGATCGACCTGAATGAGAAGAATTACAAGCAGGCACTGAAGAAGTATGACATGCTCTGCCTGCTCTTCC
ATGAGCCTGTGAGCTCTGACAAGGTCTCCCAGAAGCAGTTCCAGATGACAGAGATGGTCCTGGAGCTGGCAGCTCAGGTCCTGGAGCCC
AGGAGCATTGGCTTTGGAATGGTGGACTCCAAGAAGGATGCCAAGCTTGCCAAAAAGTTAGGCTTGCTTGAAGAGGGAAGTCTCTACGT
CTTTAAGGATGAGCGGTTGGTTGAATTTGATGGAGAATTGTCTGCAGATGTCTTGGTGGAATTCCTCTTGGATTTGCTAGAAGACCCCG
TGGAGGTCATAAACAGCAAGCTGGAGCTGCAGGCCTTTGACCAGATCGACGACGAAATCAAACTCATTGGCTACTTCAAGGGAGAAGAC
TCAGAACATTTCAAGGCATTTGAAGAAGCTGCTGAACAATTCCAACCCTATGTCAAGTTCTTTGCCACCTTTGACAAAGGGGTTGCCAA
GAAGCTAGGCCTGAAGATGAATGAGGTGGACTTCTATGAACCCTTTATGGATGAGCCTGTTCACATCCCTGATAAGCCTTATTCCGAAG
AGGAGCTGGTTGATTTTGTGAGGGAGCACAGAAGGGCCACCTTGAGGAAGCTGCGCCCAGAGGACATGTTTGAGACATGGGAGGATGAC
```

ATGGAGGGTATCCATATTGTAGCCTTTGCTGAAGAAGATGACCCAGATGGTTTTGAGTTCCTGGAAATCCTGAAGCAGGTTGCCAGGGA
CAACACTGATAATCCTGACCTGAGCATTGTCTGGATTGACCCTGACGACTTTCCTCTTCTCATCACTTACTGGGAGAAGACCTTCAAGA
TCGACCTGTTCAGACCACAGATCGGGGTGGTGAACGTCACGGACGCTGACAGCATCTGGATGGACATCAGAGATGATGATGACCTGCCC
ACAGCAGAGGAGCTGGAGGACTGGATAGAGGATGTGCTTTCTGGGAAGATCAATACCGAGGATGATGACGACGATGAAGATGACGATGA
CGACGACGATGATGACGATGATGACGACGACGATGACGATGATGACGACGATGATGACGACGATGATGACTAACTGTGACTTGGTGCAG
TTTGACTTGTGGGGCCCGAGAGCCCTCGCCCTGTGGCAGGTCCCTCCATTGGAAGACCTATGTTTGAGCATCAGCTGCTCCTCTTTCCC
CTGGCCCCTCTCCCCCTGCCCTCGCCCTTGCCGGGACCCCCTTGCCTGATTCCCAGCGGTGCTGAGCCACCAGGACTGCCCCTGTGGAG
GGCAGCACCTGCAGGCACCCAGCACCGCTGCCAATCATGCCTCCTTAGTAAGGACAGTAGGAATCTGCAGGGAATCTGCCCTGAGTGTC
CCAGGGCAGGAAAAAAGTGCCTGCCTGCTTGCTGCCAGAGGGATGCAGGGAGCCAGTTTGTATTTTCTGTGTTCATCAGCCCAGCACTT
AACATCCAAAGACCAAATCTCACTTCAAGACATAGGGAAAAGCCTAACTGTAAAGTTTAGCCCTTTTATTAAACACAATGCCCTTTGCA
GCTACTCTGCCCAGACCACTCAATCCTGCAAGTTCTGCTGCGGGAGTCTGTAGCTACTCCCTAACATGCTAAGAGCCAGAAAAATGCTT
TGGGAGGGGAGGGCAATTTTAGGGACGCTATTAAAGTTTTCAATGATTCCACATTAACAACAGGATATTTGTGTCTGGCAGTGCAATGC
AAAACCAAACACTGGGTCCTCTGTCTGAACCTCTTTCTTCTTGAAAGCTTGGGCCACAGCTGGAGCCACTGCAGTTGGCCTGATATGAG
ACACAAAGCTCATGAGTTCCTGAACTGCTGGATGGGCTGGTCCAGCTCTGAGCCCAATGTGAGTTAAAATGACTGAGTCTGAAGATCAT
GGACTGAGCCAAGTCAATGGAAACACCTATAGGGGCCCTCCCTGAAGCATTCCTATTCCCCATGACCTTCCAACTTTTGCTGATTCTTC
ACCCAAAGCCCGTGTAGCATCTTTTCCCATGATCAAGATATTGGGAGGGAACACATCTACCTATCTAAACAGATAGTGGATGAGGTATG
TTGGGGTGTGAGCAGCATAGGGATTCAAGCAGTTTGGCTTTTGAGTATTTAGTTGCACAGTTACTCACTCCCTGCTTAGAGACTCAGAT
ATCCCATGTGAGAAACCCCTTTGGCTGCAGGCTGAGAAGGATCTCAGGGTACCAGTTTGCAGTCAAGGGGTTCACTCACAAAGCCAAGG
GTCTGTACCATGGTCCTGGCGAGTGCAGCACCATCCATGTGGAGGTCATTGTGGCAGCATTAGGTCTCCCTGCTTTCCCATGCTGGCAG
ATTTCAATTGTCTTAGCTTCTTATGTTTGGCAATAGTGCAATGAATGGAGAGTTCAGAGATTTTTTTCTTTATAAATACATTTATAAAG
ATGTTCTCCAGTTGTTCAGAGTTGCTAGCATAGGTAGTGTATGTGTACACATCTCTTCTTTTGCTACAAACAAAAAACATTGTTCCGAA
CATCTTTATACTGGCTCATGACAGAGGTACGTGATGTGAGGTGAAATAACCATGTGAGGGACAGAGGAATCAGAGTTACACAAATGTGA
ATCAGCCTGATGAGAAACAAGGTTTACATACTATATATTTCCTTTATCAGATGAACTGCAAAGCAAGATTTTATAGATTTTTTTTTCC
AGCCTGTTGAACAGTTTCTTTGACTCTCTCTCTACATTAGCTGAAATAGAAATTTGAAACTATTGGTATAAAAAAGCTGAAGCATTGAT
GTGTTCCCATGTGTGCTATATCCCCTTGGTCGAAAATACCAGTATGAACAACACATAGCTGGATTCCTCTCTGTCTCTATGCACTTGAA
TGAATACTGTGCTAACATGGTAGATGACTGTAGGCCAAGTTAGCTTCTTGTTGAGCATCAAACCCTCAACGTGCTTTTCAGGTACAGCC
TATTAGAGGAATAGACAAG

>CASQ2_Aegithalos_concinnus_denovo_CDS
ATGAAGGCAATTTGCTGGATCCTGGCAGGTTTTTACCTGCTTTTCTGCTGTAAGGCAGAAGAGGGACTGAACTTCCCTACTTATGATGG
GAAAGACCGAGTGATCGACCTGAATGAGAAGAATTACAAGCAGGCACTGAAGAAGTATGACATGCTCTGCCTGCTCTTCCATGAGCCTG
TGAGCTCTGACAAGGTCTCCCAGAAGCAGTTCCAGATGACAGAGATGGTCCTGGAGCTGGCAGCTCAGGTCCTGGAGCCCAGGAGCATT
GGCTTTGGAATGGTGGACTCCAAGAAGGATGCCAAGCTTGCCAAAAAGTTAGGCTTGCTTGAAGAGGGAAGTCTCTACGTCTTTAAGGA
TGAGCGGTTGGTTGAATTTGATGGAGAATTGTCTGCAGATGTCTTGGTGGAATTCCTCTTGGATTTGCTAGAAGACCCCGTGGAGGTCA
TAAACAGCAAGCTGGAGCTGCAGGCCTTTGACCAGATCGACGACGAAATCAAACTCATTGGCTACTTCAAGGGAGAAGACTCAGAACAT
TTCAAGGCATTTGAAGAAGCTGCTGAACAATTCCAACCCTATGTCAAGTTCTTTGCCACCTTTGACAAAGGGGTTGCCAAGAAGCTAGG
CCTGAAGATGAATGAGGTGGACTTCTATGAACCCTTTATGGATGAGCCTGTTCACATCCCTGATAAGCCTTATTCCGAAGAGGAGCTGG
TTGATTTTGTGAGGGAGCACAGAAGGGCCACCTTGAGGAAGCTGCGCCCAGAGGACATGTTTGAGACATGGGAGGATGACATGGAGGGT
ATCCATATTGTAGCCTTTGCTGAAGAAGATGACCCAGATGGTTTTGAGTTCCTGGAAATCCTGAAGCAGGTTGCCAGGGACAACACTGA
TAATCCTGACCTGAGCATTGTCTGGATTGACCCTGACGACTTTCCTCTTCTCATCACTTACTGGGAGAAGACCTTCAAGATCGACCTGT
TCAGACCACAGATCGGGGTGGTGAACGTCACGGACGCTGACAGCATCTGGATGGACATCAGAGATGATGATGACCTGCCCACAGCAGAG
GAGCTGGAGGACTGGATAGAGGATGTGCTTTCTGGGAAGATCAATACCGAGGATGATGACGACGATGAAGATGACGATGACGACGACGA
TGATGACGATGATGACGACGACGATGACGATGATGACGACGATGATGACGACGATGATGACTAA

>CASQ2_Aegithalos_concinnus_denovo_protein
MKAICWILAGFYLLFCCKAEEGLNFPTYDGKDRVIDLNEKNYKQALKKYDMLCLLFHEPVSSDKVSQKQFQMTEMVLELAAQVLEPRSI
GFGMVDSKKDAKLAKKLGLLEEGSLYVFKDERLVEFDGELSADVLVEFLLDLLEDPVEVINSKLELQAFDQIDDEIKLIGYFKGEDSEH
FKAFEEAAEQFQPYVKFFATFDKGVAKKLGLKMNEVDFYEPFMDEPVHIPDKPYSEEELVDFVREHRRATLRKLRPEDMFETWEDDMEG
IHIVAFAEEDDPDGFEFLEILKQVARDNTDNPDLSIVWIDPDDFPLLITYWEKTFKIDLFRPQIGVVNVTDADSIWMDIRDDDDLPTAE
ELEDWIEDVLSGKINTEDDDDDEDDDDDDDDDDDDDDDDDDDDDDDDDD*

9.2 *Aegithalos iouschistos* (black-browed tit)

From: Trinity *de novo* assembly of SRR7244720. CASQ2 as TRINITY_DN2488_c0_g1_i1

>CASQ2_Aegithalos_iouschistos_denovo_transcript_assembly
AAGGACTGACATCTTCCACTCCCTCCCTCTTGCCCACTCCCTCTCAGATGATGCAGAGTGCCAGCAAGTCCCTCAATGGTGTCATATCT
GTCCTTTTTATTCATTGCAGGGTTTATTTTTAGCCTGAAACAACTGCTTCCTAAAAATGGAGTTCCTAATGACACGGGAGCTGGACACA
GCTATGTAAGGTATCCAGGGCTTGACAGCTTCCCCCGTCTGTCTCTCTCTTTGTTGTGAGTACAGGACAGCAAAGTTTGCTGCTCCCCA
CCAGCCTCGCTGTGTTTGGGCTCAACACTAGGAAAGCAGCAAAGAGAAACTTAGAGGAACTAAGAGAAACTTAGTTCAAACTGCACCTG
GAGCCTTTGGTCTCCCTCTCTTCTCAAATTAAGAAAGAGGGGGACATTTACCCACCCTTTGCTGCACTCCAAGGGTGCCCCTATGAAGG
CAATCTGCTGGATCCTGGCAGGTTTTTACCTGCTTTTCTGCTGTAAGGCAGAAGAGGGACTGAACTTCCCTACTTATGATGGGAAAGAC

CGAGTGATCGACCTGAATGAGAAGAATTACAAGCAGGCACTGAAGAAGTATGACATGCTCTGCCTGCTCTTCCATGAGCCTGTGAGCTC
TGACAAGGTCTCCCAGAAGCAGTTCCAGATGACAGAGATGGTCCTGGAGCTGGCAGCTCAGGTCCTGGAGCCCAGGAGCATTGGCTTTG
GAATGGTGGACTCCAAGAAGGATGCCAAGCTTGCCAAAAAGTTAGGCTTGCTTGAAGAGGGAAGTCTCTATGTCTTTAAGGACGAGCGG
TTGGTTGAATTTGATGGAGAATTGTCTGCAGATGTCTTAGTGGAATTCCTCTTGGATTTGCTAGAAGACCCCGTGGAGGTCATAAACAG
CAAGCTGGAGCTGCAGGCCTTTGACCAGATCGACGACGAAATCAAACTCATTGGCTACTTCAAGGGAGAAGACTCAGAACATTTCAAGG
CATTTGAAGAAGCTGCTGAACAATTCCAACCCTACGTCAAGTTCTTTGCCACCTTTGACAAAGGGGTTGCCAAGAAGCTAGGCCTGAAG
ATGAATGAGGTGGACTTCTATGAACCCTTTATGGATGAGCCTGTTCACATCCCTGATAAGCCTTATTCCGAGGAGGAGCTGGTTGATTT
TGTGAGAGAGCACAGAAGGGCCACCTTGAGGAAGCTGCGCCCAGAGGACATGTTTGAGACATGGGAGGATGACATGGAGGGTATCCATA
TTGTAGCCTTTGCTGAAGAAGATGACCCAGATGGTTTTGAGTTCCTGGAAATCCTGAAGCAGGTTGCCAGGGACAACACTGATAATCCT
GACCTGAGCATTGTCTGGATTGACCCTGACGACTTTCCTCTTCTCATCACTTACTGGGAGAAGACCTTCAAGATCGACCTGTTCAGACC
ACAGATCGGGGTGGTGAACGTCACGGACGCTGACAGCATCTGGATGGACATCAGAGATGATGATGACCTGCCCACAGCAGAGGAGCTGG
AGGACTGGATAGAGGATGTGCTTTCTGGGAAGATCAATACCGAGGATGATGATGACGATGAAGATGACGATGACGACGATGATGATGAT
GATGATGACGACGACGATGACGATGATGACGACGATGATGACGATGATGATGACTAACTGTGACTTGGTGCAGTTTGACTTGTGGGGCC
TGAGAGCCCTCGCCCTGTGGCAGGTCCCTCCATTGGAAGACCTATGTTTGAGCATCAGCTGCTCCTCTTTCCCCTGGCCCCTCTCCCCC
TGCCCTTGCCCTTGCCGGGACCCCCTTGCCTGATTCCCAGCGGTGCTGAGCCACCAGGACTGCCCCTGTGGAGGGCAGCACCTGCAGGC
ACCCAGCACCGCTGCCAATCATGCCTCCTTAGTAAGGACAGTAGGAATCTGCAGGGAATCTGCCCTGAGTGTCCCAGGGCAGGAAAAAA
GTGCCTGCCTGCTTGCTGCCAGAGGGATGCAGGGAGCCAGTTTGTATTTTCTGTGTTCATCAGCCCAGCACTTAACATCCAAAGACCAA
ATCTCACTTCAAGACATAGGGAAAAGCCTAACTGTAAAGTTTAGCCCTTTTATTAAACACAATGCCCTTTGCAGCTACTCTGCCCAGAC
CACTCAATCCTGCGAGTTCTGCTGCGGGAGTCTGTAGCTACTCCCTAACATGCTAAGAGCCAGAAAAATGCTTTGGGAGGGGAGGGCAA
TTTTAGGGACGCTATTAAAGTTTTCAATGATTCCACATTAACAACAGGATATTTGTGTCTGGCAGTGCAATGCAAAACCAAACACCGGG
TCCTCTGTCTGAACCTCTTTCTTCTTGAAAGCTTGGGCCACAGCTGGAGTCACTGCAGTTGGCCTGATATGAGACACAAAGCTCATGAG
TTCCTGAACTGCTGGATGGGCTGGTCCAGCTCTGAGCCCAATGTGAGTTCAAACGACTGAGTCTGAAGATCATGGACTGATGCAAGTCA
ATGGAAACACGTATAGGGGCCCTCCCTGAAGCATTCCTATTCCCCATGACCTTCCAACTTTTGCTGACGCTTCACCCAAAGCCCGTGTA
GCATCTTTTCCCATGATCAAGATGTTGGGAGGGAACACATCTACCTATCCAAACAGATAGTGGATGAGGTGTGTTGGGGTGTGAGCAGC
AGAGGGATTCAAGCAGTTTGGCTTTTGAGTATTTAGTTGCACAGTTACTCACTCCCTGCTTAGAGACTCAGATATCCCACGTGAGAAAC
CTCTTTGGCTGCAGGCTGAGAAGGACCTCAGGGTACCAGTTTGCAGTCAAGGGGTTCACTCACAAAGCCAAGGGTCTGTACCATGGTCC
TGGCGAGTGCAGCAGCATCCATGCGGAGGTCATTGTGGCAGCATTAGGTTTCCCTGCTTTCCCATGCTGGCAGATTTCAATTGTCTTAG
CTTCTTATGTTTGGCAATAGTGCAATGAATGGAGAGTTCAGAGATGGTTTTTTCTTTATAAATACATTTATAAAGATGTTCTCCAGTTG
TTCAGAGTTGCTAGCATAGGTAGTGTATGTGTACACATCTCTTCTTTTGCTACAAACAAAAAACATTGTTCCGAACATCTTTATACTGG
CTCATGACAGAGGTACGTGATGTGAGGTGAAATAACCATGTGAGGGACAGAGGAATCAGAGTTATACAAATGTGAATCAGCCTGATGAG
AAACAAGGTTTACATACTATATATTTCCTTTATCAGATGAACTGCAAAGCAAGATTTTATAGATCTTTTTTTTTTTTTCCAGCCTGTTG
AACAGTTTCTTTGACTCTCTCTCTACATTAGCTGAAATAGAAATTTGAAACTATTGGTATAAAAAAGCTGAAGTATTGATGTGTTCCCA
TGTGTGCCATATCCCTTTGGTCGAAAATACCAGTATGAACAACACATAGCTGGATTTCTCTCTGTCTCTATGCACTTGAATGAATACTG
TGCTAGCATGGTAGATGACTGTAGGCCAAGTTAGCTTCCTGTTGAGCATCAAACCCTCAACCTACTTTTCAGGTACAGCCTATTAGAGG
AATAGACAAGGCAGCAAAGCCCCCAGGTCAATCATTTGCTCTTCCGTGCTTCCCTGTGATTGCCTGGAAGATGAACCAAGTTTCTTCAG
GGAGAACAATCAACTGGTTGCTCTGGTGTGATGAACAACTTTAAAGTTTTGAAACTCAGAGTGCTCTCAGCATAGACGTCTCCTCCTCT
CTTCCCCTCACACCCTACTCTCTGCTGGCTGACAGGGTGCCCAGCACTCTCATTCTGCTTTTTCCTCAAGGGGTTTACTGGAGGACCCC
AGCTCAGAGAGGGAGCAAGCAGGACTCAAGGTCCCATTCACCCCACTGGTAAAGAGCA

>CASQ2_Aegithalos_iouschistos_denovo_CDS
ATGAAGGCAATCTGCTGGATCCTGGCAGGTTTTTACCTGCTTTTCTGCTGTAAGGCAGAAGAGGGACTGAACTTCCCTACTTATGATGG
GAAAGACCGAGTGATCGACCTGAATGAGAAGAATTACAAGCAGGCACTGAAGAAGTATGACATGCTCTGCCTGCTCTTCCATGAGCCTG
TGAGCTCTGACAAGGTCTCCCAGAAGCAGTTCCAGATGACAGAGATGGTCCTGGAGCTGGCAGCTCAGGTCCTGGAGCCCAGGAGCATT
GGCTTTGGAATGGTGGACTCCAAGAAGGATGCCAAGCTTGCCAAAAAGTTAGGCTTGCTTGAAGAGGGAAGTCTCTATGTCTTTAAGGA
CGAGCGGTTGGTTGAATTTGATGGAGAATTGTCTGCAGATGTCTTAGTGGAATTCCTCTTGGATTTGCTAGAAGACCCCGTGGAGGTCA
TAAACAGCAAGCTGGAGCTGCAGGCCTTTGACCAGATCGACGACGAAATCAAACTCATTGGCTACTTCAAGGGAGAAGACTCAGAACAT
TTCAAGGCATTTGAAGAAGCTGCTGAACAATTCCAACCCTACGTCAAGTTCTTTGCCACCTTTGACAAAGGGGTTGCCAAGAAGCTAGG
CCTGAAGATGAATGAGGTGGACTTCTATGAACCCTTTATGGATGAGCCTGTTCACATCCCTGATAAGCCTTATTCCGAGGAGGAGCTGG
TTGATTTTGTGAGAGAGCACAGAAGGGCCACCTTGAGGAAGCTGCGCCCAGAGGACATGTTTGAGACATGGGAGGATGACATGGAGGGT
ATCCATATTGTAGCCTTTGCTGAAGAAGATGACCCAGATGGTTTTGAGTTCCTGGAAATCCTGAAGCAGGTTGCCAGGGACAACACTGA
TAATCCTGACCTGAGCATTGTCTGGATTGACCCTGACGACTTTCCTCTTCTCATCACTTACTGGGAGAAGACCTTCAAGATCGACCTGT
TCAGACCACAGATCGGGGTGGTGAACGTCACGGACGCTGACAGCATCTGGATGGACATCAGAGATGATGATGACCTGCCCACAGCAGAG
GAGCTGGAGGACTGGATAGAGGATGTGCTTTCTGGGAAGATCAATACCGAGGATGATGATGACGATGAAGATGACGATGACGACGATGA
TGATGATGATGATGACGACGACGATGACGATGATGACGACGATGATGACGATGATGATGACTAA

>CASQ2_Aegithalos_iouschistos_denovo_protein
MKAICWILAGFYLLFCCKAEEGLNFPTYDGKDRVIDLNEKNYKQALKKYDMLCLLFHEPVSSDKVSQKQFQMTEMVLELAAQVLEPRSI
GFGMVDSKKDAKLAKKLGLLEEGSLYVFKDERLVEFDGELSADVLVEFLLDLLEDPVEVINSKLELQAFDQIDDEIKLIGYFKGEDSEH
FKAFEEAAEQFQPYVKFFATFDKGVAKKLGLKMNEVDFYEPFMDEPVHIPDKPYSEEELVDFVREHRRATLRKLRPEDMFETWEDDMEG
IHIVAFAEEDDPDGFEFLEILKQVARDNTDNPDLSIVWIDPDDFPLLITYWEKTFKIDLFRPQIGVVNVTDADSIWMDIRDDDDLPTAE
ELEDWIEDVLSGKINTEDDDDDEDDDDDDDDDDDDDDDDDDDDDDDDD*

9.3 *Daphoenositta chrysoptera* (varied sittella)

From: Trinity *de novo* assembly of SRR10852805. CASQ2 from TRINITY_DN395_c1_g3_i2.

```
>CASQ2_Daphoenositta_chrysoptera_transcript_assembly
GACAGCTTCTCCCATCTGTCTCTCTCTTTGTTGTGAGTACAGGACAGCAAAGTTTGCTGCTCCCCACCAGCCTTGCAGTGTTTGGGCTG
AACACTAGGAAAGCAGCAGAGAGAAACTTAGGGAAACTAAGAGAAACTTAGTTCAAACTTCACCTGGAGTCTTTGGTCTCCCTCTCTTC
TCAAATTAAGAAAGAGGGGGACATTTACCCACCCTTTTCTGCACTCCAAGGGTGCCCCTATGAAGGCAATTTGCTGGATCGTGGCAGGT
TTTTACCTGCTTTTCTGCTGCAAGGCAGAAGAGGGACTGAACTTCCCCACTTACGATGGGAAAGACCGAGTGATCGACCTGAACGAGAA
GAATTACAAGCAGGCACTGAAGAAGTATGACATGCTCTGCCTGCTCTTCCATGAGCCTGTGAGCTCTGACAAGGTCTCCCAGAAGCAGT
TCCAGATGACAGAGATGGTCCTAGAGCTGGCAGCTCAAGTCCTGGAACCCAGGAGCATTGGCTTTGGGATGGTGGACTCCAAGAAGGAT
GCCAAGCTTGCCAAAAAGTTAGGCTTGGTTGAAGAGGGAAGTCTCTATGTCTTTAAGGACGAGCGGTTGGTTGAATTTGATGGGGAACT
GTCCGCAGATGTCTTGGTGGAATTCCTCTTGGATTTGCTAGAAGACCCCGTGGAGGTCATAAACAGCAAGCTGGAGCTTCAGGCCTTTG
ACCAGATTGACGAAGAAATCAAACTCATTGGCTACTTCAAGGGAGAAGATTCAGAACATTTCAAGGCATTTGAAGAAGCCGCTGAACAA
TTCCAGCCGTACATCAAATTCTTTGCCACCTTTGACAAAGGGGTTGCCAAGAAGCTAGGCCTAAAGATGAACGAGGTGGACTTCTATGA
ACCCTTTATGGATGAGCCTGTTCACATCCCTGATAAACCTTACTCAGAAGAGGAGCTGGTTGATTTTGTGAGAGAGCACAGAAGGGCCA
CCTTGAGGAAGCTGCGCCCAGAGGACATGTTTGAGACGTGGGAGGATGACATGGAAGGTATCCATATTGTAGCCTTTGCTGAAGAAGAT
GACCCAGATGGTTTTGAGTTCCTGGAAATCCTGAAGCAGGTTGCCAGGGACAACACTGACAATCCCGACCTGAGCATTGTCTGGATTGA
CCCTGACGACTTTCCTCTGCTCATCACTTACTGGGAGAAGACCTTCAAGATTGACCTGTTCAGACCACAAATCGGGGTGGTGAACGTCA
CAGACGCTGACAGCGTCTGGATGGACATCAGAGATGACGATGACCTGCCCACAGCTGAGGAGCTGGAGGACTGGATAGAGGACGTGCTT
TCTGGGAAGATAAATACCGAAGATGATGATGACGATGAAGATGATGACGATGACGACGATGATGATGATGATGACGATGATGATGATGA
CGATGACGACGATGACGATGACGATGATGATGACTAACTGTGACTCTGTGCAGTTTGACTTGTGGGGCCCGAGAGCCCTCGCCCTGCGG
CAGGTCCCTCCATTGGAAGACCTATGTTTGAGCATCAGCAAGCACCGCTGCTCCTCTTTCCCCCTGCCCCGCTCCCCCTG
```

```
>CASQ2_Daphoenositta_chrysoptera_CDS
ATGAAGGCAATTTGCTGGATCGTGGCAGGTTTTTACCTGCTTTTCTGCTGCAAGGCAGAAGAGGGACTGAACTTCCCCACTTACGATGG
GAAAGACCGAGTGATCGACCTGAACGAGAAGAATTACAAGCAGGCACTGAAGAAGTATGACATGCTCTGCCTGCTCTTCCATGAGCCTG
TGAGCTCTGACAAGGTCTCCCAGAAGCAGTTCCAGATGACAGAGATGGTCCTAGAGCTGGCAGCTCAAGTCCTGGAACCCAGGAGCATT
GGCTTTGGGATGGTGGACTCCAAGAAGGATGCCAAGCTTGCCAAAAAGTTAGGCTTGGTTGAAGAGGGAAGTCTCTATGTCTTTAAGGA
CGAGCGGTTGGTTGAATTTGATGGGGAACTGTCCGCAGATGTCTTGGTGGAATTCCTCTTGGATTTGCTAGAAGACCCCGTGGAGGTCA
TAAACAGCAAGCTGGAGCTTCAGGCCTTTGACCAGATTGACGAAGAAATCAAACTCATTGGCTACTTCAAGGGAGAAGATTCAGAACAT
TTCAAGGCATTTGAAGAAGCCGCTGAACAATTCCAGCCGTACATCAAATTCTTTGCCACCTTTGACAAAGGGGTTGCCAAGAAGCTAGG
CCTAAAGATGAACGAGGTGGACTTCTATGAACCCTTTATGGATGAGCCTGTTCACATCCCTGATAAACCTTACTCAGAAGAGGAGCTGG
TTGATTTTGTGAGAGAGCACAGAAGGGCCACCTTGAGGAAGCTGCGCCCAGAGGACATGTTTGAGACGTGGGAGGATGACATGGAAGGT
ATCCATATTGTAGCCTTTGCTGAAGAAGATGACCCAGATGGTTTTGAGTTCCTGGAAATCCTGAAGCAGGTTGCCAGGGACAACACTGA
CAATCCCGACCTGAGCATTGTCTGGATTGACCCTGACGACTTTCCTCTGCTCATCACTTACTGGGAGAAGACCTTCAAGATTGACCTGT
TCAGACCACAAATCGGGGTGGTGAACGTCACAGACGCTGACAGCGTCTGGATGGACATCAGAGATGACGATGACCTGCCCACAGCTGAG
GAGCTGGAGGACTGGATAGAGGACGTGCTTTCTGGGAAGATAAATACCGAAGATGATGATGACGATGAAGATGATGACGATGACGACGA
TGATGATGATGATGACGATGATGATGATGACGATGACGACGATGACGATGACGATGATGATGACTAA
```

```
>CASQ2_Daphoenositta_chrysoptera_protein
MKAICWIVAGFYLLFCCKAEEGLNFPTYDGKDRVIDLNEKNYKQALKKYDMLCLLFHEPVSSDKVSQKQFQMTEMVLELAAQVLEPRSI
GFGMVDSKKDAKLAKKLGLVEEGSLYVFKDERLVEFDGELSADVLVEFLLDLLEDPVEVINSKLELQAFDQIDEEIKLIGYFKGEDSEH
FKAFEEAAEQFQPYIKFFATFDKGVAKKLGLKMNEVDFYEPFMDEPVHIPDKPYSEEELVDFVREHRRATLRKLRPEDMFETWEDDMEG
IHIVAFAEEDDPDGFEFLEILKQVARDNTDNPDLSIVWIDPDDFPLLITYWEKTFKIDLFRPQIGVVNVTDADSVWMDIRDDDDLPTAE
ELEDWIEDVLSGKINTEDDDDDEDDDDDDDDDDDDDDDDDDDDDDDDDDD*
```

9.4 *Lophophanes dichrous* (grey-crested tit)

From: Trinity *de novo* assembly of SRR10852805. CASQ1 from complement of TRINITY_DN2500_c0_g1_i1. CASQ2 from TRINITY_DN966_c1_g1.

```
>CASQ1_Lophophanes_dichrous_transcript_assembly
GTGTCCCCCTGTCCCTCTGTCCCGCTCTCCCGTGTCCCCCTGTCCCTCTGTCCCTCTGTCCCGTGTCCCCCTGTCCCTCTGTCCCTCTG
TCCCGTGTCCCCATGTCCCCGCTGTGGCCGTGCCGGTGGCTCCTGGCTCTGCTGGCTCTGTCCCCGGGGGTCCCGGGGGTCCCGGGGGT
CTCGGGGGGTCCCGGGGGGGCCGCTGAGCTGCGGCGGCGCCCCGGGGACACCGGACACCGGCCCGAGGAGCTGGAGGCGCTGGAGGCTC
TGGGGACCCCCGTGCAGGTGGTGGCAGCCGGGCAGGAGCTCCGGGCCTTTGGGGACATCGAGGAGGAGCCCAAAGTCATCGGGTACTTC
GAGGGACGGGACTCGGAGCCCTTCCAGGCCTTCTCCGCCACCGCCCGGCGCTTCCACCCCTCCCTCCTCTTCTTCGCCACCTTCGACCC
CCAGGCGGCGCAGGAGCTGCGGCTGGGGCTGAACCAGCTCCATCTCTTCGAGCCCTTCCTGGAGCAGCCCCGGAGCTTCCGGGGGGACC
CCGGGGACCCCCACGGGCTCGAGGCCTTCGTGGAGAGAAACAAGCGGGCCACTCTGCAGAAACTCAAAGCTCAGAGCACGTCCCAGAAC
TGGGAGGACCTTTGGGATGGGACCCACATCGTGGCCTTTGCCGAGGGGGATGATCCCGACGGGTTCGAGTTCCTGGAGATCCTGAAGGA
GGTGGCCCGGGGCAAGAGGGACAATCCCGACTTCAGCATCCTGTGGATCGATCCCGACGATTTCCCGATGCTCGTCCCCTCCTGGGAGG
ACACCTTTGACATCGACCTGTCCCGGCCCCAGCTCGGCGTGGTCAATGGCACTGACCATGCCGGCAGCGTGTGGCTGGACATGGAGGAT
```

GAGGAGGATCTGCCGGGACCTGAGGAGGTCCTGGAGTGGCTCGAGGAGGTTCTGGAGGGGGACACTGGGGACAGGGGTGGCGATGACGA
TGAAGAGGATGATGATGATGATGAAGATGAAGATGATGATGAAGATGATGACGATGACGATGAAGACGATGATGGTGACAAAGTTGATG
ATGATGATGATG

>CASQ1_Lophophanes_dichrous_CDS_partial
ATGTCCCCGCTGTGGCCGTGCCGGTGGCTCCTGGCTCTGCTGGCTCTGTCCCCGGGGGTCCCGGGGGTCCCGGGGGTCTCGGGGGGTCC
CGGGGGGGCCGCTGAGCTGCGGCGGCGCCCCGGGGACACCGGACACCGGCCCGAGGAGCTGGAGGCGCTGGAGGCTCTGGGGACCCCCG
TGCAGGTGGTGGCAGCCGGGCAGGAGCTCCGGGCCTTTGGGGACATCGAGGAGGAGCCCAAAGTCATCGGGTACTTCGAGGGACGGGAC
TCGGAGCCCTTCCAGGCCTTCTCCGCCACCGCCCGGCGCTTCCACCCCTCCCTCCTCTTCTTCGCCACCTTCGACCCCCAGGCGGCGCA
GGAGCTGCGGCTGGGGCTGAACCAGCTCCATCTCTTCGAGCCCTTCCTGGAGCAGCCCCGGAGCTTCCGGGGGGACCCCGGGGACCCCC
ACGGGCTCGAGGCCTTCGTGGAGAGAAACAAGCGGGCCACTCTGCAGAAACTCAAAGCTCAGAGCACGTCCCAGAACTGGGAGGACCTT
TGGGATGGGACCCACATCGTGGCCTTTGCCGAGGGGGATGATCCCGACGGGTTCGAGTTCCTGGAGATCCTGAAGGAGGTGGCCCGGGG
CAAGAGGGACAATCCCGACTTCAGCATCCTGTGGATCGATCCCGACGATTTCCCGATGCTCGTCCCCTCCTGGGAGGACACCTTTGACA
TCGACCTGTCCCGGCCCCAGCTCGGCGTGGTCAATGGCACTGACCATGCCGGCAGCGTGTGGCTGGACATGGAGGATGAGGAGGATCTG
CCGGGACCTGAGGAGGTCCTGGAGTGGCTCGAGGAGGTTCTGGAGGGGGACACTGGGGACAGGGGTGGCGATGACGATGAAGAGGATGA
TGATGATGATGAAGATGAAGATGATGATGAAGATGATGACGATGACGATGAAGACGATGATGGTGACAAAGTTGATGATGATGATGAT
G

>CASQ1_Lophophanes_dichrous_protein_partial
MSPLWPCRWLLALLALSPGVPGVPGVSGGPGGAAELRRRPGDTGHRPEELEALEALGTPVQVVAAGQELRAFGDIEEEPKVIGYFEGRD
SEPFQAFSATARRFHPSLLFFATFDPQAAQELRLGLNQLHLFEPFLEQPRSFRGDPGDPHGLEAFVERNKRATLQKLKAQSTSQNWEDL
WDGTHIVAFAEGDDPDGFEFLEILKEVARGKRDNPDFSILWIDPDDFPMLVPSWEDTFDIDLSRPQLGVVNGTDHAGSVWLDMEDEEDL
PGPEEVLEWLEEVLEGDTGDRGGDDDEEDDDDDEDEDDDEDDDDDDEDDDGDKVDDDDD

>CASQ2_Lophophanes_dichrous_transcript_assembly
CAGCTATGTAAGGTATCCAGGGCTTGGCCTGACAGCTTCTCCCGTCTGTCTCTCTCTTTGTTGTGAGTACAGGACAGCAAAGTTTGCTG
CTCGCCACCAGCCTTGCAGTGTTTGGGCTCAACACTAAGAAAGCAGCAGATAGAAACTCAGAGAAACTTAGTTCAAACTTCACCTGGAG
TCTTTGGTCTCCCTCACTTCTCAAATTAAGAGAGAGGGGCACATTTACCCACCCTTTGCTGCACTCCAAGGGTGCCCCTATGAAGGCAA
TTTGCTGGATCCTGGCAGGTTTTTACCTGCTTTTCTGCTGCAAGGCAGAAGAAGGACTGAACTTCCCTACTTATGATGGGAAAGACCGA
GTGATCGACCTGAACGAGAAGAATTACAAGCAGGCACTGAAGAAGTATGACATGCTCTGCCTGCTCTTCCATGAGCCTGTGAGCTCTGA
CAAGGTCTCCCAGAAGCAGTTCCAGATGACAGAGATGGTCCTGGAGCTGGCAGCTCAGGTCCTGGAGCCCAGGAGCATTGGCTTTGGGA
TGGTGGACTCCAAGAAGGATGCCAAGCTTGCTAAGAAGTTAGGCTTGGTTGAAGAGGGAAGTCTCTATGTCTTTAAGGATGAGCGGTTG
GTTGAGTTTGATGGGGAACTGTCTGCAGATGTCTTGGTGGAATTCCTCTTGGATTTGCTAGAAGACCCCGTGGAGGTCATAAACAGCAA
GCTGGAGCTTCAGGCCTTTGACCAGATCGACGACGAAATCAAACTCATCGGCTACTTCAAGGGAGAAGACTCAGAACATTACAAGGCAT
TTGAAGAAGCTGCTGAACAATTCCAGCCCTATGTCAAGTTTTTTGCCACCTTTGACAAAGGGGTTGCCAAGAAGCTGGGCCTAAAGATG
AATGAGGTGGACTTCTATGAACCCTTTATGGATGAGCCTGTTCACATCCCCGATAAGCCCTACTCCGAAGAGGAGCTGGTTGATTTTGT
GAGAGAGCACAGAAGGGCCACCTTGAGGAAGCTGCGCCCAGAGGACATGTTTGAGACGTGGGAGGATGACATGGAGGGTATCCACATCG
TAGCCTTTGCTGAAGAAGATGACCCAGATGGTTTTGAGTTCCTGGAAATCCTGAAGCAGGTTGCCAGGGACAACACCGATAATCCTGAC
CTGAGCATTGTCTGGATTGACCCTGATGACTTTCCTCTGCTCATCACTTACTGGGAGAAGACCTTCAAGATTGACCTGTTCAGACCACA
GATTGGGGTGGTGAACGTCACAGACGCTGACAGCGTCTGGATGGACATCAGAGATGATGATGACCTGCCCACAGCCGAGGAGCTGGAGG
ACTGGATAGAGGACGTGCTTTCTGGGAAGATAAATACCGAAGATGATGACGACGATGAAGATGACGATGATGACGATGATGATGATGAC
GACGACGATGACGATGACGACGATGAAGATGACGATGATGACGATGATGATGATGACGACGACGATGACGATGACGACGACGATGACGA
CGATGATGACGATGACGATGACTAACTGTGACCCTGTGCAGTTTGACTTGTGGGGCCCGAGAGCCCTTGCCCTGCGGCAGGTCCCTCCA
TTGGAAGACCTATGTTTGAGCATCAGCAAGCACCGCCGCTCCTCTTTCCCCCCTGCCCCGCTCCCCCTGCCCTCGCCCGTGCCGGGACC
CCCTCGCCTGATTCCCAGCGGTGCTGAGCCACCGGGACTGCCCCTGCGGAGGGCAGCACCTGCAGGCACCCAGCACCGCTGCAAATCAT
GCCTCCTTAGTAAGGACAGCAGGAATCTGCAGGGAATCTGCCCCGAGTGTCCCAGGGCAGGAGAAAAGTGCCTGCCTGCTTGCTGCCAG
AGGGACGCAGGGAGCCAGTTTGTATTTTCTGTGTTCATCAGCCCAGCACTTAACATCCAAAGACCAAATCTCACTTCAAGACATAGGGA
AAACCCTAACTGTAAAGTTTAGCCCTTTTATTAAACACAATGCCCTTTGCAGCTACTCTGCCAAGACCACTCAATCCTGCAAGTTCTGC
TGCGGGAGTCTGTAGCTACTCCCTAACATGCTAAGAGCCAGAAAAATGCTTTGGGAGGGGAGGGAGGTTTTAGGGATGCTATTAAAGTT
TTCAGTGATTCCACATAATGACAGGATATTCTTGTCTAGCAGTGCAATACAAAACCAAACACCGGGTCCCCTGTCTGAACTTCTTTCTT
CTCCAAAGCTCAGGCCACAGCTGGAGTCAGTGCAGGGGGCCTGATACAAGACACAAAGCTTGTGAGTTCCTGAACTGCTGGACAGGCTG
GCCCTGCTCTGAGCCCAAAGTGAGTCCAAACCACTGAGTCCGAAGATCATGGCACTGAGCCAAGCCAATGGAAATATCCATAAGGGCCC
ACCCTGAAGCATTCCTATTCCCCATGACCTTCCAGCTTTTGCTGACTCTTCACCCAAAGCCCATGTAGCATCTTTTCCCATGATCAAGA
TGTTGGGAGGGAACACATCCATATTAGATAGTGGATGAGGTGCATTGGGGTGTGAGCAGCAGACGGATCCAAGCTGTTTGCCTTTCGAG
TATTTCGTTTCTCCCTGCTTGGAGACTCAGATTTCCCATGTGAGAGGCCTCTTTTGACTGCAGGCTGAGAAGAAGCTCAGGGTTTGACC
AGTTTGCAGTCAAGAGGGTCACTCACAAAGACAAAGGTCTGTACCATGGTCCTGGCAAGTGTAGCACCATCCATGTGCAGGTCACTGTG
GCAGCATTAGGCCTCCCTGCTTTCCCATGCTGGCGGATTTCAGTTGTCTTAGCTTATCCTTGGCAATAATACAATGAATGGAGAGTTTG
GAGATGGTTTTATCTTTATAAACAGATTTATAAAGATGTTCTCCAGTTGTTCTGAGGTGCTAGCATAGGTAGTGTACACATCTCCTCTC
TTGCTATAAATGAAAAACATTGTTCCTTACACTGGCTTGGGACATAGGTAGGTGATGTGAGGGTGCGAGGGACAGAGGAGTCTGAGTTA
TGCAAATGTGAATCAGCCTGATGAGAAACAAGGTTTACACTCTATATATTTCCTTCATCAGATTAATTGCAAAGCAAGATTTTATAGAT
ATTTTTTTCCTGTCTGTTTATCAGTTTCTTTGACTCTCTCTATATTAGCTGAAATAGAAATTTGAAACTACTGGTATAAAATAGCTGAA

GTATTGATATGTTCTCATGTGTGCCATATCCCCTTGGTGGAAAATGCCAGCATGAACAACACATAGCTGGATTTCTCTCTGTCTCTGTG
CACTTGAATGAATACTGTGCAACATGGTAGATGACTATAGGCCAAGTTAGCTCCTTGTTGAGCAT

>CASQ2_Lophophanes_dichrous_CDS
ATGAAGGCAATTTGCTGGATCCTGGCAGGTTTTTACCTGCTTTTCTGCTGCAAGGCAGAAGAAGGACTGAACTTCCCTACTTATGATGG
GAAAGACCGAGTGATCGACCTGAACGAGAAGAATTACAAGCAGGCACTGAAGAAGTATGACATGCTCTGCCTGCTCTTCCATGAGCCTG
TGAGCTCTGACAAGGTCTCCCAGAAGCAGTTCCAGATGACAGAGATGGTCCTGGAGCTGGCAGCTCAGGTCCTGGAGCCCAGGAGCATT
GGCTTTGGGATGGTGGACTCCAAGAAGGATGCCAAGCTTGCTAAGAAGTTAGGCTTGGTTGAAGAGGGAAGTCTCTATGTCTTTAAGGA
TGAGCGGTTGGTTGAGTTTGATGGGGAACTGTCTGCAGATGTCTTGGTGGAATTCCTCTTGGATTTGCTAGAAGACCCCGTGGAGGTCA
TAAACAGCAAGCTGGAGCTTCAGGCCTTTGACCAGATCGACGACGAAATCAAACTCATCGGCTACTTCAAGGGAGAAGACTCAGAACAT
TACAAGGCATTTGAAGAAGCTGCTGAACAATTCCAGCCCTATGTCAAGTTTTTTGCCACCTTTGACAAAGGGGTTGCCAAGAAGCTGGG
CCTAAAGATGAATGAGGTGGACTTCTATGAACCCTTTATGGATGAGCCTGTTCACATCCCCGATAAGCCCTACTCCGAAGAGGAGCTGG
TTGATTTTGTGAGAGAGCACAGAAGGGCCACCTTGAGGAAGCTGCGCCCAGAGGACATGTTTGAGACGTGGGAGGATGACATGGAGGGT
ATCCACATCGTAGCCTTTGCTGAAGAAGATGACCCAGATGGTTTTGAGTTCCTGGAAATCCTGAAGCAGGTTGCCAGGGACAACACCGA
TAATCCTGACCTGAGCATTGTCTGGATTGACCCTGATGACTTTCCTCTGCTCATCACTTACTGGGAGAAGACCTTCAAGATTGACCTGT
TCAGACCACAGATTGGGGTGGTGAACGTCACAGACGCTGACAGCGTCTGGATGGACATCAGAGATGATGATGACCTGCCCACAGCCGAG
GAGCTGGAGGACTGGATAGAGGACGTGCTTTCTGGGAAGATAAATACCGAAGATGATGACGACGATGAAGATGACGATGATGACGATGA
TGATGATGACGACGACGATGACGATGACGACGATGAAGATGACGATGATGACGATGATGATGATGACGACGACGATGACGATGACGACG
ACGATGACGACGATGATGACGATGACGATGACTAA

>CASQ2_Lophophanes_dichrous_protein
MKAICWILAGFYLLFCCKAEEGLNFPTYDGKDRVIDLNEKNYKQALKKYDMLCLLFHEPVSSDKVSQKQFQMTEMVLELAAQVLEPRSI
GFGMVDSKKDAKLAKKLGLVEEGSLYVFKDERLVEFDGELSADVLVEFLLDLLEDPVEVINSKLELQAFDQIDDEIKLIGYFKGEDSEH
YKAFEEAAEQFQPYVKFFATFDKGVAKKLGLKMNEVDFYEPFMDEPVHIPDKPYSEEELVDFVREHRRATLRKLRPEDMFETWEDDMEG
IHIVAFAEEDDPDGFEFLEILKQVARDNTDNPDLSIVWIDPDDFPLLITYWEKTFKIDLFRPQIGVVNVTDADSVWMDIRDDDDLPTAE
ELEDWIEDVLSGKINTEDDDDDEDDDDDDDDDDDDDDDDDEDDDDDDDDDDDDDDDDDDDDDDDDDDD*

9.5 *Melospiza melodia* (song sparrow)

From: Trinity *de novo* assembly of SRR26155936. CASQ1 from complement of TRINITY_DN2237_c0_g1_i9. CASQ2 from TRINITY_DN5723_c0_g3_i2.

>CASQ1_Melospiza_melodia_transcript_assembly
GTCCGTCTGTCCCCGCCGTGTCCGTCTGTCCCCGCCGTGTCCCCTCCCGCCGCCGGAAGGGGCTGGCGCTGTCGCCGTGTCCCCAAGGG
GGTCGCCGGAGTCCTTGTCTGTCCCCGGGGGTCTGCATGTCCGTCTGTCCCCGGGGTCTGCGTGTCCGTCTGTCCCCGGGGTCTGTGTG
TCCGTCTGTCCCCAAGGCCTCTCCGTGTGTCCCCAGGGTGTCTCTGTCCCCTGGCTGTGTCCCTGCCCGCCTGTCCCTCTGTCCCTCGC
TCTCTGTGTCTGTCCTTGTGTCCGTGTGTCCATCCCCGTGTCCCCGTGTCCCTCTTGTCAGTGTGTCCCTGTCCCTCTGTCCCTGTCCC
GCGTCCCTCTGTCCCTCTCCGTGTCCCTCTGTCCTCCTGTCCCCCTGTCCCTCTGTCCGTCTGCCCCCGTCCCCATGTCCCCTCTGTGG
CTCCTGGCGCTGCTGGCCCTGGCCCCGAGGGTCCCGGGGCATCCCGGGGATTGTCCCGGTGTCTGTCCCGCTGTCTGTCCCGCCGTCTG
TCCCGCTGTCTGTCCCGGTGCCCATCCCGGTTCTCCCGGGGGCAGCGGGCACCGGCCCGAGGAGCTGCGGGCGCTCGAGCTCCGGGACT
TTGGGGACATCGAGGAGGAGCCCGAAGGCAGCGGGGACTGCTGGGGACAGCGCAGGCCTTCCAGTCCTTCATCGCCGCCCGGCGCTTCC
ACCCCTCCCTCCTCTTCCTCACCACCTTCGACACCCAGGGCCCCTCTGTGGCAACTCAGAGCTCAGAGCACGGCGGGGAGCTGGGACGC
CCTGCGCGGGACCCCCATCGTGGCCTTCGCCGTGGGGGATGATCCCGATGGGTTCGAGTTCGTGGAGACGCTGAGGGAGGTGGCCCAGG
ACAGGAGGGACCAGCCCGGCTTCAGCATCCTCTGGATCGACCCCGGAGATTTCCCGGGGCTCGTCCCCACCTGGGAGGACACCTTCGAC
ATCGACCTGTCCCGGCCACAGCTGGGCGTGGTCAATGGCACCGACTTGGCCACCAGCGTGTGGCTGGACATGGAGGATGAGGAGGATCT
GCCAGGGCCTGAGGAGGTCCTGGAGTGGCTCCAGGAGGTGCTGGAGGGGGACACTGGGGATGGTGAGGATGAAGAGGATGACGATGATG
AAGAGGATGAAGAGGATGACGATGATGAAGATGATGAAGATGATGACGATGATGATGAAGATGATGATGATGAAGTTGATGACGATGAT
GATGACTGACAACAGTGACGACAACGGTGATGATGATGGCAATGACAATGATGACAATGAAAATGATGATGATGATGATGATGATGATG
ATGATGATGAAAATGATGGCACTGACCATGACAATGACGATGACGTTGACTGACAATGACAACAAAGATGACGATGAAGACAAAGGTGA
TGACAATGACATTTACAATGACAATGATGTTGACGATGATGATGATGGCGACGATGATGATGACAATGATGATGACAACAAAACAATGA
TGACGTTGATGATGACAATGAAAATAATGGCAACAATGATGATGATGACAATCATGATGGTGATGAAAACAGTGACAATGATGATGATG
A

>CASQ1_Melospiza_melodia_CDS
ATGTCCCCTCTGTGGCTCCTGGCGCTGCTGGCCCTGGCCCCGAGGGTCCCGGGGCATCCCGGGGATTGTCCCGGTGTCTGTCCCGCTGT
CTGTCCCGCCGTCTGTCCCGCTGTCTGTCCCGGTGCCCATCCCGGTTCTCCCGGGGGCAGCGGGCACCGGCCCGAGGAGCTGCGGGCGC
TCGAGCTCCGGGACTTTGGGGACATCGAGGAGGAGCCCGAAGGCAGCGGGGACTGCTGGGGACAGCGCAGGCCTTCCAGTCCTTCATCG
CCGCCCGGCGCTTCCACCCCTCCCTCCTCTTCCTCACCACCTTCGACACCCAGGGCCCCTCTGTGGCAACTCAGAGCTCAGAGCACGGC
GGGGAGCTGGGACGCCCTGCGCGGGACCCCCATCGTGGCCTTCGCCGTGGGGGATGATCCCGATGGGTTCGAGTTCGTGGAGACGCTGA
GGGAGGTGGCCCAGGACAGGAGGGACCAGCCCGGCTTCAGCATCCTCTGGATCGACCCCGGAGATTTCCCGGGGCTCGTCCCCACCTGG
GAGGACACCTTCGACATCGACCTGTCCCGGCCACAGCTGGGCGTGGTCAATGGCACCGACTTGGCCACCAGCGTGTGGCTGGACATGGA
GGATGAGGAGGATCTGCCAGGGCCTGAGGAGGTCCTGGAGTGGCTCCAGGAGGTGCTGGAGGGGGACACTGGGGATGGTGAGGATGAAG

AGGATGACGATGATGAAGAGGATGAAGAGGATGACGATGATGAAGATGATGAAGATGATGACGATGATGATGAAGATGATGATGATGAA
GTTGATGACGATGATGATGACTGA

>CASQ1_Melospiza_melodia_protein
MSPLWLLALLALAPRVPGHPGDCPGVCPAVCPAVCPAVCPGAHPGSPGGSGHRPEELRALELRDFGDIEEEPEGSGDCWGQRRPSSPSS
PPGASTPPSSSSPPSTPRAPLWQLRAQSTAGSWDALRGTPIVAFAVGDDPDGFEFVETLREVAQDRRDQPGFSILWIDPGDFPGLVPTW
EDTFDIDLSRPQLGVVNGTDLATSVWLDMEDEEDLPGPEEVLEWLQEVLEGDTGDGEDEEDDDDEEDEEDDDDEDDEDDDDDDEDDDDE
VDDDDDD*

>CASQ2_Melospiza_melodia_transcript_assembly
ATTTTTGTCAATTCCTAGTAGGACTGCCTGTGCCTTGACTCCCTGTGTGAGTGAAGTCTGTCCCTTCTCAAACCCTGCCTGCATATTCC
CAGGACTGACATCTTCCACTCCCTCCCTCTTGCCCACTCTGTCTCTCTCCGATGATGCAGAGTGCCAGCAAGTCCCTCAATGGTGTCAT
ATCTGTCCTTTTTATTCATTGCAGGGTTTATTTTTAGCCTGAAACAACTGCTTCCTAAAAATGGAGTTCCTAATGACACGGGAGCTGGA
CACAGCTATGTAAGGTATCCAGGGCTTGGCCTGACAGCTTCTCCCGTCTGTCTCTCTCTTTGTTGTGAGTACAGGACAGCAAAGTTTGC
TGCTCCCCGCCAGCCTTGCAGTGTTTGGGCTCAACTCTAGGAAAGCAGCAGAGAGAAACTTAGAGAAACTAGGAGAAACTTAGCTCAAA
CTTCACCTGGAGTCCTTGGTCTCCCTCTCTTCTCAAATCAAGAAAGAGGGGGACATTTACCCACCCTTTGCTGCACTCCAAGGGTGCCC
CTATGAAGGCAATTTGCTGGATCCTGGCAGGTTTTTACCTGCTTTTCTGCTGCAAGGCAGAAGAGGGACTGAACTTCCCTACTTACGAT
GGGAAAGACCGAGTGATCGACCTGAACGAGAAGAATTACAAGCAGGCACTGAAGAAGTATGACATGCTCTGCCTGCTCTTCCATGAGCC
TGTGAGCTCTGACAAGGTCTCCCAGAAGCAGTTCCAGATGACAGAGATGGTGCTGGAGCTGGCAGCTCAGGTCCTGGAGCCCAGGAGCA
TTGGCTTTGGGATGGTGGACTCCAAGAAGGATGCCAAGCTTGCCAAAAAGTTAGGCTTGCTTGAAGAGGGGAGTCTCTATGTCTTTAAG
GACGAGCGGTTGGTTGAATTTGATGGGGAACTGTCTGCAGATGTCTTGGTGGAATTCCTCTTGGATTTGCTGGAGGACCCCGTGGAGAT
CATCAACAGCAAGCTGGAGCTTCAGGCCTTTGACCAGATCGACGACGAAATCAAACTCATCGGCTACTTCAAGGGAGAAGACTCAGAAC
ATTTCAAGGCATTTGAAGAAGCTGCTGAACAATTCCAGCCCTACGTCAAGTTCTTTGCCACCTTTGACAAAGGGGTTGCCAAGAAGCTT
GGCCTAAAGATGAATGAGGTGGACTTCTACGAACCCTTTATGGATGAGCCTGTCCACATCCCTGATAAGCCTTACTCAGAAGAGGAGCT
GGTTGATTTTGTGAGAGAGCACAGAAGGGCCACCTTGAGGAAGCTGCGCCCCGAGGACATGTTTGAGACATGGGAGGATGACATGGAGG
GCATTCACATCGTAGCCTTTGCTGAAGAAGATGACCCAGATGGTTTTGAGTTCCTGGAAATCCTGAAGCAGGTTGCCAGGGACAACACC
GATAATCCCGACCTGAGCATTGTCTGGATTGACCCTGACGACTTTCCTTTGCTCATCACTTACTGGGAGAAGACCTTCAAGATCGACCT
GTTCAGACCACAGATTGGGGTGGTGAACGTCACAGATGCTGACAGCGTCTGGATGGACATCAGAGATGATGACGACCTGCCCACAGCCG
AGGAGCTGGAGGACTGGATAGAGGACGTGCTTTCTGGGAAGATAAACACCGAGGATGATGACGACGATGAAGACGACGACGATGACGAC
GATGACGACGATGACGATGATGACGACGACGATGACGATGATGATGATGACGACGACTAACTGTGACTCTGTGCAGTTTGACTTGTGGG
GCCCGAGAGCCCCGGCCCTGCGGCAGGTCCCTCCATCGGAAGACCTTTGTTTGAGCATCAGCAAGCACCGCTGCTCCTCTCTTTCCCCG
GCCCCGCTCCCCTGCCCTCGCCCTTGCCGGGAGCCCCTGGCCTGATTCCCAGCGGTGCTGAGCCCCCGGGGCCGCCCCTGTGGAGGGCA
GCGCCTGCGGGCGCCCAGCACCGCAGGGAATCACGCCTCCTTAGCAAGGACTGCAGGAACCTGCAGGGAATCTGCCCCAGTGTCCCAGG
GCAGGAGAAAAGTGCCTGCCTGCTTGCTGCCAGAGGGACGCAGGGAGCCACTTTGTATTTTCTGTGTTCATCAGCCCAGCACTTAACAT
CCAAAGACCAAATCTCACTTCAAGACATAGGGAAAACCCTAACTGTAAAGTCTAGCCCTTTTATTAAACACGATGCCCTTTGCAGCTAC
TCTGCCAAGACCACTCAATCCTGCAAGTTCTGCTGTGGGAGTCTGTAGCTACTCCCTAACATGCTAAGAGCCAGAAAAATGCTTTGGGA
GGGGAGGGAGGTTTTAGGGATGCTATTAAAGTTTTCAGTGATTCCACATAACGACAGGATATTCTTGTCTGGCAGCGCAATGCAAAACC
AAACACCGGGTCCCCTGTCTGAGCCTCTTTCTTCTTGAAAGCTCGGGCCACAGCTGGGGTCAGTGCAGGGATCCTGATATGAGACACAA
AGCTCATGAACTCCTGAACTGCTGGACAGGCTGGTCCAGCTCTGAGCCCAGTGTGAGTCCAAACCACTGAGCCTGAAGATCATGGCACT
GAGCCAAGCCAATGGAAACATCCACAGGGGCCCATCCTGAAGCACTCCTATTCCCCATGACCTTCCAGCTTTTGCTGACTCTTCACCCA
AAGCCCATGCAGCATCTTTTCCCATGATCAAGATGTCAGGAGGGAACACATCCATCCAAACACATTAGACAGTGGATGAGGTGTGTTGG
GGTGTGAGCAGTGAAGAGGATTCAAGCTGTTTGGCTTTTGAGTACTTAGTTGCACAGCTACTCCCTGCCTAGAGACTCAGATGTCCCAT
GTGAGAAGCCTCTCTTGGCTGCAGGCTGAGAAGGAGTTGAGGGTGCCAGTTTGCAGTCAAGGGGGTCACTCACAAAGCCCAGGGTCCAT
TCCATGGTCCTGGTCAGTGCAGCACCATCCATGTGCCAGGCACTGTGGCTGCATTAGGTCTCCCTGCTTCCCCATGCTGGCAAATTTCA
ATTGTCTCGGCTTCTTATGTTTGGCAATAGTGCAATGTATGGAGAGTTCAGAGATGTATAAATACATTTATAGAGATGTTCTGCCGTTG
TTCAGAGTTGTTAGCATAGGTAGTGTACACATCTCCTCTCTTGCTACAAACAAAAAACATTGTTCTGACCATCCTTATACTGGCTTAGG
ACAGAGAAATGTGATGTGAGGGACAGAAGAATCAGTTACACAAATGTGAATCTTGGTGAGAAACAAGGTTTACACACTATATATTTCCT
TTATCAGATGAACTGCAAAGCAAGATTTTATAGATATTTTTTCCCCTACCTGTTGATGAGTTTCTTTGACTCCCTCTCTCTACATTAGC
TGAAATAGAAATTTGAAACTACTGGTATGAAAAAGCTGAAGTATTGCTATGTTCCCATTTGTGCCATGTCCCCTTGCTGGAAAATACCA
GTATGAACAAGACATAGCTAGATTTCTCTCTGTCTCTATGCACTAGAATGAATACTATGCTAACATGGTAGATGGCTTATGCCCAATTT
AGCTCCTTGCTGAGCATCAAACCCTCAACATGTGCTTTTCAGACACAGCTTGTTGGAAGAATAGATAGGGCAGCAAAGCCCCCAGGTCA
ACCATTTGCTCTTCCATGCTTTCTTATGATTGCTCAGAAGATGAACCAAGTTTGTTCAGGGAGAACAATCAACTGGTTGCTCTGAGGTG
ATGAATAACTTCCAAAAGTCTTGAAATTCAGTGTGCTCTCAGCATGGAGGTTTCCTCCTCTCTTCCATTCCTACTCTCTGCTGGCTGAC
AGGGTGCCCAGCACTCTCCTTTTTCCTCAAGGTTTGCTGGGGGAGCCCAGCTCAAGGAGCAGGACTCAAGGTCTCATTCACCCACACTG
GTAAGGAGCAAGAGGAGGCAGAAGCAGCCCTTTGGTATCCTCCTGGTCTGTGATTAAGGGACAACTACTGGCCTGAGGAAAGAACTAGA
TTATCTTTACCTTTGGGCTATTCCCAGGAGCAAATCATGAACAGTTTCACCCTGCATAAGGTAACAGCCTGCAATGACCATGTGCCTCC
TCATCAGTCACTCCAAACACAAAGAATAATGAAAAGTTCCTCTACTGGCTTTCCTTACTGGCACAATCTCCAGATGTGCCGAGTGCCTG
AGTGCTTTCTGTGTGCTCCTCTCCCCCTGTGGATGCCCCTCTGCTCTGAAGTGCCCCACTCTAGATGCTCTGAGAGGCCCAGATTGCCC
AGGCAGACCCTGCAGACAGCACTAGGGACATGTTCAAGTGCACAGGTGTTCCACAGGCACTTGAACATGTCAGAGCAAGAGACCTCTAG
GGGGAAAAGAAATGATGTACAAGTGTAGAGGCTGAACTCATACTGGGGAGTGGTGCCAGTTGTTTGTGGAAAGTTTCCCTTGAGTTATG
CAGGAAAGAGCTAATCCTACAGCTGGCACTCCTTCATGGTGCCAGGGATGGGGCTGTCCTATTTCTCTTCCCTCAGTCTGCCATTACAG

TTCTCAGCATTGTCTTTACCCACAAACAGGAACTGTTTATTGCCAGCTTTTGGGACAATCACATAAAAAGTTGGGAAAGCGTTGCCACA
GAGGAAGGAGGGCAGCAATGAGGATGGGTGGGCAGCAAAGCTTTTCCCATTAACACCTTGTCTGCCCCAAGATCCTAATGACCAAACAG
CTGAAGGGCACAATGCCCCTTGTGAAGGAGAGGGCAGCTTGAATTTGAGTGTTTTGCTTGCAGGGACAGGTAAGACAGGGGTGTGGGGA
TGACACACAGGATCACAGGCCCAGGTGCTCCTGGGCTCCTCTGTCGCAAGTAGGAACACCTGGCATTTGAAAGGCACTGGTGGCCCCGA
GGAGCAAAGCCAGCCTCACCCTGCCCACTATACACTGGGAGGAAAGAGTCAGCACATTTGGATGAGGAACCTGCAAAAAAAACCCTTTAT
TTAAAAAACACAAATTTGTGTATGTGGGCAGCACTGAGTGCTTAACAAGCACGTCTTTCTTCACAAATAGCTCTTTTGCTCCAGCATGT
TAACTAGACATATTAAACAGGTCTTTATACAGATAAAGACAGGAATTGTATTTTGCCCTCACCCAACCACGAGTGCTTCAAGC

>CASQ2_Melospiza_melodia_CDS
ATGAAGGCAATTTGCTGGATCCTGGCAGGTTTTTACCTGCTTTTCTGCTGCAAGGCAGAAGAGGGACTGAACTTCCCTACTTACGATGG
GAAAGACCGAGTGATCGACCTGAACGAGAAGAATTACAAGCAGGCACTGAAGAAGTATGACATGCTCTGCCTGCTCTTCCATGAGCCTG
TGAGCTCTGACAAGGTCTCCCAGAAGCAGTTCCAGATGACAGAGATGGTGCTGGAGCTGGCAGCTCAGGTCCTGGAGCCCAGGAGCATT
GGCTTTGGGATGGTGGACTCCAAGAAGGATGCCAAGCTTGCCAAAAAGTTAGGCTTGCTTGAAGAGGGGAGTCTCTATGTCTTTAAGGA
CGAGCGGTTGGTTGAATTTGATGGGGAACTGTCTGCAGATGTCTTGGTGGAATTCCTCTTGGATTTGCTGGAGGACCCCGTGGAGATCA
TCAACAGCAAGCTGGAGCTTCAGGCCTTTGACCAGATCGACGACGAAATCAAACTCATCGGCTACTTCAAGGGAGAAGACTCAGAACAT
TTCAAGGCATTTGAAGAAGCTGCTGAACAATTCCAGCCCTACGTCAAGTTCTTTGCCACCTTTGACAAAGGGGTTGCCAAGAAGCTTGG
CCTAAAGATGAATGAGGTGGACTTCTACGAACCCTTTATGGATGAGCCTGTCCACATCCCTGATAAGCCTTACTCAGAAGAGGAGCTGG
TTGATTTTGTGAGAGAGCACAGAAGGGCCACCTTGAGGAAGCTGCGCCCCGAGGACATGTTTGAGACATGGGAGGATGACATGGAGGGC
ATTCACATCGTAGCCTTTGCTGAAGAAGATGACCCAGATGGTTTTGAGTTCCTGGAAATCCTGAAGCAGGTTGCCAGGGACAACACCGA
TAATCCCGACCTGAGCATTGTCTGGATTGACCCTGACGACTTTCCTTTGCTCATCACTTACTGGGAGAAGACCTTCAAGATCGACCTGT
TCAGACCACAGATTGGGGTGGTGAACGTCACAGATGCTGACAGCGTCTGGATGGACATCAGAGATGATGACGACCTGCCCACAGCCGAG
GAGCTGGAGGACTGGATAGAGGACGTGCTTTCTGGGAAGATAAACACCGAGGATGATGACGACGATGAAGACGACGACGATGACGACGA
TGACGACGATGACGATGATGACGACGACGATGACGATGATGATGATGACGACGACTAA

>CASQ2_Melospiza_melodia_protein
MKAICWILAGFYLLFCCKAEEGLNFPTYDGKDRVIDLNEKNYKQALKKYDMLCLLFHEPVSSDKVSQKQFQMTEMVLELAAQVLEPRSI
GFGMVDSKKDAKLAKKLGLLEEGSLYVFKDERLVEFDGELSADVLVEFLLDLLEDPVEIINSKLELQAFDQIDDEIKLIGYFKGEDSEH
FKAFEEAAEQFQPYVKFFATFDKGVAKKLGLKMNEVDFYEPFMDEPVHIPDKPYSEEELVDFVREHRRATLRKLRPEDMFETWEDDMEG
IHIVAFAEEDDPDGFEFLEILKQVARDNTDNPDLSIVWIDPDDFPLLITYWEKTFKIDLFRPQIGVVNVTDADSVWMDIRDDDDLPTAE
ELEDWIEDVLSGKINTEDDDDDEDDDDDDDDDDDDDDDDDDDDDDDDDD*

9.6 *Onychostruthus taczanowskii* (white-rumped snowfinch)

From: Trinity *de novo* joint assembly of SRR18552131 and SRR18552120. CASQ1 from complement of TRINITY_DN570_c2_g1_i19. CASQ2 from TRINITY_DN7783_c0_g1_i7.

>CASQ1_Onychostruthus_taczanowskii_transcript_assembly
GCCTTGCCGGTGGCTCCTGGCGCTGCTGGCCCTGGTCCCGGGGGGCCTCCCGGGGGTCCCGGGGGTCCCGGGCTGTCCCGGGGGGGCTC
TGTCGCTGCCGCCCCCGGACGGGCGGAGCCGCCTCCTCCCGGTGCCCCCCGGGACCCTCCCCGAGCTGCGCCGCCGCTTCCCGGTGCTG
GCGCTGCTGCGGAGCCCCGGGCACCGGGAGCACCGGCCCGAGGAGCTGCGGGCGCTCGAGCTGGTGGCGCAGGCGCTGGAGCCCCGCGG
GGTCGGCTTCGGCATGGTGGGCACGGAGAGCGACCCCGAGCTGGCCCGGGACTGGGGCCTGGGGGGGCGCTGGCGCCTGGCGCTGCTGA
GGGGACACTCGGTGACAGAGTACCCCGGGGAGCTGGCAGTGCCAGCCCTGCTGTGCTTCCTGAGCCAGGCGCTGCCCCCCCCCGTGCAG
GTGCTGGTGGCCGGGCCGCAGCTCGGGGACTTTGGGGACATCGAGGAGGAGCCCAAAGCCATCGGCTTCTTCCGGGGACCCGAGTCACG
GGCTTTCCAGTCCTTCTCGGCCGCCGCCCGCCGCCTCCACCCCTCCCTCCTCTTCTTCGCCACCTTCGACCCCGAGGCGGCGCGGGCGC
TGGGGCTGCGCCCCGAGCGGGTTTTGCTCTTCGAGCCCTTCCTGGAGCGGCCCCGGCGCTTCCGGGGGGACCCCCGGGACCCCCCCCAG
ATGGAGGCGTTCCTGCAGCGCGGCCGGCGGGCCACTCTGCGGAAACTCAAAGCTCAGAGCATGGCCGGGAGCTGGGAGGACGCCCTGGA
CGGGACCCCCATCGTGGCCTTCGCCAAGGGGGATGATCCCGACGGGTTCGAGTTCCTGGAGACCCTGAAGGAGGTGGCCCAGGCCAGGA
GGGACCGGCCCGGCTTCGGCGTGCTCTGGATCGACCCCGGCGACTTCCCGGGGCTCGTCCCCTCCTGGGAGGACACCTTTGACATCGAC
CTGTCCCGGCCACAGCTGGGCGTGGTCAATGGCACCGACCACGCCAGCAGCGTGTGGCTGCACATGGAGCACGAGGAGGACCTGCCGGG
GCCCGAGGAGGTCCTGGAGTGGCTCCAGGAGGTCCTGGAGGGGGACACCGGGGACAGCGGGGACGACGGCCAGGGTGACGATGAAGATG
AGGAAGATGAAGATGAAGAAGATGAAGATGATGAAGAAGATGGAGATGAAGATGATGAAGATGAGGAAGATGATGAAGACGATGAAGAT
GATGAAGATGATGATGATGAAGATGAAGATGAGGAGCAATTTTGGGTCAAAACCAGCCCAAAATGGACCCTTGGTTCTCCACGATGATG
AAGATGAAGATGATGAAGATGACAATGAATTTTAGGTCAAAAAATGCCCAAATTGGGCCCTTGTCCCACCCCACCCCCACCCGGAGCCT
TCCTCCCTCCCCTCCTCCCCCTCGCCCCTCCCCCAATGAAGCCCCGCCCCCAATAAAGCCCCGCCCCCTCAGCGTTGTCCACTCTGGGT
TCT

>CASQ1_Onychostruthus_taczanowskii_CDS_partial
GCCTTGCCGGTGGCTCCTGGCGCTGCTGGCCCTGGTCCCGGGGGGCCTCCCGGGGGTCCCGGGGGTCCCGGGCTGTCCCGGGGGGGCTC
TGTCGCTGCCGCCCCCGGACGGGCGGAGCCGCCTCCTCCCGGTGCCCCCCGGGACCCTCCCCGAGCTGCGCCGCCGCTTCCCGGTGCTG
GCGCTGCTGCGGAGCCCCGGGCACCGGGAGCACCGGCCCGAGGAGCTGCGGGCGCTCGAGCTGGTGGCGCAGGCGCTGGAGCCCCGCGG
GGTCGGCTTCGGCATGGTGGGCACGGAGAGCGACCCCGAGCTGGCCCGGGACTGGGGCCTGGGGGGGCGCTGGCGCCTGGCGCTGCTGA
GGGGACACTCGGTGACAGAGTACCCCGGGGAGCTGGCAGTGCCAGCCCTGCTGTGCTTCCTGAGCCAGGCGCTGCCCCCCCCCGTGCAG

GTGCTGGTGGCCGGGCCGCAGCTCGGGGACTTTGGGGACATCGAGGAGGAGCCCAAAGCCATCGGCTTCTTCCGGGGACCCGAGTCACG
GGCTTTCCAGTCCTTCTCGGCCGCCGCCCGCCGCCTCCACCCCTCCCTCCTCTTCTTCGCCACCTTCGACCCCGAGGCGGCGCGGGCGC
TGGGGCTGCGCCCCGAGCGGGTTTTGCTCTTCGAGCCCTTCCTGGAGCGGCCCCGGCGCTTCCGGGGGGACCCCCGGGACCCCCCCCAG
ATGGAGGCGTTCCTGCAGCGCGGCCGGCGGGCCACTCTGCGGAAACTCAAAGCTCAGAGCATGGCCGGGAGCTGGGAGGACGCCCTGGA
CGGGACCCCCATCGTGGCCTTCGCCAAGGGGGATGATCCCGACGGGTTCGAGTTCCTGGAGACCCTGAAGGAGGTGGCCCAGGCCAGGA
GGGACCGGCCCGGCTTCGGCGTGCTCTGGATCGACCCCGGCGACTTCCCGGGGCTCGTCCCCTCCTGGGAGGACACCTTTGACATCGAC
CTGTCCCGGCCACAGCTGGGCGTGGTCAATGGCACCGACCACGCCAGCAGCGTGTGGCTGCACATGGAGCACGAGGAGGACCTGCCGGG
GCCCGAGGAGGTCCTGGAGTGGCTCCAGGAGGTCCTGGAGGGGGACACCGGGGACAGCGGGGACGACGGCCAGGGTGACGATGAAGAT
GAGGAAGATGAAGATGAAGAAGATGAAGATGATGAAGAAGATGGAGATGAAGATGATGAAGATGAGGAAGATGATGAAGACGATGAAG
ATGATGAAGATGATGATGATGAAGATGAAGATGAGGAGCAATTTTGGGTCAAAACCAGCCCAAAATGGACCCTTGGTTCTCCACGATG
A

>CASQ1_Onychostruthus_taczanowskii_protein_partial
XPCRWLLALLALVPGGLPGVPGVPGCPGGALSLPPPDGRSRLLPVPPGTLPELRRRFPVLALLRSPGHREHRPEELRALELVAQALEPR
GVGFGMVGTESDPELARDWGLGGRWRLALLRGHSVTEYPGELAVPALLCFLSQALPPPVQVLVAGPQLGDFGDIEEEPKAIGFFRGPES
RAFQSFSAAARRLHPSLLFFATFDPEAARALGLRPERVLLFEPFLERPRRFRGDPRDPPQMEAFLQRGRRATLRKLKAQSMAGSWEDAL
DGTPIVAFAKGDDPDGFEFLETLKEVAQARRDRPGFGVLWIDPGDFPGLVPSWEDTFDIDLSRPQLGVVNGTDHASSVWLHMEHEEDLP
GPEEVLEWLQEVLEGDTGDSGDDGQGDDEDEEDEDEEDEDDEEDGDEDDEDEEDDEDDEDDEDDDDEDEDEEQFWVKTSPKWTLGSPR
*

>CASQ2_Onychostruthus_taczanowskii_transcript_assembly
TGTGAGTACAGGACAGCAAAGTTTGCTGCTCCCGACCAGCCTTGCAGTGTTTGGGCTCAAGTCTAGGAAAGCAGCAGAGAGAAACTTAG
AGAAACTAAGAGAAACTTAGTTCAAACTTCACCTGGAGTCTTTGGTCTCCCTCTCTTCTCAAATTTAGAAACAGGGGGACATTTACCCA
CCCTTTGCTGCACTCCAAGGGTGCCCCTATGAAGGCAATTTGCTGGATCCTGGCAGGTTTTTACCTGCTTTTCTGCTGCAAGGCAGAAG
AGGGACTGAACTTCCCTACTTACGATGGGAAAGACCGAGTGATCGACCTGAACGAGAAGAATTACAAGCAGGCACTGAAGAAGTATGAC
ATGCTCTGCCTGCTCTTCCATGAGCCTGTGGGCTCTGACAAGGTCTCCCAGAAGCAGTTCCAGATGACAGAGATGGTCCTGGAGCTGGC
AGCTCAGGTCCTGGAGCCCAGGAGCATTGGCTTTGGGATGGTGGACTCCAAGAAGGATGCCAAACTTGCCAAAAAGTTAGGCTTGCTTG
AAGAGGGAAGTCTCTATGTCTTTAAGGATGAGCGGTTGGTTGAATTTGATGGGGAACTGGCTGCAGATGTCTTGGTGGAATTCCTCTTG
GATTTGCTGGAAGACCCCGTGGAGGTCATCAACAGCAAGCTGGAGCTTCAGGCCTTTGACCAGATCGACGACGAAATCAAACTCATCGG
CTACTTCAAGGGAGAAGACTCAGAACATTTCAAGGCATTTGAAGAAGCTGCTGAACATTTCCAGCCCTATGTCAAGTTCTTTGCCACCT
TTGATAAAGGGGTTGCCAAGAAGCTAGGCCTAAAGATGAATGAGGTGGACTTCTATGAACCCTTTATGGATGAGCCTGTTCACATCCCT
GATAAGCCTTACTCAGAAGAGGAGCTGGTGGACTTTGTGAGAGAGCACAGAAGGGCCACCTTGAGGAAGCTGCGCCCAGAGGACATGTT
TGAGACGTGGGAGGATGACATGGAAGGTATTCACATTGTAGCCTTTGCTGAAGAAGATGACCCAGACGGTTTTGAGTTCCTGGAAATCC
TGAAGCAGGTTGCCAGGGACAACACCGATAATCCTGACCTGAGCATCGTCTGGATTGACCCTGACGACTTTCCTTTGCTCATCACGTAC
TGGGAGAAGACCTTCAAGATCGACCTGTTCAGACCACAGATCGGGGTGGTGAACGTCACTGACGCTGACAGCGTCTGGATGGACATCAG
AGATGATGACGACCTGCCCACAGCCGAGGAGCTGGAGGACTGGATAGAGGACGTGCTTTCCGGGAAGATAAATACCGAAGATGACGACG
ACGATGAAGATGACGACGACGATGATGACGACGATGATGATGACGACGACGATGATGACGACGACGATGACGACGACGACGATGATGAC
GACTAACTGTGACTCTGTGCAGTTTGACTTGTGGGGCCCGAGAGCCCTGGCCCTGCGGCAGGTCCCTCCATTTGAAGACCTATGTTTGA
GCATCAGCAAGCACCGCTGCTCCTCTTTATCCCCCGGCCCCGCTCCCCCTGCCCTCGCTCTTGCCGGGAGCCCCTTGCCTGATTCCCAG
TGGTGCTGAGCCAGCGGGACTGCCCCGGCGGAGGGCAGCACCTGCAGGCACCCAGCACCGCTGCAAATCATGCCTCCTTAGTAAGGACA
GTAGGAATCTGCAGGGAATCTGCCCCGAGTGTCCCAGGGCAGGAGAAAAGTTCCTGCCTGCTTGCTGCCAGAGGGACGCAGGGAGCCAG
TTTGTATTTTCTGTGTTCATCAGCCCAGCACTTAACATCCAAAGACCAAATCTCACTTCAAGACATAGGGAAAACCCTAACTGTAAAGT
TTAGCCCTTTTATTAAACACAATGCCCTTTGCAGCTACTCTGCCAAGACCACTCAATCCTGCAAGTTCTGCTGAGGGAGTCTGTAGCTA
CTCCCTAACATGCTAAGAGCCAGAAAAATGCTTTGGGAGGGGAGGGAGGTTTTAGGGATGCTATTAAAGTTTTCAGTGATTCCACATAA
TGACAGGATATTCTTGTCTGGCAGTGCAATGCAAAACCAAACACCGGGTCCCCTGTCTGAACTTCTTTCTTCTTGAAAGCTGAGGCCAC
AGCTGGAGTCAGTGCAGGGGCCCTGATATGAGACACAAAGCTCATGAGTTCCTGAACTGCTGGACAGGCTGGTCCAGCTCGGAGCCCAG
TGTGAGTACAAACCACTGAGTCTGAAGATCACGGCACTGAGCCAAGCCAATGGGAACATTCACAGGGGCCCACCCTGAAGCACTTTTAT
TCCCCATGACCTTCCAGCTTTTGCTGACTCTTCACCCAAAGCCAATGTAGCATCTTTTCCCATGATCAAGATGTCAGGAGGGAACACAT
CCATCCAAACATATTAGATAGTGGATGAGTTGCAGTGGGGTGTGAGCAGCGGAGGGATTCAAGCTGTTTGGCTTTTGAGTATTTAGTTG
CAGTTACTCCCTGCTTAGAGACTCAGATATCCCATGTGAGAAGCCCCTTTTGGCTGCAGGCTGAGAAGGAGTTCAGGGTACCAGTTTGC
AGTCAAGGGGGTCACTCACAAAGCCCAGGGTCCGTACCTCGGTCCTGGCGAGTGCAGCACCATCCATGTGCAGGTCACTGTGGCAGCAT
TAGTTCTCCCTGCTTTCCCATGCTGGCAGATTTCAATTGTCTTAGCGTCTTATTAGTTTGGCAATAGTGCAATGAATGGAGAGTTCAGA
GATGGTGTAAAATACATTTATAAAGATGTTCTGCAGTTGTTCAGAGTTGCTGGCATAGCTGGTGTACACATCTCCTCTCTTGCTACAAA
CAAAAAACATTGTTCTGACCATCCTTATACTGCCTTAGGACAGAGGTACGTGATGTGAGGGGAAATAACAGCGTGAGGAACAGAAGAAT
CAGTCACACAAATGCGAATCTTGATGAGAAACAAGGTTTACACACTATATATTTCCTTTATTAGATGAACTGCAAAGCAAGATTTTATA
GATTTTTTCCTGCCTGTTGATAAGTTTCTTTGACTCCCTCTCTCTACATTAGCTGAAACAGAAATTTGAAACTACTGATATGAAAAAGC
TGAAGTATTGATATGTTCCCGTGTGTGCCATATCCCCTTGGTGGAAAATACCAGTATGAACAACACATAGGTGGATTTC

>CASQ2_Onychostruthus_taczanowskii_CDS
ATGAAGGCAATTTGCTGGATCCTGGCAGGTTTTTACCTGCTTTTCTGCTGCAAGGCAGAAGAGGGACTGAACTTCCCTACTTACGATGG
GAAAGACCGAGTGATCGACCTGAACGAGAAGAATTACAAGCAGGCACTGAAGAAGTATGACATGCTCTGCCTGCTCTTCCATGAGCCTG

TGGGCTCTGACAAGGTCTCCCAGAAGCAGTTCCAGATGACAGAGATGGTCCTGGAGCTGGCAGCTCAGGTCCTGGAGCCCAGGAGCATT
GGCTTTGGGATGGTGGACTCCAAGAAGGATGCCAAACTTGCCAAAAAGTTAGGCTTGCTTGAAGAGGGAAGTCTCTATGTCTTTAAGGA
TGAGCGGTTGGTTGAATTTGATGGGGAACTGGCTGCAGATGTCTTGGTGGAATTCCTCTTGGATTTGCTGGAAGACCCCGTGGAGGTCA
TCAACAGCAAGCTGGAGCTTCAGGCCTTTGACCAGATCGACGACGAAATCAAACTCATCGGCTACTTCAAGGGAGAAGACTCAGAACAT
TTCAAGGCATTTGAAGAAGCTGCTGAACATTTCCAGCCCTATGTCAAGTTCTTTGCCACCTTTGATAAAGGGGTTGCCAAGAAGCTAGG
CCTAAAGATGAATGAGGTGGACTTCTATGAACCCTTTATGGATGAGCCTGTTCACATCCCTGATAAGCCTTACTCAGAAGAGGAGCTGG
TGGACTTTGTGAGAGAGCACAGAAGGGCCACCTTGAGGAAGCTGCGCCCAGAGGACATGTTTGAGACGTGGGAGGATGACATGGAAGGT
ATTCACATTGTAGCCTTTGCTGAAGAAGATGACCCAGACGGTTTTGAGTTCCTGGAAATCCTGAAGCAGGTTGCCAGGGACAACACCGA
TAATCCTGACCTGAGCATCGTCTGGATTGACCCTGACGACTTTCCTTTGCTCATCACGTACTGGGAGAAGACCTTCAAGATCGACCTGT
TCAGACCACAGATCGGGGTGGTGAACGTCACTGACGCTGACAGCGTCTGGATGGACATCAGAGATGATGACGACCTGCCCACAGCCGAG
GAGCTGGAGGACTGGATAGAGGACGTGCTTTCCGGGAAGATAAATACCGAAGATGACGACGACGATGAAGATGACGACGACGATGATGA
CGACGATGATGATGACGACGACGATGATGACGACGACGATGACGACGACGACGATGATGACGACTAA

>CASQ2_Onychostruthus_taczanowskii_protein
MKAICWILAGFYLLFCCKAEEGLNFPTYDGKDRVIDLNEKNYKQALKKYDMLCLLFHEPVGSDKVSQKQFQMTEMVLELAAQVLEPRSI
GFGMVDSKKDAKLAKKLGLLEEGSLYVFKDERLVEFDGELAADVLVEFLLDLLEDPVEVINSKLELQAFDQIDDEIKLIGYFKGEDSEH
FKAFEEAAEHFQPYVKFFATFDKGVAKKLGLKMNEVDFYEPFMDEPVHIPDKPYSEEELVDFVREHRRATLRKLRPEDMFETWEDDMEG
IHIVAFAEEDDPDGFEFLEILKQVARDNTDNPDLSIVWIDPDDFPLLITYWEKTFKIDLFRPQIGVVNVTDADSVWMDIRDDDDLPTAE
ELEDWIEDVLSGKINTEDDDDDEDDDDDDDDDDDDDDDDDDDDDDDDDDDDD*

9.7 *Pardaliparus venustulus* (yellow-bellied tit)

From: Trinity *de novo* joint assembly of SRR7244725 and SRR7244689. CASQ1 from TRINITY_DN975_c0_g2_i3. CASQ2 from TRINITY_DN2399_c0_g1.

>CASQ1_Pardaliparus_venustulus_transcript_assembly
CGTCCCCGTGTCCGTGTGTCCGTGTGTCCATCCCCGTCCGTGTGTCCCGGTGTCTCCGTGTTCTCTTGTCCCCGTGTTCCCCTGTCCGT
GTGTCCATACCCGTGTCCCTCTGTCCCCTGTCCCTCTGTCCCCTGTCTCTCTGTCCCTCTGTCTCTCTGTCCCTCTGTCCCCGTGTCCC
TCTGTCCCGTGTCCCTCTGTCCCTCTGTCCCGTGTCCCCATGTCCCCGCTGTGGCCCTGCCGGTGGCTCCTGGCTCTGCTGGCTCTGTC
CCCGGGTGTCCCGGGGGTCTCGGGGGGTCCCGGGGGACAGGGAGGCTCCGGGAGCCCCGGGGACACCGGACACCGGCCCGAGGAGCTGG
AGGCTCTGGGGACCCCCGTGCAGGTGGTGGCAGCCGGGCAGGAGCTCCAGGCCTTTGGGGACATCGAGGAGGAGCCCAAAGTCATCGGG
TACTTCGAGGGACGGGACTCGGAGCCCTTCCAGGCCTTCTCCGCCACCGCCCGGCGCTTCCACCCCTCCCTCCTCTTCTTCGCCACCTT
CGACCCCCAGGCGGCGCAGGAGCTGCGGCTGGGGCTGAACCAGCTCCACCTCTTTGAGCCCTTCCTGGAGCAGCCCCGGAGCTTTTGGG
GGGACCCCGGGGACCCCCACGGGCTCGAGGCCTTCGTGGAGAGCAGCAAGCGGGCCACTCTGCAGAAACTCAAAGCTCAGAGCACGTCC
CAGAACTGGGAGGACCTTTGGGATGGGACCCACATCGTGGCCTTCGCCGAGGGGGACGATCCCGACGGGTTCGAGTTCCTGGAGATCCT
GAAGGAGGTGGCCCAGGACAAGAGGGACAATCCCGACTTCAGCATCCTGTGGATCGATCCCGATGATTTCCCGATGCTCGTCCCCTCCT
GGGAGGACACCTTCGACCTCGACCTGTCCCGGCCCCAGCTCGGCGTGGTCAATGGCACTGACCGTGCCGGCAGCGTGTGGCTGGACATG
GAGGATGAGGAGGATCTGCCGGGACCTGAGGAGGTCCTGGAGTGGCTCGAGGAGGTTCTGGAGGGGGACACTGGGGACAGGGATGGCGA
TGATGATGAAGACGATGACGATGACGATGAAGATGATGATGAAGACGATGACAATGAAGATGAAGATGACGATGGTGACAAAGTTGATG
ATGATGATGATGCTGACTCATAACAATGATGACGATGACGATGATGTCGTCACTGACGATGATGAAGATGATGACAATTACAATGATGA
TGATGATGATGAAGATGACAATGACAACAATGACATGACAATGACGAAGATGACGATGAAGATGATGATGACAATGA

>CASQ1_Pardaliparus_venustulus_CDS
ATGTCCCCGCTGTGGCCCTGCCGGTGGCTCCTGGCTCTGCTGGCTCTGTCCCCGGGTGTCCCGGGGGTCTCGGGGGGTCCCGGGGGACA
GGGAGGCTCCGGGAGCCCCGGGGACACCGGACACCGGCCCGAGGAGCTGGAGGCTCTGGGGACCCCCGTGCAGGTGGTGGCAGCCGGGC
AGGAGCTCCAGGCCTTTGGGGACATCGAGGAGGAGCCCAAAGTCATCGGGTACTTCGAGGGACGGGACTCGGAGCCCTTCCAGGCCTTC
TCCGCCACCGCCCGGCGCTTCCACCCCTCCCTCCTCTTCTTCGCCACCTTCGACCCCCAGGCGGCGCAGGAGCTGCGGCTGGGGCTGAA
CCAGCTCCACCTCTTTGAGCCCTTCCTGGAGCAGCCCCGGAGCTTTTGGGGGGACCCCGGGGACCCCCACGGGCTCGAGGCCTTCGTGG
AGAGCAGCAAGCGGGCCACTCTGCAGAAACTCAAAGCTCAGAGCACGTCCCAGAACTGGGAGGACCTTTGGGATGGGACCCACATCGTG
GCCTTCGCCGAGGGGGACGATCCCGACGGGTTCGAGTTCCTGGAGATCCTGAAGGAGGTGGCCCAGGACAAGAGGGACAATCCCGACTT
CAGCATCCTGTGGATCGATCCCGATGATTTCCCGATGCTCGTCCCCTCCTGGGAGGACACCTTCGACCTCGACCTGTCCCGGCCCCAGC
TCGGCGTGGTCAATGGCACTGACCGTGCCGGCAGCGTGTGGCTGGACATGGAGGATGAGGAGGATCTGCCGGGACCTGAGGAGGTCCTG
GAGTGGCTCGAGGAGGTTCTGGAGGGGGACACTGGGGACAGGGATGGCGATGATGATGAAGACGATGACGATGACGATGAAGATGATGA
TGAAGACGATGACAATGAAGATGAAGATGACGATGGTGACAAAGTTGATGATGATGATGATGCTGACTCATAA

>CASQ1_Pardaliparus_venustulus_protein
MSPLWPCRWLLALLALSPGVPGVSGGPGGQGGSGSPGDTGHRPEELEALGTPVQVVAAGQELQAFGDIEEEPKVIGYFEGRDSEPFQAF
SATARRFHPSLLFFATFDPQAAQELRLGLNQLHLFEPFLEQPRSFWGDPGDPHGLEAFVESSKRATLQKLKAQSTSQNWEDLWDGTHIV
AFAEGDDPDGFEFLEILKEVAQDKRDNPDFSILWIDPDDFPMLVPSWEDTFDLDLSRPQLGVVNGTDRAGSVWLDMEDEEDLPGPEEVL
EWLEEVLEGDTGDRDGDDDEDDDDDEDDDEDDDNEDEDDDGDKVDDDDDADS*

>CASQ2_Pardaliparus_venustulus_transcript_assembly
CCCAGGACTCACATCTTCCACTCCCTCCCTCTTGCCCACTCCCTCTCTCTCAGATGATGCAGAGTGCCAGCAAGTCCCTCAATGGTGTC

ATATCTGTCCTTTTTATTCATTGCAGGGTTTATTTTTAGCCTGAAACAACTGCTTCCTAAAAATGGAGTTCCTAATGACACGGGAGCTG
GACACAGCTATGTAAGGTATCCAAGGCTTGGCCTGACAGCTTCTCCCGTCTGTCTCTCTCTTTGTTGTGAGTACAGGACAGCAAAGTTT
GCTGCTCGCCACCAGCCTTGCAGTGTTTGGGCTCAACACTAGGAAAGCAGCAGAGAGAAACTCAGAGAAACTTAGTTCAAACTTCACCT
GGAGTCTTTGGTCTCCCTCTCTTCTCAAATTAAGAAAGAGGGGCACATTTACCCACCCTTTGCTGCACTCCAAGGGTGGCCCTATGAAG
GCAATTTGCTGGATCCTGGCAGGTTTTTACCTGCTTTTCTGCTGCAAGGCAGAAGAAGGACTGAACTTCCCTACTTATGATGGGAAAGA
CCGAGTGATCGACCTGAACGAGAAGAATTACAAGCAGGCACTGAAGAAGTATGACATGCTCTGCCTGCTCTTCCATGAGCCTGTGAGCT
CTGACAAGGTCTCCCAGAAGCAGTTCCAGATGACAGAGATGGTCCTGGAGCTGGCAGCTCAGGTCCTGGAGCCCAGGAGCATTGGCTTT
GGGATGGTGGACTCCAAGAAGGATGCCAAGCTTGCAAAGAGGTTAGGCTTGGTTGAAGAGGGAAGTCTCTATGTCTTTAAGGACGAGCG
GTTGGTTGAGTTTGATGGGGAACTGTCTGCAGATGTCTTGGTGGAATTCCTCTTGGATTTGCTAGAAGACCCCGTGGAGGTCATAAACA
GCAAGCTGGAGCTTCAGGCCTTTGACCAGATCGACGATGAAATCAAACTCATCGGCTACTTCAAGGGAGAAGACTCAGAACATTACAAG
GCATTTGAAGAAGCTGCTGAACAATTCCAGCCCTATGTCAAGTTCTTTGCCACCTTTGACAAAGGGGTTGCCAAGAAGCTGGGCCTAAA
GATGAACGAGGTGGACTTCTATGAACCCTTTATGGATGAGCCTGTTCACATCCCTGATAAGCCCTACTCCGAAGAGGAACTGGTTGATT
TTGTGAGAGAGCACAGAAGGGCCACCTTGAGGAAGCTGCGCCCAGAGGACATGTTTGAGACGTGGGAGGATGACATGGAGGGTATCCAC
ATCGTAGCCTTTGCTGAAGAAGATGACCCAGATGGTTTTGAGTTCCTGGAAATCCTGAAGCAGGTTGCCAGGGACAACACCGATAATCC
TGACCTGAGCATTGTCTGGATTGACCCTGATGACTTTCCTCTGCTCATCACTTACTGGGAGAAGACCTTCAAGATTGACCTGTTCAGAC
CACAGATCGGGGTGGTGAACGTCACGGACGCTGACAGCGTCTGGATGGACATCAGAGATGATGATGACCTGCCCACAGCCGAGGAGCTG
GAGGACTGGATAGAGGACGTGCTTTCCGGGAAGATAAATACCGAAGACGATGACGACGATGAAGATGATGACGATGACGATGATGATGA
CGACGATGACGACGACGATGACGACGACGACGATGACGATGATGACGATGACGATGACTAACTGTGACTCTGTGCAGTTTGACTTGTGG
GGCCCGAGAGCCCTTGCCCTGCGGCAGGTCCCTCCATTGGAAGACCTATGTTTGAGCATCAGCAAGCACCGCCGCTCCTCTTTCCCCCC
TGCCCCGCTCCCCCTGCCCTCGCCCGTGCCGGGACCCCCTCGCCTGATTCCCAGCGGTGCTGAGCCACCGGGACTGCCCCTGCGGAGGG
CAGCACCTGCAGGCACCCAGCACCGCTGCAAATCATGCCTCCTTAGTAAGGACAGCAGGAATCTGCAGGGAACCTGCCCCGAGTGTCCC
AGGGCAGGAGAAAAGTGCCTGCCTGCTTGCTGCCAGAGGGACGCAGGGAGCCAGTTTGTATTTTCTGTGTTCATCAGCCCAGCACTTAA
CATCCAAAGACCAAATCTCACTTCAAGACATAGGGAAAACCCTAACTGTAAAGTTTAGCCCTTTTATTAAACACAATGCCCTTTGCAGC
TACTCTGCCAAGACCACTCAATCCTGCAAGTTCTGCTGCGGGAGTCTGTAGCTACTCCCTAACGTGCTAAGAGCCAGAAAAATGCTTTG
GGAGGGGAGGGAGGTTTTAGGGATGCTATTAAAGTTTTCAGTGATTCCACATAATGACAGGATATTCTTGTCTGGCAGTGCAATACAAA
ACCAAACACCGGGTCCCCTGTCTGAACTTCTTTCTTCTCCAAAGCTCAGGCCACAGCTGGAGTCAGTGCAGGGGGCCTGATACAAGACA
CAAAGCTCGTGAGTTCCTGAACTGCTGGACAGGCTGGACCATCTCTGAGCCCAAAGTGAGTCCAAACCACTGAATCCAAAGATCATGGC
ACTGAGCCAAGCCAATGGAAATATCCATAAGGGCCCACCCTGAAGCATTCCTATTCCCCATGACCTTCCAGCTTTTGCTGACTCTTCAC
TCAAAGCCCGTGTAGCATCTTTTCCCATGATCAAGATGTTGGGAGGGAACACATCCATATTAGATAGTGGATGAGGTGCATTGGGGTGT
GAGCAGCAGACAGATCCAAGCTGTTTGCCTTTCGAGTATTTAGTTTCCTCTGTGCTTGGAGACTCAGATATCACATGTGAGAGGCCTCT
TTTGACTGCAGGCTGAGAAGAAGCTCAGGGTTTGACTAGTTTGCAGTCAAGAGGGTCACTCACAAAGGCAAGGGTCTGTACCATGGTCC
TGGCAAGTGTAGCACCATCCATGTGCAGGTCACTGTGGCAGCATTAGGCCTCCCTGCTTTCCCATGCTGGCGGATTTCAGTTGTCTTAG
CTTATCTTTGGCAATAGTGCAATGAATGGAGAGTTTGGAGATGGTTTTATCTTTATAAATAGATTTATAAAGATGTTCTCCAGTTGTTC
TGAGATGCTAGCATAGGTAGTGTACACATCTCCTCTCTTGCTATAAATGAAAAACATTGTTCCTTATACTGGCTTGGGACAGAGGTAGG
TGATGTGAGAGGAAGTAACAGTGTGAGGGACAGAGGAGTCTGAGTTATGCAAATGTGAATCAGCCTGATGAGAAACAAGGTTTACACTC
TATATATTTCCTTTATCAAATTAATTGCAAAGCAAGATTTTATAGATATTTTTTTCCTGTTTGTTTTTCAGTTTCTTTGACTCTCTCTA
CATTAGCTGAAATAGAAATTTGAAACTACTGGTATAAAATAGCTGAAGTATTGATATGTTCCCATGTGTGCCATATCCCCTTGGTGGAA
AATGCCAGCATGAACAACACATAGCTGGATTTCTCTCTGTCTCTATGCATGAATGAATGAATACTGTGCAACATGGTAGATGACTATAG
GCCAAGTTAGCTCCTTGTTGAGCATCAAACCCTCAACATGTGCTTTTCAGATACAGCCTGTTAGAAGAATAGACAAGGCAGCAAAGTCC
CCAGCTCAACCATTTGTTCTGCCACGCTTCCTTATGATGGCCTGGAAGATGAGCCAAGTCTGTTCAGGGAGAACAATCAACTGGTTGCT
CTGGGTGATGAACAACTTTGGAAAGTTTTGAAATTCAGAGTATTCTCAGCATGGAGGTCTCCTCCTCTCTTCCACTCACACCCTACTCT
TTGCTGGCCAACAGGGTGCCCAGCACTCTCCTTCTGCTTTTTCCTCAAGAGGTTTGCTCAGAGAGGGAGCGAGGAGGACTCAAAGTCTC
ATTCACCTACAGTGGTAAGGAACAAGAGGCAGAAGTATTCCCCTGGTAGTCTCCTGGCATCTGATTAAGGGACAACTACTGACCTGAGG
AAAGAACTAGCTTATCTTTACCTTTGGGCTATTCCCAGGAGCAAAACATGCACAGTTTCACCCTGTATAAGGTGCAGCCTGCACCGGCC
ATGTGCCTCCTCATCAGTCACTCCAAACATGAAGAATAATGAAAAATTCCTCTTGTGCCTTTCCTTACTGGCACAATCTCCAGATGTGC
CAATTGCTTGAGTGCTTTCTGTGTGATCCTCTCCCACTGTGGATGCCCCTCTGTTCTGAAGTGCCCCTCTCTAGATGCTCTGAGAGGCG
CAGATTACCCCAGCAGAGCAGACAGACCCTGCTGACAGCACCACGTTCAAGCACACAGGTGTTTCACAGGCACTTAAACACTTCAAAAC
AACAGACCTCTAGGGGGAAAAGAAAGAAACAATGTAGAAGAGTAGAGGCTGAACTCATACTGCAGAGTGGTGCCAGTGGCTTATGGGAA
GTTTTCCTTGAGTTATGCAAGAAAGAGCTAATCCTACAGCTGCCACTCCTTCATGGTGCAGGGATGGGGCTGCCCTTTCTCTCTTCCCT
CAGTCCACCATAACAGTTCTCATCCCTGTTTTTGCCCACAAACAGGAACTGTTTATCGTCATCTTTTGGGACAATCGCATAAAAACTTA
GGAAAGCTTTGCCACAGAGGAAGGATGGCAGCAATGGGGATGGGTGGGCAGCAAAGCCTCTCCCATTTATCTGTAACACCTTGTCTACC
CCAAGATCCTAATAACCAAACTGCTGAATGATGCAGTGTCCCTTGTGAAGGAGATGGCAGCTTGAATTTGAGTGTTTTGCTTGCAGGGA
CAGGTAAGGCAATGGTATGGAGATGACACACAGGACCCCAGTCCTCCCCTCTGAGGTGCTCCTTGGCTCCTCTGTCACAAGTAGGAACA
CCTGGCATTTGAAAGGCACTGGTGGCCCTGAGGAGCAAAGTCAGCTTAACCCTGCCTACTATTCACTGAGAAGAAACAGCACATTTGGA
TGAGGAACCTACAAAATAACCCTTTATTAAAAAAAAAATATAAGTATGTGGGCAGCACTGAGTGCTCAACAAGCACATCTCTCTTAACAA
ATAGTTCTTTTTCTCCAGCATGTTAACTAGATATATTGGAAAGGTCTTTATACAGATAAAGACAGGGATTGTATTTTGCCCTCATCCAA
ACACGAGTGCTTCAAGCTGATCAGTTTCTTCACAGAATCATCCAATCATTTTGGTTGGAAAAGACCCCCTAAGATCATTGAGTCTGACC
ATTAACCCACCACTGTCAAGTTAATACCCAAAATACTCAAAAACCTGGTCTTATCTGTCCCCAGTGATAGAGAGGACCCCACATGCAAT
ATGTGGCAAGAACACCAAGTTATGCAGCGCCTCCTCCCAGGCCCCAGACACAGGGCTGGAGGGCACAGCCGAGGGGTTTGTTTGGGGTG
CTGTACCACAGCCCATCCTGCGCTGCTCATGGGCATCAATCAAGGGAGAAGGAAATCCATTAGGAGCAACTGCAGCTCTCACACCGCAG

CATGGAGGAGAAAGTTTCTGCCTTTGCAGCAGTCGCTGATGTAGAGGCTCTGTGAACTCAAAAGTGCTCTGAGTTTATGTCAACTCTTT
GGGAAACAGCTGCAGGAGGAAAGGACAAGGGAGACACTCTGGAATTGGCAGATTAGTCTGTGTTTTCAGGGTCAGCAGTTTTTGTGATG
TTTCTGAGGAGTGATGAGTCTCCTGTCTCCCATGGGAAAGGAACAGTCCCATAGTAAAACCAGAGATCTCAGCTGCTTCTCAGCCCAGA
ATGAAGCTGCTCAGCCAGACACCAATGTGAACACAGAGCTGAGGACACCGGCTGCTTCTCCATGTTTGCCTGCATATCATACCTAAAGA
AATGACGGATTCATTACAGAGACCAAACAGAAAACATCGTCTTTTATTCCCAGGAAAATAATTAATTAGACAGGGACATATCACTTCCC
AGCTGCCACTGACTTACAGTCTTGTGAAAAGAGGACAAGACTGCATCTGGATTCCTCTCCAATGCAGCAAAGCTTTGGCTTTGAGGAAG
CCCCACACACAGCGAGTTGCACACCAATGCCTGGACTTGGGGGGCTGAAGAGGAGGATGGGCAAACTGGCAAGAAGCACCCCAGCCCCC
TGAGGCAAAGAGCTCACTGCCTCCTGGCTGCTTTCACACCCCAGGCACATCCATGCCATGCAGTGCAAGACCAGCTGCTAGAAACTGGA
AACAGCAAAGCACAGCAGGAAGGATAACTGGCCTCAGTAATGAGGCTGTGCCAGCAGGACTGTGTCCTACCTGAGACCCACTGCACTGC
AGCATCCCCTGTCTTCTCCCCACTCCCTTGTACCACTGACCTCTAGCAAAGCAGGCCTTGGATGGTCACTACCCCTGGGGACAGCCGTG
GGATGACTCTCACACCTCTTCCGGCAGAAAATGACATCTAATGACAATGGGGACCATGGGGCAGGGCCTTGGCCCAGCTCCCAGAACAT
TCAGCCAGGTTTAAAAGGTGCTAAGCTATTTAAAGGAAAAAGAAAAAAAAAGCTACAGAGCTTCCTTGCAGCTTGAAGAGCCATTTCAGG
AATCAGATTACAAACTGCCTTTGGGATGGAAGCAAAGATCCTTCCGGTGCCTCGCTCCCCCCTTGCGCTTCCTCTTGCCAAGCCCCTTG
TGATAAAGGGGATCAGTAAAGCACTCTAAGGACATTGTCCCTAATGCTATGGAAAACAAGAAAACAGCTTTTTCACCTTCTTTTCATGC
CTCTTGACTTAATACTCTTCTGACCCTCACTGGCTCCAATAATTTATTTTCTCTCTCCATTTCTATGTAGCCCATCTTCCTCTTCTTCT
CAACCCACCCCCACAGCATATGGTACTGAGGGTCACTGAAGGACAGAGCCCTTCCCTGCCCCTACTGCCCCTTCCCCAGCCCAGGGGGA
CAGCAGCCTCCTCCACACCACTGCCATGAACTGCCTGTGCTGTATCCCCACACCTGTGAACAGGAGGAGGGACAACATTTCCTTTCTGA
GGAGGAAGATGTCCAGACAAGGCTGTCACTGGCTTTTATTTTGAGGGGGACAGGTGGGACATGCTCAGGGAAAAGTCATTTCACCAGCC
TGGACAGAAGAATTTTTTGGTGGAACTCTTTTCCCACTGCCCCAAAATGTAGATGAGGAACCACCATCCTGCAACCAAGCCCTGACTGC
TGTCACTGTGGGAAGAGACCAGTAACACCCAGTGCCACCAGCAATCACACCACCACATCATCTGAGCAGAAGTGTCATGGCTTCACAT
AC-AGGAATAAACCTTGAGGGTTAAACGAGCTGAGTTTGCTGGAGGGGGTATCCCACAATCAACAGCAGGCAGAGAATCACCTCCCCA
CCTCCCTGCCAGAGACTCCCAGTGCTGTCCTGACTGAGCCCTCCCTTGGTCGTGGGTGGTAAATCACTTACATAGCCATGGGCAACCT
C

>CASQ2_Pardaliparus_venustulus_CDS
ATGAAGGCAATTTGCTGGATCCTGGCAGGTTTTTACCTGCTTTTCTGCTGCAAGGCAGAAGAAGGACTGAACTTCCCTACTTATGATGG
GAAAGACCGAGTGATCGACCTGAACGAGAAGAATTACAAGCAGGCACTGAAGAAGTATGACATGCTCTGCCTGCTCTTCCATGAGCCTG
TGAGCTCTGACAAGGTCTCCCAGAAGCAGTTCCAGATGACAGAGATGGTCCTGGAGCTGGCAGCTCAGGTCCTGGAGCCCAGGAGCATT
GGCTTTGGGATGGTGGACTCCAAGAAGGATGCCAAGCTTGCAAAGAGGTTAGGCTTGGTTGAAGAGGGAAGTCTCTATGTCTTTAAGGA
CGAGCGGTTGGTTGAGTTTGATGGGGAACTGTCTGCAGATGTCTTGGTGGAATTCCTCTTGGATTTGCTAGAAGACCCCGTGGAGGTCA
TAAACAGCAAGCTGGAGCTTCAGGCCTTTGACCAGATCGACGATGAAATCAAACTCATCGGCTACTTCAAGGGAGAAGACTCAGAACAT
TACAAGGCATTTGAAGAAGCTGCTGAACAATTCCAGCCCTATGTCAAGTTCTTTGCCACCTTTGACAAAGGGGTTGCCAAGAAGCTGGG
CCTAAAGATGAACGAGGTGGACTTCTATGAACCCTTTATGGATGAGCCTGTTCACATCCCTGATAAGCCCTACTCCGAAGAGGAACTGG
TTGATTTTGTGAGAGAGCACAGAAGGGCCACCTTGAGGAAGCTGCGCCCAGAGGACATGTTTGAGACGTGGGAGGATGACATGGAGGGT
ATCCACATCGTAGCCTTTGCTGAAGAAGATGACCCAGATGGTTTTGAGTTCCTGGAAATCCTGAAGCAGGTTGCCAGGGACAACACCGA
TAATCCTGACCTGAGCATTGTCTGGATTGACCCTGATGACTTTCCTCTGCTCATCACTTACTGGGAGAAGACCTTCAAGATTGACCTGT
TCAGACCACAGATCGGGGTGGTGAACGTCACGGACGCTGACAGCGTCTGGATGGACATCAGAGATGATGATGACCTGCCCACAGCCGAG
GAGCTGGAGGACTGGATAGAGGACGTGCTTTCCGGGAAGATAAATACCGAAGACGATGACGACGATGAAGATGATGACGATGACGATGA
TGATGACGACGATGACGACGACGATGACGACGACGACGATGACGATGATGACGATGACGATGACTAA

>CASQ2_Pardaliparus_venustulus_protein
MKAICWILAGFYLLFCCKA-EEGLNFPTYDGKDRVIDLNEKNYKQALKKYDMLCLLFHEPVSSDKVSQKQFQMTEMVLELAAQVLEPRS
IGFGMVDSKKDAKLAKRLGLVEEGSLYVFKDERLVEFDGELSADVLVEFLLDLLEDPVEVINSKLELQAFDQIDDEIKLIGYFKGEDSE
HYKAFEEAAEQFQPYVKFFATFDKGVAKKLGLKMNEVDFYEPFMDEPVHIPDKPYSEEELVDFVREHRRATLRKLRPEDMFETWEDDME
GIHIVAFAEEDDPDGFEFLEILKQVARDNTDNPDLSIVWIDPDDFPLLITYWEKTFKIDLFRPQIGVVNVTDADSVWMDIRDDDDLPTA
EELEDWIEDVLSGKINTEDDDDDEDDDDDDDDDDDDDDDDDDDDDDDDDDD*

9.8 *Passer montanus* (Eurasian tree sparrow)

From: Trinity *de novo* assembly of SRR18552105. No CASQ1 found. CASQ2 from TRINITY_DN4804_c0_g1_i6.

>CASQ2_Passer_montanus_transcript_assembly
CTCTCTTTGTTGTGAGTACAGGACAGCAAAGTTTACTGCTCCCCACCAGCCTTGCAGTGTTTGGGCTCAACTCTAGGAAAGCAGCAGAG
AGAAACTTAGAGAAACTAAGAGAAACTTAGTTCAAACTTCACCTGGAGTCTTTGGTCTCCCTCTCTTCTCAAATTAAGAAACAGGGGGA
CATTTACCCACCCTTTGCTGCACTCCAAGGGTGCCCCTATGAAGGCAATTTGCTGGATCCTGGCAGGTTTTTACCTGCTTTTCTGCTGC
AAGGCAGAAGAGGGACTGAACTTCCCTACTTACGATGGTAAAGACCGAGTGATCGACCTCAACGAGAAGAATTACAAGCAGGCACTGAA
GAAGTATGACATGCTCTGCCTGCTCTTCCACGAGCCTGTGAGCTCTGACAAGGTCTCCCAGAAGCAATTCCAGATGACAGAGATGGTCC
TGGAGCTGGCAGCTCAGGTCCTGGAGCCCAGGAGCATTGGCTTTGGGATGGTGGACTCCAAGAAGGATGCCAAGCTTGCCAAAAAATTA
GGCTTGCTTGAAGAGGGAAGTCTCTATGTTTTTAAGGATGAGCGGTTGGTTGAATTTGATGGAGAACTGTCTGCAGATGTCTTGGTGGA
ATTCCTCTTGGATTTGCTGGAAGACCCCGTGGAGGTCATCAACAGCAAGCTGGAGCTTCAGGCCTTTGACCAGATCGACGACGAAATCA
AACTCATCGGCTACTTCAAGGGAGAAGACTCAGAACATTTCAAGGCATTTGAAGAAGCTGCTGAACATTTCCAGCCCTATGTCAAGTTC
TTTGCCACCTTTGATAAAGGGGTTGCCAAGAAGCTAGGCCTAAAGATGAACGAGGTGGACTTCTACGAACCCTTTATGGATGAGCCTGT

TCACATCCCTGATAAGCCTTACTCAGAAGAGGAGCTGGTTGATTTTGTGAGAGAGCACAGAAGGGCCACCTTGAGGAAGCTGCGCCCAG
AGGACATGTTTGAGACATGGGAGGATGACATGGAGGGTATTCACATCGTAGCCTTTGCTGAAGAAGATGACCCAGATGGTTTTGAGTTC
CTGGAAATCCTGAAGCAGGTTGCCAGGGACAACACTGATAATCCCGACCTGAGCATTGTCTGGATTGACCCTGACGACTTTCCTTTGCT
CATCACTTACTGGGAGAAGACCTTCAAGATCGACCTGTTCAGACCACAGATCGGGGTGGTGAACGTCACGGACGCTGACAGCGTCTGGA
TGGACATCAGAGATGATGATGACCTGCCCACAGCCGAGGAGCTGGAGGACTGGATAGAGGACGTGCTTTCCGGGAAGATAAATACCGAA
GATGATGATGATGATGAAGATGACGACGATGATGATGACGACGATGATGATGACGACGACGATGATGACGACGACGATGACGATGATGA
CGATGATGACGACTAACTGTGACTCTGTGCAGTTTGACTTGTGGGGCCCGAGAGCCCTGGCCCTGCGGCAGGTCCCTCCATTTGAAGAC
CTATGTTTGAGCATCAGCGAGCACCGCTGCTCCTCTTTATCCCCCGGCCCCGCTCCCCCTGCCCTCGCCCTTGCCGGGAGCCCCTTGCC
TGATTCCCAGCGGTGCTGAGCCAGCGGGACTGCCCCTGCCCTGCGGAGGCAGCACCTGCAGGCACCCAGCACCGCTGCAAATCATGCCT
CCTTAGTAAGGACAGTAGGAATCTGCAGGGAATCTGCCCCGAGTGTCCCAGGGCAGGAGAAAAGTGCCTGCCTGCTTGCTGCCAGAGGG
ACGCAGGGAGCCAGTTTGTATTTTCTGTGTTCATCAGCCCAGCACTTAACATCCAAAGACCAAATCTCACTTCAAGACATAGGGAAAAC
CCTAACTGTAAAGTTTAGCCCTTTTATTAAACACAATGCCCTTTGCAGCTACTCTGCCAAGACCACTCAATCCTGCAAGTTCTGCTGCG
GGAGTCTGTAGCTACTCCCTAACATGCTAAGAGCCAGAAAAATGCTTTGGGAGGGGAGGGAGGTTTAGGGATGCTATTAAAGTTTTCA
GTGATTCCACATAATGACAGGATATTCTTGACTGGCAGTGCAATGCAAAACCAAACACCGGGTCCCCTGTCTGAATCTCTTTCTTCTTG
AAGGCTGAGGCCACAGCTGGAGTCAGTGCAGGGACCCTGATATGAGACACAAAGCTCTTGAGTTCCTGAACTGCCGGACAGGCTGGTCC
AGCTCGGAGCCCAATGTGAGTACAAACCACTGAGTCTGAAGATCATGGCACTGAGCCAAGCCAATGGAAACATCCACAGGAGCCCACCC
TGAAGCACTCCTATTCCCCATGACCTTTTGCTGACTCTTCACCCAAAGCCAATGTAGCATCTTTTCCCATGATCAAGATGTCAGGAGGG
AACACGTCCATCCAAACATATTAGATAGTGGATGAGCTGCATTGGGGTGTGAGCAGCTGAGGGATTCAAGCTGTTTGGCTTTTGAGTAT
TTAGTTGCAGTTACTCCCTGCTTAGAGACTCAGATATCCCATGTGAGAAGCCCCTTTTGGCTGCAGGCTGAGAAGGAGTTCAGGGCACC
AGTTTGCAGTCAAGGGGGTCACTCACAAAGCCCAGGGTCCGTTCCTGGGTCCTGGCGAGTGCAGCAGCATCCATGTGCAGGTCACTGTG
GCAGCATTAGGTCTCCCTGCTTTCCCATGCTGGCAGATTTCAATTGTCTTAGCTTCTTATTAGTTTGGCAATAGTGCAATGAATGGAG
AGTTCAGAGATGGTGTTATCTTTATAAATACATTTATAAAGATGTTCTGCAGTTGTTCAGAGTTGCTAGCATAGGTGGTGTACACATT
TCTCTTGCTACAAACAAAAAAACATTGTTCTGACCATCCTTATACTGGCTTCGGACAGAGGTACGTGATGTGAGGGGAAATAAAAGTGT
GAGGGACAGAAGAATCAGTTACACAAATGCGAATCTTGATGAGAAACAAGGTCTACACACTATATATTTCCTTTATCAGATGAACTGC
AAAGCAAGATTTTATAGATTTTTTTCCTGCCTGTTGATCAGTTTCTTTGACCCCCTCTCTCTACATTAGCTGAAATAGAAACTTGAA
A

>CASQ2_Passer_montanus_CDS
ATGAAGGCAATTTGCTGGATCCTGGCAGGTTTTTACCTGCTTTTCTGCTGCAAGGCAGAAGAGGGACTGAACTTCCCTACTTACGATGG
TAAAGACCGAGTGATCGACCTCAACGAGAAGAATTACAAGCAGGCACTGAAGAAGTATGACATGCTCTGCCTGCTCTTCCACGAGCCTG
TGAGCTCTGACAAGGTCTCCCAGAAGCAATTCCAGATGACAGAGATGGTCCTGGAGCTGGCAGCTCAGGTCCTGGAGCCCAGGAGCATT
GGCTTTGGGATGGTGGACTCCAAGAAGGATGCCAAGCTTGCCAAAAAATTAGGCTTGCTTGAAGAGGGAAGTCTCTATGTTTTTAAGGA
TGAGCGGTTGGTTGAATTTGATGGAGAACTGTCTGCAGATGTCTTGGTGGAATTCCTCTTGGATTTGCTGGAAGACCCCGTGGAGGTCA
TCAACAGCAAGCTGGAGCTTCAGGCCTTTGACCAGATCGACGACGAAATCAAACTCATCGGCTACTTCAAGGGAGAAGACTCAGAACAT
TTCAAGGCATTTGAAGAAGCTGCTGAACATTTCCAGCCCTATGTCAAGTTCTTTGCCACCTTTGATAAAGGGGTTGCCAAGAAGCTAGG
CCTAAAGATGAACGAGGTGGACTTCTACGAACCCTTTATGGATGAGCCTGTTCACATCCCTGATAAGCCTTACTCAGAAGAGGAGCTGG
TTGATTTTGTGAGAGAGCACAGAAGGGCCACCTTGAGGAAGCTGCGCCCAGAGGACATGTTTGAGACATGGGAGGATGACATGGAGGGT
ATTCACATCGTAGCCTTTGCTGAAGAAGATGACCCAGATGGTTTTGAGTTCCTGGAAATCCTGAAGCAGGTTGCCAGGGACAACACTGA
TAATCCCGACCTGAGCATTGTCTGGATTGACCCTGACGACTTTCCTTTGCTCATCACTTACTGGGAGAAGACCTTCAAGATCGACCTGT
TCAGACCACAGATCGGGGTGGTGAACGTCACGGACGCTGACAGCGTCTGGATGGACATCAGAGATGATGATGACCTGCCCACAGCCGAG
GAGCTGGAGGACTGGATAGAGGACGTGCTTTCCGGGAAGATAAATACCGAAGATGATGATGATGATGAAGATGACGACGATGATGATGA
CGACGATGATGATGACGACGACGATGATGACGACGACGATGACGATGATGACGATGATGACGACTAA

>CASQ2_Passer_montanus_protein
MKAICWILAGFYLLFCCKAEEGLNFPTYDGKDRVIDLNEKNYKQALKKYDMLCLLFHEPVSSDKVSQKQFQMTEMVLELAAQVLEPRSI
GFGMVDSKKDAKLAKKLGLLEEGSLYVFKDERLVEFDGELSADVLVEFLLDLLEDPVEVINSKLELQAFDQIDDEIKLIGYFKGEDSEH
FKAFEEAAEHFQPYVKFFATFDKGVAKKLGLKMNEVDFYEPFMDEPVHIPDKPYSEEELVDFVREHRRATLRKLRPEDMFETWEDDMEG
IHIVAFAEEDDPDGFEFLEILKQVARDNTDNPDLSIVWIDPDDFPLLITYWEKTFKIDLFRPQIGVVNVTDADSVWMDIRDDDDLPTAE
ELEDWIEDVLSGKINTEDDDDDEDDDDDDDDDDDDDDDDDDDDDDDDDDD*

9.9 *Periparus rubidiventris* (rufous-vented tit)

From trinity *de novo* assembly of SRR7244645. CASQ1 from TRINITY_DN2383_c0_g1_i16. CASQ2 from TRINITY_DN4020_c2_g1_i1.

>CASQ1_Periparus_rubidiventris_transcript_assembly
CCATGTCCCCGCTGTGGCCCTGCCGGTGGCTCCTGGCTCTGCTGGCTCTGTCCCCGGGGGTCCCGGGGGTCTCGGGGGGTCCTGGGGGG
CAGGGAGGCTCCGGGAGCCCCCCCGAGCGCCCCGGGGACACCGGACACCGGCCCGAGGAGCTGGAGGAGCTGGAGGCTCTGGGGACCCC
CGTGCAGGTGGTGGCAGCCGGGCAGGAGCTCCAGGCCTTTGGGGACATCGAGGAGGAGCCCAAAGTCATCGGGTACTTCGAGGGACGGG
ACTCGGAGCCCTTCCAGGCCTTCTCCGCCACCGCCCAGCGCTTCCACCCCTCCCTCCTCTTCTTCGCCACCTTCGACCCCCAGGCGGCG
CAGGAGCTGCGGCTGGGGCTGAACCACCTCCATCTCTTCGAGCCCTTCCTGGAGCAGCCCCGGAGCTTTCGGGGGGACCCCGGGGACCC
CCAAGGGCTCGAGGCCTTCGTGGAGAGAAACAAGCGGGCCACTCTGCAGAAACTCAAAGCTCAGAGCACGTCCCAGAACTGGGAGGACC

TTTGGGATGGGACCCACATCGTTGCCTTCGCCGAGGGGGATGATCCCGACGGGTTCGAGTTCCTGGAGATCCTGAAGGAGGTGGCCCAG
GACAAGAGGGACAATCCCGACTTCAGCATCCTGTGGATCGATCCCGATGATTTCCCGATGCTCGTCCCCTCCTGGGAGGACACCTTTGA
CCTCGACCTGTCCCGGCCTCAGCTCGGCGTGGTCAATGGCACTGATCGTGCCGGCAGCGTGTGGCTGGACATGGAGGATGGCGATGATG
ATGACGATGATGATGAAGATGAAGACGATGACGATGAAGATGAAGACGACGATGGTGACAAAGTTGATGACGATGATGATGCTGACTCA
TAACAATGATGACGATGACGATGATGTCGTCACTGACGATGATGATGATGACAATTACAATGACAATGATGATGATGATGATGACGACA
GTGACATGACAATGATGAAGATGACGATGAAGATGATGATGACAATGACAACACTGACAATGATGAAGATGATGACAATTACAATGACA
ATGATGATGATGATGATGACGACAGTGACATGACAATGATGAAGATGACGATGAAGATGATGATGACAATGACAACACTGACAATGATG
AAGATGATGACAATTACAATGACAATGATGACGATGATGATGGTGGTGATGATGACGATGGTGACAATGACAACACTGACAATGATGAA
GATGATGACAATTACAATGACAATGATGACGATGATGATGGTGGTGATGATGACGATGGTGACAATGATGATGAAAACAATGACGATGC
CAATGATGATGAAGTCGATGATGATGATGATGATGATGATGATGATGATGATGACGATGGTGACAATGATGATGAAAACAATGACGATG
CCAATGATGATGAAGTCGATGATGATGATGATGATGATG

>CASQ1_Periparus_rubidiventris_CDS
ATGTCCCCGCTGTGGCCCTGCCGGTGGCTCCTGGCTCTGCTGGCTCTGTCCCCGGGGGTCCCGGGGGTCTCGGGGGGTCCTGGGGGGCA
GGGAGGCTCCGGGAGCCCCCCCGAGCGCCCCGGGGACACCGGACACCGGCCCGAGGAGCTGGAGGAGCTGGAGGCTCTGGGGACCCCCG
TGCAGGTGGTGGCAGCCGGGCAGGAGCTCCAGGCCTTTGGGGACATCGAGGAGGAGCCCAAAGTCATCGGGTACTTCGAGGGACGGGAC
TCGGAGCCCTTCCAGGCCTTCTCCGCCACCGCCCAGCGCTTCCACCCCTCCCTCCTCTTCTTCGCCACCTTCGACCCCCAGGCGGCGCA
GGAGCTGCGGCTGGGGCTGAACCACCTCCATCTCTTCGAGCCCTTCCTGGAGCAGCCCCGGAGCTTTCGGGGGGACCCCGGGGACCCCC
AAGGGCTCGAGGCCTTCGTGGAGAGAAACAAGCGGGCCACTCTGCAGAAACTCAAAGCTCAGAGCACGTCCCAGAACTGGGAGGACCTT
TGGGATGGGACCCACATCGTTGCCTTCGCCGAGGGGGATGATCCCGACGGGTTCGAGTTCCTGGAGATCCTGAAGGAGGTGGCCCAGGA
CAAGAGGGACAATCCCGACTTCAGCATCCTGTGGATCGATCCCGATGATTTCCCGATGCTCGTCCCCTCCTGGGAGGACACCTTTGACC
TCGACCTGTCCCGGCCTCAGCTCGGCGTGGTCAATGGCACTGATCGTGCCGGCAGCGTGTGGCTGGACATGGAGGATGGCGATGATGAT
GACGATGATGATGAAGATGAAGACGATGACGATGAAGATGAAGACGACGATGGTGACAAAGTTGATGACGATGATGATGCTGACTCATA
A

>CASQ1_Periparus_rubidiventris_protein
MSPLWPCRWLLALLALSPGVPGVSGGPGGQGGSGSPPERPGDTGHRPEELEELEALGTPVQVVAAGQELQAFGDIEEEPKVIGYFEGRD
SEPFQAFSATAQRFHPSLLFFATFDPQAAQELRLGLNHLHLFEPFLEQPRSFRGDPGDPQGLEAFVERNKRATLQKLKAQSTSQNWEDL
WDGTHIVAFAEGDDPDGFEFLEILKEVAQDKRDNPDFSILWIDPDDFPMLVPSWEDTFDLDLSRPQLGVVNGTDRAGSVWLDMEDGDDD
DDDDEDEDDDDEDEDDDGDKVDDDDDADS*

>CASQ2_Periparus_rubidiventris_transcript_assembly
-TAAAAATGGAGTTCCTAATGACACGGGAGCTGGACACAGCTATGTAAGGTATCCAAGGCTTGGCCTGACAGCTTCTCCCGTCTGTCTC
TCTCTTTGTTGTGAGTACAGGACAGCAAAGTTTGCTGCTCGCCACCAGCCTTGCAGTGTTTGGGCTCAACACTAGGAAAGCAGCAGAGA
GAAACTCAGAGAAACTTAGTTCAAACTTCACCTGGAGTCTTTGGTCTCCCTCTCTTCTCAAATTACGAAAGAGGGGCACATTTACCCAC
CCTTTGCTGCACTCCAAGGGTGGCCCTATGAAGGCAATTTGCTGGATCCTGGCAGGTTTTTACCTGCTTTTCTGCTGCAAGGCAGAAGA
AGGACTGAACTTCCCTACTTATGATGGGAAAGACCGAGTGATCGACCTGAACGAGAAGAATTACAAGCAGGCACTGAAGAAGTATGACA
TGCTCTGCCTGCTCTTCCATGAGCCTGTGAGCTCTGACAAGGTCTCCCAGAAGCAGTTCCAGATGACAGAGATGGTCCTGGAGCTGGCA
GCTCAGGTCCTGGAGCCCAGGAGCATTGGCTTTGGGATGGTGGACTCCAAGAAGGATGCCAAGCTTGCCAAGAAGTTAGGCTTGGTTGA
AGAGGGAAGTCTCTATGTCTTTAAGGACGAGCGGTTGGTTGAGTTTGATGGGGAACTGTCTGCAGATGTCTTGGTGGAATTCCTCTTGG
ATTTGCTAGAAGACCCCGTGGAGGTCATAAACAGCAAGCTGGAGCTTCAGGCCTTTGACCAGATCGACGACGAAATCAAACTCATCGGC
TACTTCAAGGGAGAAGACTCAGAACATTACAAGGCATTTGAAGAAGCTGCTGAACAATTCCAGCCCTATGTCAAGTTCTTTGCCACCTT
TGACAAAGGGGTTGCCAAGAAGCTAGGCCTAAAGATGAATGAGGTGGACTTCTATGAACCCTTTATGGATGAGCCTGTTCACATCCCCG
ATAAGCCCTACTCTGAAGAGGAGCTGGTTGATTTTGTGAGAGAGCACAGAAGGGCCACCTTGAGGAAGCTGCGCCCAGAGGACATGTTT
GAGACGTGGGAGGATGACATGGAGGGTATCCACATCGTAGCCTTTGCTGAAGAAGATGACCCAGATGGTTTTGAGTTCCTGGAAATCCT
GAAGCAGGTTGCCAGGGACAACACCGATAATCCTGACCTGAGCATTGTCTGGATTGACCCTGATGACTTTCCTCTGCTCATCACTTACT
GGGAGAAGACCTTCAAGATTGACCTGTTCAGACCACAGATCGGGGTGGTGAACGTCACGGACGCTGACAGCGTCTGGATGGACATCAGA
GATGATGATGACCTGCCCACAGCCGAGGAGCTGGAGGACTGGATAGAGGACGTGCTTTCCGGGAAGATAAATACCGAAGACGATGACGA
TGATGAAGATGATGACGATGATGATGATGATGACGACGACGATGACGACGATGACGACGACGACGATGACGATGATGACGATGACGATG
ACTAACTGTGACTCTGTGCAGTTTGACTTGTGGGGCCCGAGAGCCCTTGCCCTGCGGCAGGTCCCTCCATTGGAAGACCTATGTTTGAG
CATCAGCAAGCACCGCCGCTCCTCTTTCCCCCCTGCCCCGCTCCCCCTGCCCTCGCCCGTGCCGGGACCCCCTCGCCTGATTCCCAGCG
GTGCTGAGCCACCGGGACTGCCTCTGCGGAGGGCAGCACCTGCAGGCACCCAGCACCGCTGCAAATCACGCCTCCTTAGTAAGGACAGC
AGGAATCTGCAGGGAACCTGCCCCGAGTGTCCCAGGGCAGGAGAAAAGTGCCTGCCTGCTTGCTGCCAGAGGGACGCAGGGAGCCAGTT
TGTATTTTCTGTGTTCATCAGCCCAGCACTTAACATCCAAAGACCAAATCTCACTTCAAGACATAGGGAAAACCCTAACTGTAAAGTTT
AGCCCTTTTATTAAACACAATGCCCTTTGCAGCTACTCTGCCAAGACCACTCAATCCTGCAAGTTCTGCTGCGGGAGTCTGTAGCTACT
CCCTAACGTGCTAAGAGCCAGAAAAATGCTTTGGGAGGGGAGGGAGGTTTTAGGGATGCTATTAAAGTTTTCAGTGATTCCACATAATG
ACAGGATATTCTTGTCTGGCAGTGCAATACAAAACCAAATACCGGGTCCCCTGTCTGAACTTCTTTCTTCTCCAAAGCTCAGGCCACAG
CTGGAGTCAGTGCAGGGGGCCTGATACAAGACACAAAGCTCATGAGTTCCTGAACTGCTGGACAGGCTGGACCAGCTCTGAGCCCAAAG
TGAGTCCAAACCACTGAATCCAAAGATCATGGCACTGAGCCAAGCCAGTGGAAATATCCATAAGGGCCCACCCTGAAGCATTCCTATTC
CCCATGACCTTCCAGCTTTTGCTGACTCTTCAAAGCCCGTGTAGCATCTTTTCCCATGATCAAGATGTTGGGAGGGAACACATCCATAT
TAGATAGTGGATGAGGTGCATTGGGGTGTGAGCAGCAGACAGATCCAAGCTGTTTGCCTTTCGAGTATTTAGTTCCTCTGTGCTTGGA

GACTCAGATATCCCATGTGAGAGGCCTCTTTTGACTGCAGGCTGAGAAGAAGCTCAGGGTTTGACTAGTTTGCAGTCAAGAGGGTCACT
CACAAAGGCAAGGGTCTGTACCATGGTCCTGGCAAGTGTAGCACCATCCATGTGCAGGTCACTGTGGCAGCATTAGGCCTCCCTGCTTT
CCCATGCTGGCAGATTTCAGTTGTCTTAGCTTATCTTTGGCAATAGTGCAATGAATGGAGAGTTTGGAGATGGTTTTATCTTTATAAAT
AGATTTATAAAGATGTTCTCCAGTTGTTCTGAGATGCTAGCATAGGTAGTGTACACATCTCCTCTCTTGCTATAAATAAAAAACATTGT
TCCTTATACTGGCTTGGGACAGAGGTAGGTGATGTGAGAGGAAGTAACAGTGTGAGGGACAGAGAAGTCTGAGTTATGCAAATGTGAAT
CAGCCTGATGAGAAACAAGGTTTACACTCTATATATTTCCTTTATCAGATTAATTGCAAAGCAAGATTTTATAGATATTTTTTTCCTGA
TTGTTTTTCAGTTTCTTTGACTCTCTCTACATTAGCTGAAATAGAAATTTGAAACTACTGGTATAAAATAGCTGAAGTATTGATATGTT
CCCATGTGTGCCATATCCCCTTGGTGGAAAATGCCAGCATGAACAACACATAGCTGGATTTCTCTCTGTCTCTATGCATGAATGAATGA
ATACTGTGCAACATGGTAGATG

>CASQ2_Periparus_rubidiventris_CDS
ATGAAGGCAATTTGCTGGATCCTGGCAGGTTTTTACCTGCTTTTCTGCTGCAAGGCAGAAGAAGGACTGAACTTCCCTACTTATGATGG
GAAAGACCGAGTGATCGACCTGAACGAGAAGAATTACAAGCAGGCACTGAAGAAGTATGACATGCTCTGCCTGCTCTTCCATGAGCCTG
TGAGCTCTGACAAGGTCTCCCAGAAGCAGTTCCAGATGACAGAGATGGTCCTGGAGCTGGCAGCTCAGGTCCTGGAGCCCAGGAGCATT
GGCTTTGGGATGGTGGACTCCAAGAAGGATGCCAAGCTTGCCAAGAAGTTAGGCTTGGTTGAAGAGGGAAGTCTCTATGTCTTTAAGGA
CGAGCGGTTGGTTGAGTTTGATGGGGAACTGTCTGCAGATGTCTTGGTGGAATTCCTCTTGGATTTGCTAGAAGACCCCGTGGAGGTCA
TAAACAGCAAGCTGGAGCTTCAGGCCTTTGACCAGATCGACGACGAAATCAAACTCATCGGCTACTTCAAGGGAGAAGACTCAGAACAT
TACAAGGCATTTGAAGAAGCTGCTGAACAATTCCAGCCCTATGTCAAGTTCTTTGCCACCTTTGACAAAGGGGTTGCCAAGAAGCTAGG
CCTAAAGATGAATGAGGTGGACTTCTATGAACCCTTTATGGATGAGCCTGTTCACATCCCCGATAAGCCCTACTCTGAAGAGGAGCTGG
TTGATTTTGTGAGAGAGCACAGAAGGGCCACCTTGAGGAAGCTGCGCCCAGAGGACATGTTTGAGACGTGGGAGGATGACATGGAGGGT
ATCCACATCGTAGCCTTTGCTGAAGAAGATGACCCAGATGGTTTTGAGTTCCTGGAAATCCTGAAGCAGGTTGCCAGGGACAACACCGA
TAATCCTGACCTGAGCATTGTCTGGATTGACCCTGATGACTTTCCTCTGCTCATCACTTACTGGGAGAAGACCTTCAAGATTGACCTGT
TCAGACCACAGATCGGGGTGGTGAACGTCACGGACGCTGACAGCGTCTGGATGGACATCAGAGATGATGATGACCTGCCCACAGCCGAG
GAGCTGGAGGACTGGATAGAGGACGTGCTTTCCGGGAAGATAAATACCGAAGACGATGACGATGATGAAGATGATGACGATGATGATGA
TGATGACGACGACGATGACGACGATGACGACGACGACGATGACGATGATGACGATGACGATGACTAA

>CASQ2_Periparus_rubidiventris_protein
MKAICWILAGFYLLFCCKAEEGLNFPTYDGKDRVIDLNEKNYKQALKKYDMLCLLFHEPVSSDKVSQKQFQMTEMVLELAAQVLEPRSI
GFGMVDSKKDAKLAKKLGLVEEGSLYVFKDERLVEFDGELSADVLVEFLLDLLEDPVEVINSKLELQAFDQIDDEIKLIGYFKGEDSEH
YKAFEEAAEQFQPYVKFFATFDKGVAKKLGLKMNEVDFYEPFMDEPVHIPDKPYSEEELVDFVREHRRATLRKLRPEDMFETWEDDMEG
IHIVAFAEEDDPDGFEFLEILKQVARDNTDNPDLSIVWIDPDDFPLLITYWEKTFKIDLFRPQIGVVNVTDADSVWMDIRDDDDLPTAE
ELEDWIEDVLSGKINTEDDDDDEDDDDDDDDDDDDDDDDDDDDDDDDDDD*

9.10 *Poecile palustris* (marsh tit)

From: Trinity *de novo* assembly of SRR7244681. CASQ1 from the complement of TRINITY_DN5_c3_g1_i1. CASQ3 from the complement of TRINITY_DN6101_c0_g1_i1.

>CASQ1_Poecile_palustris_transcript_assembly
CTGCCGGTGGCTCCTGGCTCTGCTGGCTCTGTCCCCGGGTGTCCCGGGGGTCCCGGGGGTCTCGGGGGGTCCGGGGGGGCCGCTGCTGC
CGGTGAGCTCCGGGAGCCCCCCCGAGCGCCCCGGGGACACCGGACCCCGGCCCGAGGAGCTGGAGGCGCTGGAGGCTCTGGGGACCCCC
GTGCAGGTGGTGGCAGCCGGGCAGGAGCTCCAGGCCTTTGGGGACATCGAGGAGGAGCCCAAAGTCATCGGGTACTTCGAGGGACGGGA
CTCGGAGCCCTTCCAGGCCTTCTCCGCCACCGCCCGGCGCTTCCACCCCTCCCTCCTCTTCTTCGCCACCTTCGACCCCCAGGCGGCGC
AGGAGCTGCGGCTGGGGCTGAACCAGCTCCACCTCTTCGAGCCCTTCCTGGAGCAGCCCCGGAGCTTTTGGGGGGACCCCGGGGACCCC
CACGGGATCGAGGCCTTCGTGGAGAGCAGCAAACGGGCCACTCTGCAGAAACTCAAAGCTCAGAGCACGTCCCAGAACTGGGAGGACCT
TTGGGATGGGACCCACATCGTGGCCTTCGCCGAGGGGGATGATCCCGACGGGTTCGAGTTCCTGGAGATCCTGAAGGAGGTGGCCCGGG
ACAAGAGGGACAATCCCGAGTTCAGAATCCTGTGGATCGATCCCGATGATTTCCCGATGCTCGTCCCCTCCTGGGAGGACACCTTCGAC
ATCGACCTGTCCCGGCCCCAGCTCGGCGTGGTCAATGGCACCGACCATGCCAGCAGCGTGTGGCTGGACATGGAGGATGAGGAGGATCT
GCCGGGACCTGAGGAGGTCCTGGAGTGGCTCGAGGAGGTTCTGGAGGGGGACACTGGGGACAGGGATGGCGATGACGATGAAGATGATG
ATGATGAAGATGATGATGATGAAGATGATGATGATGATGAAGATGATGATGATGA

>CASQ1_Poecile_palustris_CDS_partial
CTGCCGGTGGCTCCTGGCTCTGCTGGCTCTGTCCCCGGGTGTCCCGGGGGTCCCGGGGGTCTCGGGGGGTCCGGGGGGGCCGCTGCTGC
CGGTGAGCTCCGGGAGCCCCCCCGAGCGCCCCGGGGACACCGGACCCCGGCCCGAGGAGCTGGAGGCGCTGGAGGCTCTGGGGACCCCC
GTGCAGGTGGTGGCAGCCGGGCAGGAGCTCCAGGCCTTTGGGGACATCGAGGAGGAGCCCAAAGTCATCGGGTACTTCGAGGGACGGGA
CTCGGAGCCCTTCCAGGCCTTCTCCGCCACCGCCCGGCGCTTCCACCCCTCCCTCCTCTTCTTCGCCACCTTCGACCCCCAGGCGGCGC
AGGAGCTGCGGCTGGGGCTGAACCAGCTCCACCTCTTCGAGCCCTTCCTGGAGCAGCCCCGGAGCTTTTGGGGGGACCCCGGGGACCCC
CACGGGATCGAGGCCTTCGTGGAGAGCAGCAAACGGGCCACTCTGCAGAAACTCAAAGCTCAGAGCACGTCCCAGAACTGGGAGGACCT
TTGGGATGGGACCCACATCGTGGCCTTCGCCGAGGGGGATGATCCCGACGGGTTCGAGTTCCTGGAGATCCTGAAGGAGGTGGCCCGGG
ACAAGAGGGACAATCCCGAGTTCAGAATCCTGTGGATCGATCCCGATGATTTCCCGATGCTCGTCCCCTCCTGGGAGGACACCTTCGAC
ATCGACCTGTCCCGGCCCCAGCTCGGCGTGGTCAATGGCACCGACCATGCCAGCAGCGTGTGGCTGGACATGGAGGATGAGGAGGATCT
GCCGGGACCTGAGGAGGTCCTGGAGTGGCTCGAGGAGGTTCTGGAGGGGGACACTGGGGACAGGGATGGCGATGACGATGAAGATGATG

ATGATGAAGATGATGATGATGAAGATGATGATGATGATGAAGATGATGATGAT

>CASQ1_Poecile_palustris_protein_partial
XCRWLLALLALSPGVPGVPGVSGGPGGPLLPVSSGSPPERPGDTGPRPEELEALEALGTPVQVVAAGQELQAFGDIEEEPKVIGYFEGR
DSEPFQAFSATARRFHPSLLFFATFDPQAAQELRLGLNQLHLFEPFLEQPRSFWGDPGDPHGIEAFVESSKRATLQKLKAQSTSQNWED
LWDGTHIVAFAEGDDPDGFEFLEILKEVARDKRDNPEFRILWIDPDDFPMLVPSWEDTFDIDLSRPQLGVVNGTDHASSVWLDMEDEED
LPGPEEVLEWLEEVLEGDTGDRDGDDDEDDDDEDDDDEDDDDDEDDDDX

>CASQ2_Poecile_palustris_transcript_assembly
GACACAGCTATGTAAGGTATCCAGGGCTTGGCCTGACAGCTTCTCCCGTCTGTCTCTCTCTTTGTTGTGAGTACAGGACAGCAAAGTTT
GCTGCTCGCCACCAGCCTTGCAGTGTTTGGGCTCAACACTAGGAAAGCAGCAGATAGAAACTCAGAGAAACTTAGTTCAAACTTCACCT
GGAGTCTTTGATCTCCCTCTCTTCTCAAATTAAGAGAGAGGGGCACATTTACCCACCCTTTGCTGCACTCCAAGGGTGGCCCTATGAAG
GCAATTTGCTGGATCCTGGCAGGTTTTTACCTGCTTTTCTGCTGCAAGGCAGAAGAAGGACTGAACTTCCCTACTTATGATGGGAAAGA
CCGAGTGATCGACCTGAACGAGAAGAATTACAAGCAGGCACTGAAGAAGTATGACATGCTCTGCCTGCTCTTCCATGAGCCTGTGAGCT
CTGACAAGGTCTCCCAGAAGCAGTTCCAGATGACAGAGATGGTCCTGGAGCTGGCAGCTCAGGTCCTGGAGCCCAGGAGCATTGGCTTT
GGGATGGTGGACTCCAAGAAGGATGCCAAGCTTGCTAAGAAGTTAGGCTTGGTTGAAGAGGGAAGTCTCTATGTCTTTAAGGACGAGCG
GTTGGTTGAGTTTGATGGGGAACTGTCTGCAGATGTCTTGGTGGAATTCCTCTTGGATTTGCTAGAAGACCCCGTGGAGGTCATAAACA
GCAAGCTGGAGCTTCAGGCCTTTGACCAGATTGACGACGAAATCAAACTCATCGGCTACTTCAAGGGAGAAGACTCAGAACATTACAAG
GCATTTGAAGAAGCTGCTGAACAATTCCAGCCCTATGTCAAGTTCTTTGCCACCTTTGACAAAGGGGTTGCCAAGAAGCTGGGCCTAAA
GATGAACGAGGTGGACTTCTATGAACCCTTTATGGATGAGCCTGTTCACATCCCTGATAAGCCCTACTCCGAAGAGGAGCTGGTTGATT
TTGTGAGAGAGCACAGAAGGGCCACCTTGAGGAAGCTGCGCCCAGAGGACATGTTTGAGACGTGGGAGGATGACATGGAGGGTATCCAC
ATCGTAGCCTTTGCTGAAGAAGATGACCCAGACGGTTTTGAGTTCCTGGAAATCCTGAAGCAGGTTGCCAGGGACAACACCGATAATCC
TGACCTGAGCATTGTCTGGATTGACCCTGATGACTTTCCTCTGCTCATCACTTACTGGGAAAAGACCTTCAAGATTGACCTGTTCAGAC
CACAGATCGGGGTGGTGAACGTCACGGATGCTGACAGCGTCTGGATGGACATCAGAGATGATGATGACCTGCCCACAGCCGAGGAGCTG
GAGGACTGGATAGAGGACGTGCTTTCCGGGAAGATAAATACCGAAGATGATGACGACGATGAAGATGACGATGATGACGATGATGATGA
TGATGACGACGACGACGATGATGACGACGACGATGACGATGATGATGATGACGATGACTAACTGTGACTCTGTGCAGTTTGACTTGTGG
GGCCCGAGAGCCCTTGCCCTGCGGCAGGTCCCTCCATTGGAAGACCTATGTTTGAGCATCAGCAAGCACCGCCGCTCCTCTTTCCCCCC
TGCCCCGCTCCCCCTGCCCTCGCCTGTGCCGGGACCCCCTCGCCTGATTCCCAGCGGTGCTGAGCCACCGGGACTGCCCCTGCGGAGGG
CAGCACCTGCAGGCACCCAGCACCGCTGCAAATCATGCCTCCTTAGTAAGGACAGCAGGAATCTACAGGGAACCTGCCCCGAGTGTCCC
AGGGCAGGAGAAAAGTGCCTGCCTGCTTGCTGCCAGAGGGACGCAGGGAGCCAGTTTGTATTTTCTGTGTTCATCAGCCCAGCACTTAA
CATCCAAAGACCAAATCTCACTTCAAGACATAGGGAAAACCCTAACTGTAAAGTTTAGCCCTTTTATTAAACACAGTGCCCTTTGCAGC
TACTCTGCCAAGACCACTCAATCCTGCAAGTTCTGCTGCAGGAGTCTGTAGCTACTCCCTAACGTGCTAAGAGCCAGAAAAATGCTTTG
GGAGGGGAGGGAGGTTTTAGGGATGCTATTAAAGTTTTCAGTGATTCCACATAATGACAGGATATTCTTGTCTGGCAGTGCAATACAAA
ACCAAACACCGGGTCCCCTGTCTGAACTTCTTTCTTCTCCAAAGCTCAGGCCACAGCTGGAGTCAGTGCAGGGGGCCTGACACAAAGCT
CATGAGTTCCTGAACTGCTGGACAGGCTGGCCCAGCTCTGAGCCCAAAGTGAGTCCAAACCACTGAGTCTGAAGATCATGGCACTGAGC
CAAGCCAATGGAAATATCCATAAGGGCCCACCCTGAAGCATTCCTATTCCCCATGACCTTCCAGCTTTTGCTGACTCTTCACCCAAAGC
CCGTGTAGCATCTTTTCCCATGATCAAGATGTTGGGAGGGAACACATCCATATTAGATAGTGGATGAGGTGCATTGGGGTGTGAGGAGC
AGACGGATCCAAGCTGTTTGCCTTTCGAGTATTTAGTTTCTCCCTGCTTGGAGACTCAGATTTCCCATGTGAGAGGCCTCTTTTGACTG
CAGGCTGAGAAGAAGCTCAGGGTTTGACCAGTTTGCAGTCAAGAGGGTCACTCACGAAGGCAAGGGTCTGTACCATGGTCCTGGCAAGT
GTAGCACCATCGATGTGCAGGTCACTGTGGCAGCATTAGGCCTCCCTGCTTTCCCATGCTGGCAGATTTCAGTTGTCTTAGCTTATCTT
TGGCAATAGTGCAATGAATGGAGAGTTTGGAGATGGTTTTATCTTTATAAATAGATTTATAAAGATGTTCTCCAGTTGTTCTGAGATGC
TAGCATAGGTAGTGTACACATCTTCTCTCTTGCTATAAATGAAAAACATTGTTCCTTATACTGGCTTGGGACAGAGGTAGGTGATGTGA
GGGGAAGTAACAGTGCGAGGGACAGAGGAGTCTGAGTTATGCAAATGTGAATCAGCCTGATGAGAAACAAGGTTTACACTCTATATATT
TCCTTTATCAGATTAATTGCAAAGCAAGATTTTATAGATATTTTTTTTCCTGTTTGTTTTTCAGTTTCTTTGACTCTCTCTACATTGGC
TGAAATAGAAATTTGAAACTACTGGTATAAAATAGCTGAAGTATTGATATGTTCCCATGTGTGCCATATCCCCTTGGTGGAAAATGCCA
GCATGAACAACACATAGCTGGATTTCTCTCTGTCTCTATGCACTTGAATGAATACTGTGCAACATGGTAGATGACTACAGGCCAAGTTA
GCTCCTTGTTGAGCATCAAACCCTCAACATGTGCTTTTCAGATACAGGCTGTTAGAATAATAGACAAGGCAGCAAAGTCCCCAGGTCAA
CCATTTGTTCTGCCATGCTTCCTTATGATTTCCTGGAAGATGAGCCAAGTCTGTTCAGGGAGAACAATCAACTGGTTGCTCTGGGGTGA
TGAACAACTTCGGAAAGTTTTGAAATTCGGAGTGTTCTCAGCATGGAGGTCTCCTCCTCTCTTCCACTCACACCCTACTCTTTGCTGGC
CGACAGGGTGCCCAGCACTCTCCTTCTGCTTTTTCCTCAAGAGGTTTGCTCAGAGAGGGAGTGAGGAGGACTCAAAGTCTCATTCACCT
GCAGTGGTAAGGAACAAGAAGAGGCAGAAGTATTCCCCTGGTAGTCTCCTGGCATCTGATTAAGGGACAACTACTGACCTGAGGAAAGA
ACTAGCTTAGCTTTACCTTTGGGCTATTCCCAGGAGCAAAACATGCACAGTTTCACCCTGCATAAGGTGCAGCCTGCACCAGCCATGTG
CCTCCTCATCAGTCACTCCAAACATGAAGAATAATGAAAAATTCCTCTTGTGCCTTTCCTAACTGGCACAATCTCCAGATGTGTCAGTT
GCTTGAGTACTTTCTGTGTGATCCTCTCCCATTGTGGATGCCCCTCTGTTCTGAAGTGCCCCTCTCTAGATGCTCTGAGAGGCGCAGAT
TATCCCAGCAGAGCAGGCAGACCCTGCTGACAGCACCACGTTCAAGCACACAGGTGTTTCACAGGCACTTAAACACTCCAAAACAACAG
ACCTCTAGGGGGAAAAGAAAGAAACAATGTAGAAGTGTAGAGGCTGAACTCATACTGCAGAGTGGTGCCAGTGGTTTATGGGAAGTTTC
CCTTGAGTTATGCAAGAAAGAGCTAATCCTACAGCTGCCACTCCTTCATGGTGCAGGGATGGGGCTGCCCTTTCTCTCTTCCCTCAGTC
CACCATAACAGTTCTCATCACTGTTTTTGCCCACAAACAGGAACTGTTTATCATCATCTTTTGGGACAATCGCATAAAAAGTTAGGAAA
GCTTTGCCACAGAGGAAGGATGGCAGCAATGAGGATGGGTGGGCAGCAAAGCCTCTCCCATTTATCTGTAACACCTTGTCTACCCCAAG
ATCCTAATAACCAAACTGCTGAATGATGCAGTGCCCCTTGTGAAGGAGATGGCAGCTTGAATTTGAGTGTTTTGCTTGCAGGGACAGGT
AAGACAATGGTATGGAGATGACACACAGGACCCCGCTCCTCTCCTCTAAGGTGCTCCTTGGCTTCTCTGTCACAAGTAGGAACACCTGG

CATTTGAAAGGCACTGGTGGCCCTGAGGAGCAAAGTCAGCCTAACCCTGCCTACAATTCACTGAGAAGAAACAGCACATTTGGATGAGG
AATCTACAAAATAACCCTTTATTTAAAAAAAAATATAAATTTGTGTATGTGGGCAGCACTGAGTGCTCAACAAGCACATCTCTCATAAC
AAATAGTTCTTTTTCTCCAGCATGTTAACTAGATATATTGGAAAGGTCTTTATCCAGATAAAGACGGAGATTTTATTTTGCCCTCATCC
AAACACAAGTGCTTCAAGCTGATCAGTTTCTTCACAGAATCATCCAATCATTTTGGTTGGAAAAGACCCCCTAAGATCATTGAGTCTGA
CCATTAACCCACCACTGTCAAGTTAATACCCAAAGTACTCAAAAACCTGGTCTTATCTGTCCCCAGTGATAAAGAGGACCCCACATGCA
ATATGTGGCAAGAACACCTCCTCCCAGGCCCCAGACACAAGGCTGGGGGGCACAGCCGAGGGGTTTGTTTGGGGTGCTGTCCCACAGCC
CATCCTGTGCTGCTCATGGGCAACAATCAAGGGAGAAGGAGATCCATTAGGAGCAGCTGCAGCTCTCATACCGCAGCATGGAGGAGAAA
GTTTCTGCCTTTGCAGCACTCGCTGATGTAGAGACTCTGTGAACTCAAAAGTGCTCTGAGTTTATGTCAACTCTTTGGGAAACAGCTGC
AGGAGGAAAGGACAAGGGAGACACTCTGGAACTGGCAGATTACTCTGTGTTTTCAGGGTCAGCAGTTTTTGTGATGTTTCTGAGGAGTG
ATGAGTCTCCTGTCTCCCATGGGAAAGGAACAGTCTCATAGTAAAACCAGAGATCTCAGCTACTTCTCAGCCCAGAATGAAGCTGCTCA
GCCAGACACCAACGTGAACACAGAGCTGAGGACACCGGCTGCTTCTCCATGTTTGTCTGCATATCATACCTAAAGAAATGACGGATTCA
TTACAGAGACCAAACAGAAAACATTGTCTTTTATTCCCAGGAAAATAATTAATTAGACAGGAACATATCACTTCCCAGCTGCCACTGAC
TAACAGTCTTGTGAAAAGAGGACAAGACTGCATCTGGATTCTTCTCCAATGCAGCAAAGCTTTGGCTTTGAGGAAGTCCCACACACAGC
CTGTTGCACACCAATGCCTGGACTTGGGGGGCTGAAGAGGAAGATGGGCAAACAGGCAAGAAGCACCCCAGCCCTCTGAGGCAAAGAGC
TCACTGCCTCCTGGCTGCTTTCACACCCCAGGCACATCCATGCCATGCAGTGCAAGACCAGCTGCTAGAAACTGGAAACAGCAAAGCAC
AGCAGGAAGGATAACTGGCCTCAGTAATGAGGCTGTGCCAGCAGGACTGTGTCCTACCTGAGACCCACTGCACTGCAGCATCCCCTGTC
TTCTCCCCACTCCCTTGTACCACTGACCTTTAGCAAAGCAGGCCTTGGATGGTCACTACCCCTGGGGACAGCCGTGGGATGACTCTCAC
ACCTCTTTGGCAAAAGATGACATCTAATGACAATGGGGACCCATATGGGACAGGGCCTTGGCCCAGCTCCCAGAACACTCTGCCAGGTT
TAAAAGAGGTGACTCGGTCGATCTCCAGGAGGTCGTCCAGGATGATGT

>CASQ2_Poecile_palustris_CDS
ATGAAGGCAATTTGCTGGATCCTGGCAGGTTTTTACCTGCTTTTCTGCTGCAAGGCAGAAGAAGGACTGAACTTCCCTACTTATGATGG
GAAAGACCGAGTGATCGACCTGAACGAGAAGAATTACAAGCAGGCACTGAAGAAGTATGACATGCTCTGCCTGCTCTTCCATGAGCCTG
TGAGCTCTGACAAGGTCTCCCAGAAGCAGTTCCAGATGACAGAGATGGTCCTGGAGCTGGCAGCTCAGGTCCTGGAGCCCAGGAGCATT
GGCTTTGGGATGGTGGACTCCAAGAAGGATGCCAAGCTTGCTAAGAAGTTAGGCTTGGTTGAAGAGGGAAGTCTCTATGTCTTTAAGGA
CGAGCGGTTGGTTGAGTTTGATGGGGAACTGTCTGCAGATGTCTTGGTGGAATTCCTCTTGGATTTGCTAGAAGACCCCGTGGAGGTCA
TAAACAGCAAGCTGGAGCTTCAGGCCTTTGACCAGATTGACGACGAAATCAAACTCATCGGCTACTTCAAGGGAGAAGACTCAGAACAT
TACAAGGCATTTGAAGAAGCTGCTGAACAATTCCAGCCCTATGTCAAGTTCTTTGCCACCTTTGACAAAGGGGTTGCCAAGAAGCTGGG
CCTAAAGATGAACGAGGTGGACTTCTATGAACCCTTTATGGATGAGCCTGTTCACATCCCTGATAAGCCCTACTCCGAAGAGGAGCTGG
TTGATTTTGTGAGAGAGCACAGAAGGGCCACCTTGAGGAAGCTGCGCCCAGAGGACATGTTTGAGACGTGGGAGGATGACATGGAGGGT
ATCCACATCGTAGCCTTTGCTGAAGAAGATGACCCAGACGGTTTTGAGTTCCTGGAAATCCTGAAGCAGGTTGCCAGGGACAACACCGA
TAATCCTGACCTGAGCATTGTCTGGATTGACCCTGATGACTTTCCTCTGCTCATCACTTACTGGGAAAAGACCTTCAAGATTGACCTGT
TCAGACCACAGATCGGGGTGGTGAACGTCACGGATGCTGACAGCGTCTGGATGGACATCAGAGATGATGATGACCTGCCCACAGCCGAG
GAGCTGGAGGACTGGATAGAGGACGTGCTTTCCGGGAAGATAAATACCGAAGATGATGACGACGATGAAGATGACGATGATGACGATGA
TGATGATGATGACGACGACGACGATGATGACGACGACGATGACGATGATGATGATGACGATGACTAA

>CASQ2_Poecile_palustris_protein
MKAICWILAGFYLLFCCKAEEGLNFPTYDGKDRVIDLNEKNYKQALKKYDMLCLLFHEPVSSDKVSQKQFQMTEMVLELAAQVLEPRSI
GFGMVDSKKDAKLAKKLGLVEEGSLYVFKDERLVEFDGELSADVLVEFLLDLLEDPVEVINSKLELQAFDQIDDEIKLIGYFKGEDSEH
YKAFEEAAEQFQPYVKFFATFDKGVAKKLGLKMNEVDFYEPFMDEPVHIPDKPYSEEELVDFVREHRRATLRKLRPEDMFETWEDDMEG
IHIVAFAEEDDPDGFEFLEILKQVARDNTDNPDLSIVWIDPDDFPLLITYWEKTFKIDLFRPQIGVVNVTDADSVWMDIRDDDDLPTAE
ELEDWIEDVLSGKINTEDDDDDEDDDDDDDDDDDDDDDDDDDDDDDDDDD*

9.11 *Pseudopodoces humilis* (Tibetan ground-tit)

From: Trinity *de novo* assembly of SRR768236. CASQ1 from complement of TRINITY_DN1657_c0_g3_i1. CASQ2 from TRINITY_DN131_c0_g1_i1.

>CASQ1_Pseudopodoces_humilis_transcript_assembly
TGTCCCTCTGTCCCTCTGTCCCGTGTCCCTCTGTCCCGTGTCCCCATGTCCCCGCTGTGGCCCTGCCGGTGGCTCCTGGCTCTGCTGGC
TCTGTCCCCGGGTGTCCCGGGTGTCCCGGGGGTCTCGGGGGGTCCCGGGGGGCCGCTGCTCCCGGTGAGCTCCGGGAGCCCCCCCGAGC
TGCGGCGGCGGCCCGGGGACACCGGACAGCGGCCCGAGGAGCTGGAGGCTCTGGGGACCCCCGTGCAGGTGGTGGCAGCCGGGCAGGAG
CTCCGGGCCTTTGGGGACATCGAGGAGGAGCCCAAAGTCATCGGGTACTTCGAGGGACGGGACTCGGAGTCCTTCCAGGCCTTCTCCGC
CACCGCCCAGCGCTTCCACCCCTCCCTCCTCTTCTTCGCCACCTTCGACCCCCAGGCGGCGCAGGAGCTGCGGCTGGGGCTGAACCAGC
TCCATCTCTTCGAGCCCTTCCTGGAGCAGCCCCGGAGCTTTCGGGGGGACCCCGGGGACCCCCACGGGCTCGAGGCCTTCGTGGAGAGC
AACAAGCGGGCCACTCTGCAGAAACTCAAAGCTCAGAGCACGTCCCAGAACTGGGAGGACCTTTGGGATGGGACCCACATCGTGGCCTT
TGCCGAGGGGGATGATCCCGACGGGTTCGAGTTCCTGGAGATCCTGAAGGAGGTGGCCCAGGACAAGAGGGACAATCCCGATTTCAGCA
TCCTGTGGATCGATCCCCATGATTTCCCGCTGCTCGTCCCCTCCTGGGAGGACACCTTTGACATCGACCTGTCCCGGCCCCAGCTCGGC
GTGGTCAATGGCACTGACCATGCCGGCAGCGTGTGGCTGGACATGGAGGATGAGGAGGATCTGCCGGGACCTGAGGAGGTCCTGGGGTG
GCTCAAGGAGGTTCTGGAGGGGGACACTGGGGACAGGGATGGCCATGACGATGAAGACGATGACGATGAAGATGACGATGATGATGAAG
ACGATGACAATGAAGATGAAGATGATGACGACGATGATGATGATGACGATGGTGACAAAGTTGATGACGATGATGGTGCTGACTCATAA
CAATGATGACGATGACGATGATGTCGTCACTGACGATGTTGAAGATGATGACAATTACAATGATGATGATGATGACGATGATGATGAAG

ATGACAATGACGACAATGACATGACAATGATGATGACGATGAAGATGATAATGACAATGACAACACTGACAATGATGAAGATGATGACA
ATTACAATGACAATGATGATGATGATGATGACGATGACGATGATGACGAAATTGATGATGACGATGATGACGACAATGATGATGATGAC
GATGGTGACAAAGTTGATGACGATGATGGTGCTGACTCATAACAATGATGACGATGACGATGATGTCGTCACTGACGATGTTGAAGATG
ATGACAATTACAATGATGATGATGATGACGATGATGAAGATGATGACAATGACGACAATGACATGACAATGATGATGACGATGAAGATG
ATAATGACAATGACAACACTGACAATGATGAAGATGATGACAATTACAATGACAATGATGATGATGATGATGACGATGACGATGATGAC
GAAATTGATGATGACGATGATGACGACAATGATGATGATGACGATGGTGACAAAGTTGATGACGATGATGGTGCTGACTCATAACAATG
ATGACGATGACGATGATGTCGTCACTGACGATGTTGAAGATGATGACAATTACAATGATGATGATGATGACGATGATGATGAAGATGAC
AATGACGACAATGACATGACAATGATGATGACGATGAAGATGATAATGACAATGACAACACTGACAATGATGAAGATGATGACAATTAC
AATGACAATGATGATGATGATGATGACGATGACGATGATGACGAAATTGATGATGACGATGATGACGACAATGATGATGATGACGATGG
TGACAATGATGATGAAAACAATGAAGATGTCAATGATGATGATGATGATGATGGTGACGATGGTGACGATGAAAACAATGACGATGTCA
ATAATGATGATGATGATGATGATGATGATGATGATGAT

>CASQ1_Pseudopodoces_humilis_CDS
ATGTCCCCGCTGTGGCCCTGCCGGTGGCTCCTGGCTCTGCTGGCTCTGTCCCCGGGTGTCCCGGGTGTCCCGGGGGTCTCGGGGGGTCC
CGGGGGGCCGCTGCTCCCGGTGAGCTCCGGGAGCCCCCCCGAGCTGCGGCGGCGGCCCGGGGACACCGGACAGCGGCCCGAGGAGCTGG
AGGCTCTGGGGACCCCCGTGCAGGTGGTGGCAGCCGGGCAGGAGCTCCGGGCCTTTGGGGACATCGAGGAGGAGCCCAAAGTCATCGGG
TACTTCGAGGGACGGGACTCGGAGTCCTTCCAGGCCTTCTCCGCCACCGCCCAGCGCTTCCACCCCTCCCTCCTCTTCTTCGCCACCTT
CGACCCCCAGGCGGCGCAGGAGCTGCGGCTGGGGCTGAACCAGCTCCATCTCTTCGAGCCCTTCCTGGAGCAGCCCCGGAGCTTTCGGG
GGGACCCCGGGGACCCCCACGGGCTCGAGGCCTTCGTGGAGAGCAACAAGCGGGCCACTCTGCAGAAACTCAAAGCTCAGAGCACGTCC
CAGAACTGGGAGGACCTTTGGGATGGGACCCACATCGTGGCCTTTGCCGAGGGGGATGATCCCGACGGGTTCGAGTTCCTGGAGATCCT
GAAGGAGGTGGCCCAGGACAAGAGGGACAATCCCGATTTCAGCATCCTGTGGATCGATCCCCATGATTTCCCGCTGCTCGTCCCCTCCT
GGGAGGACACCTTTGACATCGACCTGTCCCGGCCCCAGCTCGGCGTGGTCAATGGCACTGACCATGCCGGCAGCGTGTGGCTGGACATG
GAGGATGAGGAGGATCTGCCGGGACCTGAGGAGGTCCTGGGGTGGCTCAAGGAGGTTCTGGAGGGGGACACTGGGGACAGGGATGGCCA
TGACGATGAAGACGATGACGATGAAGATGACGATGATGATGAAGACGATGACAATGAAGATGAAGATGATGACGACGATGATGATGATG
ACGATGGTGACAAAGTTGATGACGATGATGGTGCTGACTCATAA

>CASQ1_Pseudopodoces_humilis_protein
MSPLWPCRWLLALLALSPGVPGVPGVSGGPGGPLLPVSSGSPPELRRRPGDTGQRPEELEALGTPVQVVAAGQELRAFGDIEEEPKVIG
YFEGRDSESFQAFSATAQRFHPSLLFFATFDPQAAQELRLGLNQLHLFEPFLEQPRSFRGDPGDPHGLEAFVESNKRATLQKLKAQSTS
QNWEDLWDGTHIVAFAEGDDPDGFEFLEILKEVAQDKRDNPDFSILWIDPHDFPLLVPSWEDTFDIDLSRPQLGVVNGTDHAGSVWLDM
EDEEDLPGPEEVLGWLKEVLEGDTGDRDGHDDEDDDDEDDDDDEDDDNEDEDDDDDDDDDDGDKVDDDDGADS*

>CASQ2_Pseudopodoces_humilis_transcript_assembly
CAGGGCTTGGCCTGACAGCTTCTCCCGTCTGTCTCTCTCTTTGTTGTGAGTACAGGACAGGAAAGTTTGCTGCTCGCCACCAGCCTTGC
AGTGTTTGGGCTCAACACTAGGAAAGCAGCAGATAGAAACTCAGAGAAACTTAGTTCAAACTTCACCTGGAGTCTTTGGTCTCCCTCTC
TTCTCAAATTAAGAGAGAGGGGCACATTTACGCACCCTTTGCTGCACTCCAAGGGTGCCCCTATGAAGGCAATTTGCTGGATCCTGGCA
GGTTTTTACCTGCTTTTCTGCTGCAAGGCAGAAGAAGGACTGAACTTCCCTACTTATGATGGGAAAGACCGAGTGATCGACCTGAACGA
GAAGAATTACAAGCAGGCACTGAAGAAGTATGACATGCTCTGCCTGCTCTTCCATGAGCCTGTGAGCTCTGACAAGGTCTCCCAGAAGC
AGTTCCAGATGACAGAGATGGTCCTGGAGCTGGCAGCTCAGGTCCTGGAGCCCAGGAGCATTGGCTTTGGGATGGTGGACTCCAAGAAG
GATGCCAAGCTTGCCAAGAAGTTAGGCTTGGTTGAAGAGGGAAGTCTCTATGTCTTTAAGGACGAGCGGTTGGTTGAGTTTGATGGGGA
ACTGTCTGCAGATGTCCTGGTGGAATTCCTCTTGGATTTGCTAGAAGACCCTGTGGAGGTCATAAACAGCAAGCTGGAGCTTCAGGCCT
TTGACCAGATCGACGACGAAATCAAACTCATCGGCTACTTCAAGGGAGAAGACTCAGAACATTACAAGGCATTTGAAGAAGCTGCTGAA
CAATTCCAGCCCTATGTCAAGTTCTTTGCCACCTTTGACAAAGGGGTTGCCAAGAAGCTAGGCCTAAAGATGAACGAGGTGGACTTCTA
TGAACCCTTTATGGATGAGCCTGTTCACATCCCCGATAAGCCCTACTCCGAAGAGGAGCTGGTTGATTTTGTGAGAGAGCACAGAAGGG
CCACCTTGAGGAAGCTGCGCCCAGAGGACATGTTTGAGACGTGGGAGGATGACATGGAGGGTATCCACATCGTAGCCTTTGCTGAAGAA
GATGACCCAGATGGTTTTGAGTTCCTGGAAATCCTGAAGCAGGTTGCCAGGGACAACACTGATAATCCTGACCTGAGCATTGTCTGGAT
TGACCCTGATGACTTTCCTCTGCTCATCACTTACTGGGAGAAGACCTTCAAGATTGACCTGTTCAGACCACAGATCGGGGTGGTGAACG
TCACAGACGCTGACAGCGTCTGGATGGACATCAGAGATGATGATGACCTGCCCACAGCCGAGGAGCTGGAGGACTGGATAGAGGACGTG
CTTTCCGGGAAGATAAATACCGAAGATGATGACGACGATGAAGATGATGATGATGACGATGATGATGACGATGATGACGATGACGATGA
CGACGACGACGATGACGATGATGACGATGACGATGACTAACTGTGACTCTATGCAGTTTGACTTGTGGGGCCCGAGAGCCCTTGCCCTG
CGGCAGGTCCCTCCATTGGAAGACCTATGTTTGAGCATCAGCAAGCACCGCCGCTCCTCTTTCCCCCCTGCCCCGCTCCCCCTGCCCTC
GCCCGTGCCGGGACCCCCTCGCCTGATTCCCAGCGGTGCTGAGCCACCGGGACTGCCCCTGCGGAGGGCAGCACCTGCAGGCACCCAGC
ACCGCTGCAAATCATGCCTCCTTAGTAAGGACAGCAGGAATCTGCAGGGAATCTGCCCCGAGTGTCCCAGGGCAGGAGAAAAGTGCCTG
CCTGCTTGCTGCCACAGGGACGCAGGGAGCCAGTTTGTACTTTCCGTGTTCATCAGCCCAGCACTTAACATCCAAAGACCAAATCTCAC
TTCAAGACATAGGGAAAACCCTAACTGTAAAGTTTAGCCCTTTTATTAAACACAATGCCCTTTGCAGCTACTCTGCCAAGACCACTCAA
TCCTGCAAGTTCTGCTGCGGGAGTCTGTAGCTACTCCCTAACATGCTAAGAGCCAGAAAAATGCTTTGGGAGGGGAGGGAGGTTTTAGG
GATGCTATTAAAGTTTTCATTGATTCCACATAATGACAGGATATTCTTGTCCGGCAGTGCAATACAAAACCAAACACCGGGTCCCCTGT
CTGAACTTCTTTCTTCTCCAAAGCTCAGGCCACAGCTGGAGTCAGTGCAGGGGGCCTGATACAAGACACAAAGCTCATGAGTTCCTGAA
CTGCTGGACAGGCTGGCCCAGCTCTGAGCCCAATGTGAGTCCAAACCACTGAGTCTGAAGGTCATGGCACTGAGCCAAGCCAATGGAAA
TATCCATAAGGGTATATGAAGCATTCCTATTCCCCATGAACTTCCAGCTTTTGCTGACTCTTCACTCAAAGCCCGTGTAGCATCTTTTC
CCATGATCGAGATGTTGGGAGGGAACACATCCATATTAGATAGTGGATGAGGTGCACTGGGGTGTGAGCAGCAGACGGATCCAAGCTGT

TTGCCTTTCGAGTATTTAGTTTCTCCCTGCTTGGAGACTCAGATATCCCATGTGAGAGGCCTCTTTTGACTGCAGGCTGAGAAGGAGCT
CAGGGTTTGGCCAGTTTGCAGTCAAGAGGATCACTCACAAAGGCAAGGGTCTGTACCATGGTCCTGGCAAGTGTAGCACCACCCATGTG
CAGGTCACTGTGGCAGCATTAGGCCTCCCTGCTTTCCCATGCTGGCAGATTTCAGTTGTCTTAGCTTATCTTTGGCAATAGTGCAATGA
ATGGAGAGTTTGGAGATGGTTTTATCTTTATAAATAGATTTATAAAGATGTTCTCCAGTTGTTCTGAGATGCTAGCATAGGTAGTATAC
ACATCTCCTCTCTTGCTATAAATGAAAAACATTGTTCCTTATACTGGCCTGGGACAGAGGTAGGTGATGTGAGGGGAAGTAACAGTGCG
AGGGACAGAGGAGTCTGAGTTATGCAAATGTGAATCAGCCCGATGAAAAACAAGGCTTACACTATATATATTTCCTTTATCAGATTAAT
TGCAAAGCAAGATTTTATAGGTATTTTTTTCCTGTCTGTTTATCAGTTTCTTTGACTCTCTCTACATTAGCTGAAATAGAAATTTGAAA
CTACTGCTATAAAATAGCTGAAGTATTGATATGTTCCCATGTGTACCATATCCCCTTGGTGGAAAATGCCAGCATGAACAACACATAGC
TGGATTTCTCTCTGTCTCTATGCACTTGAATGAATACTGTGCAACATGGTAGATGACTATAGGCCAAGTAAGCAGATACAGCCTGTTAG
AAGAATAGACAAGGCAGCAAAGTCCCCAGGTCAACCATTTGTTCTGCCATGCTTCCTTATGATTGCCTGGAAGATGAGCCAAGTCTGTT
CAGGGAGAACAATCAACTGGTTGCTCTGGGATGATGAACAACTTTGGAAAGTTTTGAAATTCAGAGTGTTCTCAGCATGGAGGTCTCCT
CCTCTCTTCCACTCACATCCTACTCTTTGCTGGCCAACAGGGTGCCCAGCACTCTCCTTCTGCTTTTTCCTCAAGGGGTTTGCTGGAGG
ACCCCAGCTCAGAGAGGGAGCGAGGAGGACTCAAAGTCTCATTCACCTACAGTGGTAAGGAACAAGAAGAGGCAGAAGTACTCCCCTGG
TAGTCTCCTGGCATCTGATTAAGGGACAACTACTGACCTGAGGAAAGAACTAGCTTATCTTTACCTTTGGGCTACCCCCAGGAGCAACA
CATGCACACTTTCACCCTGCATAAGGTGCAGCCTGCACTGGCCATGTGCCTCCTCATCAGTCACTCCAAACGTGAAGAATAATGAAAAA
TTCCTCTTGTGCCTTTCCAAACTGGCACAATCTCCAGATGTGCCAATTGCTTGAGTGCTTTCTGTGTGATCCTCTCCCACTGTGGATGC
CCCTCTGTTCTCAAGTGCCCCTCTCTAGATGCTCTGAGAGGCACAGATTACCCCAGCAGAGCAGGCAGACCCTGCTGACAGCACCATGT
TCAAGCACACAGGTGTTTCACAGGCACTTAAACACTTCAAAACAACAGACCTCTAGGGGGAAAAGAAAGAAACAATGTAGAAGTGTAGA
GACTGAACTCATACTGCAGAGTGGTGCCAGTGGTTTATGGAAAGTTTCCCTTGAGTTATGCAAGAAAGAGCTAATTCTACAGCTGCCAC
TCCTTCATGGTGCCAGGGATGGGGCTGCCCTTTCTCTCTTCCCTCAGTCCATCATAACAGTTCTCATCATTGTTTTTGCCCACAAACAG
GAACTGTTTATCGTCATCTTTTGGGACAATCGCATAAAAAGTTAGGAAAGCTTTGCCACAGAGGAAGGATGACAGCAGTGAGGATGGGT
GGGCAGCAAAGCCTCTCCCATTTATCTGTAACACCTTGTCTACCCCAAGATCCTAATAACCAAACTGCTGAATGATGCAGTGCCCCTTG
TGAAGGAGATGGCAGCTTGAATTTGAGTGTTTTGCTTGCAGGGACAGGTAAGACAATGGTATGGGGATGACACACAGGACCCCAGTCCT
CCCCTCTGAGGTGCTCCTTAGCTCCTCTGTCACAAGTAGGAACACCTGGCACTTGAAAGGCACTGGTGGCCCTGAGGAGCAGTCAGCCT
AACCCTGCCTACTATTGACTGAGAAGAAACAGCACATTCGGATGAGGAGCCTACAAAATAACCCTTTATTAAAAAAAATATAAATTTGT
TTATGTGGGCAGCACTGAGTGCTCAACAAGCACATCTCTCTTAACAAATAGTTCTTTTTCTCCAGCATGTTAACTAGATATATTGGGGA
GGTCTTTATACAATTAAAGACAGGGATTGTATTTTGCCCTCATCCAAACATGAGTGCTTCAAGCTGATCAGTTTCTTCACAGAATCATC
CAATCATTTTGGTTGGAAAAGACCCCCTAAGATCATTGAGTCTGACCATTAACCCACTACTGTCAAGTTAATACCCAAAGTACTCGAAG
ACCTGGTCTTATCTGTCCCCAGTGATAGAGAGGACCCCACATGCAATATGTGGCAAGAACACCAAGTTATGCAGCGCCTCCTCCCAGGC
CCCAGACACAGGGCTGGAGGGCACAGCCGAGGGGTTTGTTTGGGGTGCTGTACCACAGCCCATCCTGCACTGCTCATGGGAAACAACCA
AGGGAGAAGGAAATCCATTAGGAGCAACTGCAGCTCTCATACTGCAGAATGGAGGAGAAAGTTTCTGCCTTTGCAGTAGTCACTGCTGT
AGAGGCTCTGTGAACTCAAAAGTGCTCTGAGTTTATGTCAACTCTTTGGGAAACAGCTGCAGGAGGAAAGGACAAGGGAGACACTCTGG
AATTGGCAGATTACTCCGTGTTTTCAGGGTCAGCAGTTTTTGTGATGTTTCTGAGGAGTGATGAGTCTCCTGTCTCCCATGGGAAAGGA
ACAGTCCCATAGTAAAACCAGAGATCTCAGCTGCTTCTCAGCCCAGAATGAAGCTGCTCAGCCAGACACCAATGTGATCACAGAGCTGA
GGACACCGGCTGCTTCTCCACGTTTGTCTGCATATCATACCTAAAGAAATGACGGATTCATTACAGAGACCAAACAGAGAACATTGTCT
TTTATTCCCAGGAAAATAATTAATTAGACAGGAACATATCACTTCCCAGCTGCCACTGACTTACAGTCTTGTGAAAAGAGGACAAGACT
GCATCTGGATTCCTCTCCAATGCAGCAAAACTTTGGCTTTGAGGAAGTCCCACACACAGCCAGTTGCACACCAATGCCTGCCTGGACTT
GGGGGGCTGAAGAGGAGGATGGGCAAACAGGCAAGAAGCACCCCGGCCCTCTGAGGCAAAGAGCTCACTGCCTTCTGGCTGCTTTCACA
CCCCAGGCACATCCATGCCATGCAGTGCAAGACCAGCTGCTAGAAACTGGAAACAGCAAAGCACAGCAGGAAGGATAACTGGCCTCAGT
AATGAGGCTGTGCCAGCAGGACTGTGTCCTACCTGAGACCCACTGCACTGCAGCATCCCCTGTCTTCTCCCCACTCCCTTGTACCACTG
ACCTCTAGCAAAGCAGGCCTTGGATGGTCAGTACCCCTGGGGACAGCCGTGGGATGACTCTCACACCTCTTTGGCAGAAGACGACATCT
AATGACAACGGGGACCGTGGGGCAGGGCCTTGGCCCAGCTCCCAGAACACTCTGCCAGATTTAAAAGGTGCTAAGCTATTTAAAGGAAA
AAAAAAAAAAAAGCTACAGAGCTTCCTTGCAGCTTGAAGACCCATTTCAGGAATCAGATTACAAACTGCCTTTGGGATGGAAGCAAAGAT
CCTTCCGGTGCCTCGCTCCCCCCCTTGCGCTTCCTTTTGCCAAACCCCTTGTGATAAAGGGGATCAGCAAAGCACTCTAAGGGCATTGT
CCCTAATGCTATGGAAAACAAGCAAACAGCTTTTTCACCTTCTTTTCATGCCTCTTGACTTAATACTCTTCTGACCGTCACTGGCTCCA
ATAATTTATTTTCTCTCTCCATTTCTATGTAGCTCATCTTCCTCTTCTTCTCAACCACCCCCGCAGCATATGGTACTGAGGGTCACTGA
AGGACTGAGCCCTTCCCTGCCCCTACTGCCCCTTCCCCAGCCCAGGGAGACAGCAGCCTCCTTCACACCACTGCCACGAACTGCCTGTG
CTGGATCCCCACACCTGTGAACAGGAGGAGGGACATTTCCTTTCTGAGGAGGAAGATGTCCAGACAAGGCTGTCACTGACTTTTATTTT
GAGGGGTACAGGTGAGACATGCTCAGGGAAAAGTCATTTCACCAGCCTGGACAGAAGAATTTTTTGGTGGAACTCTTTTCCCACTGCCC
CAAAATCTAGAGGAGGAACCACCATCCTGCAACCAAGCCCTGACTGCTGTCTCTGTGGGAAGAGACCAGTAATACCCAGTGCCACCAGC
AATCACACCACCACATCATCTGAGCAGAAGTGTCATGGCTTCACATACAAGGAATAAACCTTGAGCGTTAAACGAGCTGAGTTTGCTGG
AGGGGGTATCCCACGATCAACAGCAGGCAGAGAACCACCTCCCCACCTCCCTGCCAGGGACTCCCAGTGCTGTCCTGACTGAGCCCTCC
CTTGGTCATGGGTGGTAAATCACTTACATAGCCATGGGCAACCTCACATGCTTTTGGATTTGGAGAAAGCCCAGCTTCAATCAGGTCCC
TCCCTCAGTAACACAGTTGAAAAATAAAGTGATGTTTGACCAAACAGCAA

>CASQ2_Pseudopodoces_humilis_CDS
ATGAAGGCAATTTGCTGGATCCTGGCAGGTTTTTACCTGCTTTTCTGCTGCAAGGCAGAAGAAGGACTGAACTTCCCTACTTATGATGG
GAAAGACCGAGTGATCGACCTGAACGAGAAGAATTACAAGCAGGCACTGAAGAAGTATGACATGCTCTGCCTGCTCTTCCATGAGCCTG
TGAGCTCTGACAAGGTCTCCCAGAAGCAGTTCCAGATGACAGAGATGGTCCTGGAGCTGGCAGCTCAGGTCCTGGAGCCCAGGAGCATT
GGCTTTGGGATGGTGGACTCCAAGAAGGATGCCAAGCTTGCCAAGAAGTTAGGCTTGGTTGAAGAGGGAAGTCTCTATGTCTTTAAGGA

CGAGCGGTTGGTTGAGTTTGATGGGGAACTGTCTGCAGATGTCCTGGTGGAATTCCTCTTGGATTTGCTAGAAGACCCTGTGGAGGTCA
TAAACAGCAAGCTGGAGCTTCAGGCCTTTGACCAGATCGACGACGAAATCAAACTCATCGGCTACTTCAAGGGAGAAGACTCAGAACAT
TACAAGGCATTTGAAGAAGCTGCTGAACAATTCCAGCCCTATGTCAAGTTCTTTGCCACCTTTGACAAAGGGGTTGCCAAGAAGCTAGG
CCTAAAGATGAACGAGGTGGACTTCTATGAACCCTTTATGGATGAGCCTGTTCACATCCCCGATAAGCCCTACTCCGAAGAGGAGCTGG
TTGATTTTGTGAGAGAGCACAGAAGGGCCACCTTGAGGAAGCTGCGCCCAGAGGACATGTTTGAGACGTGGGAGGATGACATGGAGGGT
ATCCACATCGTAGCCTTTGCTGAAGAAGATGACCCAGATGGTTTTGAGTTCCTGGAAATCCTGAAGCAGGTTGCCAGGGACAACACTGA
TAATCCTGACCTGAGCATTGTCTGGATTGACCCTGATGACTTTCCTCTGCTCATCACTTACTGGGAGAAGACCTTCAAGATTGACCTGT
TCAGACCACAGATCGGGGTGGTGAACGTCACAGACGCTGACAGCGTCTGGATGGACATCAGAGATGATGATGACCTGCCCACAGCCGAG
GAGCTGGAGGACTGGATAGAGGACGTGCTTTCCGGGAAGATAAATACCGAAGATGATGACGACGATGAAGATGATGATGATGACGATGA
TGATGACGATGATGACGATGACGATGACGACGACGACGATGACGATGATGACGATGACGATGACTAA

>CASQ2_Pseudopodoces_humilis_protein
MKAICWILAGFYLLFCCKAEEGLNFPTYDGKDRVIDLNEKNYKQALKKYDMLCLLFHEPVSSDKVSQKQFQMTEMVLELAAQVLEPRSI
GFGMVDSKKDAKLAKKLGLVEEGSLYVFKDERLVEFDGELSADVLVEFLLDLLEDPVEVINSKLELQAFDQIDDEIKLIGYFKGEDSEH
YKAFEEAAEQFQPYVKFFATFDKGVAKKLGLKMNEVDFYEPFMDEPVHIPDKPYSEEELVDFVREHRRATLRKLRPEDMFETWEDDMEG
IHIVAFAEEDDPDGFEFLEILKQVARDNTDNPDLSIVWIDPDDFPLLITYWEKTFKIDLFRPQIGVVNVTDADSVWMDIRDDDDLPTAE
ELEDWIEDVLSGKINTEDDDDDEDDDDDDDDDDDDDDDDDDDDDDDDDDD*

9.12 *Pyrgilauda ruficollis* (rufous-necked snowfinch)

From: Trinity *de novo* assembly of SRR18552123. CASQ1 from complement of TRINITY_DN366_c1_g2_i14. CASQ2 from TRINITY_DN5611_c0_g2_i1.

>CASQ1_Pyrgilauda_ruficollis_transcript_assembly
TCTTCATCATCTTCCTCATCTTCATCCTCTCCCTGTCCCCTCTCCCTCTCCCTCTCCCTCTCCCTGTCCCTGTCGCTGTCCCCATGTCC
CCTCTGTGGCCCTGCCGGTGGCTCCTGGCGCTGTTGGCCCTGGTCCCGGGGGTCCTCCCGGGGGTCCCGGGGGTCCCGGGCTGTCCCGG
GGGGGCTCTGTCGCTGCCGCCCCCGGACGGGCGGAGCCGCCTCCTCCCGGTGCCCCCCGGGACCCTCCCCGAGCTGCGCCGCCGCTTCC
CGGTGCTGGCGCTGCTGCGGAGCCCCGGGCACCGGGAGCACCGGCCCGAGGAGCTGCGGGCGCTCGAGCTGGTGGCGCAGGCGCTGGAG
CCCCGCGGGGTCGGCTTCGGCATGGTGGGCACGGAGAGCGACCCCGAGCTGGCCCGGGACTGGGGGCTGGGGGGGCGCTGGCGCCTGGC
GCTGCTGCGGGGACACTCGGTGACAGAGTACCCCGGGGAGCTGGCAGTGCCAGCCCTGCTGTGCTTCCTGAGCCAGGCGCTGCCCCCCC
CCGTGCAGGTGCTGGTGGCCGGGCCGCAGCTCGGGGACTTTGGGGACATCGAGGAGGAGCCCAAAGCCATCGGCTTCTTCCGGGGACCC
GAGTCACGGGCTTTCCAGTCCTTCTCGGCCGCCGCCCGCCGCCTCCACCCCTCCCTCCTCTTCTTCGCCACCTTCGACCCCGAGGCGGC
GCGGGCGCTGGGGCTGCGCCCCGAGCGGGTTTTGCTCTTCGAGCCCTTCCTGGAGCGGCCCCGGCGCTTCCGGGGGGACCCCCGGGACA
CCCCCCAGATGGAGGCCTTCCTGCAGCGCGGCCGGCGGGCCACTCTGCGGAAACTCAAAGCTCAGAGCATGGCCGGGAGCTGGGAGGAC
GCCCTGGACGGGACCCCCATCGTGGCCTTCGCCAAGGGGGATGATCCCGACGGGTTCGAGTTCCTGGAGACCCTGAAGGAGGTGGCCCA
GGCCAGGAGGGACCGGCCCGGCTTCGGCGTCCTCTGGATCGACCCCGGCGACTTCCCGGGGCTCGTCCCCTCGTGGGAGGACACCTTTG
ACATCGACCTGTCCCGGCCACAGCTGGGCGTGGTCAATGGCACCGACCACGCCAGCAGCGTGTGGCTGCACATGGAGCACGAGGAGGAC
CTGCCGGGGCCCGAGGAGGTCCTGGAGTGGCTCCAGGAGGTCCTGGAGGGGGACACCGGGGACACCGGGGACGACGGTGAGGGTGACGA
TGAAGATGATGATGATGATGAAGAAGATGAAGATGATAAAGATGATGAAGAAGATGGAGATGAAGATGATGAAGATGAGGAAGATGAGG
AAGATGCTGAAGATGAGGACGATGAGGGAGATGATGAAGATGA

>CASQ1_Pyrgilauda_ruficollis_CDS_partial
ATGTCCCCTCTGTGGCCCTGCCGGTGGCTCCTGGCGCTGTTGGCCCTGGTCCCGGGGGTCCTCCCGGGGGTCCCGGGGGTCCCGGGCTG
TCCCGGGGGGGCTCTGTCGCTGCCGCCCCCGGACGGGCGGAGCCGCCTCCTCCCGGTGCCCCCCGGGACCCTCCCCGAGCTGCGCCGCC
GCTTCCCGGTGCTGGCGCTGCTGCGGAGCCCCGGGCACCGGGAGCACCGGCCCGAGGAGCTGCGGGCGCTCGAGCTGGTGGCGCAGGCG
CTGGAGCCCCGCGGGGTCGGCTTCGGCATGGTGGGCACGGAGAGCGACCCCGAGCTGGCCCGGGACTGGGGGCTGGGGGGGCGCTGGCG
CCTGGCGCTGCTGCGGGGACACTCGGTGACAGAGTACCCCGGGGAGCTGGCAGTGCCAGCCCTGCTGTGCTTCCTGAGCCAGGCGCTGC
CCCCCCCCGTGCAGGTGCTGGTGGCCGGGCCGCAGCTCGGGGACTTTGGGGACATCGAGGAGGAGCCCAAAGCCATCGGCTTCTTCCGG
GGACCCGAGTCACGGGCTTTCCAGTCCTTCTCGGCCGCCGCCCGCCGCCTCCACCCCTCCCTCCTCTTCTTCGCCACCTTCGACCCCGA
GGCGGCGCGGGCGCTGGGGCTGCGCCCCGAGCGGGTTTTGCTCTTCGAGCCCTTCCTGGAGCGGCCCCGGCGCTTCCGGGGGGACCCCC
GGGACACCCCCCAGATGGAGGCCTTCCTGCAGCGCGGCCGGCGGGCCACTCTGCGGAAACTCAAAGCTCAGAGCATGGCCGGGAGCTGG
GAGGACGCCCTGGACGGGACCCCCATCGTGGCCTTCGCCAAGGGGGATGATCCCGACGGGTTCGAGTTCCTGGAGACCCTGAAGGAGGT
GGCCCAGGCCAGGAGGGACCGGCCCGGCTTCGGCGTCCTCTGGATCGACCCCGGCGACTTCCCGGGGCTCGTCCCCTCGTGGGAGGACA
CCTTTGACATCGACCTGTCCCGGCCACAGCTGGGCGTGGTCAATGGCACCGACCACGCCAGCAGCGTGTGGCTGCACATGGAGCACGAG
GAGGACCTGCCGGGGCCCGAGGAGGTCCTGGAGTGGCTCCAGGAGGTCCTGGAGGGGGACACCGGGGACACCGGGGACGACGGTGAGGG
TGACGATGAAGATGATGATGATGATGAAGAAGATGAAGATGATAAAGATGATGAAGAAGATGGAGATGAAGATGATGAAGATGAGGAAG
ATGAGGAAGATGCTGAAGATGAGGACGATGAGGGAGATGATGAAGATGA

>CASQ1_Pyrgilauda_ruficollis_protein_partial
MSPLWPCRWLLALLALVPGVLPGVPGVPGCPGGALSLPPPDGRSRLLPVPPGTLPELRRRFPVLALLRSPGHREHRPEELRALELVAQA
LEPRGVGFGMVGTESDPELARDWGLGGRWRLALLRGHSVTEYPGELAVPALLCFLSQALPPPVQVLVAGPQLGDFGDIEEEPKAIGFFR
GPESRAFQSFSAAARRLHPSLLFFATFDPEAARALGLRPERVLLFEPFLERPRRFRGDPRDTPQMEAFLQRGRRATLRKLKAQSMAGSW

EDALDGTPIVAFAKGDDPDGFEFLETLKEVAQARRDRPGFGVLWIDPGDFPGLVPSWEDTFDIDLSRPQLGVVNGTDHASSVWLHMEHE
EDLPGPEEVLEWLQEVLEGDTGDTGDDGEGDDEDDDDDEEDEDDKDDEEDGDEDDEDEEDEEDAEDEDDEGDDEDX

>CASQ2_Pyrgilauda_ruficollis_transcript_assembly
CTTTGTTGTGAGTGCAGGACAGCAAAGTTTGCTGCTCCCAACCAGCCTTGCAGTGTTTGGGCTCAAGTCTAGGAAAGCAGCAGAGAGAA
AGAGAAACTAAGAGAAACTTAGTTCAAACTTCACCTGGAGTCTTTGGTCTCCCTCTCTTCTCAAATTTAGAAGCAGGGGGACATTTACC
CACCCTTTGCTGCACTCCAAGGGTGCCCCTATGAAGGCAATTTGCTGGATCCTGGCAGGTTTTTACCTGCTTTTCTGCTGCAAGGCAGA
AGAGGGACTGAACTTCCCTACTTACGATGGGAAAGACCGAGTGATCGACCTGAACGAGAAGAATTACAAGCAGGCACTGAAGAAGTATG
ACATGCTCTGCCTGCTCTTCCATGAGCCTGTGGGCTCTGACAAGGTCTCCCAGAAGCAGTTCCAGATGACAGAGATGGTCCTGGAGCTG
GCAGCTCAGGTCCTGGAGCCCAGGAGCATTGGCTTTGGGATGGTGGACTCCAAGAAGGATGCCAAGCTTGCCAAAAAGTTAGGCTTGCT
TGAAGAGGGAAGTCTCTATGTCTTTAAGGATGAGCGGTTGGTTGAATTTGATGGGGAACTGGCTGCAGATGTCTTGGTGGAATTCCTCT
TGGATTTGCTGGAAGACCCCGTGGAGGTCATCAACAGCAAGCTGGAGCTTCAGGCCTTTGACCAGATCGACGACGAAATCAAACTCATC
GGCTACTTCAAGGGAGAAGACTCAGAACATTTCAAGGCATTTGAAGAAGCTGCTGAACATTTCCAGCCCTATGTCAAGTTCTTTGCCAC
CTTTGATAAAGGGGTTGCCAAGAAGCTAGGCCTAAAGATGAACGAGGTGGACTTCTATGAACCCTTTATGGATGAGCCTGTTCACATCC
CTGATAAGCCTTACTCAGAAGAGGAGCTGGTGGATTTTGTGAGAGAGCACAGAAGGGCCACCTTGAGGAAGCTGCGCCCAGAGGACATG
TTTGAGACGTGGGAGGATGACATGGAAGGTATTCACATTGTAGCCTTTGCTGAAGCAGATGACCCAGATGGTTTTGAGTTCCTGGAAAT
CCTGAAGCAGGTTGCCAGGGACAACACCGATAATCCCGACCTGAGCATCGTCTGGATTGACCCTGACGACTTTCCTTTGCTCATCACGT
ACTGGGAGAAGACCTTCAAGATCGACCTGTTCAGACCACAGATCGGGGTGGTGAACGTCACTGACGCTGACAGCGTCTGGATGGACATC
AGAGATGATGACGACCTGCCCACAGCCGAGGAGCTGGAGGACTGGATAGAGGACGTGCTTTCCGGGAAGATAAATACCGAAGATGACGA
CGACGATGAAGATGACGACGATGATGATGACGACGATGATGATGACGATGACGATGATGATGACGACGATGACGATGATGACGATGATG
ACGACTAACTGTGACTCTGTGCAGTTTGACTTGTGGGGCCCGAGAGCCCTGGCCCTGCGGCAGGTCCCTCCATTTGAAGACCTATGTTT
GAGCATCAGCAAGCACCGCTGCTCCTCTTTATCCCCGGCCCCGCTCCCCCTGCCCTCGCCCTTGCCGGGAGCCCCTTGCCTGATTCCCA
GCGGTGCTGAGCCAGCGGGACTGCCCCTGCGGAGGGCAGCACCTGCAGGCACCCAGCACCGCTGCAAATCATGCCTCCTTAGTAAGGAC
AGTAGGAATCTGCAGGGAATCTGCCCCGAGTGTCCCAGGGCAGGAGAAAAGTGCCTGCCTGCTTGCTGCCAGAGGGACGCAGGGAGCCA
GTTTGTATTTTCTGTGCTCATCAGCCCAGCACTTAACATCCAAAGACCAAATCTCACTTCAAGACGTAGGGAAAACCCTAACTGTAAAG
TTTAGCCCTTTTATTAAACACAATGCCCTTTGCAGCTACTCTGCCAAGACCACTCAATCCTGCAAGTTCTGCTGCGGGAGTCTGTAGCT
ACTCCCTAACATGCTAAGAGCCAGAAAAATGCTTTGGGAGGGGAGGGAGGTTTTAGGGATGCTATTAAAGTTTTCAGTGATTCCACATA
ATGACAGGATATTCTTGTCTGGCAGTGCAATGCAAAACCAAACACCGGGTCCCCTGTCTGAACTTCTTTCTTCTTGAAAGCTGAGGCCA
CAGCTGGAGTCAGTGCGGGGGCCCTGATATGAGACACAAAGCTCATGAGTTCCTGAACTGCTGGACAGGCTGGTCCAGCTCGGAGCCCA
GTGTGAGTACAAACCACTGAGTCTGAAGATCACGGCACTGAGCCAAGCCAATGGGAACATCCACAGGGGCCCACCCTGAAGCACTTCTA
TTCCCCATGACCTTCCAGCTTTTGCTGACTCTTCACCCAAAGCCAGTGTAGCATCTTTTCCCATGATCAAGATGTCAGGAGGGAACACA
TCCATCCAAACATATTAGATAGTGGATGAGTTGCATTGGGGTGTGAGCAGCGGAGGGATTCAAGCTGTTTGGCTTTTGAGTACTTAGTT
GCAGTTACTCCCTGCTTAGAGACTCAGATATCCCATGTGAGAAGCCCCTTTTGGCTGCAGGCTGAGAAGGAGTTCAGGGTACCAGTTTG
CAGTCAAGGGGGTCACTCACAAAGCCCAGGGTCCGTACCTCGGTCCTGGCGAGTGCAGCACCATCCATGTGCAGGTCACTGTGGCAGCA
TTAGATCTCCCTGCTTTCCCGTGCTGGCAGATTTCAATTGTCTTAGCTTCTTATTAGTTTGGCAATAGTGCAATGATTGGAGAGTTCAG
AGATGGTGTAAAATACATTTATAAAGATGTTCTGCAGTTGTTCAGAGTTGCTAGCATAGGTGGTGTACACATCTCCTCTCTTGCTACAA
ACAAAAAAACATTGTTCTGACCATCCTTATACTGCCTTAGGACAGAGGTACATGATGTGAGGGGAAATAACAGTGTGAGGCACAGAAGA
ATCAGTTACACAAATGCGAATCTTGATGAGAAACAAGGTTTACACACTATATATTTCCTTTATCAGATGAACTGCAAAGCAAGATTTTA
TAGATTTTTTTCCTGCCTGTTGATAAGTTTCTTTGACTCCCTCTCTCTACATTAGCTGAAATAGAAATTTGAAACTACTGATATGAAAA
AGCTGAAGTATTGATATGTTCCCATGTGTGCCATATCCCCTTGGTGGAAAAT

>CASQ2_Pyrgilauda_ruficollis_CDS
ATGAAGGCAATTTGCTGGATCCTGGCAGGTTTTTACCTGCTTTTCTGCTGCAAGGCAGAAGAGGGACTGAACTTCCCTACTTACGATGG
GAAAGACCGAGTGATCGACCTGAACGAGAAGAATTACAAGCAGGCACTGAAGAAGTATGACATGCTCTGCCTGCTCTTCCATGAGCCTG
TGGGCTCTGACAAGGTCTCCCAGAAGCAGTTCCAGATGACAGAGATGGTCCTGGAGCTGGCAGCTCAGGTCCTGGAGCCCAGGAGCATT
GGCTTTGGGATGGTGGACTCCAAGAAGGATGCCAAGCTTGCCAAAAAGTTAGGCTTGCTTGAAGAGGGAAGTCTCTATGTCTTTAAGGA
TGAGCGGTTGGTTGAATTTGATGGGGAACTGGCTGCAGATGTCTTGGTGGAATTCCTCTTGGATTTGCTGGAAGACCCCGTGGAGGTCA
TCAACAGCAAGCTGGAGCTTCAGGCCTTTGACCAGATCGACGACGAAATCAAACTCATCGGCTACTTCAAGGGAGAAGACTCAGAACAT
TTCAAGGCATTTGAAGAAGCTGCTGAACATTTCCAGCCCTATGTCAAGTTCTTTGCCACCTTTGATAAAGGGGTTGCCAAGAAGCTAGG
CCTAAAGATGAACGAGGTGGACTTCTATGAACCCTTTATGGATGAGCCTGTTCACATCCCTGATAAGCCTTACTCAGAAGAGGAGCTGG
TGGATTTTGTGAGAGAGCACAGAAGGGCCACCTTGAGGAAGCTGCGCCCAGAGGACATGTTTGAGACGTGGGAGGATGACATGGAAGGT
ATTCACATTGTAGCCTTTGCTGAAGCAGATGACCCAGATGGTTTTGAGTTCCTGGAAATCCTGAAGCAGGTTGCCAGGGACAACACCGA
TAATCCCGACCTGAGCATCGTCTGGATTGACCCTGACGACTTTCCTTTGCTCATCACGTACTGGGAGAAGACCTTCAAGATCGACCTGT
TCAGACCACAGATCGGGGTGGTGAACGTCACTGACGCTGACAGCGTCTGGATGGACATCAGAGATGATGACGACCTGCCCACAGCCGAG
GAGCTGGAGGACTGGATAGAGGACGTGCTTTCCGGGAAGATAAATACCGAAGATGACGACGACGATGAAGATGACGACGATGATGATGA
CGACGATGATGATGACGATGACGATGATGATGACGACGATGACGATGATGACGATGATGACGACTAA

>CASQ2_Pyrgilauda_ruficollis_protein
MKAICWILAGFYLLFCCKAEEGLNFPTYDGKDRVIDLNEKNYKQALKKYDMLCLLFHEPVGSDKVSQKQFQMTEMVLELAAQVLEPRSI
GFGMVDSKKDAKLAKKLGLLEEGSLYVFKDERLVEFDGELAADVLVEFLLDLLEDPVEVINSKLELQAFDQIDDEIKLIGYFKGEDSEH
FKAFEEAAEHFQPYVKFFATFDKGVAKKLGLKMNEVDFYEPFMDEPVHIPDKPYSEEELVDFVREHRRATLRKLRPEDMFETWEDDMEG

IHIVAFAEADDPDGFEFLEILKQVARDNTDNPDLSIVWIDPDDFPLLITYWEKTFKIDLFRPQIGVVNVTDADSVWMDIRDDDDLPTAE
ELEDWIEDVLSGKINTEDDDDDEDDDDDDDDDDDDDDDDDDDDDDDDDDDD*

9.13 *Taeniopygia castanotis* (Australian zebra finch)

From: Trinity *de novo* assembly of SRR9671221. No CASQ1 found. CAS2 from TRINITY_DN539_c1_g2_i3.

```
>CASQ2_Taeniopygia_castanotis_transcript_assembly
CTGACATCTTCCACTCCCTCTGTCTTGCCCACTCCCTCTCTCTCAGATGATGCAGAGTGCCAGCAAGTCCCTCAATGGTGTCATATCTG
TCCTTTTTATTCATTGCAGGGTTTATTTTTAGCCTGAAACAACTGCTTCCTAAAAATGGAGTTCCTAATGACACGGGAGCTGGACACAG
CTATGTAAGGTATCCAGGGCTTGGCCTGACAGCTTCTCCCGTCTGTCTCTCTCTTTGTTGTGAGTACAGGACAGCAAAGTTTGCTGCTC
CCCACCAGTCTTGCAGTGTTTGGGCTCAACACTAGGAAAGCAGCAGAGAGAAACTTAGAGAAACTAAGAGAAACTTAGTTCTAACTTCT
CCTGGAGTCTTTGGTCTCCCTCTCTTCTCAAATTAAGAAAGAGGGGGACATTTAACCACCCTTTGCTGCAGTCCAAGGGTGCCCCTATG
AAGGCAGTTTGCTGCATCCTGGCAGGTTTTTACCTGCTTTTCTGCTGCAAGGCAGAAGAGGGACTGAACTTCCCTACTTACGATGGGAA
AGACCGAGTGATCGACCTGAACGAGAAGAATTACAAGCAGGCACTGAAGAAGTATGACATGCTCTGCCTGCTCTTCCATGAGCCTGTGA
GCTCTGACAAGGTCTCCCAGAAGCAGTTCCAGATGACAGAGATGGTCCTGGAGCTGGCAGCTCAGGTCCTGGAGCCCAGGAGCATTGGC
TTTGGGATGGTGGACTCCAAGAAGGATGCCAAGCTTGCCAAAAAGTTAGGCTTGCTTGAAGAGGGAAGTCTCTATGTCTTTAAGGACGA
GCGGCTGGTTGAATTTGATGGGGAACTGTCTGCAGATGTCTTGGTGGAATTCCTCTTGGATTTGCTAGAAGACCCCGTGGAGGTCATAA
ACAGCAAGCTGGAGCTTCAGGCCTTTGACCAGATCGATGACGAAATCAAACTTATCGGCTACTTCAAAGGAGAAGACTCAGAACATTTC
AAGGCATTTGAAGAAGCTGCTGAACAATTCCAGCCCTACGTCAAGTTCTTTGCCACCTTTGACAAAGGGGTTGCCAAGAAGCTGGGCCT
AAAGATGAACGAGGTGGACTTCTATGAACCCTTTATGGATGAGCCTGTTCACATCCCTGATAAGCCTTACTCAGAAGAGGAGCTGGTTG
ATTTTGTGAGAGAGCACAGAAGAGCCACCTTGAGGAAGCTGCGCCCAGAGGACATGTTTGAGACGTGGGAGGATGACATGGAGGGTATT
CACATTGTAGCCTTTGCTGAAGAAGATGACCCAGATGGTTTTGAGTTCCTGGAAATCCTGAAGCAGGTTGCCAGGGACAACACCGATAA
TCCCGACCTGAGCATTGTTTGGATTGACCCTGATGACTTTCCTTTGCTCATCACTTACTGGGAGAAGACCTTCAAGATCGACCTGTTCA
GACCACAGATTGGGATAGTGAACGTCACGGATGCTGACAGCGTCTGGATGGACATCAGAGACGATGACGACCTGCCCACAGCCGAGGAG
CTGGAGGACTGGATAGAGGACGTGCTTTCTGGGAAGATAAATACTGAAGATGATGACGATGATGAAGATGATGATGATGATGATGACGA
CGACGATGATGACGATGATGATGATGACGATGATGACGACGATGATGATGACGATGATGACTAACTGTGACTCTGTGCAGTTTGACTTG
TGGGGTCCGAGAGCCCTGGCCCTGTGGCAGGTCCCTCCATTGGAAGACCTAAGTTTGAGCATCAGCAAGCACCACTGCTCCTCTTTTTC
CCCCGGCCCCGCTCCCCCTGCCCTCGCCCTTGCCGGGACCCCCTCGCCTGATTCCCAGCGGTGCTGAGCCACCGGGACTGCCCCTGCGG
AGGGCAGCACCTGCAGGCACCCAGCACCGCTGCAAATCATGCCTCCTTAGTAAGGACAGTAGGAATCTGCAGGGAATCTGCCCCGAGTG
TCCCAGGGCAGGAGAAAAGTGCCTGCCTGCTTGCTGCCAGAGGGACACAGGGAGCCAGTTTGTATTTTCTGTGTTCATCAGCCCAGCAC
TTAACATCCAAAGACCAAATCTCACTTCAAGACATAGGGAAAACCCTAACTGTAAAGTTTAGCCATTTTATTAAACACAATGCCCTTTG
CAGCTACTCTGCCAAGACCACTCAATCCTGCAAGTTGTGCTGCGGGAGTCTGTAGCTACTCCCTAATATGCTAAGAGCCAGAAAAATGC
TTTGGGAGGGGAGGGAGGTTTTAGGGATGCTATTAAAGTTTTCAGTGATTCCACATAATGACAGGATATTCTTGTCTGGCAGTGCAATG
CAAAATCAAACACCGGGTCCCCTGTCTGAACCTCTTTCTTCTTGAAAGTTCAGGCTACAGCTGGAGTCAGTGCAGGGGCCTTGATATGA
GACACAAAACCCCTGAGTTCCTGAACTGTTGGACAGGCTGGTCCAGCTTGGGGCCCAGTTGAGTCCAAACCACTGAAGATCATGGCACT
GAGCCAAGCCAATGGAAATATCCACAGGAGCCCACCCTGAAGCACTCCTATTCCCCATGACCTTCCAGCTTTTGCTGACTCTTCACCCA
AAGCCCATGTAGCATCTTTTCCATGACTGAGGTGTCAGGAGGGAACAGATCCATCCAAACATATTAGATAGTGGATGAGTTGCATTGGG
GTGTGAGCAGCAGAGGGATTCAAACTGTTTGGCTTTTGAGTATTTAGTTGCACAGTTACTCCCTGCCTAGAGACTCAGGTATCCCATGT
GAGAAGCCTCTTTTGACTGCAGGCTGAGAAGGAGTTCAGGTACCAGTCAAGGGGGTCACTCACAAAGCCAGGGGTCCATCCCACGGTCC
TGGTGAGTGCAGCACCATCCATGTGCAGGTCACTGTGGCAGCATTAGGTCTCCCTGCTTTCTCATGCCGGCAGATTTCAATTGTCTTAG
CTTCTTATGTTTGACAATAGTGCAATGAATGGAGAGTTCAGAGATGGTGTTATCTTTATAAATTCATTTATAAATACATTTATAAAGAT
GTTCTCCAGTTGTTCAGAGTTGCTAGCATAAGTAGTGTACACATCTCCTCTCTTGCTACAAACAAAAAACATTGTTCTGACCATCCTTA
TACTGGCTTAGGACAGAGGTATGTGATGTGAAGGAAAATTACAGTGTGAGGGACAGAAGAATCAGAGTTACACAAATGTGACTCCGCCT
CATGAGAAACAAGGTTTACACATTATATATTTCCTTTATCGGGTGAACTGCAAAGCAAGATTTTATAGATTTTTTTCCTGCCTGTTGAT
CAGTTTCGTTTACTCTCTCTCTCTACATTAGCTGAAATAGAAATTTGAAATTACTGGTATAAAAAAGCTGAAGTATTGATATGTTCCCA
TGTGTGCCATATCCCCTTGCTGGAAAATACCAGTATGAACAACACATAGCTGGATTTCTCTCTGTCTCTATGCACTTGAATGAATACTG
TACTAGCATGGTAGATGACTGTAGGCCAAGTTAGCTCCTTGCTGAGCAGCAAACCCTCAACATGTGCTTTTCACATACAGATTGTTAGA
AGAACAGACAAGGCAGCAAAGCCCCCAGGTCAACCATTTGCTCTTCCGTGCTTTTTTATGGTTGCCTAGAAGTTGAACCAAGTTTGTTC
AGAGAGAACAATCAACTGGTTGCTCTGGGATGATAAAAACTTTCCAAAGTCTTGAAATTCAGTATGCTCTCAGCATGGAGGTCTCCTCC
TCTCTTCCACTCACACCCTACTTTCTGCTGGTTGACAGAACACCAGCATTCTCCTTCTGCTTTTTCATCAAGGTCTGCTGGAGGATCCC
ACCTCAGAGAGGGAGCGAGCAGGACTCAAGGTCTCATTCACCCACACTGGTAAGGAGCAAGGAGAGGCAGACATAGCCCTCTGGTAGTC
TCCTGGCTTGTGATTAAGGGACAGCTACTGACCTGAGAAAAGAGCTTATCTTTACCTTTGGGCAATTCCCAGGAACAAAACATGCACAG
TTTCACCCTGCATAAGATAACAGCCTACACTGGCCATGTCCCTCCTCATCAGTCACTCCAAACACAAAGAATAACGTAAAGCTCCTCTC
CTGCCTTTCCTAACTGGCACAATCTCCAGATGTGCCGAGTGCCAGAGTGCTTTCTGTGTGCTCCTCTCCCACTGTGGATGCCCCTCTGT
TCTGAAGTGCCCCACTCTAGGTGCTCTGAGAGGCACAGATTACCCAGGCAGACCCTGCAGACAGCACTAGGGACACGTTCAAAGTGCAC
AGGTGTTTCACAGGCACTTGAACATGTCAATGCAACAGACCTCTAGGGGGAAAAGACATAAATAATGTAGAAGTGCAGAGGCTGAACTC
ATACTGGGGAGTGGTGCCAGTGGTTTATGGAAAGTTTCCCTTGAGTTATGCAGGGAAACAGCTAATCCTACAGCTGCCACTCCTTCATA
GTGCCAGGAATGGGGCTGCCCTTTCTCTCTTCTCTCAGTCTGCCATTACAGTTCTCAGCACTGTCTTTGCCCACAAACAGGAACTGTTT
ATCACCAGCTTTCAGGACAATCACACAAAAAGTTAGGAAAGCATTGCCACAGAGGAAGGATTGCAGCAATGAGGATGGCTGGACAGCAA
AGATTTTCCCATTTAACTACAACACCTTGTCTGCCCCAAAATCCTAATGACCAAAGAGCTGAAGGACACAATGGCCCTTGTGAAGGAGA
```

GGGCAGCTTGAATTTGAGTGTTTTGCTTGCAGGGACAGGTAAGACAGTGGTATGGGAATGACACACAGGACCCGAGGCCCAGGTGCTCC
TGGGCTCTTCTATCACAAATAGGAACACCTGGCATTTGAAAGGCACTGGTGGCCCTGAGGAACAAAGCCAGCCTAACCCTGCCCACTAT
ACACTGAGAAGAAACAGTCAGCACATTTGGATGAGGAACCTGCAAAATCCGCTTTATTAATAAAATACAACTTTATGTACGTGGGCAGC
ACTGAATGCTTAACAAGCACGTCTCTCTTCACAAATAATTCTTTTGCTCTAGCATGTTACTAGACATATTAAACAGGTCTCTATACAGA
TAAAGACAGGAATTGTATTTTGCCCTCATCCAACCATGAGTGCTTCAAGCTGATCAGTTTCTTCAAAGAACCATTCAATCATTTTTGTT
GGAAAAGACCCCTAAGATCATCAAGTCTGACCATTAGTCCACTACTGTCAAGTTAATACCCAAAGTACTCCAAAACCTGGCCTTGTATC
TCCCCAGTGACAGTGAAGATCCTCCTAACACGTCATATGTGAAAAGAACACCAAGTTATGCAGAACCTCCTCGCAGTCCCCAGACACAG
GGCTGAAGGGGTTTGTTTGGGGGTGCTGTACCACAGCACAGCCTGTGCTGCTCATAGGCAACAGTCAAGGGAGAAGGAAACCCACCAGG
AGCAGCACGCTGCAGCTCTCATACTGCAGCATGGAGGAAAAAGTTTCTGCCTTTGCAGCAGTCGCTGATGTAGAGGTTCTGTGAACCCA
AAAGTGCTCCGAGTTTATGTCAACCATTTGGGAAACAGCTGCAAGAGGAAAGGACCAGGAAGGCACTCTGGAAATCGCAGACTACTCCA
TGTTTTCAGGGTCAGCAGTTTTTGTGATGTTTCTGAGGAGTGATGAGTCTCCTGTCTCCCTTGGGAAAGGAATGGCCCCATAGTGAAAC
CAGAGATCTCAGCTGCTTCCCGTCCCAGAATGAAGCTGCACAGCAACATGAACACAGAGCCGAGGACACCGGCTGCTTCTCCGTGTCTG
TCTGCACATCATACCTGAAGAAATGATGGATTCATTACAGAGACCAAACACAAAACATTGCCTTTTATTCCCTGGAAAATTATTAATTA
GACAGGATCCCAGCTGCCACTGACTTACAGTCTTGTGAAAAGAGGCCAAGATTGCAGCTGGTTTCCTCTCCACTGCAGCAAAGCTTTGG
CTTTGAGGAAGCCCTGCATGCAGCCAGTTGCACACTGATGCCTGGACTTGGGGGACTGAAGAGAAGGATGGGAAAAAGGCAAGAAGCAC
CCCAGCCCTCTGAGGCAAAGCTCACTGCCTCCTGGCTGCTTTCACACCCCAGGTACATCCATGCCGCACAGTGCAAGACCAGCTGCTAG
AAACTGGAAAGAGCAGAGCACAGCAGGACGGATAACTGACCTCGGCAGTGAGGCAGAGCCCTGTACCAGCAGCTCTGTGTCCTACCTGG
GACCCAAGACTCTGGGTCAACCCACTGCACTGCAGCATCCCTTGCCTTCTCCCCACTCCCTTGTACCACTGACCAAGGGCAAGGCAGCC
CTTGGCTGGTCAGTACCCCTGGGGGACAGCCGTGGGATGACTCTCGCTCCTCTTTGACAGAAGATGACATCTCATGACAATGGGGACTA
TGGGGTAGGGCCCAGGGCAGAGACTTGGCCCAGCTCCCAGAACACTCCGCCAGGTTTGAAAGGTGCTAAGCTGTTTAAAGGGAAAAAAA
AAAAAAAAAAAAAAAACTAAAGAGCCTCCCTGGCGGCAGGAAACCCATTTT

>CASQ2_Taeniopygia_castanotis_CDS
ATGAAGGCAGTTTGCTGCATCCTGGCAGGTTTTTACCTGCTTTTCTGCTGCAAGGCAGAAGAGGGACTGAACTTCCCTACTTACGATGG
GAAAGACCGAGTGATCGACCTGAACGAGAAGAATTACAAGCAGGCACTGAAGAAGTATGACATGCTCTGCCTGCTCTTCCATGAGCCTG
TGAGCTCTGACAAGGTCTCCCAGAAGCAGTTCCAGATGACAGAGATGGTCCTGGAGCTGGCAGCTCAGGTCCTGGAGCCCAGGAGCATT
GGCTTTGGGATGGTGGACTCCAAGAAGGATGCCAAGCTTGCCAAAAAGTTAGGCTTGCTTGAAGAGGGAAGTCTCTATGTCTTTAAGGA
CGAGCGGCTGGTTGAATTTGATGGGGAACTGTCTGCAGATGTCTTGGTGGAATTCCTCTTGGATTTGCTAGAAGACCCCGTGGAGGTCA
TAAACAGCAAGCTGGAGCTTCAGGCCTTTGACCAGATCGATGACGAAATCAAACTTATCGGCTACTTCAAAGGAGAAGACTCAGAACAT
TTCAAGGCATTTGAAGAAGCTGCTGAACAATTCCAGCCCTACGTCAAGTTCTTTGCCACCTTTGACAAAGGGGTTGCCAAGAAGCTGGG
CCTAAAGATGAACGAGGTGGACTTCTATGAACCCTTTATGGATGAGCCTGTTCACATCCCTGATAAGCCTTACTCAGAAGAGGAGCTGG
TTGATTTTGTGAGAGAGCACAGAAGAGCCACCTTGAGGAAGCTGCGCCCAGAGGACATGTTTGAGACGTGGGAGGATGACATGGAGGGT
ATTCACATTGTAGCCTTTGCTGAAGAAGATGACCCAGATGGTTTTGAGTTCCTGGAAATCCTGAAGCAGGTTGCCAGGGACAACACCGA
TAATCCCGACCTGAGCATTGTTTGGATTGACCCTGATGACTTTCCTTTGCTCATCACTTACTGGGAGAAGACCTTCAAGATCGACCTGT
TCAGACCACAGATTGGGATAGTGAACGTCACGGATGCTGACAGCGTCTGGATGGACATCAGAGACGATGACGACCTGCCCACAGCCGAG
GAGCTGGAGGACTGGATAGAGGACGTGCTTTCTGGGAAGATAAATACTGAAGATGATGACGATGATGAAGATGATGATGATGATGATGA
CGACGACGATGATGACGATGATGATGATGACGATGATGACGACGATGATGATGACGATGATGACTAA

>CASQ2_Taeniopygia_castanotis_protein
MKAVCCILAGFYLLFCCKAEEGLNFPTYDGKDRVIDLNEKNYKQALKKYDMLCLLFHEPVSSDKVSQKQFQMTEMVLELAAQVLEPRSI
GFGMVDSKKDAKLAKKLGLLEEGSLYVFKDERLVEFDGELSADVLVEFLLDLLEDPVEVINSKLELQAFDQIDDEIKLIGYFKGEDSEH
FKAFEEAAEQFQPYVKFFATFDKGVAKKLGLKMNEVDFYEPFMDEPVHIPDKPYSEEELVDFVREHRRATLRKLRPEDMFETWEDDMEG
IHIVAFAEEDDPDGFEFLEILKQVARDNTDNPDLSIVWIDPDDFPLLITYWEKTFKIDLFRPQIGIVNVTDADSVWMDIRDDDDLPTAE
ELEDWIEDVLSGKINTEDDDDDEDDDDDDDDDDDDDDDDDDDDDDDDDD*

9.14 *Zonotrichia albicollis* (white throated sparrow)

From: Trinity *de novo* assembly of SRR23585157. No CASQ1 found. CASQ2 from complement of TRINITY_DN3379_c0_g1_i1.

>CASQ2_Zonotrichia_albicollis_transcript_assembly
CATATCTGTCCTTTTTATTCATTGCAGGGTTTATTTTTAGCCTGAAACAACTGCTTCCTAAAAATGGAGTTCCTAATGACACGGGAGCT
GGACACAGCTATGTAAGGTATCCAGGGCTTGGCCTGACAGCTTCTCCCGTCTGTCTCTCTCTTTGTTGTGAGTACAGGACAGCAAAGTT
TGCTGCTCCCCACCAGCCTTGCAGTGTTTGGGCTCAACTCTAGGAAAGCAGCAGAGAGAAACTTAGAGAAACTAGAGAAACTTAGTCCA
AACTTTACCTGGAGTCCTTGGTCTCCCTCTCTTCTCAAATCAAGAAAGAGGGAGACATTTACCCACCCTTTGCTGCACTCCAAGGGTGC
CCCTATGAAGGCAATTTGCTGGATCCTGGCAGGTTTTTACCTGCTTTTCTGCTGCAAGGCAGAAGAGGGACTGAACTTCCCTACTTACG
ATGGGAAAGACCGAGTGATCGACCTGAACGAGAAGAATTACAAGCAGGCACTGAAGAAGTATGACATGCTCTGCCTGCTCTTCCATGAG
CCTGTGAGCTCCGACAAGGTCTCCCAGAAGCAGTTCCAGATGACAGAGATGGTGCTGGAGCTGGCAGCTCAGGTCCTGGAGCCCAGGAG
CATTGGCTTTGGGATGGTGGACTCCAAGAAGGATGCCAAGCTTGCCAAAAAGTTAGGCTTGCTTGAAGAGGGAAGTCTCTATGTCTTTA
AGGATGAGCGGTTGGTTGAATTTGATGGGGAACTGTCTGCAGATGTCTTGGTGGAATTCCTCTTGGATTTGCTGGAGGACCCCGTGGAG
ATCATCAACAGCAAGCTGGAGCTTCAGGCCTTTGACCAGATCGACGACGAAATCAAACTCATTGGCTACTTCAAGGGAGAAGACTCAGA
ACATTTCAAGGCATTTGAAGAAGCTGCTGAACAATTCCAGCCCTACGTCAAGTTCTTTGCCACCTTTGACAAAGGGGTTGCCAAGAAGC
TTGGCCTAAAGATGAATGAGGTGGACTTCTACGAACCCTTTATGGATGAGCCTGTCCACATCCCTGATAAGCCTTACTCAGAAGAGGAG

CTGGTTGATTTTGTGAGAGAGCACAGAAGGGCCACCTTGAGGAAGCTGCGCCCCGAGGACATGTTTGAGACGTGGGAGGATGACATGGA
GGGCATTCACATCGTAGCCTTTGCTGAAGAAGATGACCCAGATGGTTTTGAGTTCCTGGAAATCCTGAAGCAGGTTGCCAGGGACAACA
CCGATAATCCTGACCTGAGCATTGTCTGGATTGACCCTGATGACTTTCCTTTGCTCATCACTTACTGGGAGAAGACCTTCAAGATCGAC
CTGTTCAGACCACAGATCGGGGTGGTGAATGTCACAGATGCTGACAGCGTCTGGATGGACATCAGAGATGATGACGACCTGCCCACAGC
CGAGGAGCTGGAGGACTGGATAGAGGATGTGCTTTCTGGGAAGATAAACACCGAGGATGATGACGACGATGAAGATGACGACGATGATG
ATGATGACGACGATGACGACGATGACGATGATGACGACGATGATGACGATGATGATGACGACGACGACTAACCGTGACTCTGTGCAGTT
TGACTTGTGGGGCCCGAGAGCCCCAGCCCTGTGGCAGGTCCCTCCATCGGAAGACCTTTGTTTGAGCATCAGCAAGCACCGCTGCTCCT
CTCTTCCCCCGGCCCCGCTCCCCTGCCCTCGCCCTTGCCGGGAGCCCCTGACCTGATTCCCAGCGGTGCTGAGCCCCGGGACTGCCCCT
GCGGAGGGCAGCGCCTGCGGACGCCCAGCACCACTGGGAATCACGCCTCCTTAGCAAGGACTGCAGGAACCTGCAGGGAATTGCCCCAG
TGTCCCAGGGCAGGAGAAAAGTGCCTGCCTGCTTGCTGCCAGAGGGACGCAGGGAGCCAGTTTGTATTTTCTGTGTTCATCAGCCCAGC
ACTTAACATCCAAAGACCAAATCTCACTTCAAGAGATCGGAAGAGCGTCGTGTAGGGAAAGAGTGTAGGCGATAGTGTTGTGGTGGGGG
GGGGGGGGGGGGTGTG

>CASQ2_Zonotrichia_albicollis_CDS
ATGAAGGCAATTTGCTGGATCCTGGCAGGTTTTTACCTGCTTTTCTGCTGCAAGGCAGAAGAGGGACTGAACTTCCCTACTTACGATGG
GAAAGACCGAGTGATCGACCTGAACGAGAAGAATTACAAGCAGGCACTGAAGAAGTATGACATGCTCTGCCTGCTCTTCCATGAGCCTG
TGAGCTCCGACAAGGTCTCCCAGAAGCAGTTCCAGATGACAGAGATGGTGCTGGAGCTGGCAGCTCAGGTCCTGGAGCCCAGGAGCATT
GGCTTTGGGATGGTGGACTCCAAGAAGGATGCCAAGCTTGCCAAAAAGTTAGGCTTGCTTGAAGAGGGAAGTCTCTATGTCTTTAAGGA
TGAGCGGTTGGTTGAATTTGATGGGGAACTGTCTGCAGATGTCTTGGTGGAATTCCTCTTGGATTTGCTGGAGGACCCCGTGGAGATCA
TCAACAGCAAGCTGGAGCTTCAGGCCTTTGACCAGATCGACGACGAAATCAAACTCATTGGCTACTTCAAGGGAGAAGACTCAGAACAT
TTCAAGGCATTTGAAGAAGCTGCTGAACAATTCCAGCCCTACGTCAAGTTCTTTGCCACCTTTGACAAAGGGGTTGCCAAGAAGCTTGG
CCTAAAGATGAATGAGGTGGACTTCTACGAACCCTTTATGGATGAGCCTGTCCACATCCCTGATAAGCCTTACTCAGAAGAGGAGCTGG
TTGATTTTGTGAGAGAGCACAGAAGGGCCACCTTGAGGAAGCTGCGCCCCGAGGACATGTTTGAGACGTGGGAGGATGACATGGAGGGC
ATTCACATCGTAGCCTTTGCTGAAGAAGATGACCCAGATGGTTTTGAGTTCCTGGAAATCCTGAAGCAGGTTGCCAGGGACAACACCGA
TAATCCTGACCTGAGCATTGTCTGGATTGACCCTGATGACTTTCCTTTGCTCATCACTTACTGGGAGAAGACCTTCAAGATCGACCTGT
TCAGACCACAGATCGGGGTGGTGAATGTCACAGATGCTGACAGCGTCTGGATGGACATCAGAGATGATGACGACCTGCCCACAGCCGAG
GAGCTGGAGGACTGGATAGAGGATGTGCTTTCTGGGAAGATAAACACCGAGGATGATGACGACGATGAAGATGACGACGATGATGATGA
TGACGACGATGACGACGATGACGATGATGACGACGATGATGACGATGATGATGACGACGACGACTAA

>CASQ2_Zonotrichia_albicollis_protein
MKAICWILAGFYLLFCCKAEEGLNFPTYDGKDRVIDLNEKNYKQALKKYDMLCLLFHEPVSSDKVSQKQFQMTEMVLELAAQVLEPRSI
GFGMVDSKKDAKLAKKLGLLEEGSLYVFKDERLVEFDGELSADVLVEFLLDLLEDPVEIINSKLELQAFDQIDDEIKLIGYFKGEDSEH
FKAFEEAAEQFQPYVKFFATFDKGVAKKLGLKMNEVDFYEPFMDEPVHIPDKPYSEEELVDFVREHRRATLRKLRPEDMFETWEDDMEG
IHIVAFAEEDDPDGFEFLEILKQVARDNTDNPDLSIVWIDPDDFPLLITYWEKTFKIDLFRPQIGVVNVTDADSVWMDIRDDDDLPTAE
ELEDWIEDVLSGKINTEDDDDDEDDDDDDDDDDDDDDDDDDDDDDDDDD*
